# Supplementary material for: tRNA expression and modification landscapes, and their dynamics during zebrafish embryo development
Source: Nucleic Acids Res. 2024 Jul 11;52(17):10575–94. doi: 10.1093/nar/gkae595 (PMC11417395; doi:10.1093/nar/gkae595)
Supplement: gkae595_Supplemental_Files [file gkae595_supplemental_files.zip › Supplementary_Files_description.pdf]

Supplementary information

tRNA expression and modification landscapes, and their dynamics during zebrafish embryo development

Tom Rappol[1], Maria Waldl[1,2,3,4], Anastasia Chugunova[5], Ivo L. Hofacker[2,6], Andrea Pauli[5] and Elisa Vilardo[1*]

[1] Centre for Anatomy & Cell Biology, Medical University of Vienna, 1090 Vienna, Austria

[2] Department of Theoretical Chemistry, University of Vienna, 1090 Vienna, Austria

[3] Vienna Doctoral School in Chemistry (DoSChem), University of Vienna, 1090 Vienna, Austria

[4] Institute of Computer Science and Interdisciplinary Center for Bioinformatics, Leipzig University, D-04107 Leipzig, Germany

[5] Research Institute of Molecular Pathology (IMP), Vienna BioCenter (VBC),1030 Vienna, Austria

[6] Faculty of Computer Science, Research Group Bioinformatics and Computational Biology, University of Vienna, 1090 Vienna, Austria

DESCRIPTION OF SUPPLEMENTARY FILES

Supplementary File 1 Supplementary text related to methods, supplementary tables, supplementary figures, and supplementary references.

Supplementary File 2 Coverage plots with misincorporation signature like in Figure 2A of all 68 tRNA clusters in the mock samples. Each page displays the results of all replicates of all samples/time points analysed. The header indicates the identity of the cluster in terms of name, anticodon contribution, and ID like in Supplementary Table 1. Each plot header indicates the sample identity and the library ID as deposited in SRA. On the x-axis both the alignment position and the canonical position (in brackets) are indicated.

Supplementary File 3 Coverage plots with misincorporation signature like in Figure 2B of all 68 tRNA clusters in the DM samples. Each page displays the results of all replicates of all samples/time points analysed. The headers and x-axis are like in Supplementary File 2.

Supplementary File 4 Coverage plots with misincorporation signature like in Figure 2C of all 68 tRNA clusters in the BS samples. Each page displays the results of all replicates of all samples/time points analysed. The headers and x-axis are like in Supplementary File 2.

Supplementary File 5 Plots showing abundance dynamics within tRNA clusters like in Figure 3C for all 68 tRNA clusters. Individual tRNA references are labelled and plotted in coloured lines if representing a minimum fraction of mapped reads of 0.002, the rest being plotted as grey lines.

Supplementary File 6 Misincorporation fraction like in Figure 7 for all 68 tRNA clusters in the mock samples. Graphs are shown for tRNA nucleotide positions that have a misincorporation fraction equal or greater than 0.15 in at least one time point. Lines represent the mean of the biological replicates, which are indicated by square, circle and triangle.

Supplementary File 7 C-retention fraction, indicative of m^5C modification, like in Figure 9 for all 68 tRNA clusters in the BS samples. Graphs are shown for tRNA nucleotide positions that have a C-retention fraction equal or greater than 0.15 in at least one time point. Lines represent the mean of the biological replicates, which are indicated by square, circle and triangle.
