# Supplementary material for: tRNA expression and modification landscapes, and their dynamics during zebrafish embryo development
Source: Nucleic Acids Res. 2024 Jul 11;52(17):10575–94. doi: 10.1093/nar/gkae595 (PMC11417395; doi:10.1093/nar/gkae595)
Supplement: gkae595_Supplemental_Files [file gkae595_supplemental_files.zip › Supplementary_File_1_Zfish_tRNA_mods_v5-refs-R2.pdf]

Supplementary information

tRNA expression and modification landscapes, and their dynamics during zebrafish embryo development

Tom Rappol[1], Maria Waldl[1,2,3,4], Anastasia Chugunova[5], Ivo L. Hofacker[2,6], Andrea Pauli[5] and Elisa Vilardo[1*]

[1] Centre for Anatomy & Cell Biology, Medical University of Vienna, 1090 Vienna, Austria

[2] Department of Theoretical Chemistry, University of Vienna, 1090 Vienna, Austria

[3] Vienna Doctoral School in Chemistry (DoSChem), University of Vienna, 1090 Vienna, Austria

[4] Institute of Computer Science and Interdisciplinary Center for Bioinformatics, Leipzig University, D-04107 Leipzig, Germany

[5] Research Institute of Molecular Pathology (IMP), Vienna BioCenter (VBC),1030 Vienna, Austria

[6] Faculty of Computer Science, Research Group Bioinformatics and Computational Biology, University of Vienna, 1090 Vienna, Austria

SUPPLEMENTARY TEXT

tRAM-seq analysis

Preprocessing: For each sample, we removed adapters with cutadapt (1) (-q 10 -e 0.15 --minimum-length=29 --discard-untrimmed -a <adapter sequence>) and extracted UMIs with umi-tools (2), trimmed leading Ts (possibly untemplated nucleotides introduced by TGIRT (3)) with cutadapt (-g 'XTTTTTTTTTTTTTTT' -e 0.0001, --minimum-length=20), filtered low-quality reads as well as short reads (<20nts) with fastp (4) (--length_required=20 --trim_poly_x poly_x_min_len=7 -p --low_complexity_filter --complexity_threshold=30) and performed standard quality control before and after each step (fastp and custom code). On average in this pre-processing step 29% of raw reads were removed with the majority (~24%) being removed in the first size selection steps. This resulted in 507173 to 11960487 filtered reads per sample, on average 3499983 (Supplementary Table 1).

Reference genome: As an initial reference set we collected 22 mitochondrial tRNA sequences from mitotRNAdb (5) (http://mttrna.bioinf.uni-leipzig.de/mtDataOutput/ August 2022) and 20577 genomic (nuclear) sequences (including 8673 high scoring genes) from a tRNAscan-SE (6) run as provided in the GtrnaDB (7,8) (gtrnadb.ucsc.edu/genomes/eukaryota/Dreri11/danRer11-tRNAs.tar.gz August 2022), extended the sequences by 3'-CCA and removed duplicates, resulting in a set of 10738 unique sequences.

In pre-studies we found that our NGS data includes reads from tRNA genes that are not covered in the high scoring GtrnaDB set. Therefore, we opted to include all predicted genes (including e.g. low confidence predictions and pseudo genes) and performed a mapping of all demethylated samples on this initial set of tRNA candidate genes with segemehl (-D 1 -M 200 -E 500 --accuracy 85) (9-11). Any gene that had a coverage of at least 500 reads per million (RPM) was included in the reference genome for further analysis. As opposed to later mapping steps, here multimappers were not assigned to one possible reference at random but were assigned to the reference that had the most reads mapping to it. This ensured that references that had no (or next to no) uniquely mapping reads assigned to them got filtered out.

After a trial run of the whole analysis pipeline we further manually refined the reference set. Since DM, BS, and Mock treated samples were sequenced in the same sequencing run, there was a potential for cross contamination due to index hopping, furthermore misincorporation due to a modification could assign reads to a gene that is not actually expressed. We therefore removed references with low coverage that only differ from a higher coverage reference at positions corresponding to a C to T transition, or that have a high misincorporation rate from known modification (e.g. references with G in known inosine positions). In addition, we re-added references that feature A instead of G nucleotides in known inosine positions.

For the mitochondrial tRNAs, we noticed inconsistencies between the sequence of in the database (5) and our own sequencing reads. We proceeded to isolate DNA from zebrafish samples and amplified the regions of mitochondrial DNA spanning mt-Arg, mt-Asp, mt-Ser(UCN) and mt-Thr. In mt-Arg, mt-Asp, and mt-Ser(UCN), we confirmed the SNPs observed in our tRAM-seq data, whilst for Thr we observed two alternative genotypes present in different samples, encoding either G or A at position 57 (Supplementary Figure S6). This diversity may be due to SNPs present in individual zebrafish within the pool of animals used per time point, or alternatively be due to heteroplasmy within individual animals in the pool. In any case, we updated our references to include the observed mitochondrial sequence variants. In conclusion, our reference included a total of 223 unique tRNA reference sequences.

Mapping: The mapping was performed with segemehl using short RNA optimized parameters (-E 500, -M 200, -D 3). The required mapping accuracy was adjusted to reflect the different number of modifications and thus different misincorporation frequencies in mock, demethylated and bisulfite treated samples (accuracy 80, 85 and 90 respectively). The mismatch tolerance also allows for mapping of SNPs and possibly left over non-templated nucleotides. For bisulfite treated samples segemehl was used with the additional option '-F 1' for BS mapping (and RNA specific post-processing). All samples achieve a mapping rate above 80% (DM: 80-97%; MOCK: 82-97%; BS: 81-94%). However, on average around 70% of the mapped reads are multimappers (DM: 55-70%; MOCK: 70-87%; BS: 63-79%). After mapping, any duplicated reads were removed based on their UMI. On average 5% of reads were filtered out in this step, with a range from two 2% to 18%.

Clustering: Given the high similarity, we decided to resolve the high number of multimappers by clustering similar tRNA genes. To retain as much resolution as possible, we decided to perform a two-step clustering based on sequence similarity and multimapper information. In the first step we align all reference sequences to the RFAM (12-14) tRNA covariance model (RF0005) with cmalign (15) and compute a pairwise edit distance of the aligned sequences. We recursively merge any references/clusters that have an edit distance below 4. For the second step, we first count the number of multimapping reads between two clusters. If more than 50% of reads that can be mapped to references in one cluster are multimappers with a second cluster, the two clusters are merged. This results in 68 well resolved clusters that retain full isoacceptor resolution except for one cluster containing lysine (anticodon TTT) and a TTA suppressor tRNA. In most cases the resolution is even better than isodecoder resolution (Supplementary Table 2). Less than 0.1% of reads are multimappers between clusters.

Alignment: The canonical positions within each reference sequence were assigned based on the alignment of the reference to the RFAM tRNA covariance model as described in the previous paragraph. In the first step, canonical positions according to (16) were manually assigned to corresponding positions in the RF0005 alignment. In a second step canonical positions of the reference sequences were automatically assigned based on the alignment of the reference to the RF0005 model. The following lines represent the consensus sequence and structure of the RF0005 tRNA alignment and the assigned canonical positions.

```
#=GC RF                     GgagauaU.A.GCucAgU...GGU...AgaGCg.u.cgGaC.UuaaAAuCcg.aag........................g...cgcg.GGU.UCg.Aa..UCCcg.c.uaucucC.a
#=GC SS_cons                (((((((,.,.<<<<___...___..._>>>>,.<.<<<<_.______>>>>.>,,........................,...,<<<.<<_.___.__.._>>>>.>.))))))).:
                            00000000 0 11111111 111222 222222 2 22333 3333333444 444-eee-eeeeeeeeeeeee-eee--44  4455 555 555 55  66666 6 6666777 7
                            12345678 9 01234567 789000 123456 7 89012 3456789012 345                        67  8901 234 567 89  01234 5 6789012 3
                                                A   AB
```

Abundance: The abundance of each cluster was computed based on the DM samples by mapping to the manually refined reference set, random assignment of multi mapping reads to one of the best matching references, counting of the mapped reads per reference, summing up the abundance of all references in a given cluster and normalizing the abundance count by the total count of mapped reads.

Misincorporation rates: Possible modification sites and changes of modification level are identified by computing position-wise misincorporation rates for all samples and all treatments. For each reference position, the total number of reads that cover the given position are counted. Furthermore, we count how many of those reads mismatch at the given position. The overall misincorporation rate per position in the clusters is computed by first adding up the mismatch counts and total coverage

count for equivalent positions in the tRNA references within the given cluster and then dividing the mismatch count by the total count. Equivalent positions between tRNA references within a cluster are defined by the covariance model based alignment described in the previous paragraph on the clustering approach.

RT stop fraction: In addition to the misincorporation rate, RT stops can indicate modified nucleotides. We assume that if a modification at position n leads to a RT stop, the last nucleotide in the generated cDNA is one position upstream, corresponding to one position downstream (n+1) towards the 3'-end of the tRNA reference sequence. Therefore, the number of reads that end at n+1 was divided by the total number of reads that map to n+1 in the given reference tRNA. Analogous to the misincorporation rate, the overall RT stop fraction of position n within a cluster was computed by summing all read-end counts at n+1 of equivalent positions and dividing by all reads that map to n+1 in the given cluster.

m^5C fractions: Based on the BS treated samples, we also detect putative m^5C modification sites and m^5C modification dynamics. Unmodified Cs are read as Ts after successful bisulfite treatment while m^5Cs are retained as Cs. Thus, m^5C modification calls can be based on a C-retention rate. For single tRNA references, the C -retention rate is only computed for C positions and the count of reads that contain a C in the mapped position is divided by the count of mapped reads that contain a C or a T in the given position.

Bisulfite treatment leads to an increase of multimappers, as any T in a read could originate either from a native T or a bisulfite converted C and could therefore be mapped to either reference sequence. To account for this, we selected to compute the C-retention rate in clusters not just based on reads that map to reference Cs but also count Ts that map to native T positions (since these could also originate from bisulfite converted Cs). Thus, per cluster and per position, we obtain the count of mapped Cs as the number of reads with a C that is mapped to a reference C; the count of mapped Ts is the number of reads with a T that is mapped to either a reference C or T. The C-retention rate is computed as usual as the count of Cs divided by the sum over the counted Cs and Ts. This C-retention rate may yield values lower than the actual m^5C methylation level if some of the references contain a native T at the position of interest. This C-retention rate is only representative for those references in a cluster that have exhibit a C or T in the given position, thus it does not provide any information on how representative these C and T containing

references are for the full cluster. Furthermore, apparent changes in the C-retention rate can also originate from changes in the abundance ratio between references with native Cs and native Ts. Such cases can be detected by comparing to the per reference abundance analysis in the DM samples.

Principal components analysis

To evaluate the reproducibility between replicates and similarities between time points, a principal components analysis was performed on the abundance data. As input data the normalized abundance (in RPM) of each tRNA cluster was used (Supplementary Figure S2). To give each cluster the same weight, the abundance of each cluster was standardized by removing the mean abundance of the cluster over all samples and scaling to unit variance.

Similarly, a principal components analysis was performed on the vst data of the mRNA-seq data (Supplementary Figure S3).

Comparison of tRAM-seq with previous tRNA sequencing strategies

The tRAM-seq protocol for library preparation was set up combining previously described protocols for small RNA sequencing (17-19). We first isolated the tRNA fraction by gel separation, and then processed it for end repair by deacylation and dephosphorylation. We reasoned that in this order we could reduce the contamination from fragments of other RNAs originating during incubations for end-repair, and at the same time reduce the substrates for PNK to the actual RNA fraction of interest (unlike mim-tRNAseq which treats first total RNA for end repair and then isolates the tRNA-sized range of RNA). Each sample was split and aliquots were subject to demethylation like in DM-tRNA-seq (19), or demethylation and bisulfite conversion for m^5C detection. The inclusion of the demethylated sample proved crucial for subsequent reference construction, abundance measurement, and interpretation of modification signatures (see results sections in main text and here below). For the subsequent library preparation steps, we followed a similar workflow as mim-tRNAseq but opted to use the adapter for 3’-ligation and the primer for reverse transcription from (17): these oligoes include randomized nucleoside residues at the 5’ end to minimize ligation bias and enable deduplication of reads originating from potential over-amplified PCR products (20).

Concerning the computational analysis, the published mim-tRNAseq pipeline bases its reference construction and alignment on the availability of modification data

from Modomics (21), which are not available for zebrafish. As consequence, the published mim-tRNAseq pipeline could not be used for our purpose. Furthermore, since mim-tRNAseq is an integrated, non-modular pipeline, using only part of its workflow or modifying it (to include read-deduplication based on UMIs, or to enable the analysis of bisulfite converted libraries for m^5C detection) was not feasible either.

For comparison with tRAM-seq, we nevertheless tested the computational analysis of mim-tRNAseq currently available on GitHub (version 1.3.7) without Modomics reference on our zebrafish NGS data. For this purpose, we first preprocessed the reads similarly as in tRAM-seq with minor changes: (i) we did no poly-T trimming since the GSNAP mapper used in the mim-tRNAseq pipline encompasses a soft-clipping step that removes the non-templated Ts; (ii) we used BBmap to deduplicate the reads instead of UMI-tools because it was easier to integrate with the mim-seq pipeline. Furthermore, we set out to find the optimal settings in mim-tRNAseq for analysis of *Danio rerio* tRNA in terms of mapping efficiency. In Supplementary Figure S 4A we show mapping statistics from a representative library (mock 24 hpf, replicate 3) with and without BBmap deduplication, and analysed with mim-tRNAseq using varying cluster identity thresholds with the following settings: mimseq --species Drer --cluster-id 0.90-0.97 --min-cov 0.002 --no-cca-analysis --max-mismatches 0.075 --remap --remap-mismatches 0.05. Additionally, we ran mim-tRNAseq using our custom generated *Danio rerio* tRNA reference using the following settings: mimseq -t tRAMseq.fa -m tRAMseq_mito.fa -o danRer11_eschColi-tRNAs.out --cluster-id 0.90 --min-cov 0.002 --no-cca-analysis --max-mismatches 0.075 --remap --remap-mismatches 0.05.

In mim-tRNAseq the clustering of the tRNAscan-SE predicted genes is exclusively based on sequence identity, whilst tRAM-seq clusters together tRNAs that have an edit distance to the RFAM covariance model below 4, and in a second step clusters with more than 50% multimappers are merged together. The lowest sequence identity setting (cluster ID) possible for clustering with mim-tRNAseq was 0.90 (any value below caused computation failure in our tests), which yielded 127 clusters versus 68 of tRAM-seq (both including 22 mitochondrial tRNAs). As shown for the representative library analysis in Supplementary Figure S4A, mim-tRNAseq performed worse than tRAM-seq in terms of mapped reads, and extent of multimappers between clusters. This difference was at least partially due to the larger number of clusters defined by mim-tRNAseq. It should be noted that mim-tRNAseq

forces the separation of different anticodon families in separate clusters, while this is not the case in tRAM-seq (resulting in tRNAs that differ only at the anticodon sequence to be still clustered together). Our rationale here was to maintain the most solid discrimination of tRNAs at the cluster level, whilst within the clusters it shall be kept into account that the extent of multimapping can affect the accurate quantification of individual references. Furthermore, mim-tRNAseq automatically discards from the reference all tRNAscan-SE predictions originating from highly repetitive genomic sequences. Considering that we detected some of those tRNAs in eggs and early embryo, such filtering needs to be reconsidered. Still, the use of our custom tRNA reference or the deduplication of reads did not improve the mapping statistics of the mim-tRNAseq analysis (Supplementary Figure S4A), suggesting that the different outcomes are due to downstream clustering and mapping by mim-tRNAseq.

Combining the results at the isoacceptor level for tRAM-seq and mim-tRNAseq analysis, we observed some differences in the overall proportions, in particular for Asp, Leu, and Lys (Supplementary Figure S4B). Comparing the inclusion/omission of the deduplication step we observed only minor differences in tRNA abundance profile, for instance some apparent correction of Asp overrepresentation; however, we anticipate that the inclusion of the deduplication step may be beneficial in the case of library preparation from low-input samples.

In the results of the mim-tRNAseq analysis, we noticed that it computed many tRNA clusters that barely have any modification signature (Supplementary Figure S4C). Inspecting those clusters closely, we noticed that they consisted of tRNA references that differ from other references at sites of high modification. For instance, mim-tRNAseq included in the reference and mapped reads to Met-CAT-97, Leu-CAG-125, and Asn-GTT-186, which do not have the highly conserved A58 (normally modified to m^1A58); similarly, mim-tRNAseq included and mapped reads to Pro-TGG-16 and Pro-AGG-33, which don't have G37 (normally modified to m^1G37), and Ile-AAT-62, which has no G26 (normally modified to $m^{2,2}$G26). We interpret this result as mim-tRNAseq being unable to distinguish tRNA modification signature due to the lack of Modomics annotation for zebrafish; the diverse ensemble of tRNA genes present in the genome of zebrafish causes the interpretation of the misincorporation signature as expression of alternative tRNA (pseudo)genes with non-typical sequence. These results highlight the power of tRAM-seq approach,

using a demethylated sample to construct the reference to clarify the majority of such ambiguous cases.

Lastly, in the results of mim-tRNAseq on our zebrafish library we also noticed that the mapping of mitochondrial tRNAs was faulty, causing the mismapping of reads, in particular for mt-His (Supplementary Figure 4D), suggesting that the mapping of mim-tRNAseq based on GSNAP may be not optimal for mapping less canonical tRNAs like the mitochondrial ones.

Supplementary Table 1 Reads pre-processing summary.

Number of reads that successfully passed a given preprocessing step in tRAM-seq.

	raw reads	adapter trimmed reads	size selection (>=29nts)	5’ polyT trimming + UMI extraction + size selection (>=20nts)	fastp qc passed reads
mean	4902502	4668533	3499983	3485431	3473257
min	1723083	1595365	507173	498841	496495
max	18460912	17098130	11960487	11820385	11737789
fraction remaining	1	0.952	0.714	0.711	0.708

Supplementary Table 2 Cluster composition.

Composition of the tRNA clusters in terms of tRNA gene reference identity and relative contribution per anticodon (based on anticodon coverage). Mitochondrial tRNAs with SNPs identified in this study were added to the reference, and are indicated with suffix “s”.

cluster name	cluster ID	anticodon ratio	tRNA name(s)
Ala-AGC	58	1	Ala-AGC-2; Ala-AGC-3; Ala-NNN-10; Ala-NNN-11; Ala-NNN-3
Ala-TGC/CGC	59	0.78_0.22	Ala-CGC-2; Ala-CGC-3; Ala-TGC-1; Ala-TGC-4
Arg-ACG	28	1	Arg-ACG-8; Arg-ACG-6; Arg-ACG-5; Arg-ACG-4; Arg-ACG-3; Arg-ACG-1
Arg-CCT	42	1	Arg-CCT-2; Arg-NNN-27
Arg-TCG	6	1	Arg-TCG-3
Arg-TCG	33	1	Arg-TCG-2; Arg-TCG-1
Arg-TCG/CCG	63	0.54_0.46	Arg-CCG-2; Arg-CCG-3; Arg-CCG-5; Arg-TCG-8; Arg-NNN-115; Arg-NNN-68
Arg-TCT	54	1	Arg-NNN-13; Arg-TCT-1; Arg-TCT-12; Arg-TCT-2; Arg-TCT-20; Arg-TCT-21; Arg-TCT-5; Arg-TCT-6
Asn-GTT	47	1	Asn-GTT-65; Asn-GTT-5; Asn-GTT-4; Asn-GTT-3; Asn-GTT-2; Asn-GTT-16; Asn-GTT-15; Asn-GTT-29
Asp-GTC	67	1	Asp-GTC-1; Asp-GTC-2; Asp-GTC-4; Asp-GTC-6
Cys-GCA	32	1	Cys-GCA-1; Cys-GCA-2
Gln-CTG/TTG	60	0.69_0.31	Gln-CTG-1; Gln-CTG-2; Gln-CTG-3; Gln-CTG-4; Gln-TTG-6; Gln-TTG-4; Gln-TTG-3; Gln-TTG-1
Glu-CTC	49	1	Glu-CTC-1; Glu-CTC-23
Glu-TTC/CTC	40	0.87_0.13	Glu-CTC-17; Glu-TTC-12; Glu-TTC-2; Glu-TTC-37; Glu-TTC-4; Glu-TTC-49
Gly-CCC	16	1	Gly-CCC-20
Gly-GCC/CCC	52	0.89_0.11	Gly-CCC-4; Gly-CCC-1; Gly-CCC-2; Gly-GCC-10; Gly-GCC-2; Gly-GCC-20; Gly-GCC-3; Gly-GCC-6; Gly-GCC-7
Gly-TCC	44	1	Gly-TCC-2; Gly-TCC-3
His-GTG	37	1	His-GTG-2; His-GTG-7
Ile-AAT	53	1	Ile-AAT-3; Ile-AAT-2; Ile-AAT-17; Ile-AAT-11; Ile-AAT-1; Ile-AAT-12
Ile-TAT	43	1	Ile-TAT-1; Ile-TAT-5

Leu-CAA	56	1	Leu-CAA-2; Leu-CAA-8; Leu-CAA-4; Leu-CAA-3; Leu-CAA-15; Leu-CAA-13; Leu-CAA-1; Leu-CAA-16
Leu-CAG	31	1	Leu-CAG-2; Leu-CAG-4; Leu-CAG-5
Leu-TAA	66	1	Leu-TAA-3; Leu-TAA-1; Leu-TAA-10; Leu-TAA-8
Leu-TAG/AAG	65	0.58_0.42	Leu-AAG-1; Leu-AAG-3; Leu-AAG-4; Leu-AAG-6; Leu-TAG-6; Leu-TAG-7; Leu-TAG-4; Leu-TAG-41; Leu-TAG-17; Leu-TAG-2; Leu-TAG-13; Leu-TAG-12
Lys-CTT	50	1	Lys-CTT-1; Lys-CTT-14; Lys-CTT-2; Lys-CTT-62; Lys-CTT-76
Lys-TTT_Sup-TTA	35	0.89_0.11	Lys-TTT-6; Lys-TTT-8; Sup-TTA-1
Met-CAT	61	1	Met-CAT-4; Met-CAT-2; Met-CAT-19; Phe-GAA-1; Phe-GAA-2
Phe-GAA	45	1	Phe-GAA-1; Phe-GAA-2
Pro-TGG/CGG/AGG	46	0.53_0.32_0.15	Pro-AGG-1; Pro-CGG-1; Pro-CGG-3; Pro-AGG-7; Pro-TGG-1; Pro-TGG-13; Pro-TGG-3
SeC-TCA	7	1	SeC-TCA-1
Ser-CGA	3	1	Ser-CGA-7
Ser-CGA	17	1	Ser-CGA-14
Ser-GCT	12	1	Ser-GCT-4
Ser-TGA	25	1	Ser-TGA-19
Ser-TGA/AGA/CGA	64	0.48_0.3_0.22	Ser-AGA-9; Ser-AGA-3; Ser-AGA-1; Ser-AGA-2; Ser-CGA-1; Ser-CGA-3; Ser-CGA-5; Ser-CGA-6; Ser-TGA-3; Ser-TGA-5; Ser-TGA-44; Ser-TGA-12; Ser-TGA-1
Thr-AGT/CGT/TGT	62	0.34_0.33_0.33	Thr-AGT-3; Thr-AGT-1; Thr-AGT-11; Thr-AGT-28; Thr-AGT-4; Thr-CGT-7; Thr-CGT-6; Thr-NNN-3; Thr-CGT-4; Thr-CGT-1; Thr-CGT-5; Thr-NNN-128; Thr-AGT-7; Thr-NNN-90; Thr-TGT-1; Thr-TGT-4
Thr-CGT	8	1	Thr-NNN-15
Thr-TGT	51	1	Thr-TGT-7; Thr-TGT-8
Trp-CCA	11	1	Trp-CCA-4
Trp-CCA	57	1	Trp-CCA-3; Trp-CCA-8; Trp-CCA-1; Trp-CCA-2
Tyr-GTA	39	1	Tyr-GTA-1; Tyr-GTA-2
Val-AAC	41	1	Val-AAC-30; Val-AAC-6
Val-CAC/TAC/AAC	55	0.77_0.14_0.09	Val-AAC-2; Val-AAC-1; Val-CAC-1; Val-CAC-11; Val-CAC-2; Val-CAC-4; Val-CAC-6; Val-CAC-8; Val-TAC-2; Val-TAC-1
Val-TAC	13	1	Val-TAC-22
Val-TAC	38	1	Val-TAC-4; Val-TAC-10
iMet-CAT	36	1	iMet-CAT-1; iMet-CAT-11; iMet-CAT-2
mt-Ala-TGC	27	1	mt-Ala-TGC
mt-Arg-TCG	30	1	mt-Arg-TCG; mt-Arg-TCGs
mt-Asn-GTT	30	1	mt-Asn-GTT
mt-Asp-GTC	23	1	mt-Asp-GTC; mt-Asp-GTCs
mt-Cys-GCA	29	1	mt-Cys-GCA
mt-Gln-TTG	29	1	mt-Gln-TTG
mt-Glu-TTC	1	1	mt-Glu-TTC
mt-Gly-TCC	24	1	mt-Gly-TCC
mt-His-GTG	0	1	mt-His-GTG
mt-Ile-GAT	19	1	mt-Ile-GAT
mt-Leu1-TAG	9	1	mt-Leu1-TAG
mt-Leu2-TAA	21	1	mt-Leu2-TAA
mt-Lys-TTT	2	1	mt-Lys-TTT
mt-Met-CAT	15	1	mt-Met-CAT
mt-Phe-GAA	14	1	mt-Phe-GAA
mt-Pro-TGG	10	1	mt-Pro-TGG
mt-Ser1-GCT	18	1	mt-Ser1-GCT
mt-Ser2-TGA	20	1	mt-Ser2-TGA; mt-Ser2-TGAs
mt-Thr-TGT	4	1	mt-Thr-TGT; mt-Thr-TGTs
mt-Trp-TCA	34	1	mt-Trp-TCA
mt-Tyr-GTA	34	1	mt-Tyr-GTA
mt-Val-TAC	48	1	mt-Val-TAC

Supplementary Table 3 Detected modifications per cluster.

Modifications inferred from RT-signature in mock samples and C retention in BS samples.

	m^1G9	m^1A9	m^1A14	acp^3U 20/20a/20	m^3C20	$m^{2,2}G$ 26/27	m^3C32	I34	m^5C34	m^1G37	m^1I37	o^2yW37	ms^2t^6A37	ms^2i^6A37	m^5C38	m^5C40	m^3Ce2	m^5C 48/49/50	m^1A58	m^5C72
Ala-AGC						+		+			+								+	
Ala-TGC/CGC				+		+					+							+	+	
Arg-ACG	+					+		+		+								+	+	
Arg-CCT	+						+												+	
Arg-TCG	+					+				+									+	
Arg-TCG	+					+				+									+	
Arg-TCG/CCG	+									+									+	
Arg-TCT	+						+												+	
Asn-GTT	+					+													+	
Asp-GTC		+													+			+/+		
Cys-GCA				+						+								+	+	+
Gln-CTG/TTG	+																	+/+	+	
Glu-CTC																		+/+	+	
Glu-TTC/CTC	+																	+/+	+	
Gly-CCC																		+/+	+	
Gly-GCC/CCC															+	+		+/+/+	+	
Gly-TCC	+																	+/+/+	+	
His-GTG										+								+	+	
Ile-AAT				+		+		+										+	+	
Ile-TAT	+					+													+	
Leu-CAA				+					+	+								+	+	
Leu-CAG				+		+				+							+	+	+	
Leu-TAA						+				+								+	+	
Leu-TAG/AAG				+		+		+		+								+	+	
Lys-CTT																		+	+	
Lys-TTT_Sup-TTA													+					+/+	+	
Met-CAT	+			+		+												+	+	
Phe-GAA			+			+						+						+/+	+	
Pro-TGG/CGG/AGG	+							+		+								+/+	+	
SeC-TCA																			+	
Ser-CGA						+	+											+	+	
Ser-CGA						+	+											+	+	
Ser-GCT						+	+										+	+	+	
Ser-TGA						+	+										+	+	+	
Ser-TGA/AGA/CGA						+	+	+									+	+	+	
Thr-AGT/CGT/TGT	+					+	+	+										+/+	+	+
Thr-CGT	+			+		+	+											+	+	+
Thr-TGT				+			+											+	+	+
Trp-CCA	+					+				+									+	
Trp-CCA	+					+				+									+	
Tyr-GTA				+		+/+				+									+	
Val-AAC						+		+										+/+	+	
Val-CAC/TAC/AAC								+										+/+	+	
Val-TAC																		+/+	+	
Val-TAC																		+/+	+	
iMet-CAT	+					+												+	+	
mt-Ala-TGC		+																		

mt-Arg-TCG		+								+										
mt-Asn-GTT						+													+	
mt-Asp-GTC		+																	+	
mt-Cys-GCA	+																		+	
mt-Gln-TTG	+																		+	
mt-Glu-TTC		+								+									+	
mt-Gly-TCC		+																		
mt-His-GTG		+																	+	
mt-Ile-GAT	+					+				+									+	
mt-Leu1-TAG		+								+								+	+	
mt-Leu2-TAA	+									+								+	+	
mt-Lys-TTT						+														
mt-Met-CAT									+										+	
mt-Phe-GAA		+								+										
mt-Pro-TGG		+								+									+	
mt-Ser1-GCT																			+	
mt-Ser2-TGA	+					+	+							+					+	
mt-Thr-TGT		+					+											+	+	
mt-Trp-TCA		+												+					+	
mt-Tyr-GTA	+					+				+								+	+	
mt-Val-TAC	+																		+	

SUPPLEMENTARY FIGURES

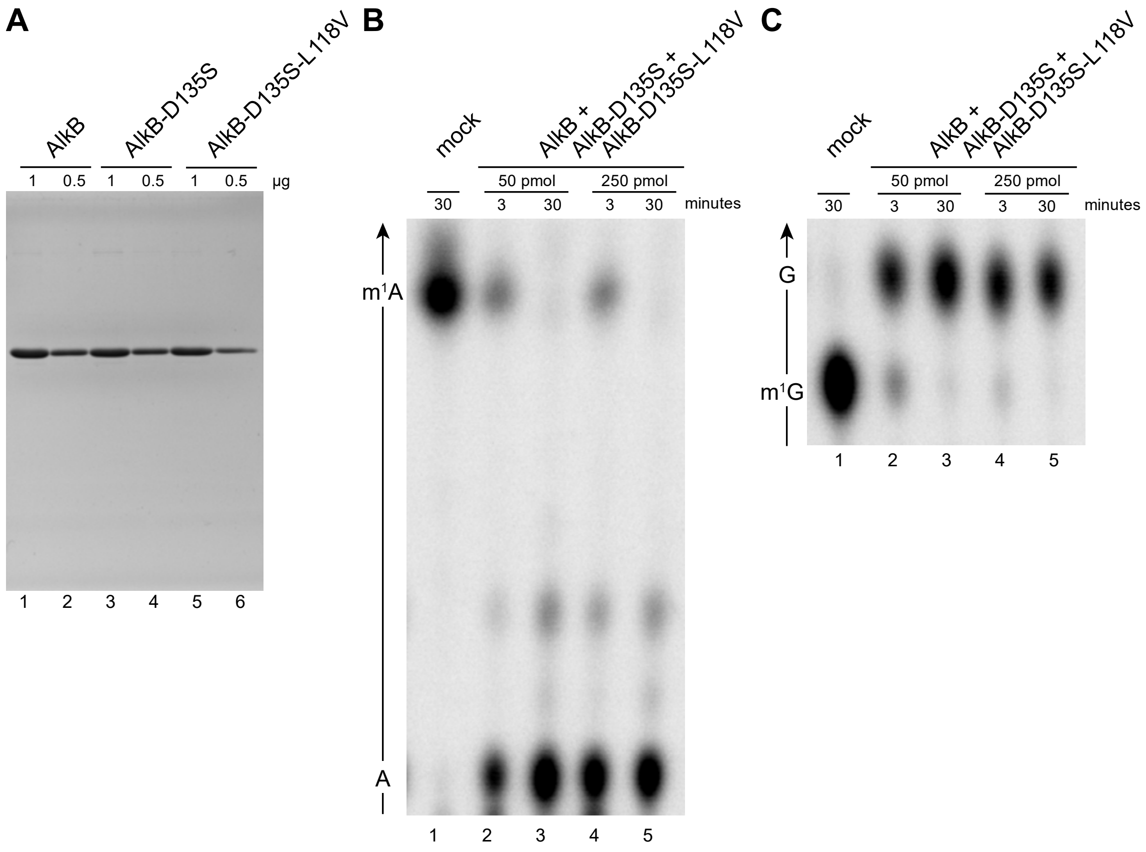

Supplementary Figure S1 AlkB purification and activity test. (**A**) Purity assessment of AlkB (and its mutants) by SDS-PAGE. On top the AlkB form and the amount loaded is annotated. (**B**) and (**C**) AlkB demethylation assay on RNA oligonucleotides carrying a 5' ^{32}P-labelled m^{1}A (B) or m^{1}G (C). The oligoes were incubated at varying concentrations of AlkB mix and reaction times. After P1 digestion, the nucleoside monophosphate residues were resolved by TLC. Arrows on the left indicate the direction of the solvent migration and highlight the nucleotide identity.

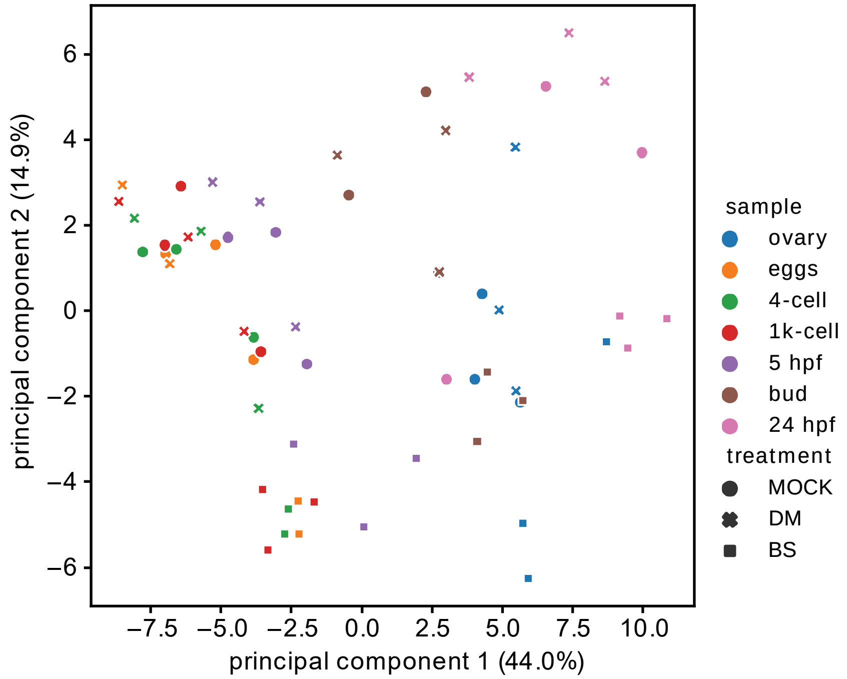

Supplementary Figure S2 PCA analysis of tRNA sequencing data. Plot of the first two dimensions of a principal component analysis, based on the normalized abundance (in RPM) of each tRNA cluster. Samples and treatments are identified by colors and symbols according to the legend.

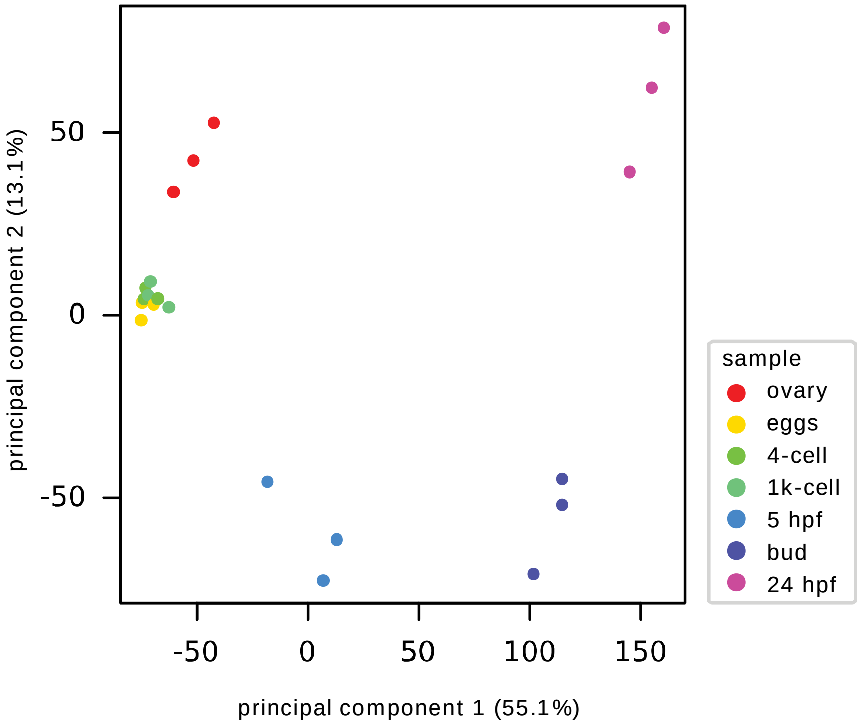

Supplementary Figure S3 PCA analysis of mRNA sequencing data. Plot of the first two dimensions of a principal component analysis, based on the vst-transformed count data. Samples are identified by colors according to the legend.

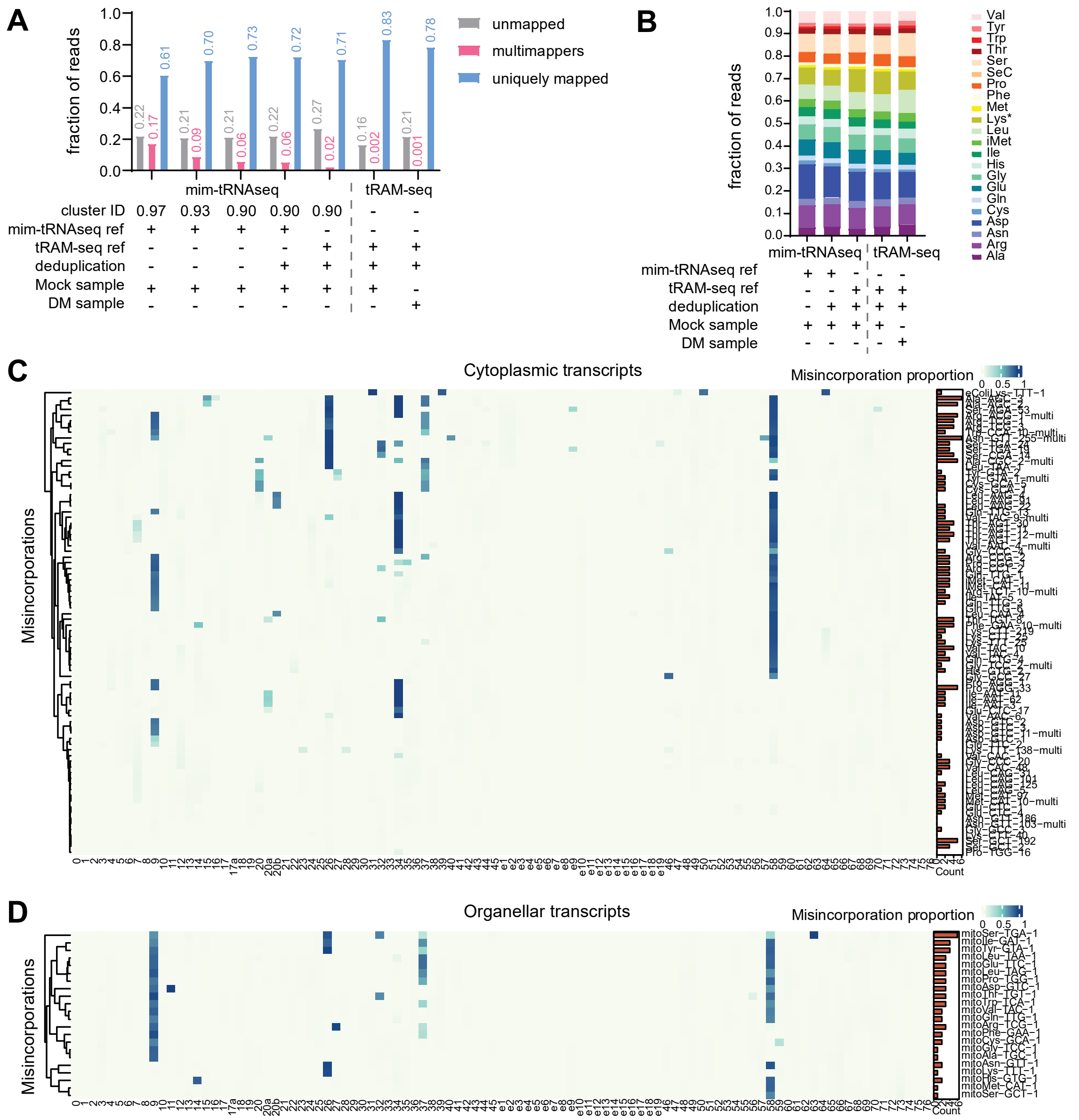

Supplementary Figure S4 Comparison of tRAM-seq with mim-tRNAseq. (**A**) Mapping statistics of representative 24 hpf libraries (mock or DM) analysed as indicated. (**B**) Distribution of mapped reads among isoacceptor families. Proportions were calculated based on the counts of reads mapped only to nucleo-cytoplasmic tRNAs. Reads mapped to Sup-tRNA are included in Lys* since they are nearly entirely multimappers between the two. (**C**) Misincorporation profile of cytosolic tRNAs computed by mim-tRNAseq (24 hpf mock library like in (A), cluster ID 0.90, no deduplication). (**D**) Misincorporation profile of mitochondrial tRNAs computed by mim-tRNAseq (same conditions as in (C)). Mim-tRNAseq erroneously shifted the mapping of reads to mt-His, resulting in the misincorporation signature of G9 being mapped to position 14. The reference for mt-Asp automatically retrieved by mim-tRNAseq from NCBI has C11, while the one from tRNAdb used in tRAM-seq has the correct T11. Additional SNPs were SerTGA C/T63, Arg C/T27 and Thr G/A57, as confirmed by Sanger sequencing (see Supplementary Figure S6).

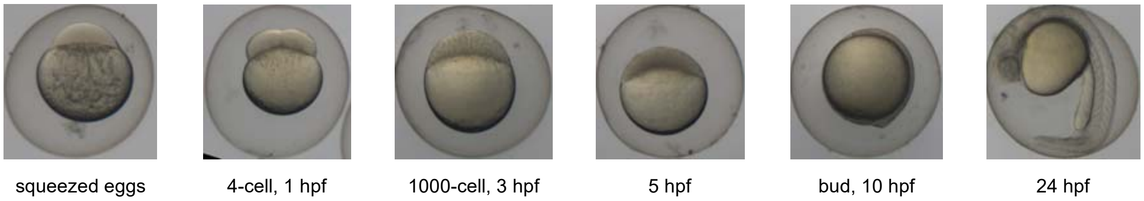

Supplementary Figure S5 Zebrafish embryo development stages. Representative microscopy images of activated eggs and embryo developmental stages used in the study. Labels indicate the stage description, and time is stated as hours post fertilization (hpf).

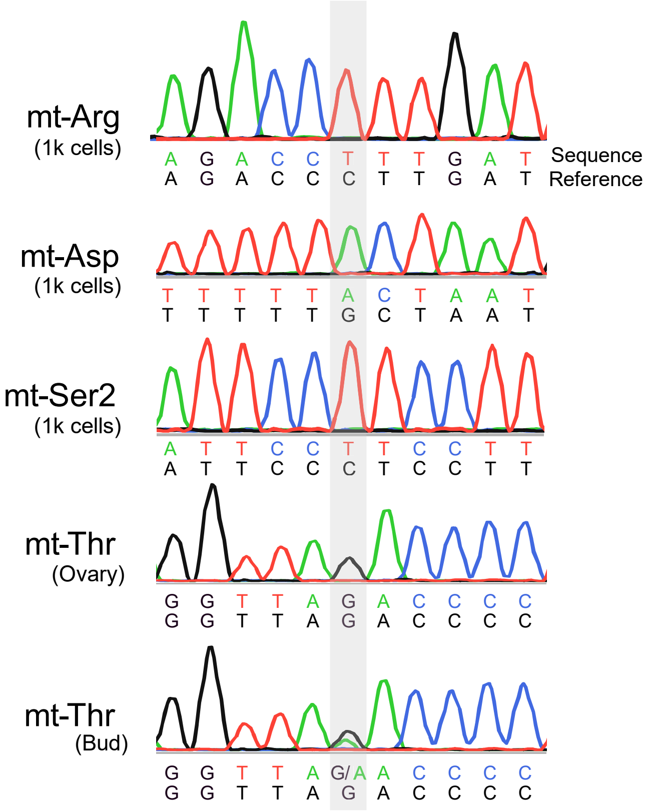

Supplementary Figure S6 Sanger sequencing analysis of mitochondrial DNA. Sanger sequencing results of amplified mitochondrial DNA from the samples indicated are shown as chromatogram traces. The obtained sequences were aligned to the reference zebrafish mitochondrial tRNA sequence from mitotRNAdb. SNPs identified are emphasized with a grey background.

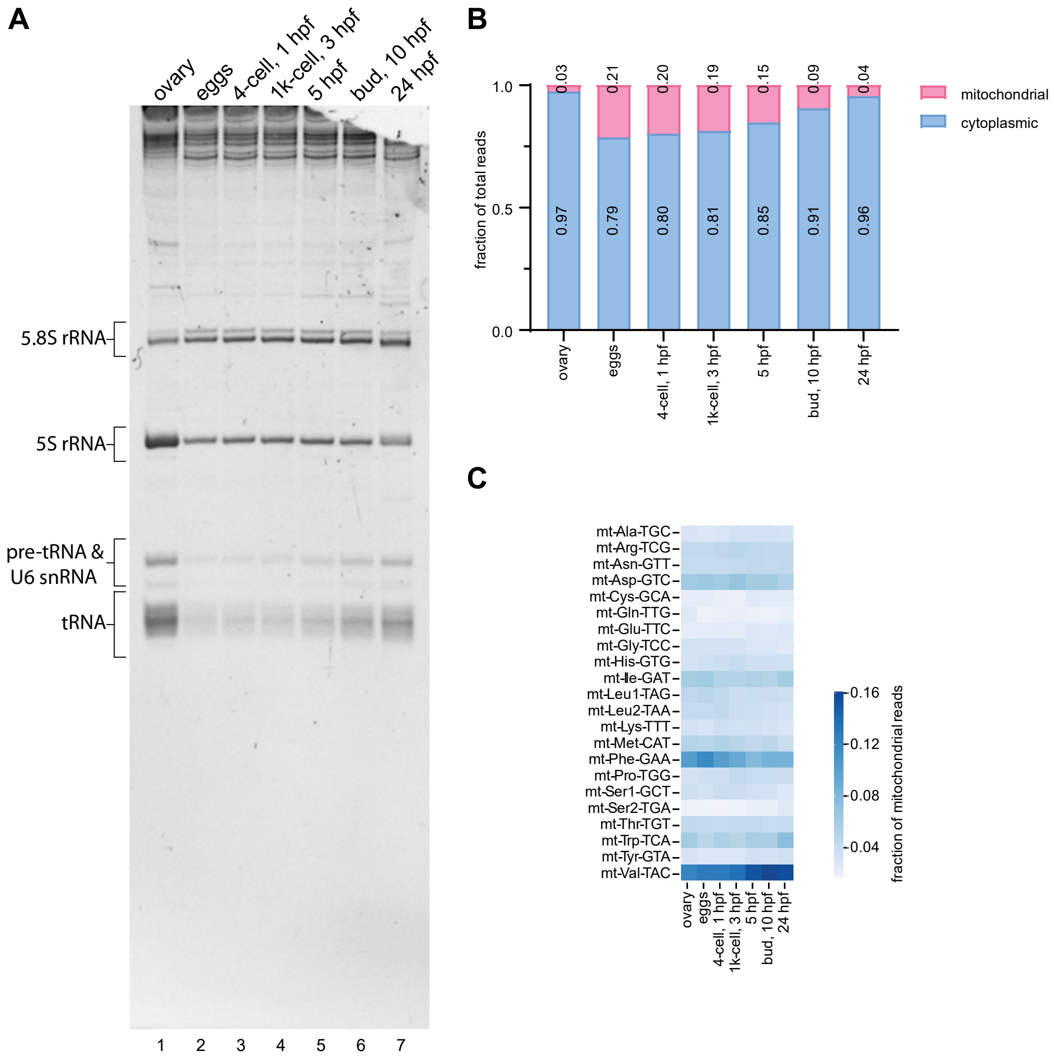

Supplementary Figure S7 Small RNA expression during zebrafish embryo development. (**A**) Total RNA extracted from zebrafish ovary, activated eggs, and embryos at different developmental stages was separated on a denaturing 15% polyacrylamide gel. The sample identity is indicated on the top; on the left the main small RNA bands are identified. (**B**) Relative abundance of mitochondrial versus nucleo-cytoplasmic tRNAs, expressed as fraction of total reads. Data are means of three biological replicates with the exception of the activated eggs (n=2). (**C**) Heat-map representing the normalized abundance of mitochondrial tRNAs (y-axis) in the analysed samples (x-axis). The number of reads mapped to each tRNA was normalized by the total mitochondrial tRNAs read count and plotted. The blue colour scale indicates the fraction of reads mapped to the individual tRNA. Data are means of three biological replicates with the exception of the activated eggs (n=2).

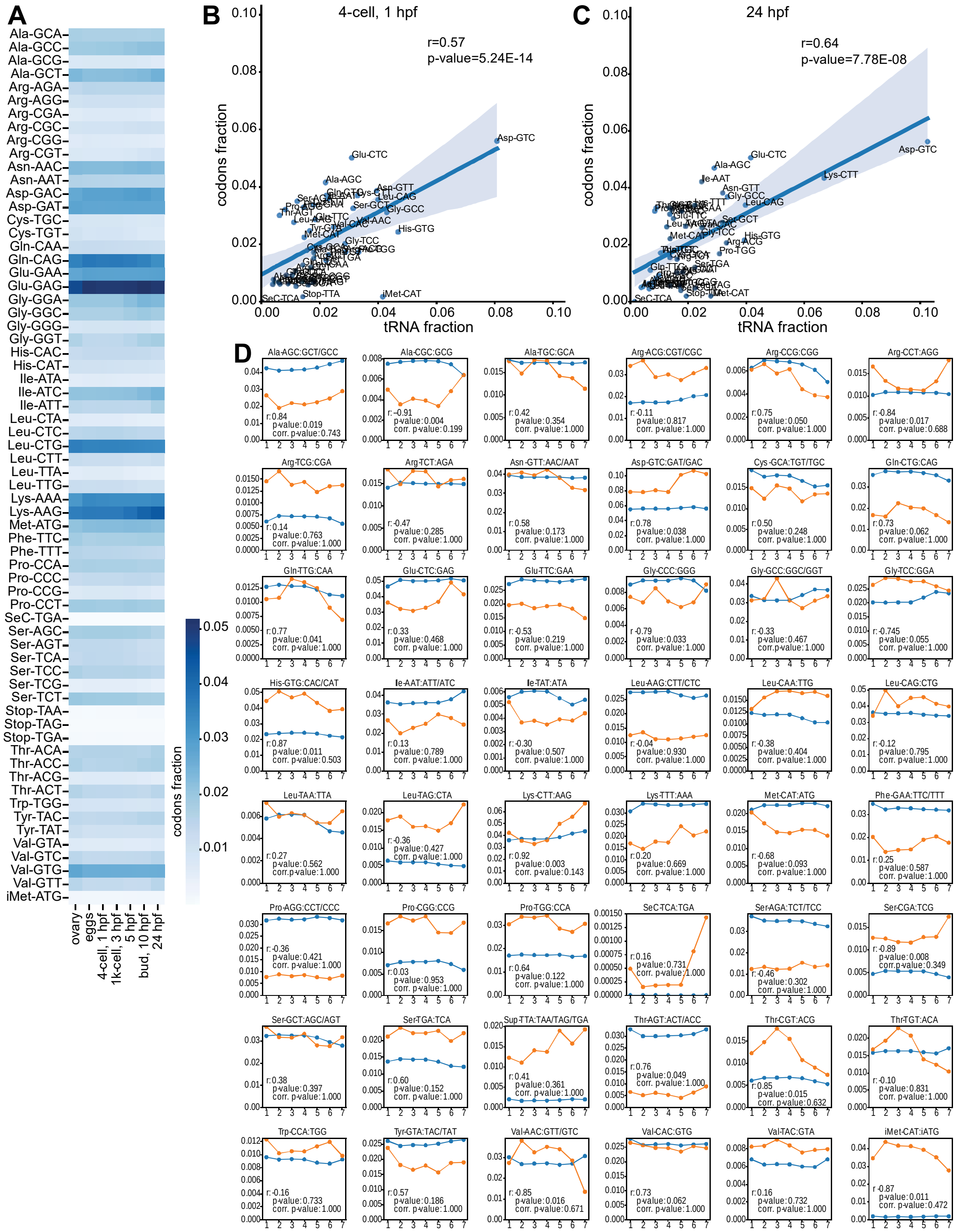

Supplementary Figure S8 Codon frequency dynamics during zebrafish embryo development. (**A**) Heat-map showing the codon frequency in the transcriptome across zebrafish ovary, activated eggs, and embryo developmental stages (x-axis). Codons and amino acids are indicated on the y-axis. The blue-colour scale indicates the codon fraction calculated as follows: for each protein coding gene, the occurrences of each codon was counted and multiplied by the gene's expression level (normalized read count). To obtain the codon frequency, the per-codon counts were summed up over all genes and then divided by the total number of codons. Data are shown as means of biological triplicates. (**B-C**)

Correlation between tRNA abundance and weighted codon frequency. The analysis is shown for the time points 4k-1 hpf (B) and 24 hpf (C). The best-fit line and 95% confidence intervals are shown as blue line and light blue area, respectively. Pearson correlation coefficient r and p-value are reported as inset in the plots. (**D**) Correlation analysis between tRNA abundance and matched codon frequencies weighted by gene expression. Fractions of total are plotted on the y-axis: the orange line indicates the tRNA abundance, data points are means of biological triplicates with the exception of the activated eggs (n=2); the blue line indicates the weighted codon frequency, data points are means of biological triplicates. Samples are abbreviated as follows: 1=ovary; 2=eggs; 3=4-cell, 1 hpf; 4=1k-cell, 3 hpf; 5=5 hpf; 6=bud, 10 hpf; 7=24 hpf. Pearson correlation coefficient r, p-value, and p-value corrected for multiple testing with Holm-Bonferroni method are reported as inset in the plots.

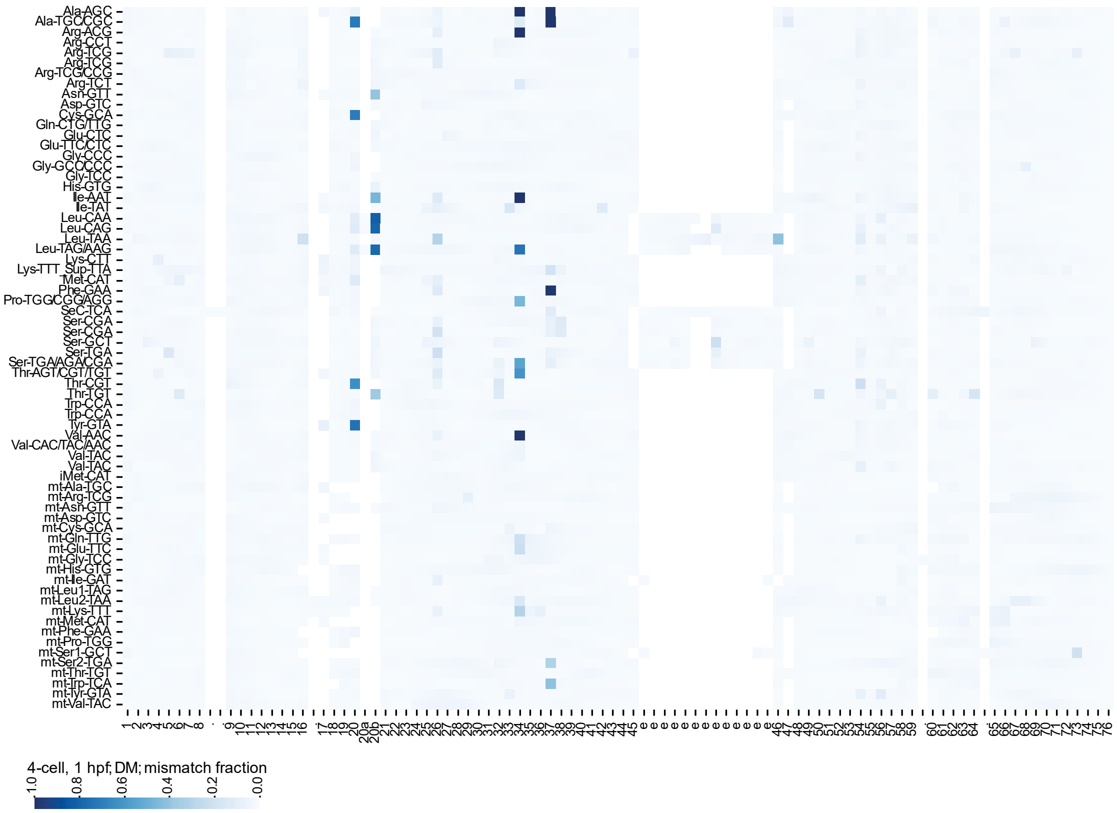

Supplementary Figure S9 Misincorporation signatures of demethylated tRNA from 4-cell stage zebrafish embryos. Heat-map of misincorporation fraction of all cytosolic tRNA clusters and mt-tRNAs (y-axis). Canonical nucleotide positions are annotated on the x-axis. Nucleotide positions that are rarely present in the tRNA clusters are annotated with a dot. The blue colour scale indicates the mean mismatch fraction across three biological replicates.

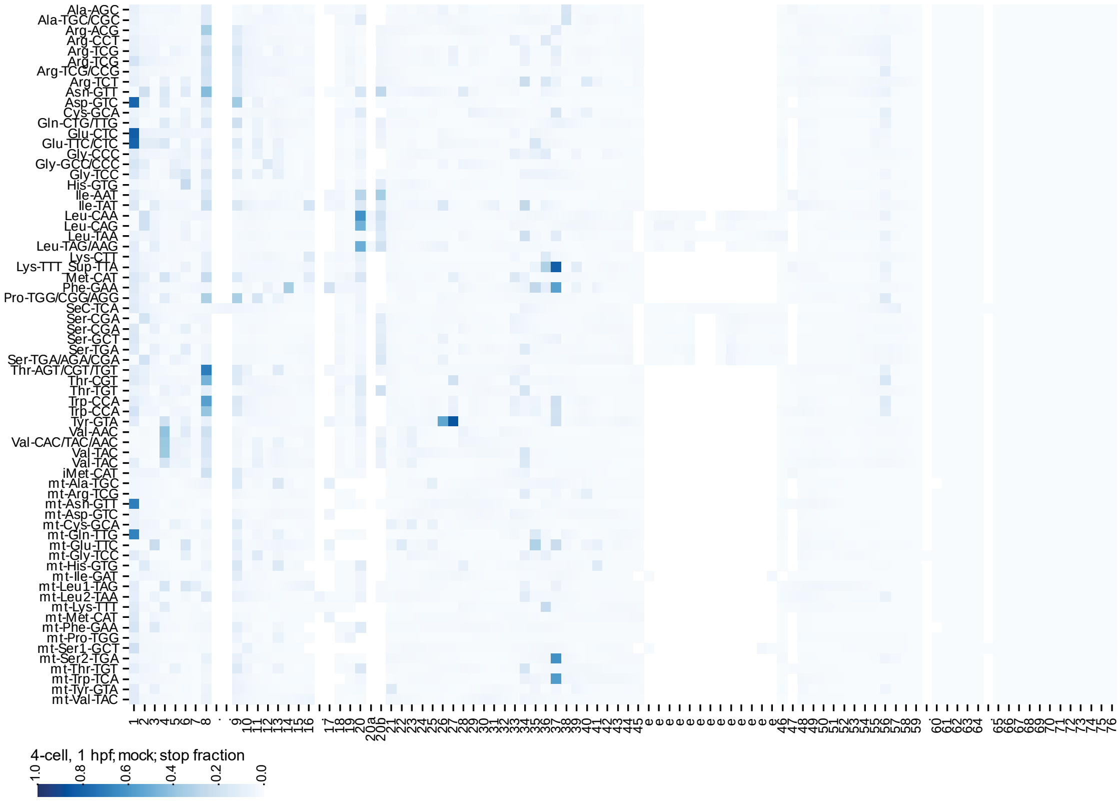

Supplementary Figure S10 RT stop signatures of tRNA from 4-cell stage zebrafish embryos. Heat-map of RT stop fraction of all cytosolic tRNA clusters and mt-tRNAs (y-axis). Canonical nucleotide positions are annotated on the x-axis. Nucleotide positions that are rarely present in the tRNA clusters are annotated with a dot. The blue colour scale indicates the mean RT stop fraction across three biological replicates. The stop signal at the 5' end of the clusters Asp-GTC, Glu-CTC, Glu-TTC/CTC, mt-Asn-GTT and mt-Gln-TTG are most likely caused by over-trimming of leading Ts (see experimental details in Supplementary File 1).

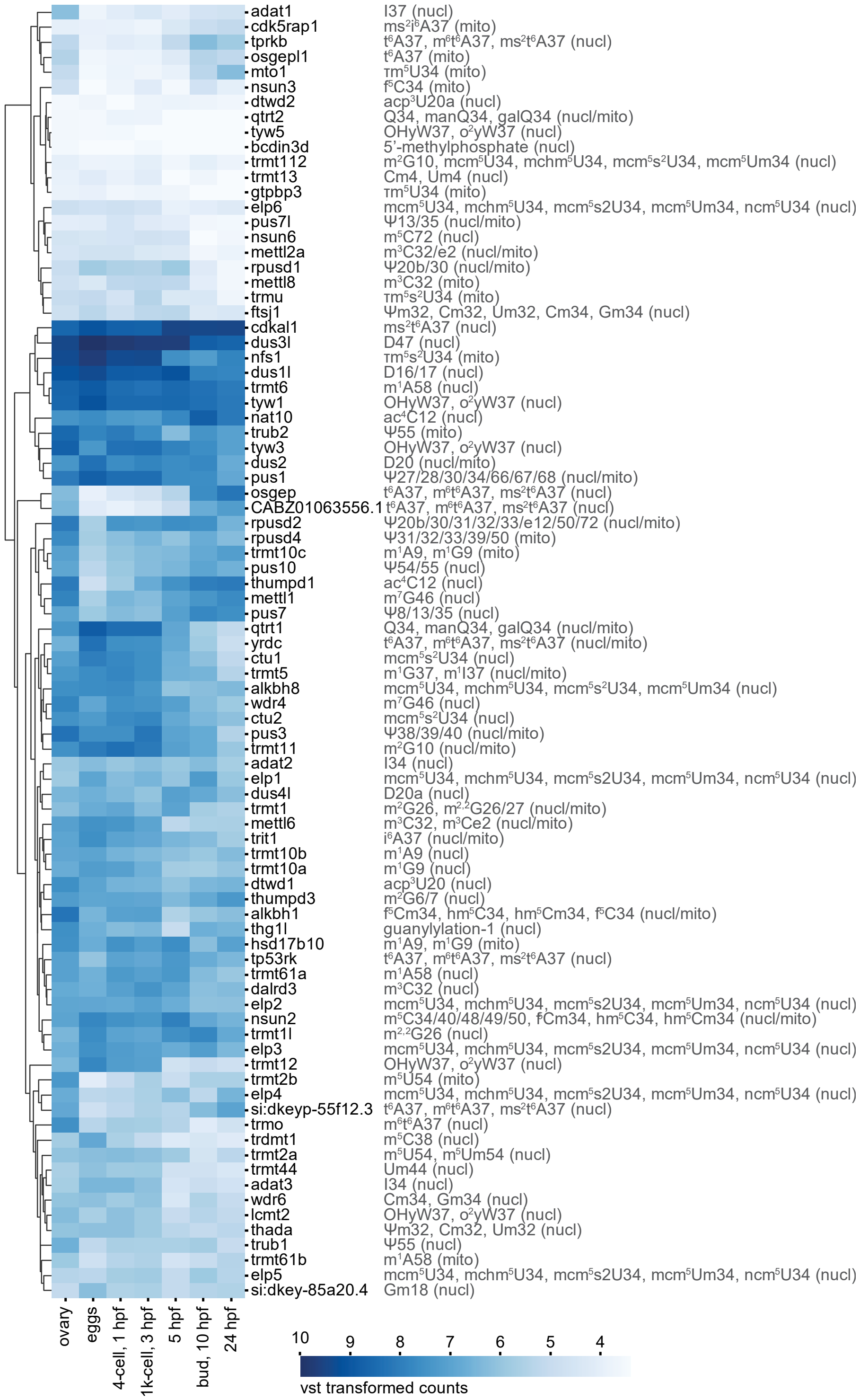

Supplementary Figure S11 Gene expression analysis of tRNA modification enzymes. Heat-map showing mean VST transformed counts (blue colour scale) for tRNA modification enzymes (y-axis) across zebrafish ovary and embryo development (x-axis). To the right, the modification in which the enzyme is involved is indicated in grey. The tRNA modification enzymes were hierarchically clustered based on their similarity in expression levels or based on their similarity in expression dynamics. The blue colour scale indicates the mean vst transformed counts across three biological replicates.

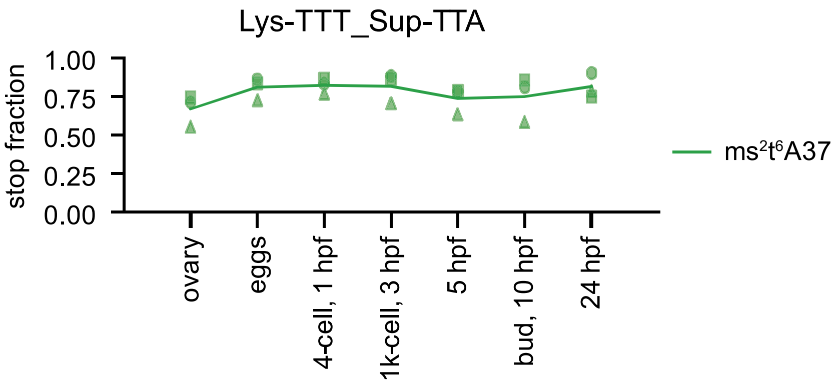

Supplementary Figure S12 RT stop caused by ms^{2}t^{6}A37 in the cluster Lys-TTT_Sup-TTA. RT stop fraction (y-axis) at position A37 in the cluster Lys-TTT_Sup-TTA across zebrafish embryo development (x-axis). The line represents the mean of three biological replicates, which are indicated by square, circle and triangle.

SUPPLEMENTARY REFERENCES

1. Martin, M. (2011) Cutadapt removes adapter sequences from high-throughput sequencing reads. *EMBnet.journal*, **17**, 10-12.
2. Smith, T., Heger, A. and Sudbery, I. (2017) UMI-tools: modeling sequencing errors in Unique Molecular Identifiers to improve quantification accuracy. *Genome Res*, **27**, 491-499.
3. Qin, Y., Yao, J., Wu, D.C., Nottingham, R.M., Mohr, S., Hunicke-Smith, S. and Lambowitz, A.M. (2016) High-throughput sequencing of human plasma RNA by using thermostable group II intron reverse transcriptases. *RNA*, **22**, 111-128.
4. Chen, S., Zhou, Y., Chen, Y. and Gu, J. (2018) fastp: an ultra-fast all-in-one FASTQ preprocessor. *Bioinformatics*, **34**, i884-i890.
5. Juhling, F., Morl, M., Hartmann, R.K., Sprinzl, M., Stadler, P.F. and Putz, J. (2009) tRNAdb 2009: compilation of tRNA sequences and tRNA genes. *Nucleic Acids Res*, **37**, D159-162.
6. Chan, P.P. and Lowe, T.M. (2019) tRNAscan-SE: Searching for tRNA Genes in Genomic Sequences. *Methods Mol Biol*, **1962**, 1-14.
7. Chan, P.P. and Lowe, T.M. (2016) GtRNAdb 2.0: an expanded database of transfer RNA genes identified in complete and draft genomes. *Nucleic Acids Res*, **44**, D184-189.
8. Chan, P.P. and Lowe, T.M. (2009) GtRNAdb: a database of transfer RNA genes detected in genomic sequence. *Nucleic Acids Res*, **37**, D93-97.
9. Otto, C., Stadler, P.F. and Hoffmann, S. (2012) Fast and sensitive mapping of bisulfite-treated sequencing data. *Bioinformatics*, **28**, 1698-1704.
10. Hoffmann, S., Otto, C., Kurtz, S., Sharma, C.M., Khaitovich, P., Vogel, J., Stadler, P.F. and Hackermuller, J. (2009) Fast mapping of short sequences with mismatches, insertions and deletions using index structures. *PLoS Comput Biol*, **5**, e1000502.
11. Otto, C., Stadler, P.F. and Hoffmann, S. (2014) Lacking alignments? The next-generation sequencing mapper segemehl revisited. *Bioinformatics*, **30**, 1837-1843.
12. Griffiths-Jones, S., Bateman, A., Marshall, M., Khanna, A. and Eddy, S.R. (2003) Rfam: an RNA family database. *Nucleic Acids Res*, **31**, 439-441.
13. Kalvari, I., Nawrocki, E.P., Argasinska, J., Quinones-Olvera, N., Finn, R.D., Bateman, A. and Petrov, A.I. (2018) Non-Coding RNA Analysis Using the Rfam Database. *Curr Protoc Bioinformatics*, **62**, e51.
14. Kalvari, I., Nawrocki, E.P., Ontiveros-Palacios, N., Argasinska, J., Lamkiewicz, K., Marz, M., Griffiths-Jones, S., Toffano-Nioche, C., Gautheret, D., Weinberg, Z. *et al.* (2021) Rfam 14: expanded coverage of metagenomic, viral and microRNA families. *Nucleic Acids Res*, **49**, D192-D200.
15. Nawrocki, E.P. and Eddy, S.R. (2013) Infernal 1.1: 100-fold faster RNA homology searches. *Bioinformatics*, **29**, 2933-2935.
16. Sprinzl, M., Horn, C., Brown, M., Ioudovitch, A. and Steinberg, S. (1998) Compilation of tRNA sequences and sequences of tRNA genes. *Nucleic Acids Res*, **26**, 148-153.
17. Kim, Y., Eggers, C., Shvetsova, E., Kleemann, L., Sin, O. and Leidel, S.A. (2021) Analysis of codon-specific translation by ribosome profiling. *Methods Enzymol*, **658**, 191-223.
18. Behrens, A., Rodschinka, G. and Nedialkova, D.D. (2021) High-resolution quantitative profiling of tRNA abundance and modification status in eukaryotes by mim-tRNAseq. *Mol Cell*, **81**, 1802-1815 e1807.

19. Zheng, G., Qin, Y., Clark, W.C., Dai, Q., Yi, C., He, C., Lambowitz, A.M. and Pan, T. (2015) Efficient and quantitative high-throughput tRNA sequencing. *Nat Methods*, **12**, 835-837.
20. Jayaprakash, A.D., Jabado, O., Brown, B.D. and Sachidanandam, R. (2011) Identification and remediation of biases in the activity of RNA ligases in small-RNA deep sequencing. *Nucleic Acids Res*, **39**, e141.
21. Boccaletto, P., Stefaniak, F., Ray, A., Cappannini, A., Mukherjee, S., Purta, E., Kurkowska, M., Shirvanizadeh, N., Destefanis, E., Groza, P. *et al.* (2022) MODOMICS: a database of RNA modification pathways. 2021 update. *Nucleic Acids Res*, **50**, D231-D235.
