# Supplementary material for: tRNA expression and modification landscapes, and their dynamics during zebrafish embryo development
Source: Nucleic Acids Res. 2024 Jul 11;52(17):10575–94. doi: 10.1093/nar/gkae595 (PMC11417395; doi:10.1093/nar/gkae595)
Supplement: gkae595_Supplemental_Files [file gkae595_supplemental_files.zip › Supplementary_file_5-abundance single refs_all-DM-v3.pdf]

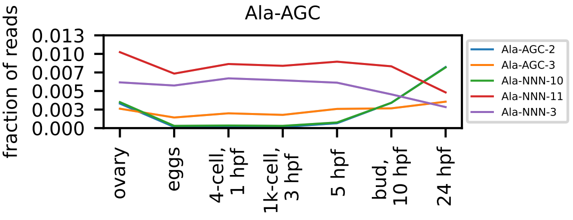

Ala-AGC
fraction of reads
0.013
0.010
0.007
0.005
0.003
0.000
ovary
eggs
4-cell, 1 hpf
1k-cell, 3 hpf
5 hpf
bud, 10 hpf
24 hpf
Ala-AGC-2
Ala-AGC-3
Ala-NNN-10
Ala-NNN-11
Ala-NNN-3

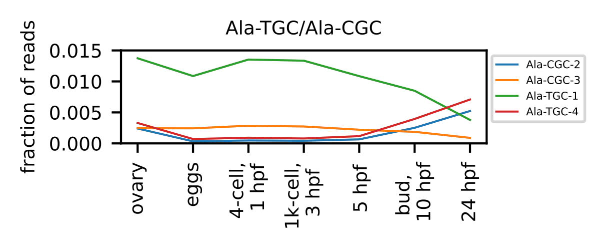

Ala-TGC/Ala-CGC
fraction of reads
0.015
0.010
0.005
0.000
ovary
eggs
4-cell, 1 hpf
1k-cell, 3 hpf
5 hpf
bud, 10 hpf
24 hpf
Ala-CGC-2
Ala-CGC-3
Ala-TGC-1
Ala-TGC-4

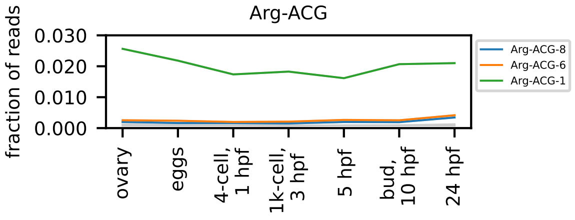

Arg-ACG
fraction of reads
0.030
0.020
0.010
0.000
Arg-ACG-8
Arg-ACG-6
Arg-ACG-1
ovary
eggs
4-cell, 1 hpf
1k-cell, 3 hpf
5 hpf
bud, 10 hpf
24 hpf

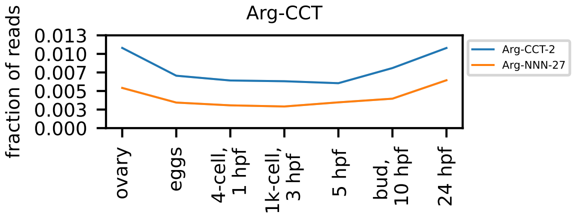

Arg-CCT
fraction of reads
0.013
0.010
0.007
0.005
0.003
0.000
Arg-CCT-2
Arg-NNN-27
ovary
eggs
4-cell, 1 hpf
1k-cell, 3 hpf
5 hpf
bud, 10 hpf
24 hpf

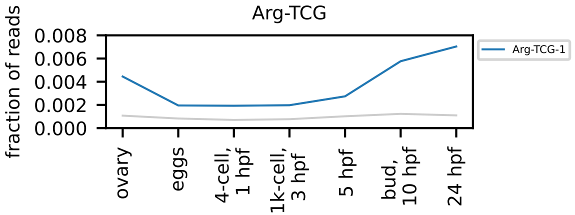

Arg-TCG
fraction of reads
0.008
0.006
0.004
0.002
0.000
Arg-TCG-1
ovary
eggs
4-cell, 1 hpf
1k-cell, 3 hpf
5 hpf
bud, 10 hpf
24 hpf

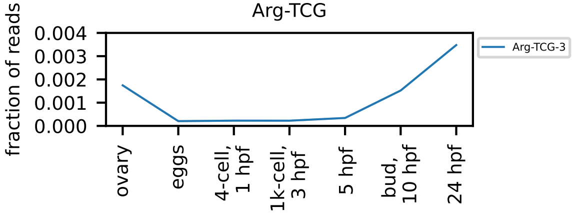
Arg-TCG
fraction of reads
0.004
0.003
0.002
0.001
0.000
Arg-TCG-3
ovary
eggs
4-cell, 1 hpf
1k-cell, 3 hpf
5 hpf
bud, 10 hpf
24 hpf

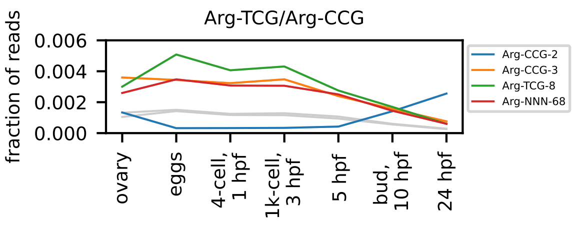
Arg-TCG/Arg-CCG
fraction of reads
0.006
0.004
0.002
0.000
ovary
eggs
4-cell, 1 hpf
1k-cell, 3 hpf
5 hpf
bud, 10 hpf
24 hpf
Arg-CCG-2
Arg-CCG-3
Arg-TCG-8
Arg-NNN-68

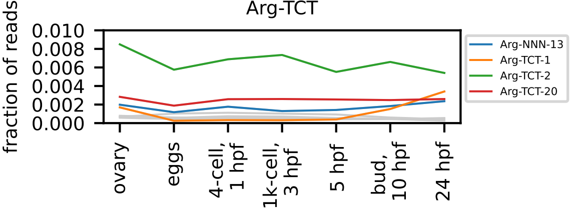

Arg-TCT
fraction of reads
0.010
0.008
0.006
0.004
0.002
0.000
Arg-NNN-13
Arg-TCT-1
Arg-TCT-2
Arg-TCT-20
ovary
eggs
4-cell, 1 hpf
1k-cell, 3 hpf
5 hpf
bud, 10 hpf
24 hpf

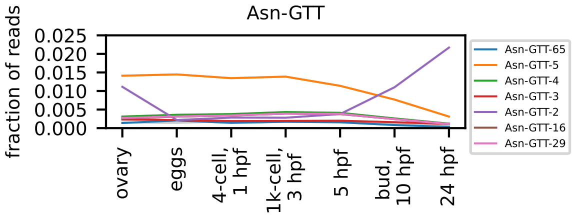
Asn-GTT
fraction of reads
0.025
0.020
0.015
0.010
0.005
0.000
ovary
eggs
4-cell, 1 hpf
1k-cell, 3 hpf
5 hpf
bud, 10 hpf
24 hpf
Asn-GTT-65
Asn-GTT-5
Asn-GTT-4
Asn-GTT-3
Asn-GTT-2
Asn-GTT-16
Asn-GTT-29

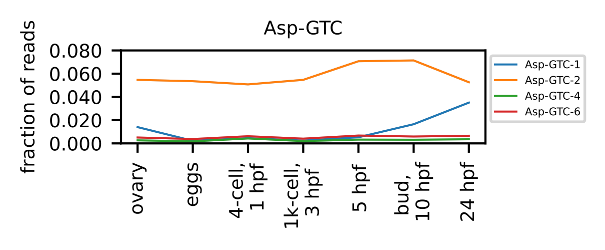

Asp-GTC
fraction of reads
0.080
0.060
0.040
0.020
0.000
ovary
eggs
4-cell, 1 hpf
1k-cell, 3 hpf
5 hpf
bud, 10 hpf
24 hpf
Asp-GTC-1
Asp-GTC-2
Asp-GTC-4
Asp-GTC-6

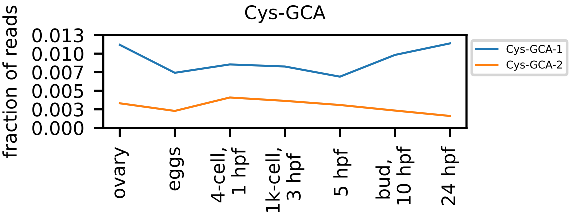
Cys-GCA
fraction of reads
0.013
0.010
0.007
0.005
0.003
0.000
ovary
eggs
4-cell,
1 hpf
1k-cell,
3 hpf
5 hpf
bud,
10 hpf
24 hpf
Cys-GCA-1
Cys-GCA-2

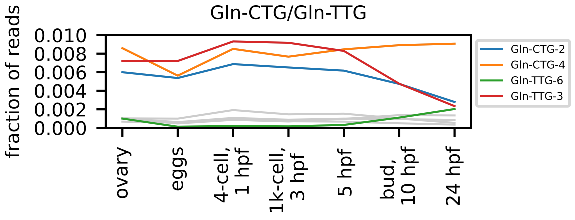

Gln-CTG/Gln-TTG
fraction of reads
0.010
0.008
0.006
0.004
0.002
0.000
ovary
eggs
4-cell, 1 hpf
1k-cell, 3 hpf
5 hpf
bud, 10 hpf
24 hpf
Gln-CTG-2
Gln-CTG-4
Gln-TTG-6
Gln-TTG-3

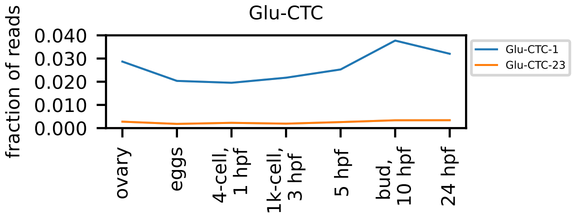

Glu-CTC
fraction of reads
0.040
0.030
0.020
0.010
0.000
ovary
eggs
4-cell, 1 hpf
1k-cell, 3 hpf
5 hpf
bud, 10 hpf
24 hpf
Glu-CTC-1
Glu-CTC-23

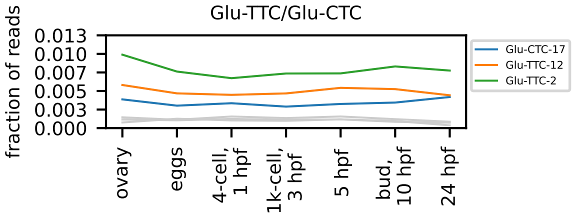

Glu-TTC/Glu-CTC
fraction of reads
0.013
0.010
0.007
0.005
0.003
0.000
ovary
eggs
4-cell, 1 hpf
1k-cell, 3 hpf
5 hpf
bud, 10 hpf
24 hpf
Glu-CTC-17
Glu-TTC-12
Glu-TTC-2

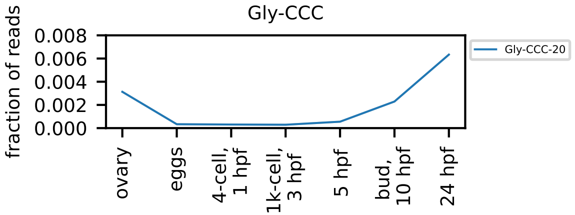
Gly-CCC
fraction of reads
0.008
0.006
0.004
0.002
0.000
Gly-CCC-20
ovary
eggs
4-cell, 1 hpf
1k-cell, 3 hpf
5 hpf
bud, 10 hpf
24 hpf

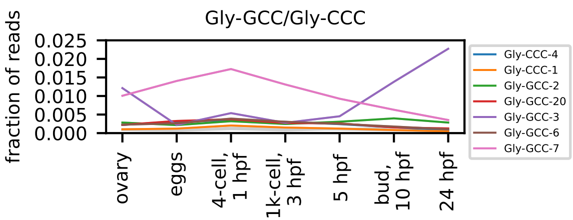
Gly-GCC/Gly-CCC
fraction of reads
0.025
0.020
0.015
0.010
0.005
0.000
ovary
eggs
4-cell, 1 hpf
1k-cell, 3 hpf
5 hpf
bud, 10 hpf
24 hpf
Gly-CCC-4
Gly-CCC-1
Gly-GCC-2
Gly-GCC-20
Gly-GCC-3
Gly-GCC-6
Gly-GCC-7

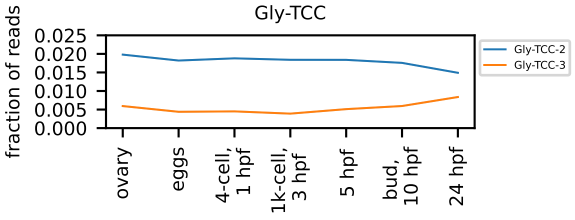

Gly-TCC
fraction of reads
0.025
0.020
0.015
0.010
0.005
0.000
Gly-TCC-2
Gly-TCC-3
ovary
eggs
4-cell, 1 hpf
1k-cell, 3 hpf
5 hpf
bud, 10 hpf
24 hpf

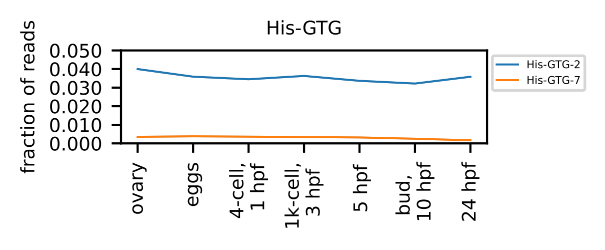
His-GTG
fraction of reads
0.050
0.040
0.030
0.020
0.010
0.000
His-GTG-2
His-GTG-7
ovary
eggs
4-cell, 1 hpf
1k-cell, 3 hpf
5 hpf
bud, 10 hpf
24 hpf

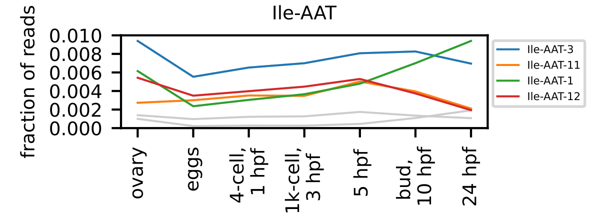
Ile-AAT
fraction of reads
0.010
0.008
0.006
0.004
0.002
0.000
ovary
eggs
4-cell, 1 hpf
1k-cell, 3 hpf
5 hpf
bud, 10 hpf
24 hpf
Ile-AAT-3
Ile-AAT-11
Ile-AAT-1
Ile-AAT-12

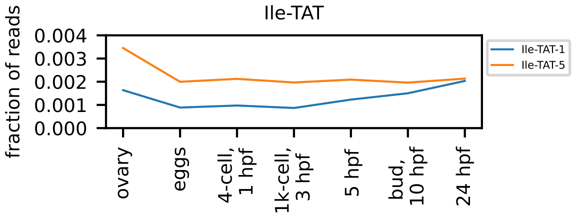

Ile-TAT
fraction of reads
0.004
0.003
0.002
0.001
0.000
ovary
eggs
4-cell, 1 hpf
1k-cell, 3 hpf
5 hpf
bud, 10 hpf
24 hpf
Ile-TAT-1
Ile-TAT-5

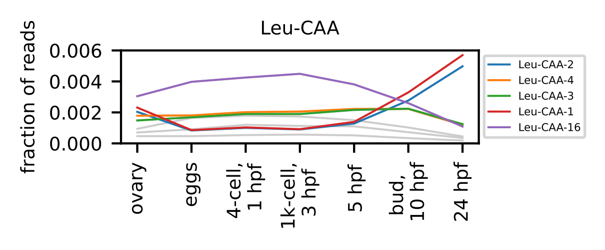

Leu-CAA
fraction of reads
0.006
0.004
0.002
0.000
Leu-CAA-2
Leu-CAA-4
Leu-CAA-3
Leu-CAA-1
Leu-CAA-16
ovary
eggs
4-cell, 1 hpf
1k-cell, 3 hpf
5 hpf
bud, 10 hpf
24 hpf

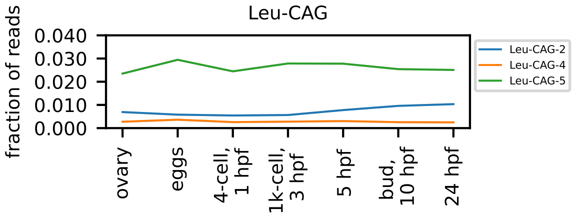

Leu-CAG
fraction of reads
0.040
0.030
0.020
0.010
0.000
ovary
eggs
4-cell, 1 hpf
1k-cell, 3 hpf
5 hpf
bud, 10 hpf
24 hpf
Leu-CAG-2
Leu-CAG-4
Leu-CAG-5

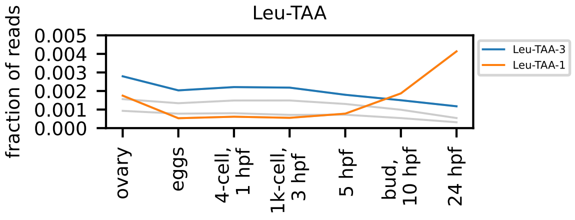

Leu-TAA
fraction of reads
0.005
0.004
0.003
0.002
0.001
0.000
ovary
eggs
4-cell, 1 hpf
1k-cell, 3 hpf
5 hpf
bud, 10 hpf
24 hpf
Leu-TAA-3
Leu-TAA-1

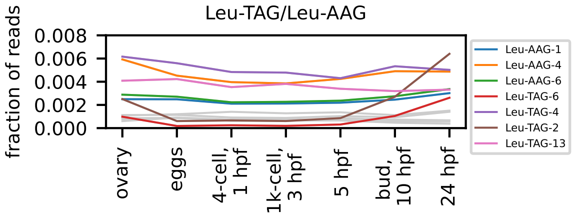

Leu-TAG/Leu-AAG
fraction of reads
0.008
0.006
0.004
0.002
0.000
ovary
eggs
4-cell, 1 hpf
1k-cell, 3 hpf
5 hpf
bud, 10 hpf
24 hpf
Leu-AAG-1
Leu-AAG-4
Leu-AAG-6
Leu-TAG-6
Leu-TAG-4
Leu-TAG-2
Leu-TAG-13

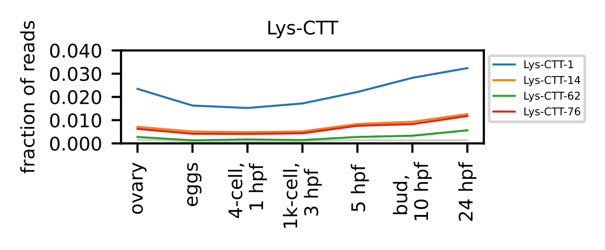

Lys-CTT
fraction of reads
0.040
0.030
0.020
0.010
0.000
ovary
eggs
4-cell, 1 hpf
1k-cell, 3 hpf
5 hpf
bud, 10 hpf
24 hpf
Lys-CTT-1
Lys-CTT-14
Lys-CTT-62
Lys-CTT-76

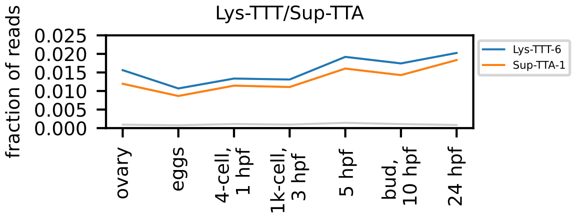

Lys-TTT/Sup-TTA
fraction of reads
0.025
0.020
0.015
0.010
0.005
0.000
Lys-TTT-6
Sup-TTA-1
ovary
eggs
4-cell, 1 hpf
1k-cell, 3 hpf
5 hpf
bud, 10 hpf
24 hpf

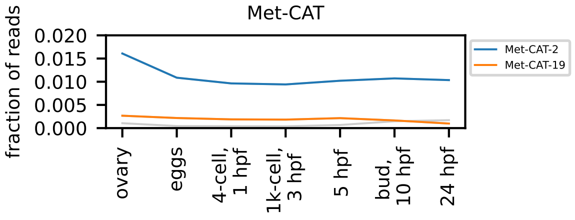

Met-CAT
fraction of reads
0.020
0.015
0.010
0.005
0.000
Met-CAT-2
Met-CAT-19
ovary
eggs
4-cell, 1 hpf
1k-cell, 3 hpf
5 hpf
bud, 10 hpf
24 hpf

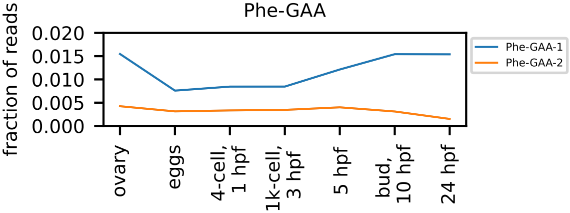

Phe-GAA
fraction of reads
0.020
0.015
0.010
0.005
0.000
Phe-GAA-1
Phe-GAA-2
ovary
eggs
4-cell, 1 hpf
1k-cell, 3 hpf
5 hpf
bud, 10 hpf
24 hpf

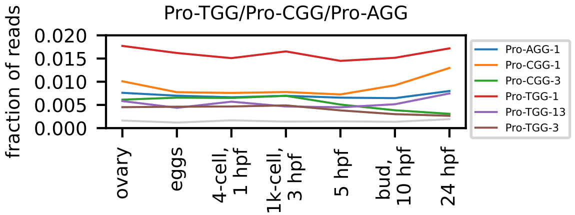

Pro-TGG/Pro-CGG/Pro-AGG
fraction of reads
0.020
0.015
0.010
0.005
0.000
ovary
eggs
4-cell, 1 hpf
1k-cell, 3 hpf
5 hpf
bud, 10 hpf
24 hpf
Pro-AGG-1
Pro-CGG-1
Pro-CGG-3
Pro-TGG-1
Pro-TGG-13
Pro-TGG-3

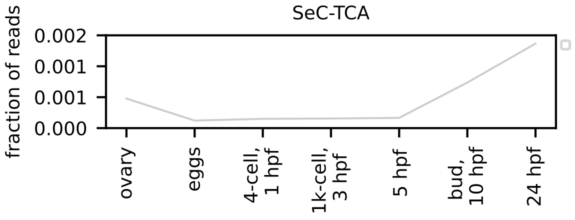
SeC-TCA
fraction of reads
0.002
0.001
0.001
0.000
ovary
eggs
4-cell, 1 hpf
1k-cell, 3 hpf
5 hpf
bud, 10 hpf
24 hpf

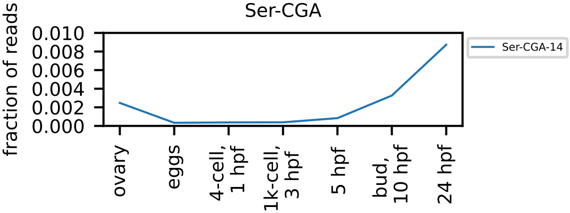
Ser-CGA
fraction of reads
0.010
0.008
0.006
0.004
0.002
0.000
Ser-CGA-14
ovary
eggs
4-cell, 1 hpf
1k-cell, 3 hpf
5 hpf
bud, 10 hpf
24 hpf

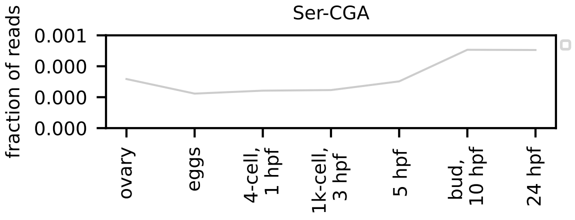
Ser-CGA
fraction of reads
0.001
0.000
0.000
0.000
ovary
eggs
4-cell, 1 hpf
1k-cell, 3 hpf
5 hpf
bud, 10 hpf
24 hpf

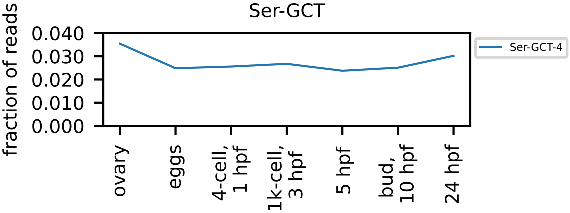

Ser-GCT
fraction of reads
0.040
0.030
0.020
0.010
0.000
Ser-GCT-4
ovary
eggs
4-cell, 1 hpf
1k-cell, 3 hpf
5 hpf
bud, 10 hpf
24 hpf

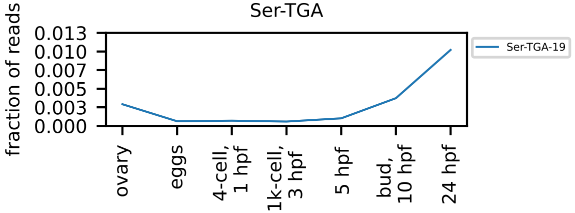

Ser-TGA
fraction of reads
0.013
0.010
0.007
0.005
0.003
0.000
Ser-TGA-19
ovary
eggs
4-cell, 1 hpf
1k-cell, 3 hpf
5 hpf
bud, 10 hpf
24 hpf

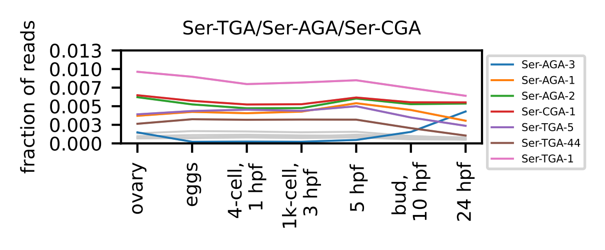
Ser-TGA/Ser-AGA/Ser-CGA
fraction of reads
0.013
0.010
0.007
0.005
0.003
0.000
ovary
eggs
4-cell, 1 hpf
1k-cell, 3 hpf
5 hpf
bud, 10 hpf
24 hpf
Ser-AGA-3
Ser-AGA-1
Ser-AGA-2
Ser-CGA-1
Ser-TGA-5
Ser-TGA-44
Ser-TGA-1

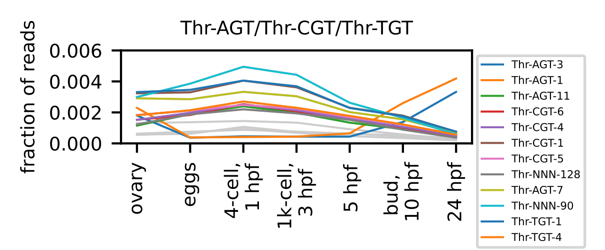

Thr-AGT/Thr-CGT/Thr-TGT
fraction of reads
0.006
0.004
0.002
0.000
ovary
eggs
4-cell, 1 hpf
1k-cell, 3 hpf
5 hpf
bud, 10 hpf
24 hpf
Thr-AGT-3
Thr-AGT-1
Thr-AGT-11
Thr-CGT-6
Thr-CGT-4
Thr-CGT-1
Thr-CGT-5
Thr-NNN-128
Thr-AGT-7
Thr-NNN-90
Thr-TGT-1
Thr-TGT-4

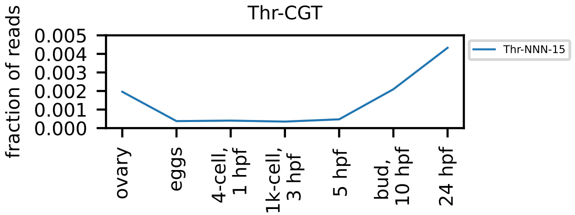

Thr-CGT
fraction of reads
0.005
0.004
0.003
0.002
0.001
0.000
Thr-NNN-15
ovary
eggs
4-cell,
1 hpf
1k-cell,
3 hpf
5 hpf
bud,
10 hpf
24 hpf

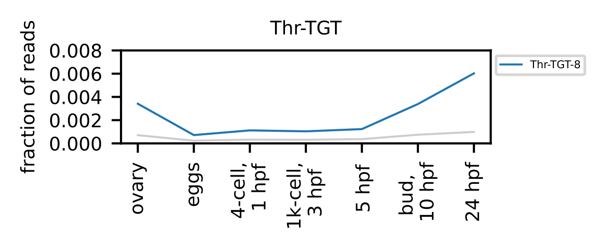

Thr-TGT
fraction of reads
0.008
0.006
0.004
0.002
0.000
Thr-TGT-8
ovary
eggs
4-cell, 1 hpf
1k-cell, 3 hpf
5 hpf
bud, 10 hpf
24 hpf

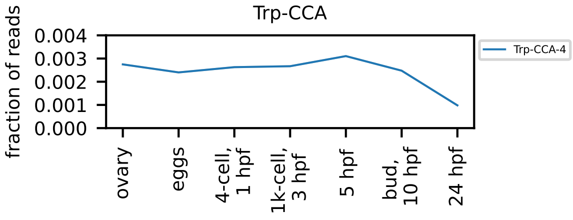

Trp-CCA
fraction of reads
0.004
0.003
0.002
0.001
0.000
ovary
eggs
4-cell, 1 hpf
1k-cell, 3 hpf
5 hpf
bud, 10 hpf
24 hpf
Trp-CCA-4

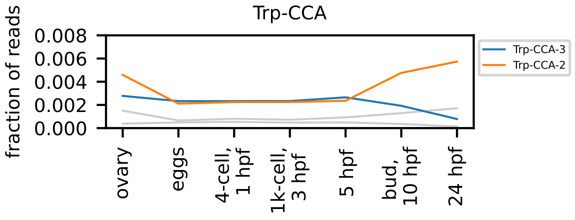

Trp-CCA
fraction of reads
0.008
0.006
0.004
0.002
0.000
Trp-CCA-3
Trp-CCA-2
ovary
eggs
4-cell, 1 hpf
1k-cell, 3 hpf
5 hpf
bud, 10 hpf
24 hpf

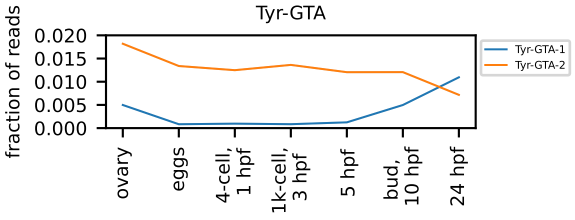

Tyr-GTA
fraction of reads
0.020
0.015
0.010
0.005
0.000
ovary
eggs
4-cell, 1 hpf
1k-cell, 3 hpf
5 hpf
bud, 10 hpf
24 hpf
Tyr-GTA-1
Tyr-GTA-2

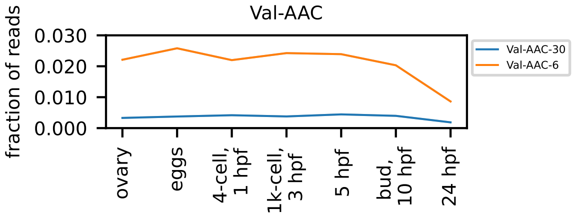

Val-AAC
fraction of reads
0.030
0.020
0.010
0.000
Val-AAC-30
Val-AAC-6
ovary
eggs
4-cell, 1 hpf
1k-cell, 3 hpf
5 hpf
bud, 10 hpf
24 hpf

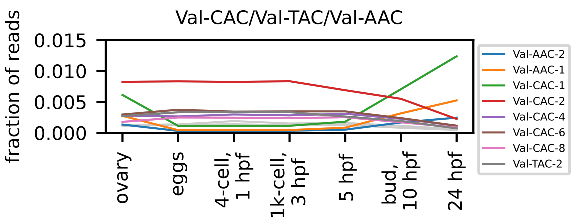
Val-CAC/Val-TAC/Val-AAC
fraction of reads
0.015
0.010
0.005
0.000
ovary
eggs
4-cell, 1 hpf
1k-cell, 3 hpf
5 hpf
bud, 10 hpf
24 hpf
Val-AAC-2
Val-AAC-1
Val-CAC-1
Val-CAC-2
Val-CAC-4
Val-CAC-6
Val-CAC-8
Val-TAC-2

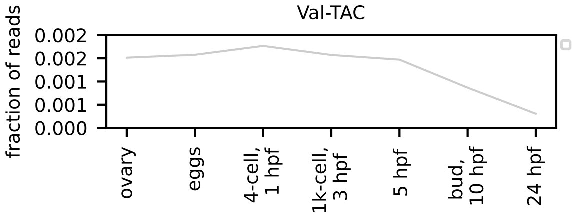
Val-TAC
fraction of reads
0.002
0.002
0.001
0.001
0.000
ovary
eggs
4-cell, 1 hpf
1k-cell, 3 hpf
5 hpf
bud, 10 hpf
24 hpf

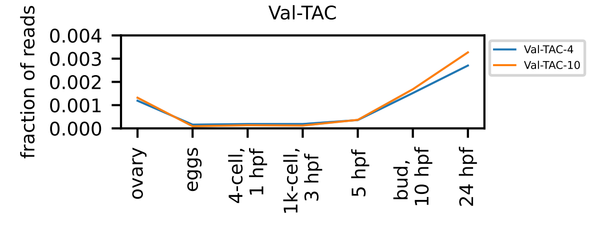
Val-TAC
fraction of reads
0.004
0.003
0.002
0.001
0.000
ovary
eggs
4-cell, 1 hpf
1k-cell, 3 hpf
5 hpf
bud, 10 hpf
24 hpf
Val-TAC-4
Val-TAC-10

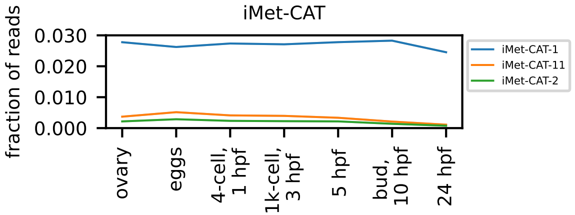
iMet-CAT
fraction of reads
0.030
0.020
0.010
0.000
ovary
eggs
4-cell, 1 hpf
1k-cell, 3 hpf
5 hpf
bud, 10 hpf
24 hpf
iMet-CAT-1
iMet-CAT-11
iMet-CAT-2

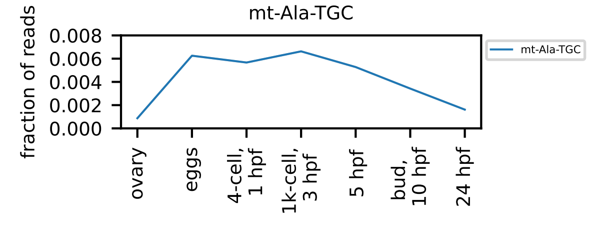
mt-Ala-TGC
fraction of reads
0.008
0.006
0.004
0.002
0.000
mt-Ala-TGC
ovary
eggs
4-cell, 1 hpf
1k-cell, 3 hpf
5 hpf
bud, 10 hpf
24 hpf

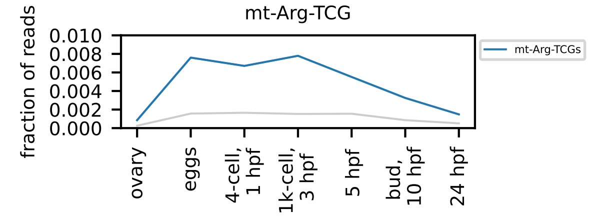
mt-Arg-TCG
fraction of reads
0.010
0.008
0.006
0.004
0.002
0.000
mt-Arg-TCGs
ovary
eggs
4-cell, 1 hpf
1k-cell, 3 hpf
5 hpf
bud, 10 hpf
24 hpf

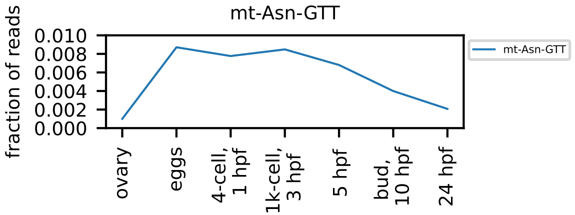

mt-Asn-GTT
fraction of reads
0.010
0.008
0.006
0.004
0.002
0.000
mt-Asn-GTT
ovary
eggs
4-cell, 1 hpf
1k-cell, 3 hpf
5 hpf
bud, 10 hpf
24 hpf

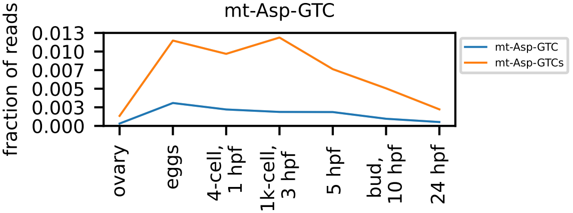
mt-Asp-GTC
fraction of reads
0.013
0.010
0.007
0.005
0.003
0.000
ovary
eggs
4-cell, 1 hpf
1k-cell, 3 hpf
5 hpf
bud, 10 hpf
24 hpf
mt-Asp-GTC
mt-Asp-GTCs

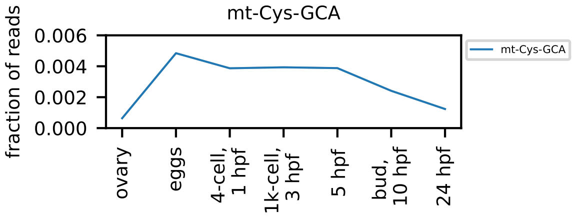
mt-Cys-GCA
fraction of reads
0.006
0.004
0.002
0.000
mt-Cys-GCA
ovary
eggs
4-cell, 1 hpf
1k-cell, 3 hpf
5 hpf
bud, 10 hpf
24 hpf

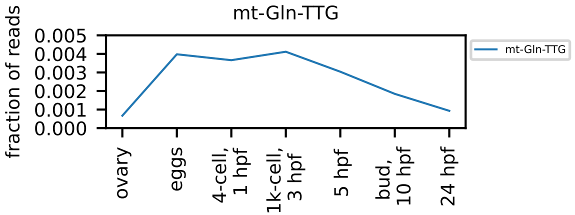
mt-Gln-TTG
fraction of reads
0.005
0.004
0.003
0.002
0.001
0.000
mt-Gln-TTG
ovary
eggs
4-cell, 1 hpf
1k-cell, 3 hpf
5 hpf
bud, 10 hpf
24 hpf

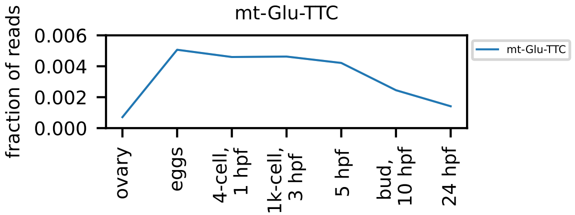

mt-Glu-TTC
fraction of reads
0.006
0.004
0.002
0.000
ovary
eggs
4-cell, 1 hpf
1k-cell, 3 hpf
5 hpf
bud, 10 hpf
24 hpf
mt-Glu-TTC

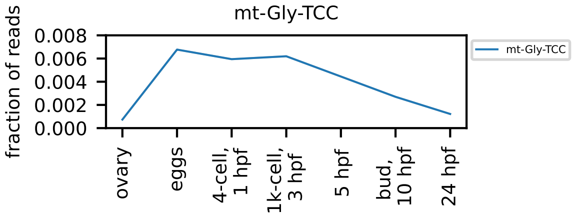
mt-Gly-TCC
fraction of reads
0.008
0.006
0.004
0.002
0.000
mt-Gly-TCC
ovary
eggs
4-cell, 1 hpf
1k-cell, 3 hpf
5 hpf
bud, 10 hpf
24 hpf

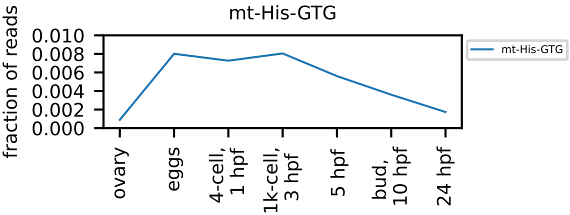
mt-His-GTG
fraction of reads
0.010
0.008
0.006
0.004
0.002
0.000
mt-His-GTG
ovary
eggs
4-cell, 1 hpf
1k-cell, 3 hpf
5 hpf
bud, 10 hpf
24 hpf

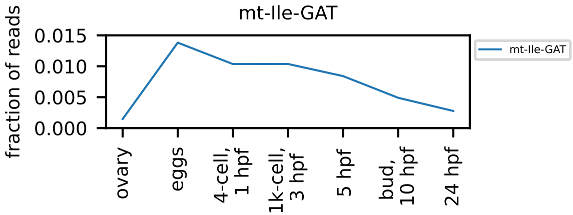
mt-Ile-GAT
fraction of reads
0.015
0.010
0.005
0.000
mt-Ile-GAT
ovary
eggs
4-cell, 1 hpf
1k-cell, 3 hpf
5 hpf
bud, 10 hpf
24 hpf

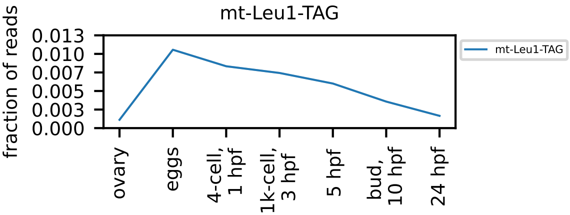

mt-Leu1-TAG
fraction of reads
0.013
0.010
0.007
0.005
0.003
0.000
ovary
eggs
4-cell, 1 hpf
1k-cell, 3 hpf
5 hpf
bud, 10 hpf
24 hpf
mt-Leu1-TAG

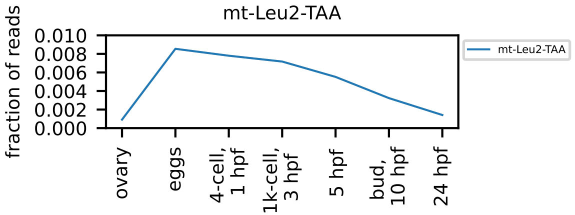

mt-Leu2-TAA
fraction of reads
0.010
0.008
0.006
0.004
0.002
0.000
mt-Leu2-TAA
ovary
eggs
4-cell, 1 hpf
1k-cell, 3 hpf
5 hpf
bud, 10 hpf
24 hpf

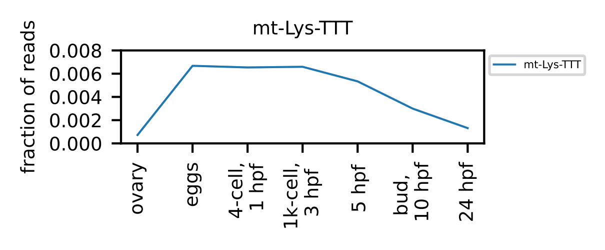

mt-Lys-TTT
fraction of reads
0.008
0.006
0.004
0.002
0.000
ovary
eggs
4-cell,
1 hpf
1k-cell,
3 hpf
5 hpf
bud,
10 hpf
24 hpf
mt-Lys-TTT

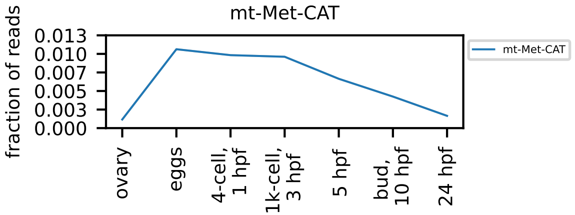

mt-Met-CAT
fraction of reads
0.013
0.010
0.007
0.005
0.003
0.000
mt-Met-CAT
ovary
eggs
4-cell, 1 hpf
1k-cell, 3 hpf
5 hpf
bud, 10 hpf
24 hpf

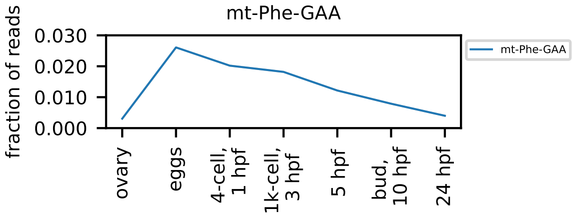

mt-Phe-GAA
fraction of reads
0.030
0.020
0.010
0.000
mt-Phe-GAA
ovary
eggs
4-cell, 1 hpf
1k-cell, 3 hpf
5 hpf
bud, 10 hpf
24 hpf

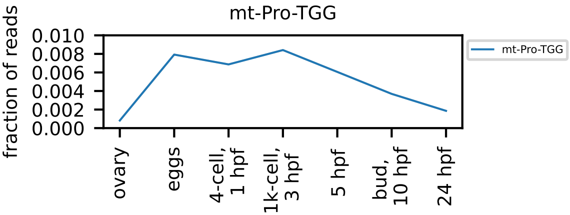
mt-Pro-TGG
fraction of reads
0.010
0.008
0.006
0.004
0.002
0.000
mt-Pro-TGG
ovary
eggs
4-cell, 1 hpf
1k-cell, 3 hpf
5 hpf
bud, 10 hpf
24 hpf

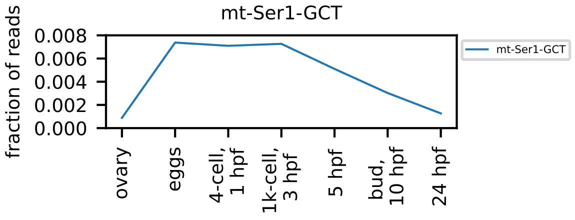

mt-Ser1-GCT
fraction of reads
0.008
0.006
0.004
0.002
0.000
mt-Ser1-GCT
ovary
eggs
4-cell, 1 hpf
1k-cell, 3 hpf
5 hpf
bud, 10 hpf
24 hpf

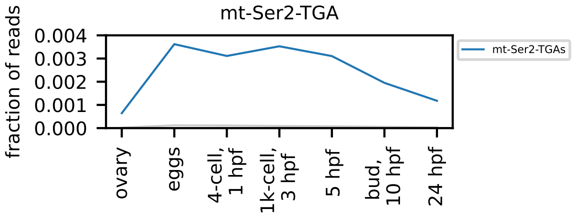

mt-Ser2-TGA
fraction of reads
0.004
0.003
0.002
0.001
0.000
mt-Ser2-TGAs
ovary
eggs
4-cell, 1 hpf
1k-cell, 3 hpf
5 hpf
bud, 10 hpf
24 hpf

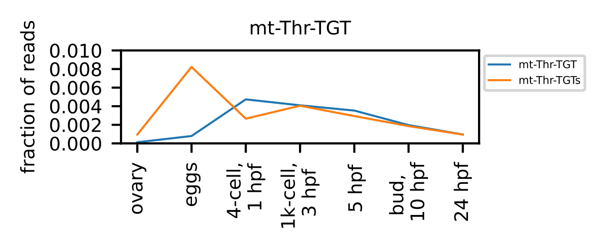
mt-Thr-TGT
fraction of reads
0.010
0.008
0.006
0.004
0.002
0.000
mt-Thr-TGT
mt-Thr-TGTs
ovary
eggs
4-cell, 1 hpf
1k-cell, 3 hpf
5 hpf
bud, 10 hpf
24 hpf

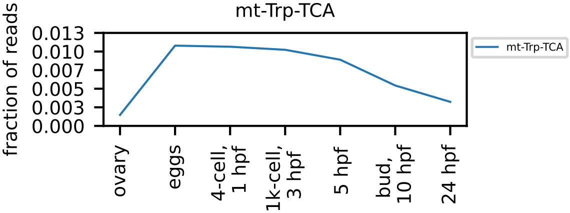
mt-Trp-TCA
fraction of reads
0.013
0.010
0.007
0.005
0.003
0.000
ovary
eggs
4-cell, 1 hpf
1k-cell, 3 hpf
5 hpf
bud, 10 hpf
24 hpf
mt-Trp-TCA

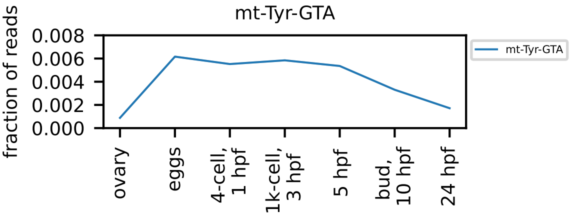
mt-Tyr-GTA
fraction of reads
0.008
0.006
0.004
0.002
0.000
mt-Tyr-GTA
ovary
eggs
4-cell, 1 hpf
1k-cell, 3 hpf
5 hpf
bud, 10 hpf
24 hpf

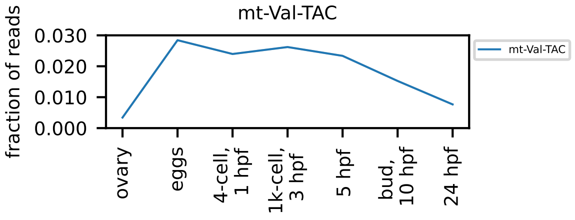

mt-Val-TAC
fraction of reads
0.030
0.020
0.010
0.000
mt-Val-TAC
ovary
eggs
4-cell, 1 hpf
1k-cell, 3 hpf
5 hpf
bud, 10 hpf
24 hpf
