# Supplementary material for: tRNA expression and modification landscapes, and their dynamics during zebrafish embryo development
Source: Nucleic Acids Res. 2024 Jul 11;52(17):10575–94. doi: 10.1093/nar/gkae595 (PMC11417395; doi:10.1093/nar/gkae595)
Supplement: gkae595_Supplemental_Files [file gkae595_supplemental_files.zip › Supplementary_file_6-modification dynamics scatterplots all-mock-v3.pdf]

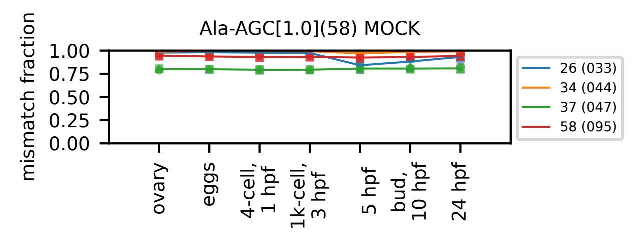

Ala-AGC[1.0](58) MOCK
mismatch fraction
1.00
0.75
0.50
0.25
0.00
ovary
eggs
4-cell, 1 hpf
1k-cell, 3 hpf
5 hpf
bud, 10 hpf
24 hpf
26 (033)
34 (044)
37 (047)
58 (095)

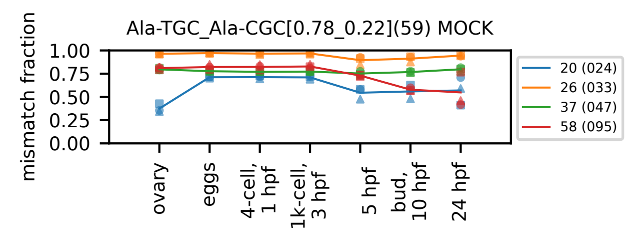

Ala-TGC_Ala-CGC[0.78_0.22](59) MOCK
mismatch fraction
1.00
0.75
0.50
0.25
0.00
ovary
eggs
4-cell, 1 hpf
1k-cell, 3 hpf
5 hpf
bud, 10 hpf
24 hpf
20 (024)
26 (033)
37 (047)
58 (095)

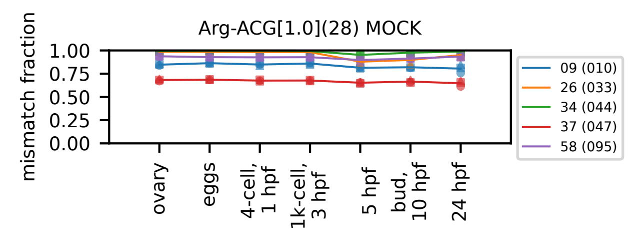

Arg-ACG[1.0](28) MOCK
mismatch fraction
1.00
0.75
0.50
0.25
0.00
ovary
eggs
4-cell, 1 hpf
1k-cell, 3 hpf
5 hpf
bud, 10 hpf
24 hpf
09 (010)
26 (033)
34 (044)
37 (047)
58 (095)

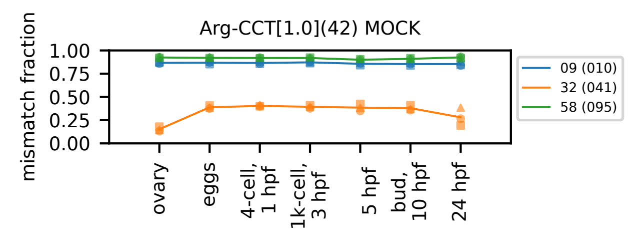

Arg-CCT[1.0](42) MOCK
mismatch fraction
1.00
0.75
0.50
0.25
0.00
ovary
eggs
4-cell, 1 hpf
1k-cell, 3 hpf
5 hpf
bud, 10 hpf
24 hpf
09 (010)
32 (041)
58 (095)

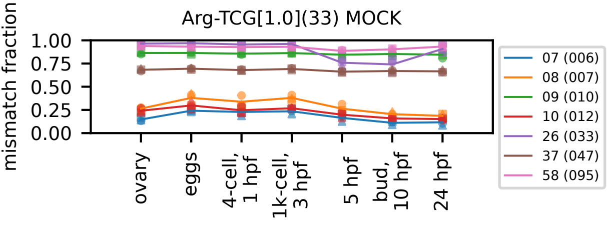

Arg-TCG[1.0](33) MOCK
mismatch fraction
1.00
0.75
0.50
0.25
0.00
ovary
eggs
4-cell, 1 hpf
1k-cell, 3 hpf
5 hpf
bud, 10 hpf
24 hpf
07 (006)
08 (007)
09 (010)
10 (012)
26 (033)
37 (047)
58 (095)

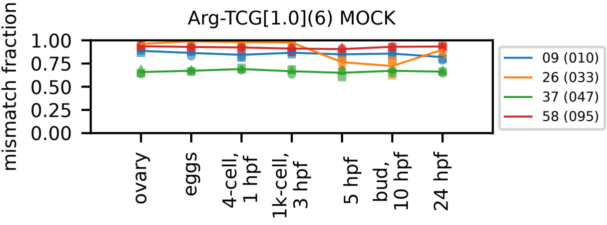

Arg-TCG[1.0](6) MOCK
mismatch fraction
1.00
0.75
0.50
0.25
0.00
ovary
eggs
4-cell, 1 hpf
1k-cell, 3 hpf
5 hpf
bud, 10 hpf
24 hpf
09 (010)
26 (033)
37 (047)
58 (095)

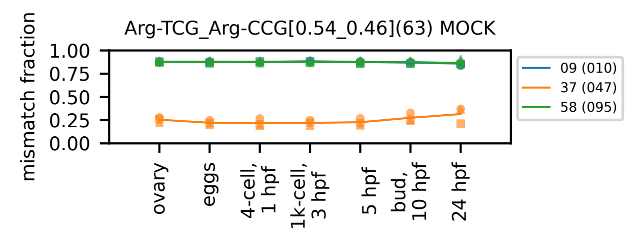
Arg-TCG_Arg-CCG[0.54_0.46](63) MOCK
mismatch fraction
1.00
0.75
0.50
0.25
0.00
ovary
eggs
4-cell, 1 hpf
1k-cell, 3 hpf
5 hpf
bud, 10 hpf
24 hpf
09 (010)
37 (047)
58 (095)

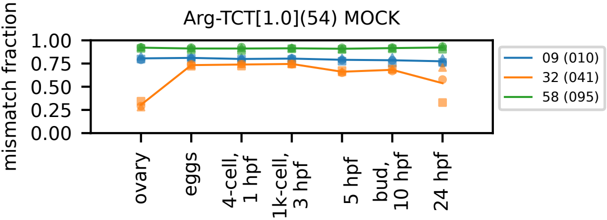

Arg-TCT[1.0](54) MOCK
mismatch fraction
1.00
0.75
0.50
0.25
0.00
ovary
eggs
4-cell, 1 hpf
1k-cell, 3 hpf
5 hpf
bud, 10 hpf
24 hpf
09 (010)
32 (041)
58 (095)

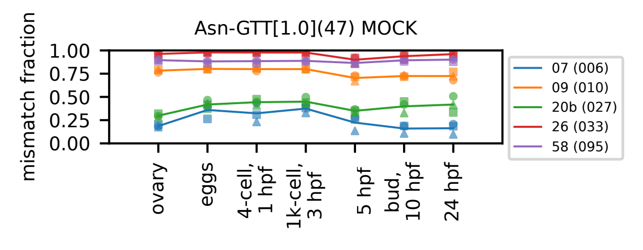

Asn-GTT[1.0](47) MOCK
mismatch fraction
1.00
0.75
0.50
0.25
0.00
ovary
eggs
4-cell, 1 hpf
1k-cell, 3 hpf
5 hpf
bud, 10 hpf
24 hpf
07 (006)
09 (010)
20b (027)
26 (033)
58 (095)

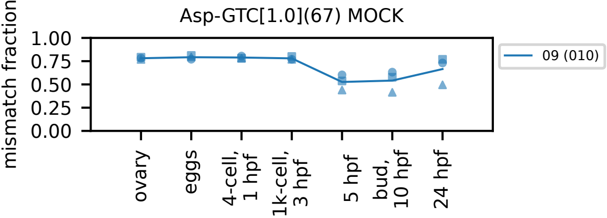

Asp-GTC[1.0](67) MOCK
mismatch fraction
1.00
0.75
0.50
0.25
0.00
ovary
eggs
4-cell, 1 hpf
1k-cell, 3 hpf
5 hpf
bud, 10 hpf
24 hpf
09 (010)

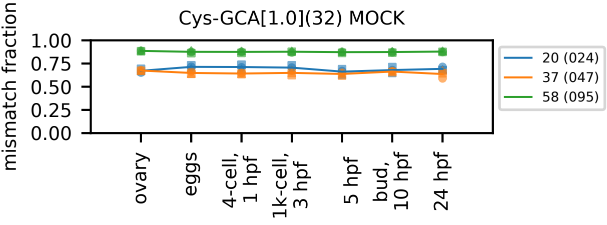

Cys-GCA[1.0](32) MOCK
mismatch fraction
1.00
0.75
0.50
0.25
0.00
ovary
eggs
4-cell, 1 hpf
1k-cell, 3 hpf
5 hpf
bud, 10 hpf
24 hpf
20 (024)
37 (047)
58 (095)

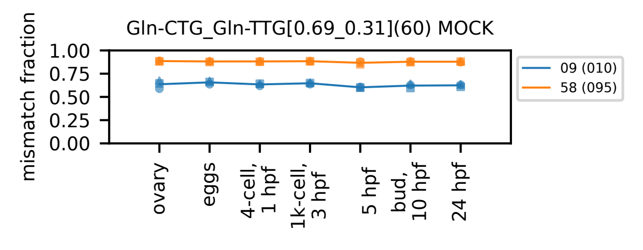

Gln-CTG_Gln-TTG[0.69_0.31](60) MOCK
mismatch fraction
1.00
0.75
0.50
0.25
0.00
ovary
eggs
4-cell, 1 hpf
1k-cell, 3 hpf
5 hpf
bud, 10 hpf
24 hpf
09 (010)
58 (095)

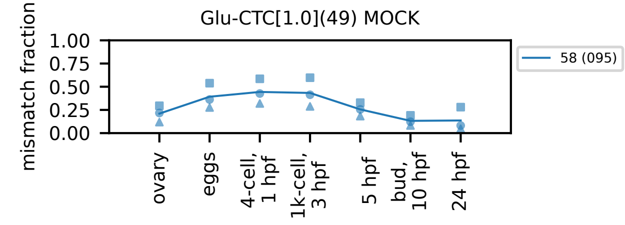

Glu-CTC[1.0](49) MOCK
mismatch fraction
1.00
0.75
0.50
0.25
0.00
ovary
eggs
4-cell, 1 hpf
1k-cell, 3 hpf
5 hpf
bud, 10 hpf
24 hpf
58 (095)

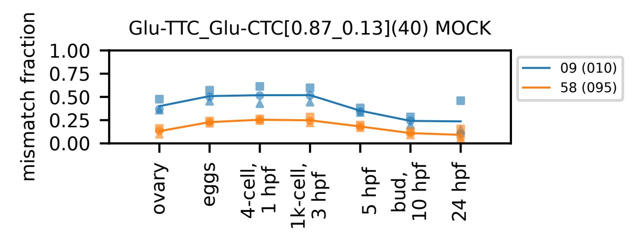

Glu-TTC_Glu-CTC[0.87_0.13](40) MOCK
mismatch fraction
1.00
0.75
0.50
0.25
0.00
ovary
eggs
4-cell, 1 hpf
1k-cell, 3 hpf
5 hpf
bud, 10 hpf
24 hpf
09 (010)
58 (095)

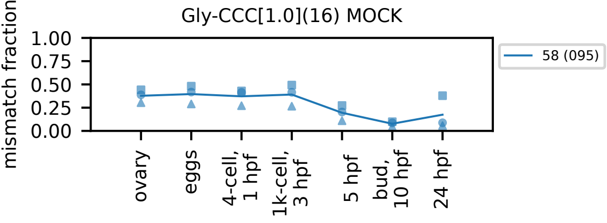

Gly-CCC[1.0](16) MOCK
mismatch fraction
1.00
0.75
0.50
0.25
0.00
58 (095)
ovary
eggs
4-cell, 1 hpf
1k-cell, 3 hpf
5 hpf
bud, 10 hpf
24 hpf

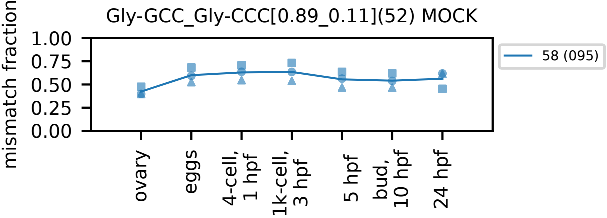

Gly-GCC_Gly-CCC[0.89_0.11](52) MOCK
mismatch fraction
1.00
0.75
0.50
0.25
0.00
ovary
eggs
4-cell, 1 hpf
1k-cell, 3 hpf
5 hpf
bud, 10 hpf
24 hpf
58 (095)

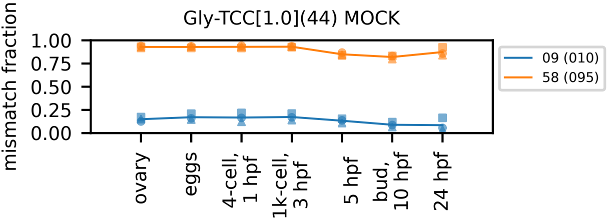

Gly-TCC[1.0](44) MOCK
mismatch fraction
1.00
0.75
0.50
0.25
0.00
ovary
eggs
4-cell, 1 hpf
1k-cell, 3 hpf
5 hpf
bud, 10 hpf
24 hpf
09 (010)
58 (095)

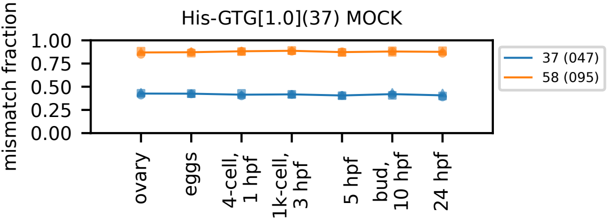

His-GTG[1.0](37) MOCK
mismatch fraction
1.00
0.75
0.50
0.25
0.00
ovary
eggs
4-cell, 1 hpf
1k-cell, 3 hpf
5 hpf
bud, 10 hpf
24 hpf
37 (047)
58 (095)

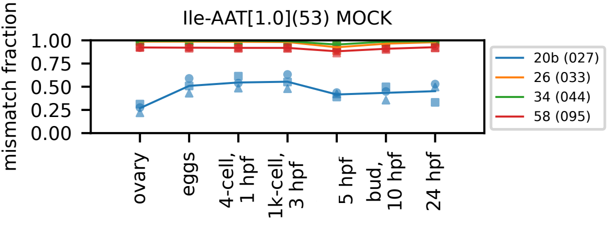

Ile-AAT[1.0](53) MOCK
mismatch fraction
1.00
0.75
0.50
0.25
0.00
ovary
eggs
4-cell, 1 hpf
1k-cell, 3 hpf
5 hpf
bud, 10 hpf
24 hpf
20b (027)
26 (033)
34 (044)
58 (095)

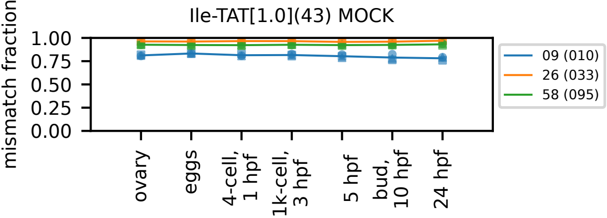
Ile-TAT[1.0](43) MOCK
mismatch fraction
1.00
0.75
0.50
0.25
0.00
ovary
eggs
4-cell, 1 hpf
1k-cell, 3 hpf
5 hpf
bud, 10 hpf
24 hpf
09 (010)
26 (033)
58 (095)

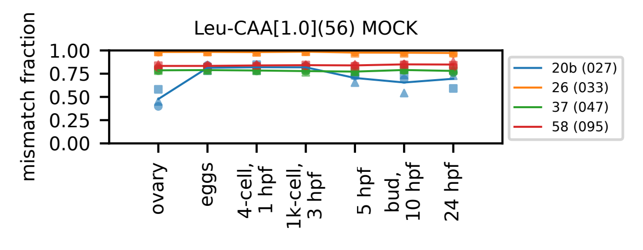

Leu-CAA[1.0](56) MOCK
mismatch fraction
1.00
0.75
0.50
0.25
0.00
ovary
eggs
4-cell, 1 hpf
1k-cell, 3 hpf
5 hpf
bud, 10 hpf
24 hpf
20b (027)
26 (033)
37 (047)
58 (095)

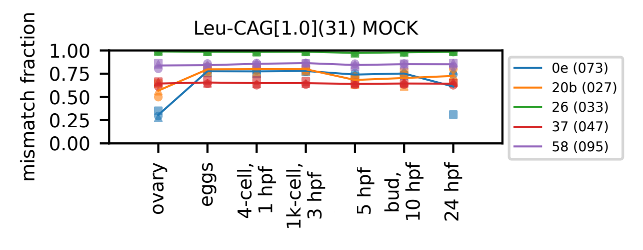
Leu-CAG[1.0](31) MOCK
mismatch fraction
1.00
0.75
0.50
0.25
0.00
ovary
eggs
4-cell, 1 hpf
1k-cell, 3 hpf
5 hpf
bud, 10 hpf
24 hpf
0e (073)
20b (027)
26 (033)
37 (047)
58 (095)

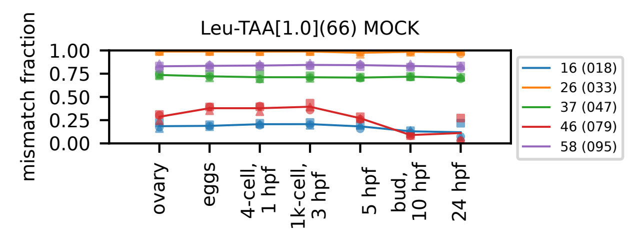

Leu-TAA[1.0](66) MOCK
mismatch fraction
1.00
0.75
0.50
0.25
0.00
ovary
eggs
4-cell, 1 hpf
1k-cell, 3 hpf
5 hpf
bud, 10 hpf
24 hpf
16 (018)
26 (033)
37 (047)
46 (079)
58 (095)

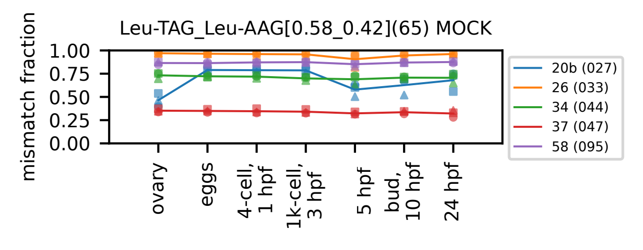
Leu-TAG_Leu-AAG[0.58_0.42](65) MOCK
mismatch fraction
1.00
0.75
0.50
0.25
0.00
ovary
eggs
4-cell, 1 hpf
1k-cell, 3 hpf
5 hpf
bud, 10 hpf
24 hpf
20b (027)
26 (033)
34 (044)
37 (047)
58 (095)

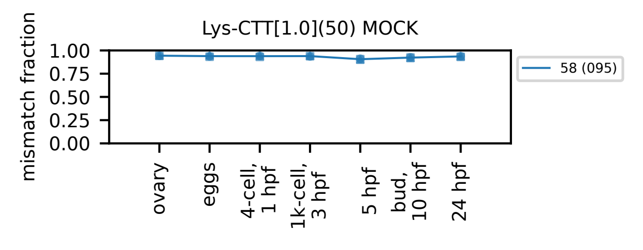
Lys-CTT[1.0](50) MOCK
mismatch fraction
1.00
0.75
0.50
0.25
0.00
ovary
eggs
4-cell, 1 hpf
1k-cell, 3 hpf
5 hpf
bud, 10 hpf
24 hpf
58 (095)

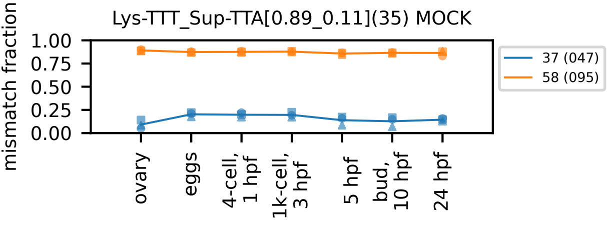

Lys-TTT_Sup-TTA[0.89_0.11](35) MOCK
mismatch fraction
1.00
0.75
0.50
0.25
0.00
ovary
eggs
4-cell, 1 hpf
1k-cell, 3 hpf
5 hpf
bud, 10 hpf
24 hpf
37 (047)
58 (095)

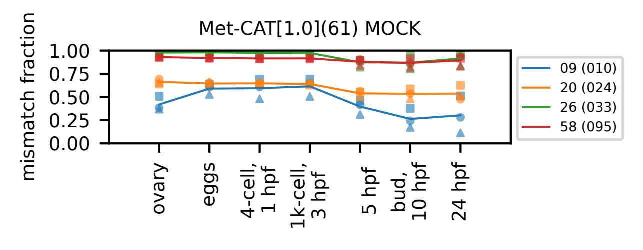
Met-CAT[1.0](61) MOCK
mismatch fraction
1.00
0.75
0.50
0.25
0.00
ovary
eggs
4-cell, 1 hpf
1k-cell, 3 hpf
5 hpf
bud, 10 hpf
24 hpf
09 (010)
20 (024)
26 (033)
58 (095)

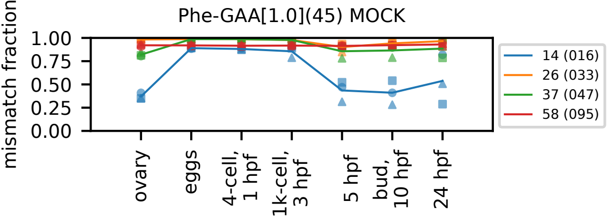

Phe-GAA[1.0](45) MOCK
mismatch fraction
1.00
0.75
0.50
0.25
0.00
ovary
eggs
4-cell, 1 hpf
1k-cell, 3 hpf
5 hpf
bud, 10 hpf
24 hpf
14 (016)
26 (033)
37 (047)
58 (095)

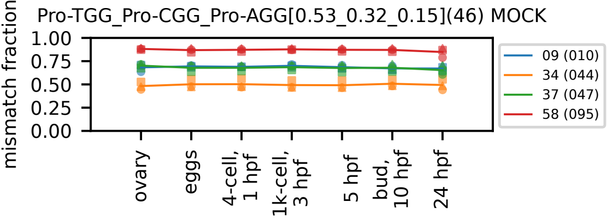
Pro-TGG_Pro-CGG_Pro-AGG[0.53_0.32_0.15](46) MOCK
mismatch fraction
1.00
0.75
0.50
0.25
0.00
ovary
eggs
4-cell, 1 hpf
1k-cell, 3 hpf
5 hpf
bud, 10 hpf
24 hpf
09 (010)
34 (044)
37 (047)
58 (095)

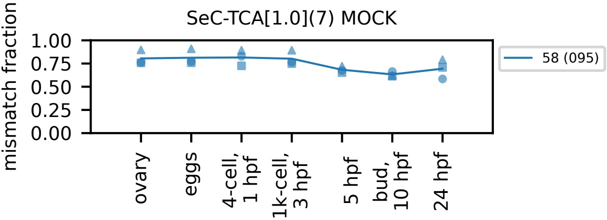

SeC-TCA[1.0](7) MOCK
mismatch fraction
1.00
0.75
0.50
0.25
0.00
ovary
eggs
4-cell, 1 hpf
1k-cell, 3 hpf
5 hpf
bud, 10 hpf
24 hpf
58 (095)

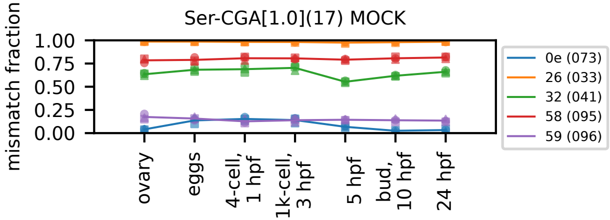

Ser-CGA[1.0](17) MOCK
mismatch fraction
1.00
0.75
0.50
0.25
0.00
ovary
eggs
4-cell, 1 hpf
1k-cell, 3 hpf
5 hpf
bud, 10 hpf
24 hpf
0e (073)
26 (033)
32 (041)
58 (095)
59 (096)

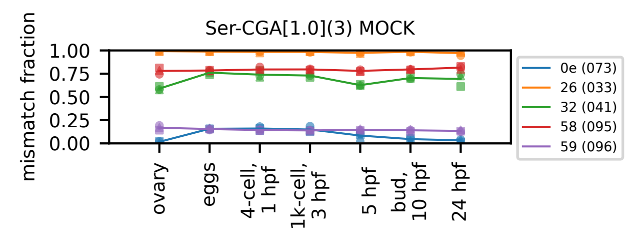

Ser-CGA[1.0](3) MOCK
mismatch fraction
1.00
0.75
0.50
0.25
0.00
ovary
eggs
4-cell, 1 hpf
1k-cell, 3 hpf
5 hpf
bud, 10 hpf
24 hpf
0e (073)
26 (033)
32 (041)
58 (095)
59 (096)

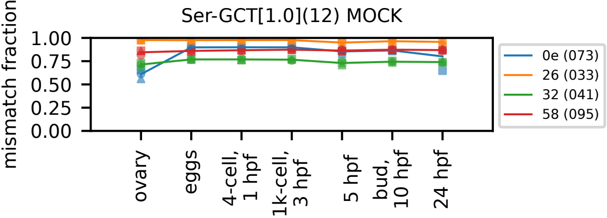

Ser-GCT[1.0](12) MOCK
mismatch fraction
1.00
0.75
0.50
0.25
0.00
ovary
eggs
4-cell, 1 hpf
1k-cell, 3 hpf
5 hpf
bud, 10 hpf
24 hpf
0e (073)
26 (033)
32 (041)
58 (095)

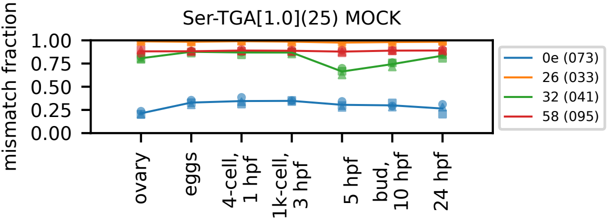
Ser-TGA[1.0](25) MOCK
mismatch fraction
1.00
0.75
0.50
0.25
0.00
ovary
eggs
4-cell, 1 hpf
1k-cell, 3 hpf
5 hpf
bud, 10 hpf
24 hpf
0e (073)
26 (033)
32 (041)
58 (095)

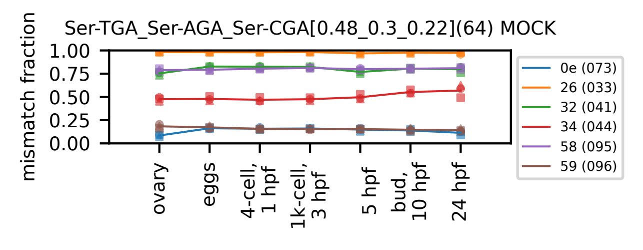
Ser-TGA_Ser-AGA_Ser-CGA[0.48_0.3_0.22](64) MOCK
mismatch fraction
1.00
0.75
0.50
0.25
0.00
ovary
eggs
4-cell, 1 hpf
1k-cell, 3 hpf
5 hpf
bud, 10 hpf
24 hpf
0e (073)
26 (033)
32 (041)
34 (044)
58 (095)
59 (096)

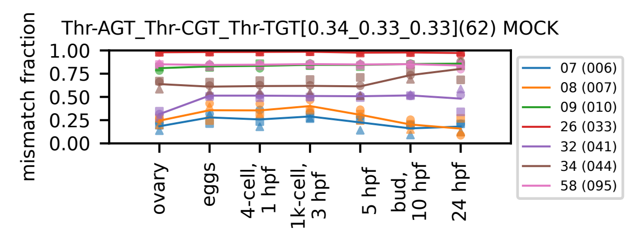
Thr-AGT_Thr-CGT_Thr-TGT[0.34_0.33_0.33](62) MOCK
mismatch fraction
1.00
0.75
0.50
0.25
0.00
ovary
eggs
4-cell, 1 hpf
1k-cell, 3 hpf
5 hpf
bud, 10 hpf
24 hpf
07 (006)
08 (007)
09 (010)
26 (033)
32 (041)
34 (044)
58 (095)

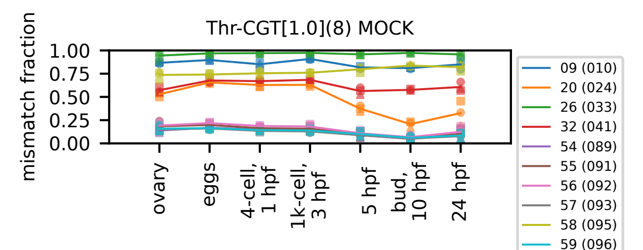

Thr-CGT[1.0](8) MOCK
mismatch fraction
1.00
0.75
0.50
0.25
0.00
ovary
eggs
4-cell, 1 hpf
1k-cell, 3 hpf
5 hpf
bud, 10 hpf
24 hpf
09 (010)
20 (024)
26 (033)
32 (041)
54 (089)
55 (091)
56 (092)
57 (093)
58 (095)
59 (096)

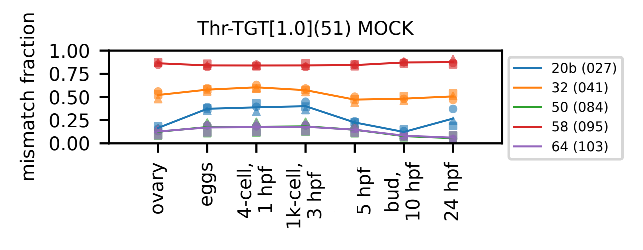

Thr-TGT[1.0](51) MOCK
mismatch fraction
1.00
0.75
0.50
0.25
0.00
ovary
eggs
4-cell, 1 hpf
1k-cell, 3 hpf
5 hpf
bud, 10 hpf
24 hpf
20b (027)
32 (041)
50 (084)
58 (095)
64 (103)

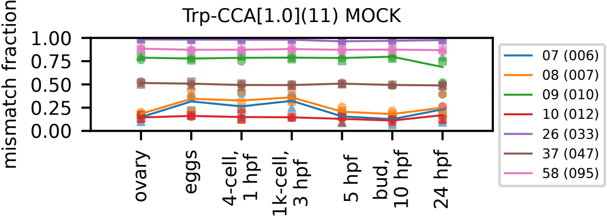

Trp-CCA[1.0](11) MOCK
mismatch fraction
1.00
0.75
0.50
0.25
0.00
ovary
eggs
4-cell, 1 hpf
1k-cell, 3 hpf
5 hpf
bud, 10 hpf
24 hpf
07 (006)
08 (007)
09 (010)
10 (012)
26 (033)
37 (047)
58 (095)

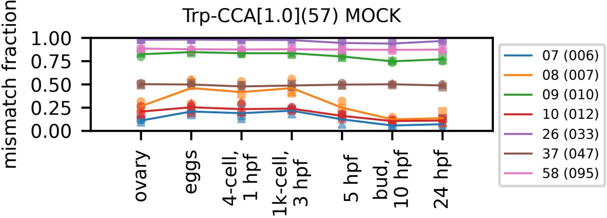

Trp-CCA[1.0](57) MOCK
mismatch fraction
1.00
0.75
0.50
0.25
0.00
ovary
eggs
4-cell, 1 hpf
1k-cell, 3 hpf
5 hpf
bud, 10 hpf
24 hpf
07 (006)
08 (007)
09 (010)
10 (012)
26 (033)
37 (047)
58 (095)

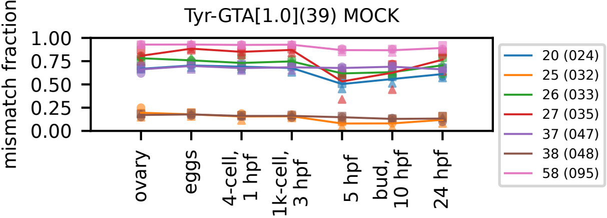

Tyr-GTA[1.0](39) MOCK
mismatch fraction
1.00
0.75
0.50
0.25
0.00
ovary
eggs
4-cell, 1 hpf
1k-cell, 3 hpf
5 hpf
bud, 10 hpf
24 hpf
20 (024)
25 (032)
26 (033)
27 (035)
37 (047)
38 (048)
58 (095)

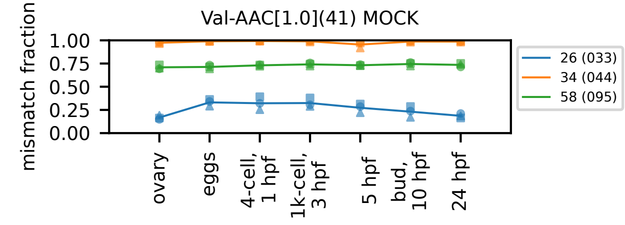

Val-AAC[1.0](41) MOCK
mismatch fraction
1.00
0.75
0.50
0.25
0.00
ovary
eggs
4-cell, 1 hpf
1k-cell, 3 hpf
5 hpf
bud, 10 hpf
24 hpf
26 (033)
34 (044)
58 (095)

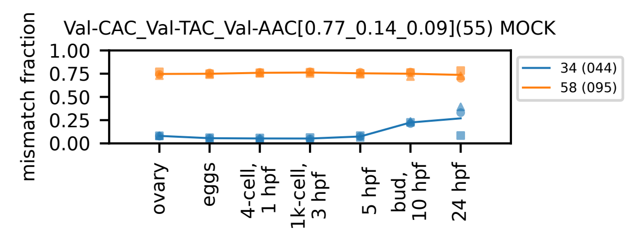

Val-CAC_Val-TAC_Val-AAC[0.77_0.14_0.09](55) MOCK
mismatch fraction
1.00
0.75
0.50
0.25
0.00
ovary
eggs
4-cell, 1 hpf
1k-cell, 3 hpf
5 hpf
bud, 10 hpf
24 hpf
34 (044)
58 (095)

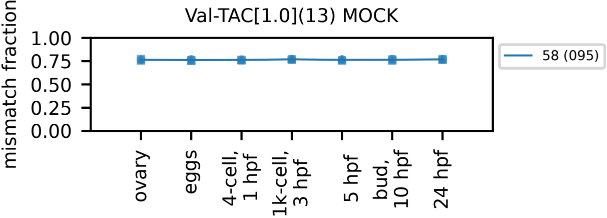

Val-TAC[1.0](13) MOCK
mismatch fraction
1.00
0.75
0.50
0.25
0.00
ovary
eggs
4-cell, 1 hpf
1k-cell, 3 hpf
5 hpf
bud, 10 hpf
24 hpf
58 (095)

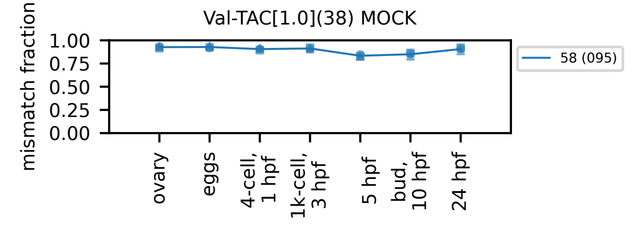

Val-TAC[1.0](38) MOCK
mismatch fraction
1.00
0.75
0.50
0.25
0.00
ovary
eggs
4-cell, 1 hpf
1k-cell, 3 hpf
5 hpf
bud, 10 hpf
24 hpf
58 (095)

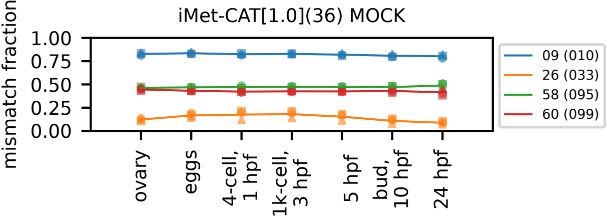
iMet-CAT[1.0](36) MOCK
mismatch fraction
1.00
0.75
0.50
0.25
0.00
ovary
eggs
4-cell, 1 hpf
1k-cell, 3 hpf
5 hpf
bud, 10 hpf
24 hpf
09 (010)
26 (033)
58 (095)
60 (099)

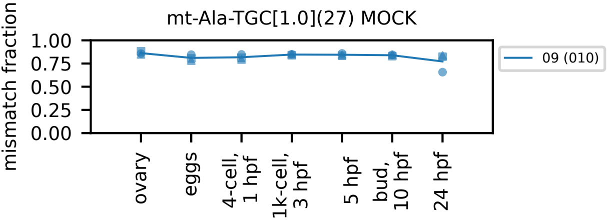

mt-Ala-TGC[1.0](27) MOCK
mismatch fraction
1.00
0.75
0.50
0.25
0.00
ovary
eggs
4-cell, 1 hpf
1k-cell, 3 hpf
5 hpf
bud, 10 hpf
24 hpf
09 (010)

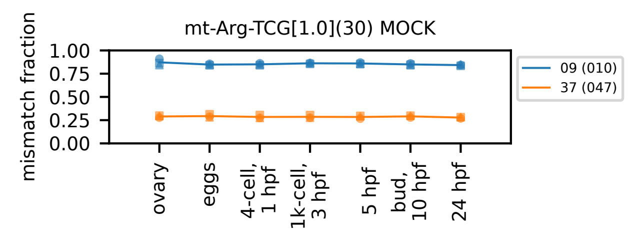
mt-Arg-TCG[1.0](30) MOCK
mismatch fraction
1.00
0.75
0.50
0.25
0.00
ovary
eggs
4-cell, 1 hpf
1k-cell, 3 hpf
5 hpf
bud, 10 hpf
24 hpf
09 (010)
37 (047)

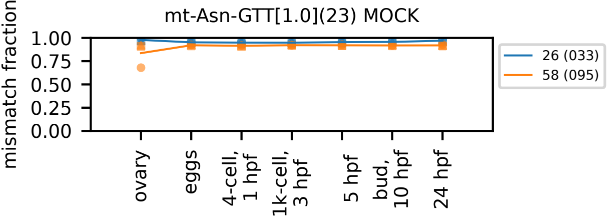
mt-Asn-GTT[1.0](23) MOCK
mismatch fraction
1.00
0.75
0.50
0.25
0.00
ovary
eggs
4-cell, 1 hpf
1k-cell, 3 hpf
5 hpf
bud, 10 hpf
24 hpf
26 (033)
58 (095)

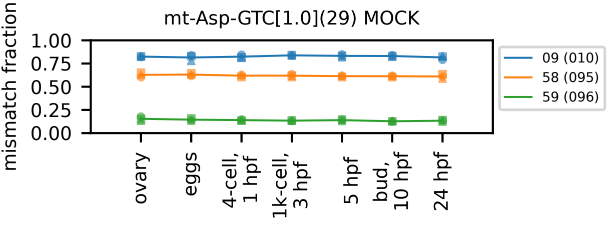
mt-Asp-GTC[1.0](29) MOCK
mismatch fraction
1.00
0.75
0.50
0.25
0.00
ovary
eggs
4-cell, 1 hpf
1k-cell, 3 hpf
5 hpf
bud, 10 hpf
24 hpf
09 (010)
58 (095)
59 (096)

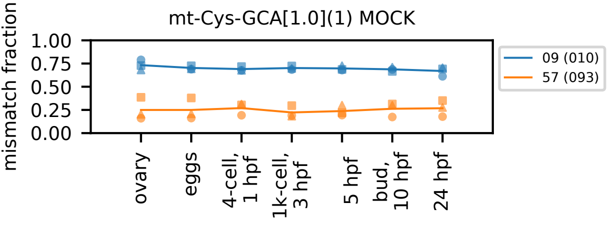

mt-Cys-GCA[1.0](1) MOCK
mismatch fraction
1.00
0.75
0.50
0.25
0.00
ovary
eggs
4-cell, 1 hpf
1k-cell, 3 hpf
5 hpf
bud, 10 hpf
24 hpf
09 (010)
57 (093)

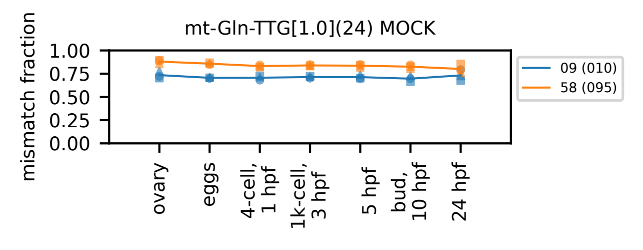

mt-Gln-TTG[1.0](24) MOCK
mismatch fraction
1.00
0.75
0.50
0.25
0.00
ovary
eggs
4-cell, 1 hpf
1k-cell, 3 hpf
5 hpf
bud, 10 hpf
24 hpf
09 (010)
58 (095)

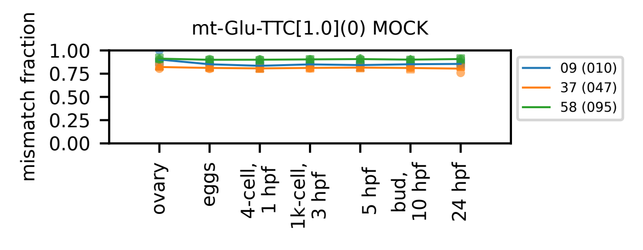
mt-Glu-TTC[1.0](0) MOCK
mismatch fraction
1.00
0.75
0.50
0.25
0.00
ovary
eggs
4-cell, 1 hpf
1k-cell, 3 hpf
5 hpf
bud, 10 hpf
24 hpf
09 (010)
37 (047)
58 (095)

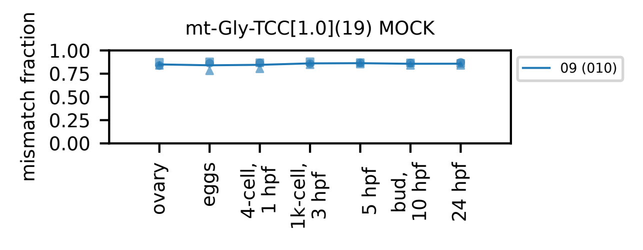
mt-Gly-TCC[1.0](19) MOCK
mismatch fraction
1.00
0.75
0.50
0.25
0.00
ovary
eggs
4-cell, 1 hpf
1k-cell, 3 hpf
5 hpf
bud, 10 hpf
24 hpf
09 (010)

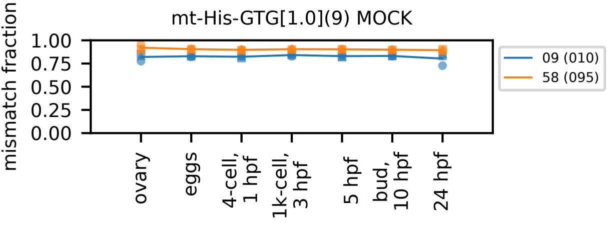

mt-His-GTG[1.0](9) MOCK
mismatch fraction
1.00
0.75
0.50
0.25
0.00
ovary
eggs
4-cell, 1 hpf
1k-cell, 3 hpf
5 hpf
bud, 10 hpf
24 hpf
09 (010)
58 (095)

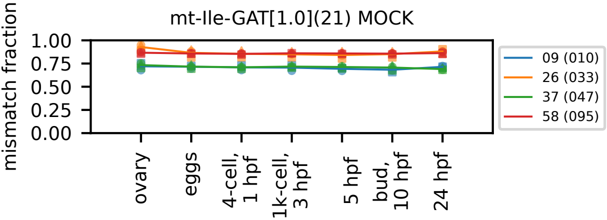

mt-Ile-GAT[1.0](21) MOCK
mismatch fraction
1.00
0.75
0.50
0.25
0.00
ovary
eggs
4-cell, 1 hpf
1k-cell, 3 hpf
5 hpf
bud, 10 hpf
24 hpf
09 (010)
26 (033)
37 (047)
58 (095)

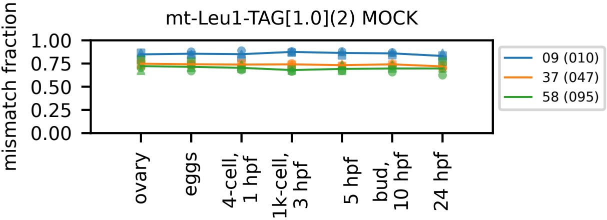
mt-Leu1-TAG[1.0](2) MOCK
mismatch fraction
1.00
0.75
0.50
0.25
0.00
ovary
eggs
4-cell, 1 hpf
1k-cell, 3 hpf
5 hpf
bud, 10 hpf
24 hpf
09 (010)
37 (047)
58 (095)

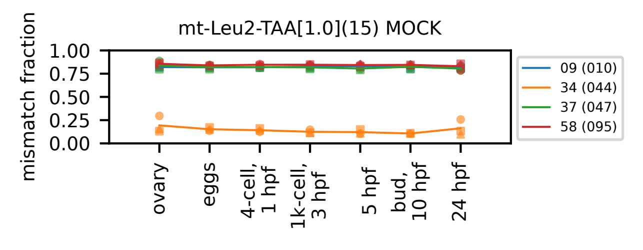
mt-Leu2-TAA[1.0](15) MOCK
mismatch fraction
1.00
0.75
0.50
0.25
0.00
ovary
eggs
4-cell, 1 hpf
1k-cell, 3 hpf
5 hpf
bud, 10 hpf
24 hpf
09 (010)
34 (044)
37 (047)
58 (095)

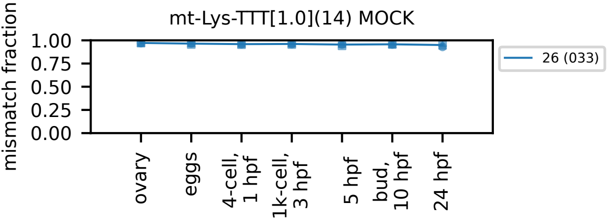
mt-Lys-TTT[1.0](14) MOCK
mismatch fraction
1.00
0.75
0.50
0.25
0.00
ovary
eggs
4-cell, 1 hpf
1k-cell, 3 hpf
5 hpf
bud, 10 hpf
24 hpf
26 (033)

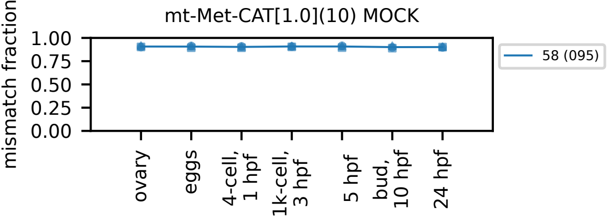
mt-Met-CAT[1.0](10) MOCK
mismatch fraction
1.00
0.75
0.50
0.25
0.00
ovary
eggs
4-cell, 1 hpf
1k-cell, 3 hpf
5 hpf
bud, 10 hpf
24 hpf
58 (095)

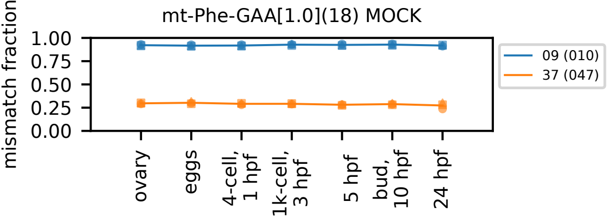

mt-Phe-GAA[1.0](18) MOCK
mismatch fraction
1.00
0.75
0.50
0.25
0.00
ovary
eggs
4-cell, 1 hpf
1k-cell, 3 hpf
5 hpf
bud, 10 hpf
24 hpf
09 (010)
37 (047)

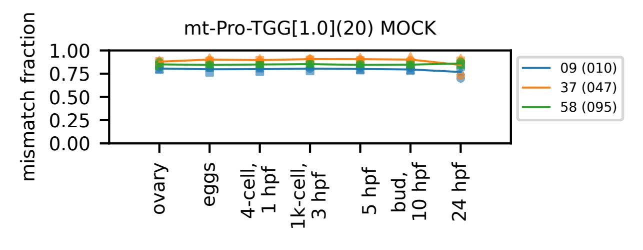

mt-Pro-TGG[1.0](20) MOCK
mismatch fraction
1.00
0.75
0.50
0.25
0.00
ovary
eggs
4-cell, 1 hpf
1k-cell, 3 hpf
5 hpf
bud, 10 hpf
24 hpf
09 (010)
37 (047)
58 (095)

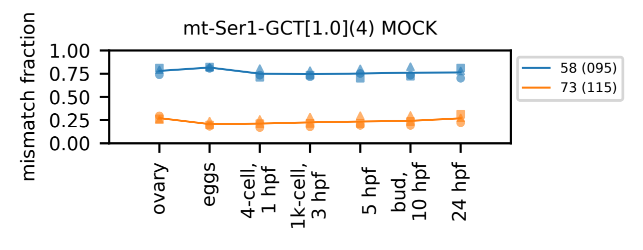
mt-Ser1-GCT[1.0](4) MOCK
mismatch fraction
1.00
0.75
0.50
0.25
0.00
ovary
eggs
4-cell, 1 hpf
1k-cell, 3 hpf
5 hpf
bud, 10 hpf
24 hpf
58 (095)
73 (115)

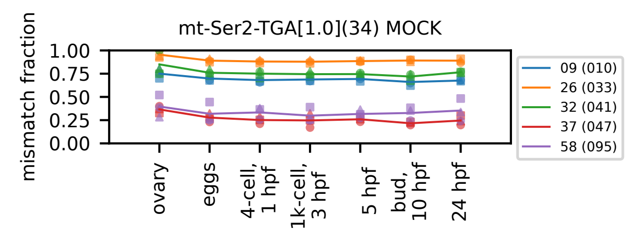

mt-Ser2-TGA[1.0](34) MOCK
mismatch fraction
1.00
0.75
0.50
0.25
0.00
ovary
eggs
4-cell, 1 hpf
1k-cell, 3 hpf
5 hpf
bud, 10 hpf
24 hpf
09 (010)
26 (033)
32 (041)
37 (047)
58 (095)

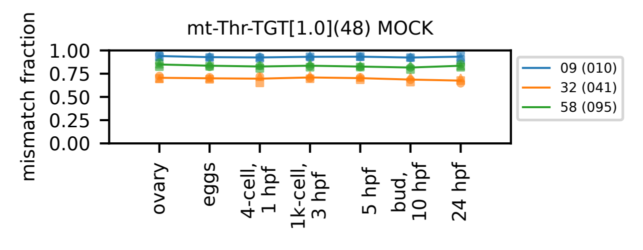

mt-Thr-TGT[1.0](48) MOCK
mismatch fraction
1.00
0.75
0.50
0.25
0.00
ovary
eggs
4-cell, 1 hpf
1k-cell, 3 hpf
5 hpf
bud, 10 hpf
24 hpf
09 (010)
32 (041)
58 (095)

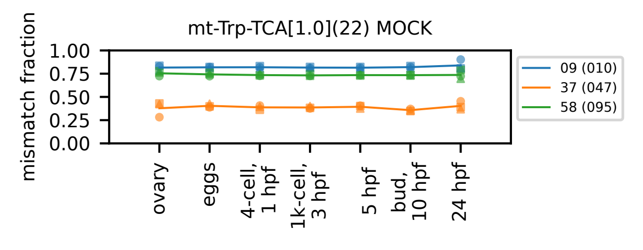

mt-Trp-TCA[1.0](22) MOCK
mismatch fraction
1.00
0.75
0.50
0.25
0.00
ovary
eggs
4-cell, 1 hpf
1k-cell, 3 hpf
5 hpf
bud, 10 hpf
24 hpf
09 (010)
37 (047)
58 (095)

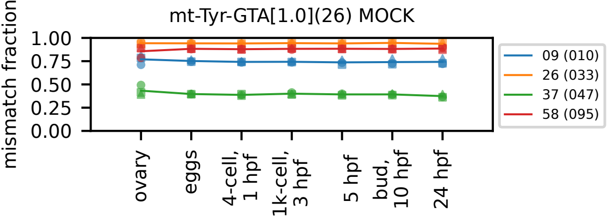

mt-Tyr-GTA[1.0](26) MOCK
mismatch fraction
1.00
0.75
0.50
0.25
0.00
ovary
eggs
4-cell, 1 hpf
1k-cell, 3 hpf
5 hpf
bud, 10 hpf
24 hpf
09 (010)
26 (033)
37 (047)
58 (095)

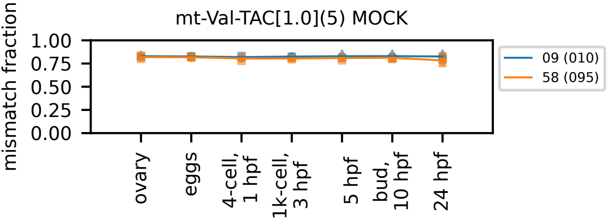

mt-Val-TAC[1.0](5) MOCK
mismatch fraction
1.00
0.75
0.50
0.25
0.00
ovary
eggs
4-cell, 1 hpf
1k-cell, 3 hpf
5 hpf
bud, 10 hpf
24 hpf
09 (010)
58 (095)
