# Supplementary material for: tRNA expression and modification landscapes, and their dynamics during zebrafish embryo development
Source: Nucleic Acids Res. 2024 Jul 11;52(17):10575–94. doi: 10.1093/nar/gkae595 (PMC11417395; doi:10.1093/nar/gkae595)
Supplement: gkae595_Supplemental_Files [file gkae595_supplemental_files.zip › Supplementary_file_7-m5C dynamics scatterplots all-BS-v3.pdf]

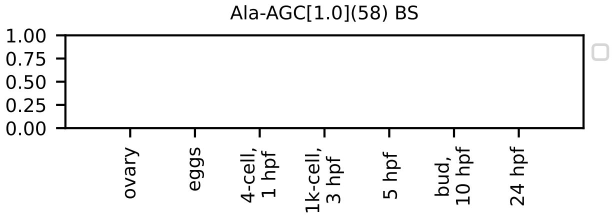

Ala-AGC[1.0](58) BS
1.00
0.75
0.50
0.25
0.00
ovary
eggs
4-cell,
1 hpf
1k-cell,
3 hpf
5 hpf
bud,
10 hpf
24 hpf

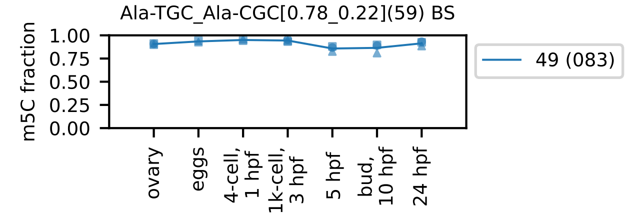
Ala-TGC_Ala-CGC[0.78_0.22](59) BS
m5C fraction
1.00
0.75
0.50
0.25
0.00
ovary
eggs
4-cell, 1 hpf
1k-cell, 3 hpf
5 hpf
bud, 10 hpf
24 hpf
49 (083)

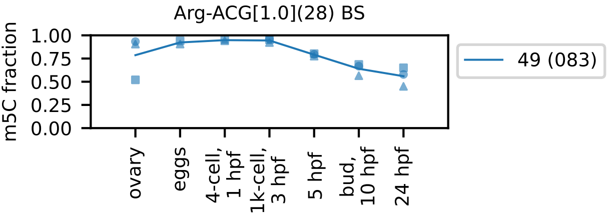

Arg-ACG[1.0](28) BS
m5C fraction
1.00
0.75
0.50
0.25
0.00
ovary
eggs
4-cell, 1 hpf
1k-cell, 3 hpf
5 hpf
bud, 10 hpf
24 hpf
49 (083)

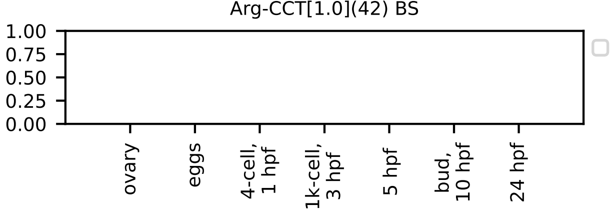
Arg-CCT[1.0](42) BS
1.00
0.75
0.50
0.25
0.00
ovary
eggs
4-cell, 1 hpf
1k-cell, 3 hpf
5 hpf
bud, 10 hpf
24 hpf

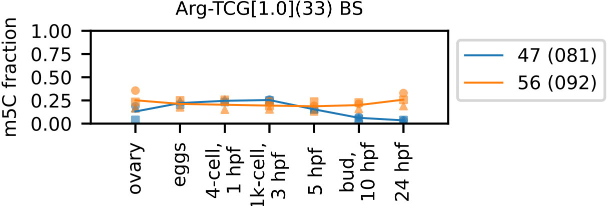
Arg-TCG[1.0](33) BS
m5C fraction
1.00
0.75
0.50
0.25
0.00
ovary
eggs
4-cell, 1 hpf
1k-cell, 3 hpf
5 hpf
bud, 10 hpf
24 hpf
47 (081)
56 (092)

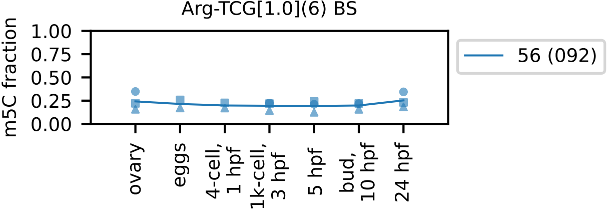
Arg-TCG[1.0](6) BS
m5C fraction
1.00
0.75
0.50
0.25
0.00
ovary
eggs
4-cell, 1 hpf
1k-cell, 3 hpf
5 hpf
bud, 10 hpf
24 hpf
56 (092)

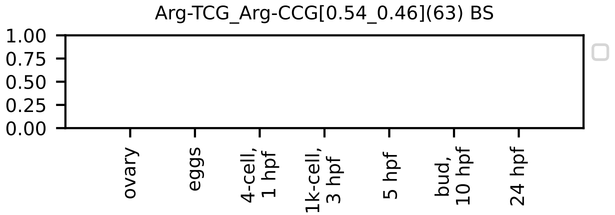

Arg-TCG_Arg-CCG[0.54_0.46](63) BS
1.00
0.75
0.50
0.25
0.00
ovary
eggs
4-cell, 1 hpf
1k-cell, 3 hpf
5 hpf
bud, 10 hpf
24 hpf

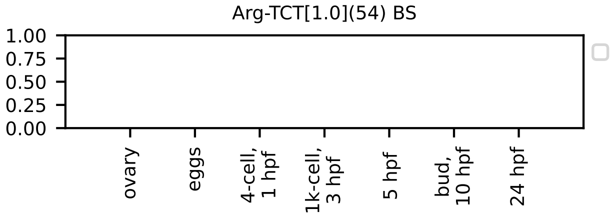
Arg-TCT[1.0](54) BS
1.00
0.75
0.50
0.25
0.00
ovary
eggs
4-cell, 1 hpf
1k-cell, 3 hpf
5 hpf
bud, 10 hpf
24 hpf

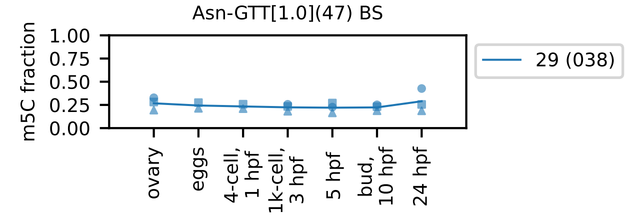
Asn-GTT[1.0](47) BS
m5C fraction
1.00
0.75
0.50
0.25
0.00
ovary
eggs
4-cell, 1 hpf
1k-cell, 3 hpf
5 hpf
bud, 10 hpf
24 hpf
29 (038)

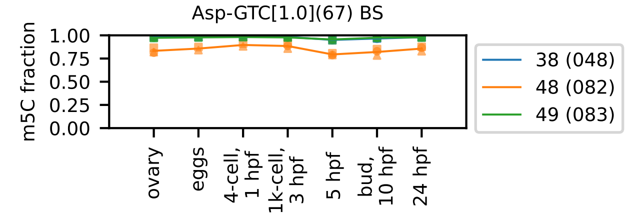

Asp-GTC[1.0](67) BS
m5C fraction
1.00
0.75
0.50
0.25
0.00
ovary
eggs
4-cell, 1 hpf
1k-cell, 3 hpf
5 hpf
bud, 10 hpf
24 hpf
38 (048)
48 (082)
49 (083)

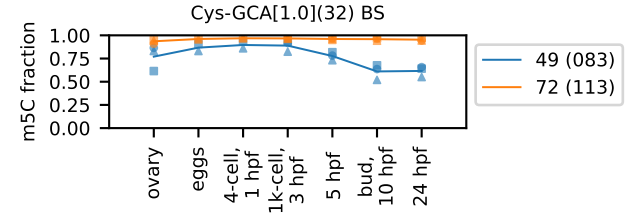

Cys-GCA[1.0](32) BS
m5C fraction
1.00
0.75
0.50
0.25
0.00
ovary
eggs
4-cell, 1 hpf
1k-cell, 3 hpf
5 hpf
bud, 10 hpf
24 hpf
49 (083)
72 (113)

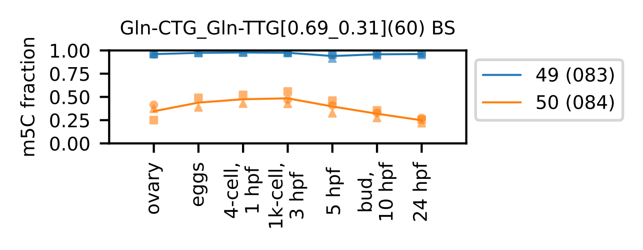

Gln-CTG_Gln-TTG[0.69_0.31](60) BS
m5C fraction
1.00
0.75
0.50
0.25
0.00
ovary
eggs
4-cell, 1 hpf
1k-cell, 3 hpf
5 hpf
bud, 10 hpf
24 hpf
49 (083)
50 (084)

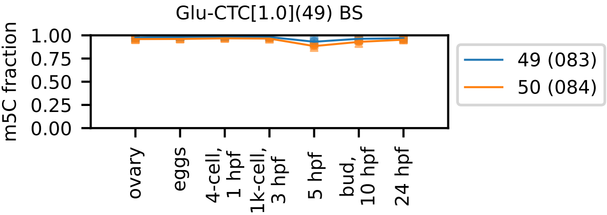
Glu-CTC[1.0](49) BS
m5C fraction
1.00
0.75
0.50
0.25
0.00
ovary
eggs
4-cell, 1 hpf
1k-cell, 3 hpf
5 hpf
bud, 10 hpf
24 hpf
49 (083)
50 (084)

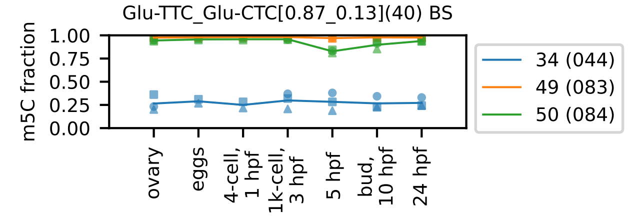
Glu-TTC_Glu-CTC[0.87_0.13](40) BS
m5C fraction
1.00
0.75
0.50
0.25
0.00
ovary
eggs
4-cell, 1 hpf
1k-cell, 3 hpf
5 hpf
bud, 10 hpf
24 hpf
34 (044)
49 (083)
50 (084)

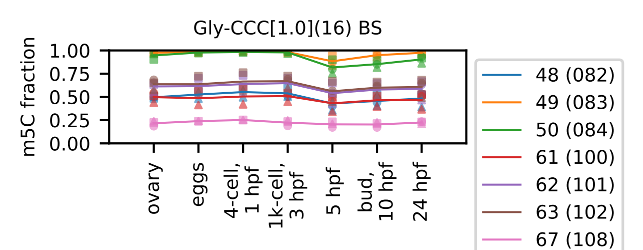

Gly-CCC[1.0](16) BS
m5C fraction
1.00
0.75
0.50
0.25
0.00
ovary
eggs
4-cell, 1 hpf
1k-cell, 3 hpf
5 hpf
bud, 10 hpf
24 hpf
48 (082)
49 (083)
50 (084)
61 (100)
62 (101)
63 (102)
67 (108)

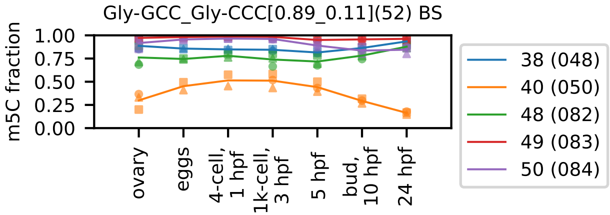

Gly-GCC_Gly-CCC[0.89_0.11](52) BS
m5C fraction
1.00
0.75
0.50
0.25
0.00
ovary
eggs
4-cell, 1 hpf
1k-cell, 3 hpf
5 hpf
bud, 10 hpf
24 hpf
38 (048)
40 (050)
48 (082)
49 (083)
50 (084)

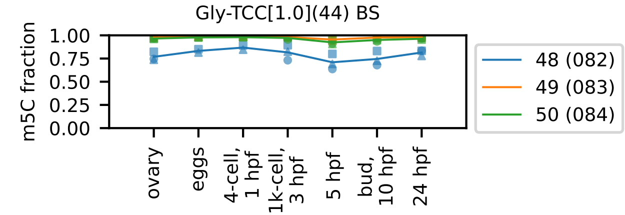
Gly-TCC[1.0](44) BS
m5C fraction
1.00
0.75
0.50
0.25
0.00
ovary
eggs
4-cell, 1 hpf
1k-cell, 3 hpf
5 hpf
bud, 10 hpf
24 hpf
48 (082)
49 (083)
50 (084)

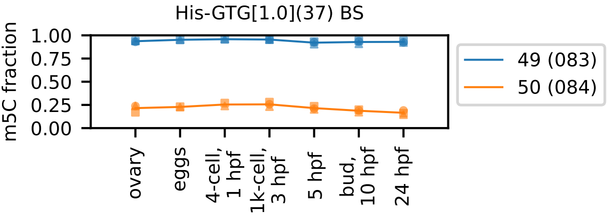
His-GTG[1.0](37) BS
m5C fraction
1.00
0.75
0.50
0.25
0.00
ovary
eggs
4-cell, 1 hpf
1k-cell, 3 hpf
5 hpf
bud, 10 hpf
24 hpf
49 (083)
50 (084)

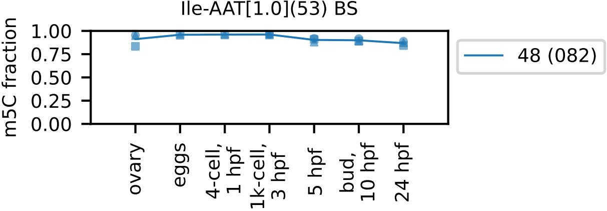
Ile-AAT[1.0](53) BS
m5C fraction
1.00
0.75
0.50
0.25
0.00
ovary
eggs
4-cell, 1 hpf
1k-cell, 3 hpf
5 hpf
bud, 10 hpf
24 hpf
48 (082)

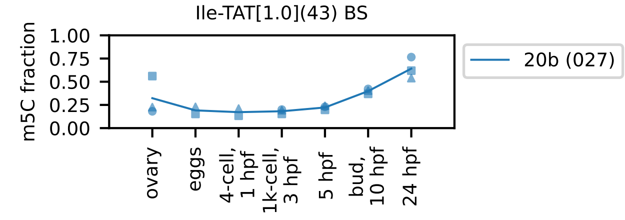
Ile-TAT[1.0](43) BS
m5C fraction
1.00
0.75
0.50
0.25
0.00
ovary
eggs
4-cell, 1 hpf
1k-cell, 3 hpf
5 hpf
bud, 10 hpf
24 hpf
20b (027)

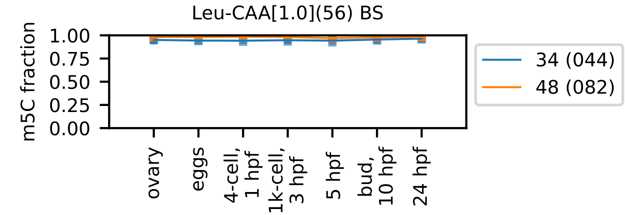
Leu-CAA[1.0](56) BS
m5C fraction
1.00
0.75
0.50
0.25
0.00
ovary
eggs
4-cell, 1 hpf
1k-cell, 3 hpf
5 hpf
bud, 10 hpf
24 hpf
34 (044)
48 (082)

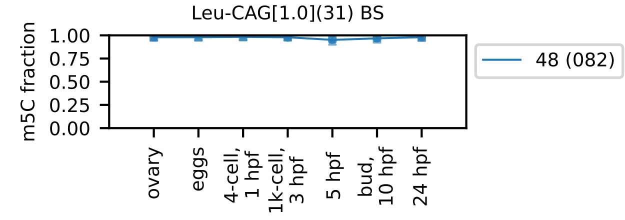
Leu-CAG[1.0](31) BS
m5C fraction
1.00
0.75
0.50
0.25
0.00
ovary
eggs
4-cell, 1 hpf
1k-cell, 3 hpf
5 hpf
bud, 10 hpf
24 hpf
48 (082)

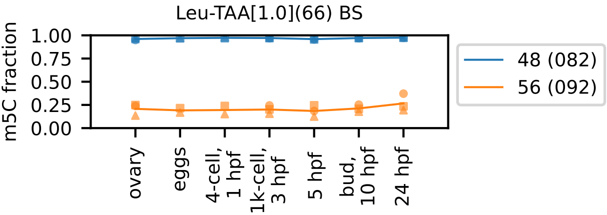

Leu-TAA[1.0](66) BS
m5C fraction
1.00
0.75
0.50
0.25
0.00
ovary
eggs
4-cell, 1 hpf
1k-cell, 3 hpf
5 hpf
bud, 10 hpf
24 hpf
48 (082)
56 (092)

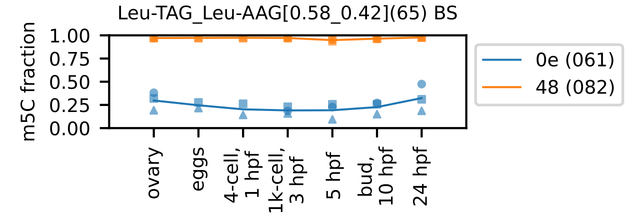
Leu-TAG_Leu-AAG[0.58_0.42](65) BS
m5C fraction
1.00
0.75
0.50
0.25
0.00
ovary
eggs
4-cell, 1 hpf
1k-cell, 3 hpf
5 hpf
bud, 10 hpf
24 hpf
0e (061)
48 (082)

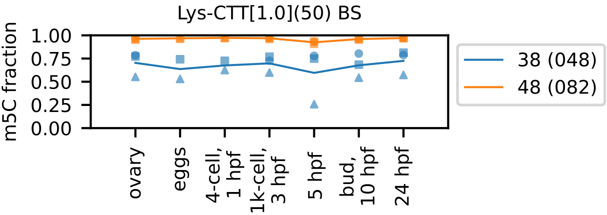
Lys-CTT[1.0](50) BS
m5C fraction
1.00
0.75
0.50
0.25
0.00
ovary
eggs
4-cell, 1 hpf
1k-cell, 3 hpf
5 hpf
bud, 10 hpf
24 hpf
38 (048)
48 (082)

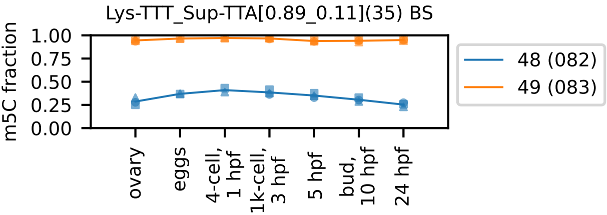
Lys-TTT_Sup-TTA[0.89_0.11](35) BS
m5C fraction
1.00
0.75
0.50
0.25
0.00
ovary
eggs
4-cell, 1 hpf
1k-cell, 3 hpf
5 hpf
bud, 10 hpf
24 hpf
48 (082)
49 (083)

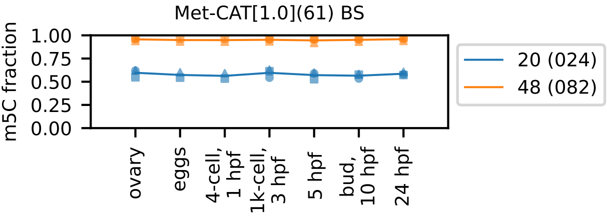
Met-CAT[1.0](61) BS
m5C fraction
1.00
0.75
0.50
0.25
0.00
ovary
eggs
4-cell, 1 hpf
1k-cell, 3 hpf
5 hpf
bud, 10 hpf
24 hpf
20 (024)
48 (082)

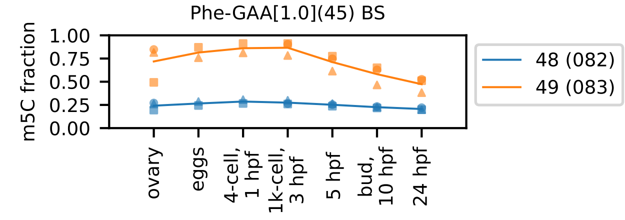
Phe-GAA[1.0](45) BS
m5C fraction
1.00
0.75
0.50
0.25
0.00
ovary
eggs
4-cell, 1 hpf
1k-cell, 3 hpf
5 hpf
bud, 10 hpf
24 hpf
48 (082)
49 (083)

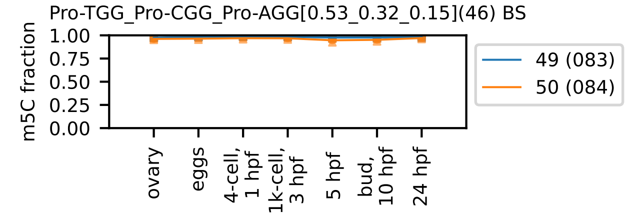

Pro-TGG_Pro-CGG_Pro-AGG[0.53_0.32_0.15](46) BS
m5C fraction
1.00
0.75
0.50
0.25
0.00
ovary
eggs
4-cell, 1 hpf
1k-cell, 3 hpf
5 hpf
bud, 10 hpf
24 hpf
49 (083)
50 (084)

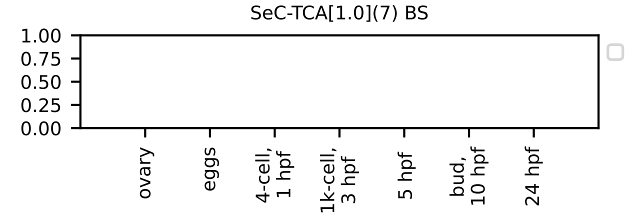

SeC-TCA[1.0](7) BS
1.00
0.75
0.50
0.25
0.00
ovary
eggs
4-cell, 1 hpf
1k-cell, 3 hpf
5 hpf
bud, 10 hpf
24 hpf

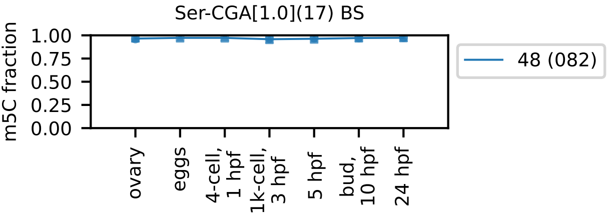

Ser-CGA[1.0](17) BS
m5C fraction
1.00
0.75
0.50
0.25
0.00
ovary
eggs
4-cell, 1 hpf
1k-cell, 3 hpf
5 hpf
bud, 10 hpf
24 hpf
48 (082)

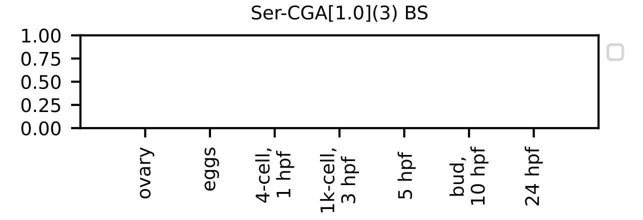

Ser-CGA[1.0](3) BS
1.00
0.75
0.50
0.25
0.00
ovary
eggs
4-cell,
1 hpf
1k-cell,
3 hpf
5 hpf
bud,
10 hpf
24 hpf

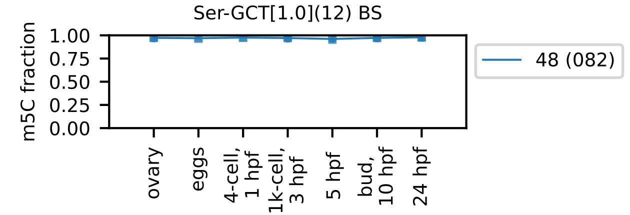

Ser-GCT[1.0](12) BS
m5C fraction
1.00
0.75
0.50
0.25
0.00
ovary
eggs
4-cell, 1 hpf
1k-cell, 3 hpf
5 hpf
bud, 10 hpf
24 hpf
48 (082)

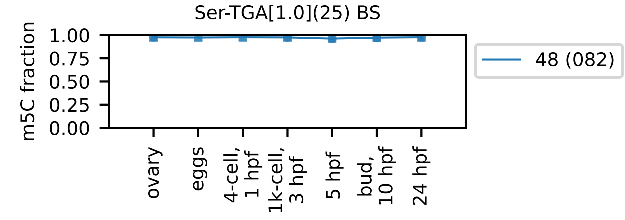

Ser-TGA[1.0](25) BS
m5C fraction
1.00
0.75
0.50
0.25
0.00
ovary
eggs
4-cell, 1 hpf
1k-cell, 3 hpf
5 hpf
bud, 10 hpf
24 hpf
48 (082)

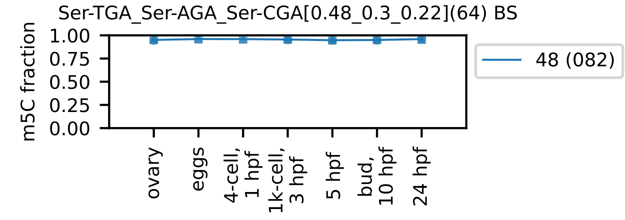
Ser-TGA_Ser-AGA_Ser-CGA[0.48_0.3_0.22](64) BS
m5C fraction
1.00
0.75
0.50
0.25
0.00
ovary
eggs
4-cell, 1 hpf
1k-cell, 3 hpf
5 hpf
bud, 10 hpf
24 hpf
48 (082)

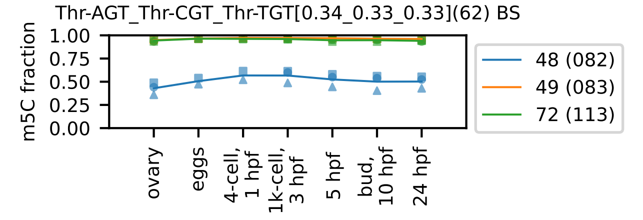
Thr-AGT_Thr-CGT_Thr-TGT[0.34_0.33_0.33](62) BS
m5C fraction
1.00
0.75
0.50
0.25
0.00
ovary
eggs
4-cell, 1 hpf
1k-cell, 3 hpf
5 hpf
bud, 10 hpf
24 hpf
48 (082)
49 (083)
72 (113)

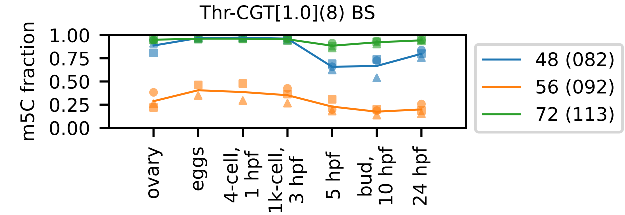

Thr-CGT[1.0](8) BS
m5C fraction
1.00
0.75
0.50
0.25
0.00
ovary
eggs
4-cell, 1 hpf
1k-cell, 3 hpf
5 hpf
bud, 10 hpf
24 hpf
48 (082)
56 (092)
72 (113)

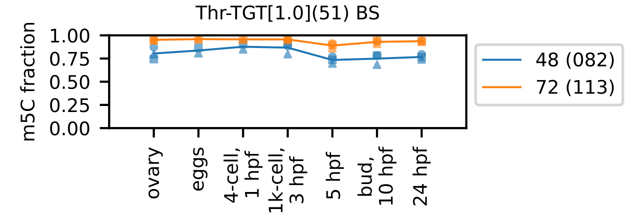
Thr-TGT[1.0](51) BS
m5C fraction
1.00
0.75
0.50
0.25
0.00
ovary
eggs
4-cell, 1 hpf
1k-cell, 3 hpf
5 hpf
bud, 10 hpf
24 hpf
48 (082)
72 (113)

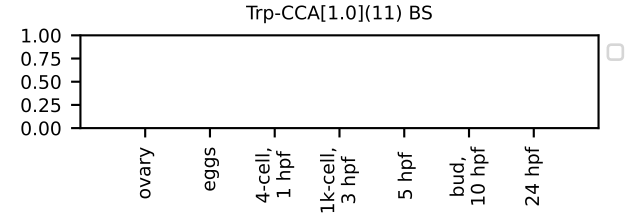
Trp-CCA[1.0](11) BS
1.00
0.75
0.50
0.25
0.00
ovary
eggs
4-cell, 1 hpf
1k-cell, 3 hpf
5 hpf
bud, 10 hpf
24 hpf

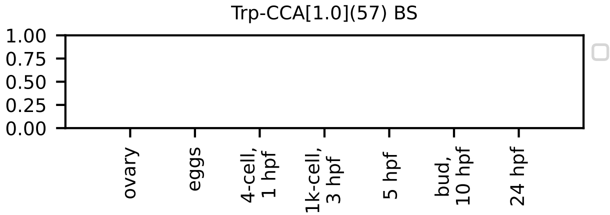
Trp-CCA[1.0](57) BS
1.00
0.75
0.50
0.25
0.00
ovary
eggs
4-cell, 1 hpf
1k-cell, 3 hpf
5 hpf
bud, 10 hpf
24 hpf

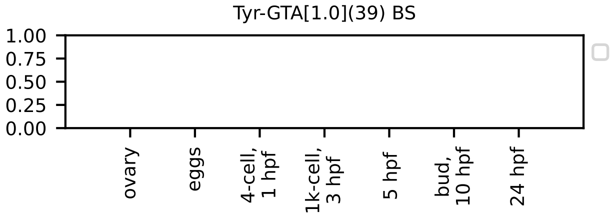
Tyr-GTA[1.0](39) BS
1.00
0.75
0.50
0.25
0.00
ovary
eggs
4-cell,
1 hpf
1k-cell,
3 hpf
5 hpf
bud,
10 hpf
24 hpf

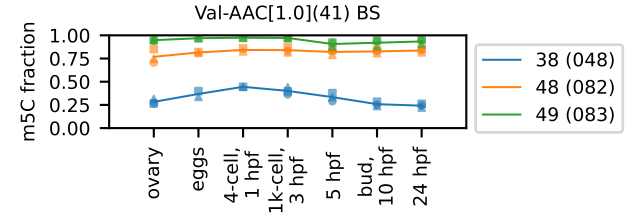
Val-AAC[1.0](41) BS
m5C fraction
1.00
0.75
0.50
0.25
0.00
ovary
eggs
4-cell, 1 hpf
1k-cell, 3 hpf
5 hpf
bud, 10 hpf
24 hpf
38 (048)
48 (082)
49 (083)

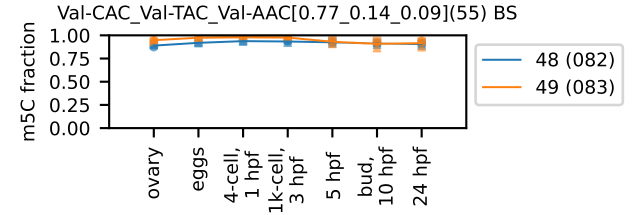

Val-CAC_Val-TAC_Val-AAC[0.77_0.14_0.09](55) BS
m5C fraction
1.00
0.75
0.50
0.25
0.00
ovary
eggs
4-cell, 1 hpf
1k-cell, 3 hpf
5 hpf
bud, 10 hpf
24 hpf
48 (082)
49 (083)

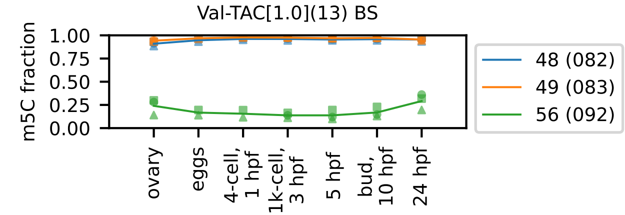

Val-TAC[1.0](13) BS
m5C fraction
1.00
0.75
0.50
0.25
0.00
ovary
eggs
4-cell, 1 hpf
1k-cell, 3 hpf
5 hpf
bud, 10 hpf
24 hpf
48 (082)
49 (083)
56 (092)

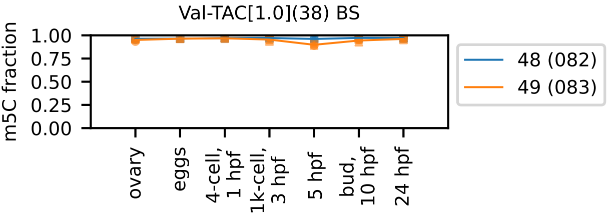
Val-TAC[1.0](38) BS
m5C fraction
1.00
0.75
0.50
0.25
0.00
ovary
eggs
4-cell, 1 hpf
1k-cell, 3 hpf
5 hpf
bud, 10 hpf
24 hpf
48 (082)
49 (083)

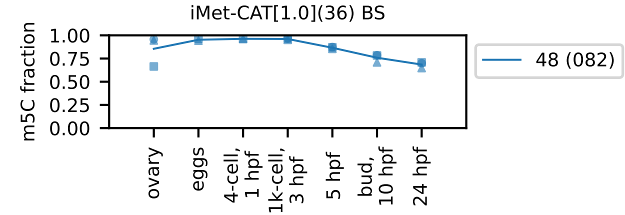
iMet-CAT[1.0](36) BS
m5C fraction
1.00
0.75
0.50
0.25
0.00
ovary
eggs
4-cell, 1 hpf
1k-cell, 3 hpf
5 hpf
bud, 10 hpf
24 hpf
48 (082)

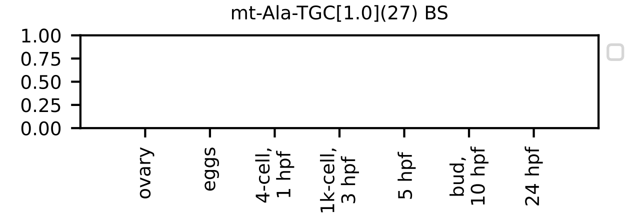
mt-Ala-TGC[1.0](27) BS
1.00
0.75
0.50
0.25
0.00
ovary
eggs
4-cell, 1 hpf
1k-cell, 3 hpf
5 hpf
bud, 10 hpf
24 hpf

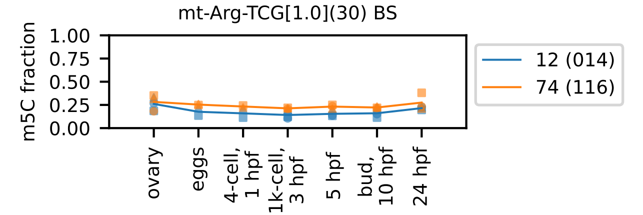

mt-Arg-TCG[1.0](30) BS
m5C fraction
1.00
0.75
0.50
0.25
0.00
ovary
eggs
4-cell, 1 hpf
1k-cell, 3 hpf
5 hpf
bud, 10 hpf
24 hpf
12 (014)
74 (116)

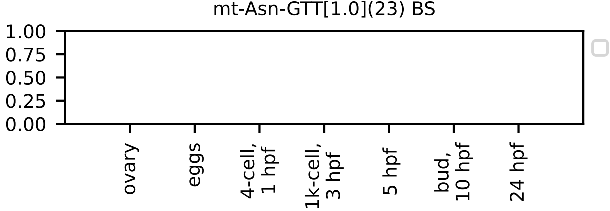

mt-Asn-GTT[1.0](23) BS
1.00
0.75
0.50
0.25
0.00
ovary
eggs
4-cell, 1 hpf
1k-cell, 3 hpf
5 hpf
bud, 10 hpf
24 hpf

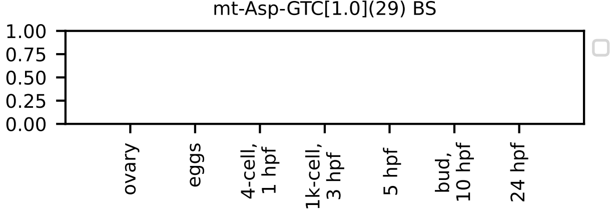
mt-Asp-GTC[1.0](29) BS
1.00
0.75
0.50
0.25
0.00
ovary
eggs
4-cell,
1 hpf
1k-cell,
3 hpf
5 hpf
bud,
10 hpf
24 hpf

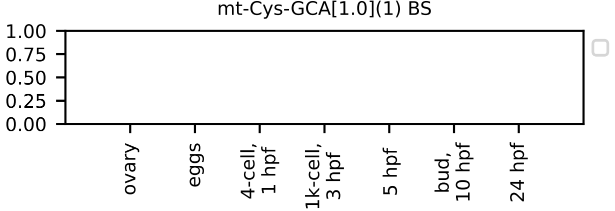
mt-Cys-GCA[1.0](1) BS
1.00
0.75
0.50
0.25
0.00
ovary
eggs
4-cell, 1 hpf
1k-cell, 3 hpf
5 hpf
bud, 10 hpf
24 hpf

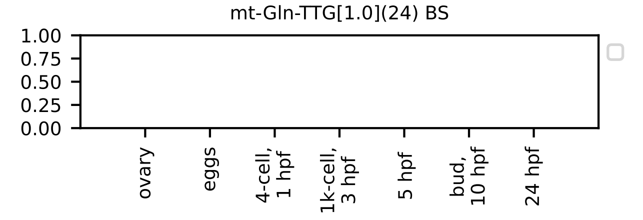

mt-Gln-TTG[1.0](24) BS
1.00
0.75
0.50
0.25
0.00
ovary
eggs
4-cell, 1 hpf
1k-cell, 3 hpf
5 hpf
bud, 10 hpf
24 hpf

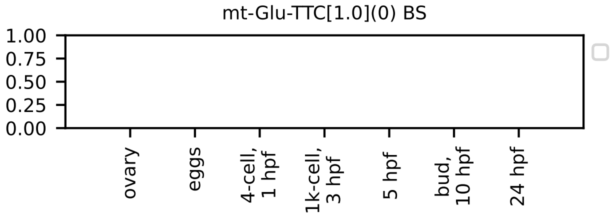
mt-Glu-TTC[1.0](0) BS
1.00
0.75
0.50
0.25
0.00
ovary
eggs
4-cell, 1 hpf
1k-cell, 3 hpf
5 hpf
bud, 10 hpf
24 hpf

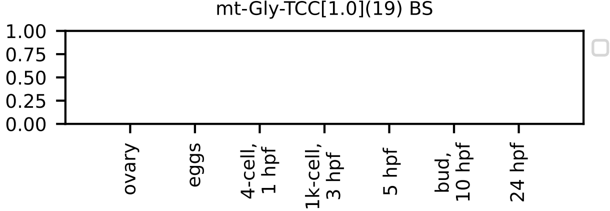
mt-Gly-TCC[1.0](19) BS
1.00
0.75
0.50
0.25
0.00
ovary
eggs
4-cell, 1 hpf
1k-cell, 3 hpf
5 hpf
bud, 10 hpf
24 hpf

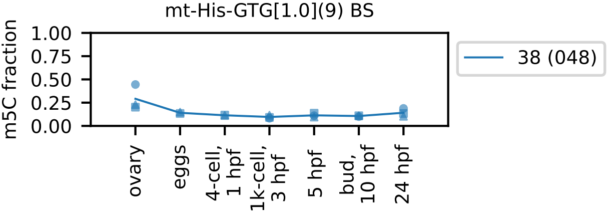

mt-His-GTG[1.0](9) BS
m5C fraction
1.00
0.75
0.50
0.25
0.00
ovary
eggs
4-cell, 1 hpf
1k-cell, 3 hpf
5 hpf
bud, 10 hpf
24 hpf
38 (048)

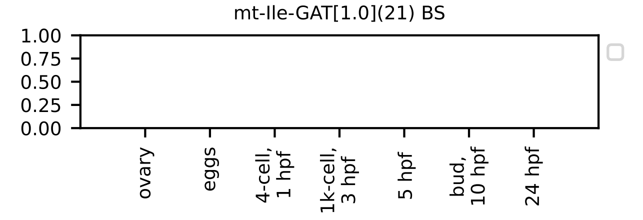
mt-Ile-GAT[1.0](21) BS
1.00
0.75
0.50
0.25
0.00
ovary
eggs
4-cell, 1 hpf
1k-cell, 3 hpf
5 hpf
bud, 10 hpf
24 hpf

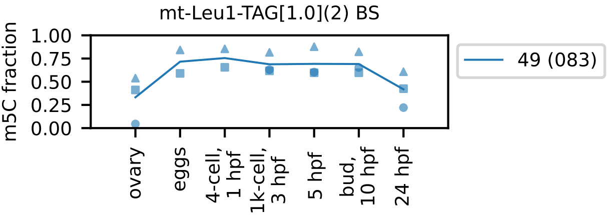
mt-Leu1-TAG[1.0](2) BS
m5C fraction
1.00
0.75
0.50
0.25
0.00
ovary
eggs
4-cell, 1 hpf
1k-cell, 3 hpf
5 hpf
bud, 10 hpf
24 hpf
49 (083)

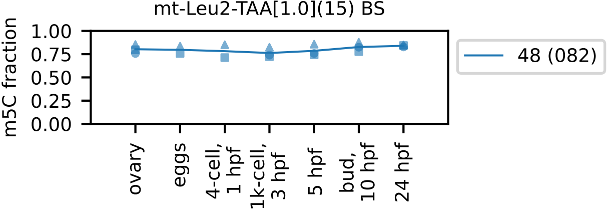
mt-Leu2-TAA[1.0](15) BS
m5C fraction
1.00
0.75
0.50
0.25
0.00
ovary
eggs
4-cell, 1 hpf
1k-cell, 3 hpf
5 hpf
bud, 10 hpf
24 hpf
48 (082)

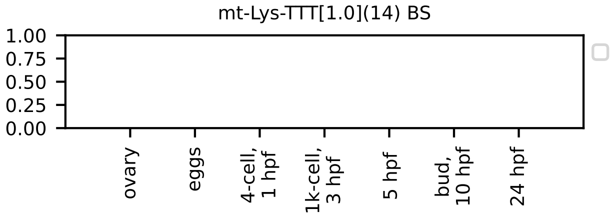
mt-Lys-TTT[1.0](14) BS
1.00
0.75
0.50
0.25
0.00
ovary
eggs
4-cell, 1 hpf
1k-cell, 3 hpf
5 hpf
bud, 10 hpf
24 hpf

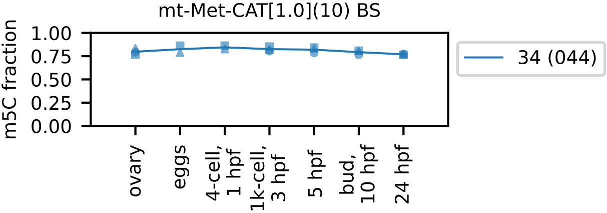
mt-Met-CAT[1.0](10) BS
m5C fraction
1.00
0.75
0.50
0.25
0.00
ovary
eggs
4-cell, 1 hpf
1k-cell, 3 hpf
5 hpf
bud, 10 hpf
24 hpf
34 (044)

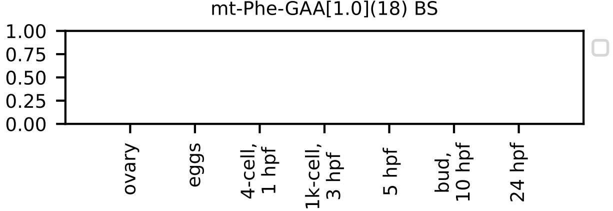

mt-Phe-GAA[1.0](18) BS
1.00
0.75
0.50
0.25
0.00
ovary
eggs
4-cell, 1 hpf
1k-cell, 3 hpf
5 hpf
bud, 10 hpf
24 hpf

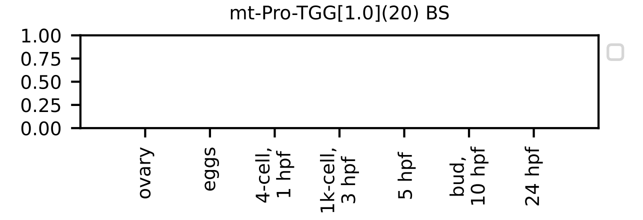

mt-Pro-TGG[1.0](20) BS
1.00
0.75
0.50
0.25
0.00
ovary
eggs
4-cell, 1 hpf
1k-cell, 3 hpf
5 hpf
bud, 10 hpf
24 hpf

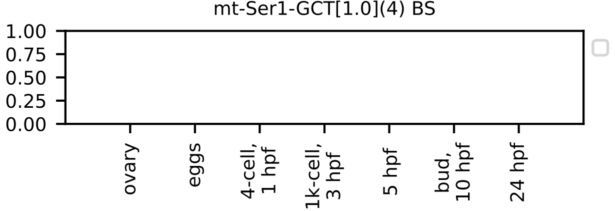
mt-Ser1-GCT[1.0](4) BS
1.00
0.75
0.50
0.25
0.00
ovary
eggs
4-cell, 1 hpf
1k-cell, 3 hpf
5 hpf
bud, 10 hpf
24 hpf

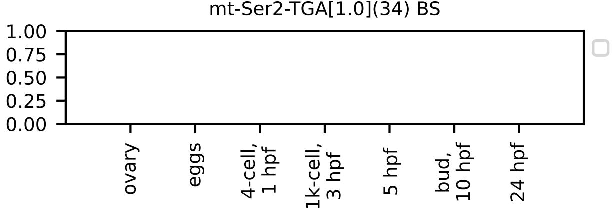
mt-Ser2-TGA[1.0](34) BS
1.00
0.75
0.50
0.25
0.00
ovary
eggs
4-cell, 1 hpf
1k-cell, 3 hpf
5 hpf
bud, 10 hpf
24 hpf

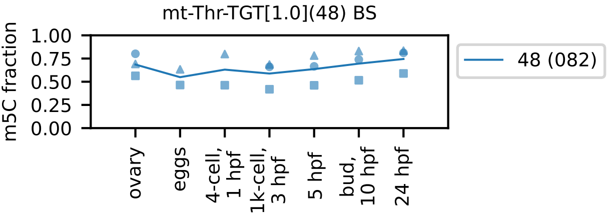

mt-Thr-TGT[1.0](48) BS
m5C fraction
1.00
0.75
0.50
0.25
0.00
ovary
eggs
4-cell, 1 hpf
1k-cell, 3 hpf
5 hpf
bud, 10 hpf
24 hpf
48 (082)

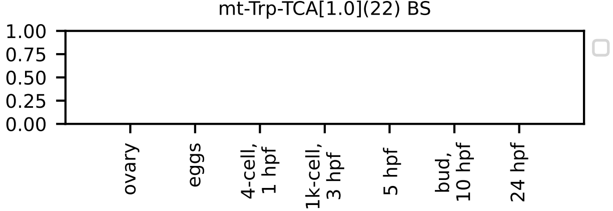
mt-Trp-TCA[1.0](22) BS
1.00
0.75
0.50
0.25
0.00
ovary
eggs
4-cell, 1 hpf
1k-cell, 3 hpf
5 hpf
bud, 10 hpf
24 hpf

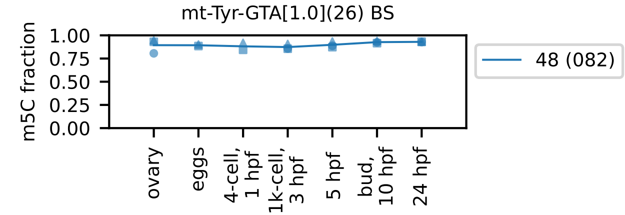

mt-Tyr-GTA[1.0](26) BS
m5C fraction
1.00
0.75
0.50
0.25
0.00
ovary
eggs
4-cell, 1 hpf
1k-cell, 3 hpf
5 hpf
bud, 10 hpf
24 hpf
48 (082)

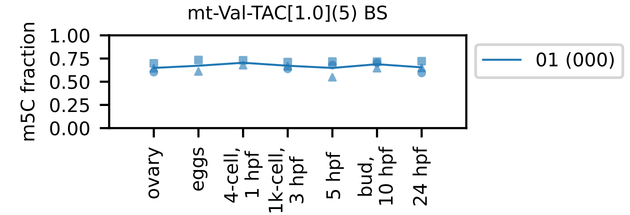
mt-Val-TAC[1.0](5) BS
m5C fraction
1.00
0.75
0.50
0.25
0.00
ovary
eggs
4-cell, 1 hpf
1k-cell, 3 hpf
5 hpf
bud, 10 hpf
24 hpf
01 (000)
