# Supplementary figures and images for: tRNA expression and modification landscapes, and their dynamics during zebrafish embryo development
Source: Nucleic Acids Res. 2024 Jul 11;52(17):10575–94. doi: 10.1093/nar/gkae595 (PMC11417395; doi:10.1093/nar/gkae595)

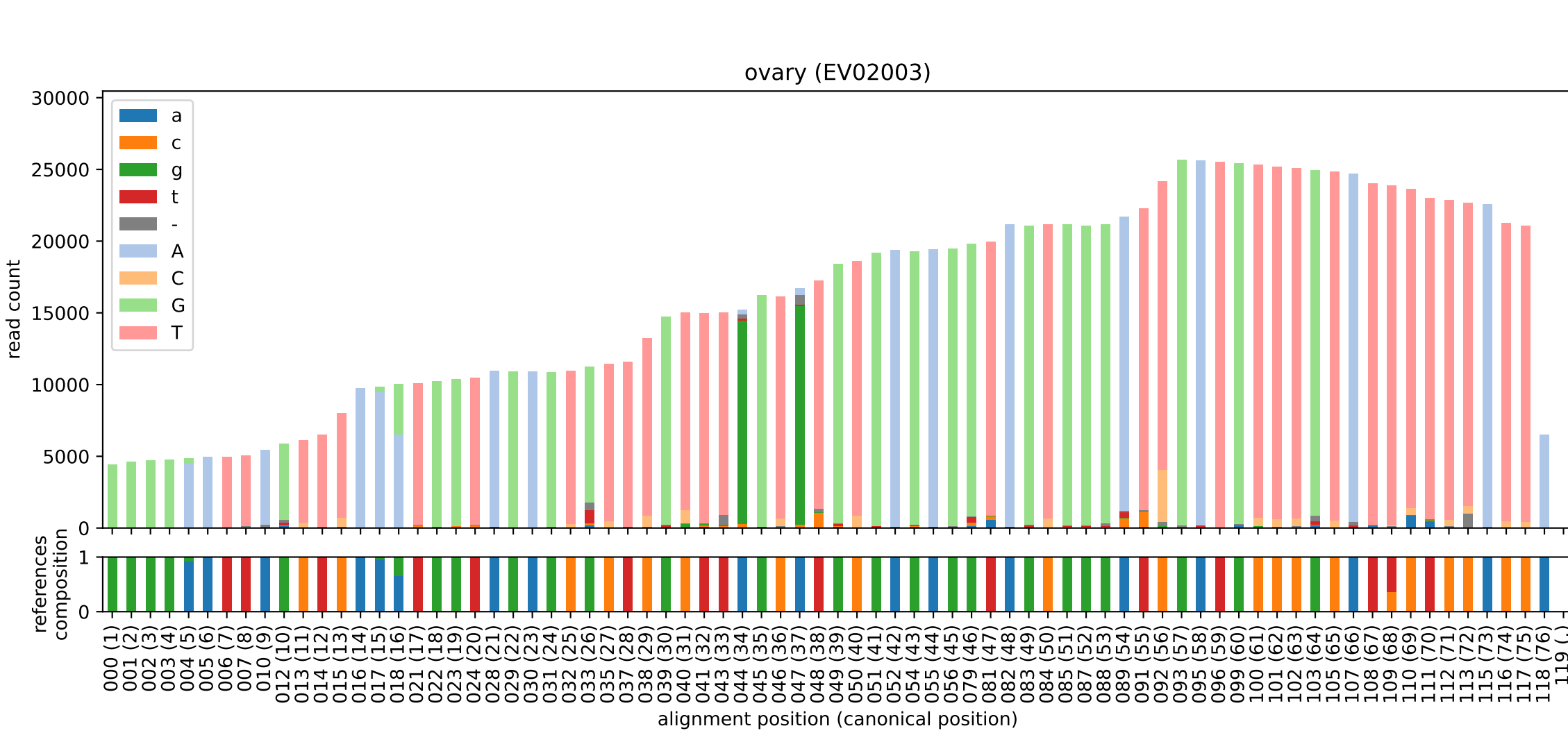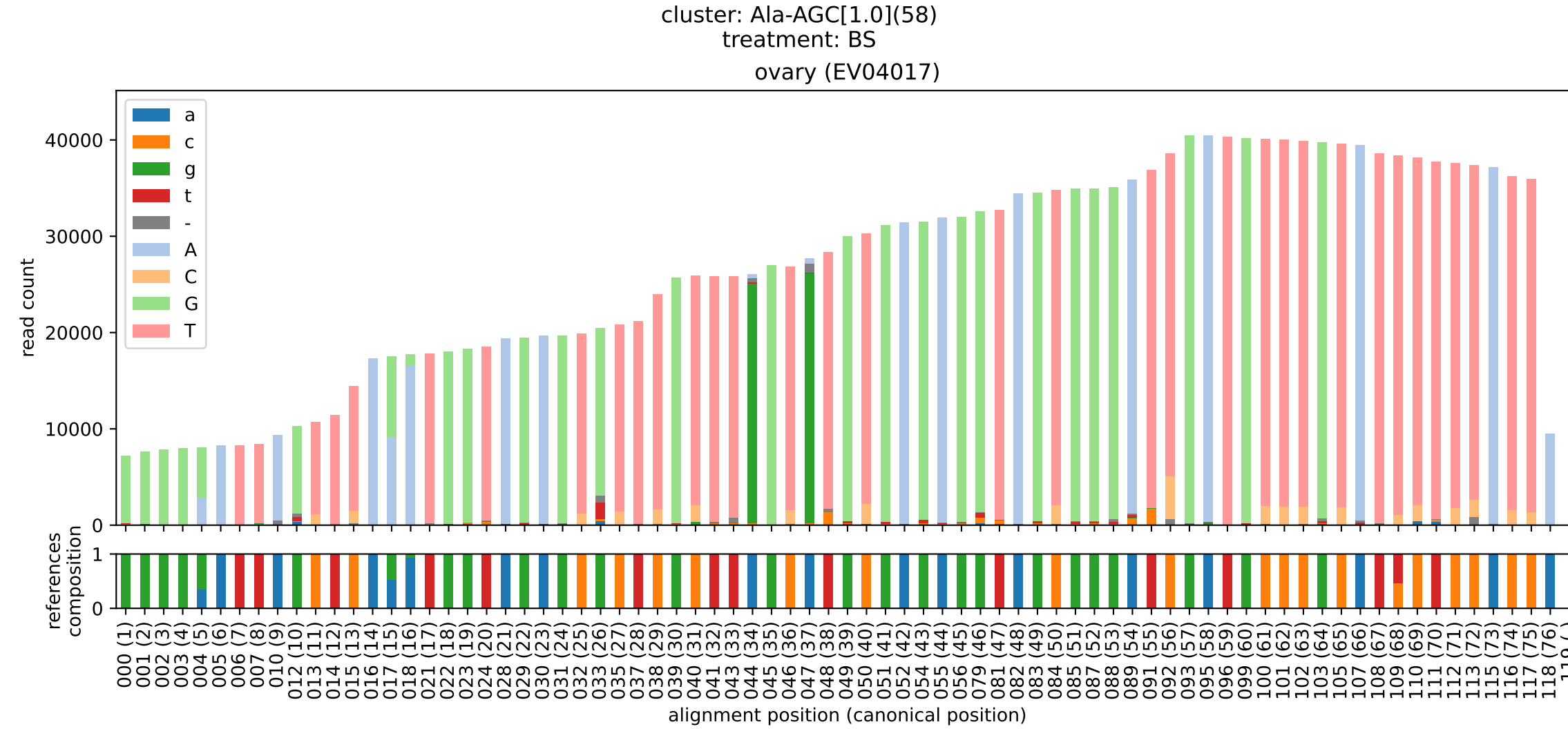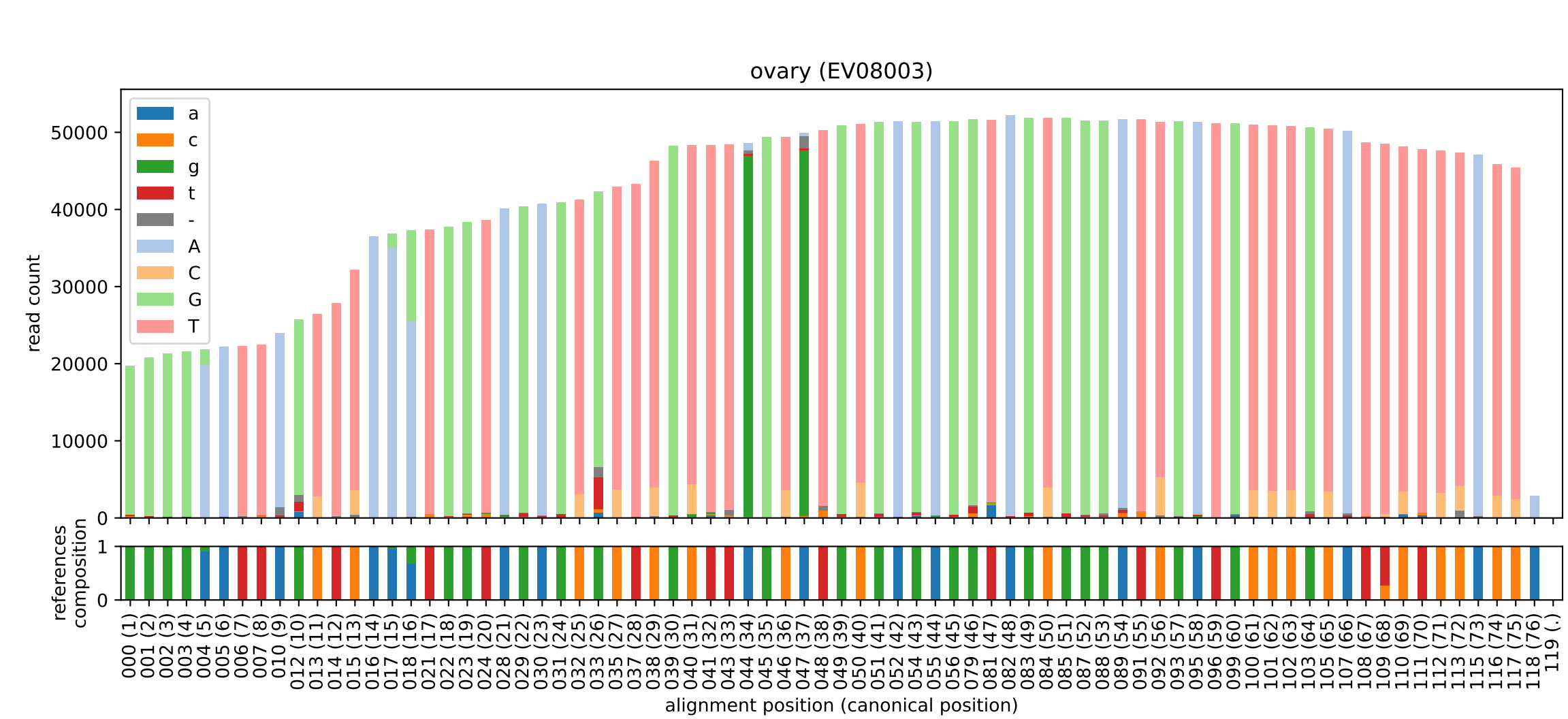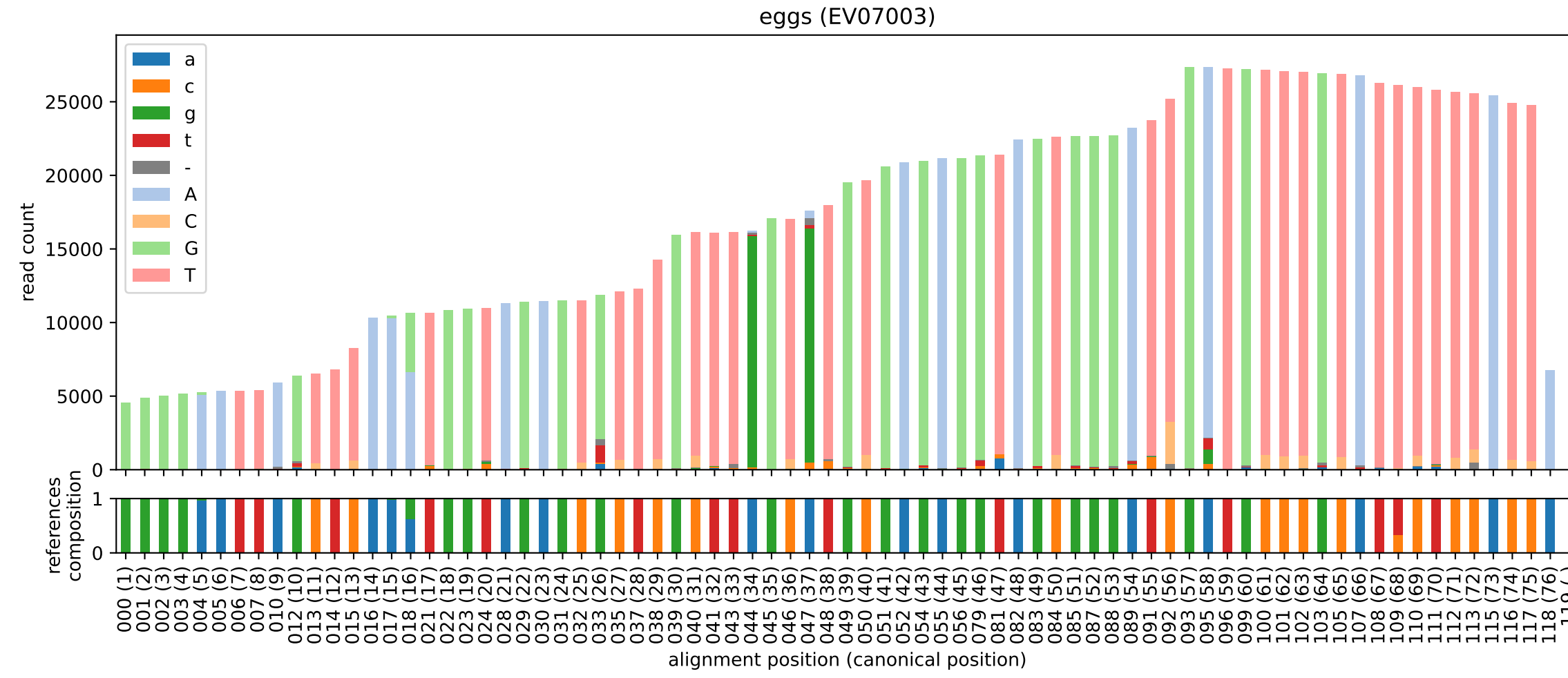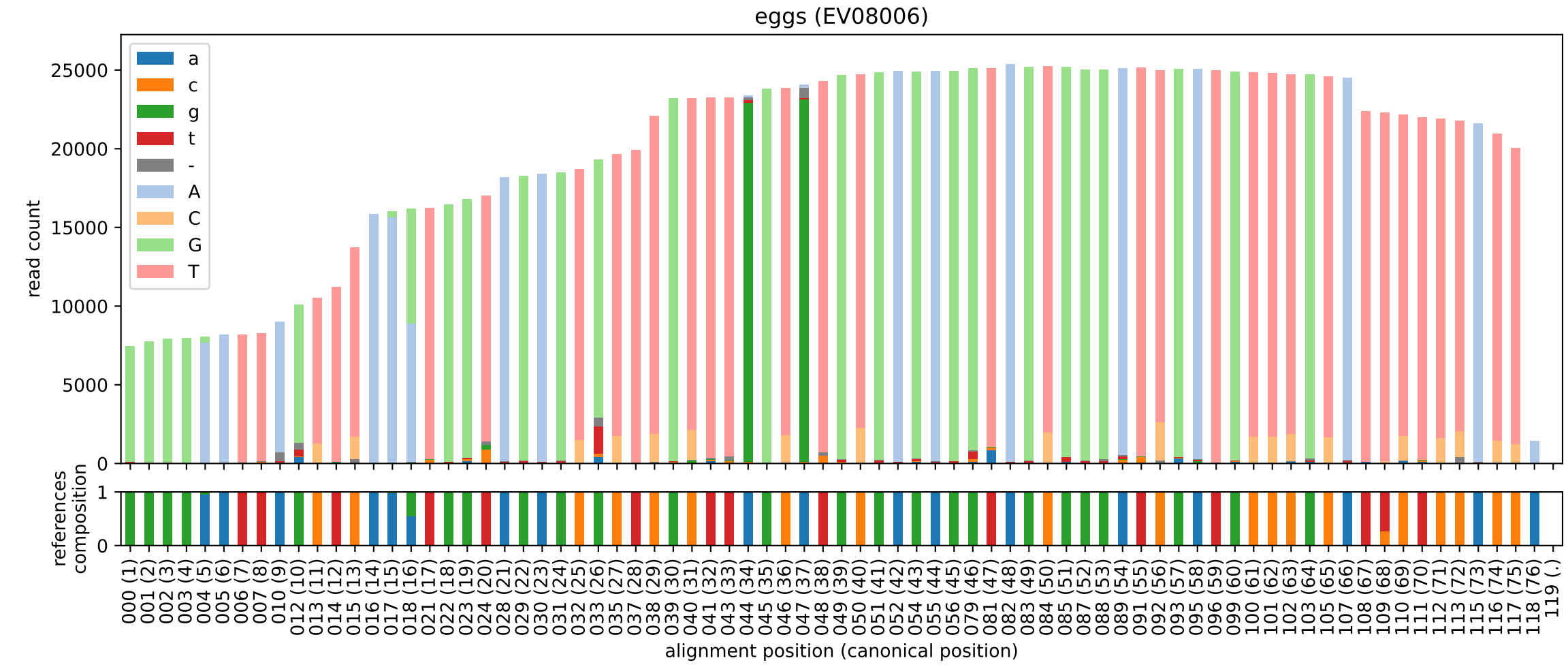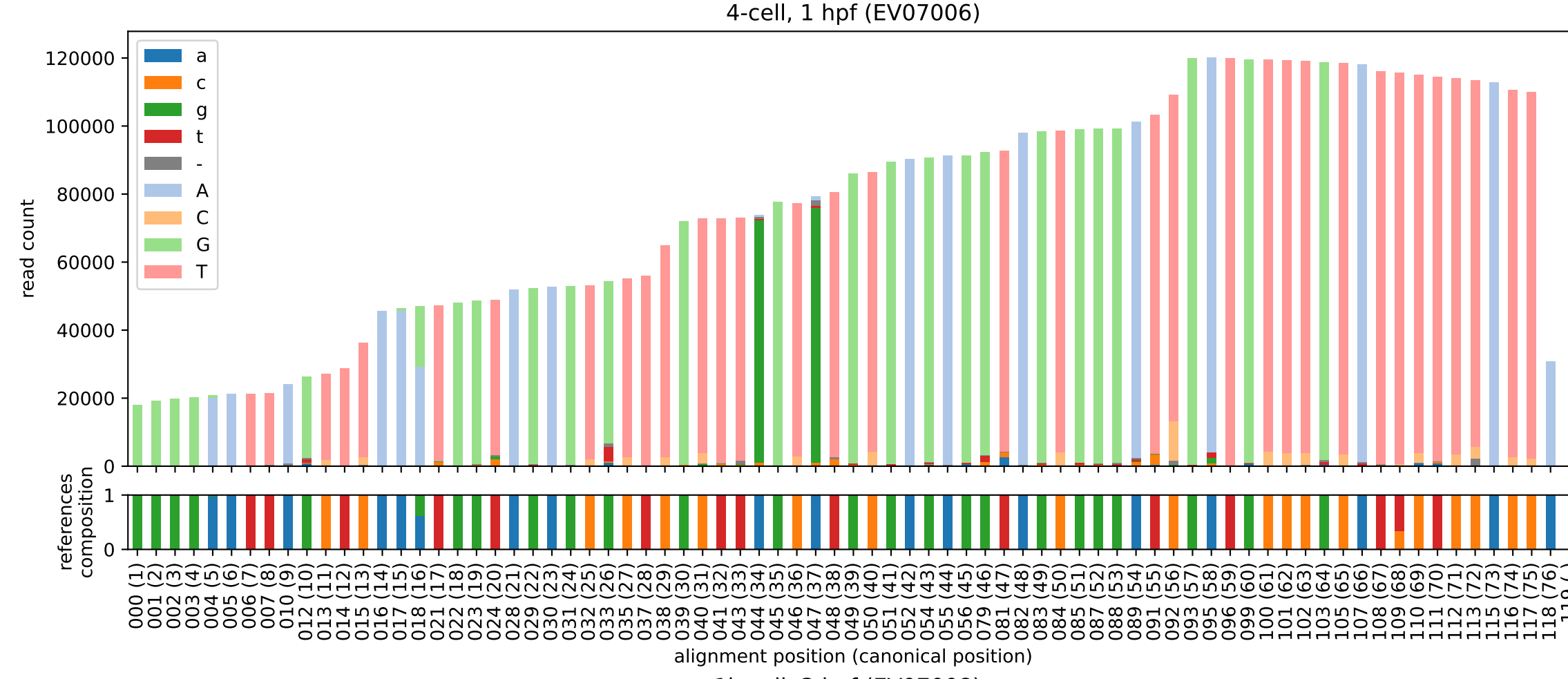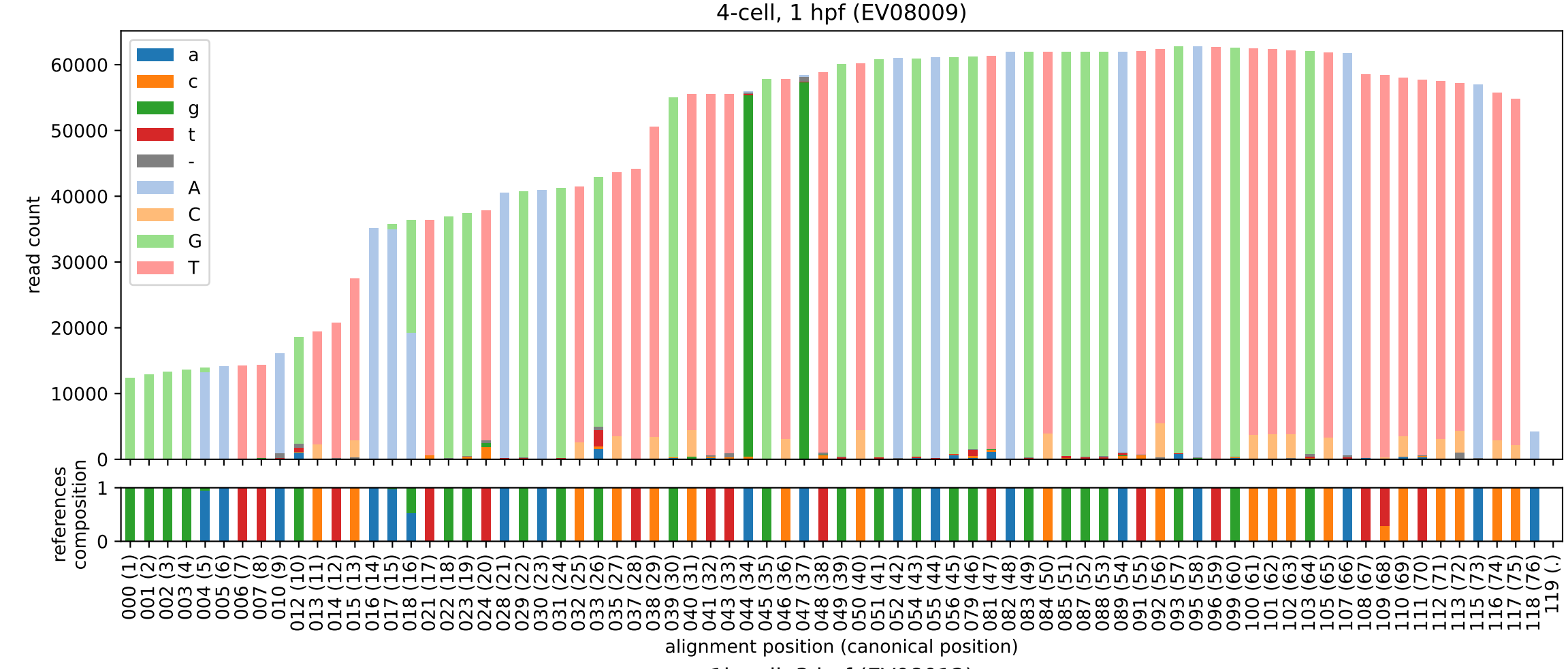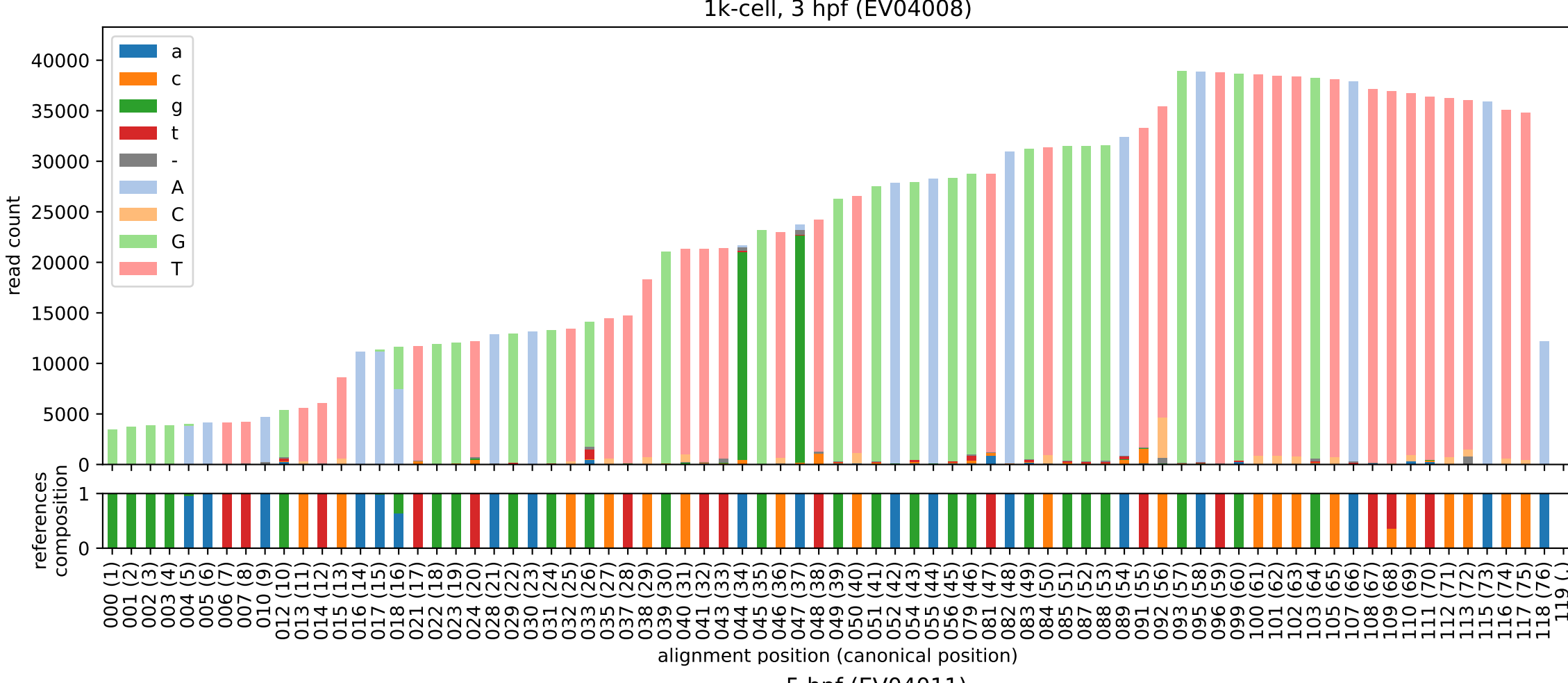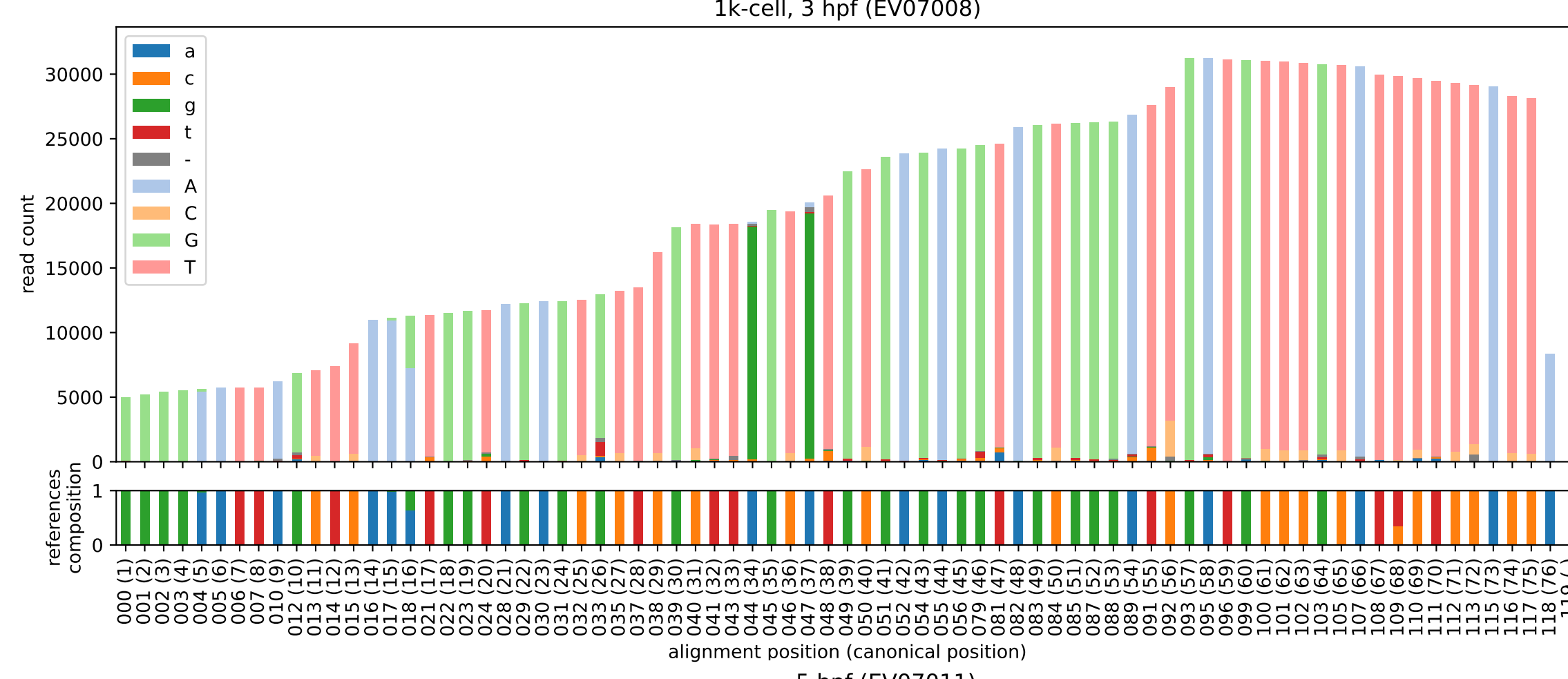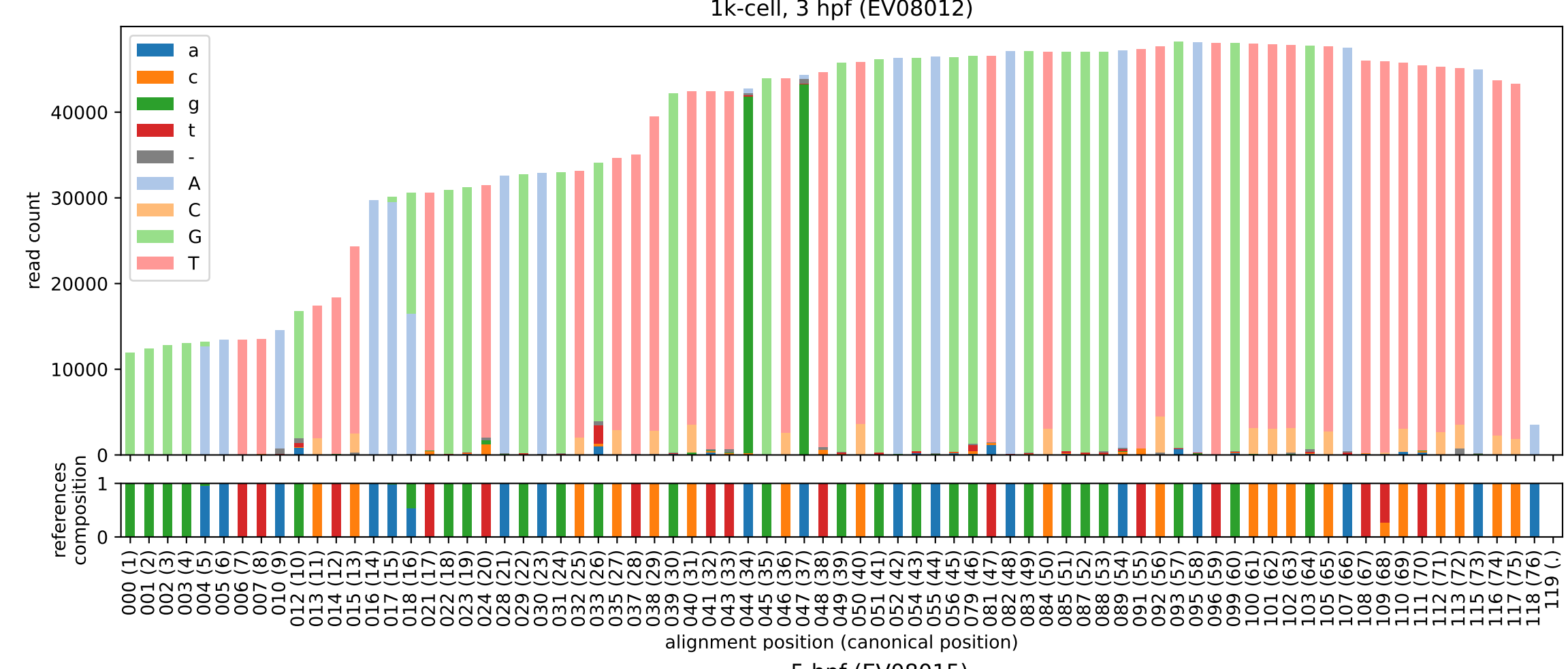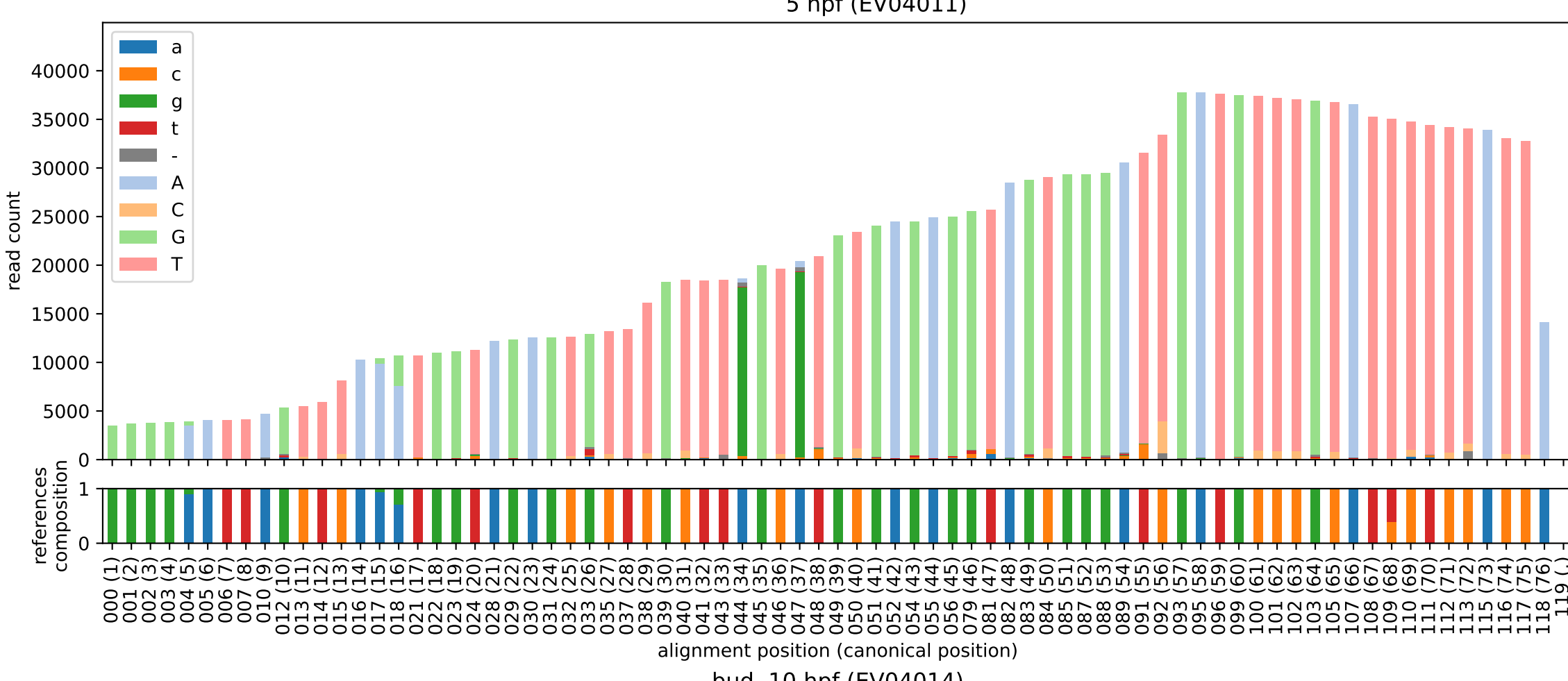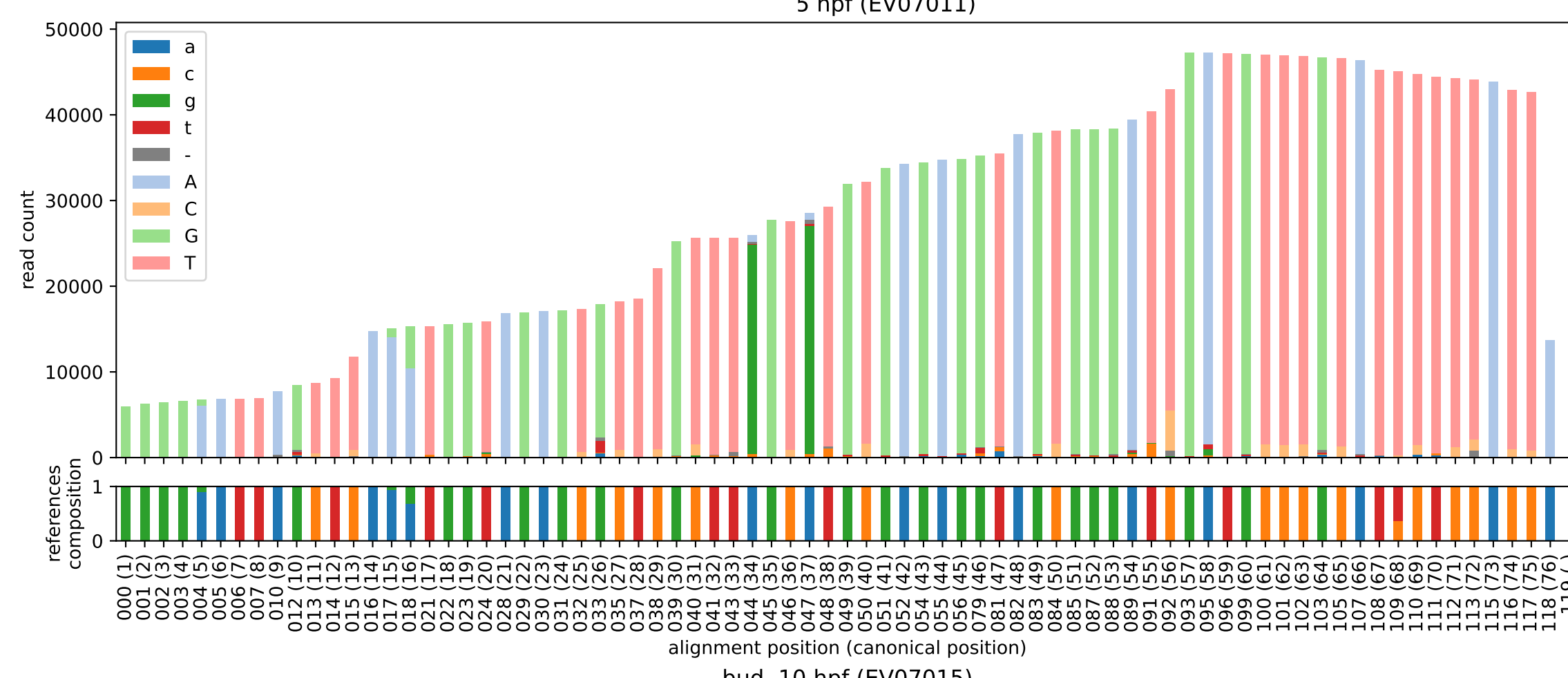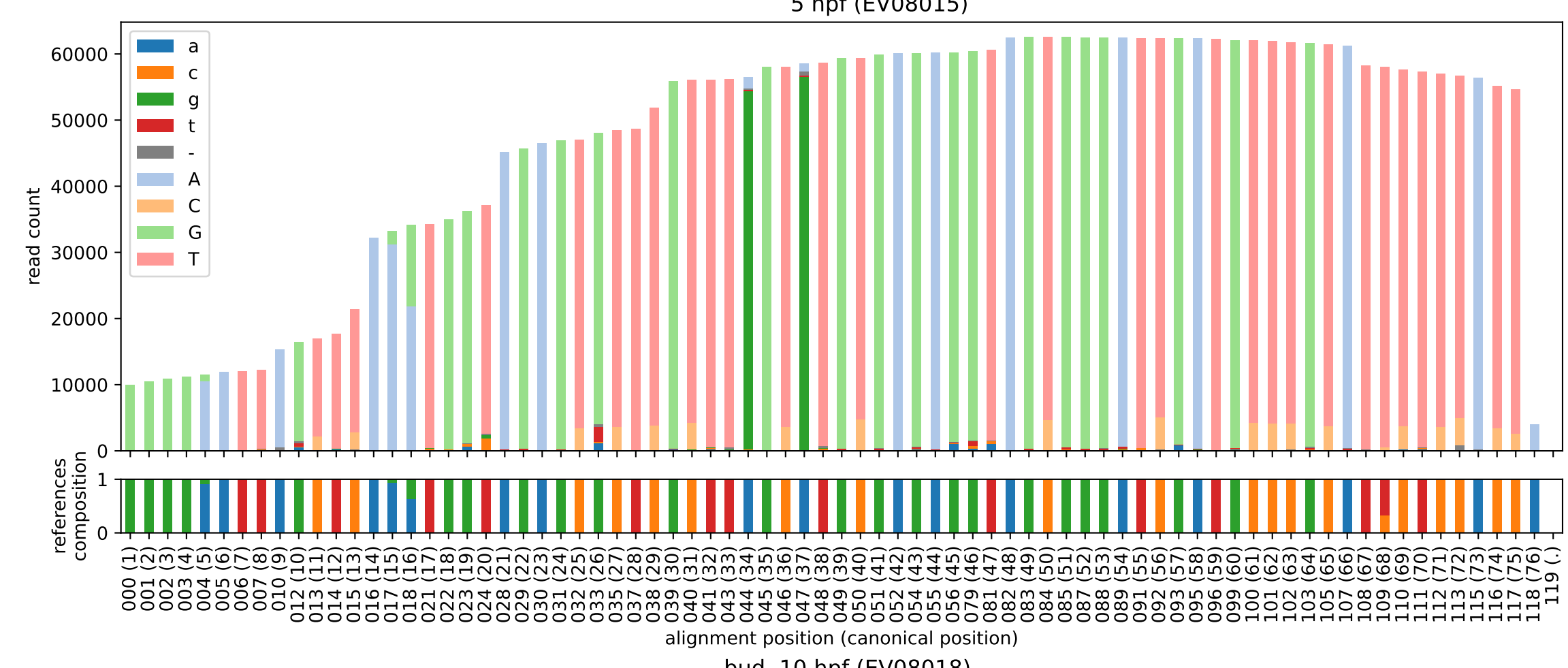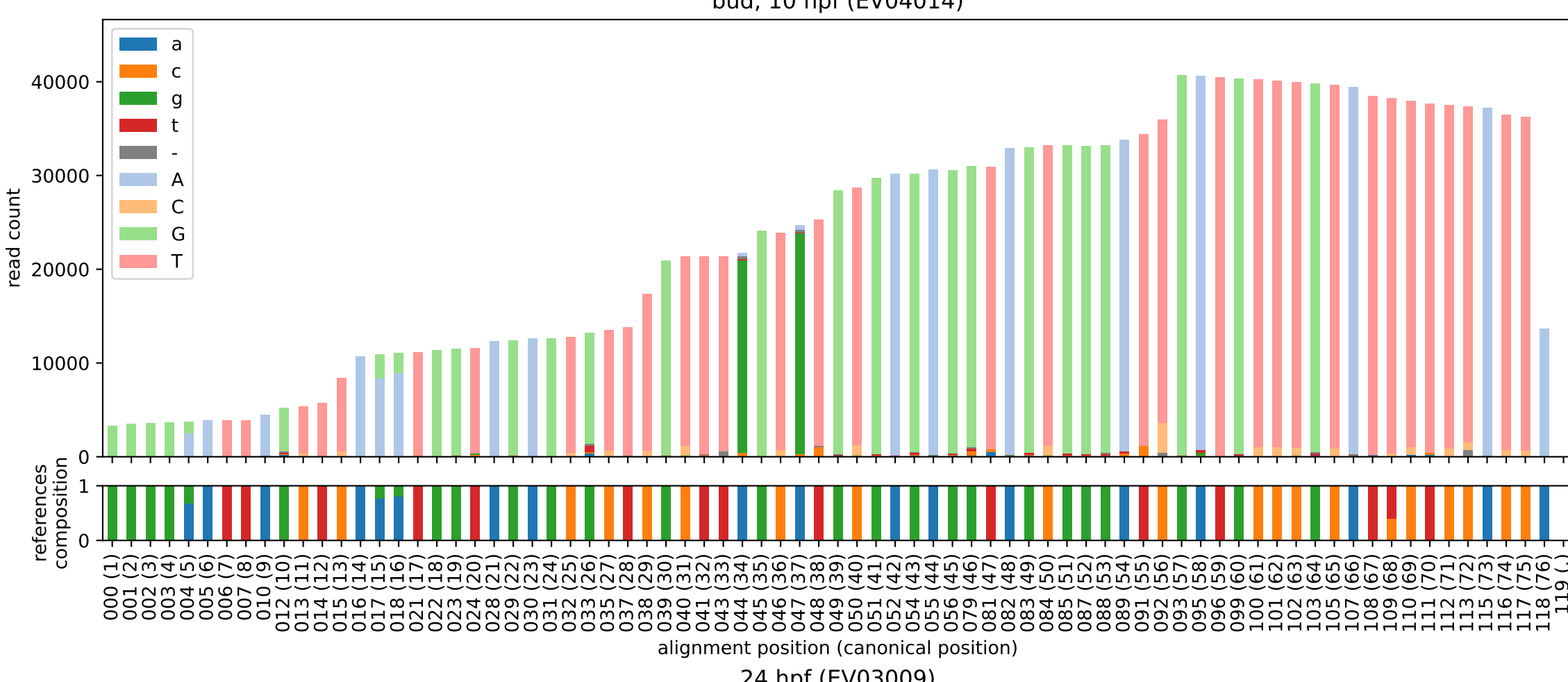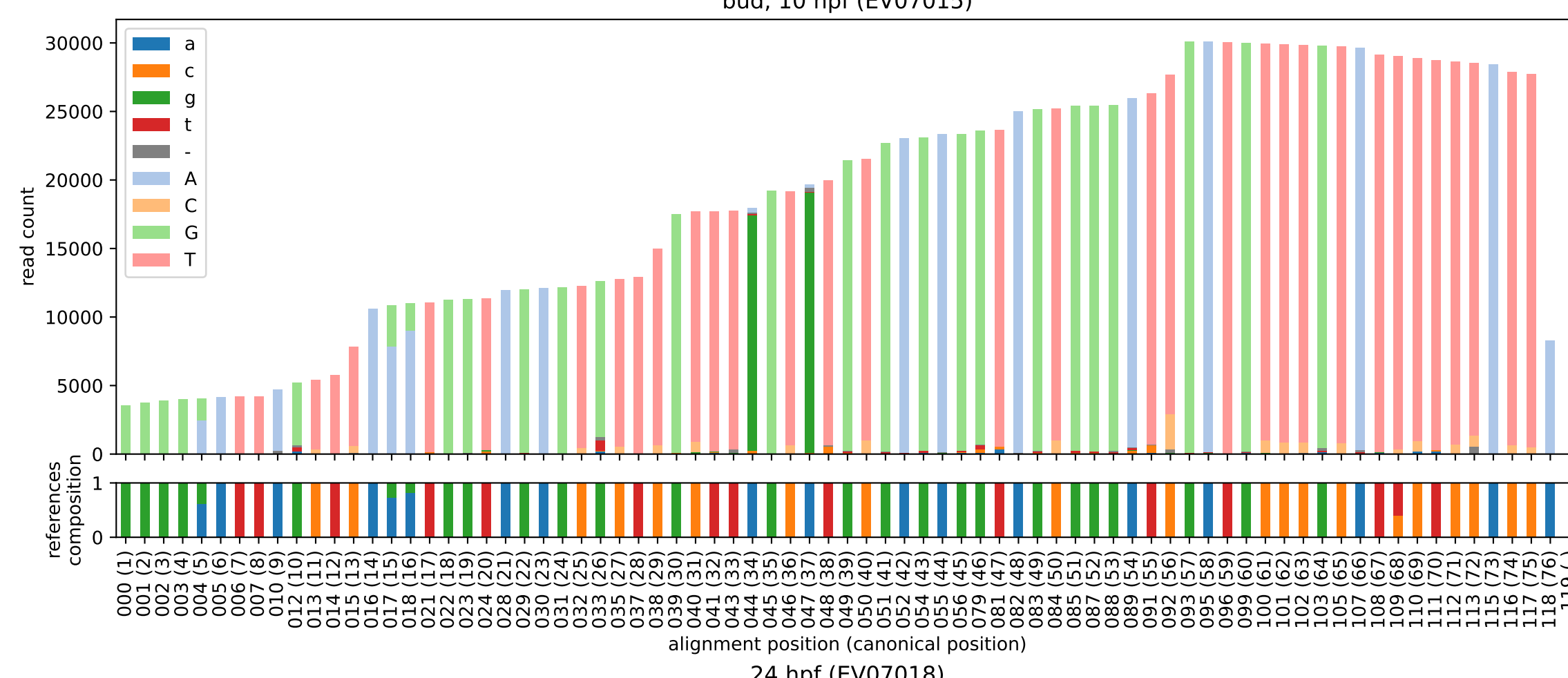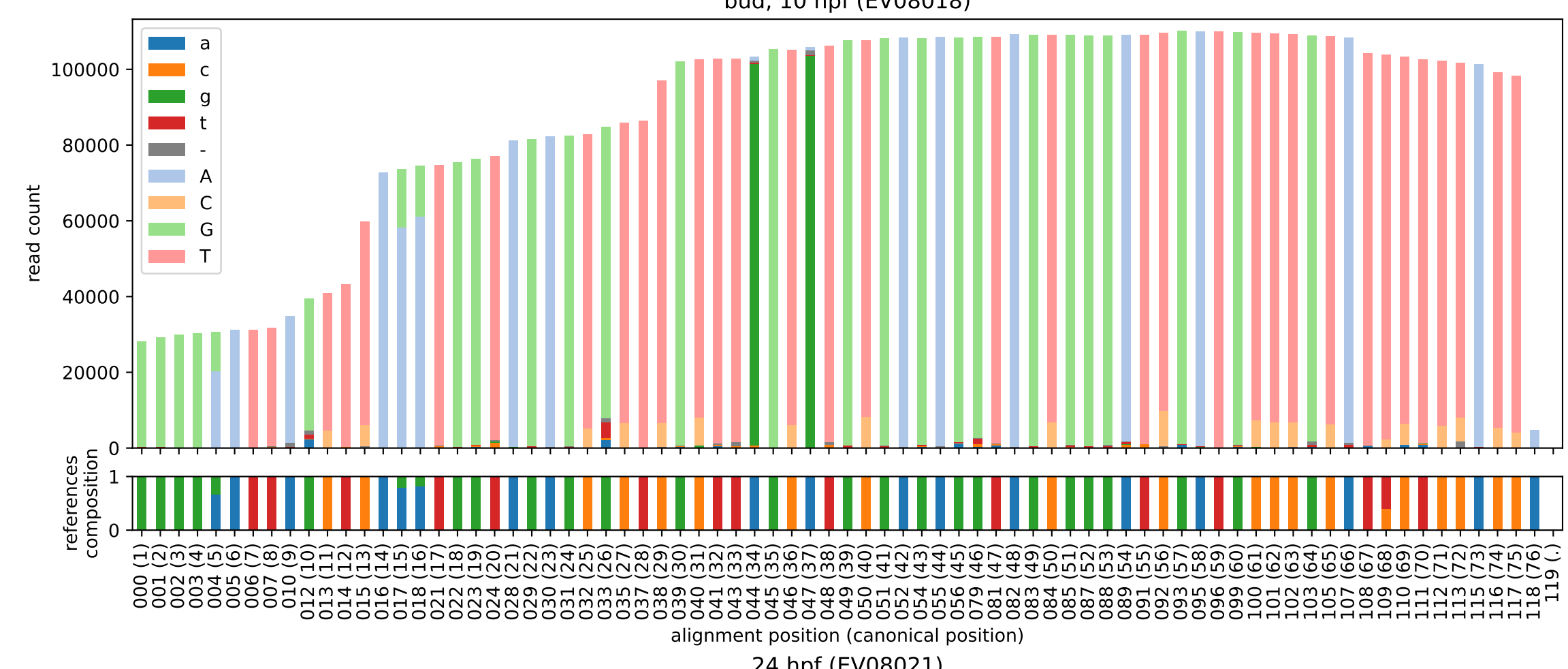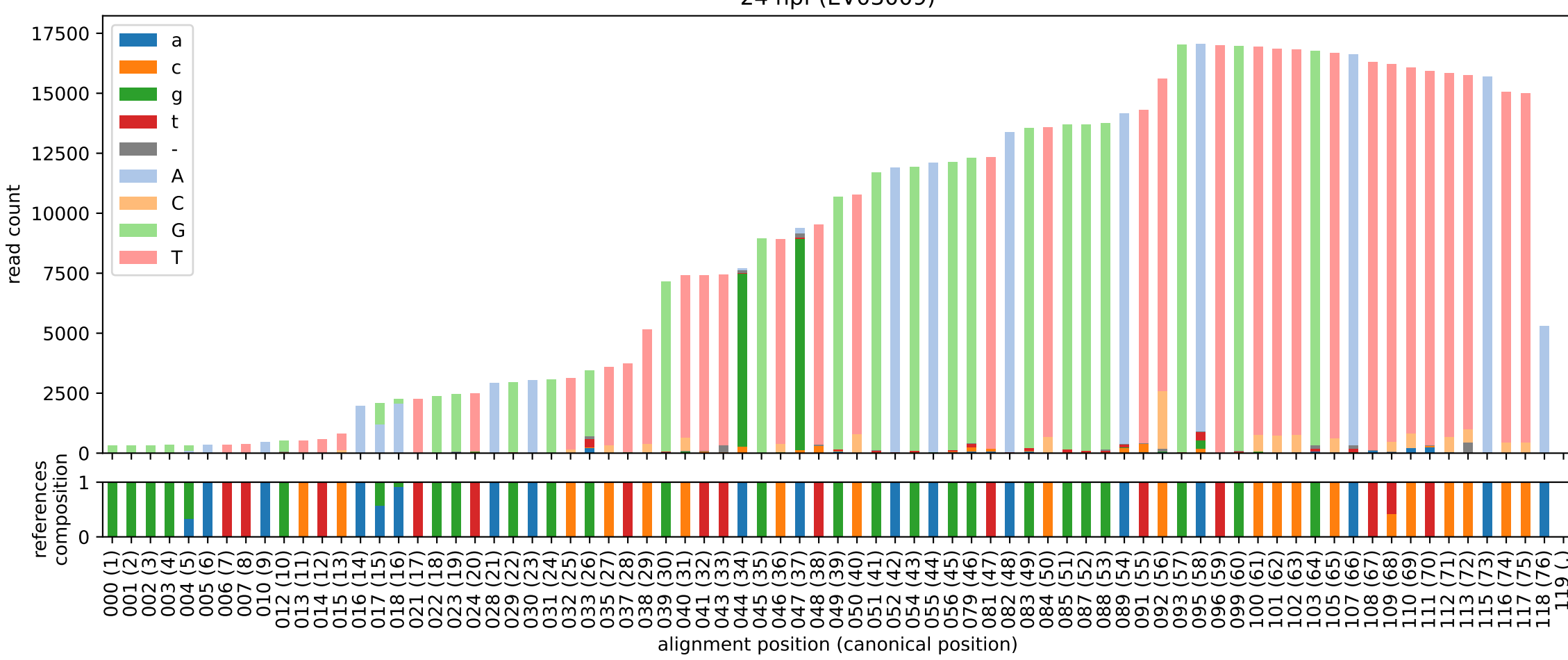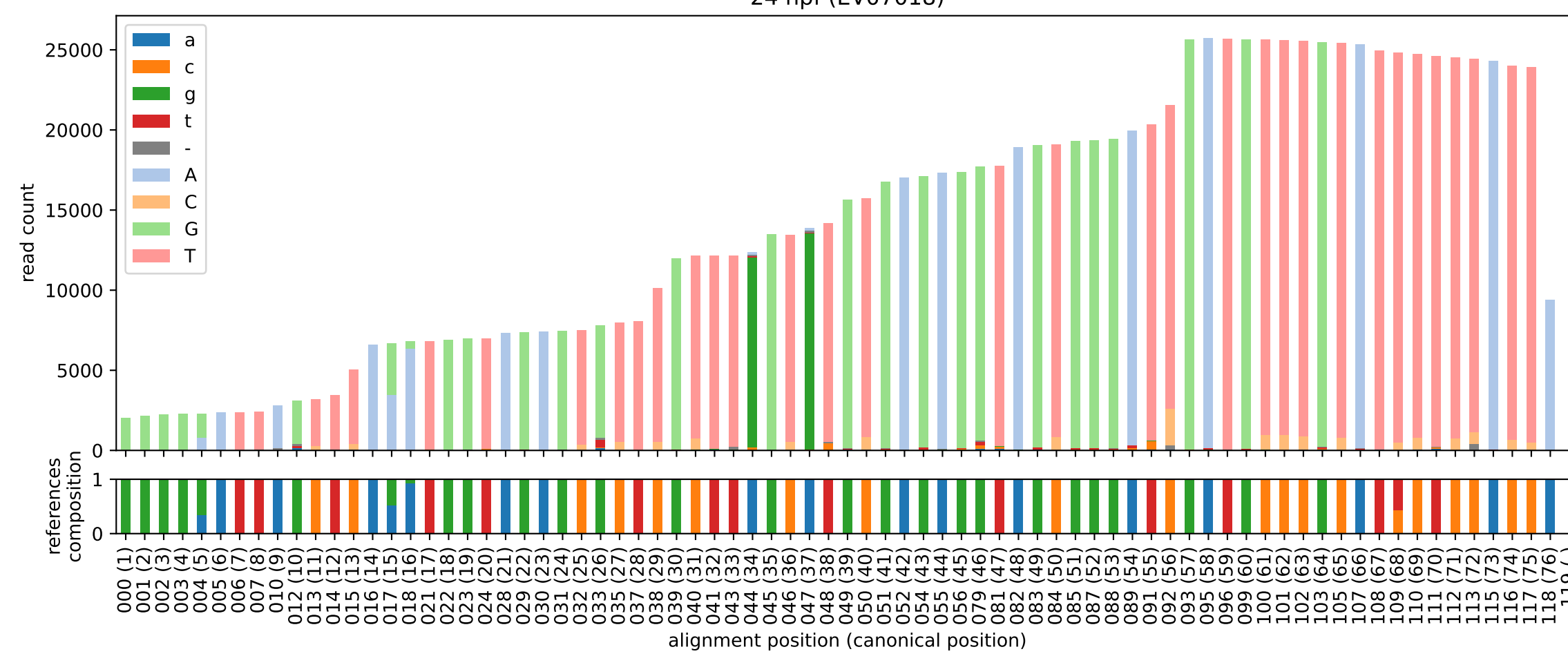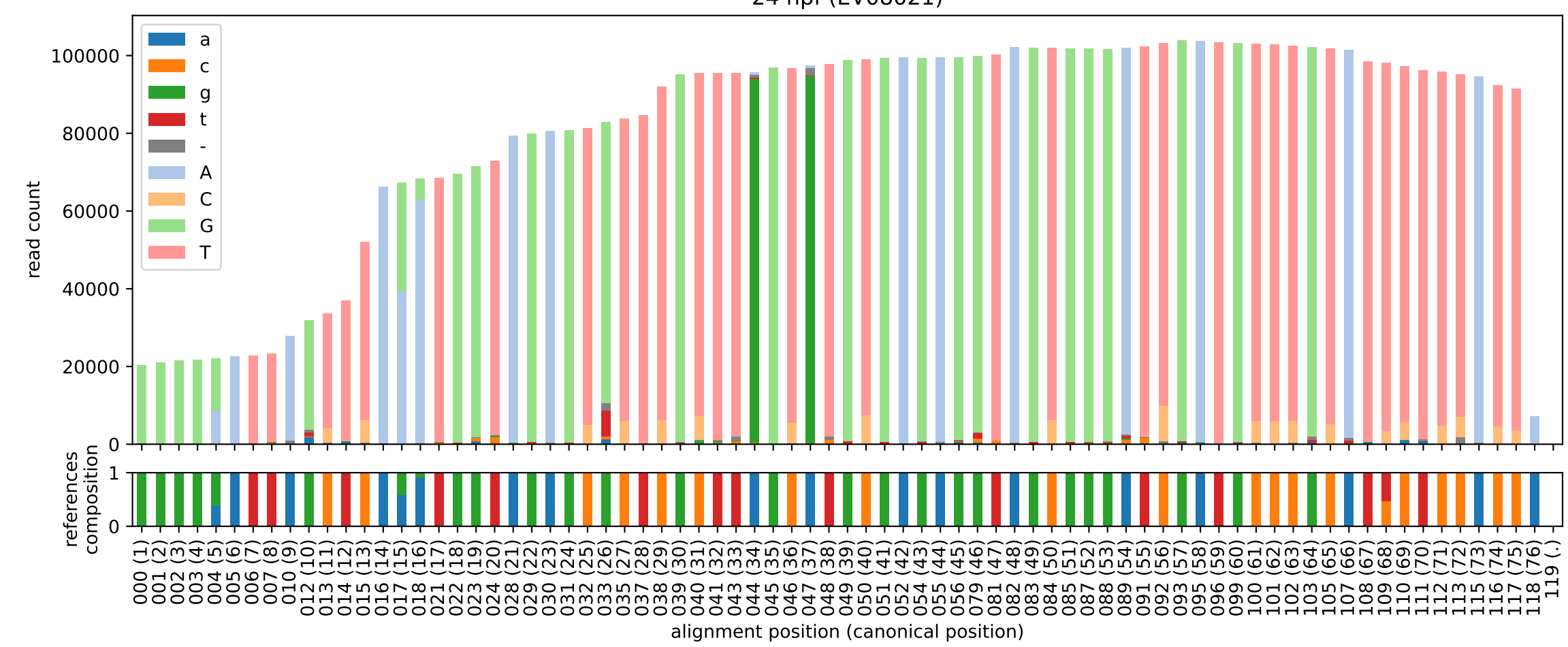



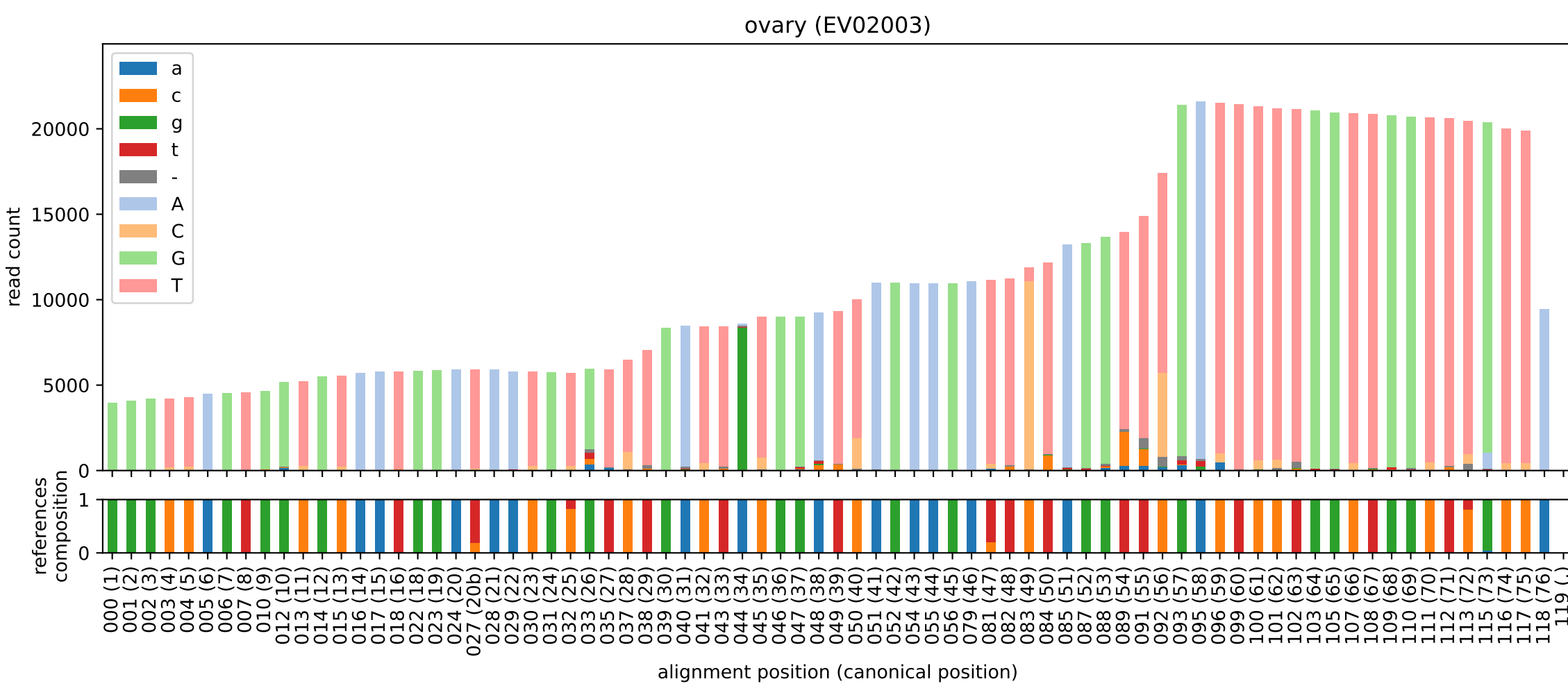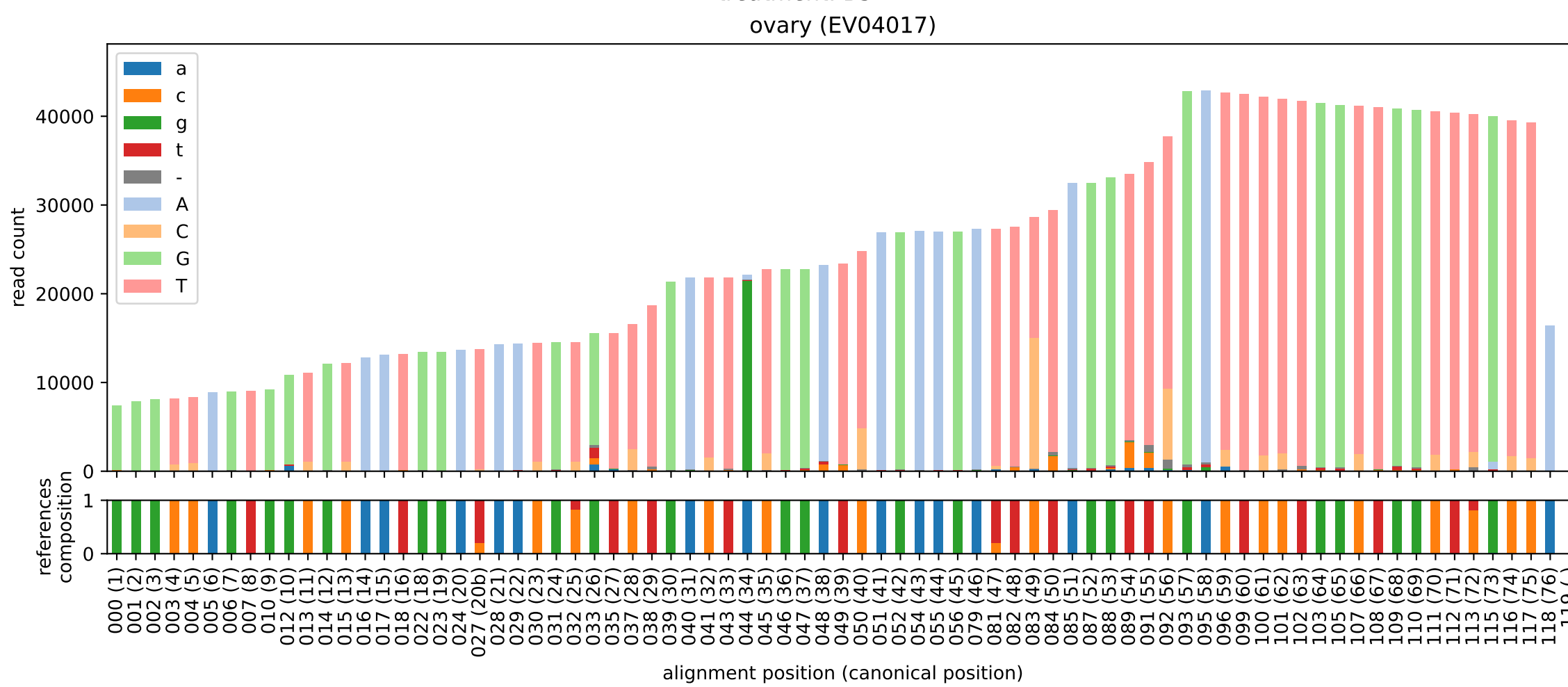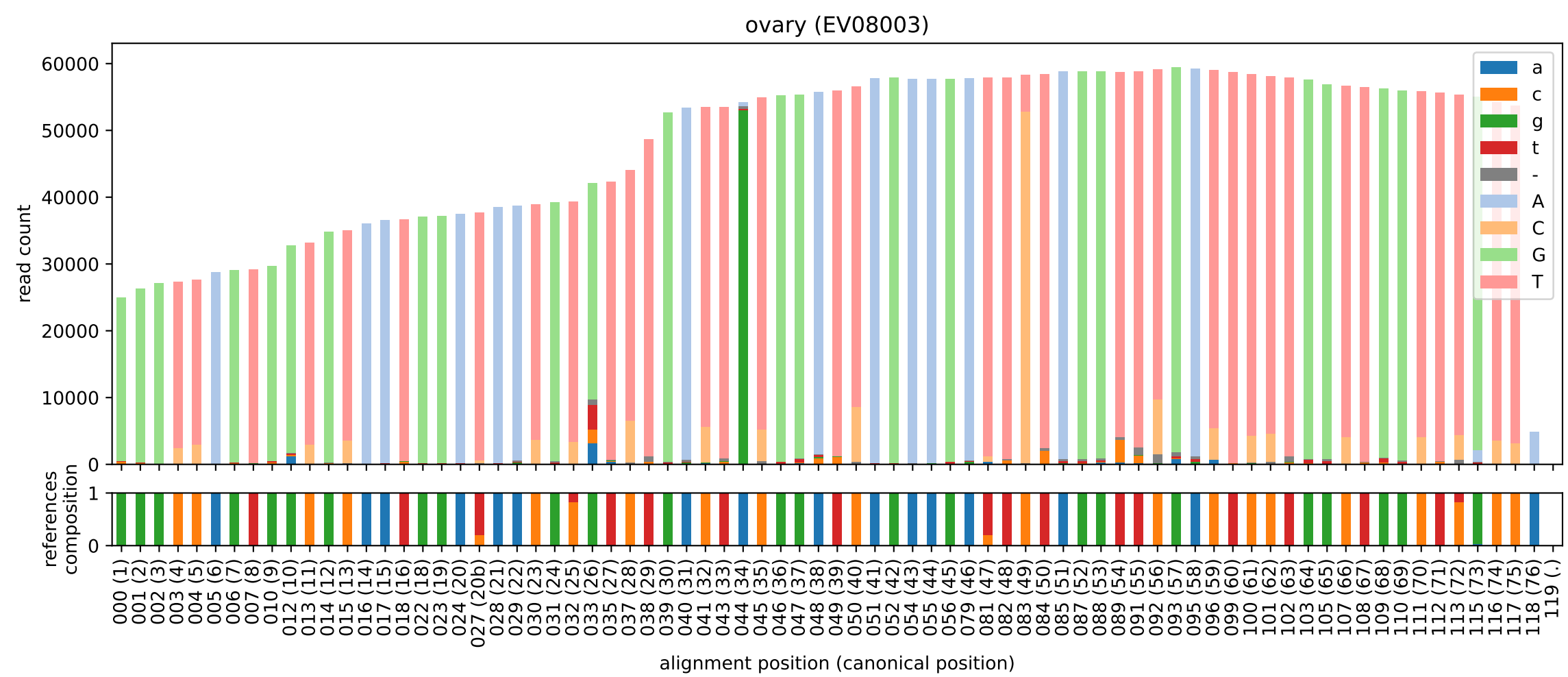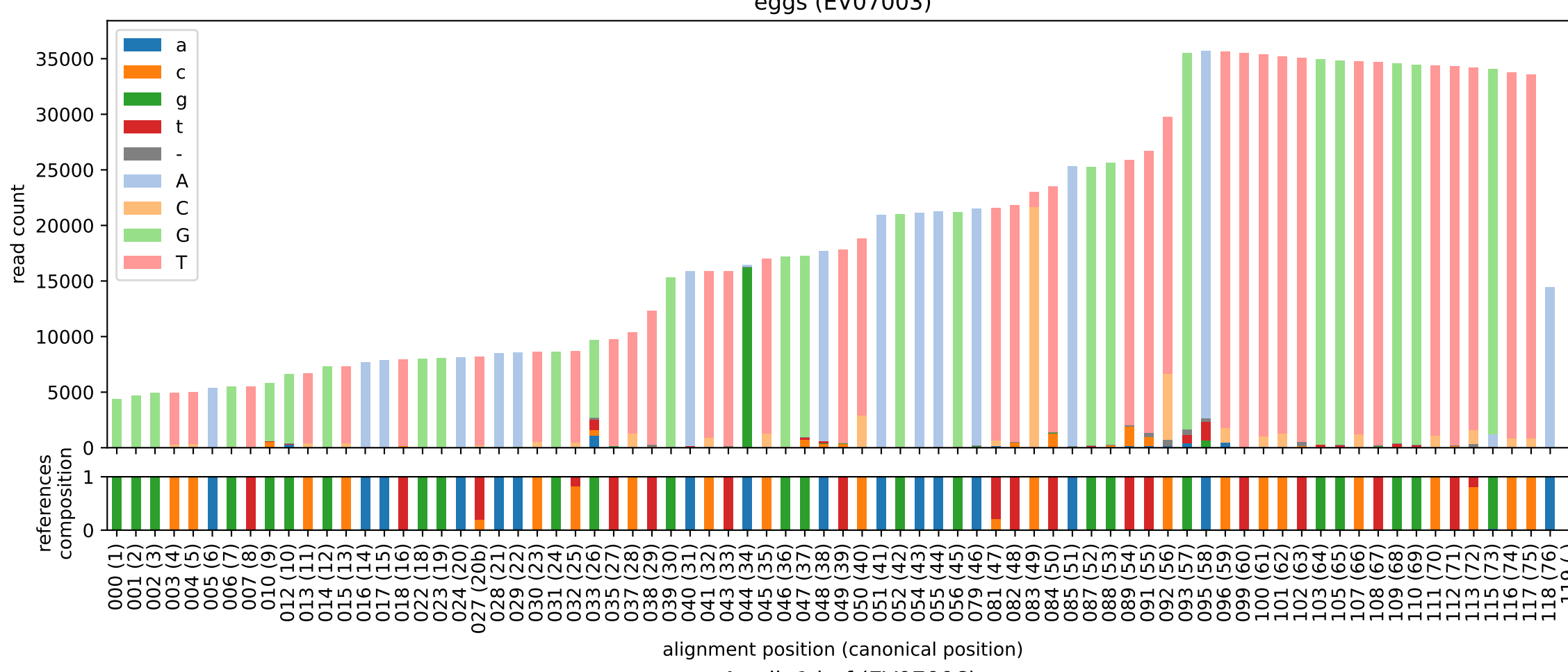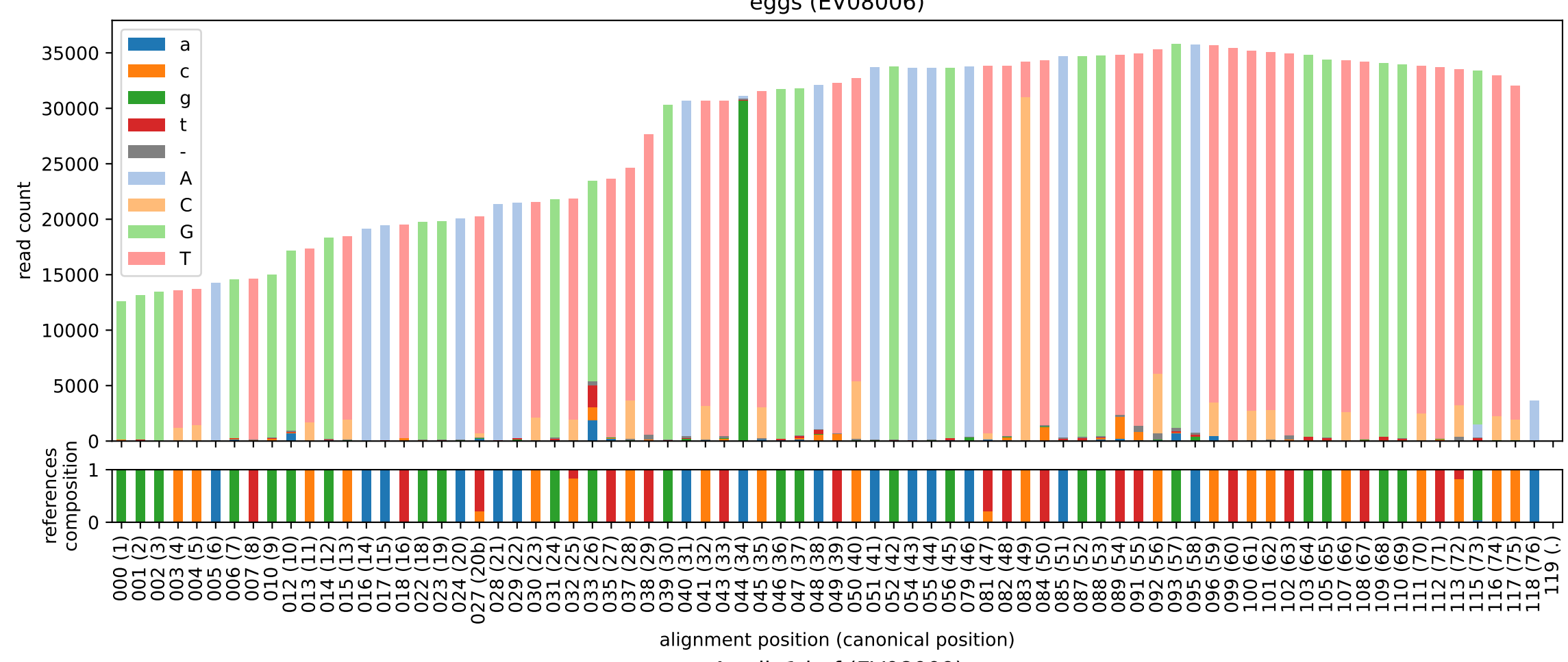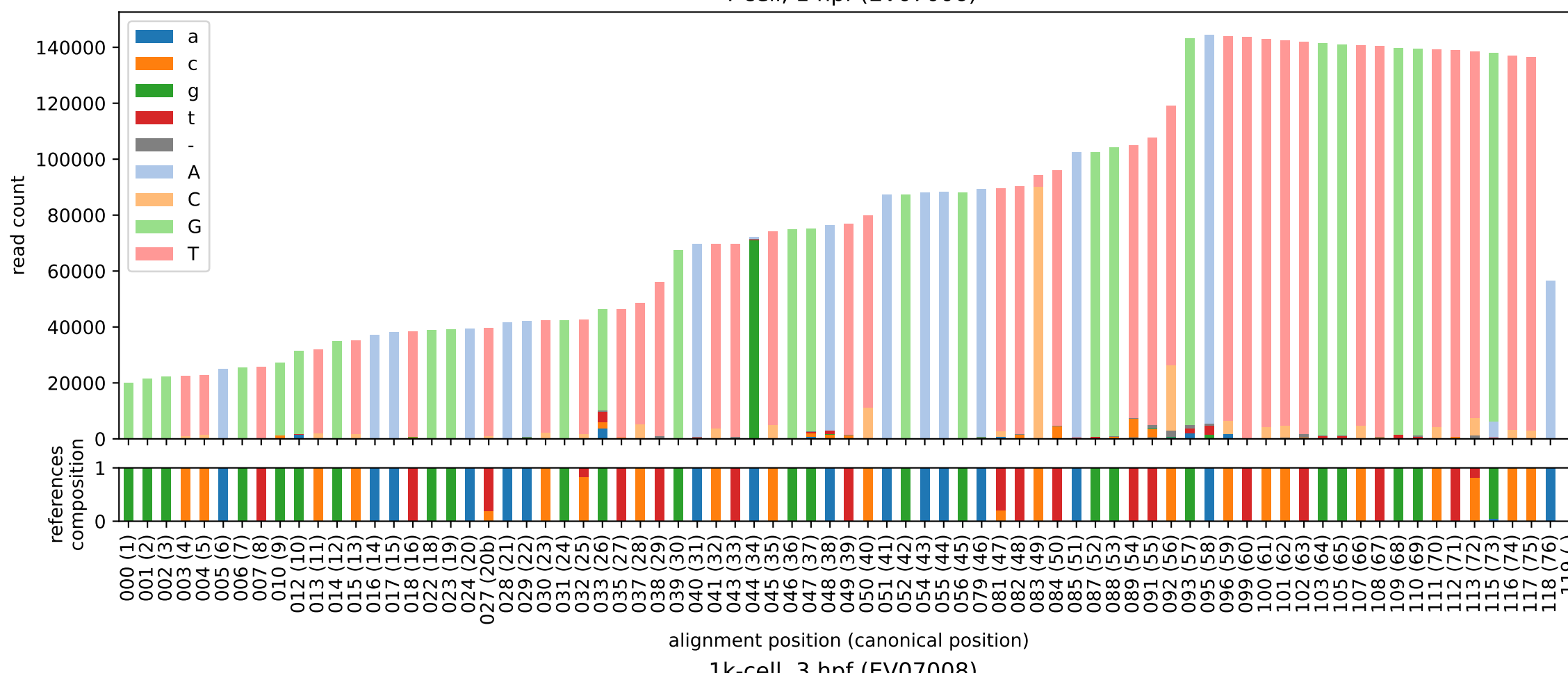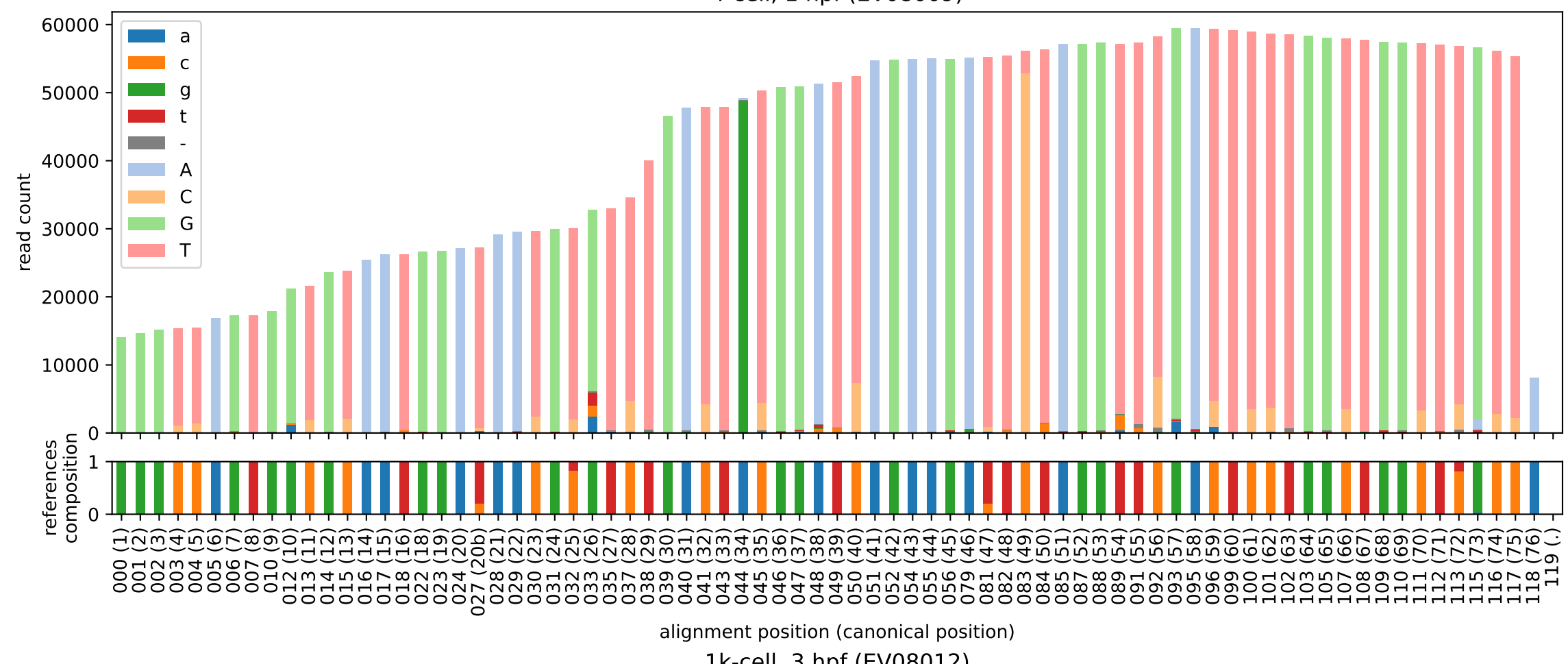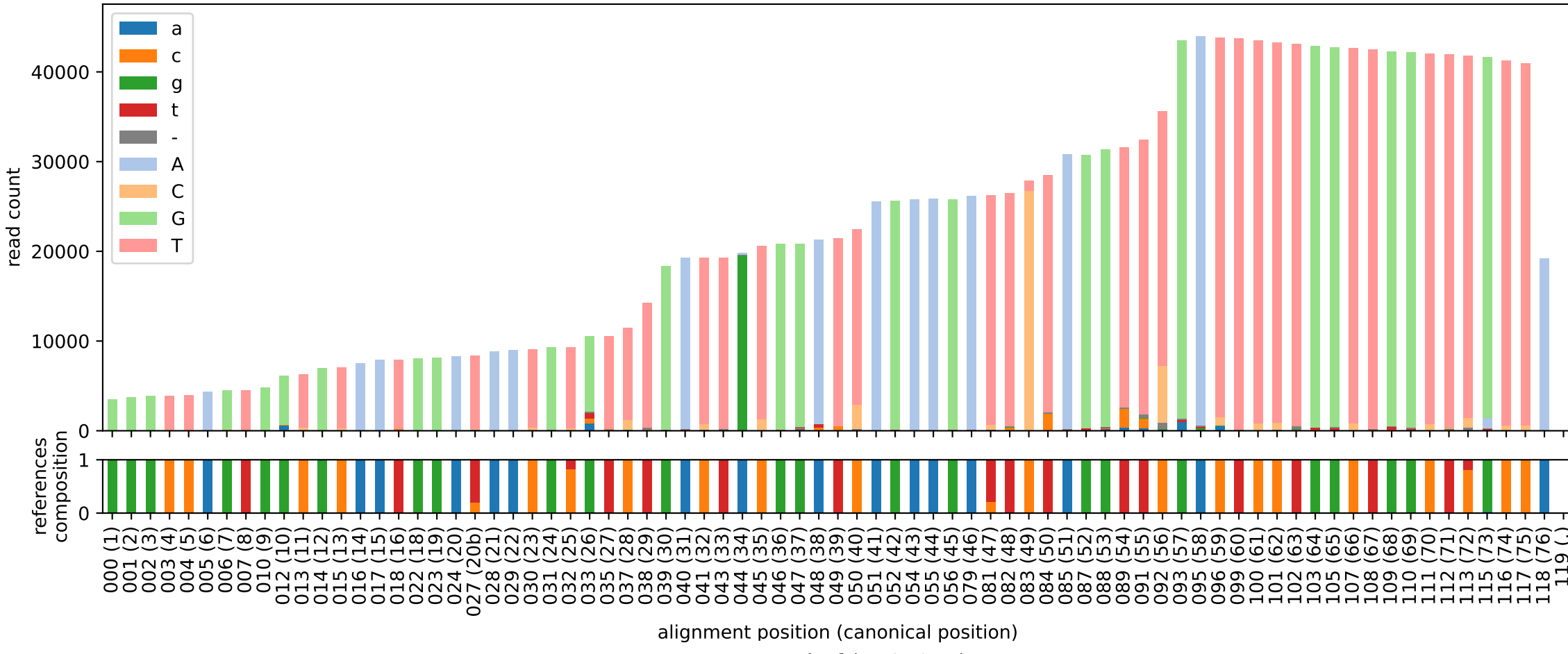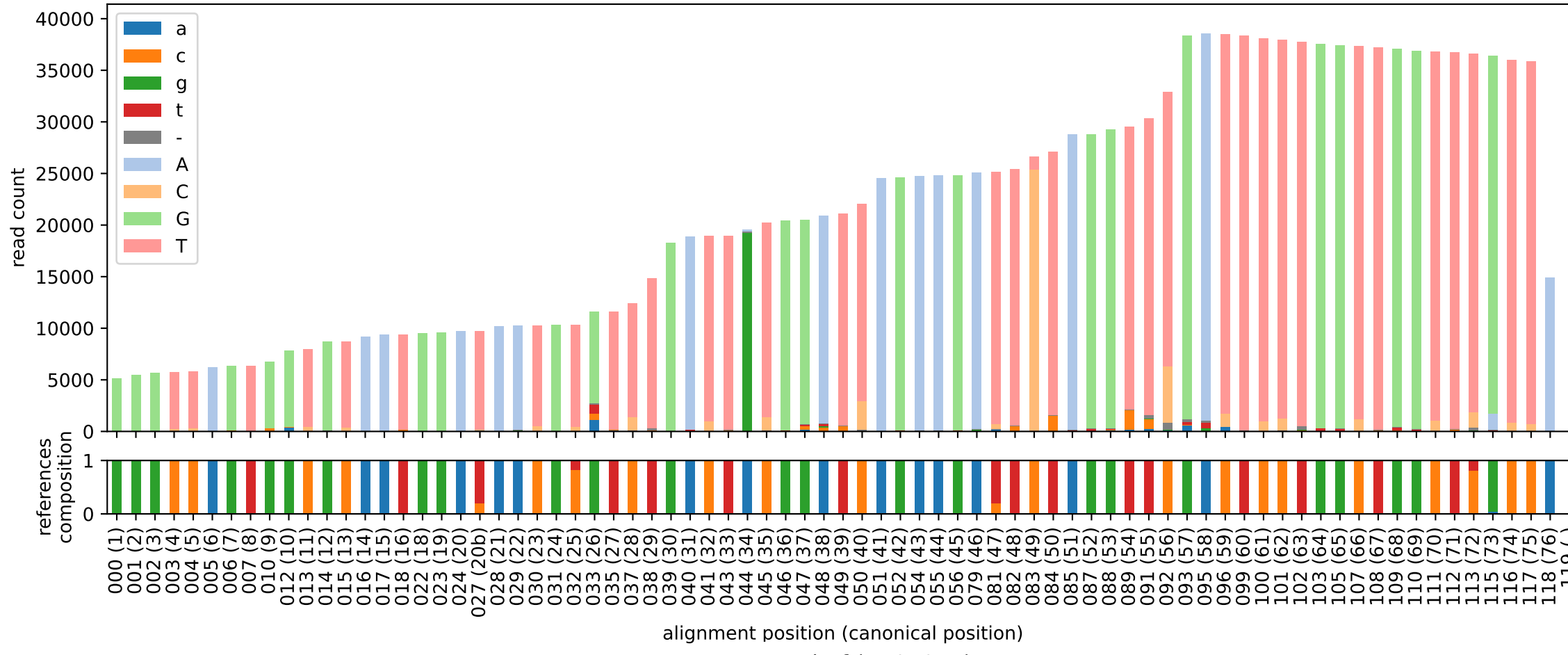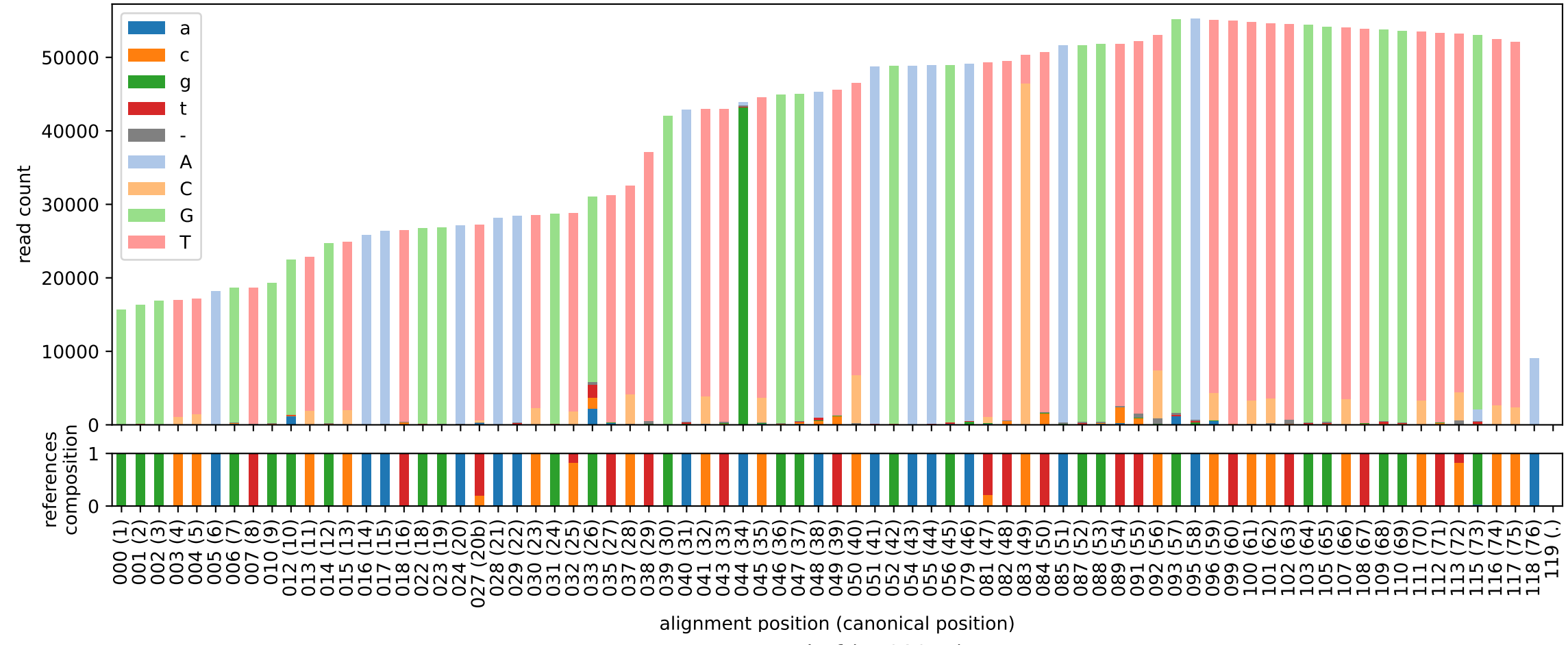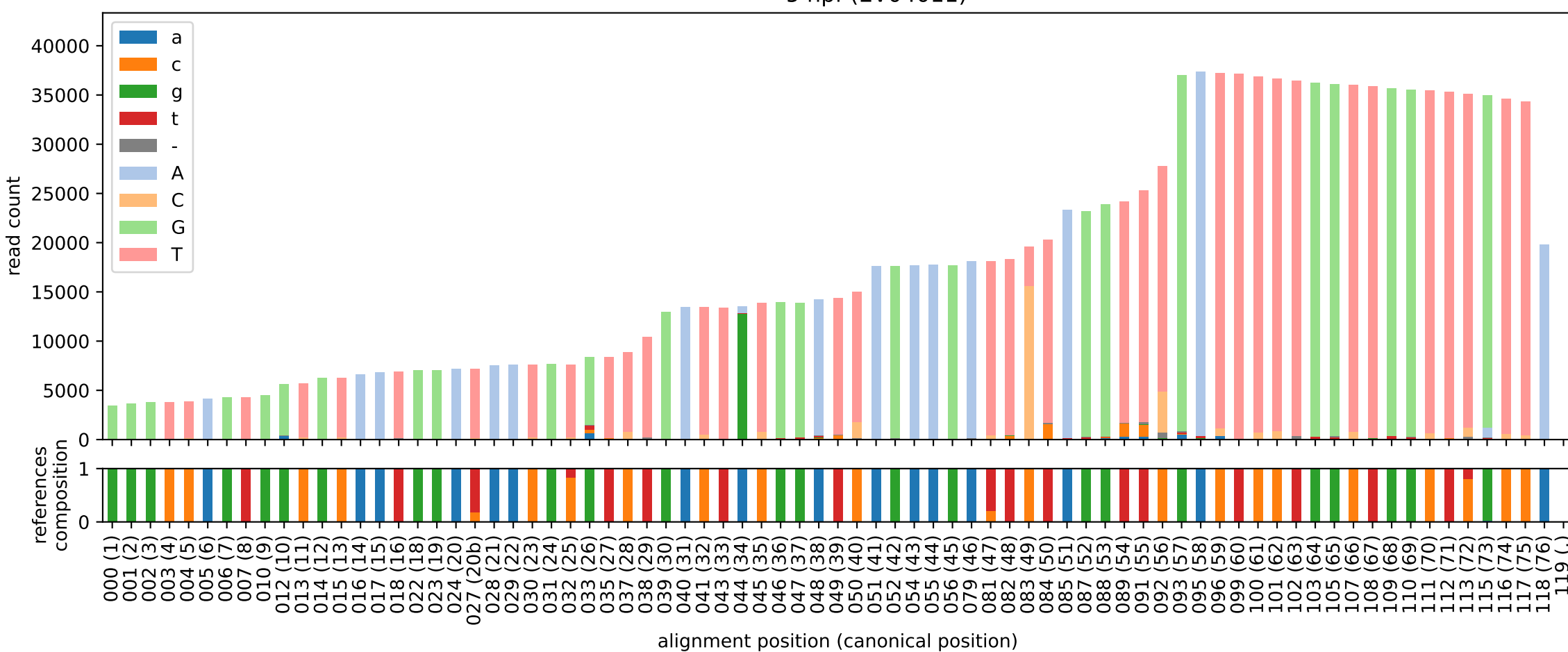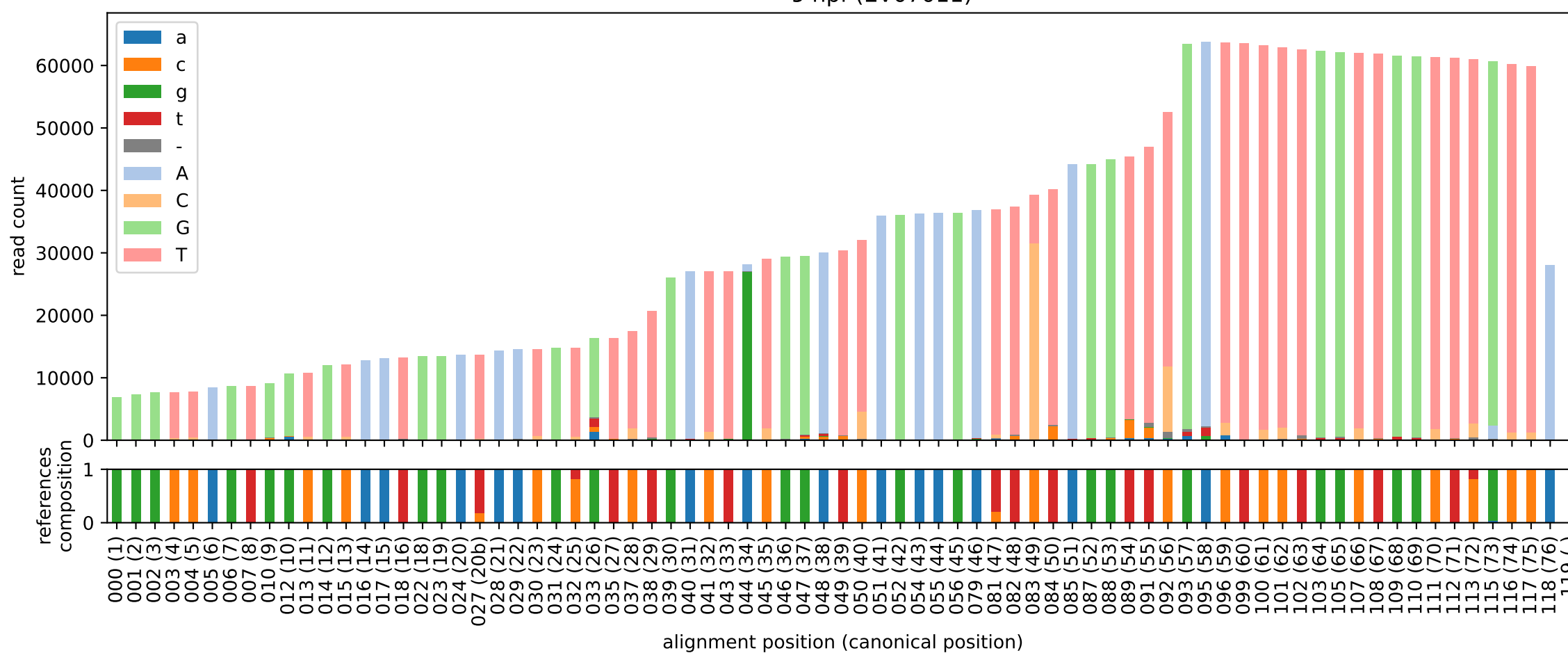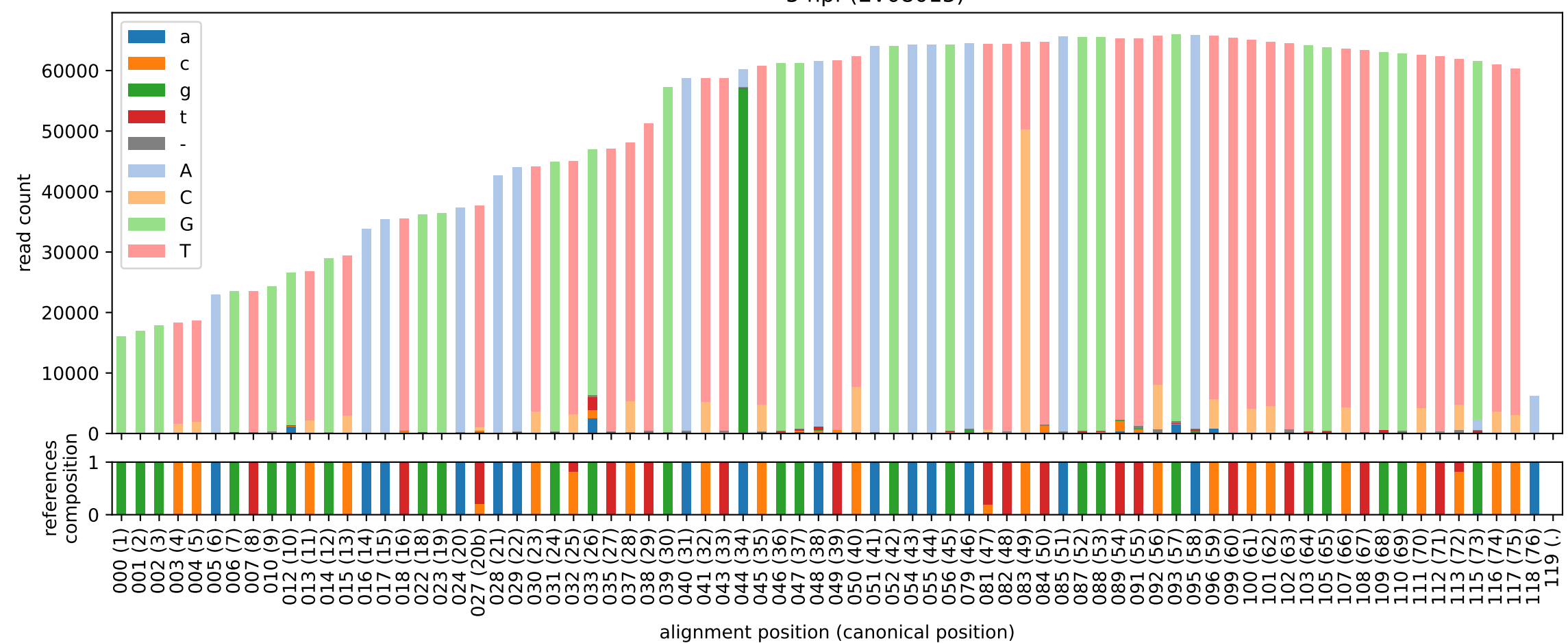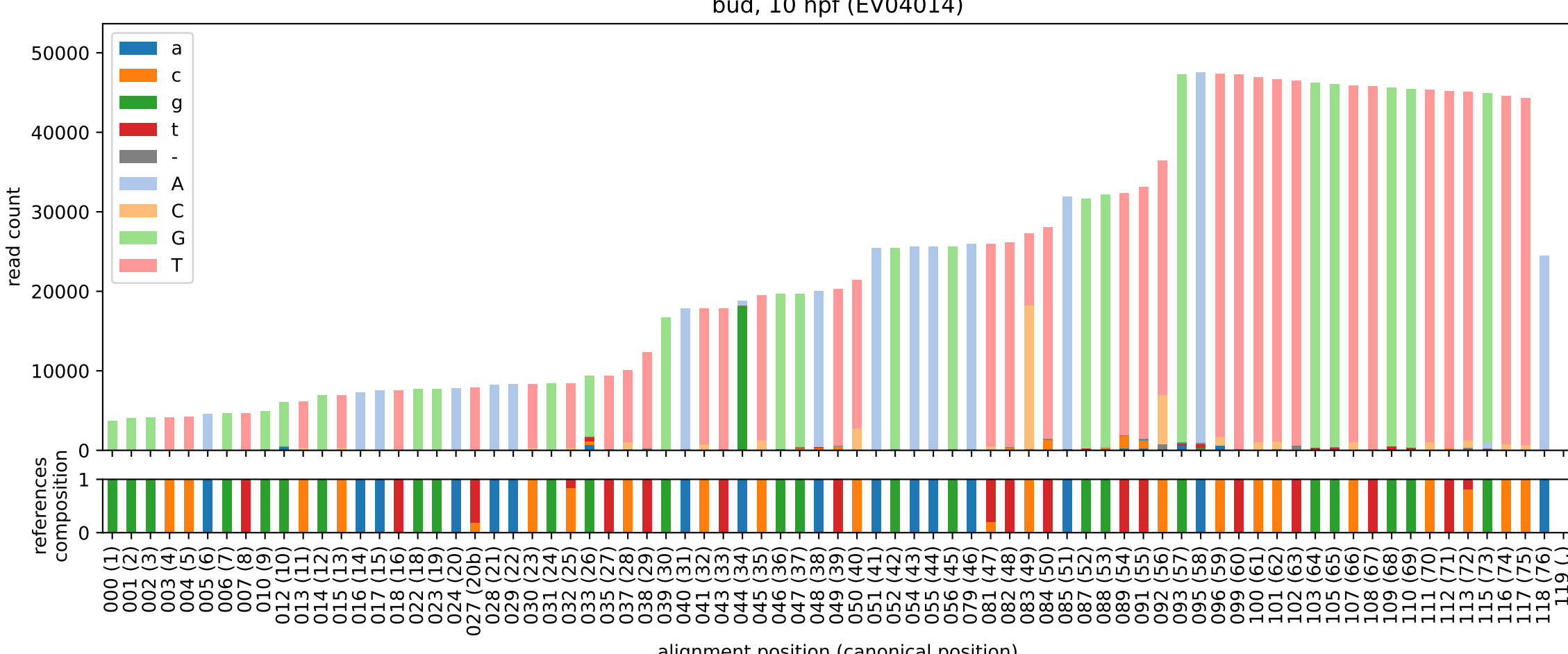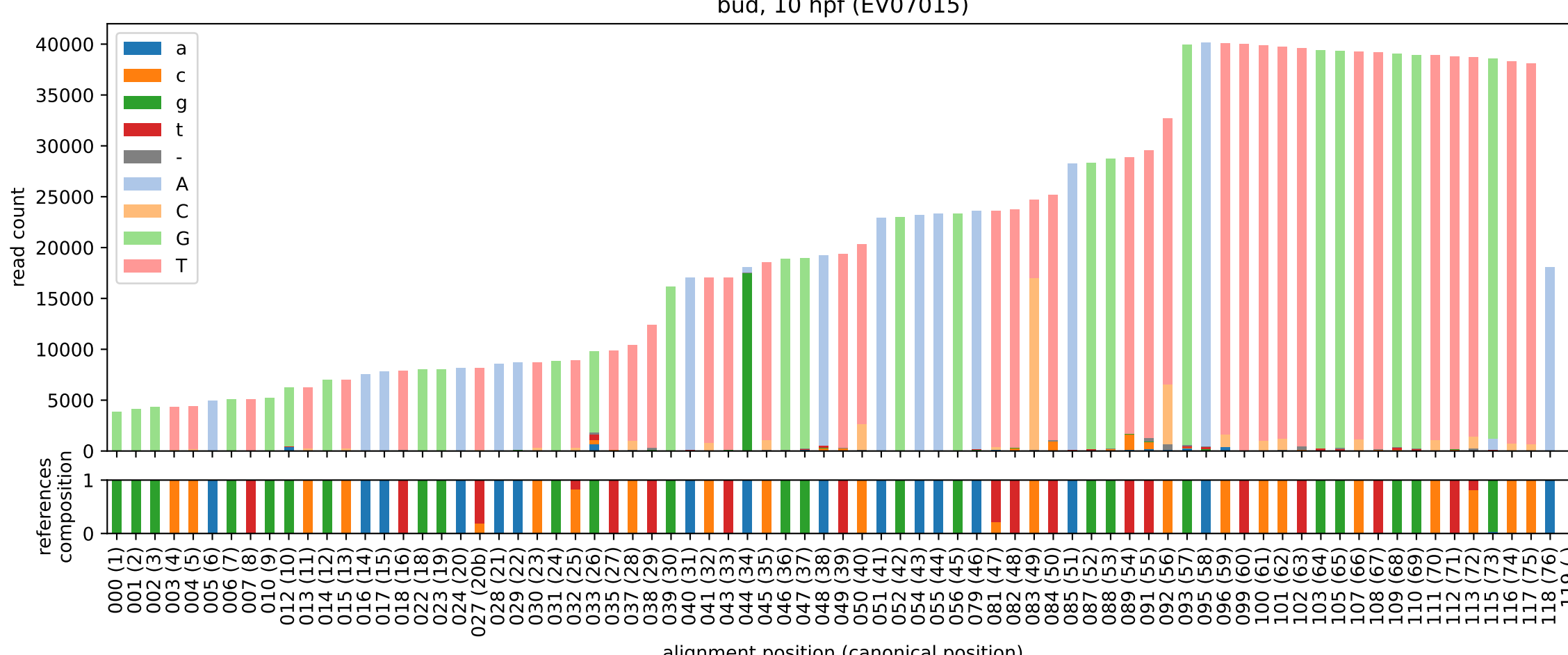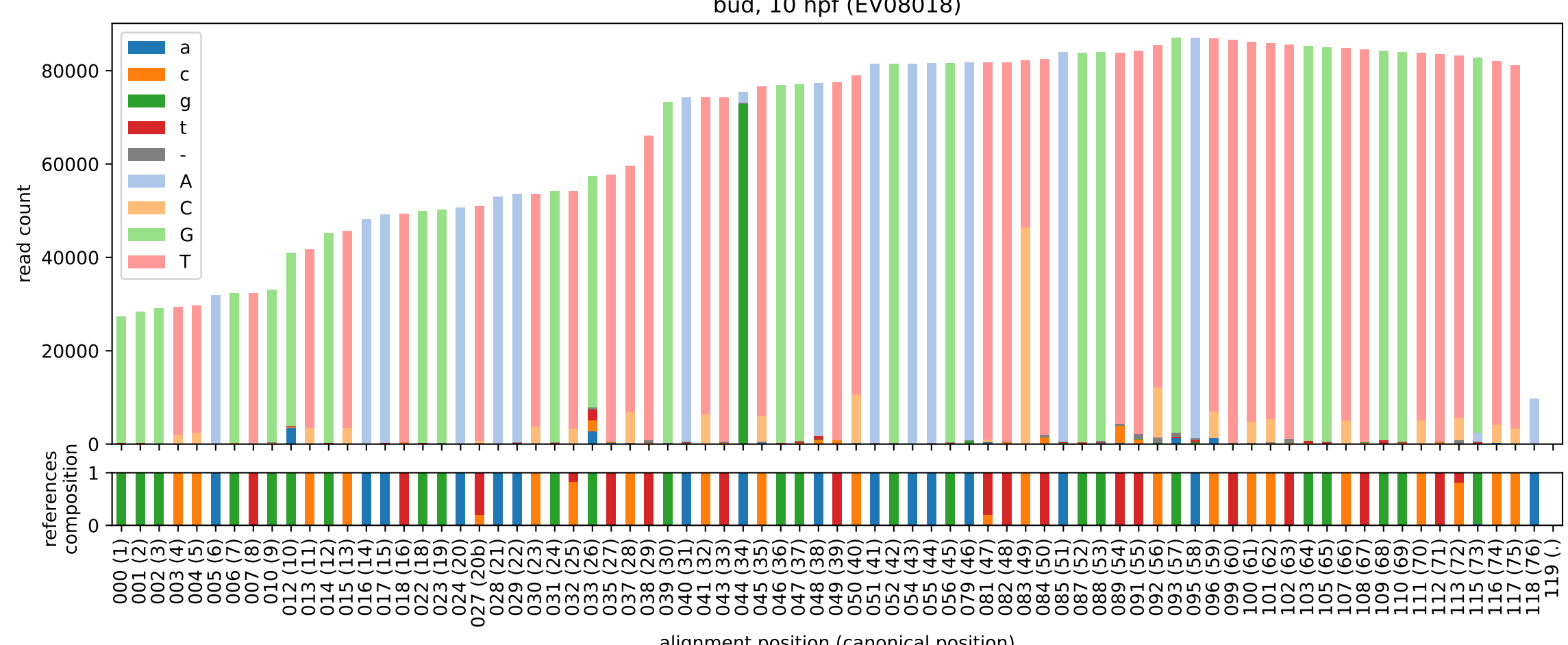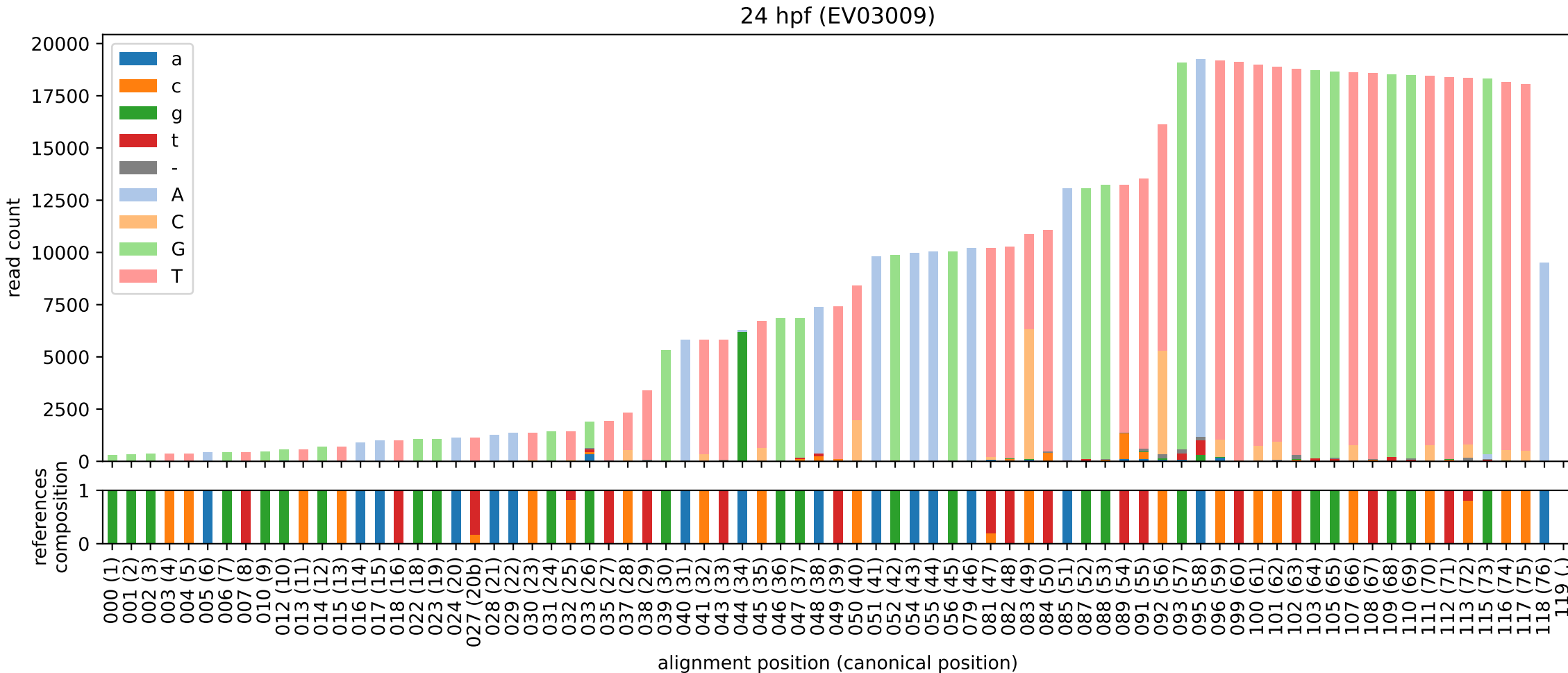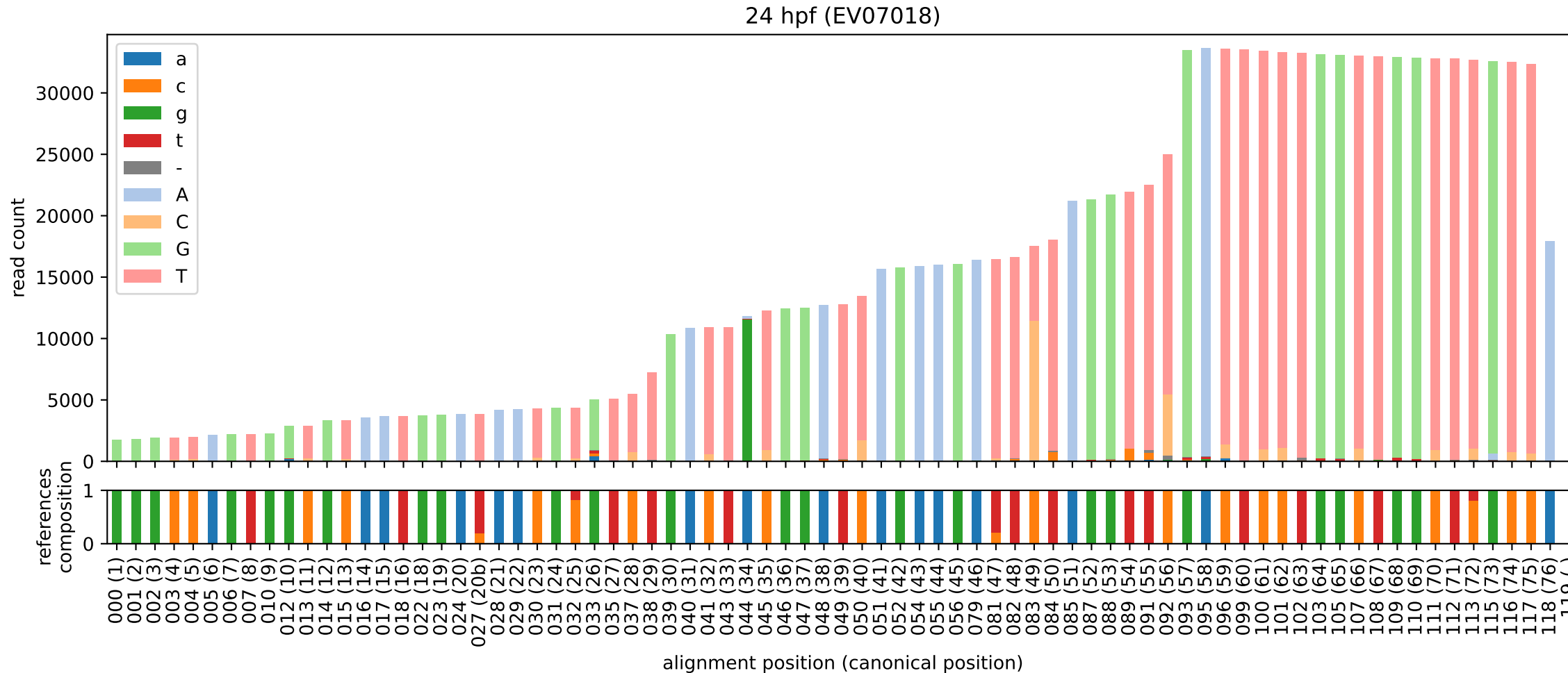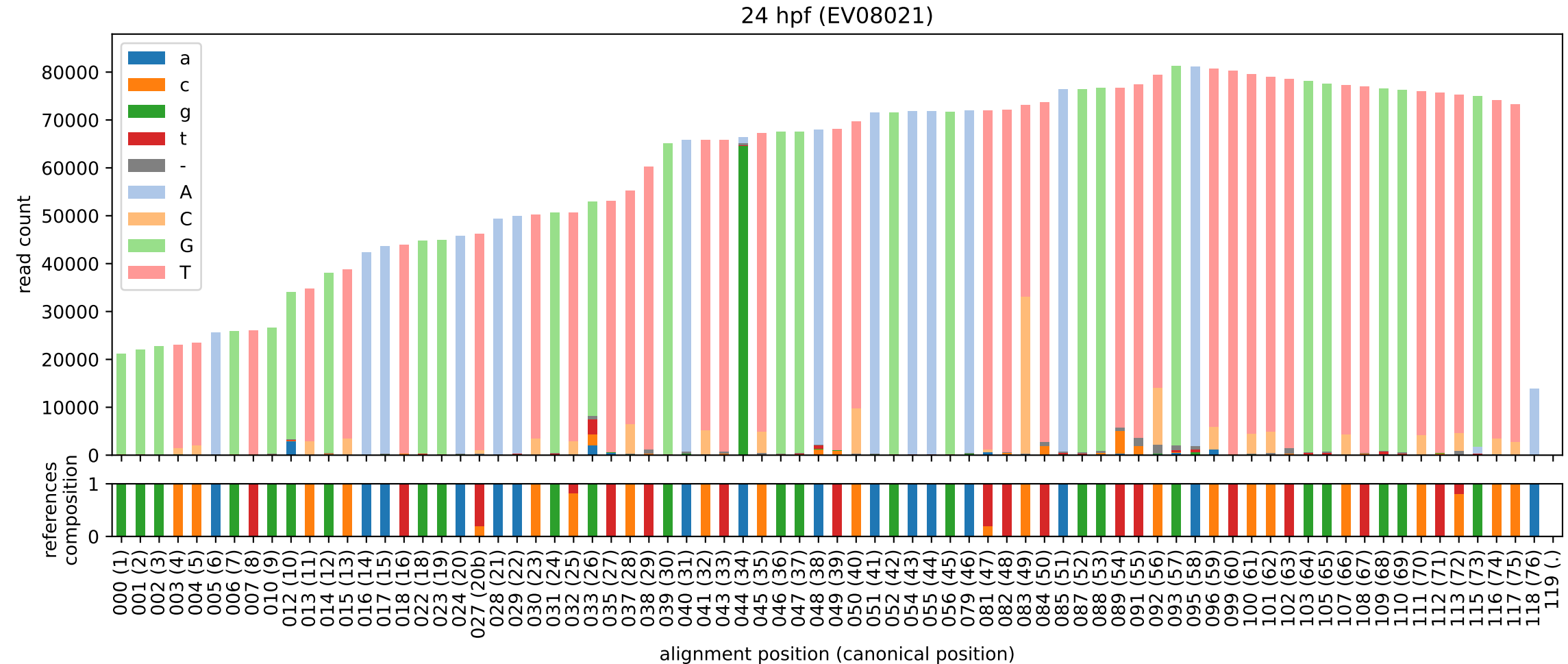

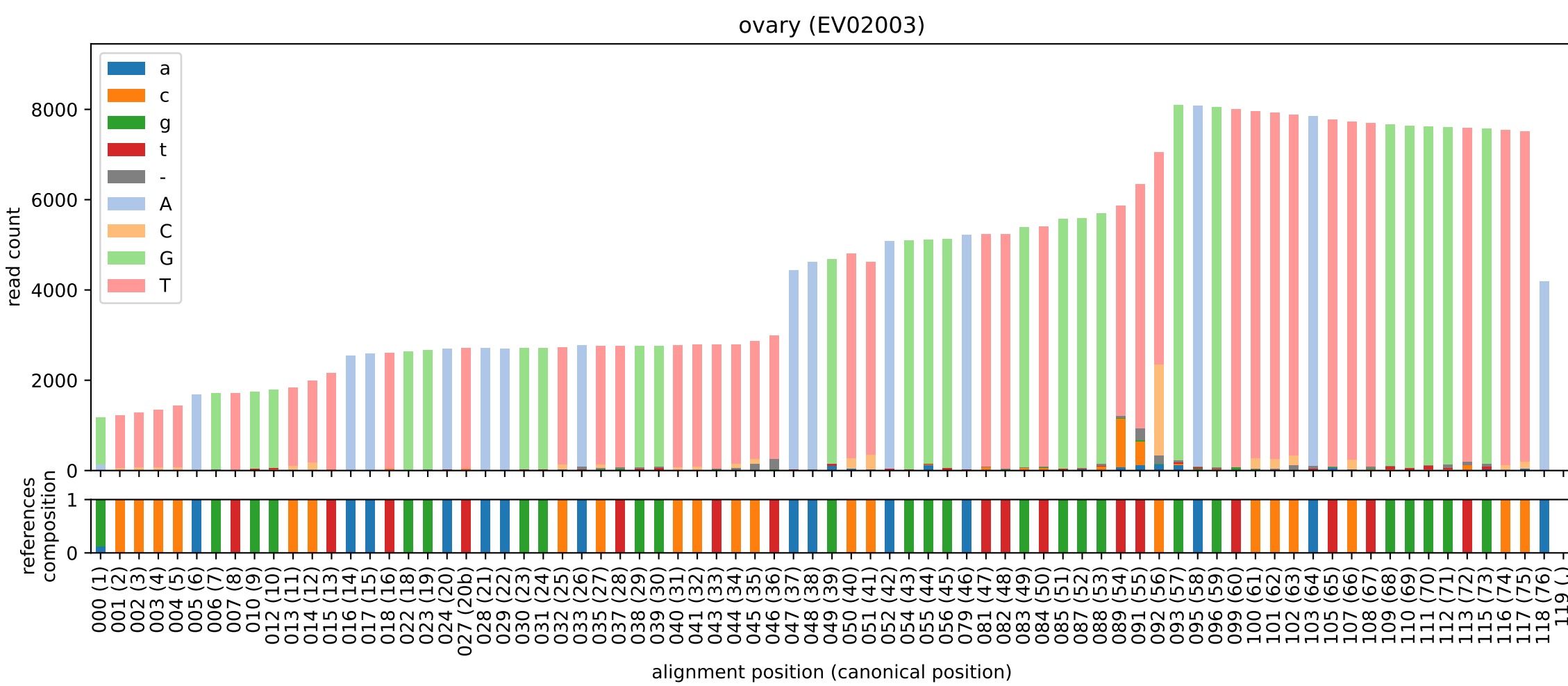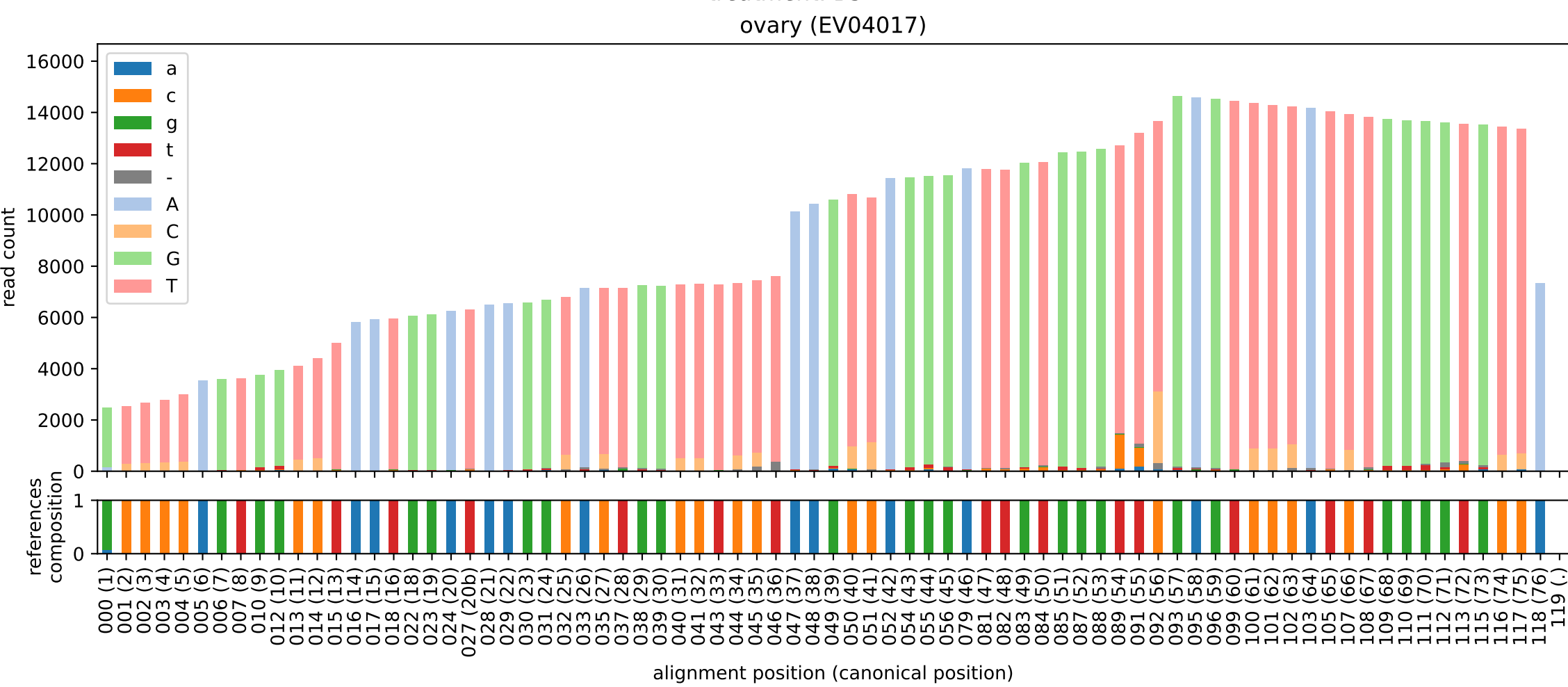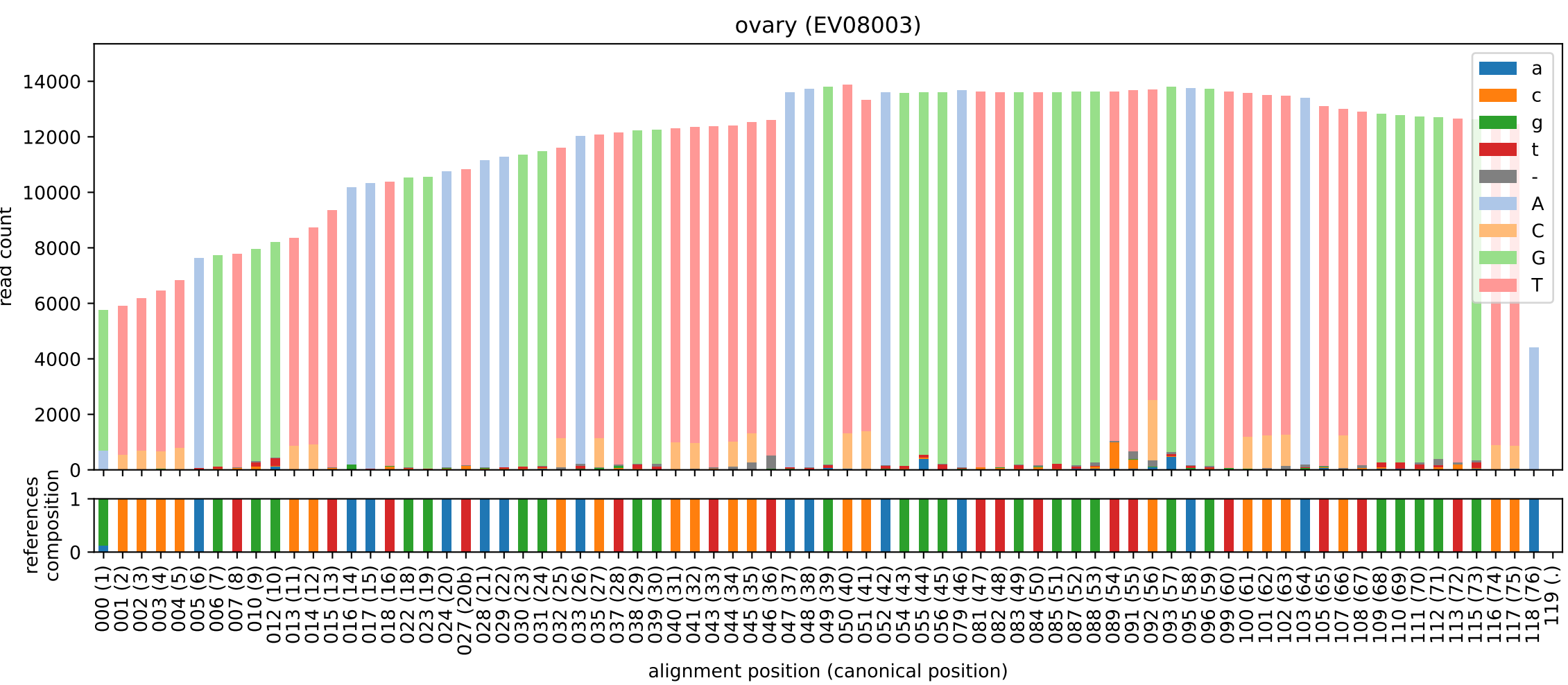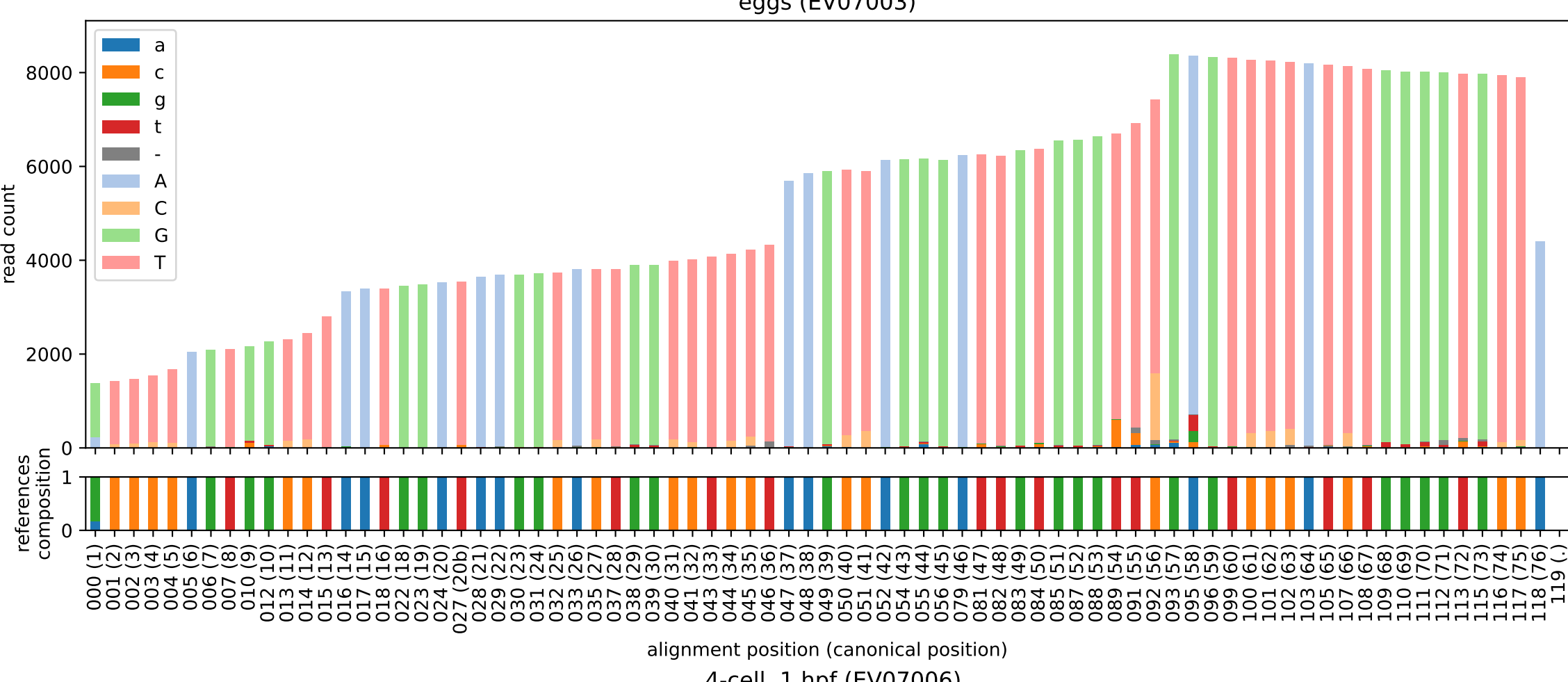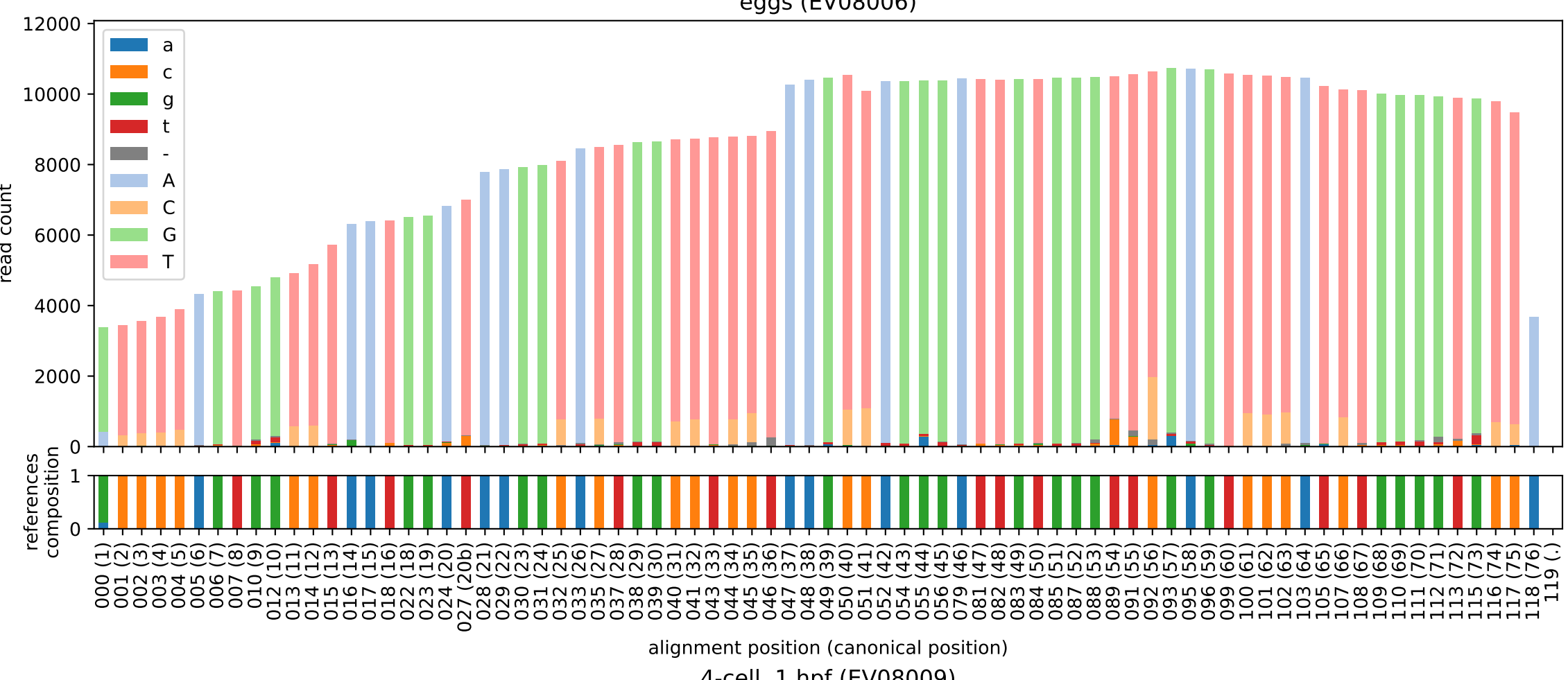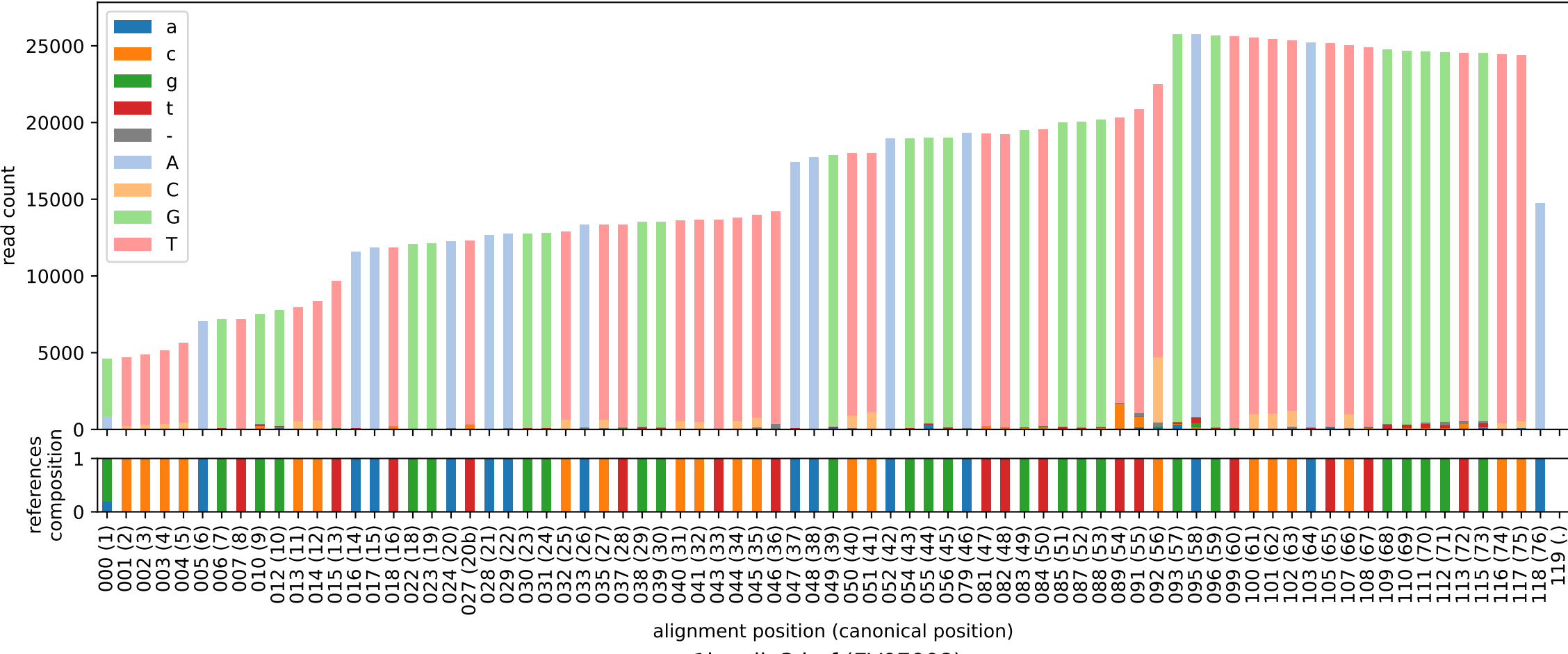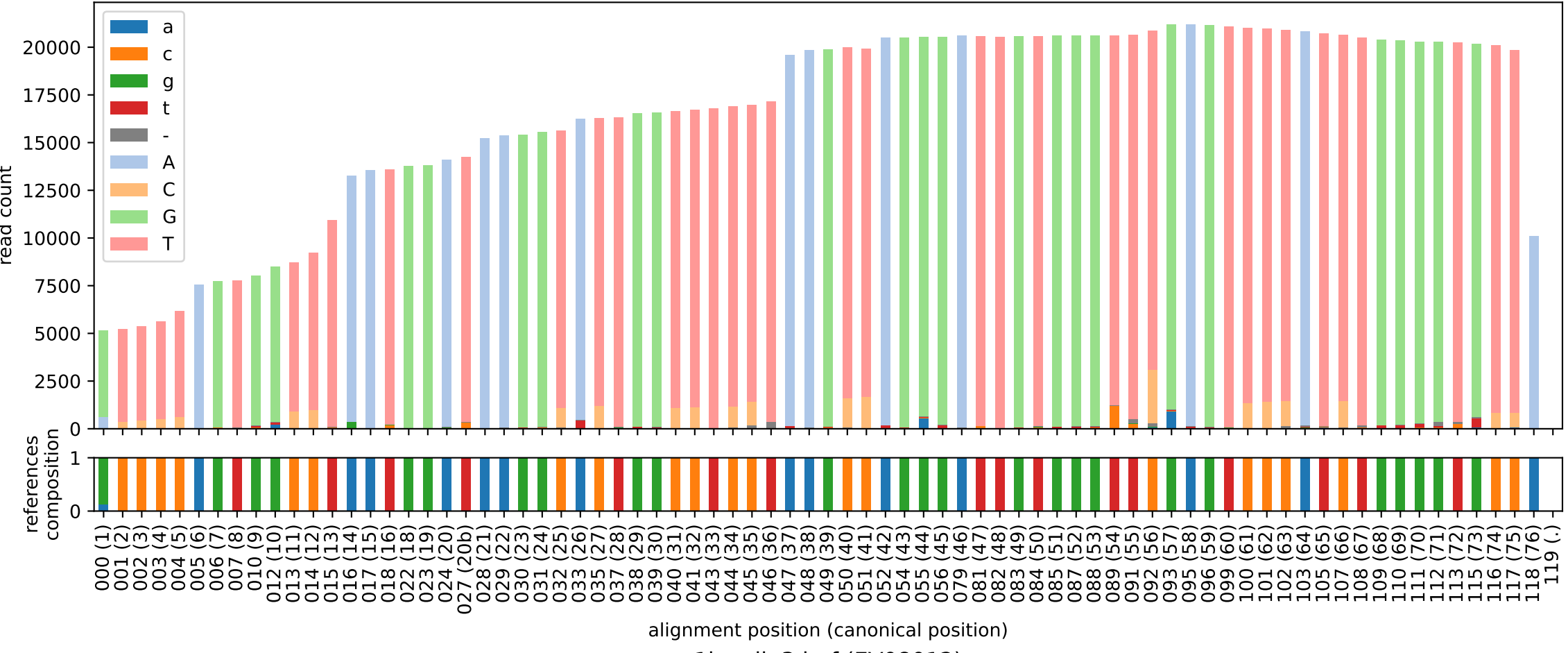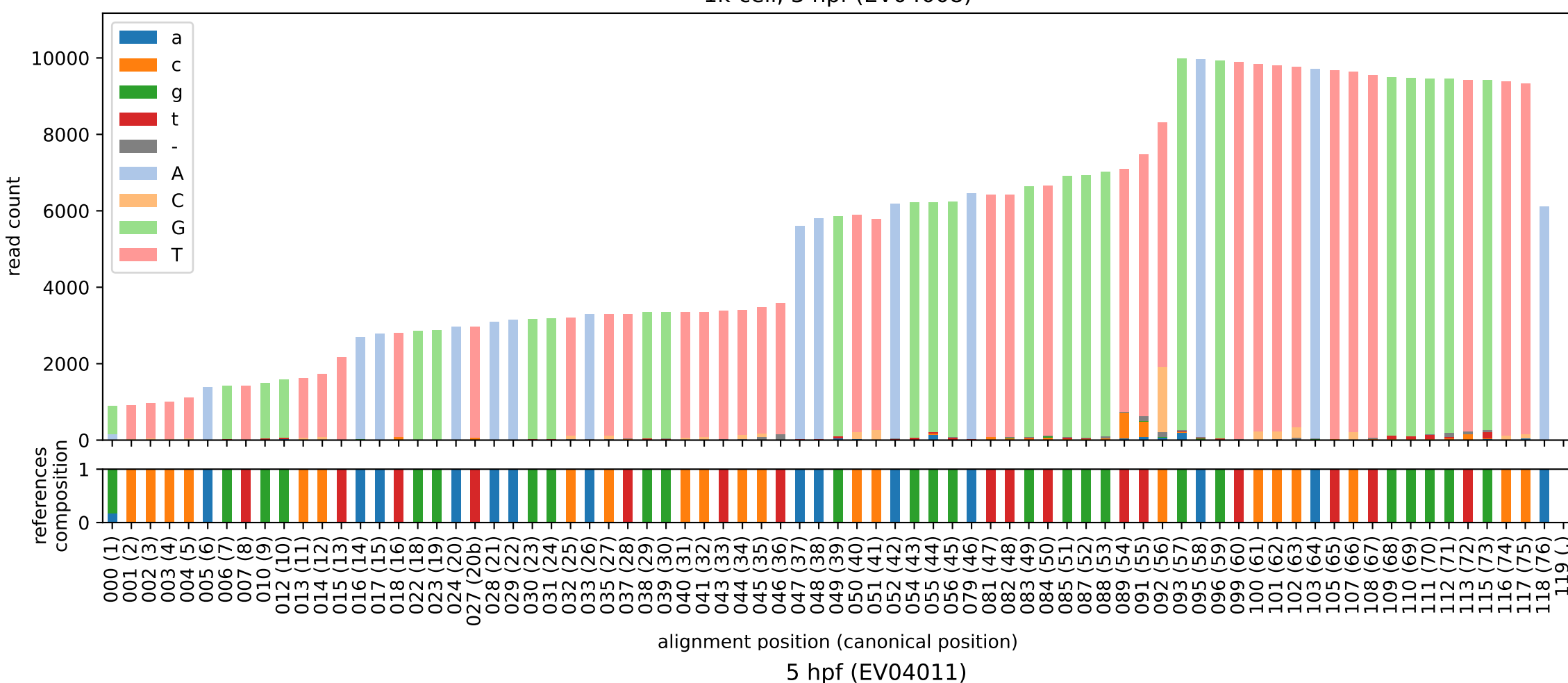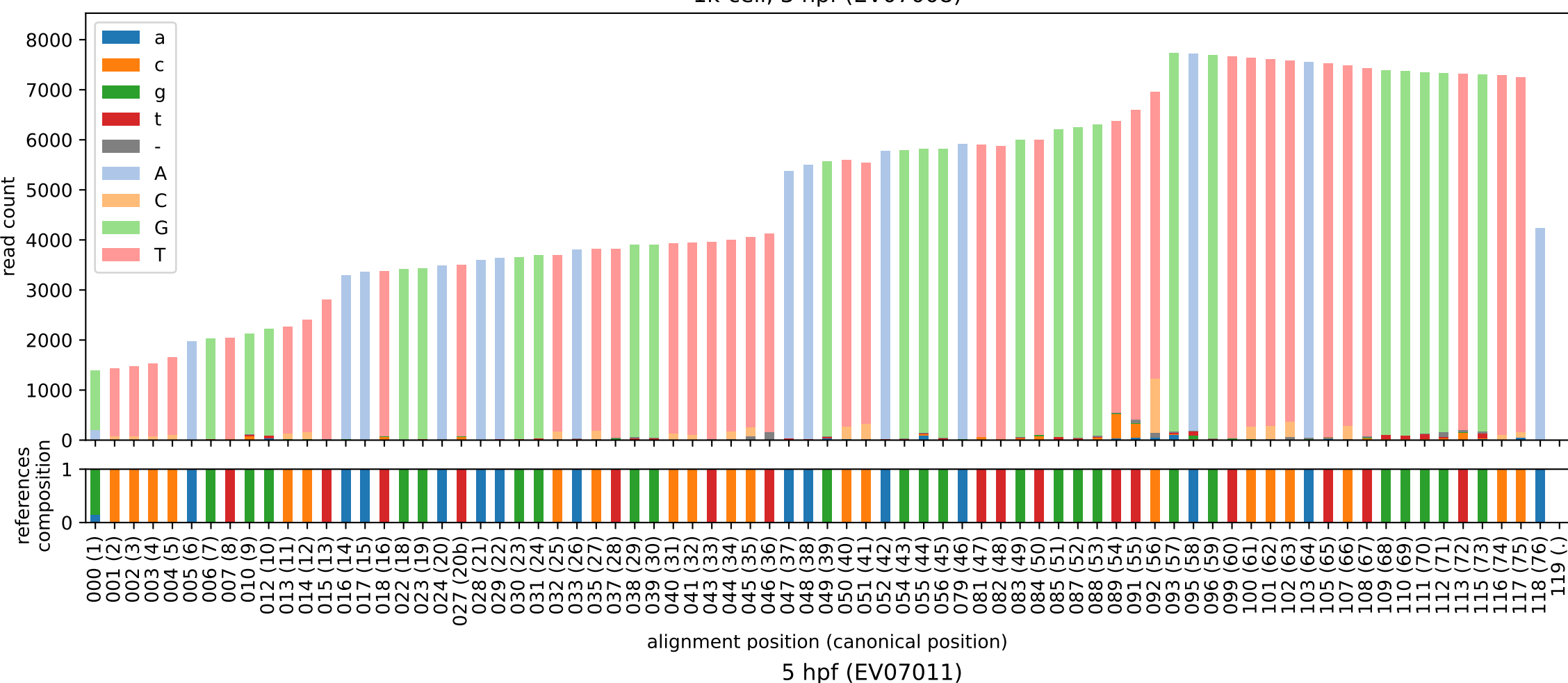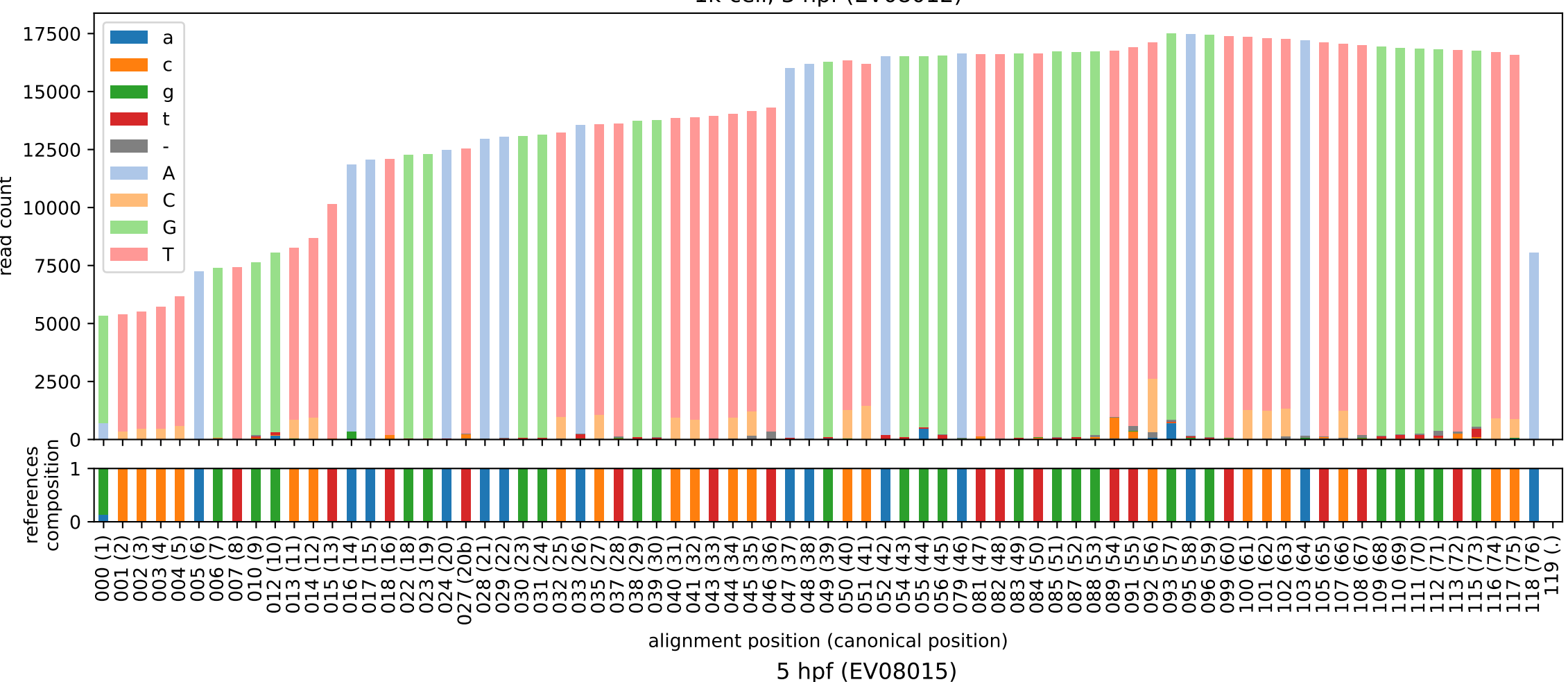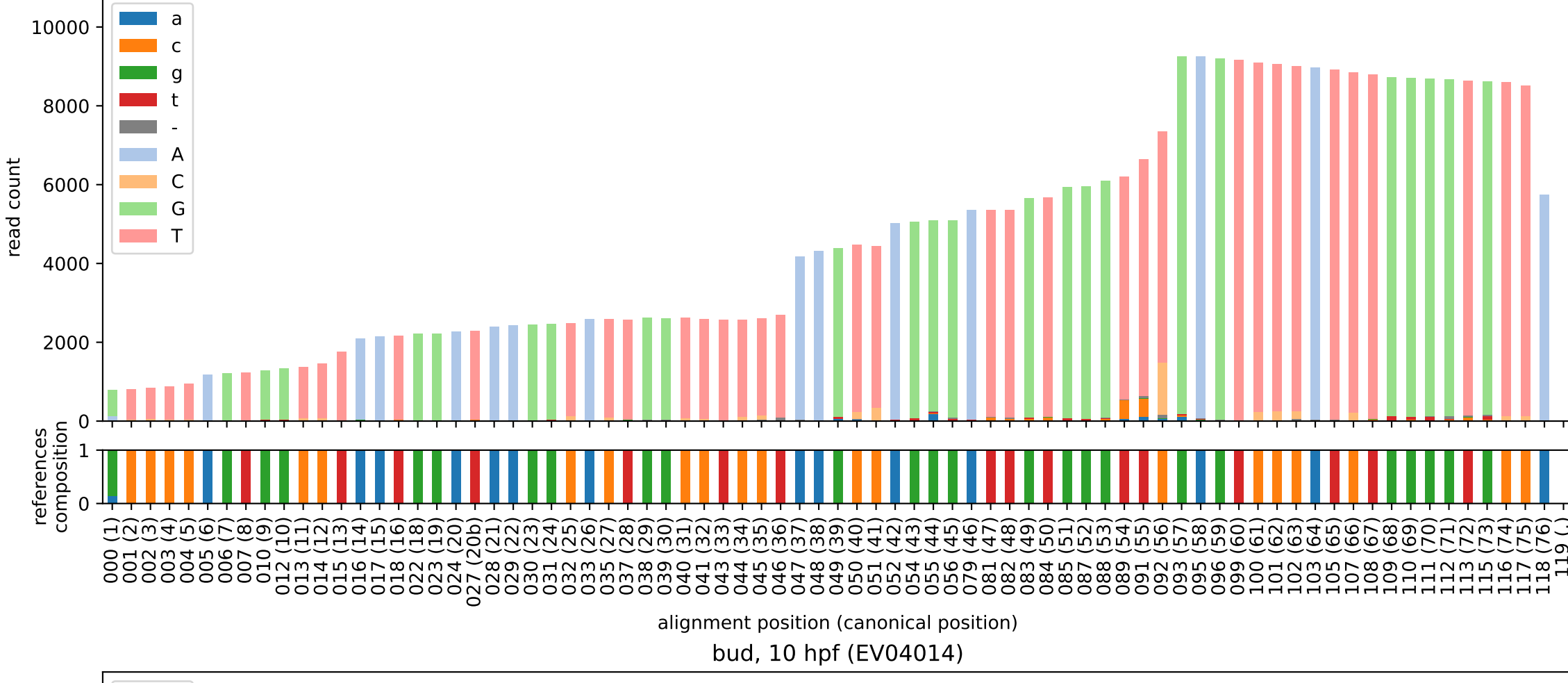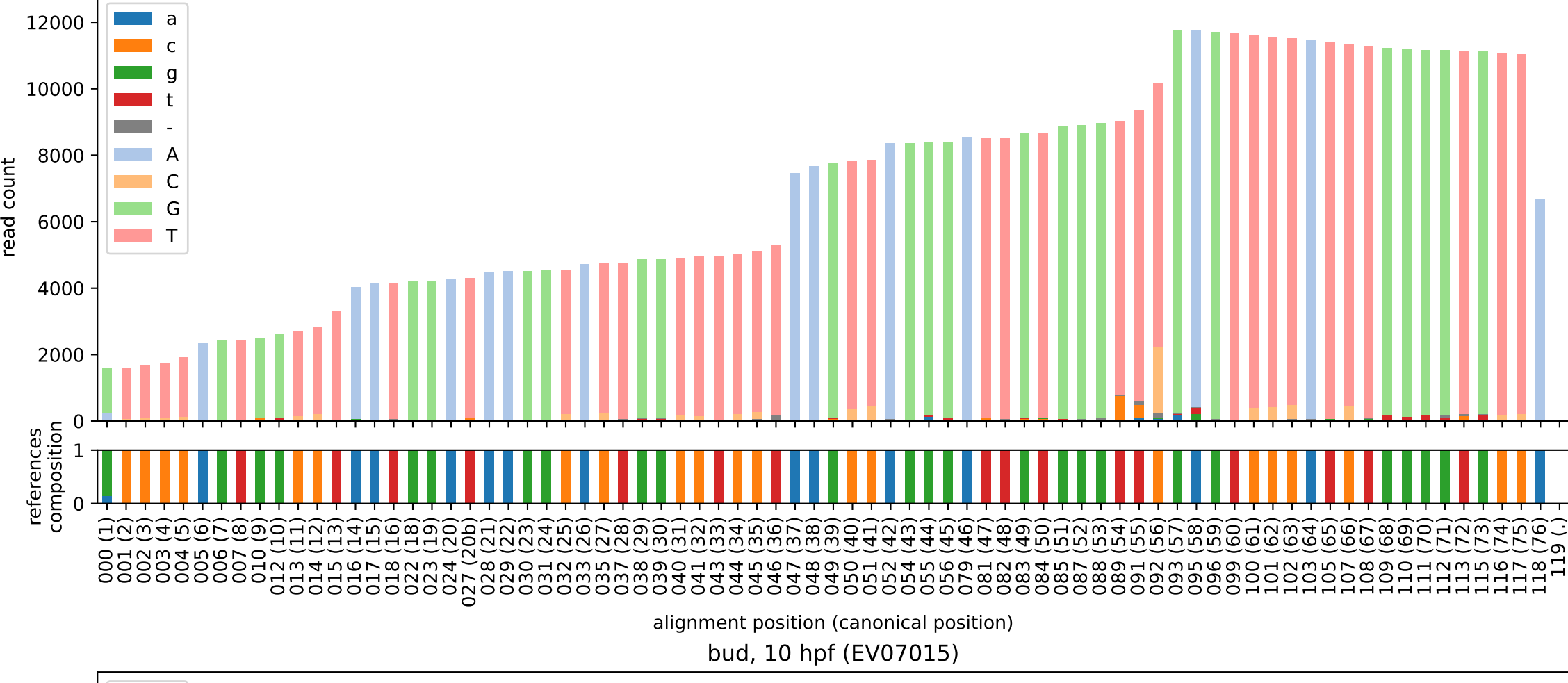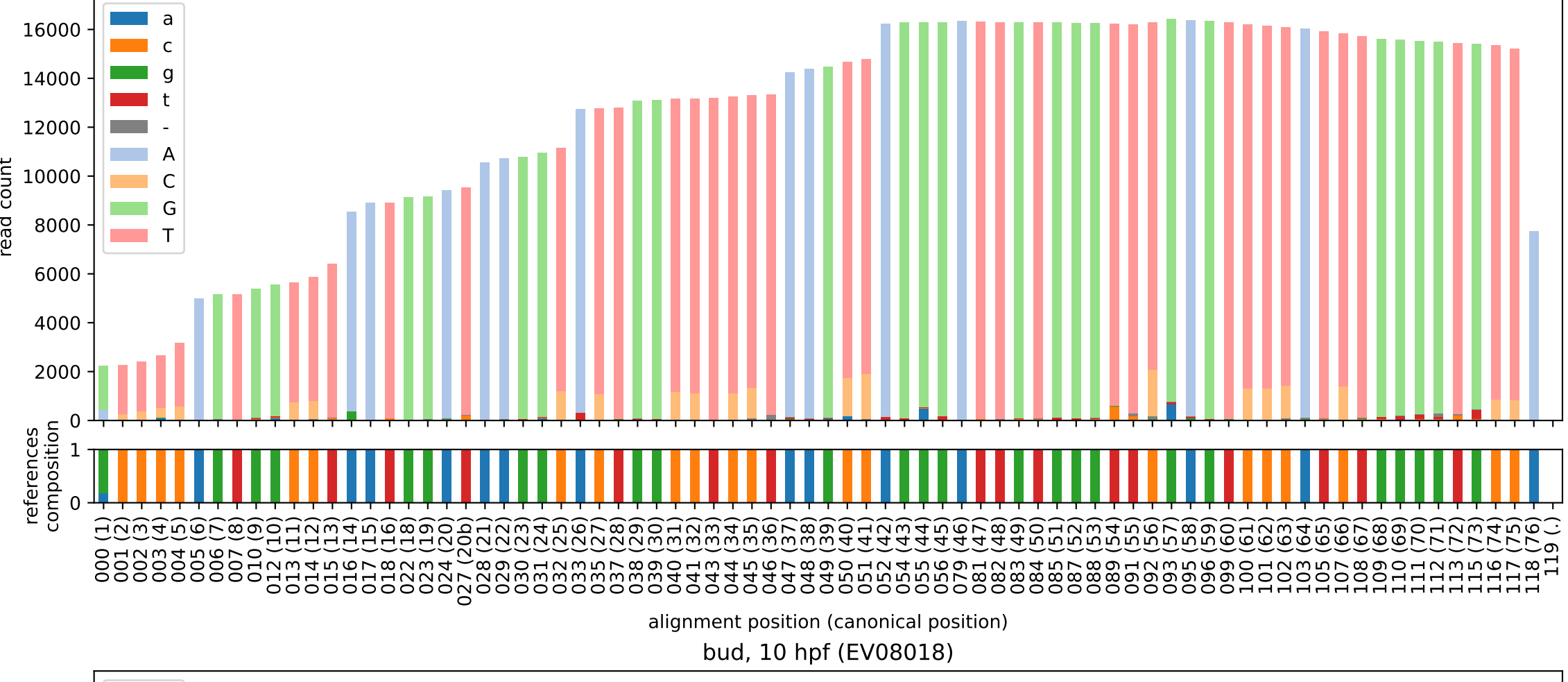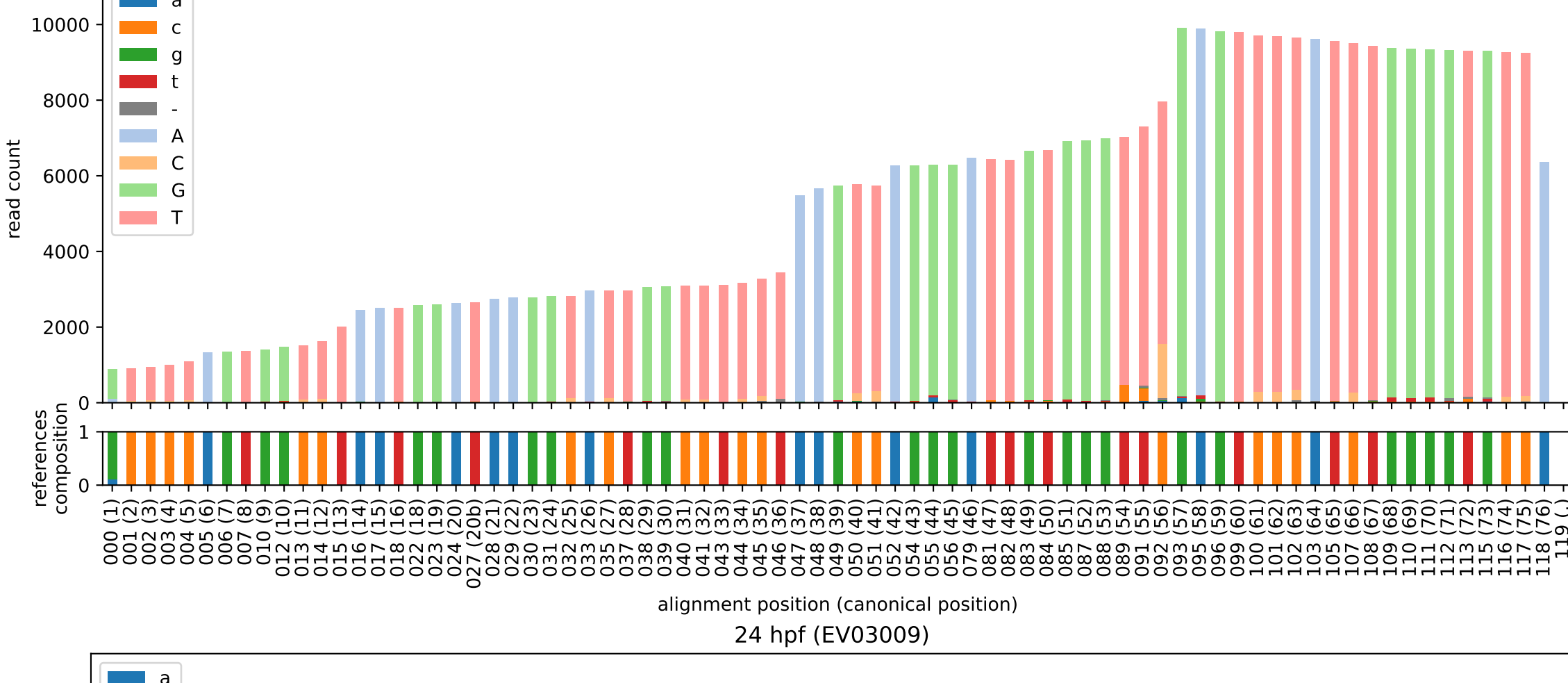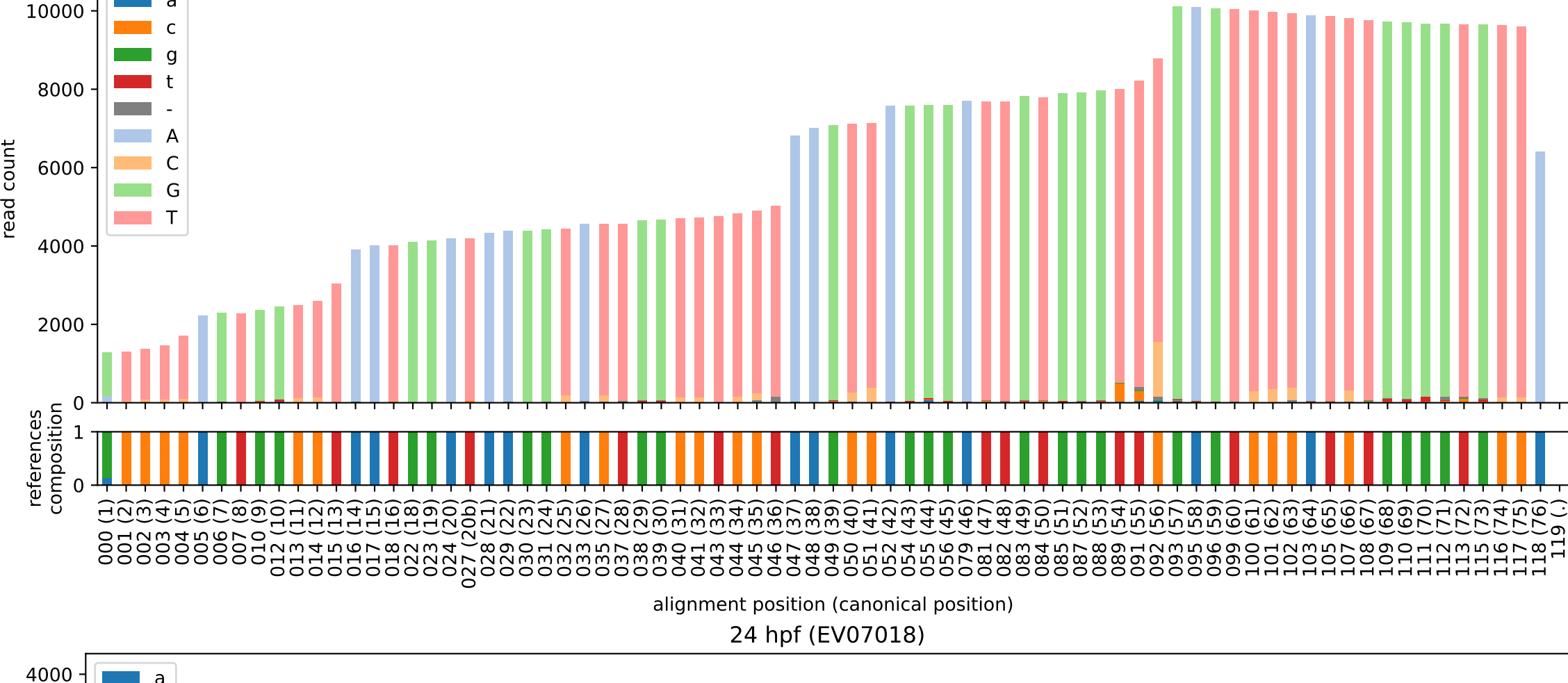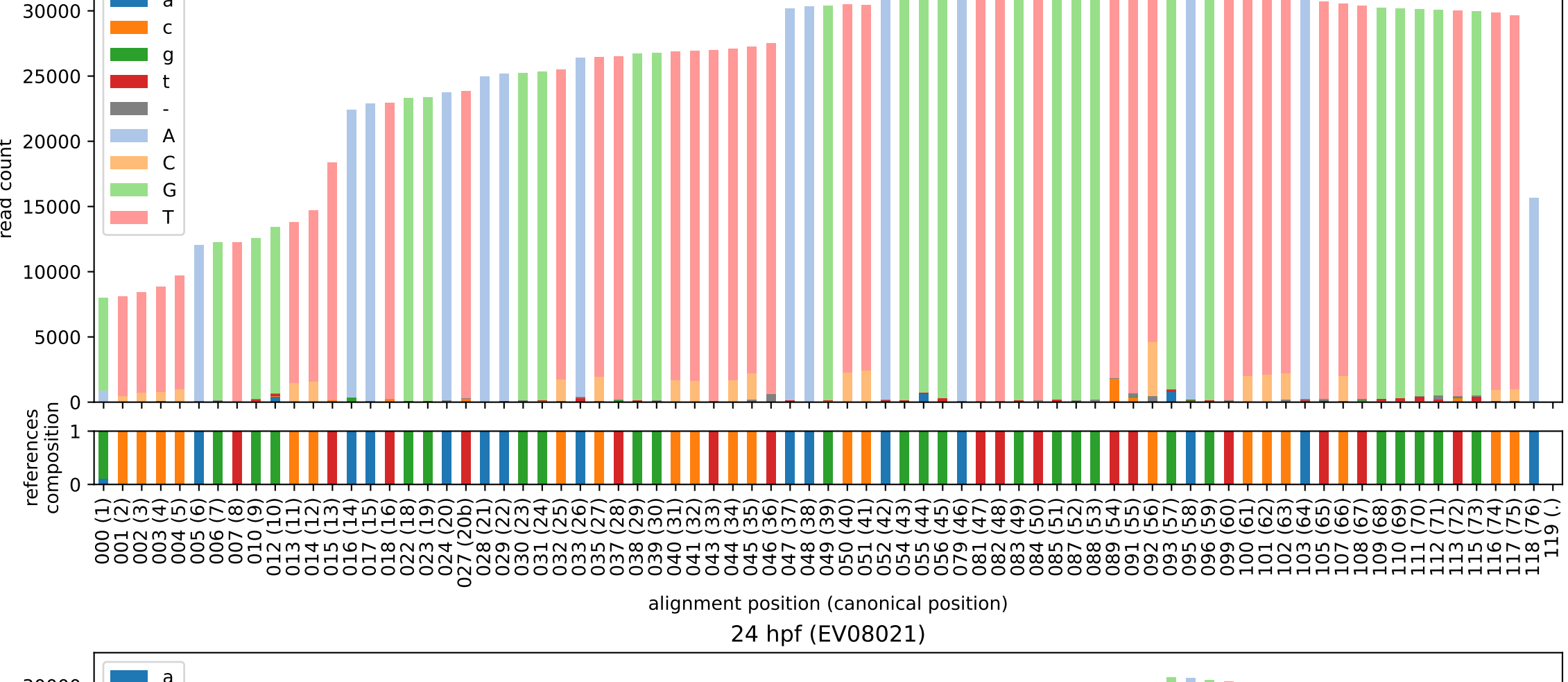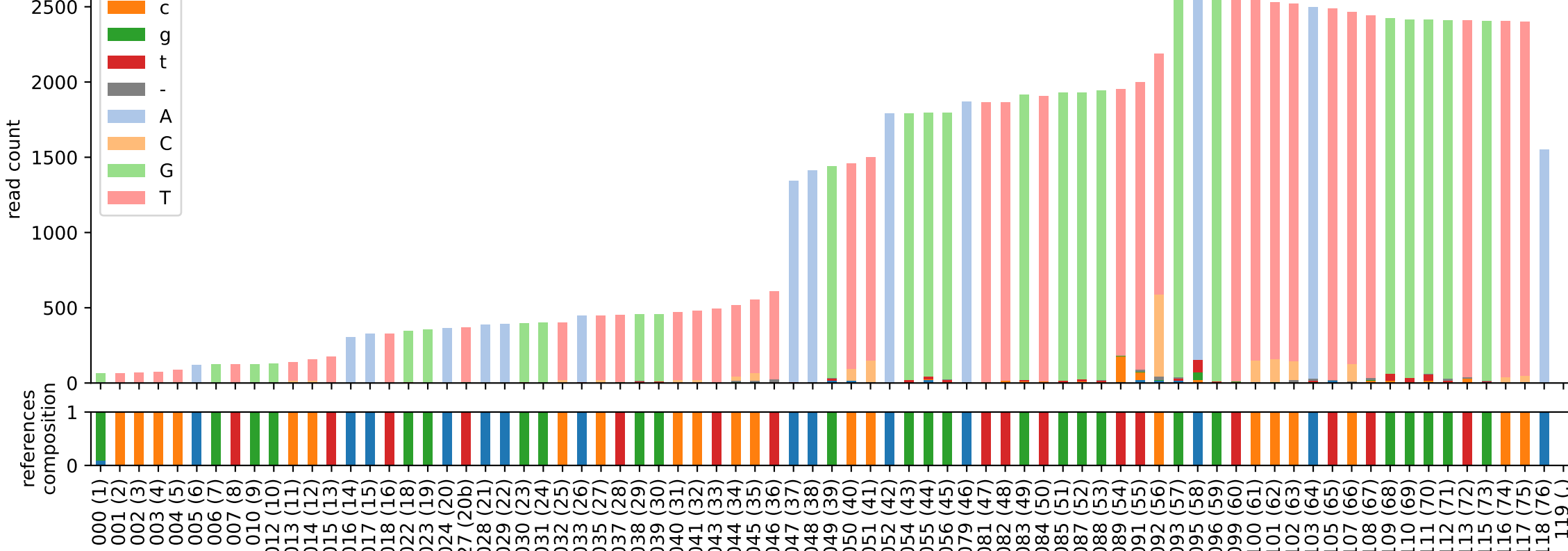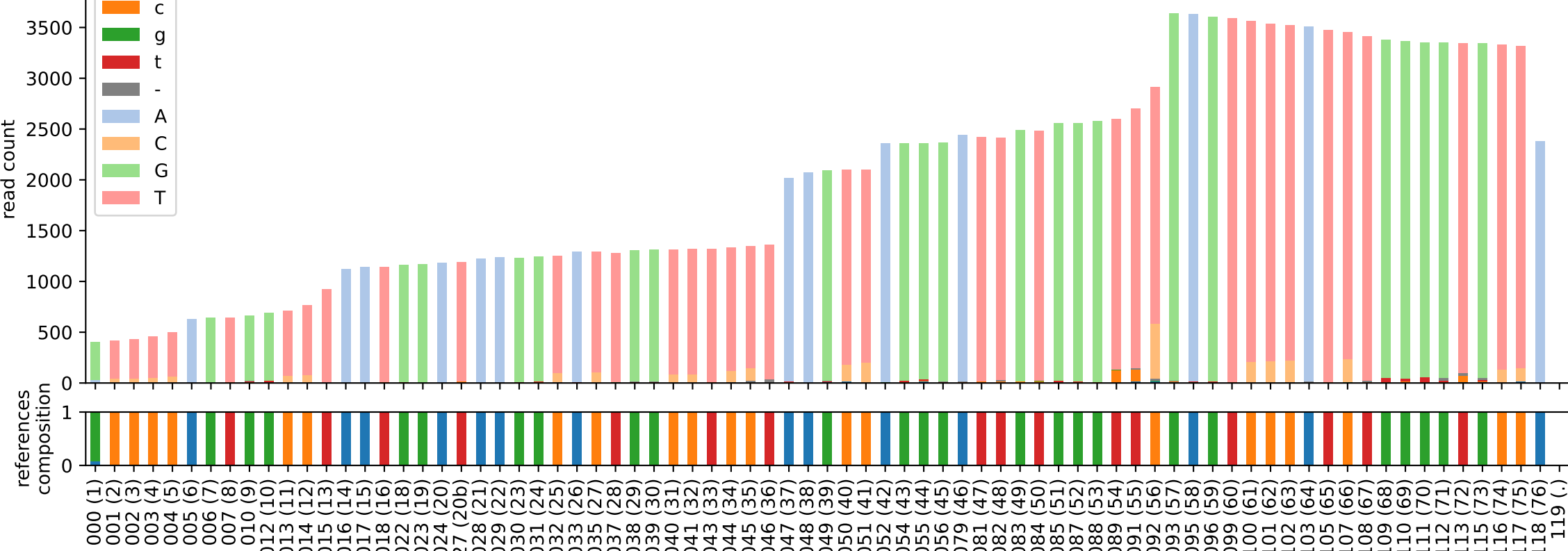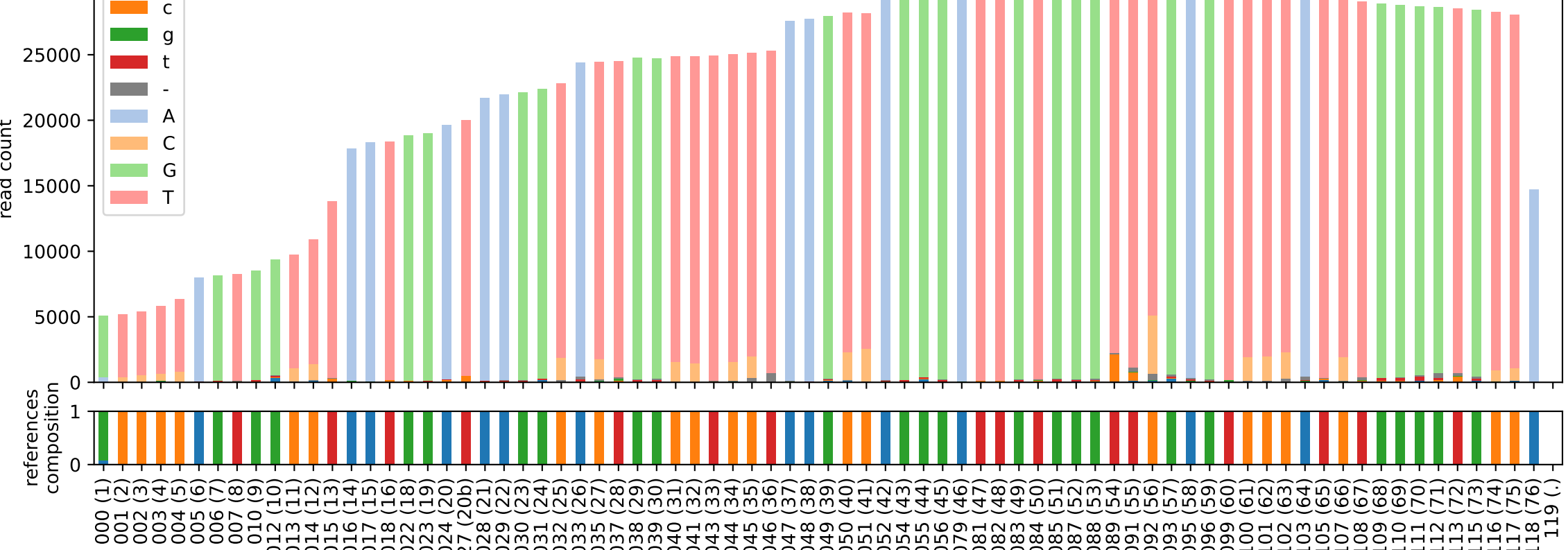

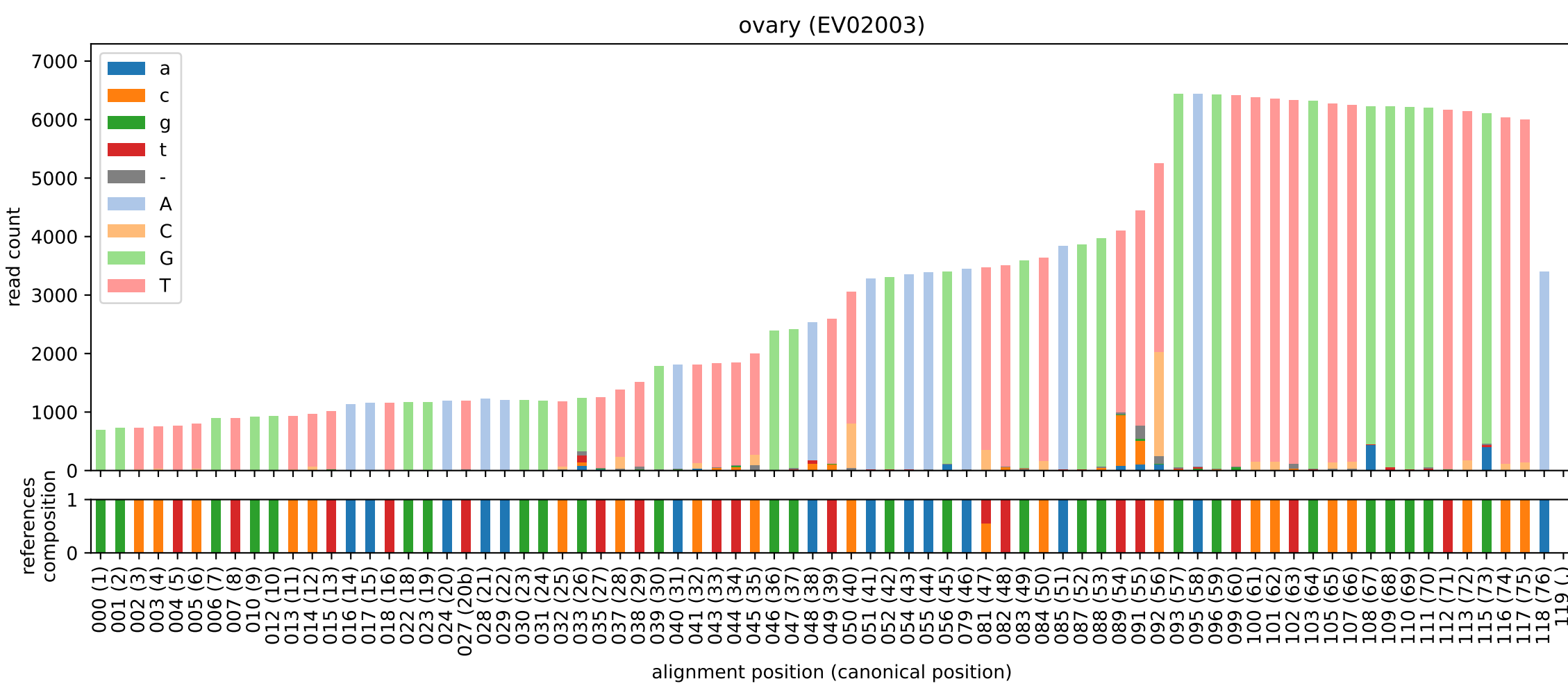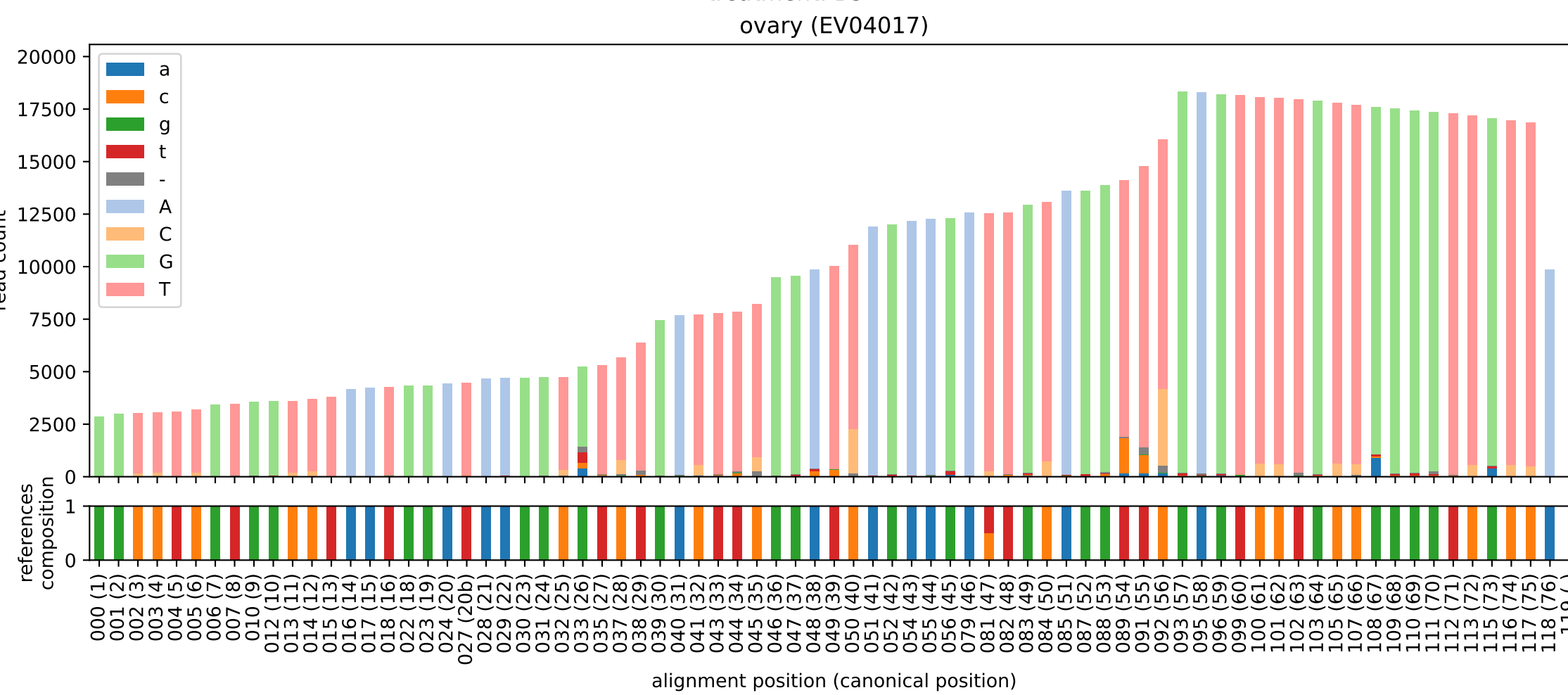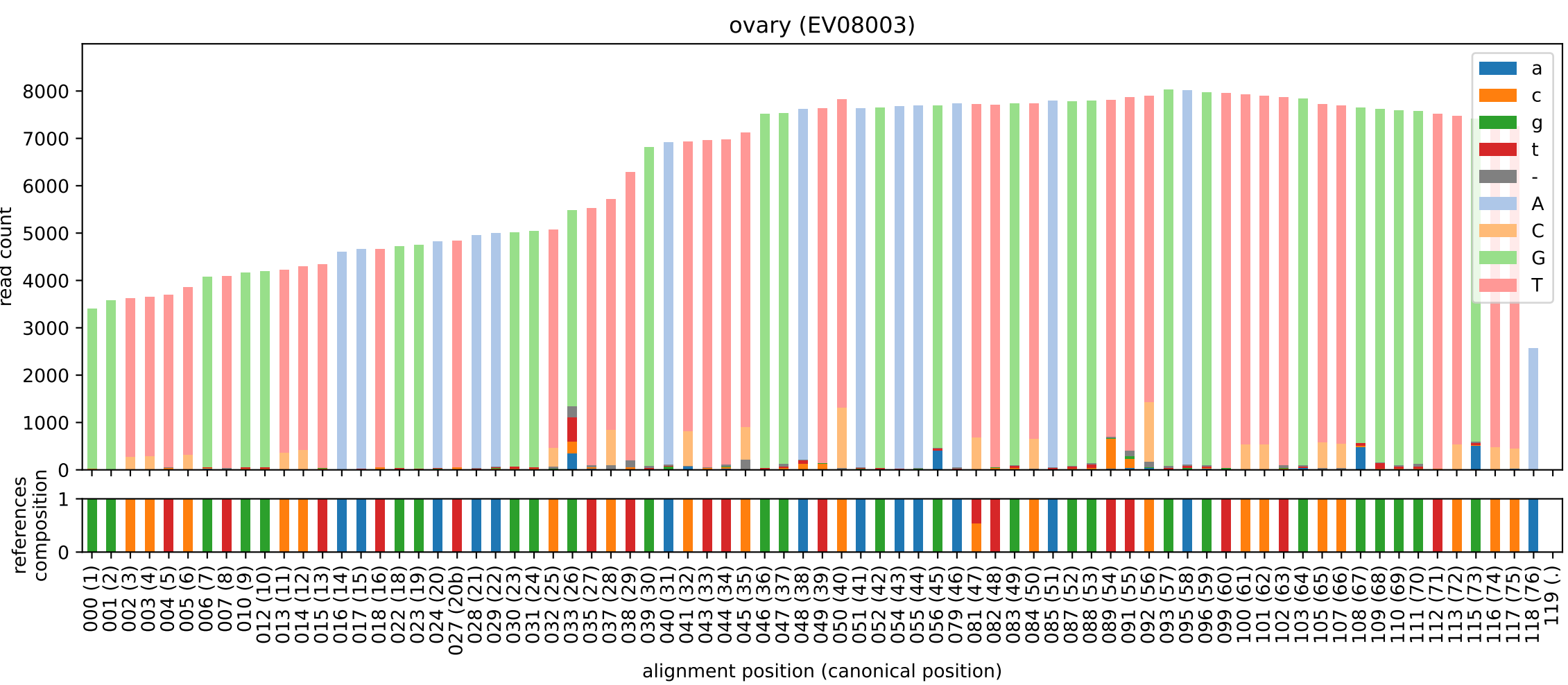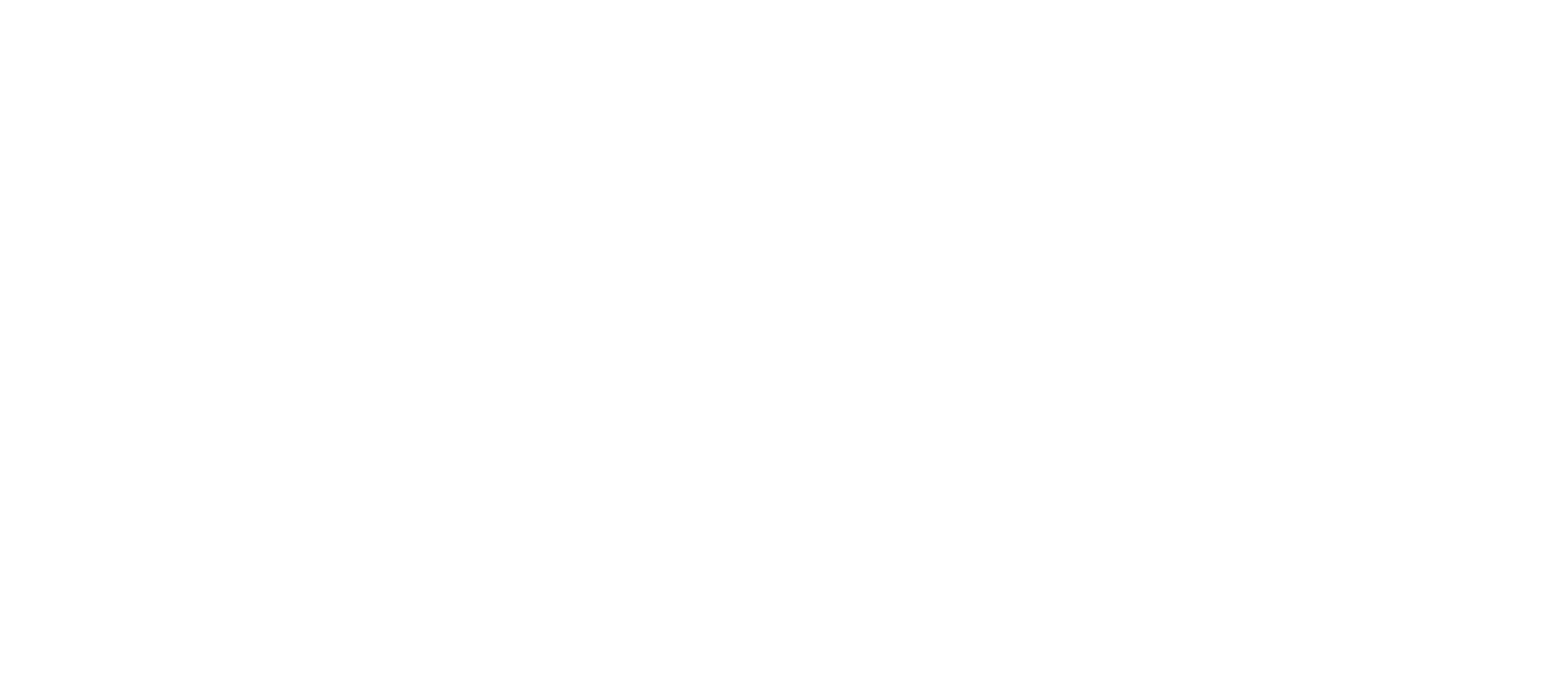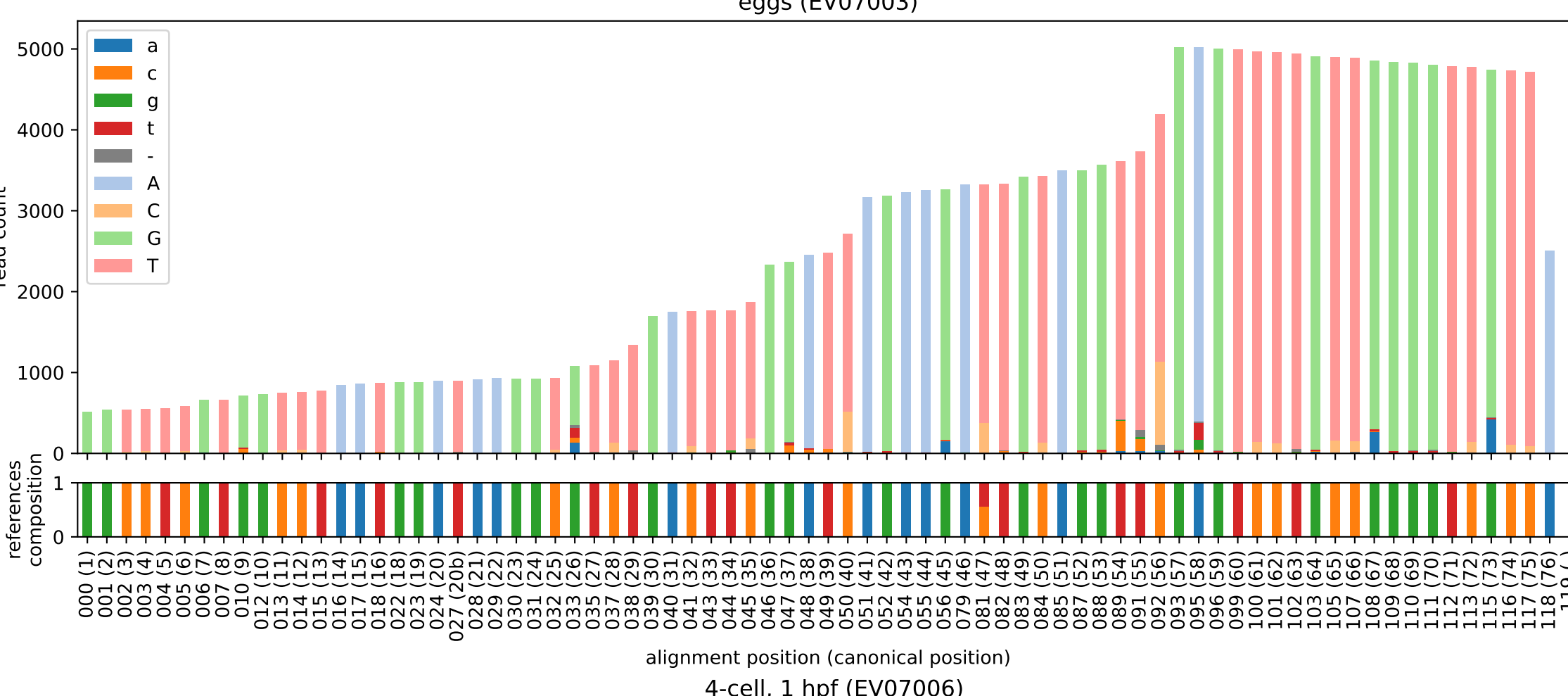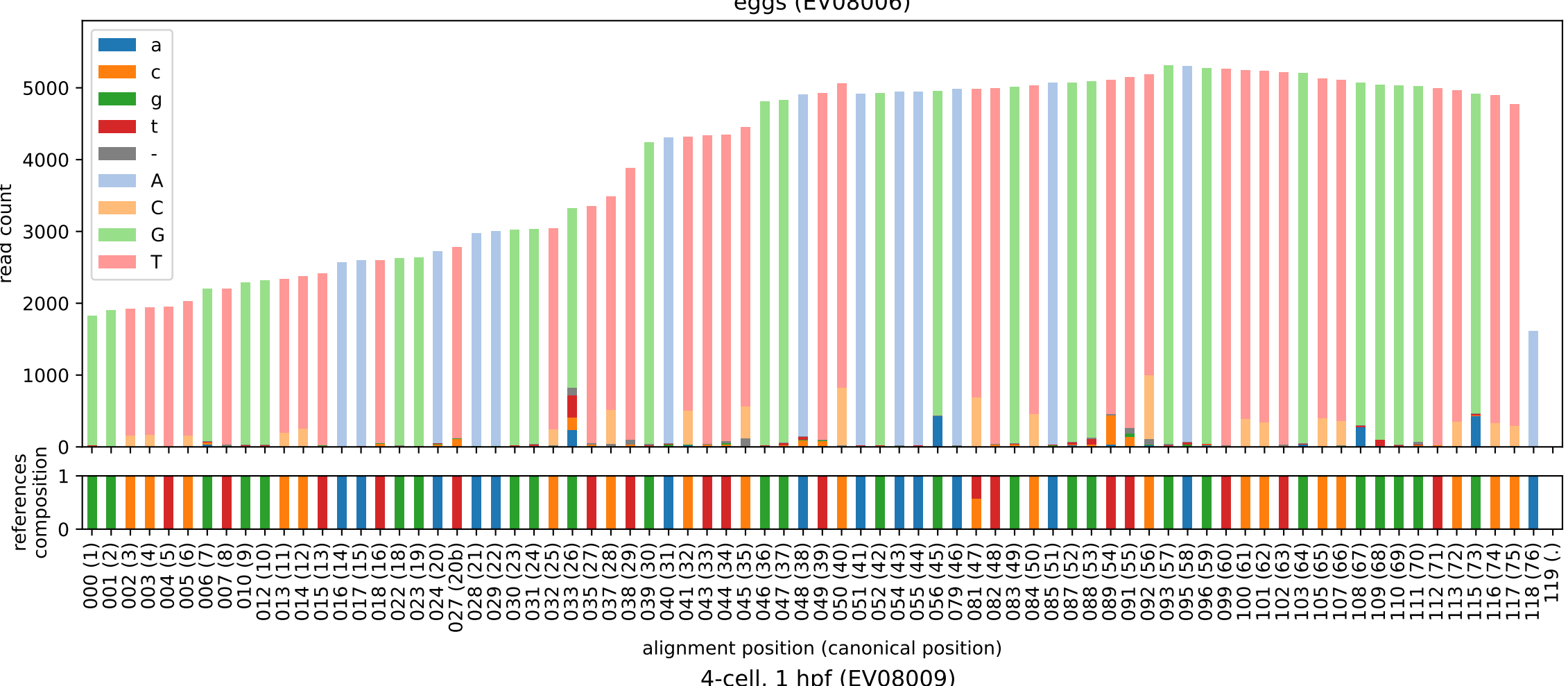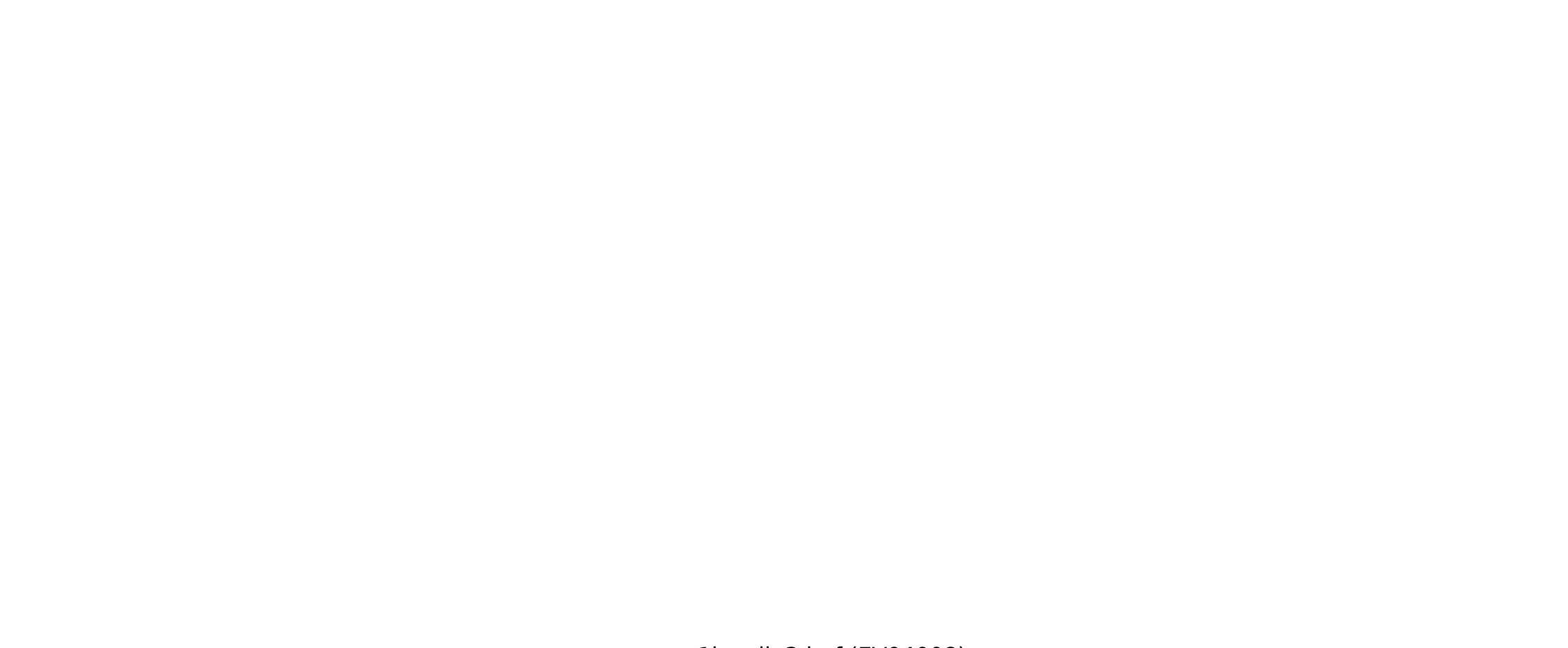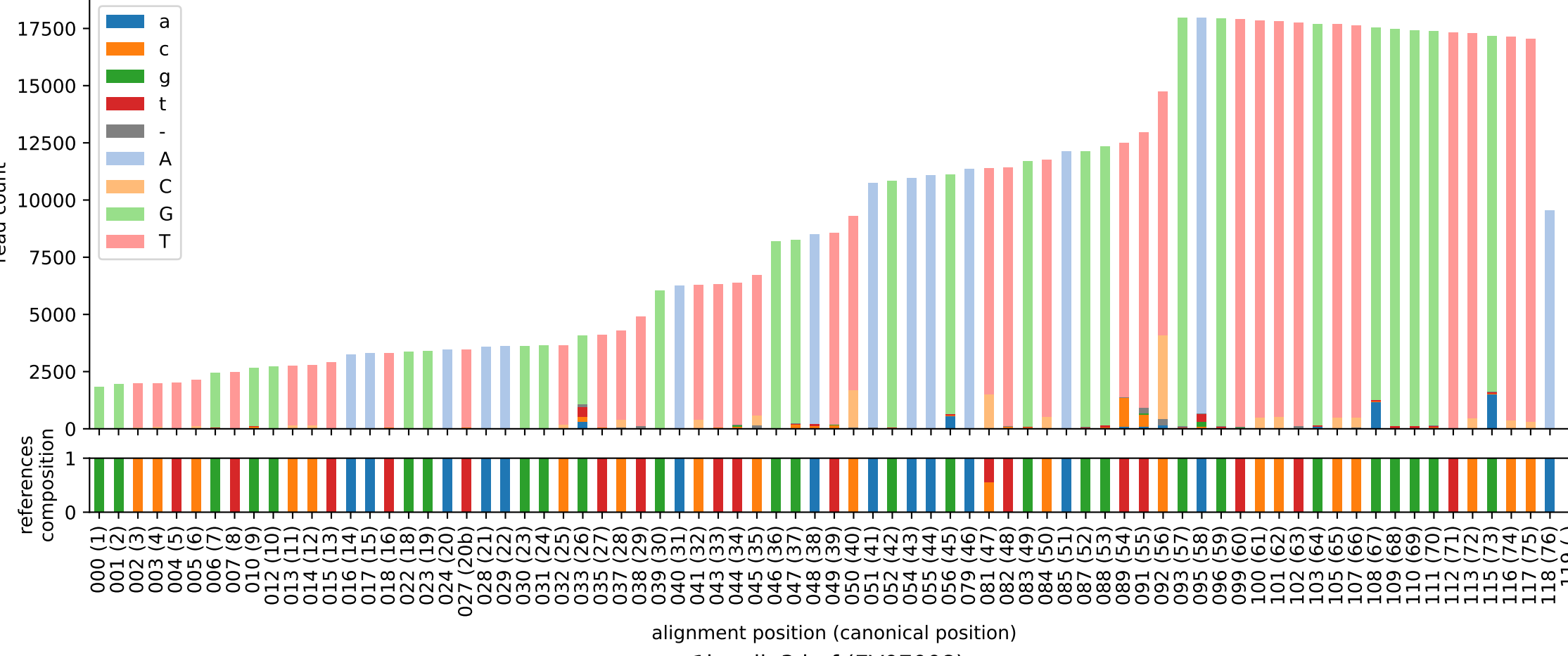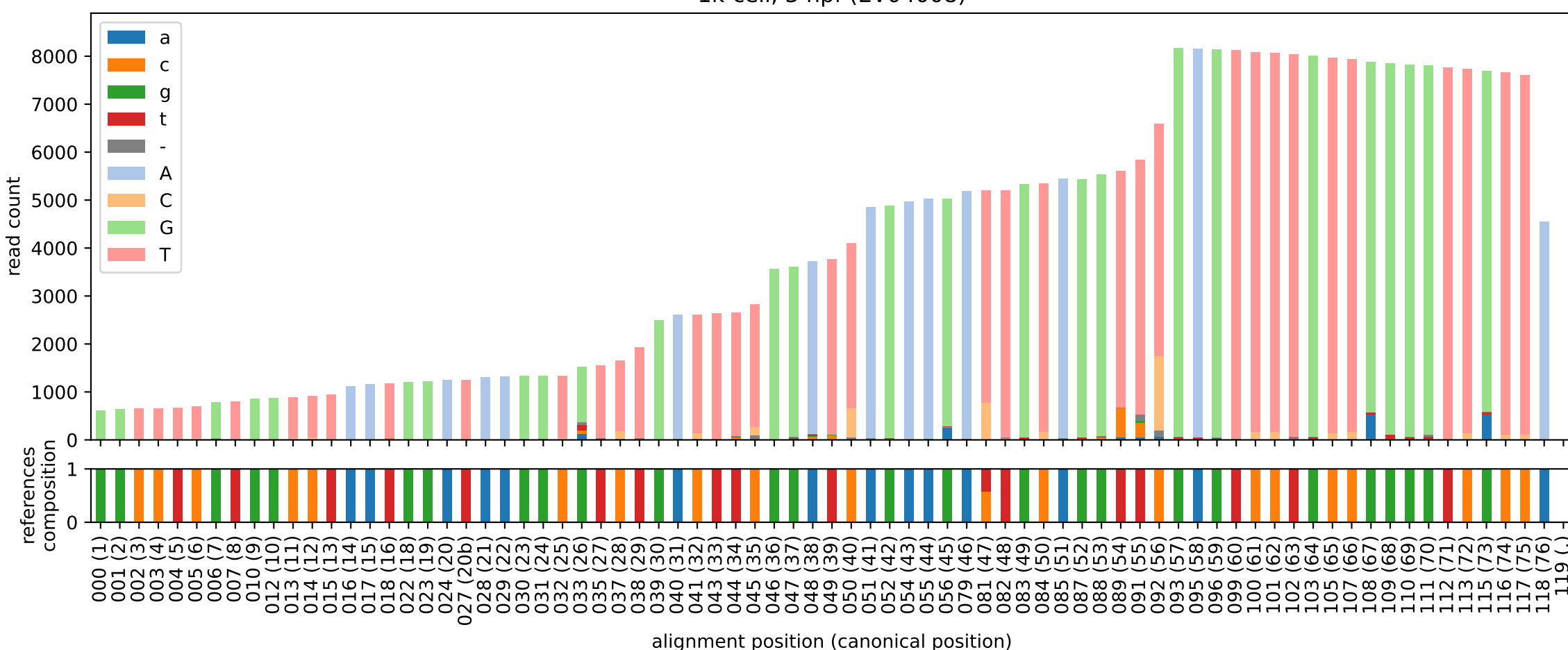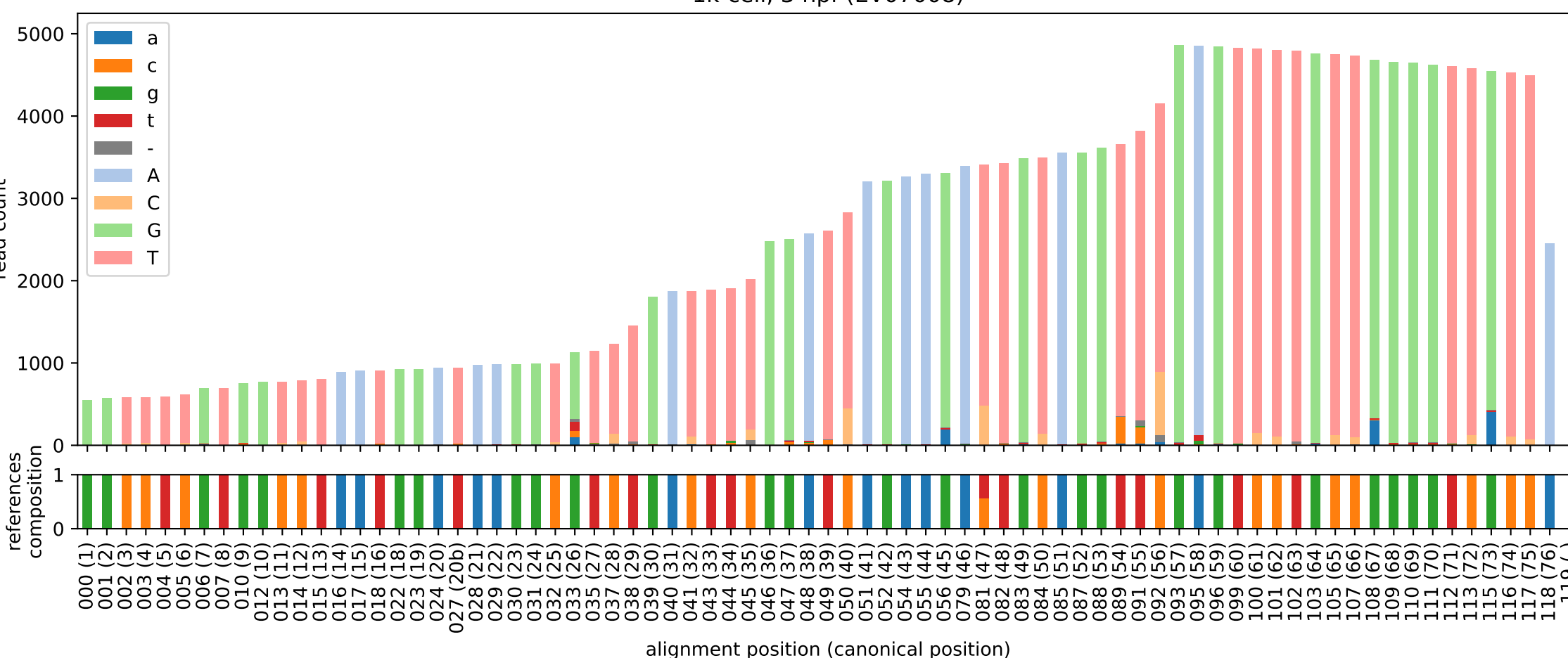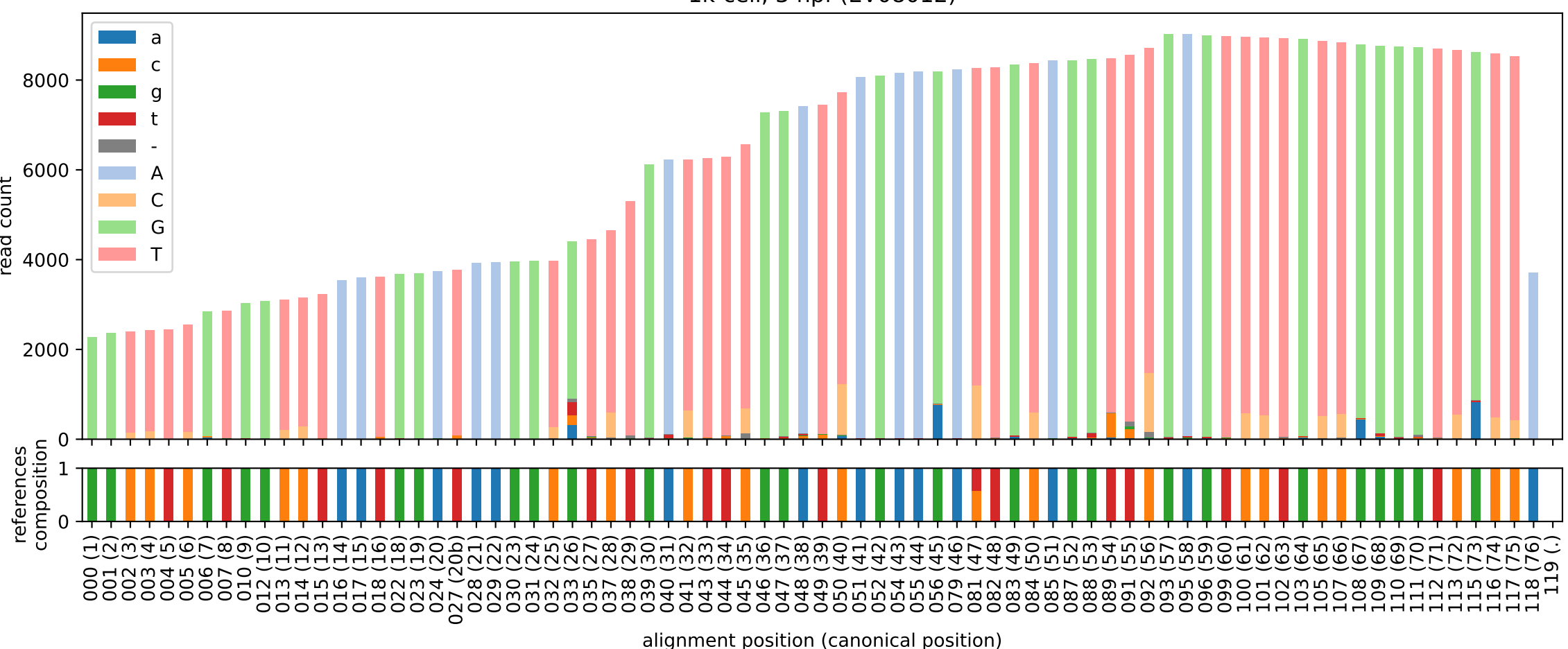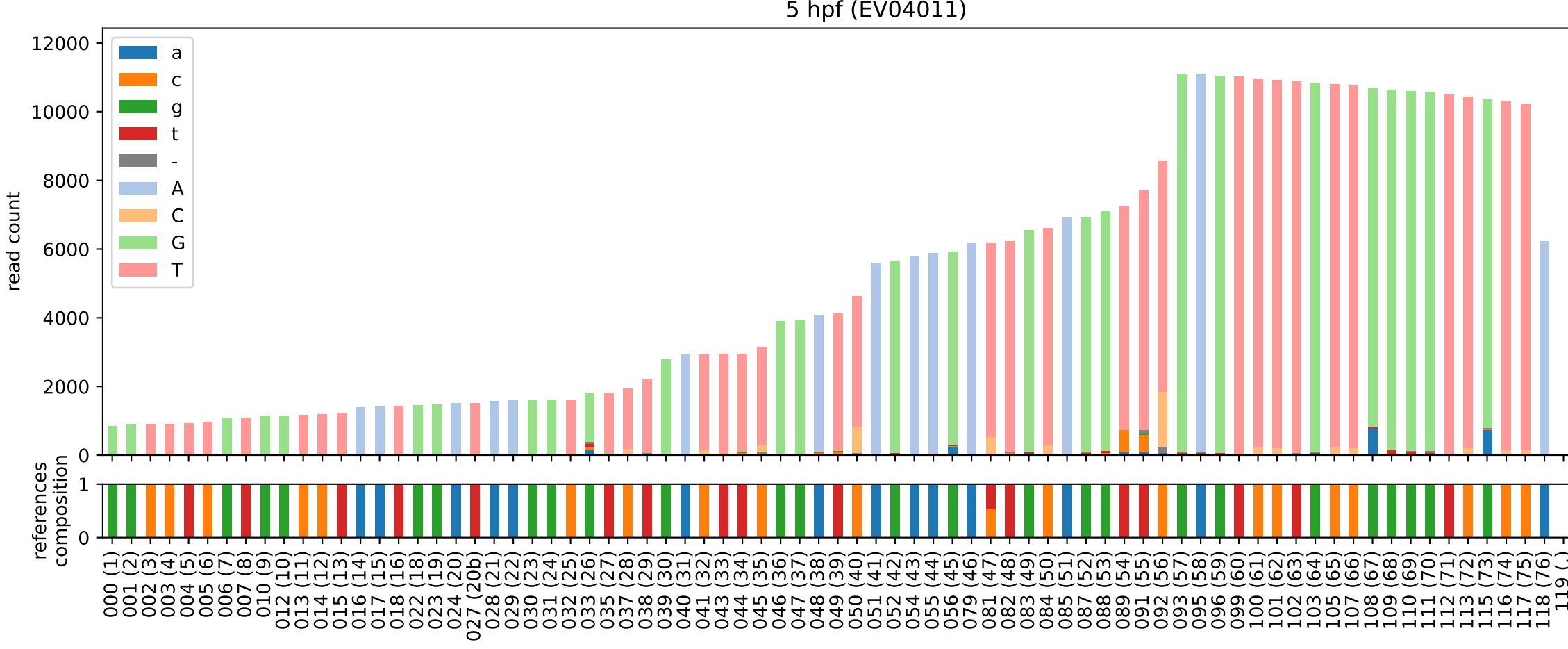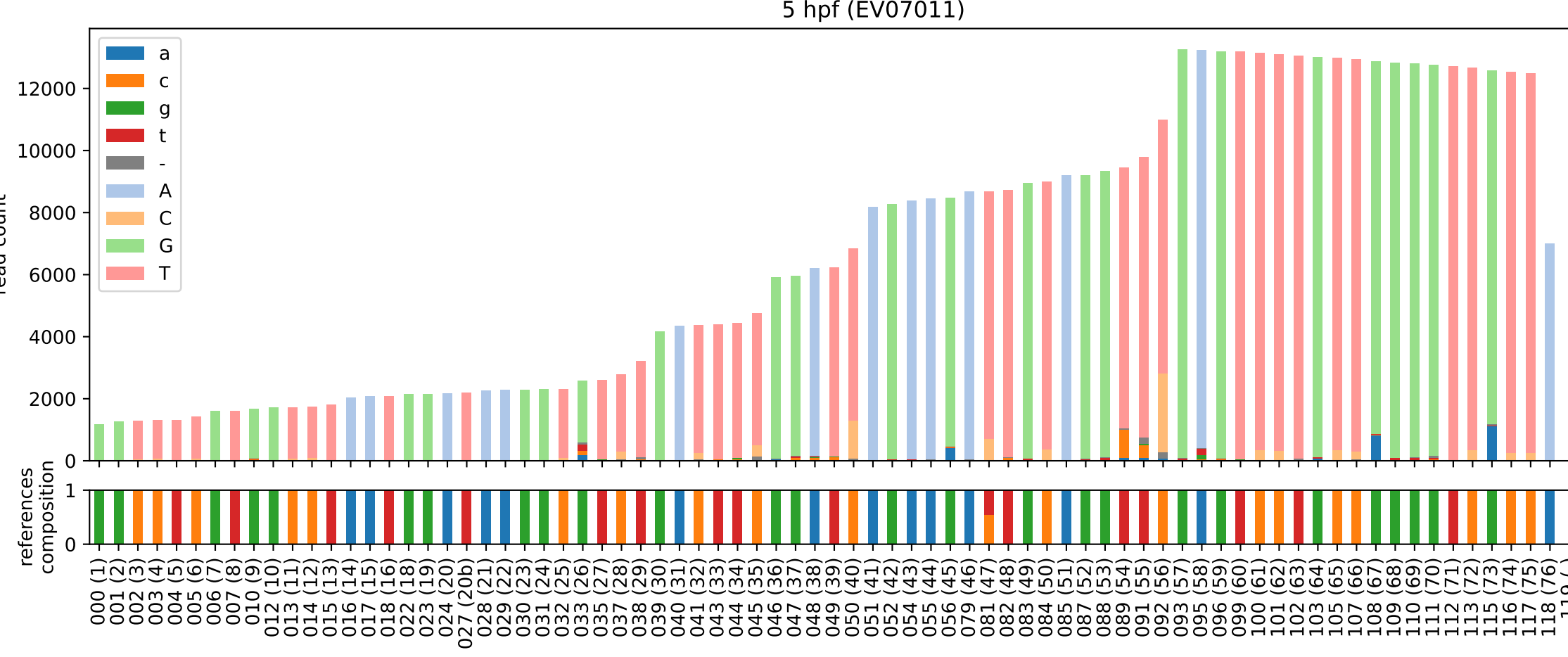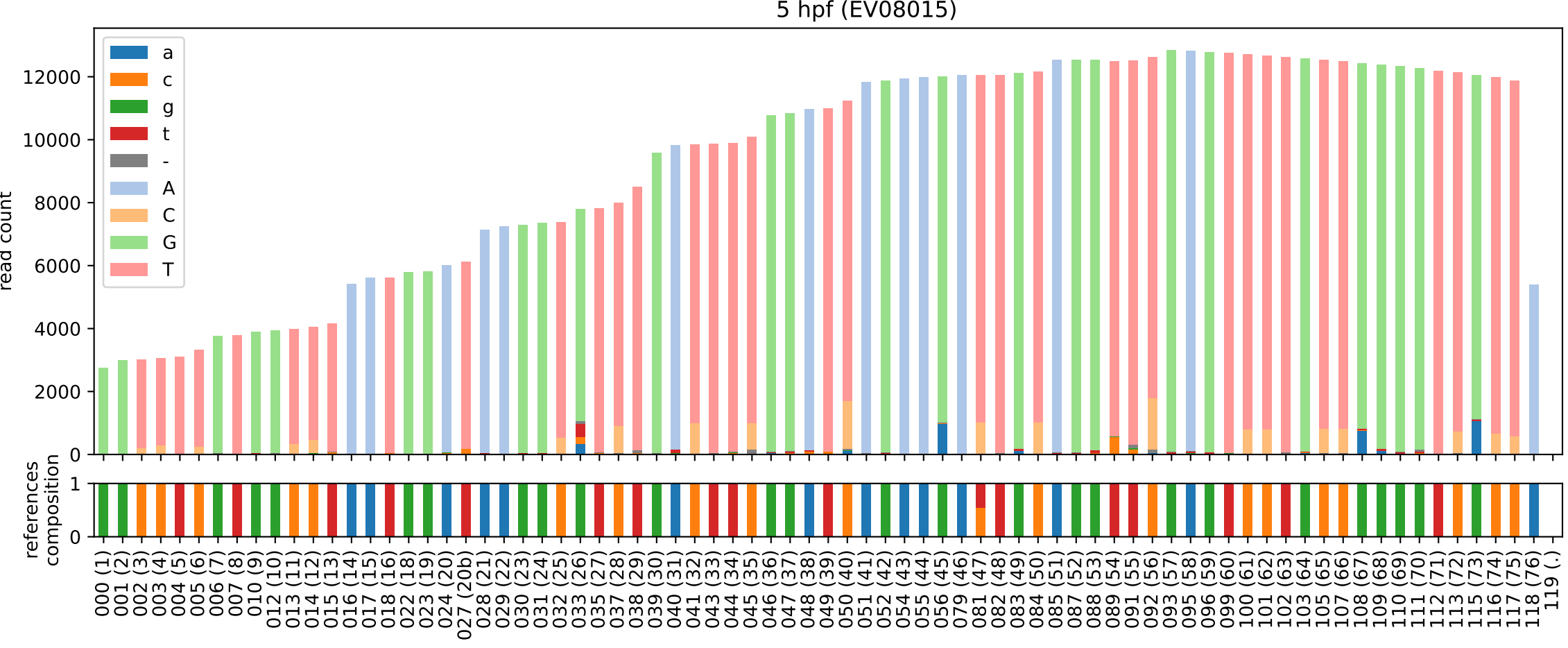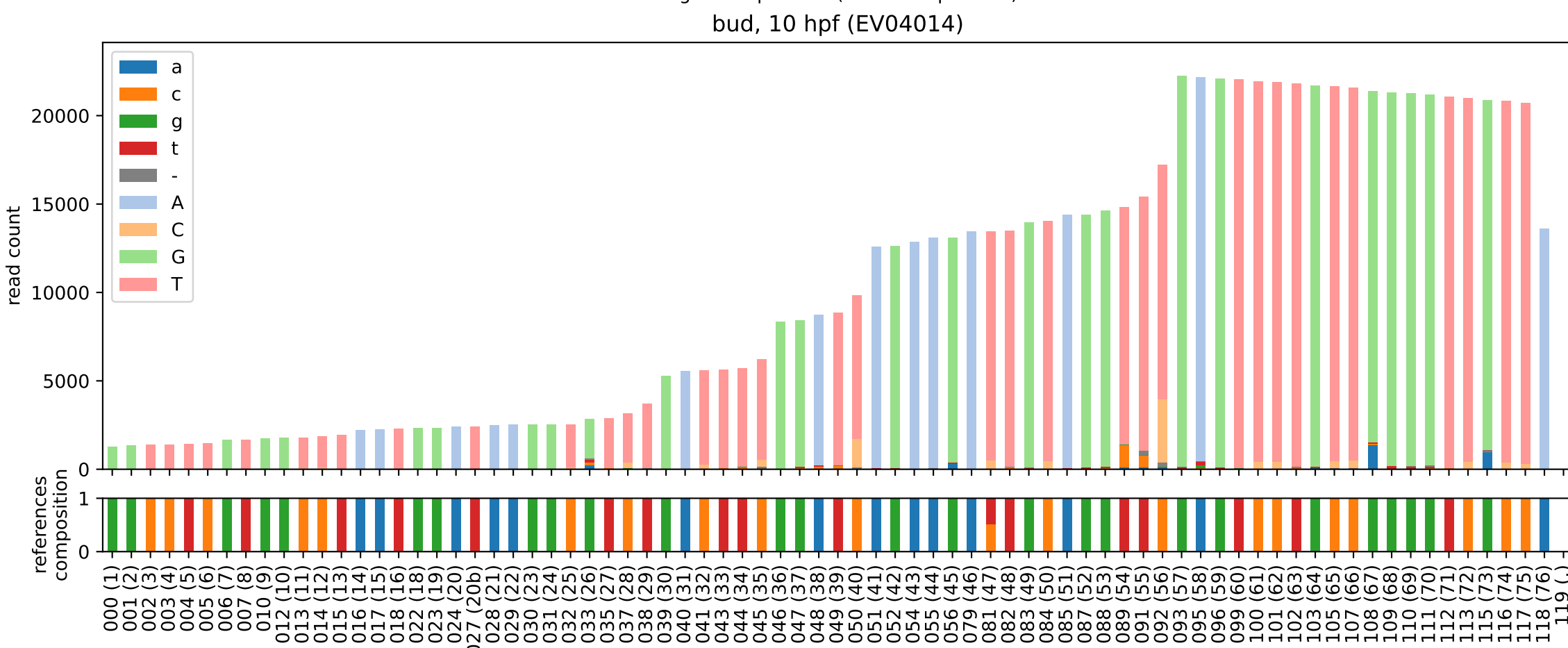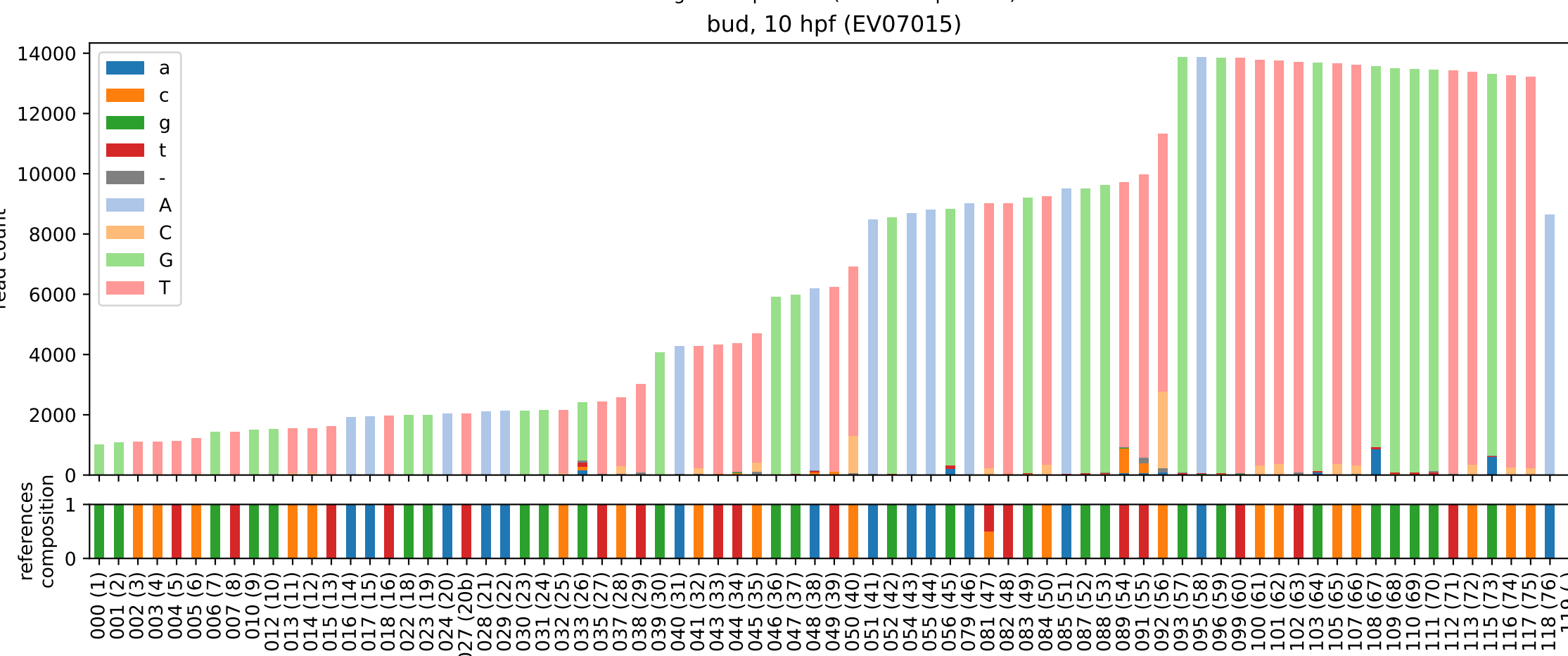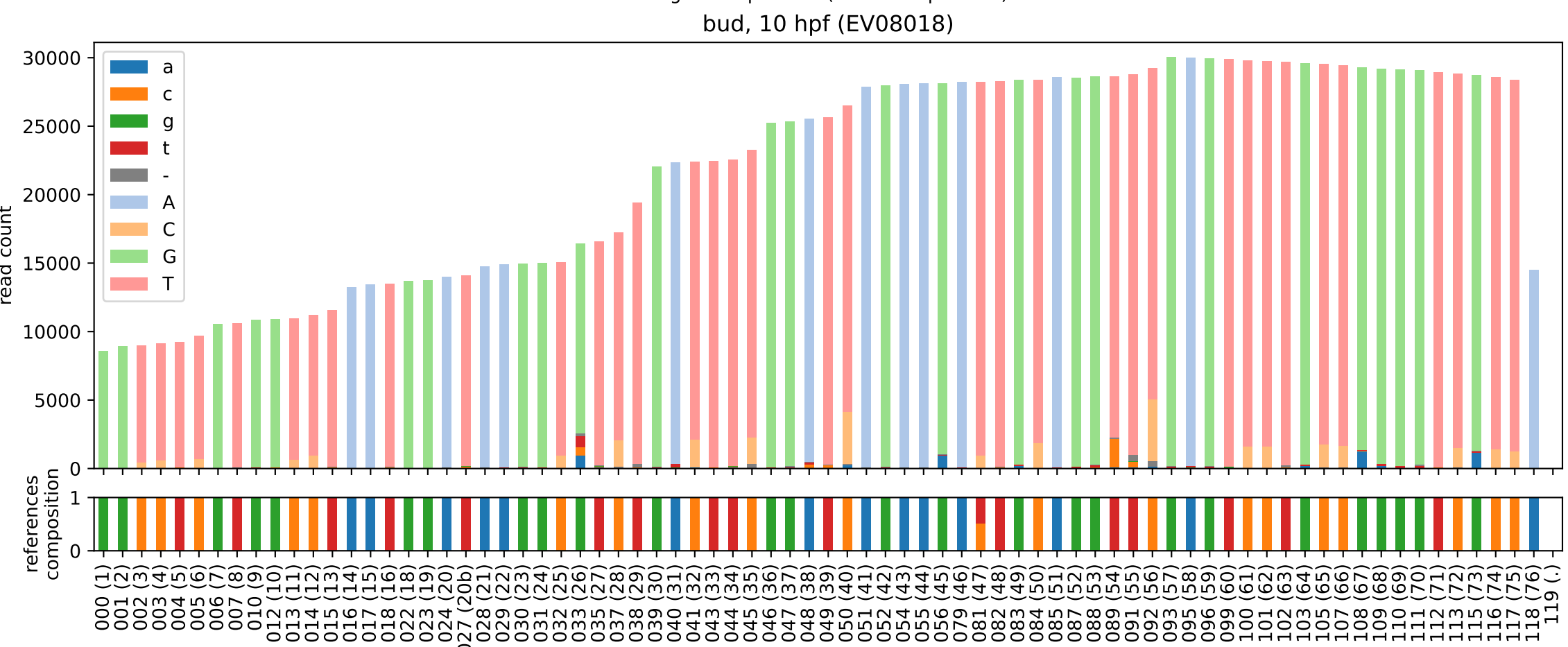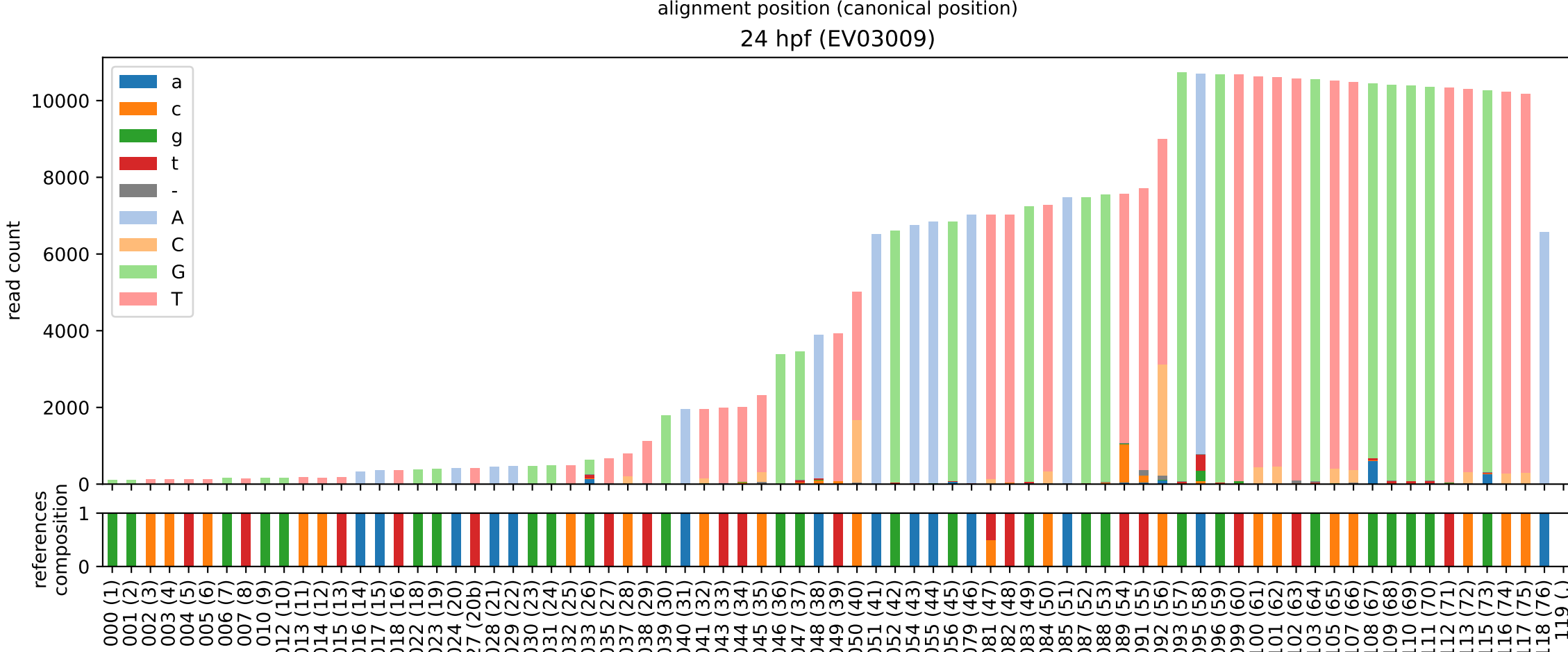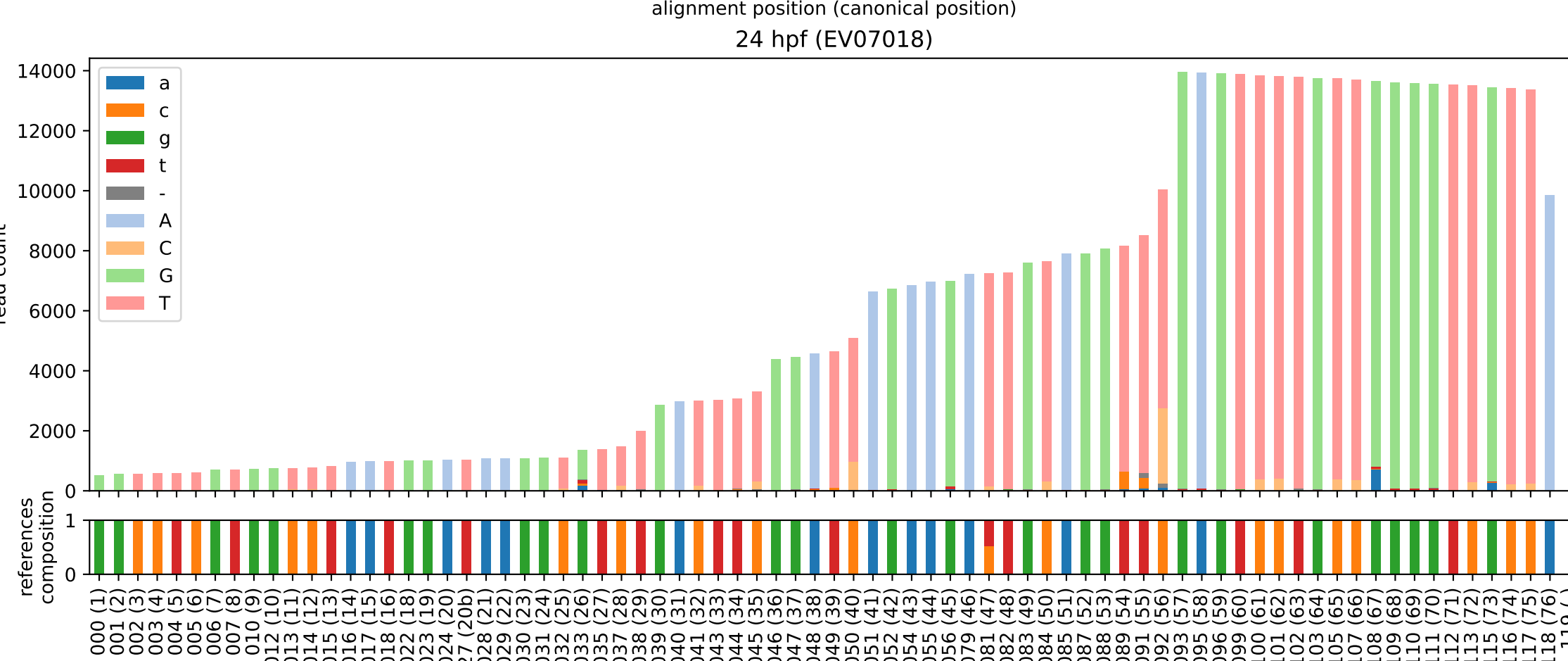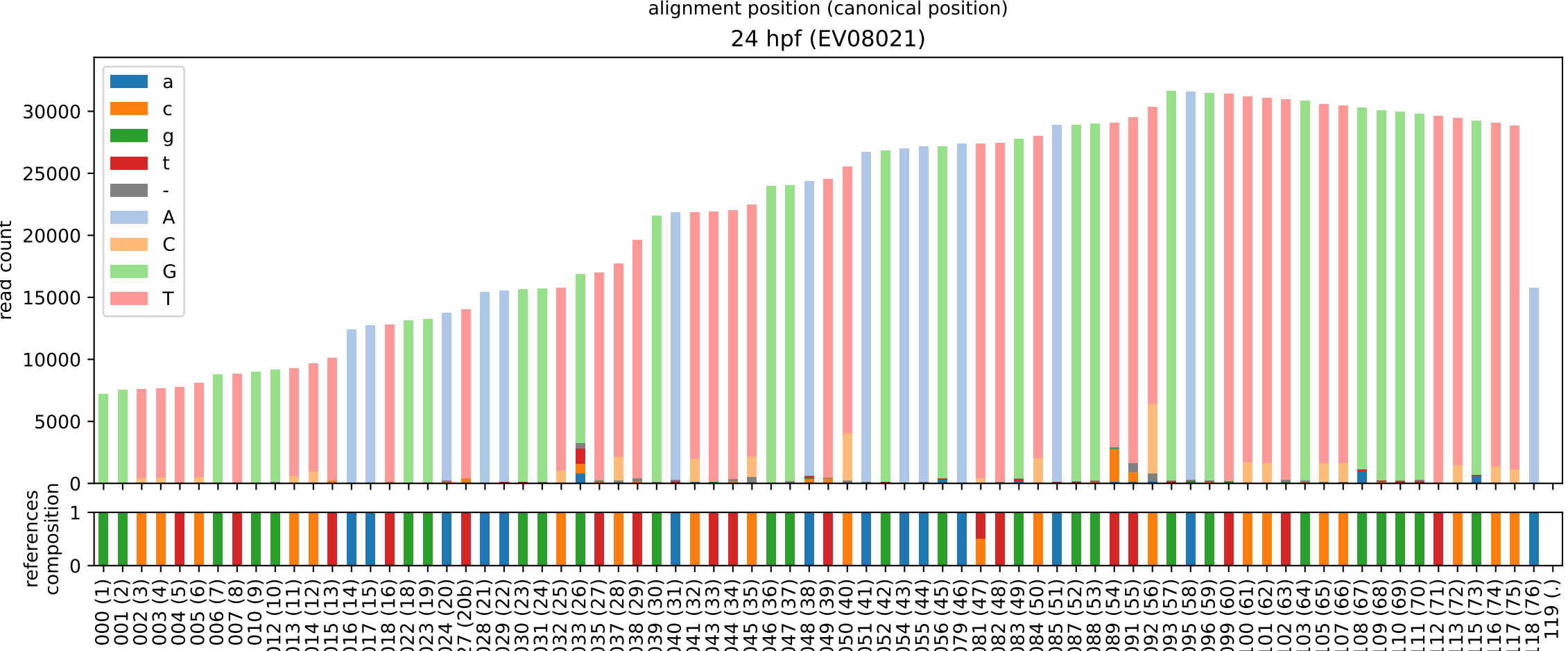

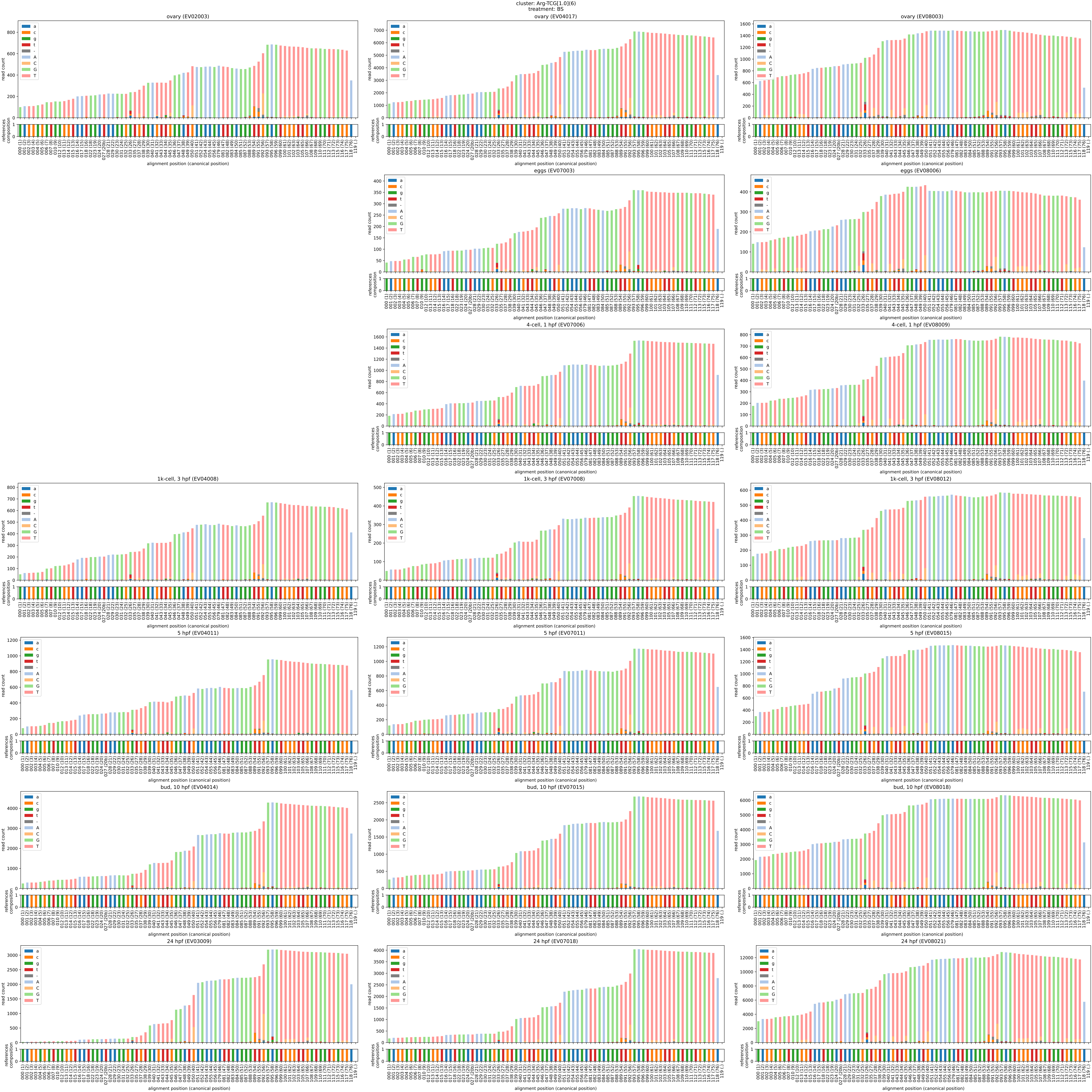



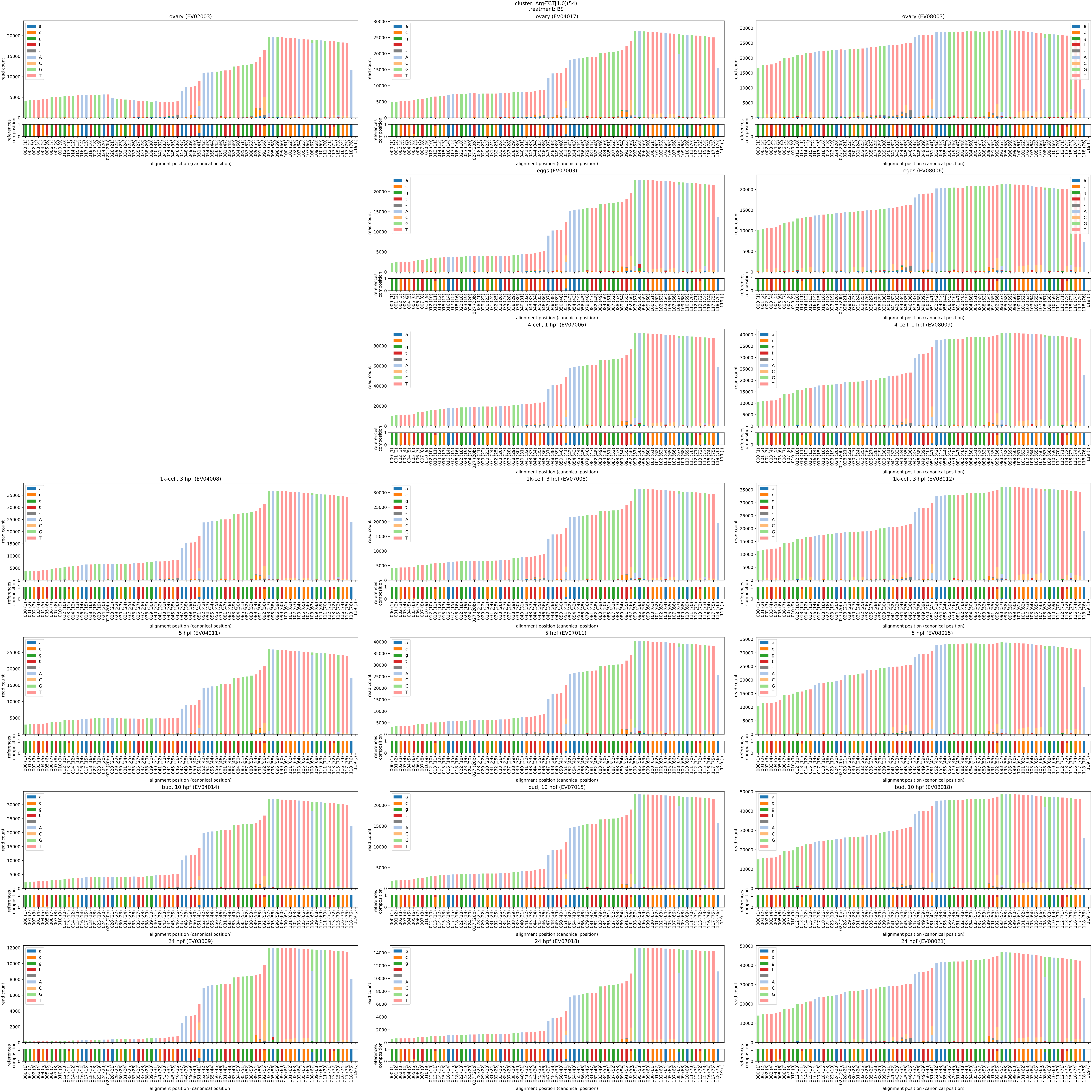



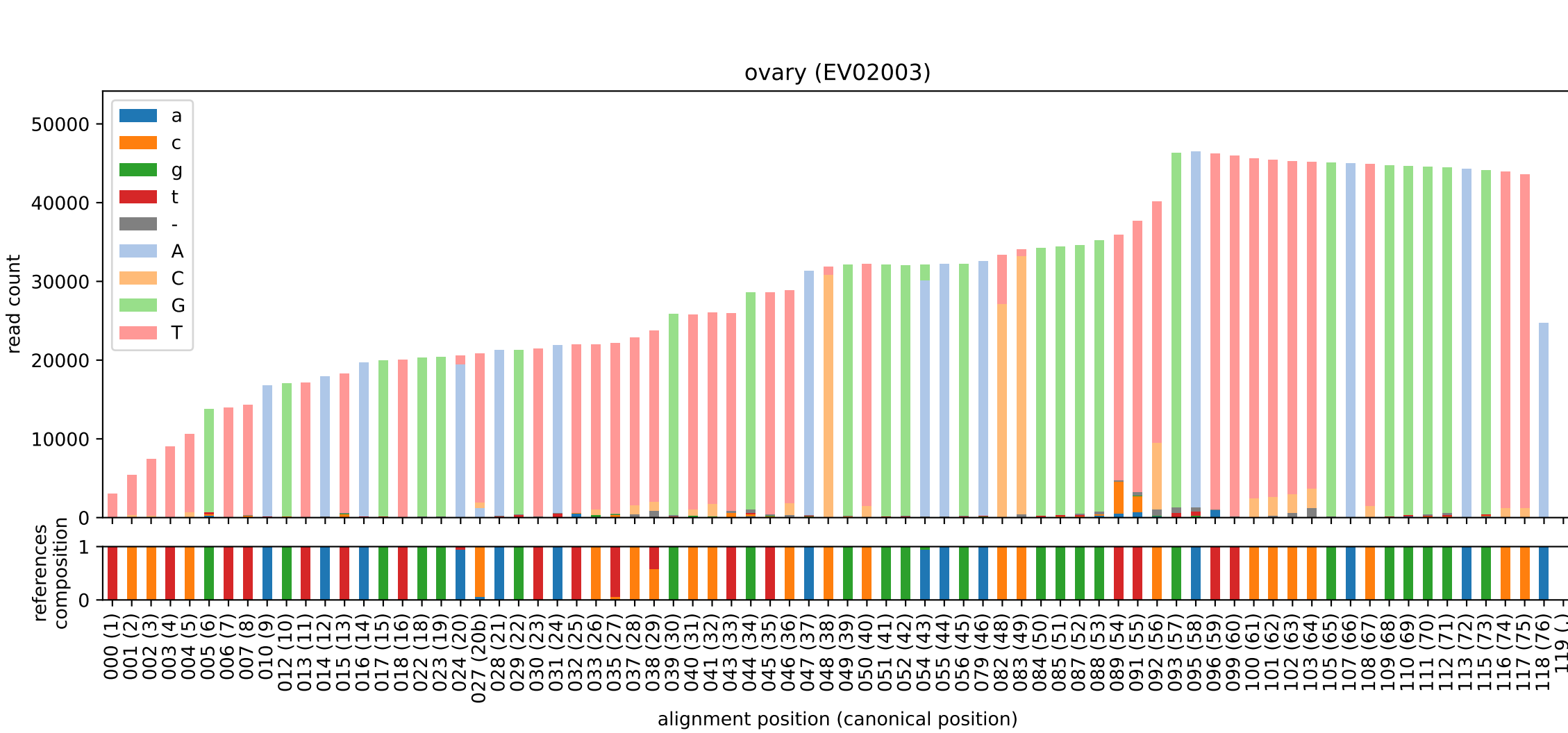

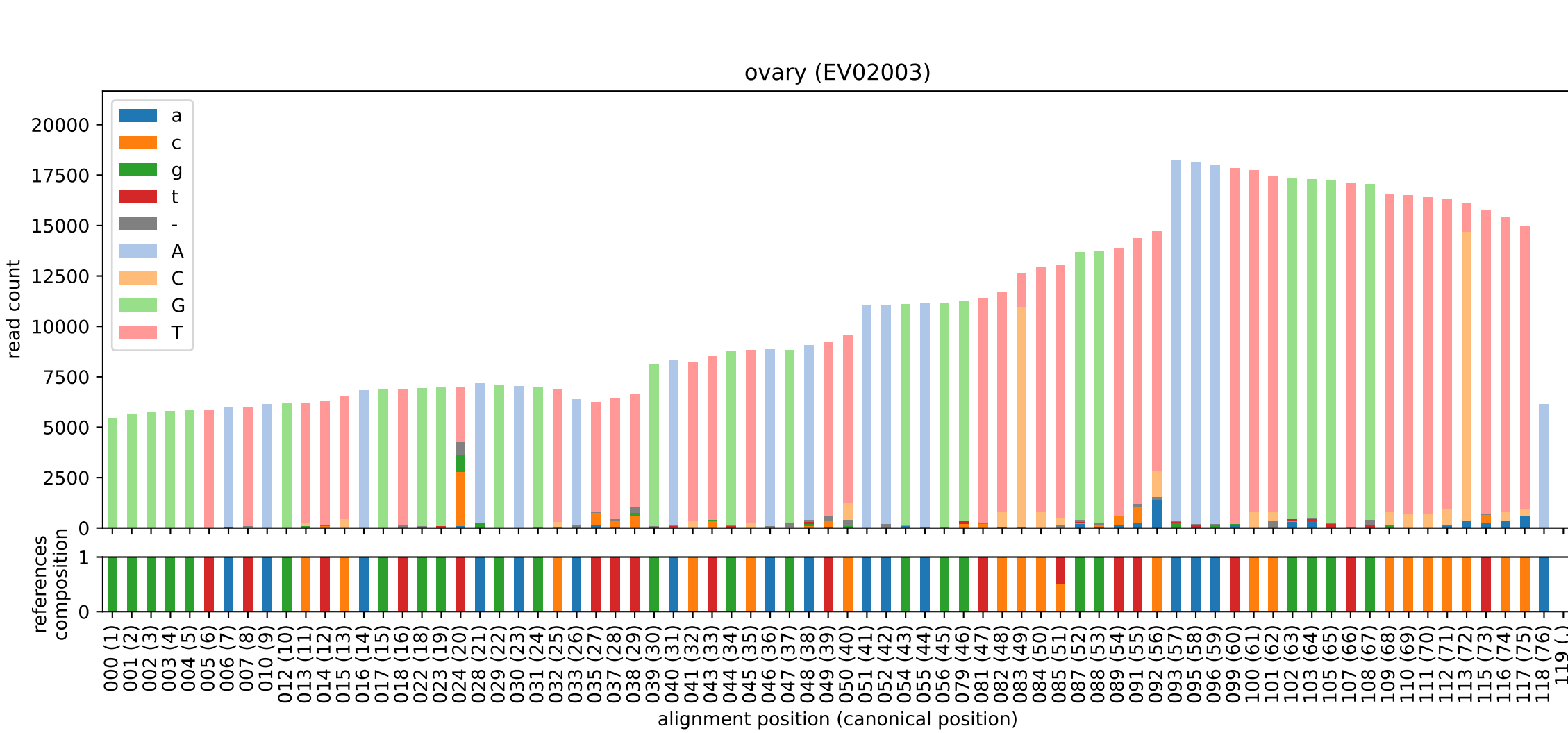









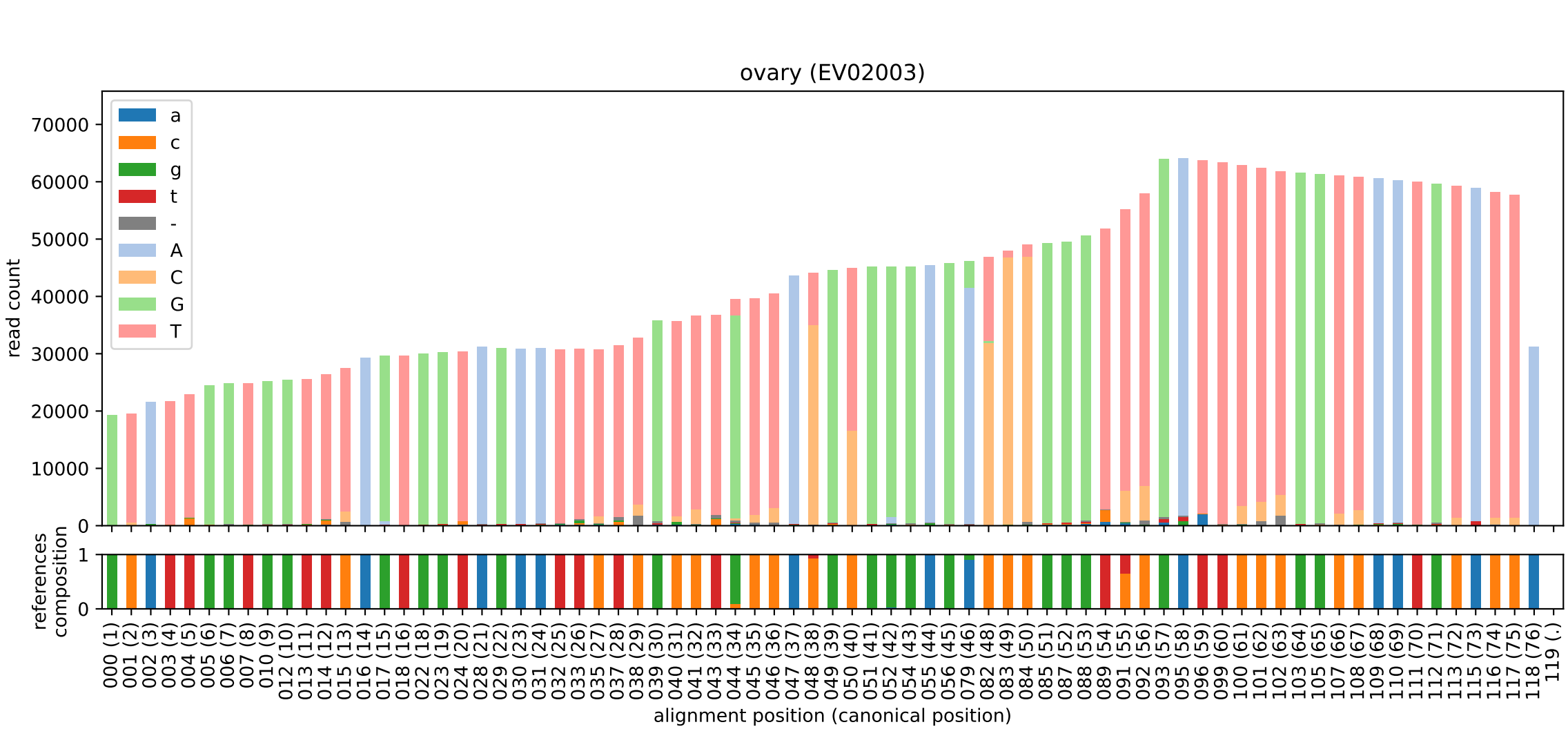







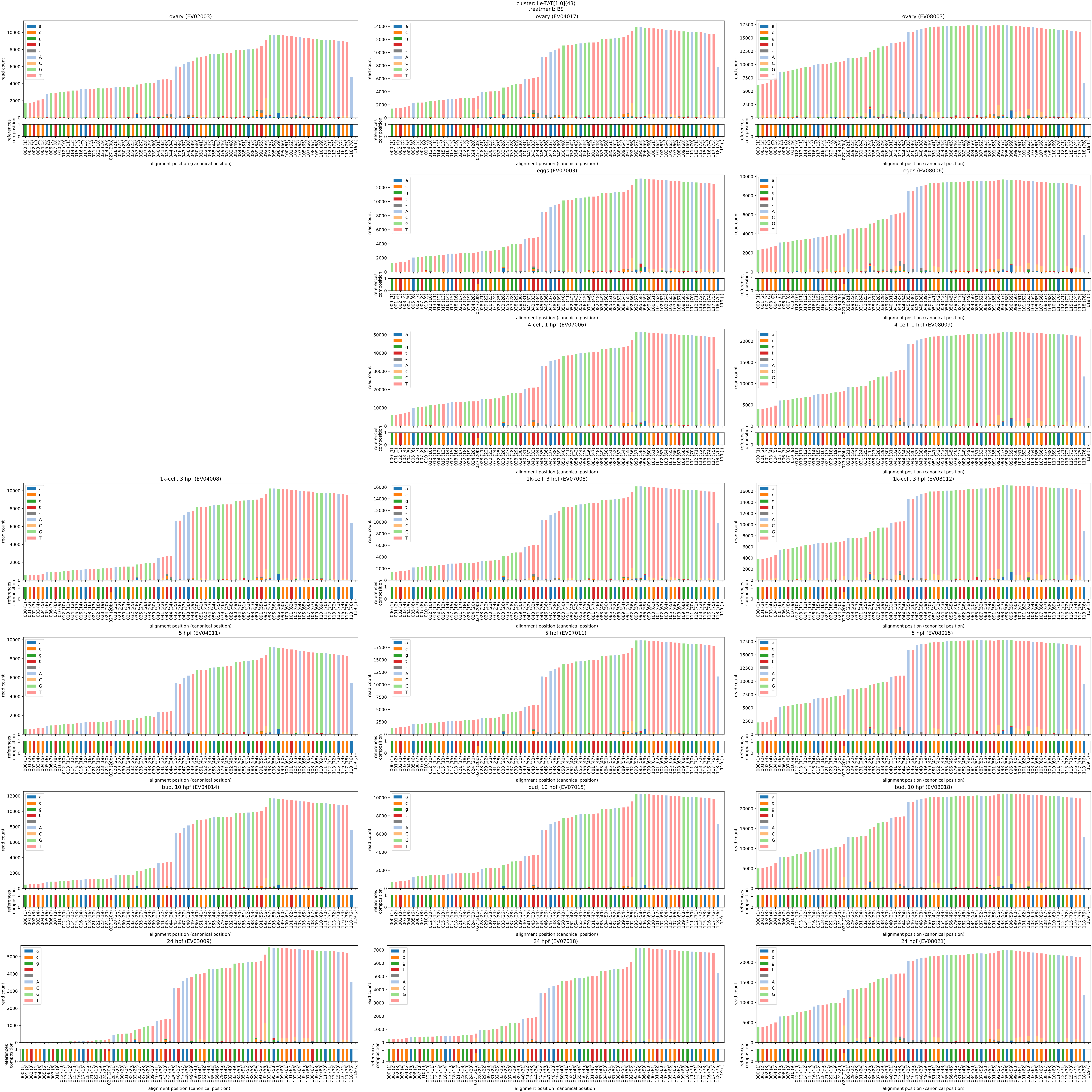

Supplement: gkae595_Supplemental_Files [file gkae595_supplemental_files.zip › Supplementary_file_4-coverage_plots_all-BS-v3.pdf]
