# Supplementary material for: tRNA expression and modification landscapes, and their dynamics during zebrafish embryo development
Source: Nucleic Acids Res. 2024 Jul 11;52(17):10575–94. doi: 10.1093/nar/gkae595 (PMC11417395; doi:10.1093/nar/gkae595)
Supplement: gkae595_Supplemental_Files [file Supplementary_file_2-coverage_plots_all-mock-v3.pdf › Supplementary_file_2-coverage_plots_all-mock-v3.pdf]

cluster: Ala-AGC[1,0](58)
treatment: MOCK

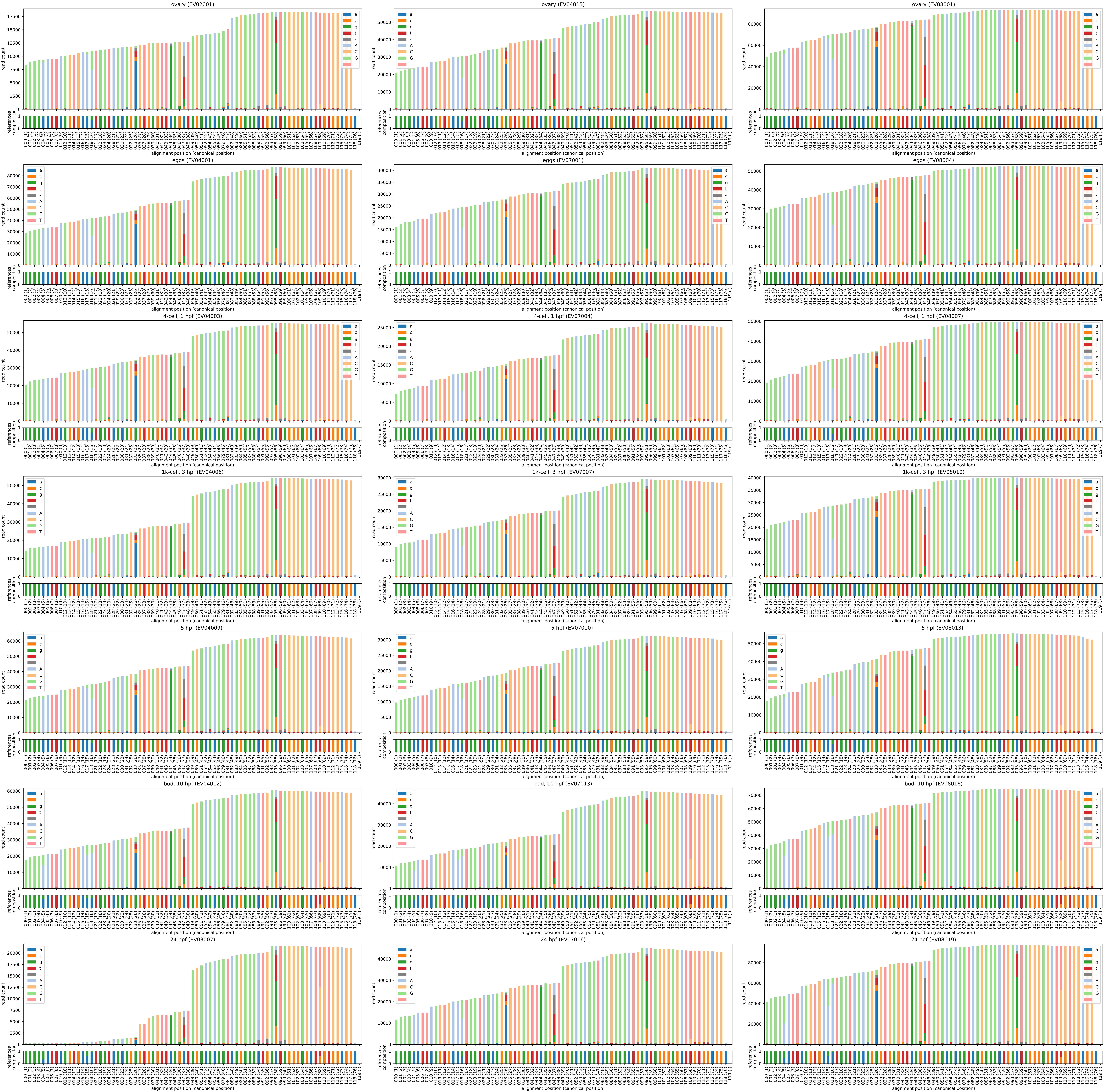

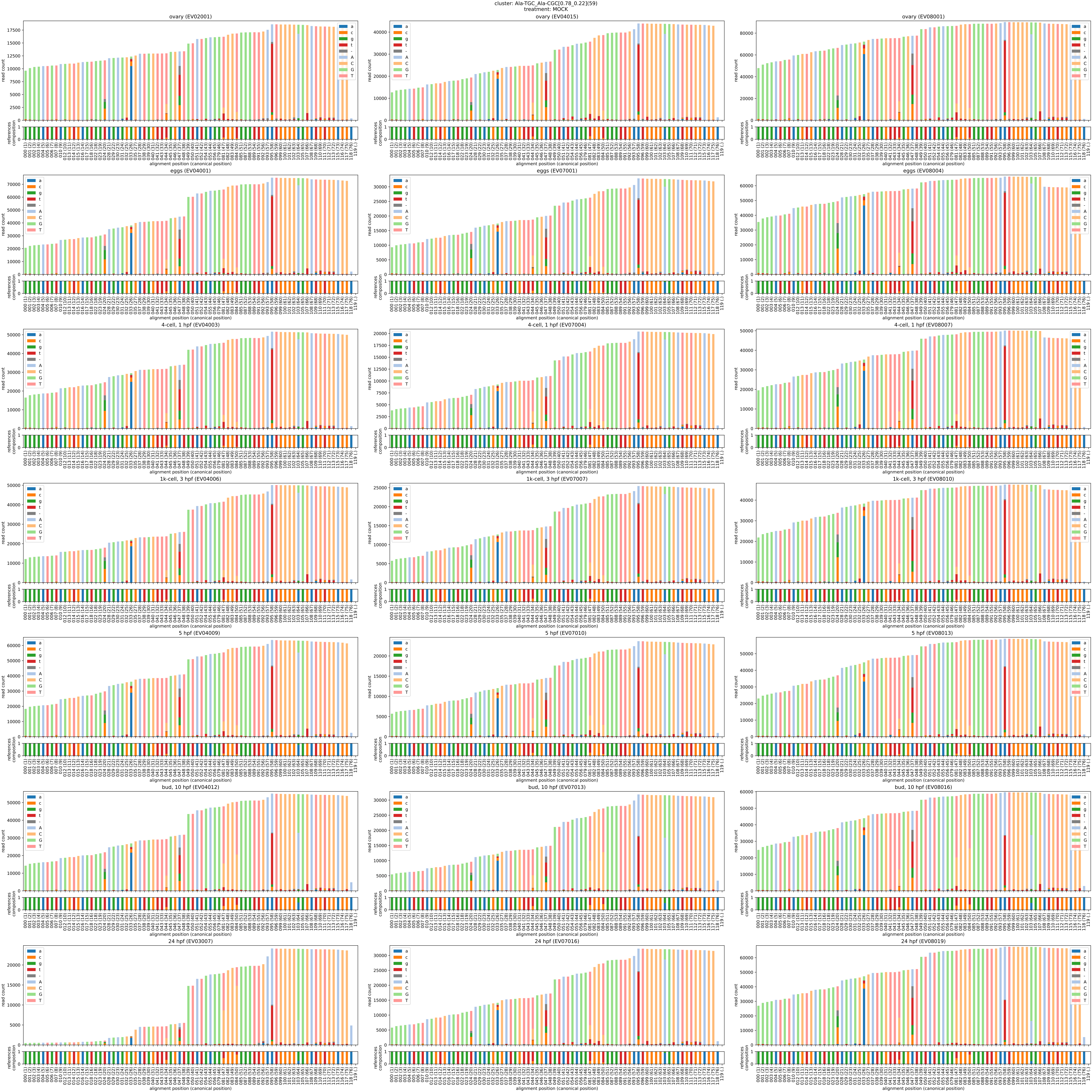

cluster: Ala-TGC_Ala-CGC[0.78_0.22](59)
treatment: MOCK
ovary (EV02001)
ovary (EV04015)
ovary (EV08001)
eggs (EV04001)
eggs (EV07001)
eggs (EV08004)
4-cell, 1 hpf (EV04003)
4-cell, 1 hpf (EV07004)
4-cell, 1 hpf (EV08007)
1k-cell, 3 hpf (EV04006)
1k-cell, 3 hpf (EV07007)
1k-cell, 3 hpf (EV08010)
5 hpf (EV04009)
5 hpf (EV07010)
5 hpf (EV08013)
bud, 10 hpf (EV04012)
bud, 10 hpf (EV07013)
bud, 10 hpf (EV08016)
24 hpf (EV03007)
24 hpf (EV07016)
24 hpf (EV08019)
read count
references composition
alignment position (canonical position)
a
c
g
t
-
A
C
G
T

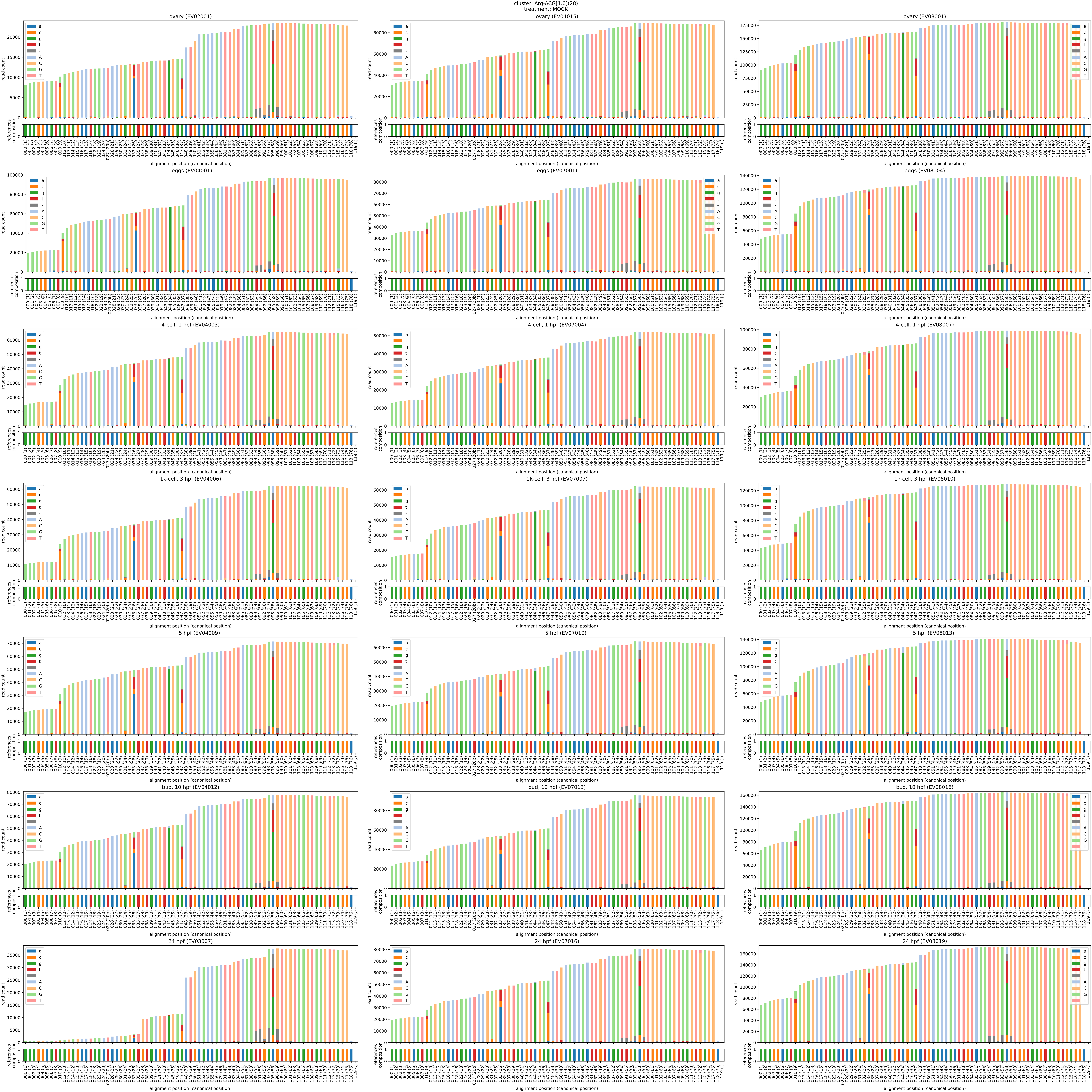

cluster: Arg-ACG[1.0](28)
treatment: MOCK
ovary (EV02001)
ovary (EV04015)
ovary (EV08001)
eggs (EV04001)
eggs (EV07001)
eggs (EV08004)
4-cell, 1 hpf (EV04003)
4-cell, 1 hpf (EV07004)
4-cell, 1 hpf (EV08007)
1k-cell, 3 hpf (EV04006)
1k-cell, 3 hpf (EV07007)
1k-cell, 3 hpf (EV08010)
5 hpf (EV04009)
5 hpf (EV07010)
5 hpf (EV08013)
bud, 10 hpf (EV04012)
bud, 10 hpf (EV07013)
bud, 10 hpf (EV08016)
24 hpf (EV03007)
24 hpf (EV07016)
24 hpf (EV08019)
read count
references composition
alignment position (canonical position)
a
c
g
t
-
A
C
G
T

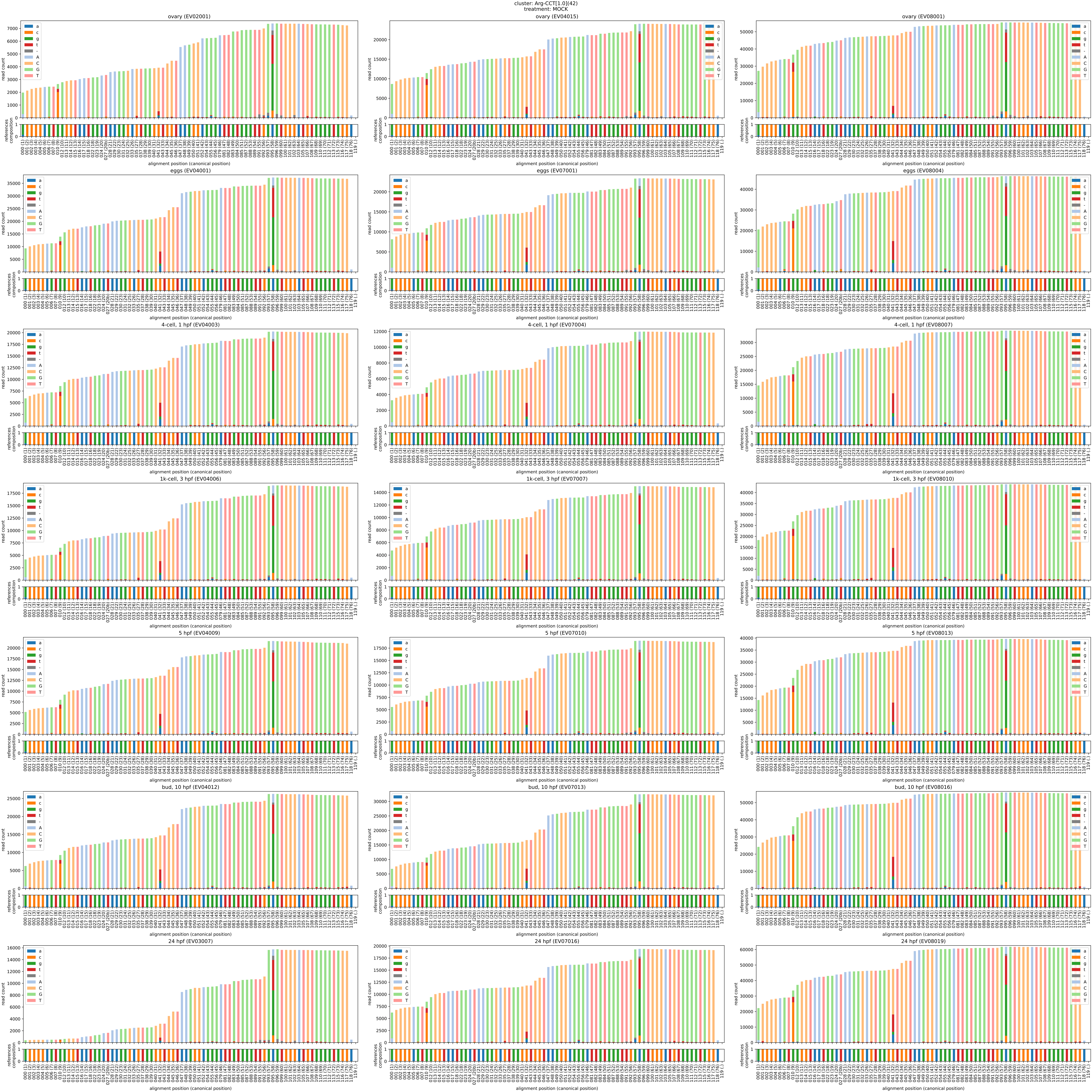
cluster: Arg-CCT[1.0](42)
treatment: MOCK
ovary (EV02001)
ovary (EV04015)
ovary (EV08001)
eggs (EV04001)
eggs (EV07001)
eggs (EV08004)
4-cell, 1 hpf (EV04003)
4-cell, 1 hpf (EV07004)
4-cell, 1 hpf (EV08007)
1k-cell, 3 hpf (EV04006)
1k-cell, 3 hpf (EV07007)
1k-cell, 3 hpf (EV08010)
5 hpf (EV04009)
5 hpf (EV07010)
5 hpf (EV08013)
bud, 10 hpf (EV04012)
bud, 10 hpf (EV07013)
bud, 10 hpf (EV08016)
24 hpf (EV03007)
24 hpf (EV07016)
24 hpf (EV08019)
read count
references composition
alignment position (canonical position)
a
c
g
t
-
A
C
G
T

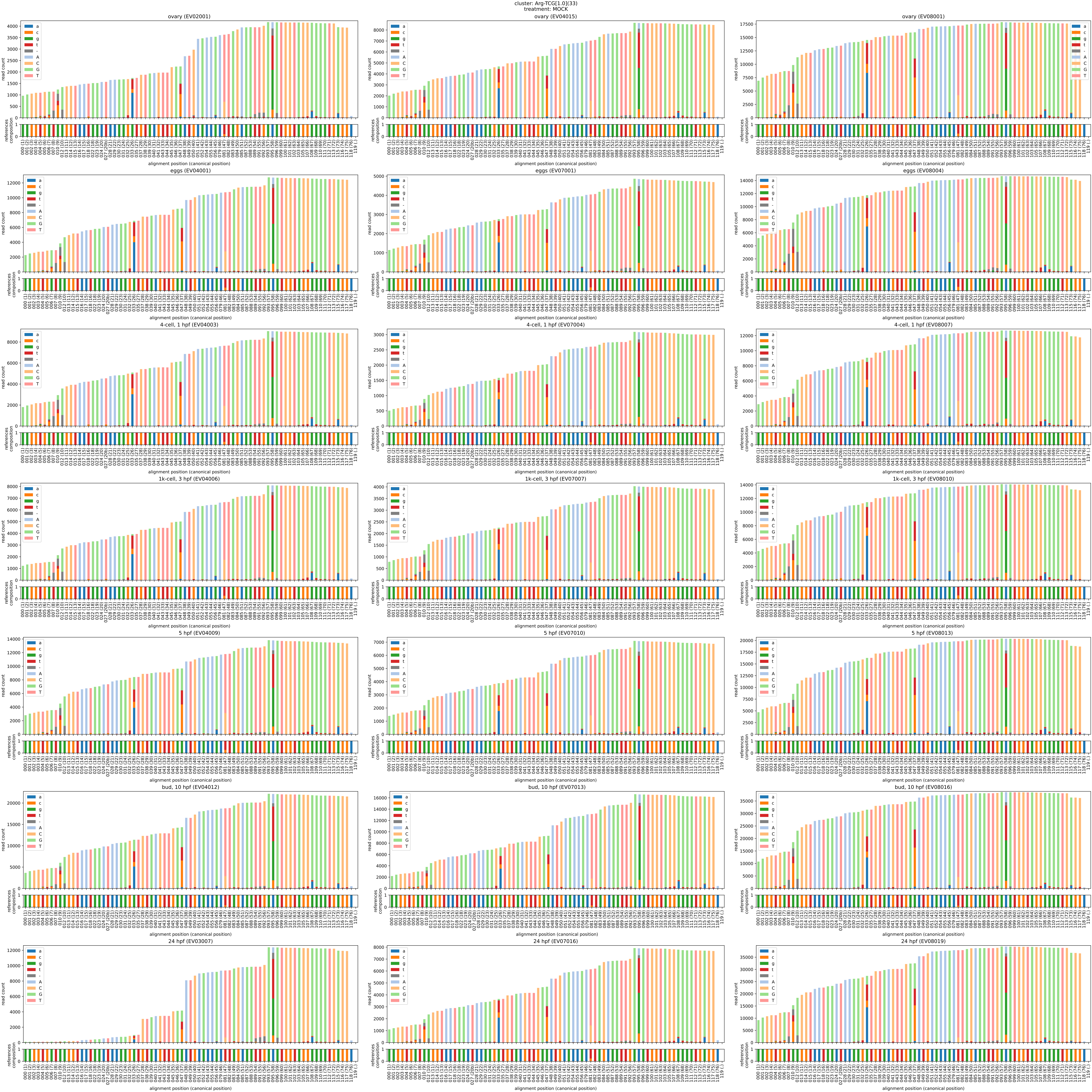
cluster: Arg-TCG[1,0](33)
treatment: MOCK
ovary (EV02001)
ovary (EV04015)
ovary (EV08001)
eggs (EV04001)
eggs (EV07001)
eggs (EV08004)
4-cell, 1 hpf (EV04003)
4-cell, 1 hpf (EV07004)
4-cell, 1 hpf (EV08007)
1k-cell, 3 hpf (EV04006)
1k-cell, 3 hpf (EV07007)
1k-cell, 3 hpf (EV08010)
5 hpf (EV04009)
5 hpf (EV07010)
5 hpf (EV08013)
bud, 10 hpf (EV04012)
bud, 10 hpf (EV07013)
bud, 10 hpf (EV08016)
24 hpf (EV03007)
24 hpf (EV07016)
24 hpf (EV08019)
read count
references composition
alignment position (canonical position)
a
c
g
t
-
A
C
G
T

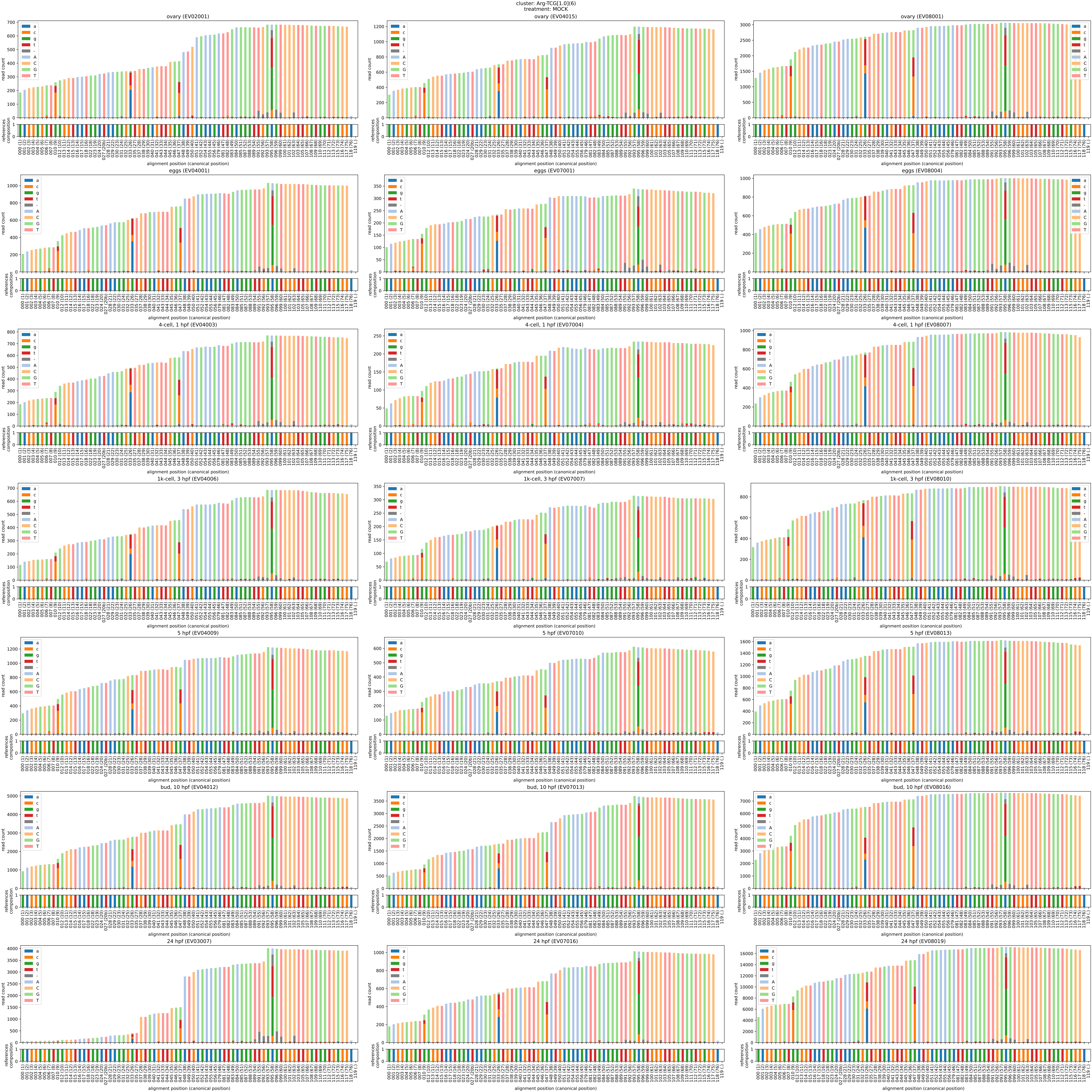
cluster: Arg-TCG[1.0](6)
treatment: MOCK
ovary (EV02001)
ovary (EV04015)
ovary (EV08001)
eggs (EV04001)
eggs (EV07001)
eggs (EV08004)
4-cell, 1 hpf (EV04003)
4-cell, 1 hpf (EV07004)
4-cell, 1 hpf (EV08007)
1k-cell, 3 hpf (EV04006)
1k-cell, 3 hpf (EV07007)
1k-cell, 3 hpf (EV08010)
5 hpf (EV04009)
5 hpf (EV07010)
5 hpf (EV08013)
bud, 10 hpf (EV04012)
bud, 10 hpf (EV07013)
bud, 10 hpf (EV08016)
24 hpf (EV03007)
24 hpf (EV07016)
24 hpf (EV08019)
read count
references composition
alignment position (canonical position)
a
c
g
t
-
A
C
G
T

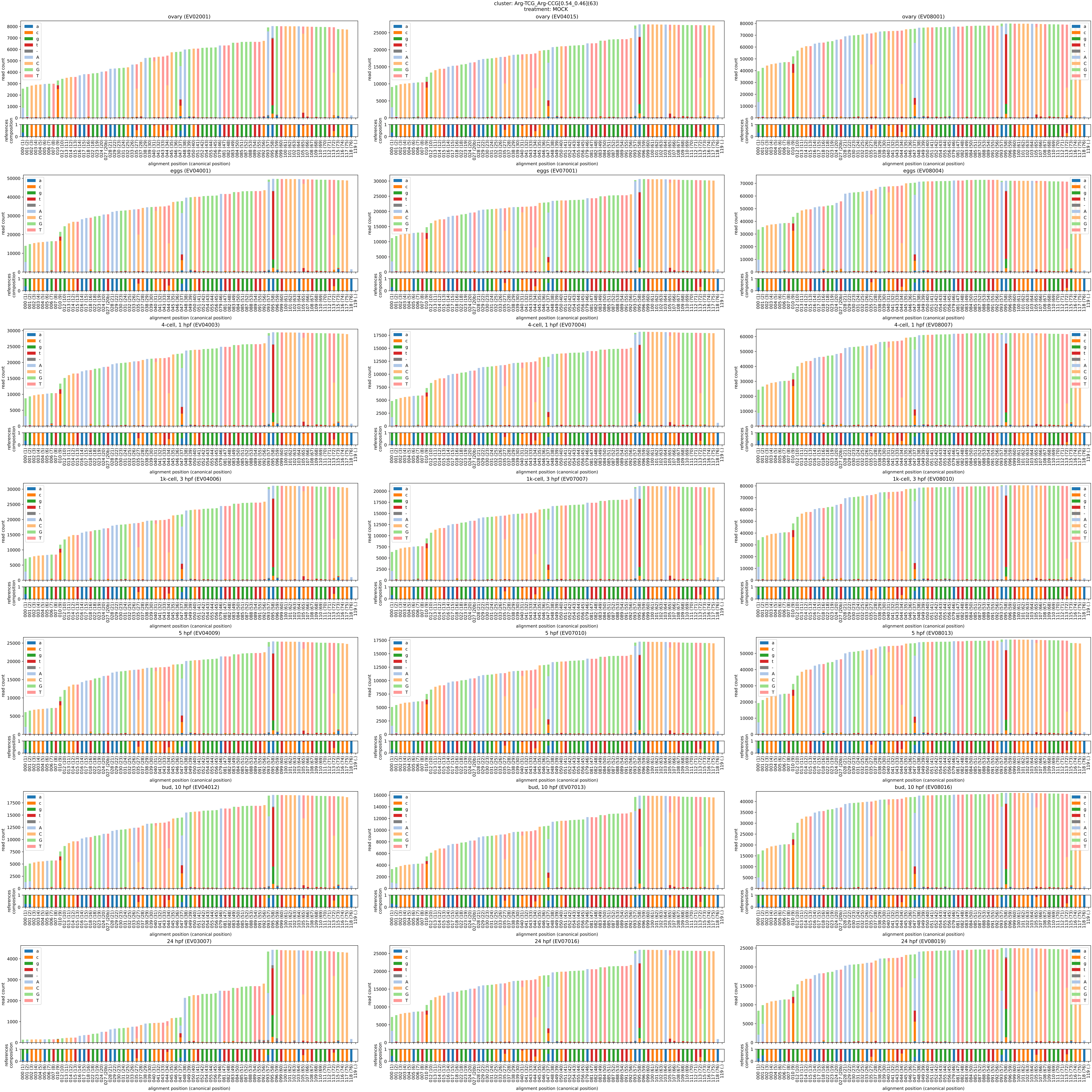
cluster: Arg-TCG_Arg-CCG[0.54_0.46](63)
treatment: MOCK
ovary (EV02001)
ovary (EV04015)
ovary (EV08001)
eggs (EV04001)
eggs (EV07001)
eggs (EV08004)
4-cell, 1 hpf (EV04003)
4-cell, 1 hpf (EV07004)
4-cell, 1 hpf (EV08007)
1k-cell, 3 hpf (EV04006)
1k-cell, 3 hpf (EV07007)
1k-cell, 3 hpf (EV08010)
5 hpf (EV04009)
5 hpf (EV07010)
5 hpf (EV08013)
bud, 10 hpf (EV04012)
bud, 10 hpf (EV07013)
bud, 10 hpf (EV08016)
24 hpf (EV03007)
24 hpf (EV07016)
24 hpf (EV08019)
read count
references composition
alignment position (canonical position)
a
c
g
t
-
A
C
G
T

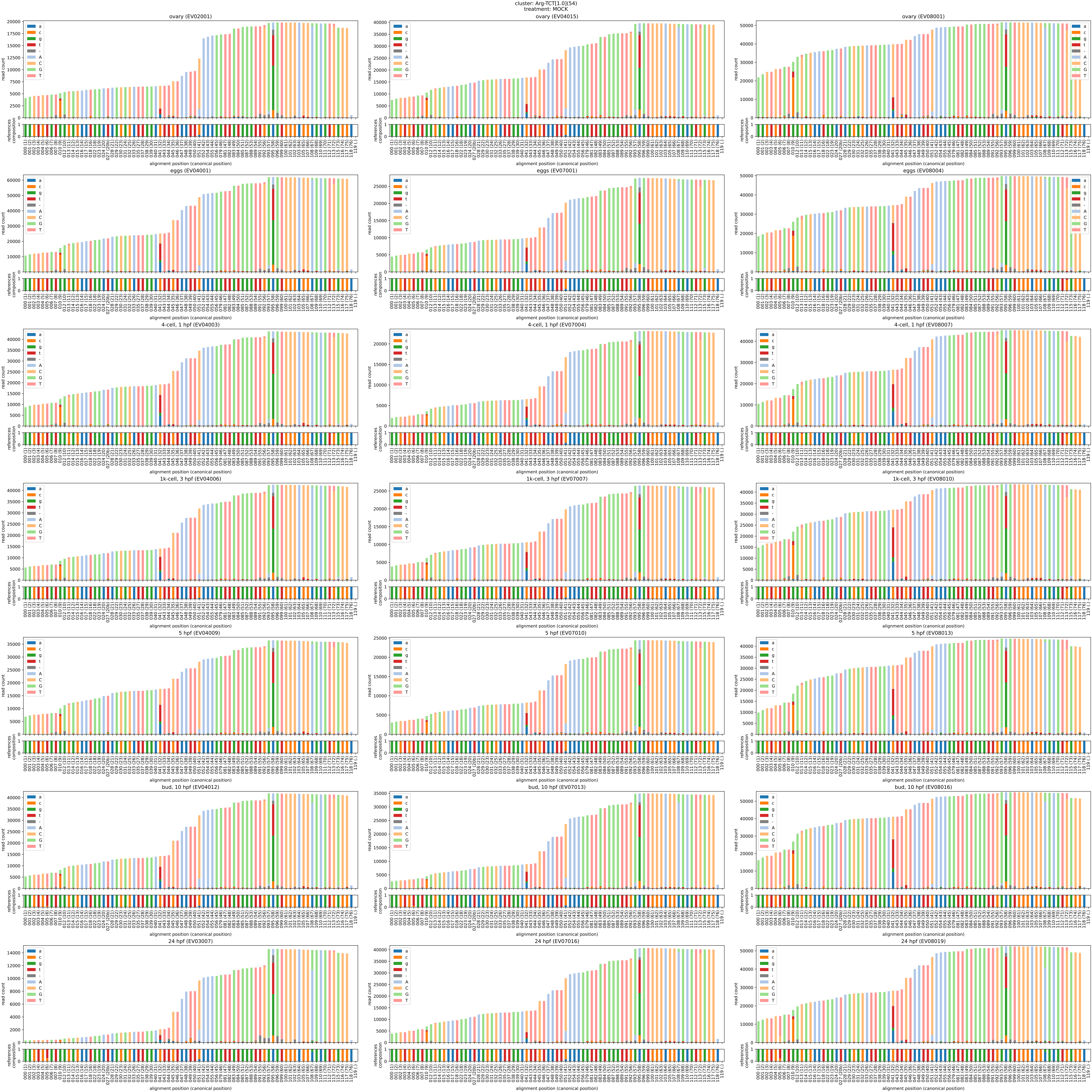
cluster: Arg-TCT[1.0](54)
treatment: MOCK
ovary (EV02001)
ovary (EV04015)
ovary (EV08001)
eggs (EV04001)
eggs (EV07001)
eggs (EV08004)
4-cell, 1 hpf (EV04003)
4-cell, 1 hpf (EV07004)
4-cell, 1 hpf (EV08007)
1k-cell, 3 hpf (EV04006)
1k-cell, 3 hpf (EV07007)
1k-cell, 3 hpf (EV08010)
5 hpf (EV04009)
5 hpf (EV07010)
5 hpf (EV08013)
bud, 10 hpf (EV04012)
bud, 10 hpf (EV07013)
bud, 10 hpf (EV08016)
24 hpf (EV03007)
24 hpf (EV07016)
24 hpf (EV08019)

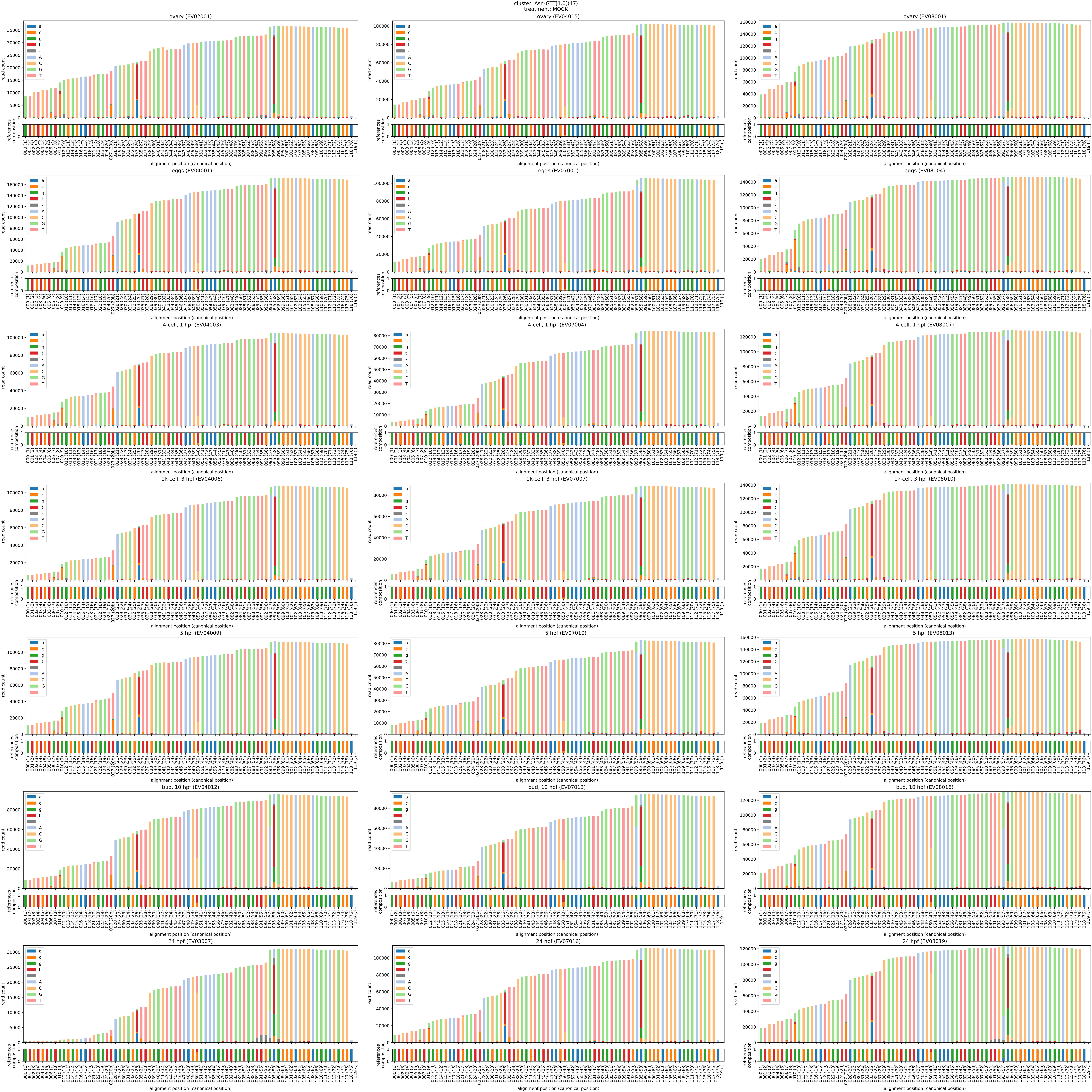
cluster: Asn-GTT[1.0](47)
treatment: MOCK
ovary (EV02001)
ovary (EV04015)
ovary (EV08001)
eggs (EV04001)
eggs (EV07001)
eggs (EV08004)
4-cell, 1 hpf (EV04003)
4-cell, 1 hpf (EV07004)
4-cell, 1 hpf (EV08007)
1k-cell, 3 hpf (EV04006)
1k-cell, 3 hpf (EV07007)
1k-cell, 3 hpf (EV08010)
5 hpf (EV04009)
5 hpf (EV07010)
5 hpf (EV08013)
bud, 10 hpf (EV04012)
bud, 10 hpf (EV07013)
bud, 10 hpf (EV08016)
24 hpf (EV03007)
24 hpf (EV07016)
24 hpf (EV08019)
read count
references composition
alignment position (canonical position)
a
c
g
t
-
A
C
G
T

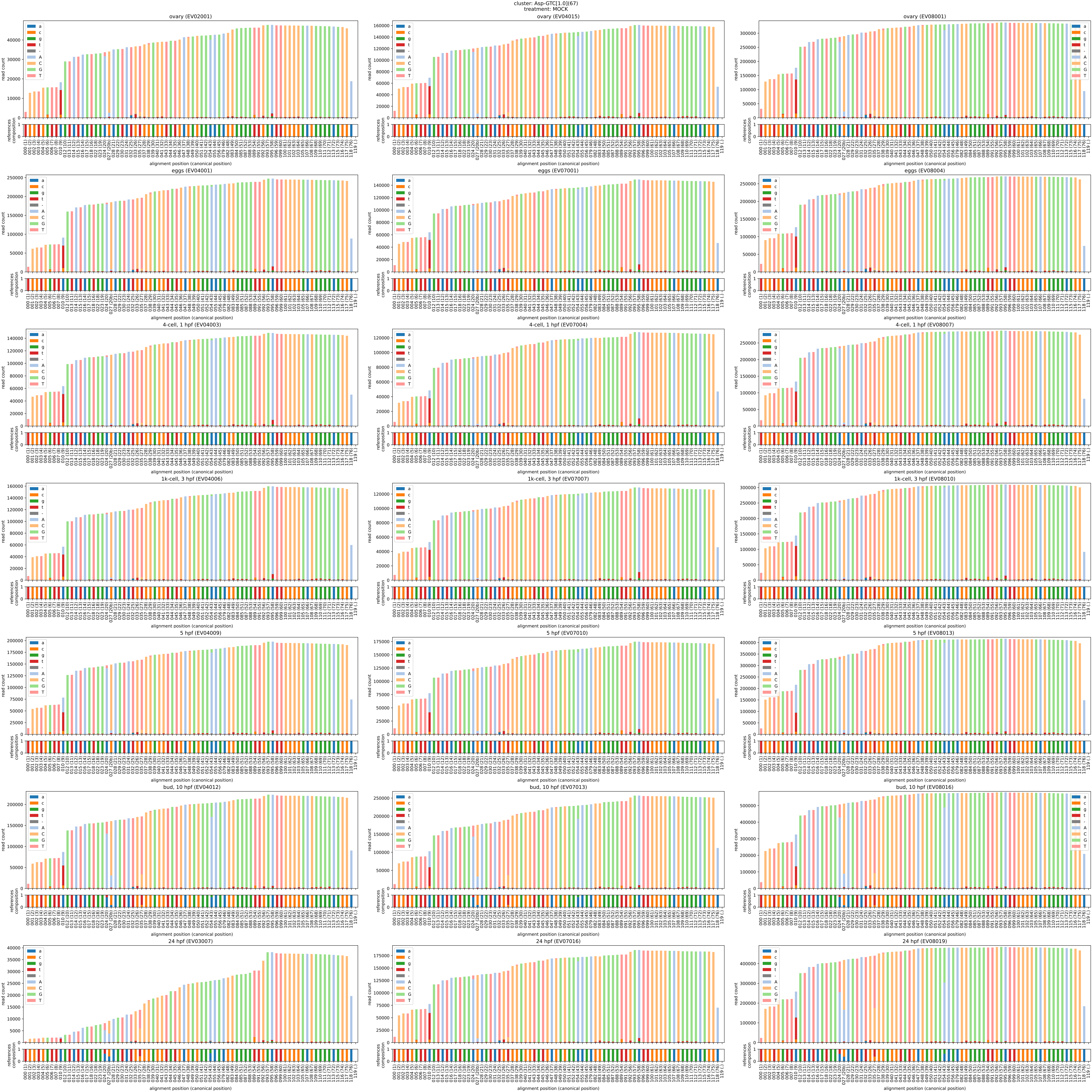
cluster: Asp-GTC[1.0](67)
treatment: MOCK
ovary (EV02001)
ovary (EV04015)
ovary (EV08001)
eggs (EV04001)
eggs (EV07001)
eggs (EV08004)
4-cell, 1 hpf (EV04003)
4-cell, 1 hpf (EV07004)
4-cell, 1 hpf (EV08007)
1k-cell, 3 hpf (EV04006)
1k-cell, 3 hpf (EV07007)
1k-cell, 3 hpf (EV08010)
5 hpf (EV04009)
5 hpf (EV07010)
5 hpf (EV08013)
bud, 10 hpf (EV04012)
bud, 10 hpf (EV07013)
bud, 10 hpf (EV08016)
24 hpf (EV03007)
24 hpf (EV07016)
24 hpf (EV08019)
read count
references composition
alignment position (canonical position)

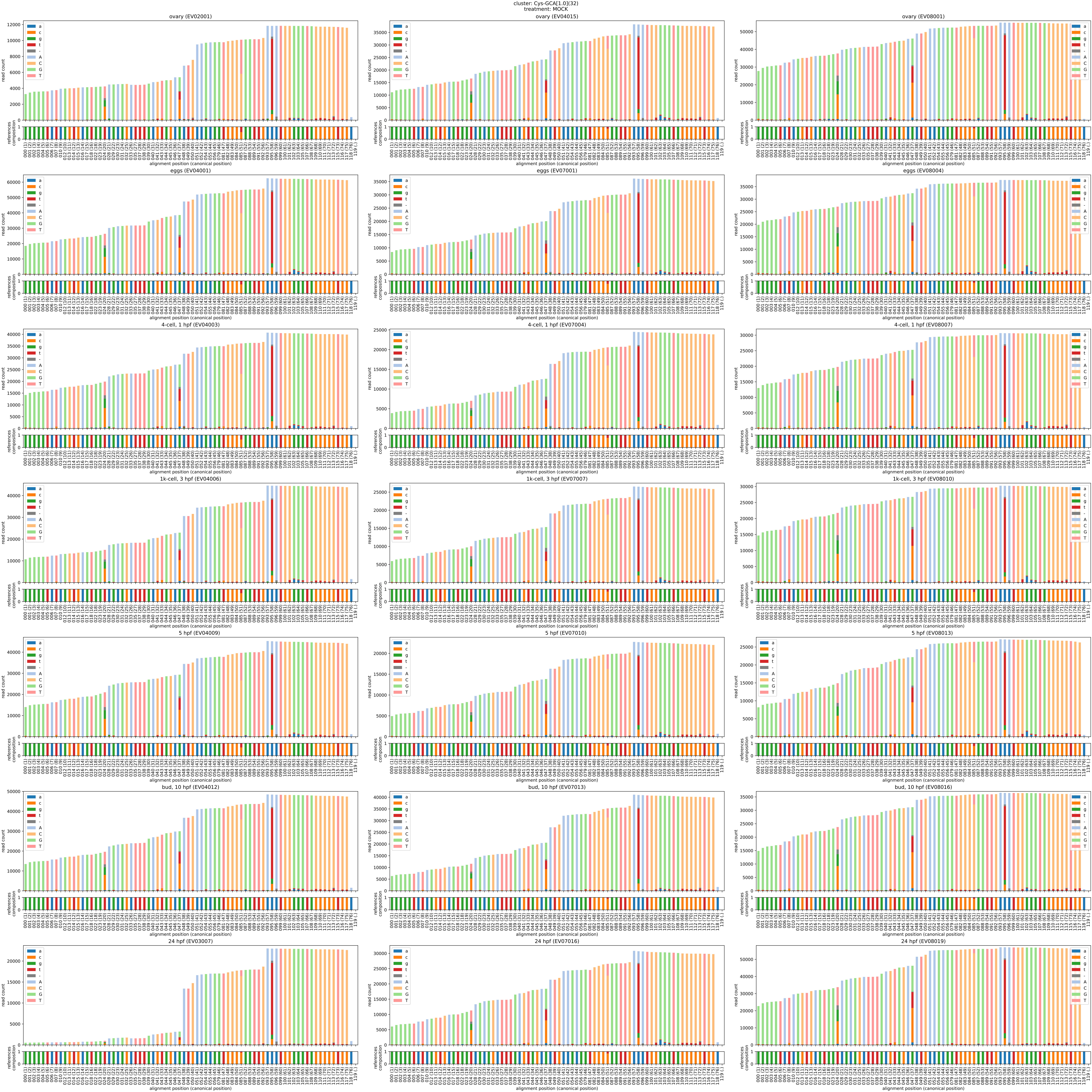
cluster: Cys-GCA[1.0](32)
treatment: MOCK
ovary (EV02001)
ovary (EV04015)
ovary (EV08001)
eggs (EV04001)
eggs (EV07001)
eggs (EV08004)
4-cell, 1 hpf (EV04003)
4-cell, 1 hpf (EV07004)
4-cell, 1 hpf (EV08007)
1k-cell, 3 hpf (EV04006)
1k-cell, 3 hpf (EV07007)
1k-cell, 3 hpf (EV08010)
5 hpf (EV04009)
5 hpf (EV07010)
5 hpf (EV08013)
bud, 10 hpf (EV04012)
bud, 10 hpf (EV07013)
bud, 10 hpf (EV08016)
24 hpf (EV03007)
24 hpf (EV07016)
24 hpf (EV08019)
read count
references composition
alignment position (canonical position)
a
c
g
t
-
A
C
G
T

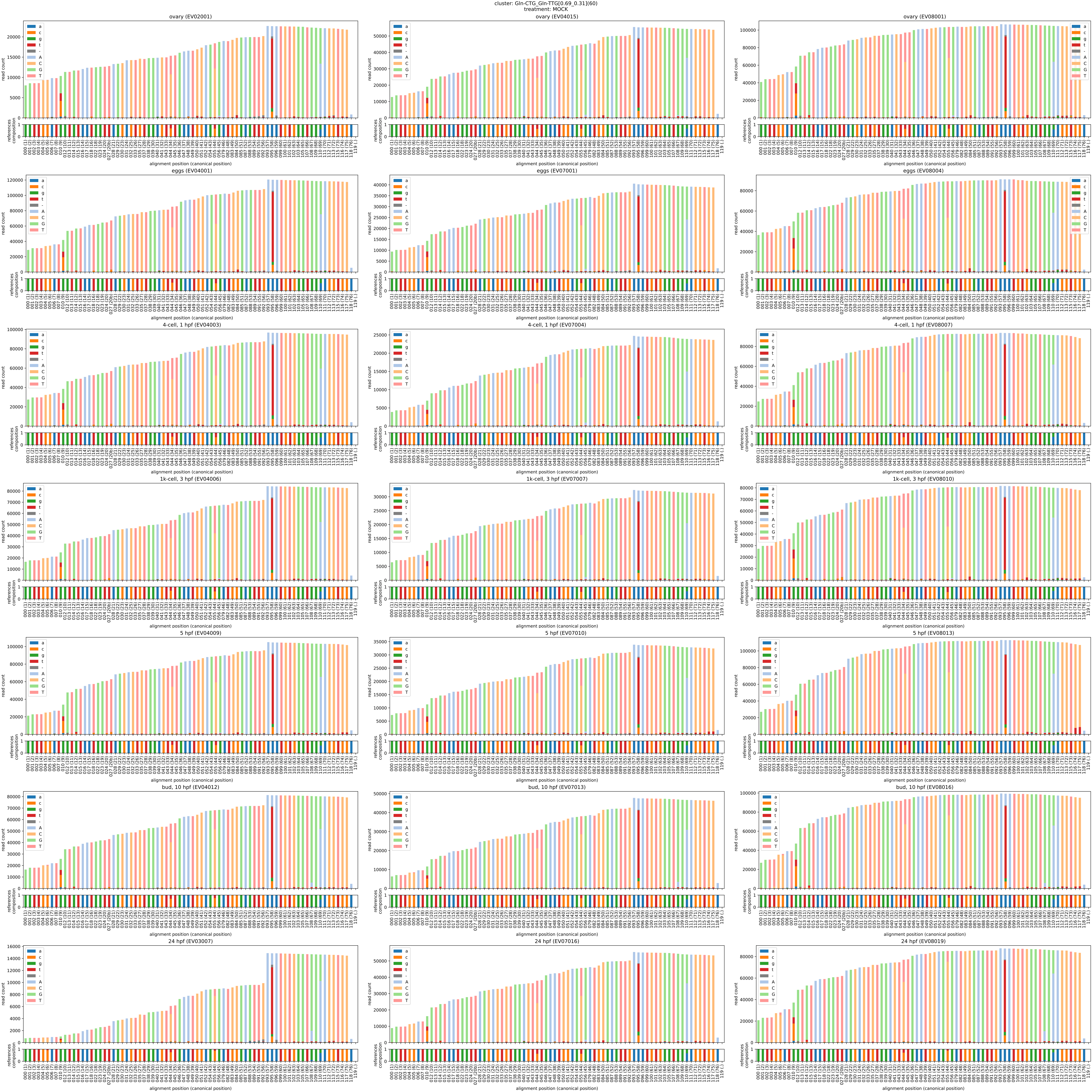
cluster: Gln-CTG_Gln-TTG[0.69_0.31](60)
treatment: MOCK
ovary (EV02001)
ovary (EV04015)
ovary (EV08001)
eggs (EV04001)
eggs (EV07001)
eggs (EV08004)
4-cell, 1 hpf (EV04003)
4-cell, 1 hpf (EV07004)
4-cell, 1 hpf (EV08007)
1k-cell, 3 hpf (EV04006)
1k-cell, 3 hpf (EV07007)
1k-cell, 3 hpf (EV08010)
5 hpf (EV04009)
5 hpf (EV07010)
5 hpf (EV08013)
bud, 10 hpf (EV04012)
bud, 10 hpf (EV07013)
bud, 10 hpf (EV08016)
24 hpf (EV03007)
24 hpf (EV07016)
24 hpf (EV08019)
read count
references composition
alignment position (canonical position)
a
c
g
t
-
A
C
G
T

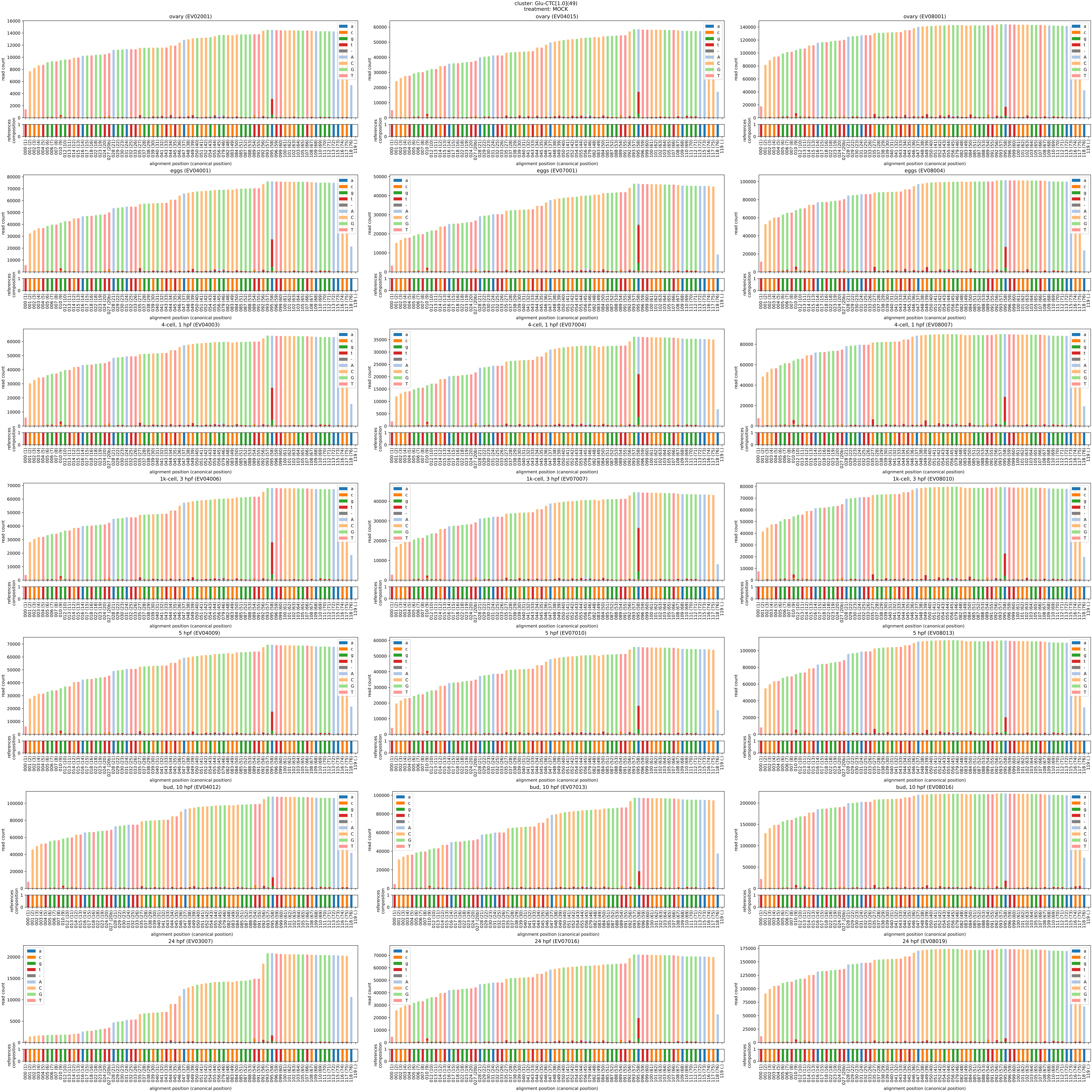
cluster: Glu-CTC[1.0](49)
treatment: MOCK
ovary (EV02001)
ovary (EV04015)
ovary (EV08001)
eggs (EV04001)
eggs (EV07001)
eggs (EV08004)
4-cell, 1 hpf (EV04003)
4-cell, 1 hpf (EV07004)
4-cell, 1 hpf (EV08007)
1k-cell, 3 hpf (EV04006)
1k-cell, 3 hpf (EV07007)
1k-cell, 3 hpf (EV08010)
5 hpf (EV04009)
5 hpf (EV07010)
5 hpf (EV08013)
bud, 10 hpf (EV04012)
bud, 10 hpf (EV07013)
bud, 10 hpf (EV08016)
24 hpf (EV03007)
24 hpf (EV07016)
24 hpf (EV08019)
read count
references composition
alignment position (canonical position)
a
c
g
t
-
A
C
G
T

cluster: Glu-TTC_Glu-CTC[0.87_0.13](40)
treatment: MOCK

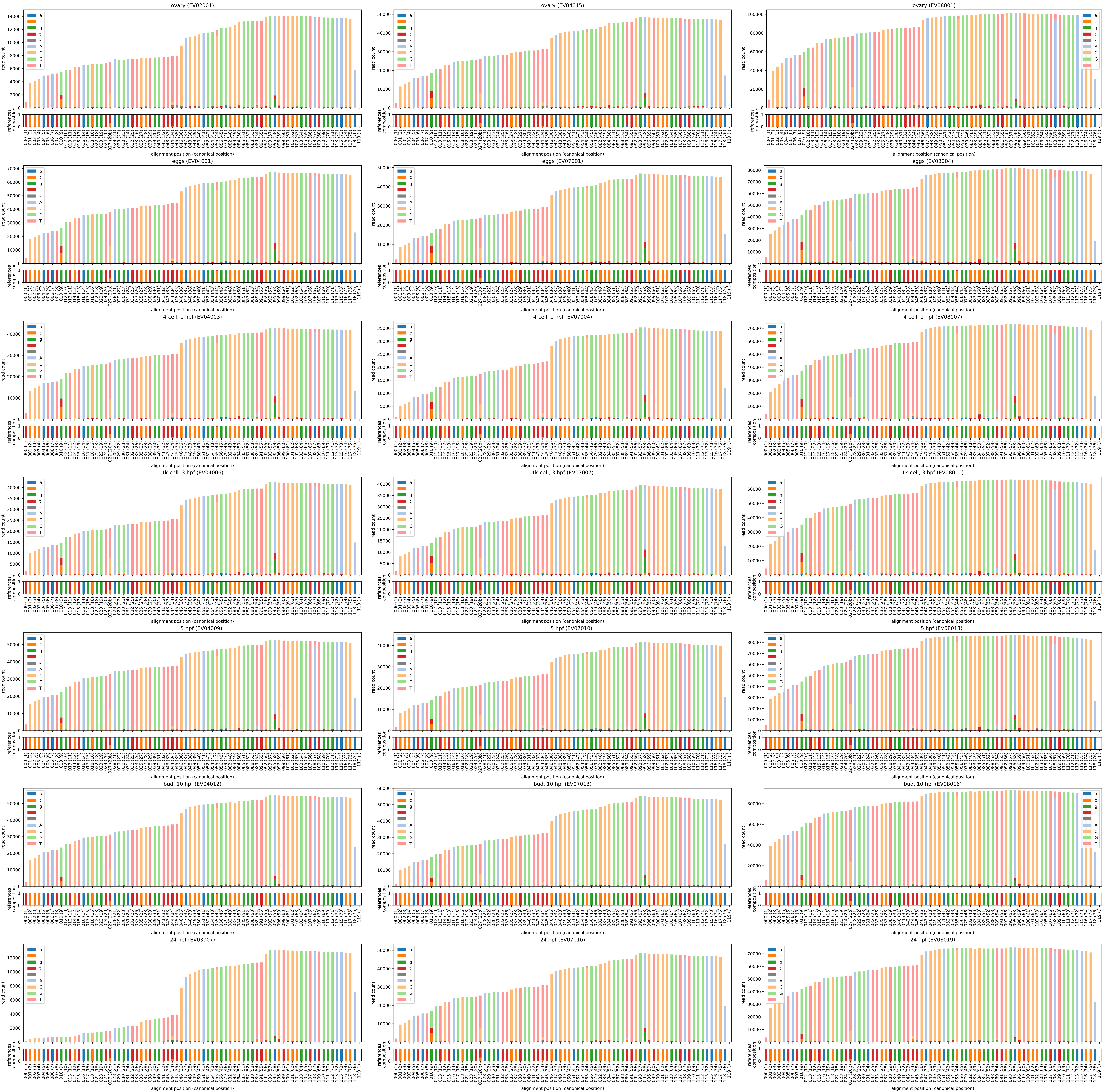

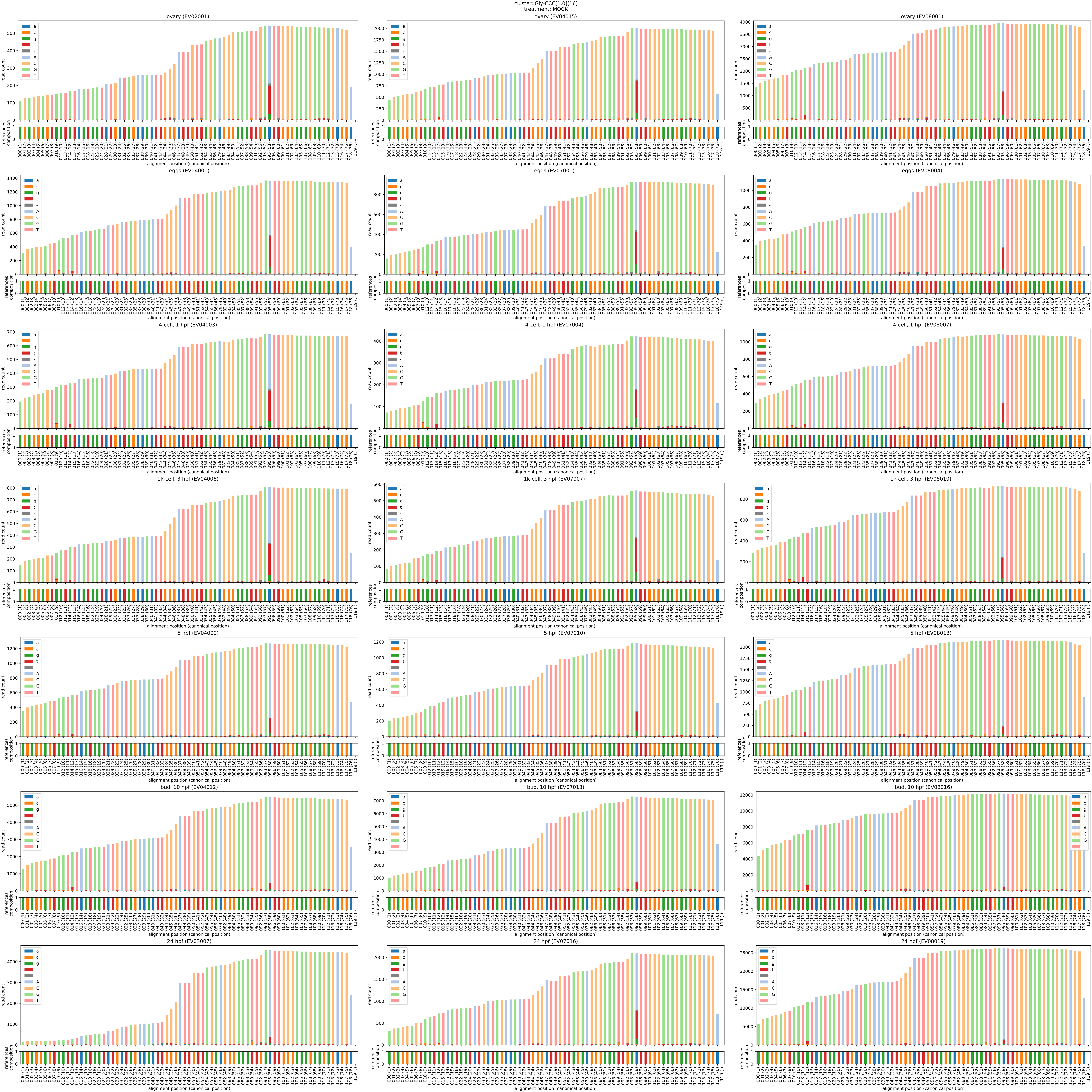
cluster: Gly-CCC[1.0](16)
treatment: MOCK
ovary (EV02001)
ovary (EV04015)
ovary (EV08001)
eggs (EV04001)
eggs (EV07001)
eggs (EV08004)
4-cell, 1 hpf (EV04003)
4-cell, 1 hpf (EV07004)
4-cell, 1 hpf (EV08007)
1k-cell, 3 hpf (EV04006)
1k-cell, 3 hpf (EV07007)
1k-cell, 3 hpf (EV08010)
5 hpf (EV04009)
5 hpf (EV07010)
5 hpf (EV08013)
bud, 10 hpf (EV04012)
bud, 10 hpf (EV07013)
bud, 10 hpf (EV08016)
24 hpf (EV03007)
24 hpf (EV07016)
24 hpf (EV08019)
read count
references composition
alignment position (canonical position)
a
c
g
t
-
A
C
G
T

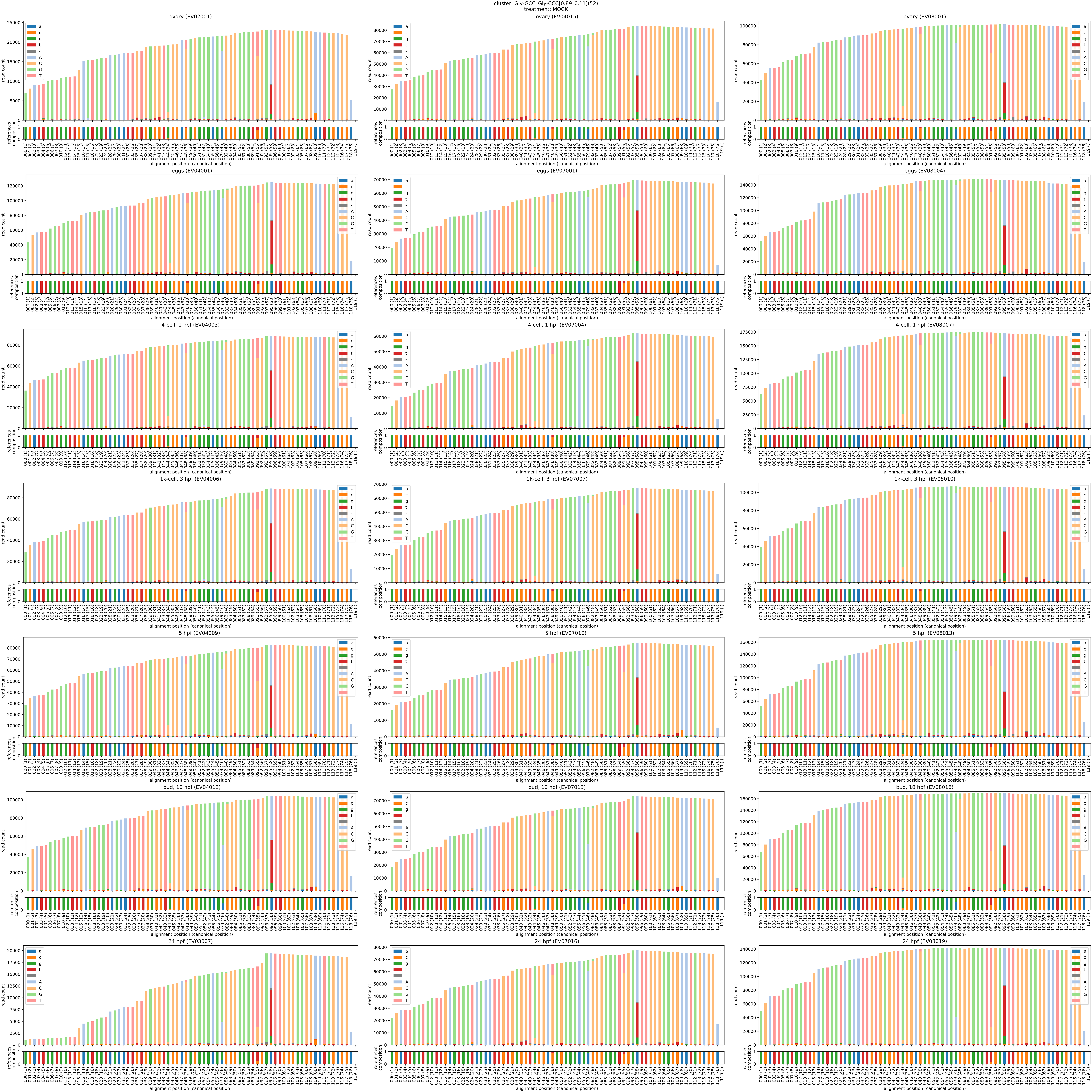

cluster: Gly-GCC_Gly-CCC[0.89_0.11](52)
treatment: MOCK
ovary (EV02001)
ovary (EV04015)
ovary (EV08001)
eggs (EV04001)
eggs (EV07001)
eggs (EV08004)
4-cell, 1 hpf (EV04003)
4-cell, 1 hpf (EV07004)
4-cell, 1 hpf (EV08007)
1k-cell, 3 hpf (EV04006)
1k-cell, 3 hpf (EV07007)
1k-cell, 3 hpf (EV08010)
5 hpf (EV04009)
5 hpf (EV07010)
5 hpf (EV08013)
bud, 10 hpf (EV04012)
bud, 10 hpf (EV07013)
bud, 10 hpf (EV08016)
24 hpf (EV03007)
24 hpf (EV07016)
24 hpf (EV08019)
read count
references composition
alignment position (canonical position)

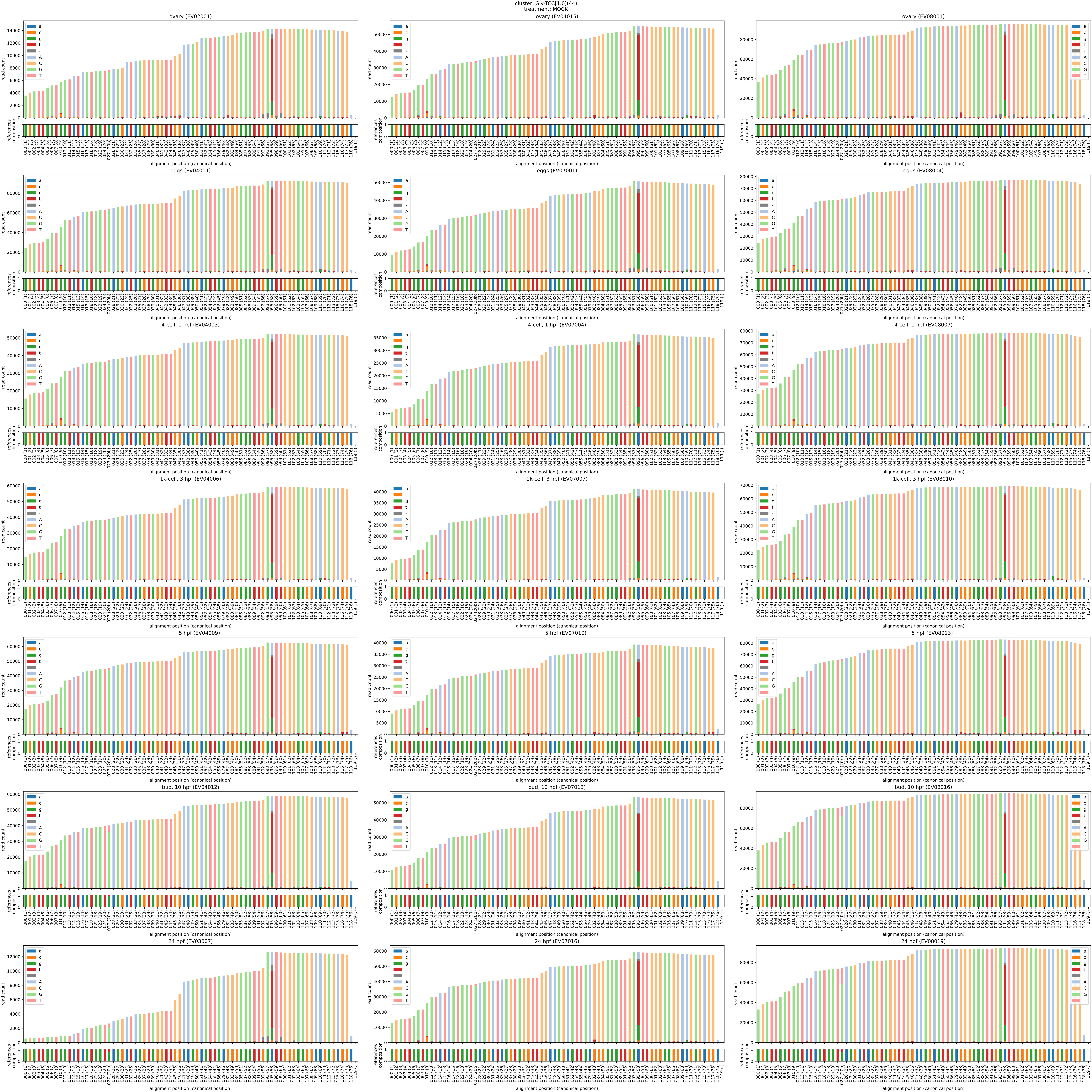
cluster: Gly-TCC[1.0](44)
treatment: MOCK
ovary (EV02001)
ovary (EV04015)
ovary (EV08001)
eggs (EV04001)
eggs (EV07001)
eggs (EV08004)
4-cell, 1 hpf (EV04003)
4-cell, 1 hpf (EV07004)
4-cell, 1 hpf (EV08007)
1k-cell, 3 hpf (EV04006)
1k-cell, 3 hpf (EV07007)
1k-cell, 3 hpf (EV08010)
5 hpf (EV04009)
5 hpf (EV07010)
5 hpf (EV08013)
bud, 10 hpf (EV04012)
bud, 10 hpf (EV07013)
bud, 10 hpf (EV08016)
24 hpf (EV03007)
24 hpf (EV07016)
24 hpf (EV08019)
read count
references composition
alignment position (canonical position)
a
c
g
t
-
A
C
G
T

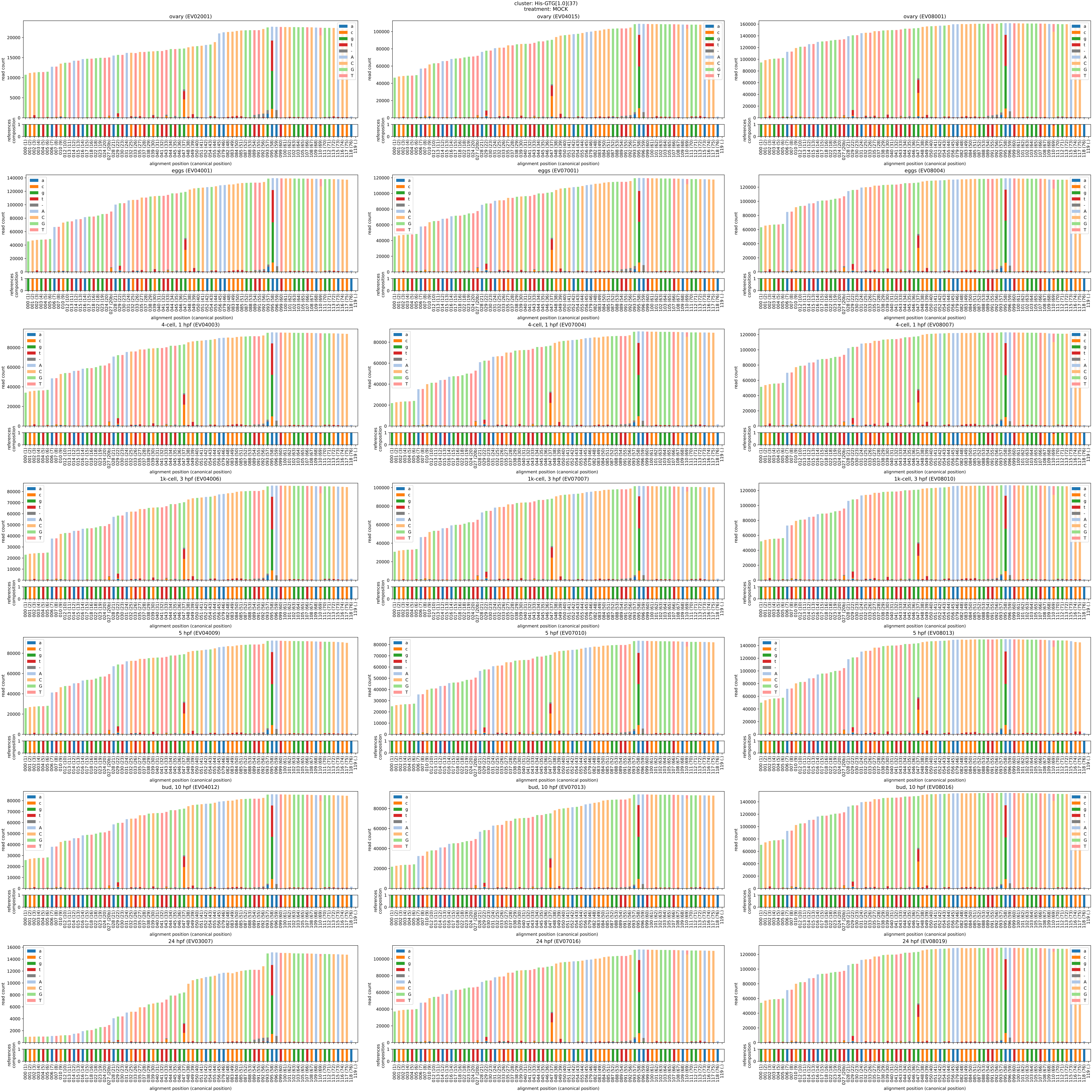

cluster: His-GTG[1.0](37)
treatment: MOCK
ovary (EV02001)
ovary (EV04015)
ovary (EV08001)
eggs (EV04001)
eggs (EV07001)
eggs (EV08004)
4-cell, 1 hpf (EV04003)
4-cell, 1 hpf (EV07004)
4-cell, 1 hpf (EV08007)
1k-cell, 3 hpf (EV04006)
1k-cell, 3 hpf (EV07007)
1k-cell, 3 hpf (EV08010)
5 hpf (EV04009)
5 hpf (EV07010)
5 hpf (EV08013)
bud, 10 hpf (EV04012)
bud, 10 hpf (EV07013)
bud, 10 hpf (EV08016)
24 hpf (EV03007)
24 hpf (EV07016)
24 hpf (EV08019)
read count
references composition
alignment position (canonical position)
a
c
g
t
-
A
C
G
T

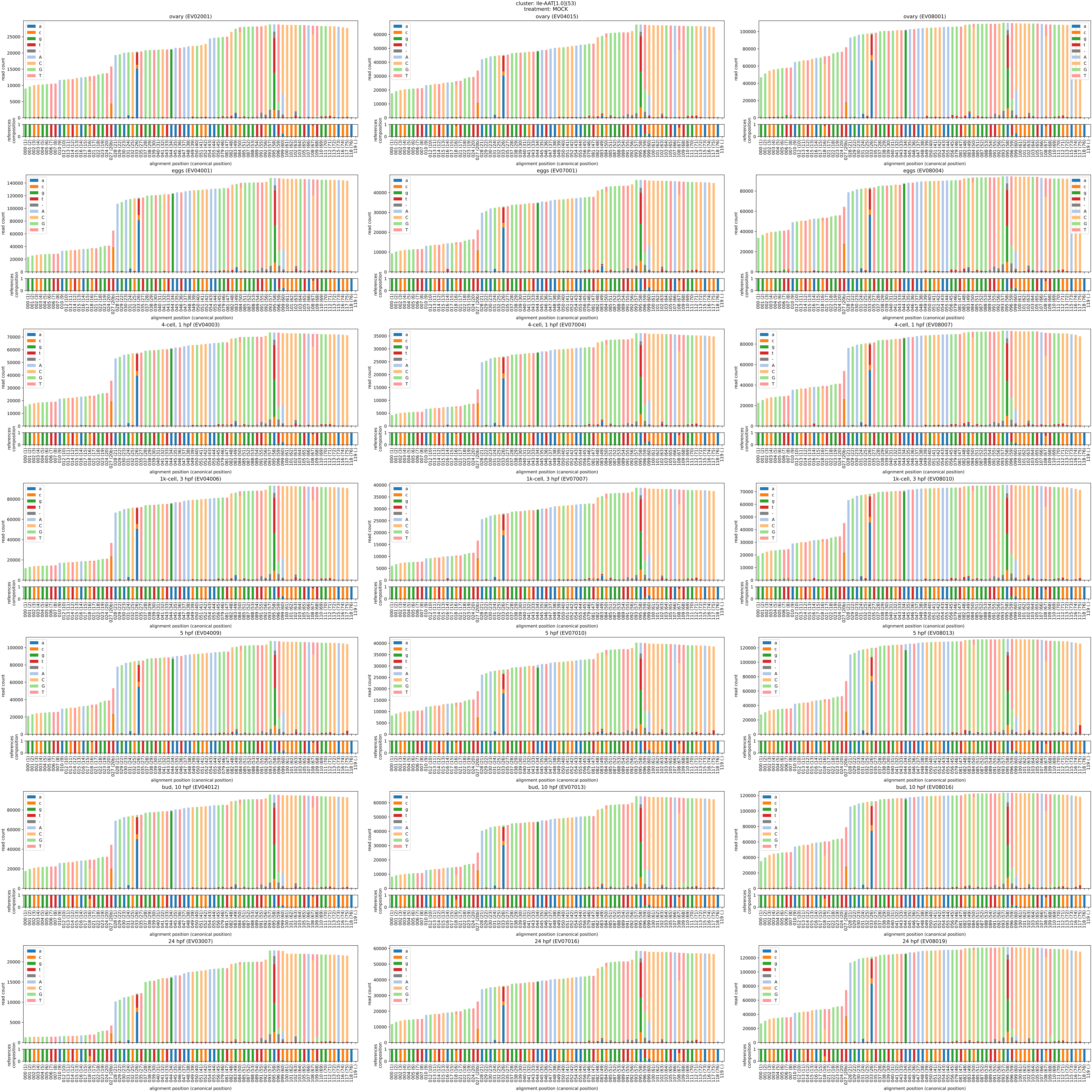
cluster: Ile-AAT[1.0](53)
treatment: MOCK
ovary (EV02001)
ovary (EV04015)
ovary (EV08001)
eggs (EV04001)
eggs (EV07001)
eggs (EV08004)
4-cell, 1 hpf (EV04003)
4-cell, 1 hpf (EV07004)
4-cell, 1 hpf (EV08007)
1k-cell, 3 hpf (EV04006)
1k-cell, 3 hpf (EV07007)
1k-cell, 3 hpf (EV08010)
5 hpf (EV04009)
5 hpf (EV07010)
5 hpf (EV08013)
bud, 10 hpf (EV04012)
bud, 10 hpf (EV07013)
bud, 10 hpf (EV08016)
24 hpf (EV03007)
24 hpf (EV07016)
24 hpf (EV08019)
read count
references composition
alignment position (canonical position)
a
c
g
t
-
A
C
G
T

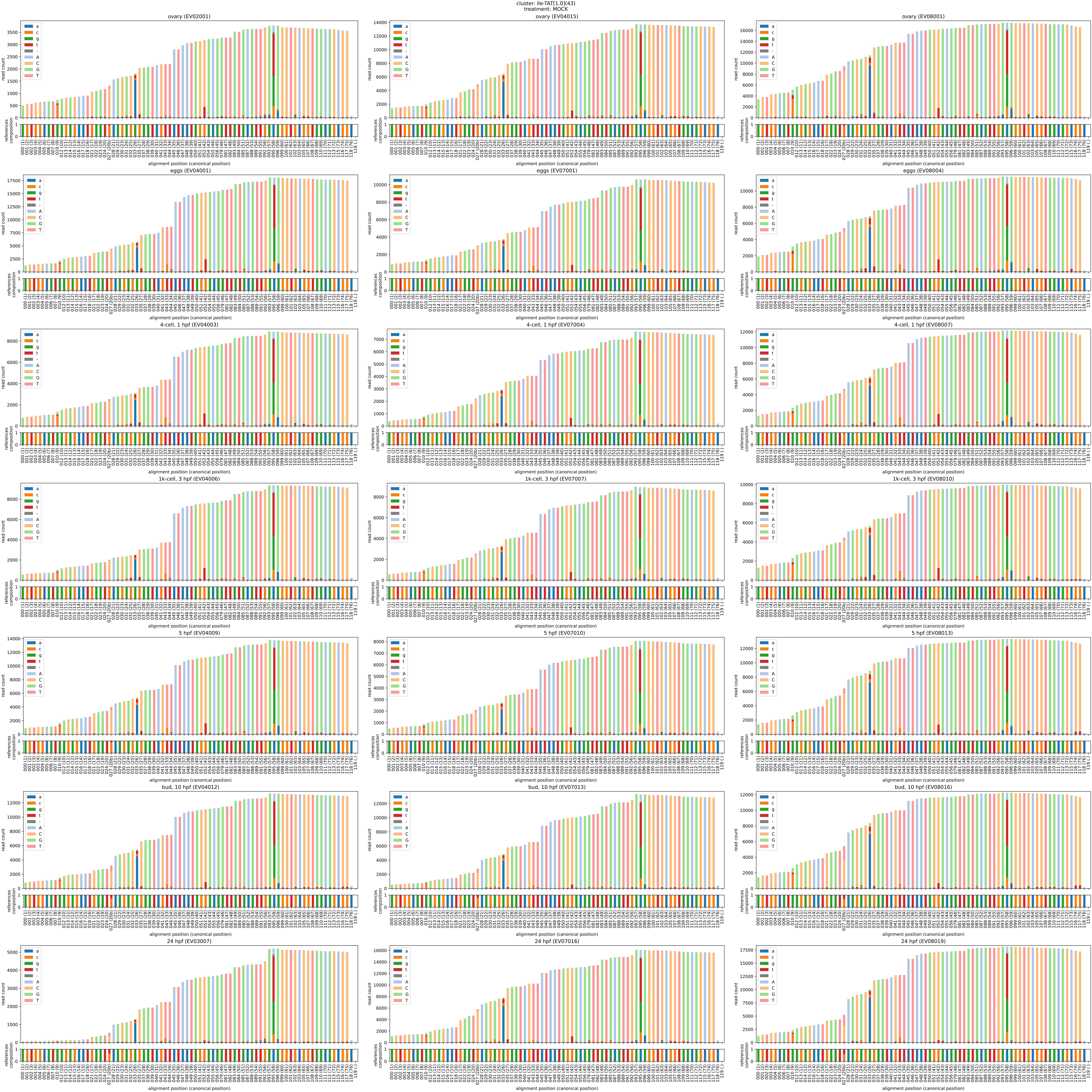
cluster: Ile-TAT[1.0](43)
treatment: MOCK
ovary (EV02001)
ovary (EV04015)
ovary (EV08001)
eggs (EV04001)
eggs (EV07001)
eggs (EV08004)
4-cell, 1 hpf (EV04003)
4-cell, 1 hpf (EV07004)
4-cell, 1 hpf (EV08007)
1k-cell, 3 hpf (EV04006)
1k-cell, 3 hpf (EV07007)
1k-cell, 3 hpf (EV08010)
5 hpf (EV04009)
5 hpf (EV07010)
5 hpf (EV08013)
bud, 10 hpf (EV04012)
bud, 10 hpf (EV07013)
bud, 10 hpf (EV08016)
24 hpf (EV03007)
24 hpf (EV07016)
24 hpf (EV08019)
read count
references composition
alignment position (canonical position)
a
c
g
t
-
A
C
G
T

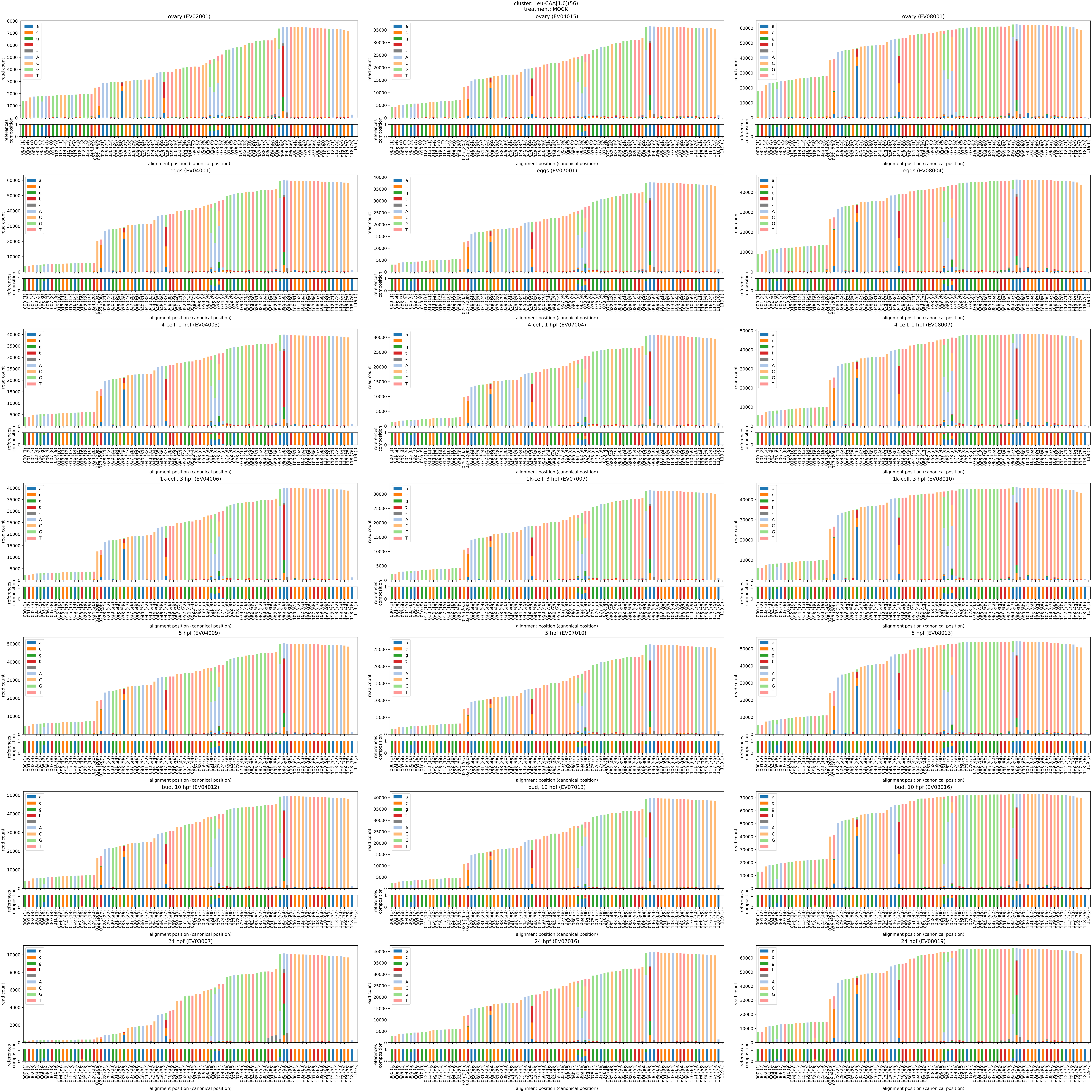
cluster: Leu-CAA[1.0](56)
treatment: MOCK
ovary (EV02001)
ovary (EV04015)
ovary (EV08001)
eggs (EV04001)
eggs (EV07001)
eggs (EV08004)
4-cell, 1 hpf (EV04003)
4-cell, 1 hpf (EV07004)
4-cell, 1 hpf (EV08007)
1k-cell, 3 hpf (EV04006)
1k-cell, 3 hpf (EV07007)
1k-cell, 3 hpf (EV08010)
5 hpf (EV04009)
5 hpf (EV07010)
5 hpf (EV08013)
bud, 10 hpf (EV04012)
bud, 10 hpf (EV07013)
bud, 10 hpf (EV08016)
24 hpf (EV03007)
24 hpf (EV07016)
24 hpf (EV08019)
read count
references composition
alignment position (canonical position)
a
c
g
t
-
A
C
G
T

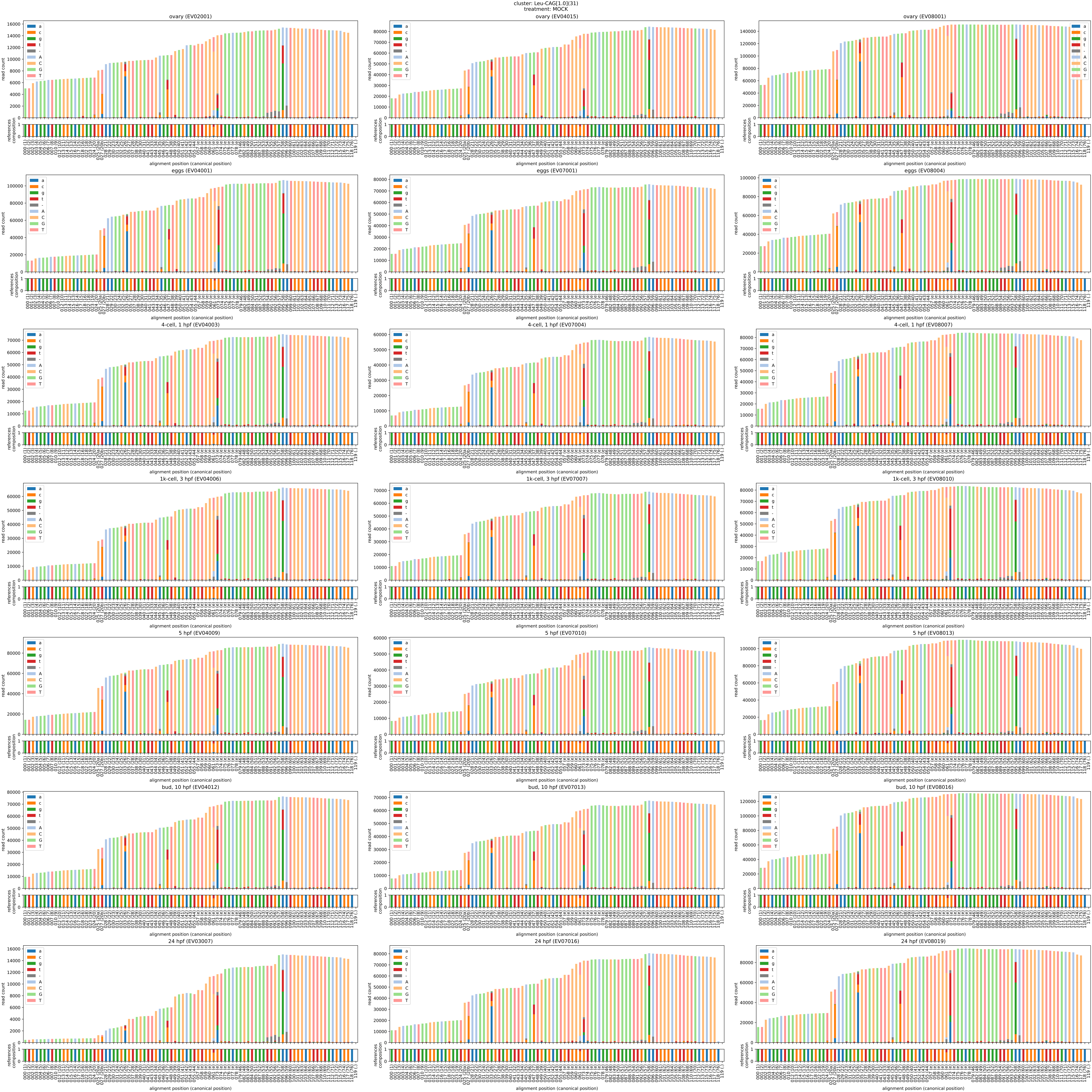
cluster: Leu-CAG[1.0](31)
treatment: MOCK
ovary (EV02001)
ovary (EV04015)
ovary (EV08001)
eggs (EV04001)
eggs (EV07001)
eggs (EV08004)
4-cell, 1 hpf (EV04003)
4-cell, 1 hpf (EV07004)
4-cell, 1 hpf (EV08007)
1k-cell, 3 hpf (EV04006)
1k-cell, 3 hpf (EV07007)
1k-cell, 3 hpf (EV08010)
5 hpf (EV04009)
5 hpf (EV07010)
5 hpf (EV08013)
bud, 10 hpf (EV04012)
bud, 10 hpf (EV07013)
bud, 10 hpf (EV08016)
24 hpf (EV03007)
24 hpf (EV07016)
24 hpf (EV08019)
read count
references composition
alignment position (canonical position)
a
c
g
t
-
A
C
G
T

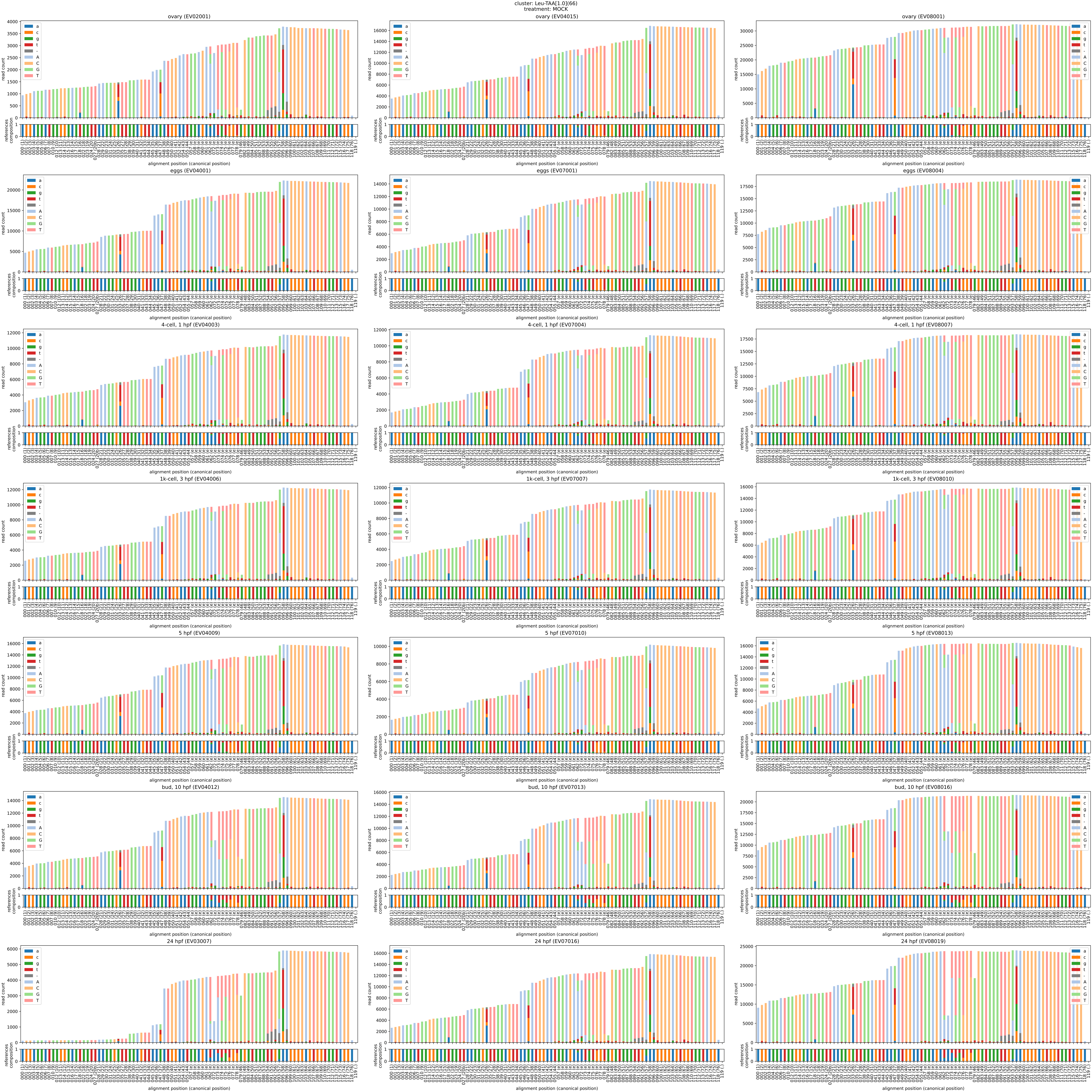
cluster: Leu-TAA[1.0](66)
treatment: MOCK
ovary (EV02001)
ovary (EV04015)
ovary (EV08001)
eggs (EV04001)
eggs (EV07001)
eggs (EV08004)
4-cell, 1 hpf (EV04003)
4-cell, 1 hpf (EV07004)
4-cell, 1 hpf (EV08007)
1k-cell, 3 hpf (EV04006)
1k-cell, 3 hpf (EV07007)
1k-cell, 3 hpf (EV08010)
5 hpf (EV04009)
5 hpf (EV07010)
5 hpf (EV08013)
bud, 10 hpf (EV04012)
bud, 10 hpf (EV07013)
bud, 10 hpf (EV08016)
24 hpf (EV03007)
24 hpf (EV07016)
24 hpf (EV08019)
read count
references composition
alignment position (canonical position)
a
c
g
t
-
A
C
G
T

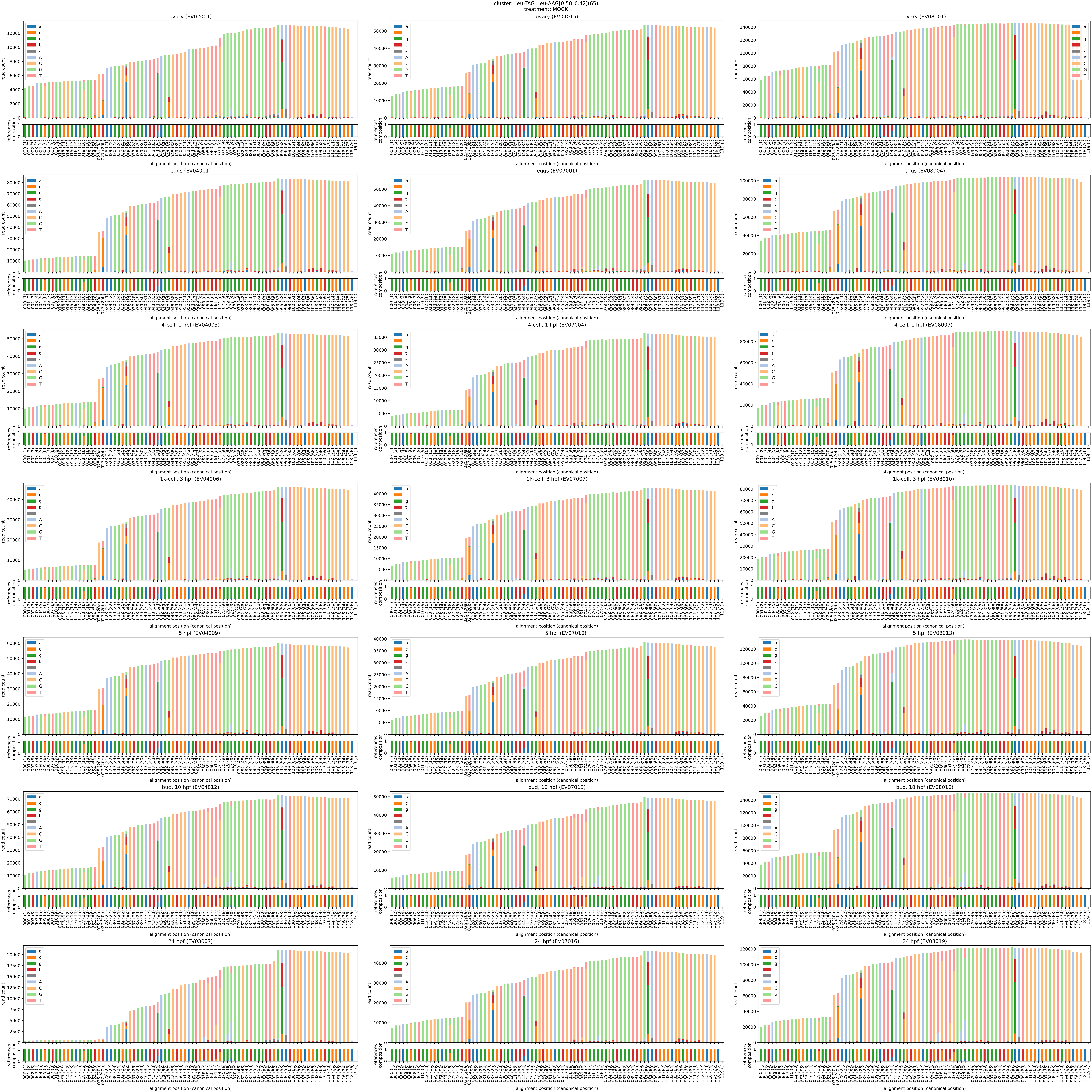
cluster: Leu-TAG_Leu-AAG[0.58_0.42](65)
treatment: MOCK
ovary (EV02001)
ovary (EV04015)
ovary (EV08001)
eggs (EV04001)
eggs (EV07001)
eggs (EV08004)
4-cell, 1 hpf (EV04003)
4-cell, 1 hpf (EV07004)
4-cell, 1 hpf (EV08007)
1k-cell, 3 hpf (EV04006)
1k-cell, 3 hpf (EV07007)
1k-cell, 3 hpf (EV08010)
5 hpf (EV04009)
5 hpf (EV07010)
5 hpf (EV08013)
bud, 10 hpf (EV04012)
bud, 10 hpf (EV07013)
bud, 10 hpf (EV08016)
24 hpf (EV03007)
24 hpf (EV07016)
24 hpf (EV08019)
read count
references composition
alignment position (canonical position)
a
c
g
t
-
A
C
G
T

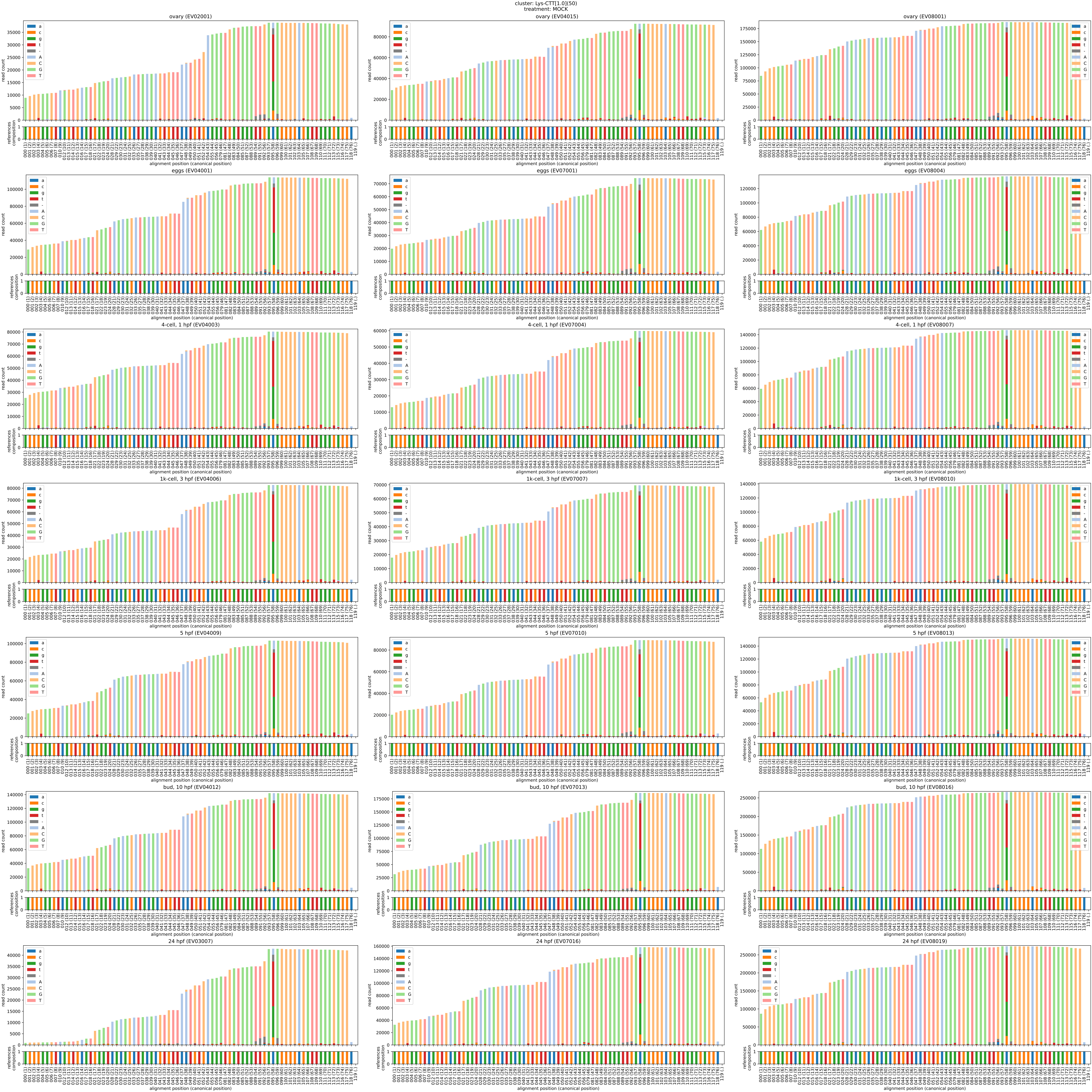
cluster: Lys-CTT[1.0](50)
treatment: MOCK
ovary (EV02001)
ovary (EV04015)
ovary (EV08001)
eggs (EV04001)
eggs (EV07001)
eggs (EV08004)
4-cell, 1 hpf (EV04003)
4-cell, 1 hpf (EV07004)
4-cell, 1 hpf (EV08007)
1k-cell, 3 hpf (EV04006)
1k-cell, 3 hpf (EV07007)
1k-cell, 3 hpf (EV08010)
5 hpf (EV04009)
5 hpf (EV07010)
5 hpf (EV08013)
bud, 10 hpf (EV04012)
bud, 10 hpf (EV07013)
bud, 10 hpf (EV08016)
24 hpf (EV03007)
24 hpf (EV07016)
24 hpf (EV08019)
read count
references composition
alignment position (canonical position)
a
c
g
t
-
A
C
G
T

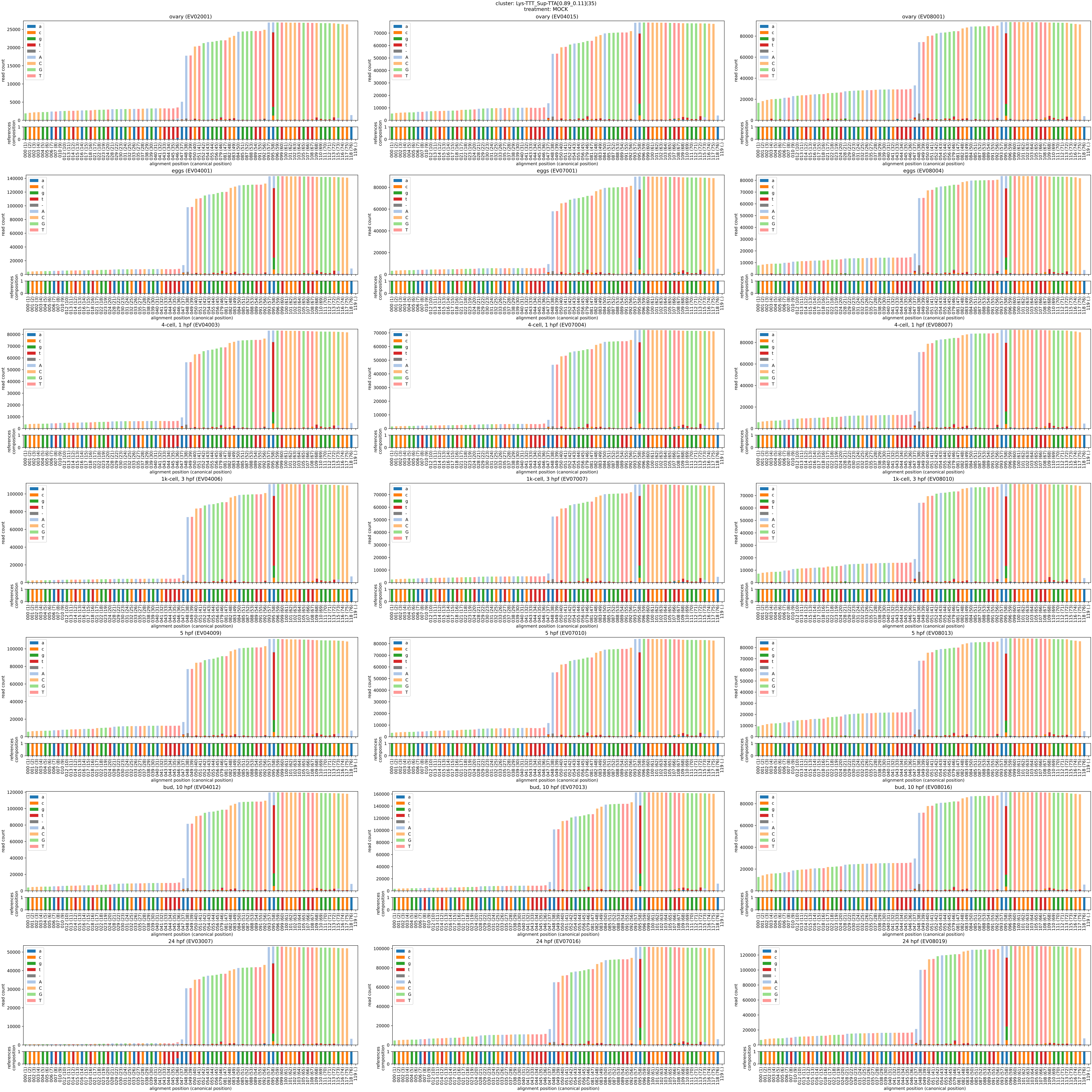
cluster: Lys-TTT_Sup-TTA[0.89_0.11](35)
treatment: MOCK
ovary (EV02001)
ovary (EV04015)
ovary (EV08001)
eggs (EV04001)
eggs (EV07001)
eggs (EV08004)
4-cell, 1 hpf (EV04003)
4-cell, 1 hpf (EV07004)
4-cell, 1 hpf (EV08007)
1k-cell, 3 hpf (EV04006)
1k-cell, 3 hpf (EV07007)
1k-cell, 3 hpf (EV08010)
5 hpf (EV04009)
5 hpf (EV07010)
5 hpf (EV08013)
bud, 10 hpf (EV04012)
bud, 10 hpf (EV07013)
bud, 10 hpf (EV08016)
24 hpf (EV03007)
24 hpf (EV07016)
24 hpf (EV08019)
read count
references composition
alignment position (canonical position)
a
c
g
t
-
A
C
G
T

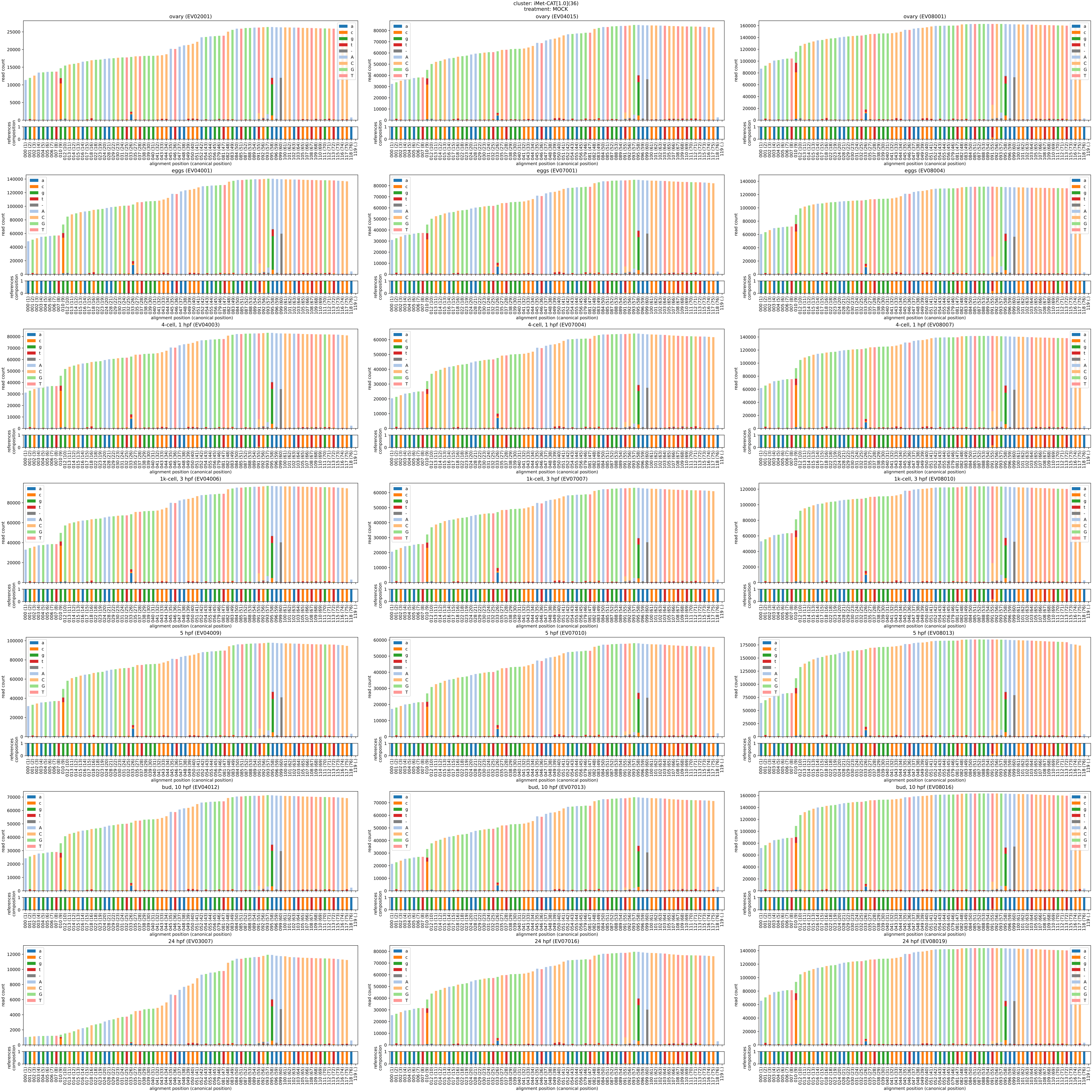
cluster: iMet-CAT[1.0](36)
treatment: MOCK
ovary (EV02001)
ovary (EV04015)
ovary (EV08001)
eggs (EV04001)
eggs (EV07001)
eggs (EV08004)
4-cell, 1 hpf (EV04003)
4-cell, 1 hpf (EV07004)
4-cell, 1 hpf (EV08007)
1k-cell, 3 hpf (EV04006)
1k-cell, 3 hpf (EV07007)
1k-cell, 3 hpf (EV08010)
5 hpf (EV04009)
5 hpf (EV07010)
5 hpf (EV08013)
bud, 10 hpf (EV04012)
bud, 10 hpf (EV07013)
bud, 10 hpf (EV08016)
24 hpf (EV03007)
24 hpf (EV07016)
24 hpf (EV08019)
read count
references composition
alignment position (canonical position)
a
c
g
t
-
A
C
G
T

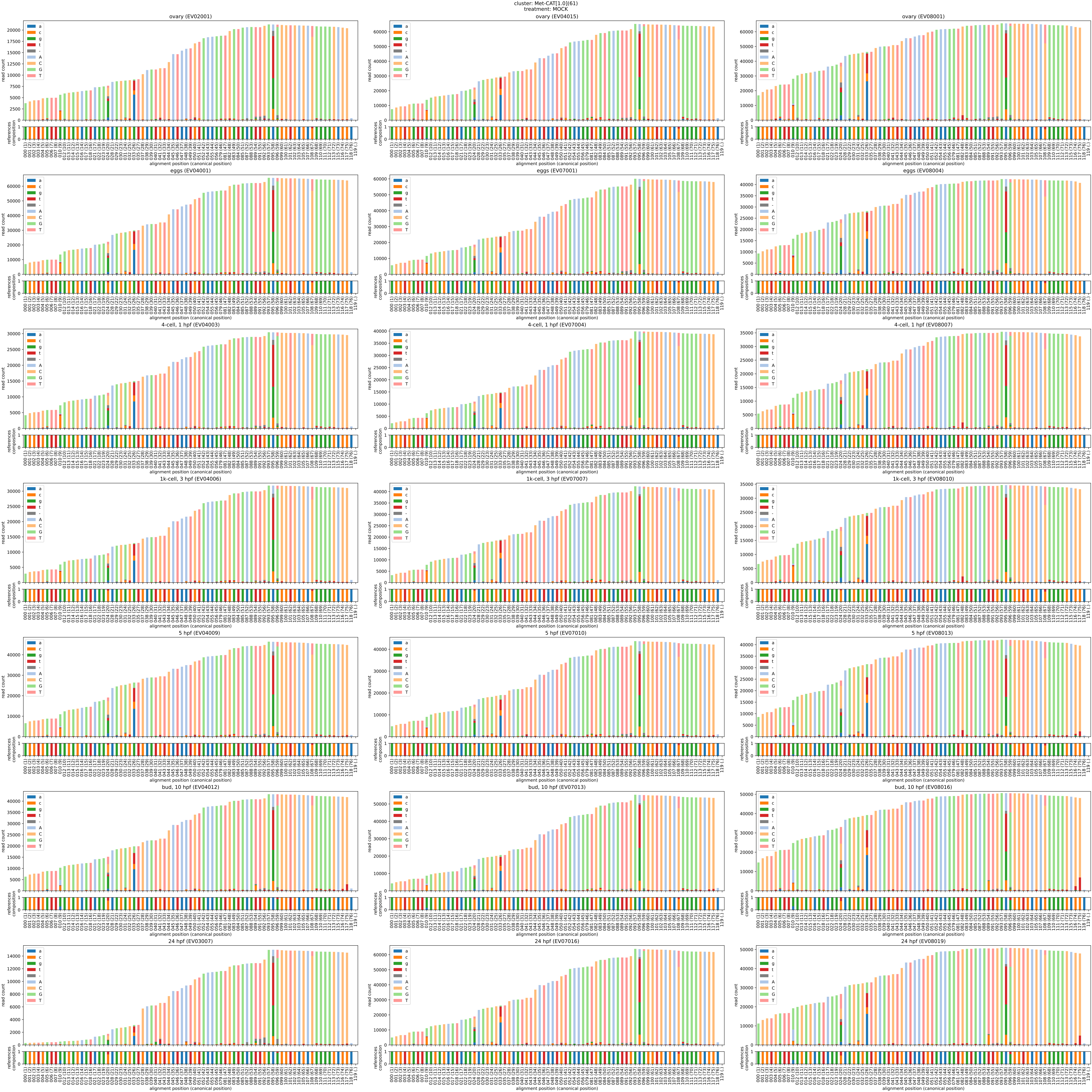

cluster: Met-CAT[1.0](61)
treatment: MOCK
ovary (EV02001)
ovary (EV04015)
ovary (EV08001)
eggs (EV04001)
eggs (EV07001)
eggs (EV08004)
4-cell, 1 hpf (EV04003)
4-cell, 1 hpf (EV07004)
4-cell, 1 hpf (EV08007)
1k-cell, 3 hpf (EV04006)
1k-cell, 3 hpf (EV07007)
1k-cell, 3 hpf (EV08010)
5 hpf (EV04009)
5 hpf (EV07010)
5 hpf (EV08013)
bud, 10 hpf (EV04012)
bud, 10 hpf (EV07013)
bud, 10 hpf (EV08016)
24 hpf (EV03007)
24 hpf (EV07016)
24 hpf (EV08019)
read count
references composition
alignment position (canonical position)
a
c
g
t
-
A
C
G
T

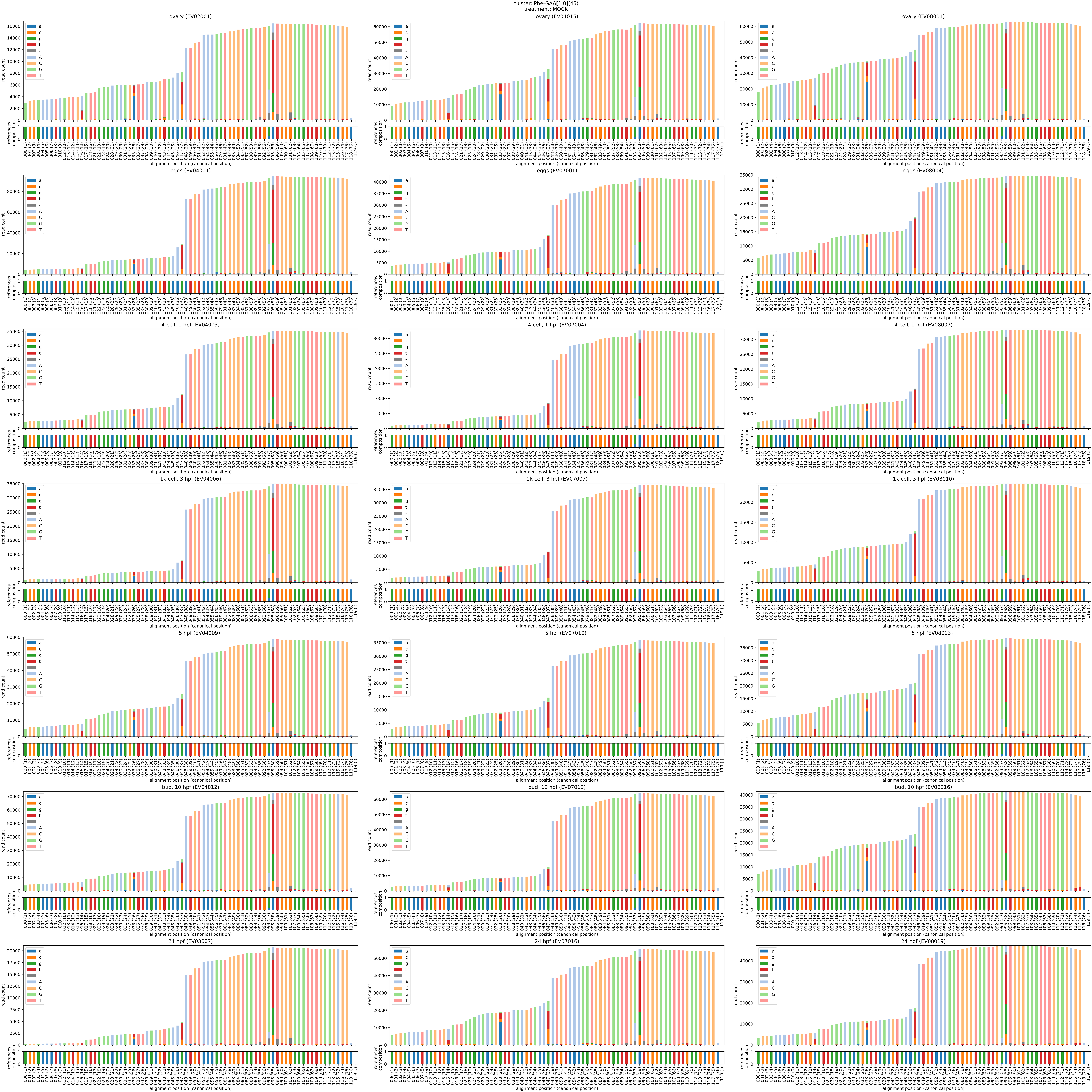
cluster: Phe-GAA[1.0](45)
treatment: MOCK
ovary (EV02001)
ovary (EV04015)
ovary (EV08001)
eggs (EV04001)
eggs (EV07001)
eggs (EV08004)
4-cell, 1 hpf (EV04003)
4-cell, 1 hpf (EV07004)
4-cell, 1 hpf (EV08007)
1k-cell, 3 hpf (EV04006)
1k-cell, 3 hpf (EV07007)
1k-cell, 3 hpf (EV08010)
5 hpf (EV04009)
5 hpf (EV07010)
5 hpf (EV08013)
bud, 10 hpf (EV04012)
bud, 10 hpf (EV07013)
bud, 10 hpf (EV08016)
24 hpf (EV03007)
24 hpf (EV07016)
24 hpf (EV08019)
read count
references composition
alignment position (canonical position)
a
c
g
t
-
A
C
G
T

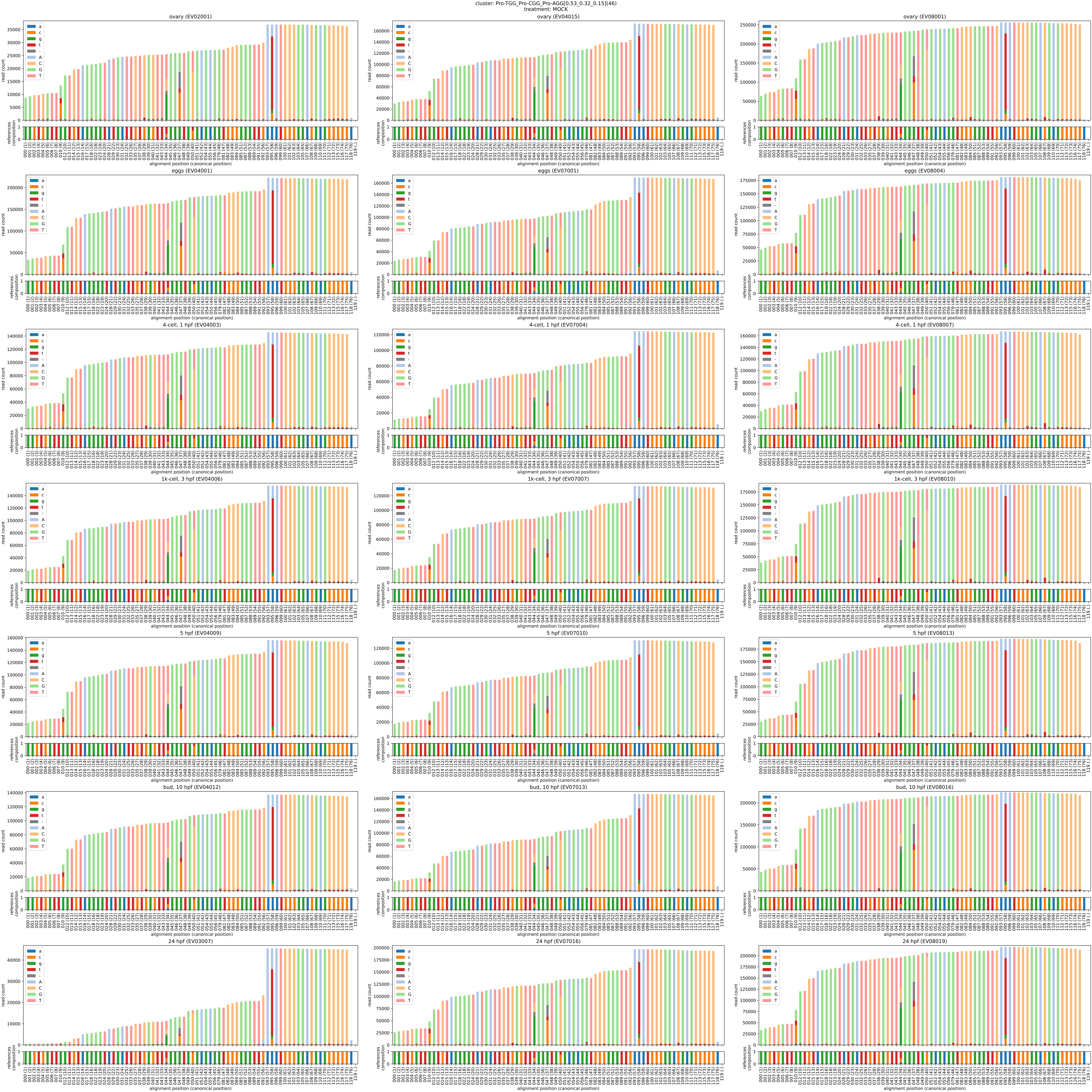
cluster: Pro-TGG_Pro-CGG_Pro-AGG[0.53_0.32_0.15](46)
treatment: MOCK
ovary (EV02001)
ovary (EV04015)
ovary (EV08001)
eggs (EV04001)
eggs (EV07001)
eggs (EV08004)
4-cell, 1 hpf (EV04003)
4-cell, 1 hpf (EV07004)
4-cell, 1 hpf (EV08007)
1k-cell, 3 hpf (EV04006)
1k-cell, 3 hpf (EV07007)
1k-cell, 3 hpf (EV08010)
5 hpf (EV04009)
5 hpf (EV07010)
5 hpf (EV08013)
bud, 10 hpf (EV04012)
bud, 10 hpf (EV07013)
bud, 10 hpf (EV08016)
24 hpf (EV03007)
24 hpf (EV07016)
24 hpf (EV08019)
read count
references composition
alignment position (canonical position)
a
c
g
t
-
A
C
G
T

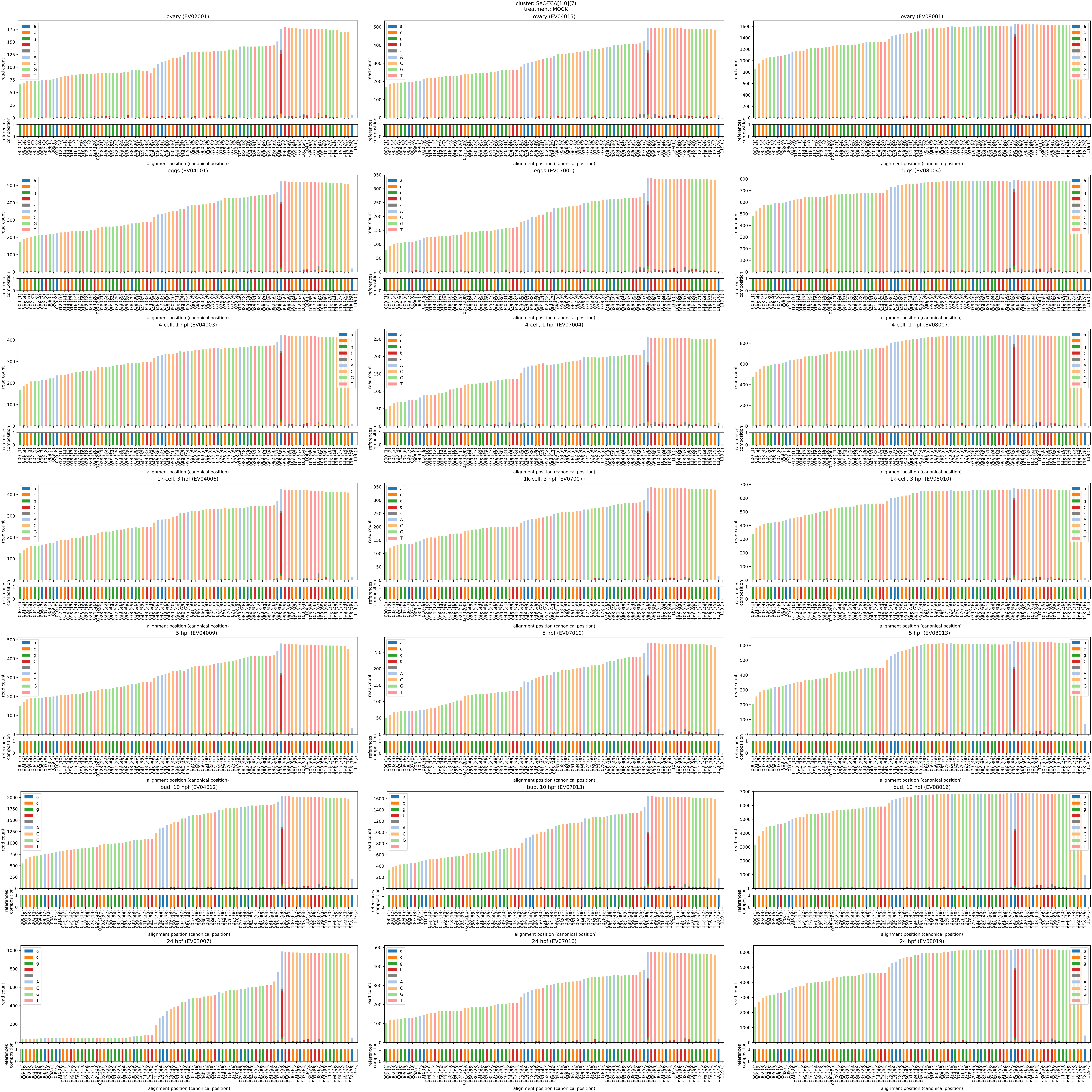
cluster: SeC-TCA[1.0](7)
treatment: MOCK
ovary (EV02001)
ovary (EV04015)
ovary (EV08001)
eggs (EV04001)
eggs (EV07001)
eggs (EV08004)
4-cell, 1 hpf (EV04003)
4-cell, 1 hpf (EV07004)
4-cell, 1 hpf (EV08007)
1k-cell, 3 hpf (EV04006)
1k-cell, 3 hpf (EV07007)
1k-cell, 3 hpf (EV08010)
5 hpf (EV04009)
5 hpf (EV07010)
5 hpf (EV08013)
bud, 10 hpf (EV04012)
bud, 10 hpf (EV07013)
bud, 10 hpf (EV08016)
24 hpf (EV03007)
24 hpf (EV07016)
24 hpf (EV08019)
read count
references composition
alignment position (canonical position)

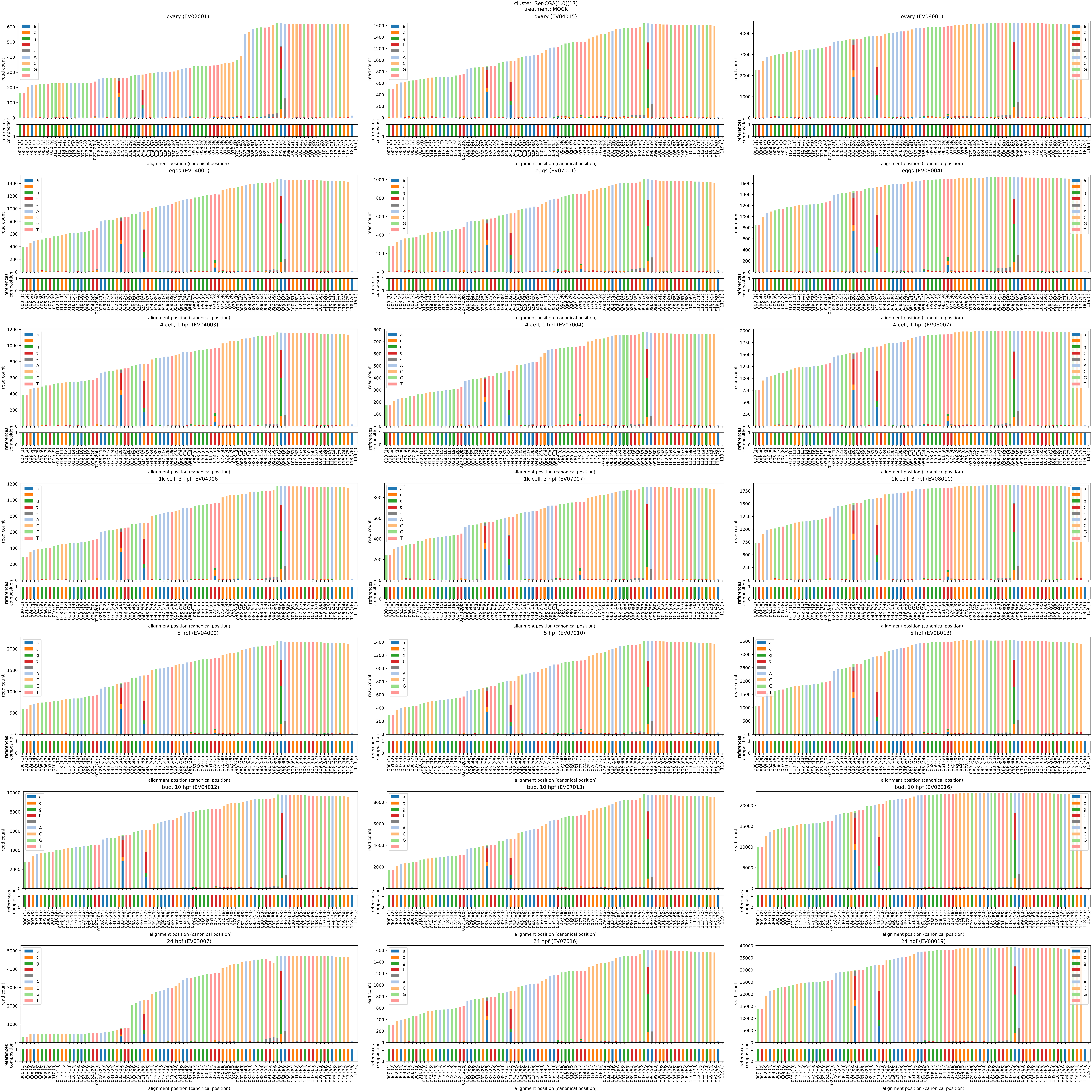
cluster: Ser-CGA[1.0](17)
treatment: MOCK
ovary (EV02001)
ovary (EV04015)
ovary (EV08001)
eggs (EV04001)
eggs (EV07001)
eggs (EV08004)
4-cell, 1 hpf (EV04003)
4-cell, 1 hpf (EV07004)
4-cell, 1 hpf (EV08007)
1k-cell, 3 hpf (EV04006)
1k-cell, 3 hpf (EV07007)
1k-cell, 3 hpf (EV08010)
5 hpf (EV04009)
5 hpf (EV07010)
5 hpf (EV08013)
bud, 10 hpf (EV04012)
bud, 10 hpf (EV07013)
bud, 10 hpf (EV08016)
24 hpf (EV03007)
24 hpf (EV07016)
24 hpf (EV08019)
read count
references composition
alignment position (canonical position)
a
c
g
t
-
A
C
G
T

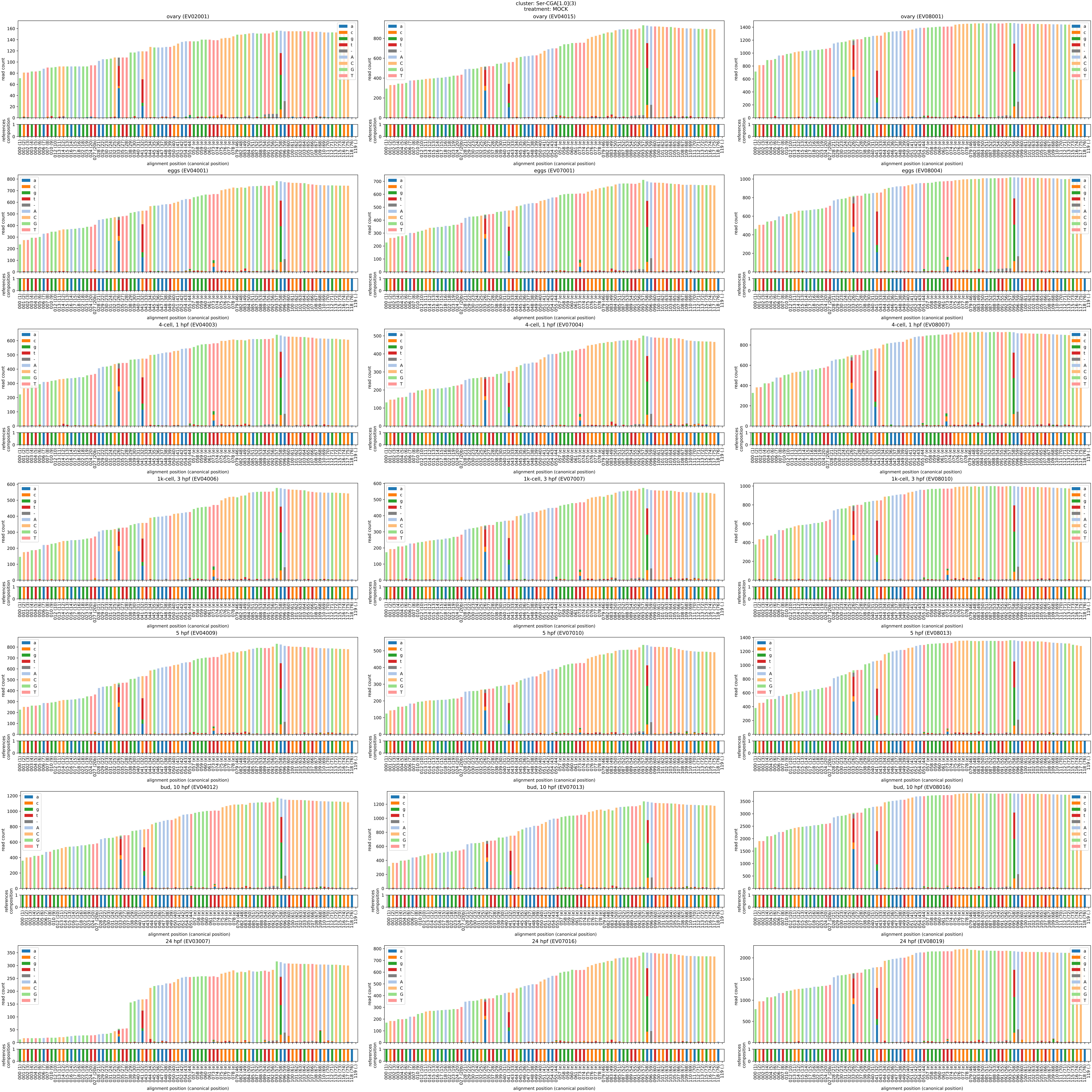
cluster: Ser-CGA[1.0](3)
treatment: MOCK
ovary (EV02001)
ovary (EV04015)
ovary (EV08001)
eggs (EV04001)
eggs (EV07001)
eggs (EV08004)
4-cell, 1 hpf (EV04003)
4-cell, 1 hpf (EV07004)
4-cell, 1 hpf (EV08007)
1k-cell, 3 hpf (EV04006)
1k-cell, 3 hpf (EV07007)
1k-cell, 3 hpf (EV08010)
5 hpf (EV04009)
5 hpf (EV07010)
5 hpf (EV08013)
bud, 10 hpf (EV04012)
bud, 10 hpf (EV07013)
bud, 10 hpf (EV08016)
24 hpf (EV03007)
24 hpf (EV07016)
24 hpf (EV08019)
read count
references composition
alignment position (canonical position)
a
c
g
t
-
A
C
G
T

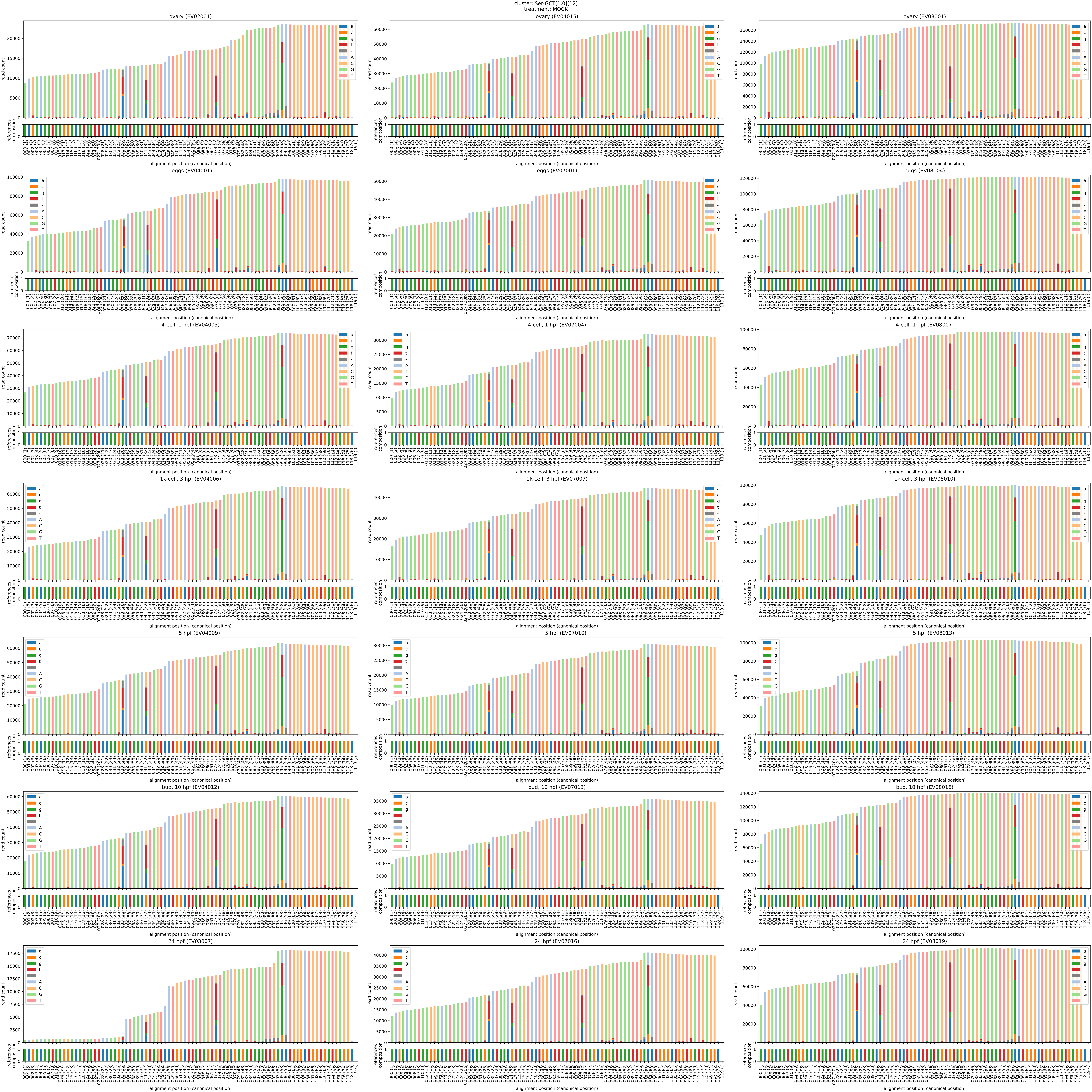
cluster: Ser-GCT[1.0](12)
treatment: MOCK
ovary (EV02001)
ovary (EV04015)
ovary (EV08001)
eggs (EV04001)
eggs (EV07001)
eggs (EV08004)
4-cell, 1 hpf (EV04003)
4-cell, 1 hpf (EV07004)
4-cell, 1 hpf (EV08007)
1k-cell, 3 hpf (EV04006)
1k-cell, 3 hpf (EV07007)
1k-cell, 3 hpf (EV08010)
5 hpf (EV04009)
5 hpf (EV07010)
5 hpf (EV08013)
bud, 10 hpf (EV04012)
bud, 10 hpf (EV07013)
bud, 10 hpf (EV08016)
24 hpf (EV03007)
24 hpf (EV07016)
24 hpf (EV08019)
read count
references composition
alignment position (canonical position)
a
c
g
t
-
A
C
G
T

cluster: Ser-TGA[1.0](25)
treatment: MOCK

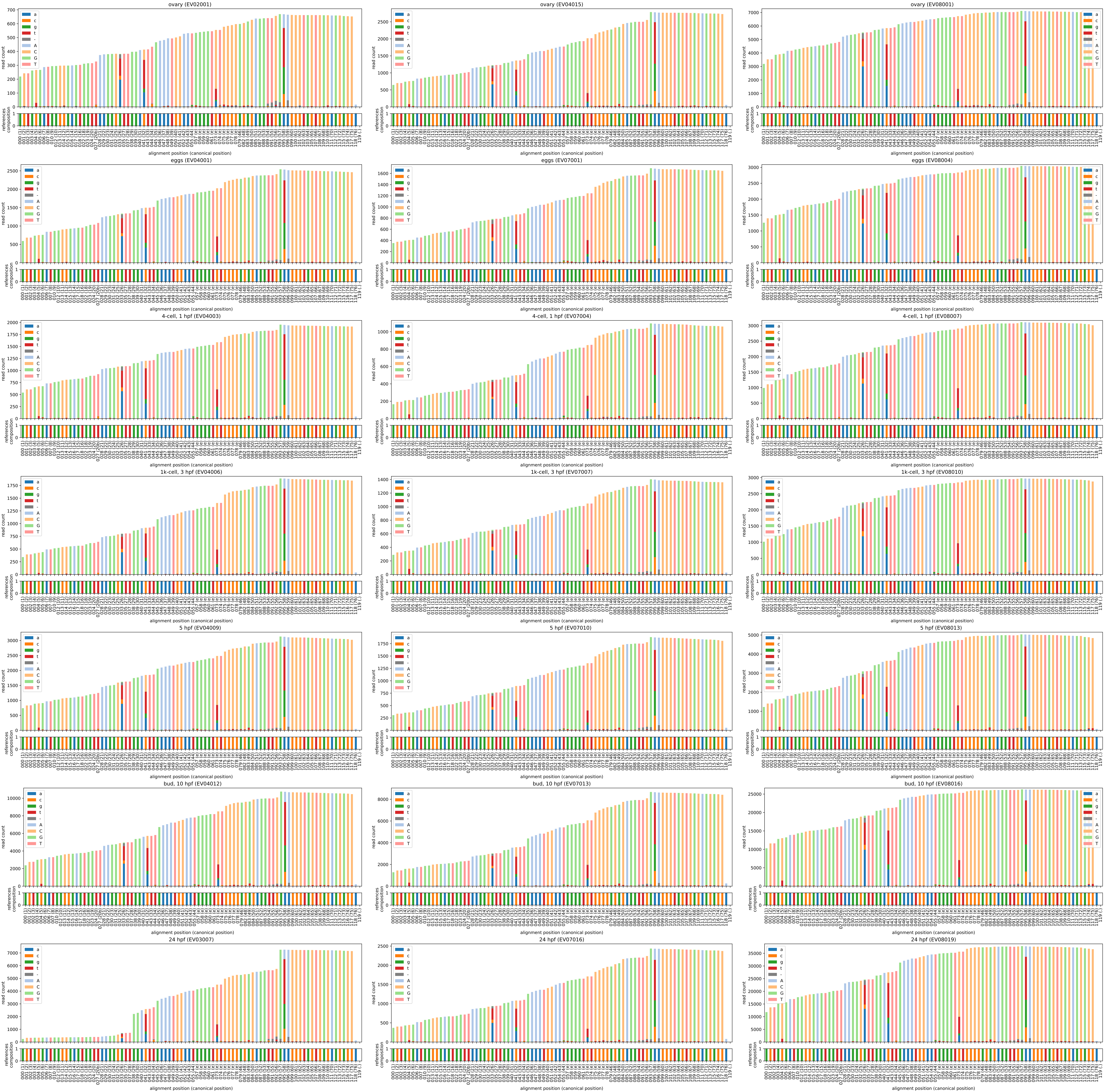

cluster: Ser-TGA_Ser-AGA_Ser-CGA[0.48_0.3_0.22](64)
treatment: MOCK

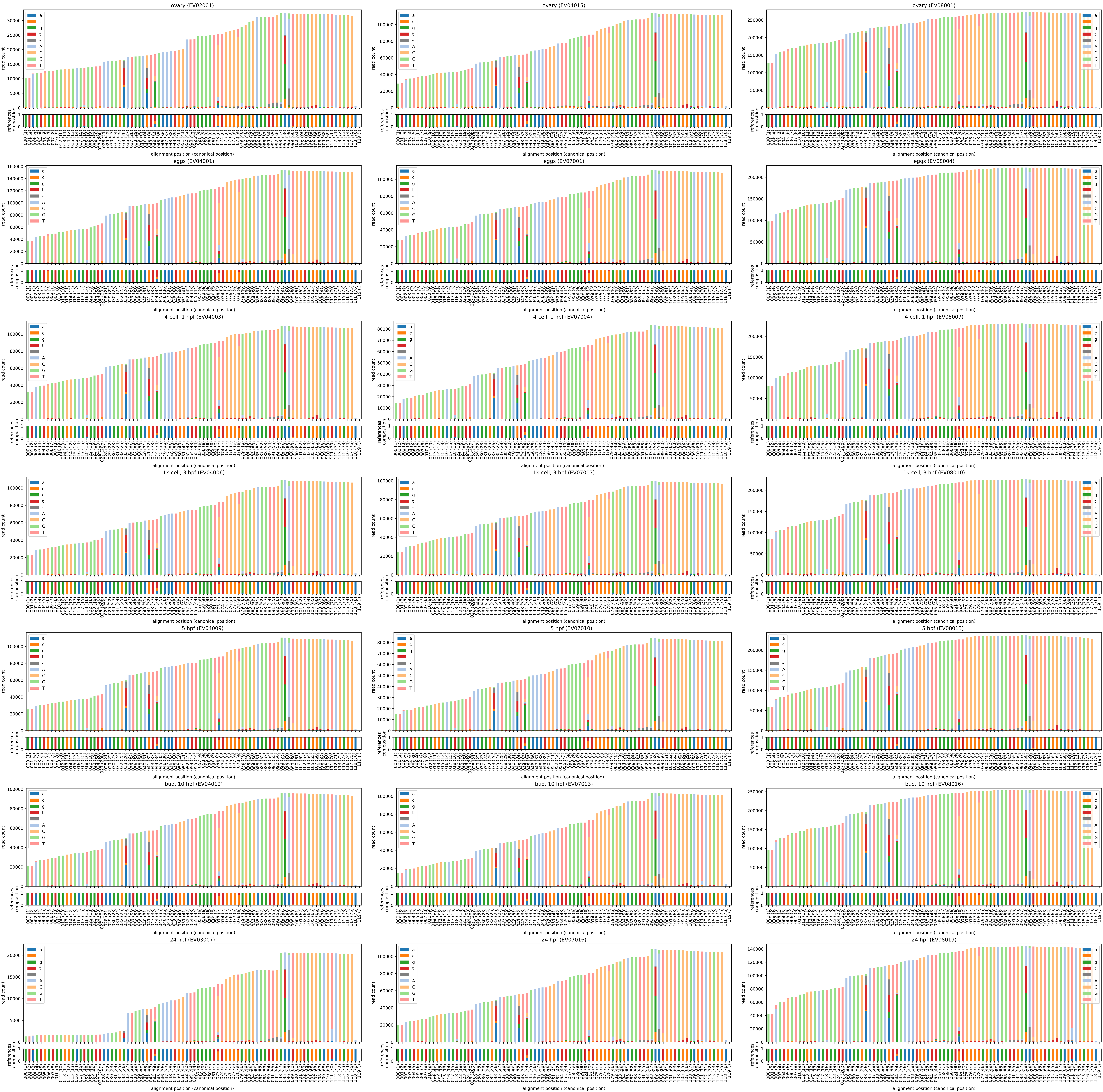

cluster: Thr-AGT_Thr-CGT_Thr-TGT[0.34_0.33_0.33](62)
treatment: MOCK

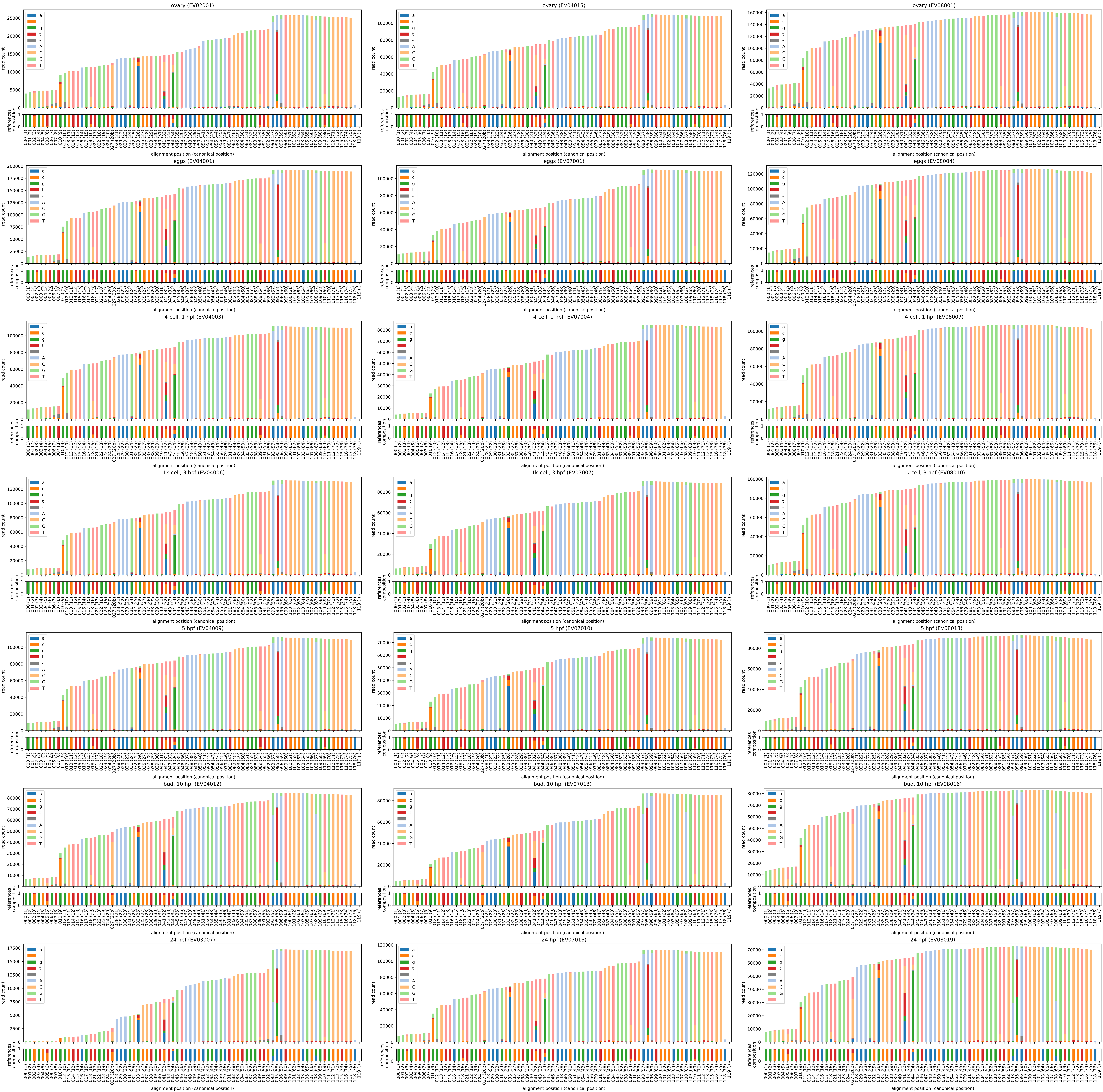

cluster: Thr-CGT[1.0](8)
treatment: MOCK

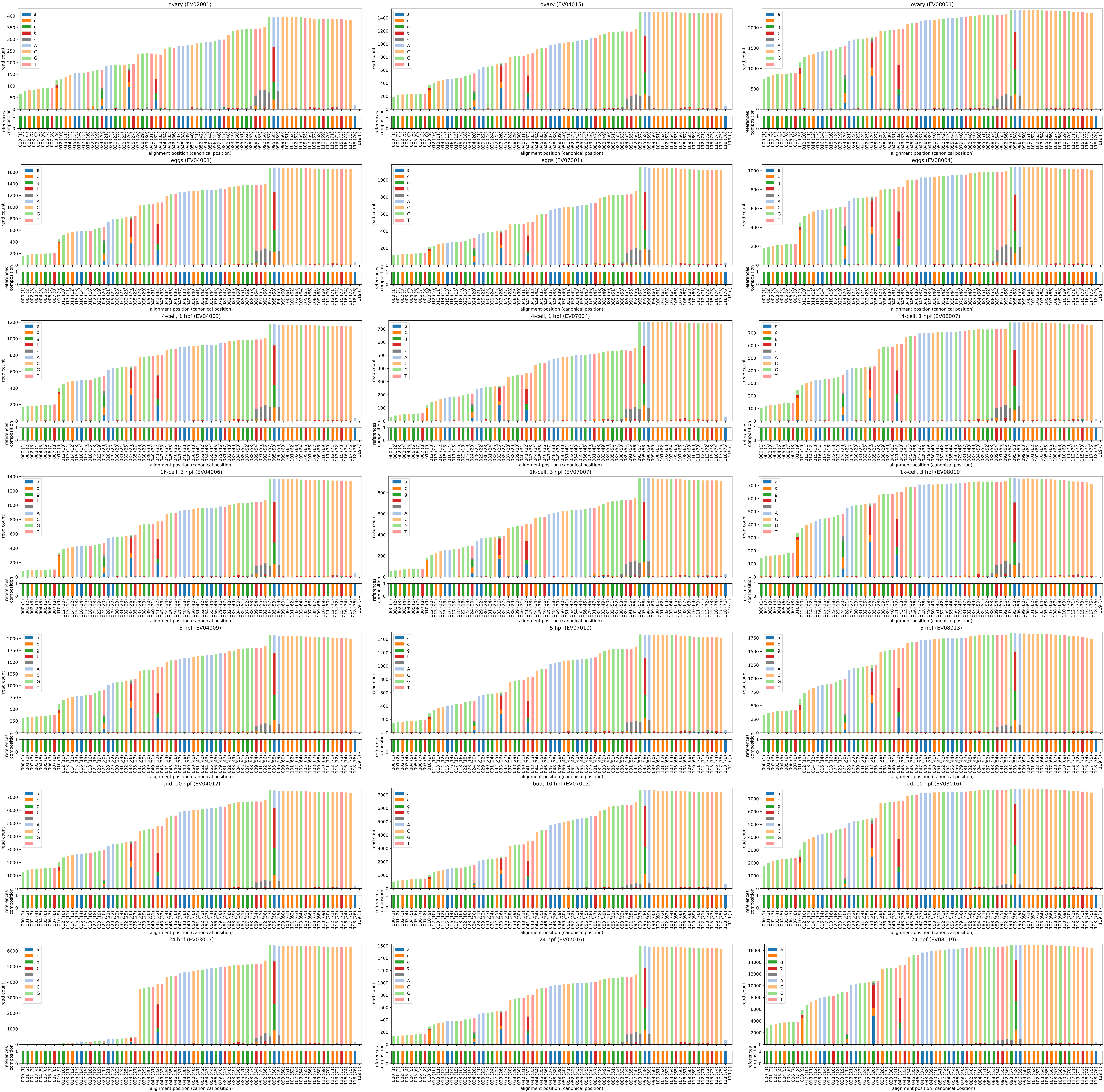

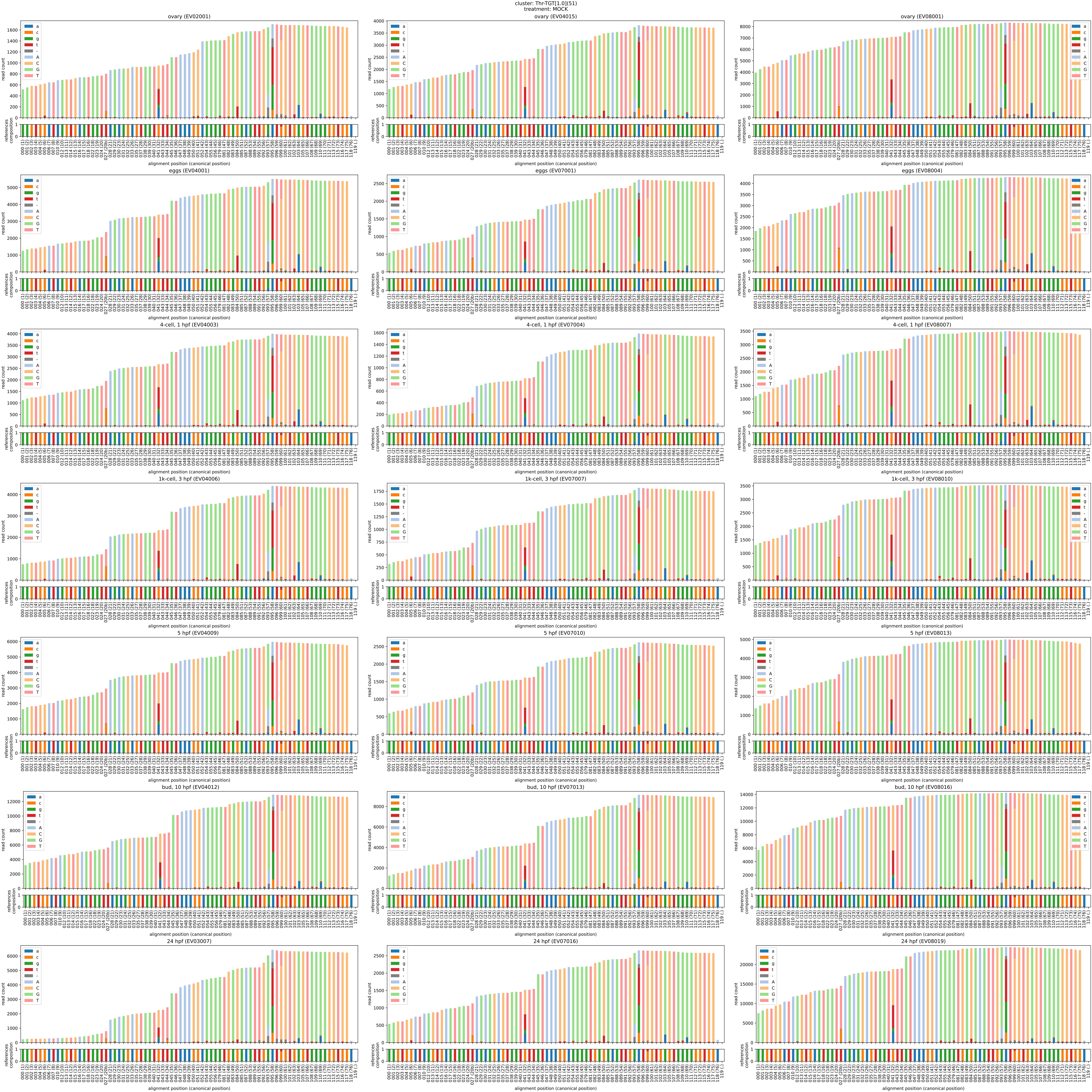
cluster: Thr-TGT[1.0](51)
treatment: MOCK
ovary (EV02001)
ovary (EV04015)
ovary (EV08001)
eggs (EV04001)
eggs (EV07001)
eggs (EV08004)
4-cell, 1 hpf (EV04003)
4-cell, 1 hpf (EV07004)
4-cell, 1 hpf (EV08007)
1k-cell, 3 hpf (EV04006)
1k-cell, 3 hpf (EV07007)
1k-cell, 3 hpf (EV08010)
5 hpf (EV04009)
5 hpf (EV07010)
5 hpf (EV08013)
bud, 10 hpf (EV04012)
bud, 10 hpf (EV07013)
bud, 10 hpf (EV08016)
24 hpf (EV03007)
24 hpf (EV07016)
24 hpf (EV08019)
read count
references composition
alignment position (canonical position)
a
c
g
t
-
A
C
G
T

cluster: Trp-CCA[1.0](11)
treatment: MOCK

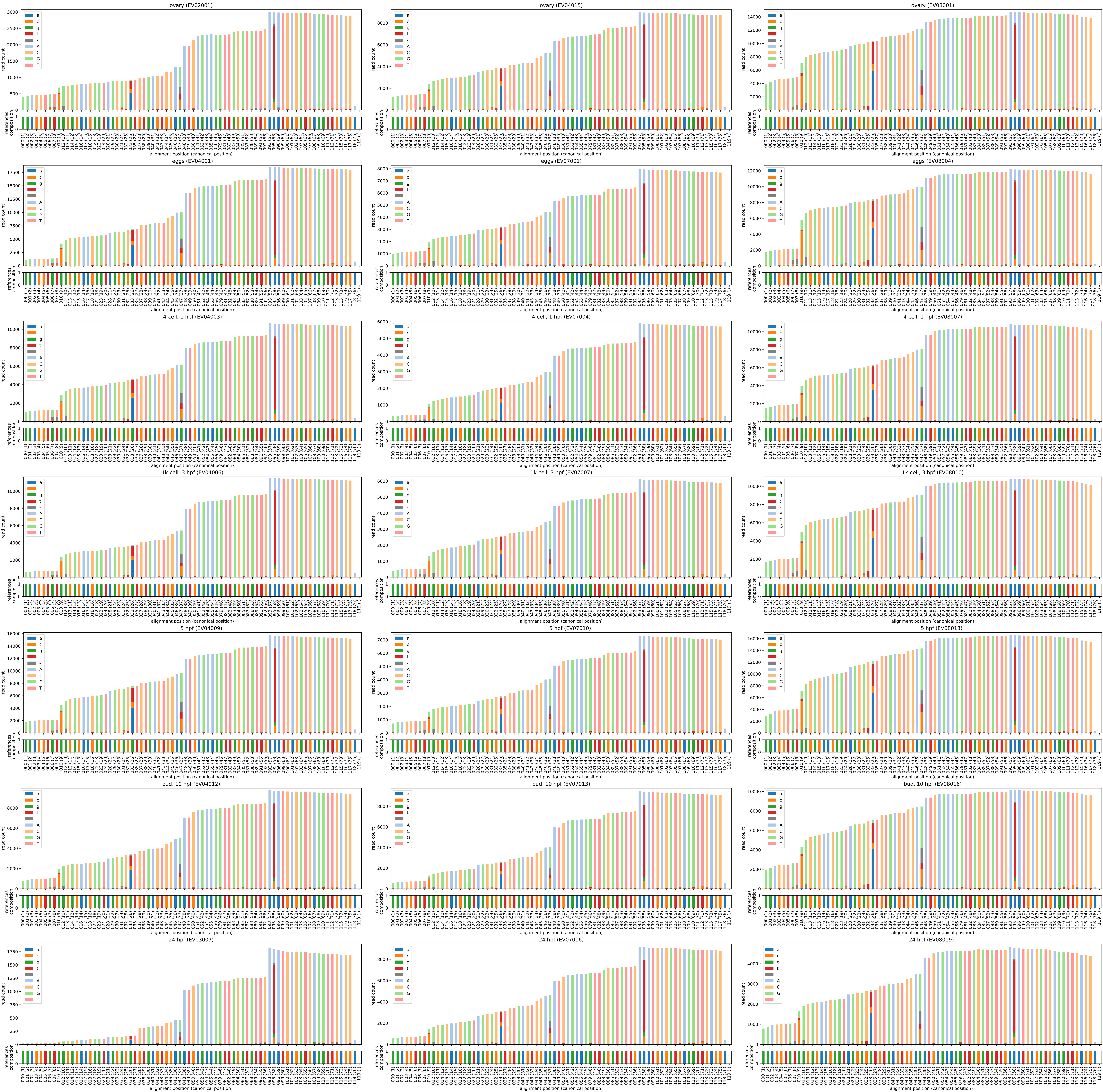

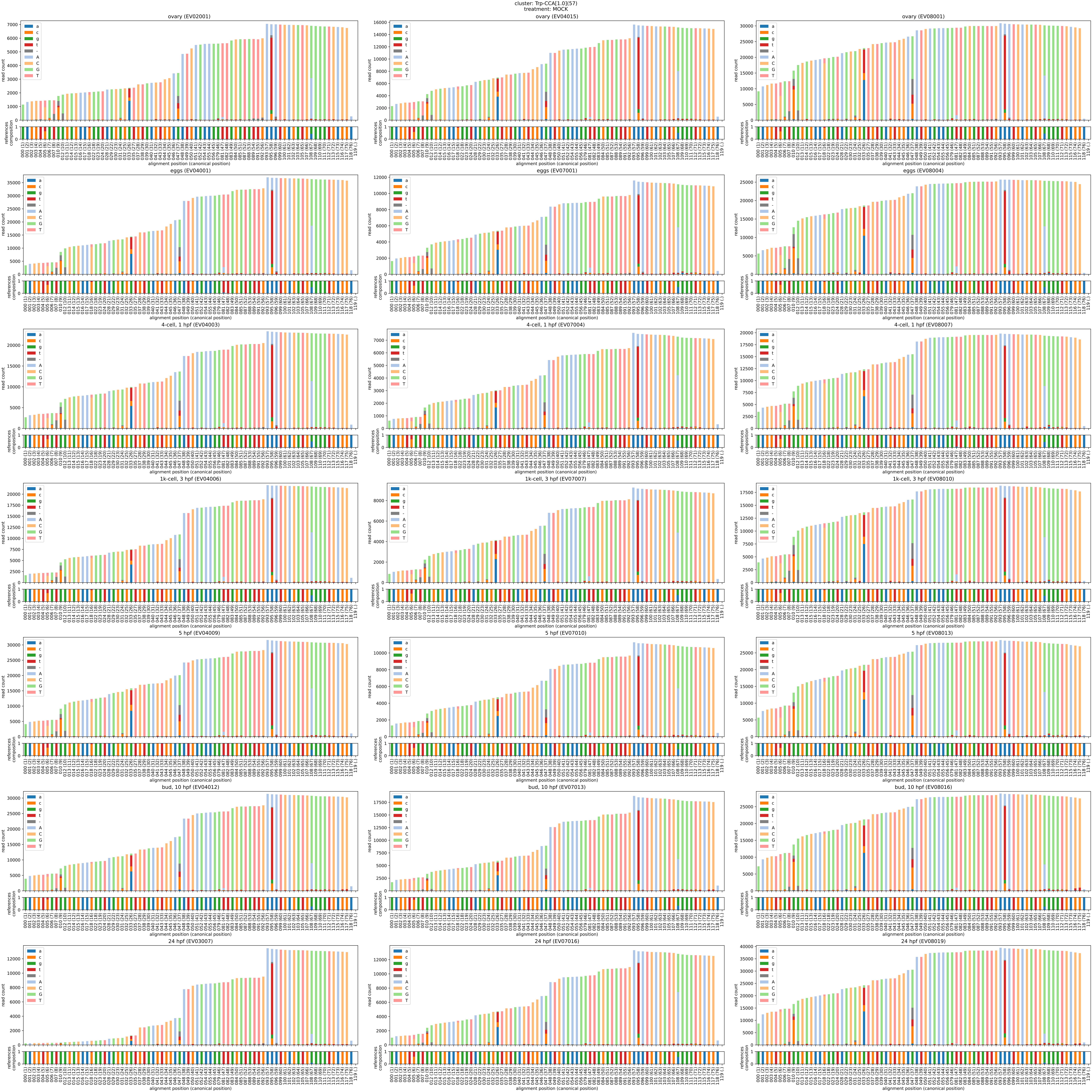
cluster: Trp-CCA[1.0](57)
treatment: MOCK
ovary (EV02001)
ovary (EV04015)
ovary (EV08001)
eggs (EV04001)
eggs (EV07001)
eggs (EV08004)
4-cell, 1 hpf (EV04003)
4-cell, 1 hpf (EV07004)
4-cell, 1 hpf (EV08007)
1k-cell, 3 hpf (EV04006)
1k-cell, 3 hpf (EV07007)
1k-cell, 3 hpf (EV08010)
5 hpf (EV04009)
5 hpf (EV07010)
5 hpf (EV08013)
bud, 10 hpf (EV04012)
bud, 10 hpf (EV07013)
bud, 10 hpf (EV08016)
24 hpf (EV03007)
24 hpf (EV07016)
24 hpf (EV08019)
read count
references composition
alignment position (canonical position)
a
c
g
t
-
A
C
G
T

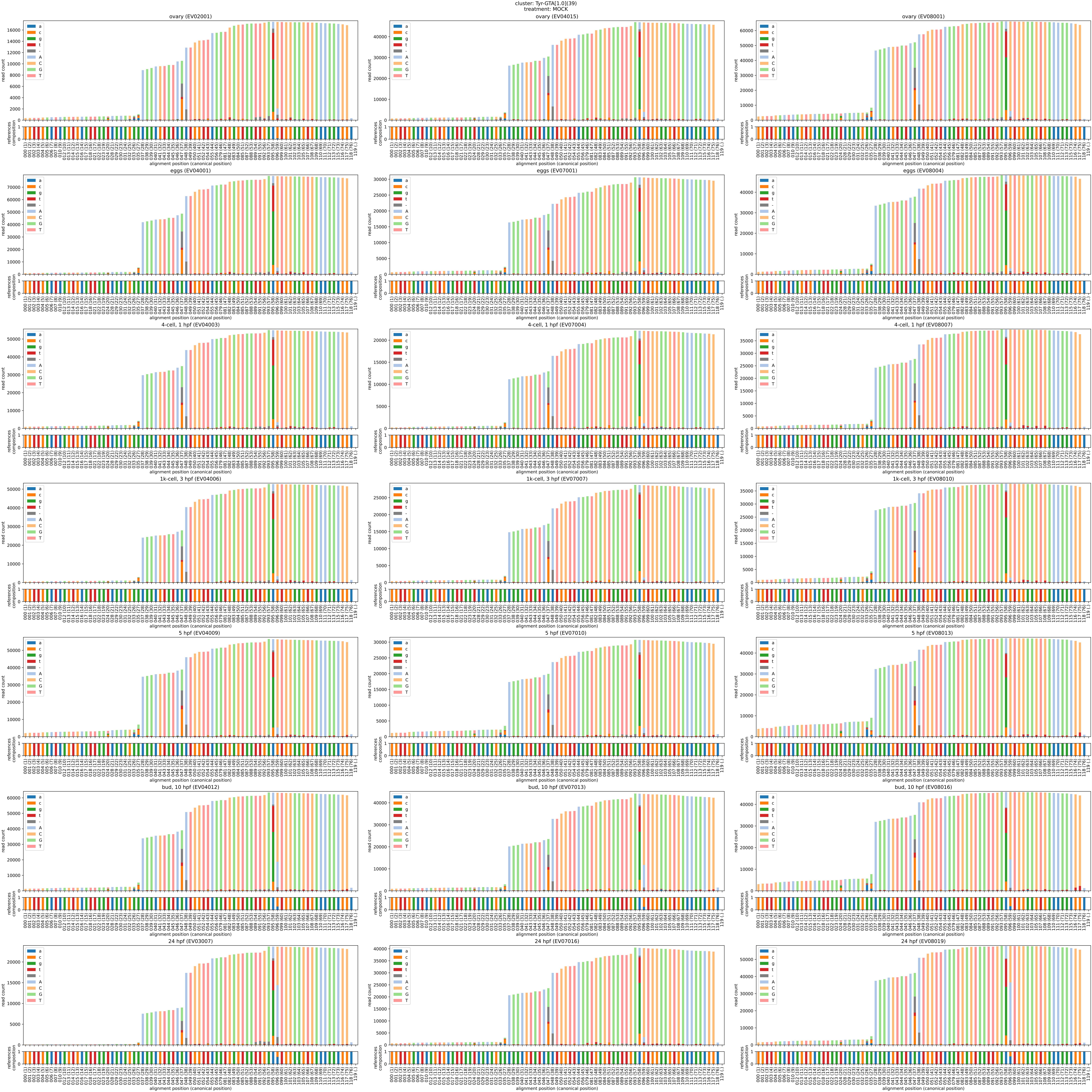

cluster: Tyr-GTA[1.0](39)
treatment: MOCK
ovary (EV02001)
ovary (EV04015)
ovary (EV08001)
eggs (EV04001)
eggs (EV07001)
eggs (EV08004)
4-cell, 1 hpf (EV04003)
4-cell, 1 hpf (EV07004)
4-cell, 1 hpf (EV08007)
1k-cell, 3 hpf (EV04006)
1k-cell, 3 hpf (EV07007)
1k-cell, 3 hpf (EV08010)
5 hpf (EV04009)
5 hpf (EV07010)
5 hpf (EV08013)
bud, 10 hpf (EV04012)
bud, 10 hpf (EV07013)
bud, 10 hpf (EV08016)
24 hpf (EV03007)
24 hpf (EV07016)
24 hpf (EV08019)
read count
references composition
alignment position (canonical position)

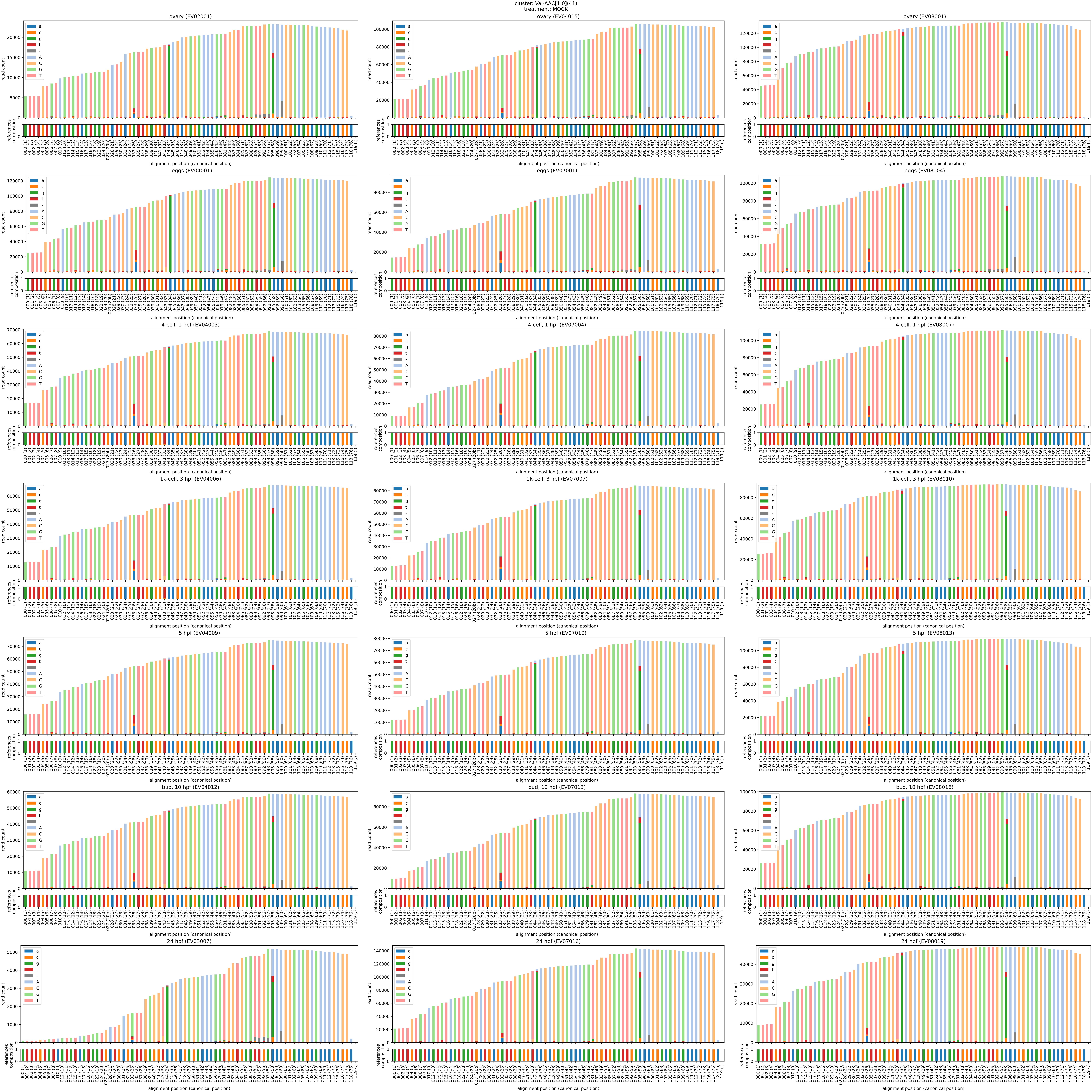
cluster: Val-AAC[1.0](41)
treatment: MOCK
ovary (EV02001)
ovary (EV04015)
ovary (EV08001)
eggs (EV04001)
eggs (EV07001)
eggs (EV08004)
4-cell, 1 hpf (EV04003)
4-cell, 1 hpf (EV07004)
4-cell, 1 hpf (EV08007)
1k-cell, 3 hpf (EV04006)
1k-cell, 3 hpf (EV07007)
1k-cell, 3 hpf (EV08010)
5 hpf (EV04009)
5 hpf (EV07010)
5 hpf (EV08013)
bud, 10 hpf (EV04012)
bud, 10 hpf (EV07013)
bud, 10 hpf (EV08016)
24 hpf (EV03007)
24 hpf (EV07016)
24 hpf (EV08019)
read count
references composition
alignment position (canonical position)
a
c
g
t
-
A
C
G
T

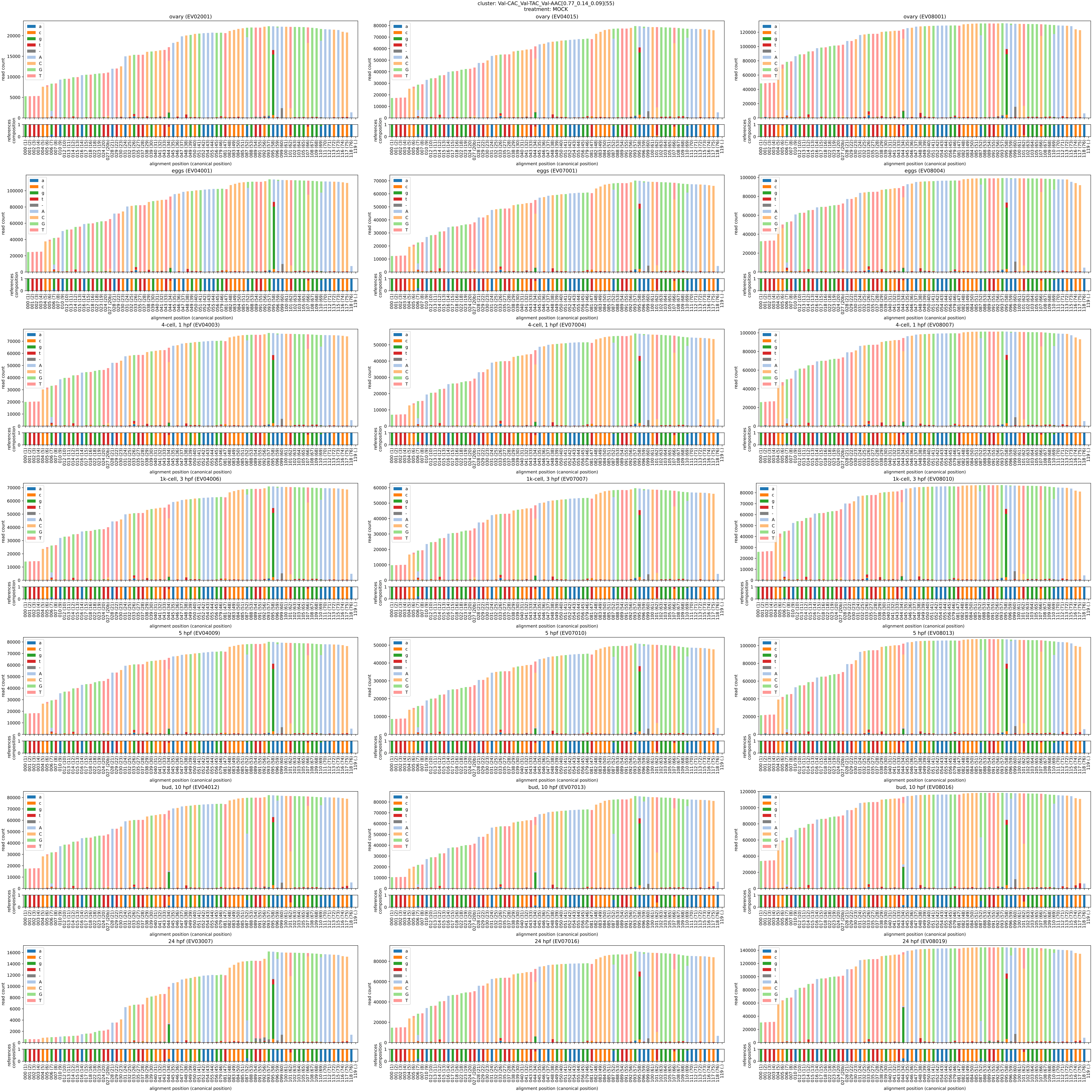
cluster: Val-CAC_Val-TAC_Val-AAC[0.77_0.14_0.09](55)
treatment: MOCK
ovary (EV02001)
ovary (EV04015)
ovary (EV08001)
eggs (EV04001)
eggs (EV07001)
eggs (EV08004)
4-cell, 1 hpf (EV04003)
4-cell, 1 hpf (EV07004)
4-cell, 1 hpf (EV08007)
1k-cell, 3 hpf (EV04006)
1k-cell, 3 hpf (EV07007)
1k-cell, 3 hpf (EV08010)
5 hpf (EV04009)
5 hpf (EV07010)
5 hpf (EV08013)
bud, 10 hpf (EV04012)
bud, 10 hpf (EV07013)
bud, 10 hpf (EV08016)
24 hpf (EV03007)
24 hpf (EV07016)
24 hpf (EV08019)
read count
references composition
alignment position (canonical position)
a
c
g
t
-
A
C
G
T

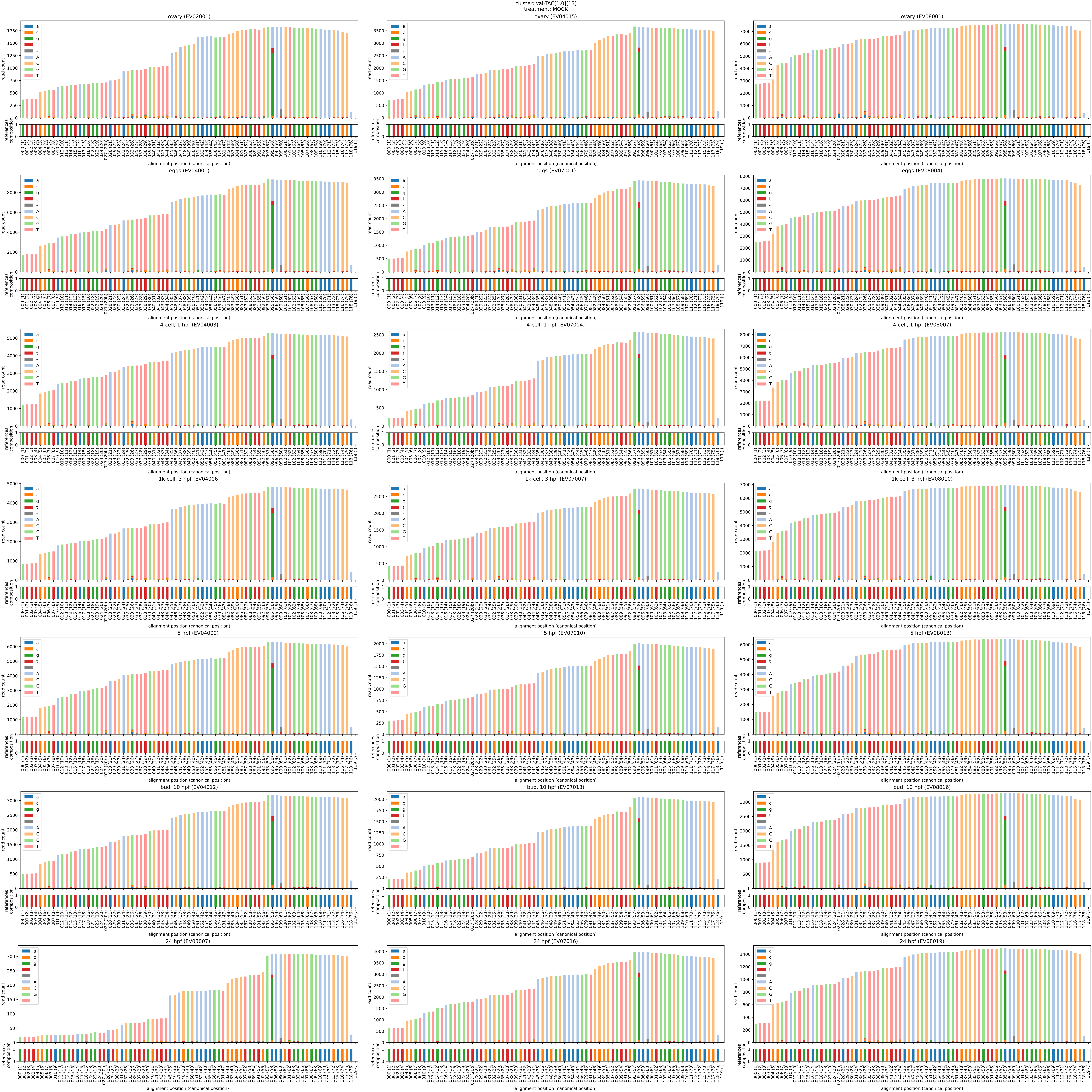
cluster: Val-TAC[1.0](13)
treatment: MOCK
ovary (EV02001)
ovary (EV04015)
ovary (EV08001)
eggs (EV04001)
eggs (EV07001)
eggs (EV08004)
4-cell, 1 hpf (EV04003)
4-cell, 1 hpf (EV07004)
4-cell, 1 hpf (EV08007)
1k-cell, 3 hpf (EV04006)
1k-cell, 3 hpf (EV07007)
1k-cell, 3 hpf (EV08010)
5 hpf (EV04009)
5 hpf (EV07010)
5 hpf (EV08013)
bud, 10 hpf (EV04012)
bud, 10 hpf (EV07013)
bud, 10 hpf (EV08016)
24 hpf (EV03007)
24 hpf (EV07016)
24 hpf (EV08019)
read count
references composition
alignment position (canonical position)
a
c
g
t
-
A
C
G
T

cluster: Val-TAC[1.0](38)
treatment: MOCK

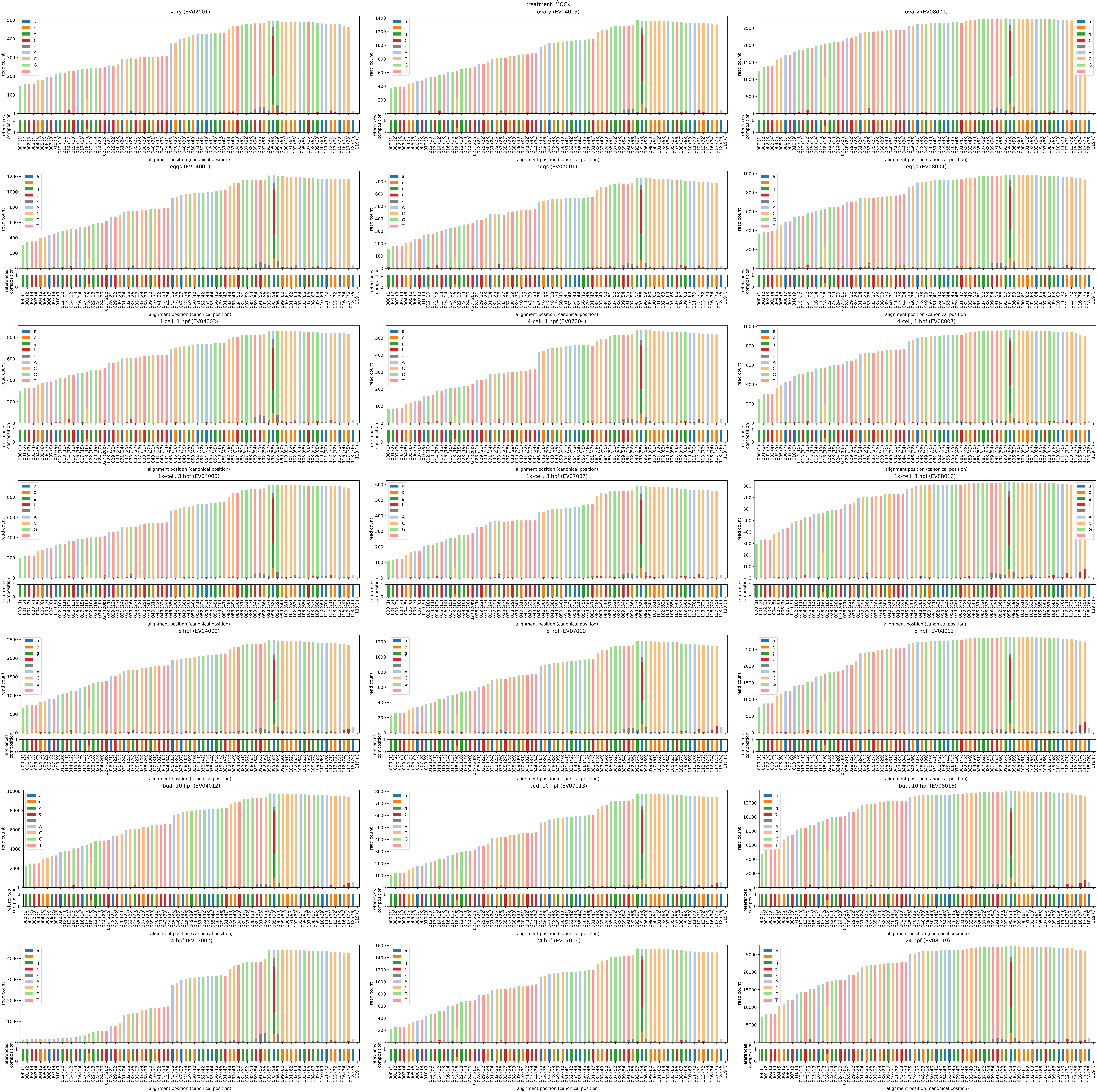

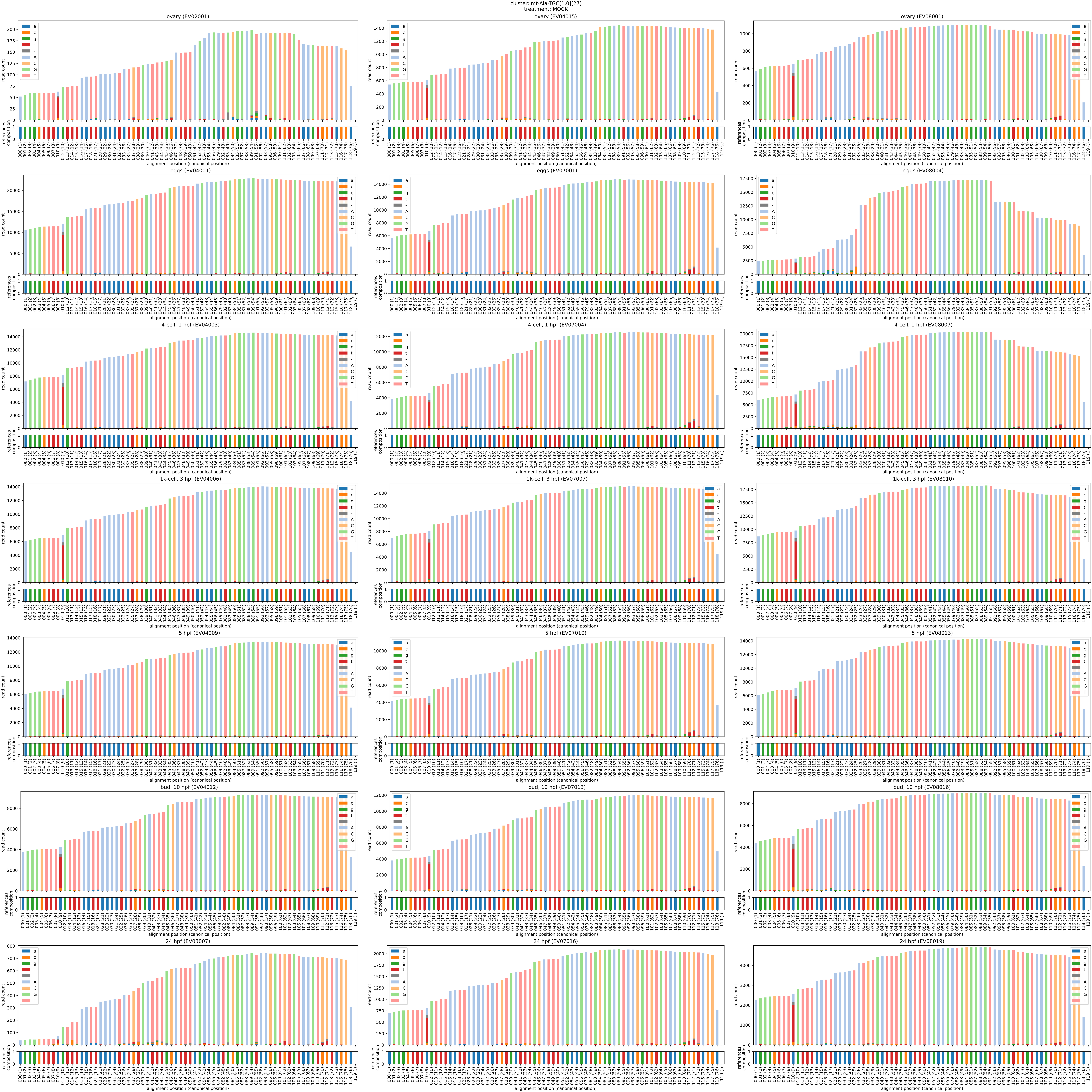
cluster: mt-Ala-TGC[1.0](27)
treatment: MOCK
ovary (EV02001)
ovary (EV04015)
ovary (EV08001)
eggs (EV04001)
eggs (EV07001)
eggs (EV08004)
4-cell, 1 hpf (EV04003)
4-cell, 1 hpf (EV07004)
4-cell, 1 hpf (EV08007)
1k-cell, 3 hpf (EV04006)
1k-cell, 3 hpf (EV07007)
1k-cell, 3 hpf (EV08010)
5 hpf (EV04009)
5 hpf (EV07010)
5 hpf (EV08013)
bud, 10 hpf (EV04012)
bud, 10 hpf (EV07013)
bud, 10 hpf (EV08016)
24 hpf (EV03007)
24 hpf (EV07016)
24 hpf (EV08019)
read count
references composition
alignment position (canonical position)
a
c
g
t
-
A
C
G
T

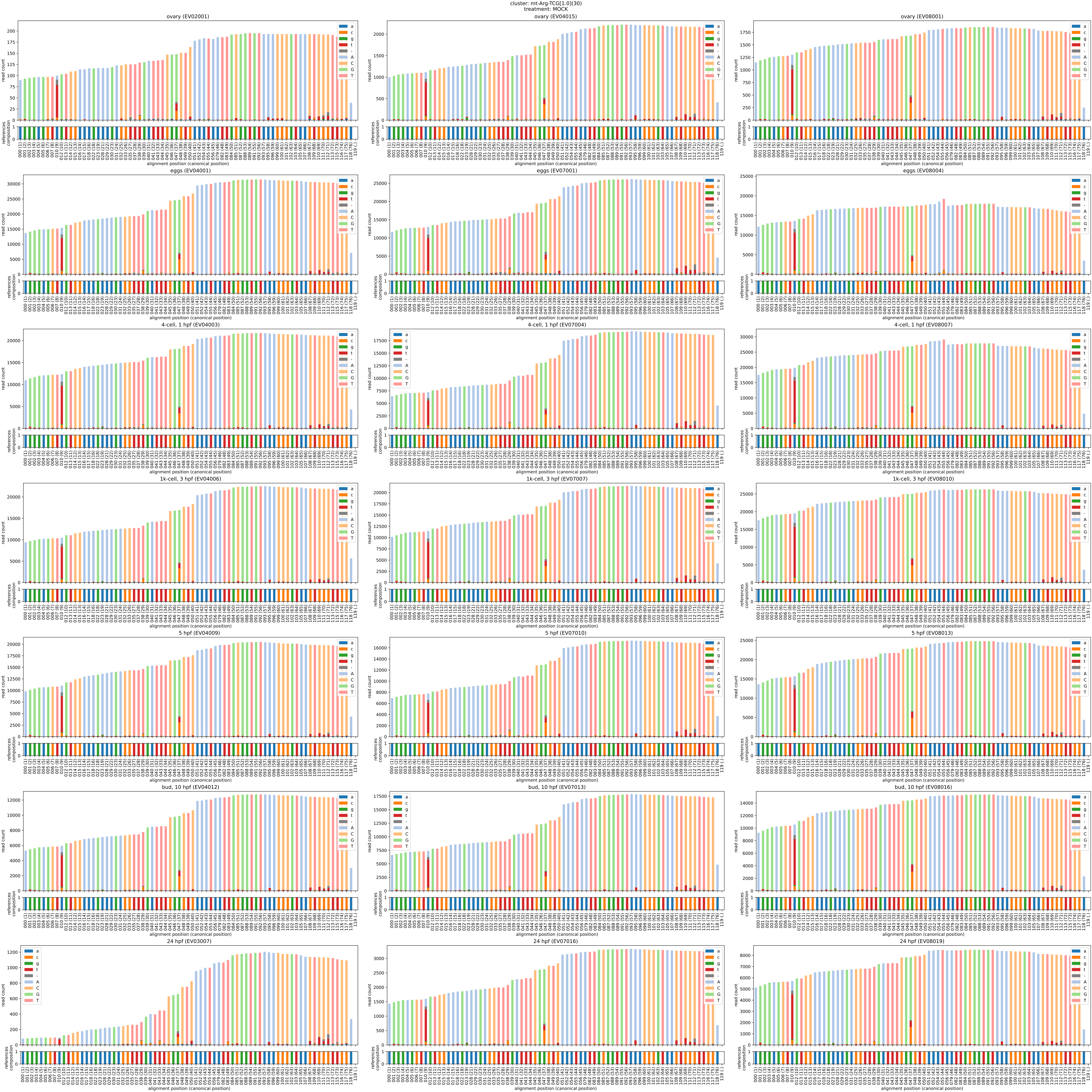
cluster: mt-Arg-TCG[1.0](30)
treatment: MOCK
ovary (EV02001)
ovary (EV04015)
ovary (EV08001)
eggs (EV04001)
eggs (EV07001)
eggs (EV08004)
4-cell, 1 hpf (EV04003)
4-cell, 1 hpf (EV07004)
4-cell, 1 hpf (EV08007)
1k-cell, 3 hpf (EV04006)
1k-cell, 3 hpf (EV07007)
1k-cell, 3 hpf (EV08010)
5 hpf (EV04009)
5 hpf (EV07010)
5 hpf (EV08013)
bud, 10 hpf (EV04012)
bud, 10 hpf (EV07013)
bud, 10 hpf (EV08016)
24 hpf (EV03007)
24 hpf (EV07016)
24 hpf (EV08019)
read count
references composition
alignment position (canonical position)

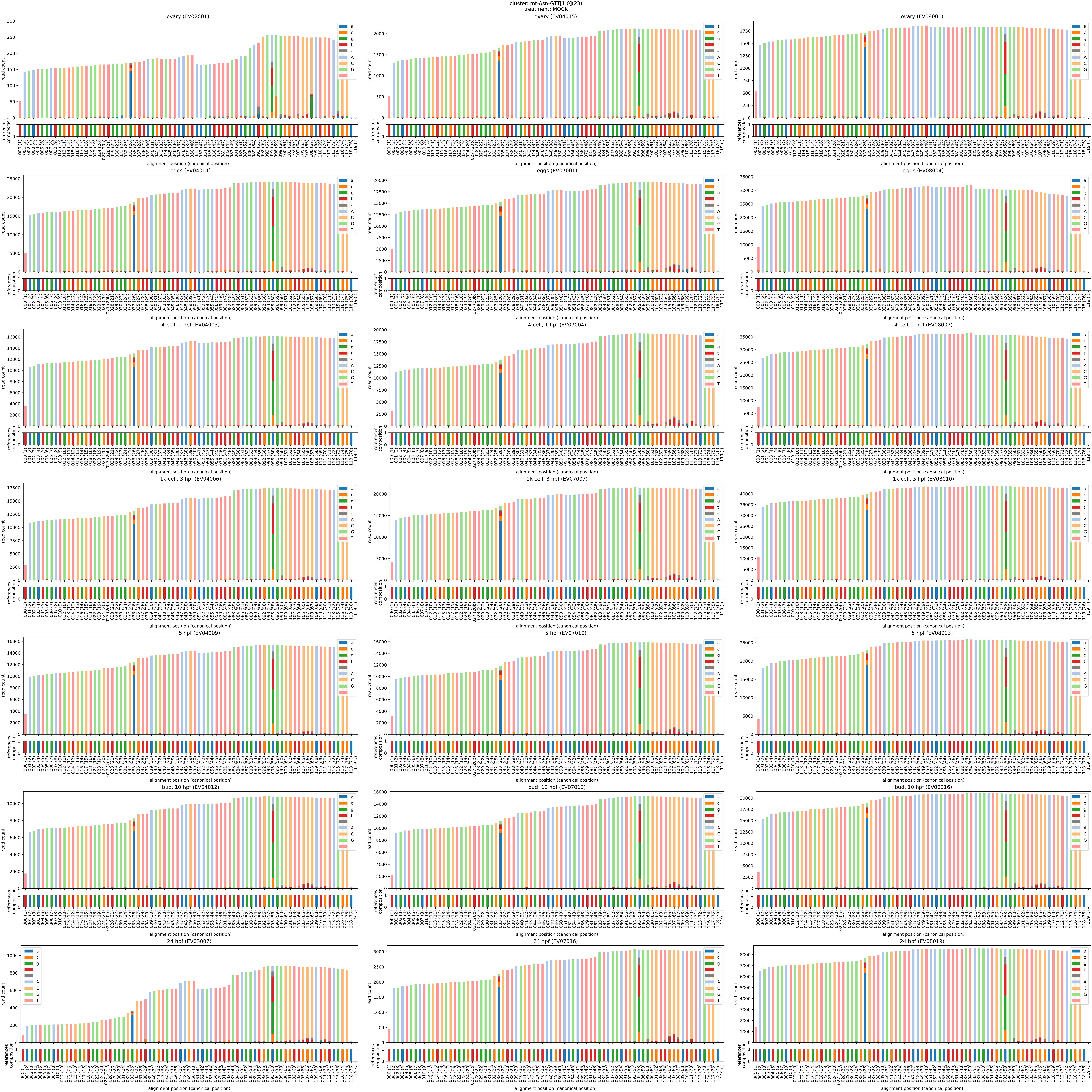
cluster: mt-Asn-GTT[1.0](23)
treatment: MOCK
ovary (EV02001)
ovary (EV04015)
ovary (EV08001)
eggs (EV04001)
eggs (EV07001)
eggs (EV08004)
4-cell, 1 hpf (EV04003)
4-cell, 1 hpf (EV07004)
4-cell, 1 hpf (EV08007)
1k-cell, 3 hpf (EV04006)
1k-cell, 3 hpf (EV07007)
1k-cell, 3 hpf (EV08010)
5 hpf (EV04009)
5 hpf (EV07010)
5 hpf (EV08013)
bud, 10 hpf (EV04012)
bud, 10 hpf (EV07013)
bud, 10 hpf (EV08016)
24 hpf (EV03007)
24 hpf (EV07016)
24 hpf (EV08019)
read count
references composition
alignment position (canonical position)
a
c
g
t
-
A
C
G
T

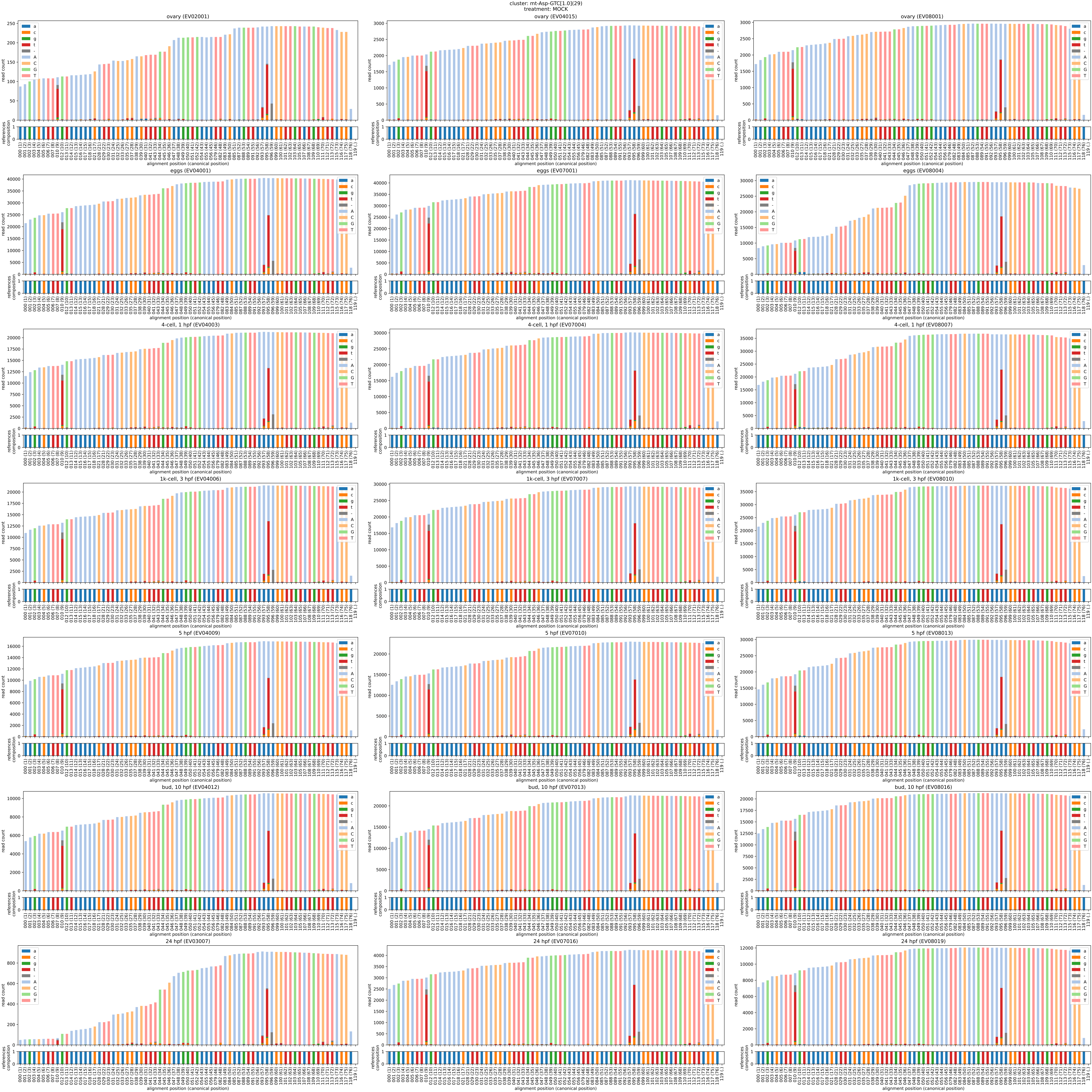
cluster: mt-Asp-GTC[1.0](29)
treatment: MOCK
ovary (EV02001)
ovary (EV04015)
ovary (EV08001)
eggs (EV04001)
eggs (EV07001)
eggs (EV08004)
4-cell, 1 hpf (EV04003)
4-cell, 1 hpf (EV07004)
4-cell, 1 hpf (EV08007)
1k-cell, 3 hpf (EV04006)
1k-cell, 3 hpf (EV07007)
1k-cell, 3 hpf (EV08010)
5 hpf (EV04009)
5 hpf (EV07010)
5 hpf (EV08013)
bud, 10 hpf (EV04012)
bud, 10 hpf (EV07013)
bud, 10 hpf (EV08016)
24 hpf (EV03007)
24 hpf (EV07016)
24 hpf (EV08019)
read count
references composition
alignment position (canonical position)
a
c
g
t
-
A
C
G
T

cluster: mt-Cys-GCA[1.0](1)
treatment: MOCK

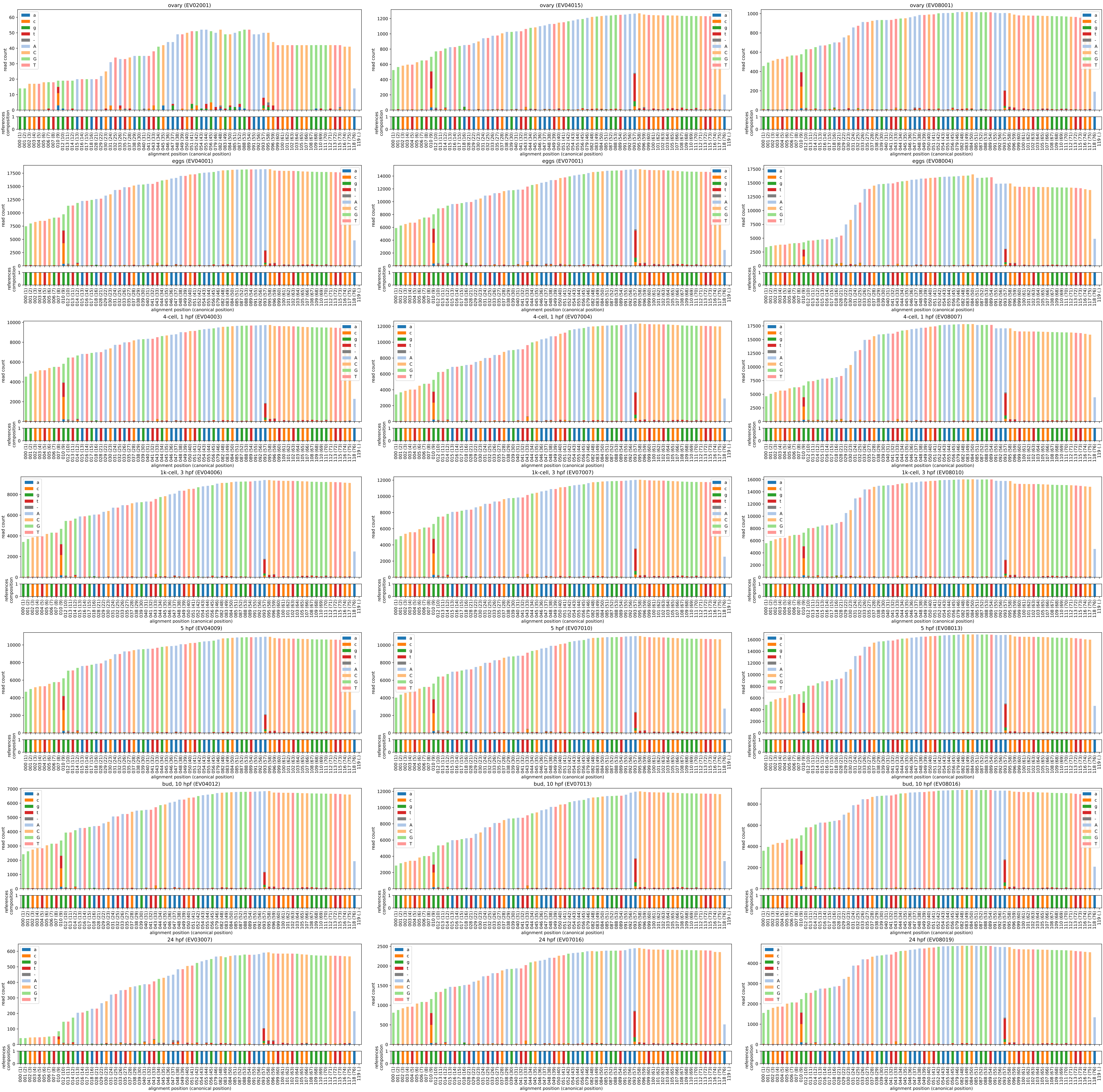

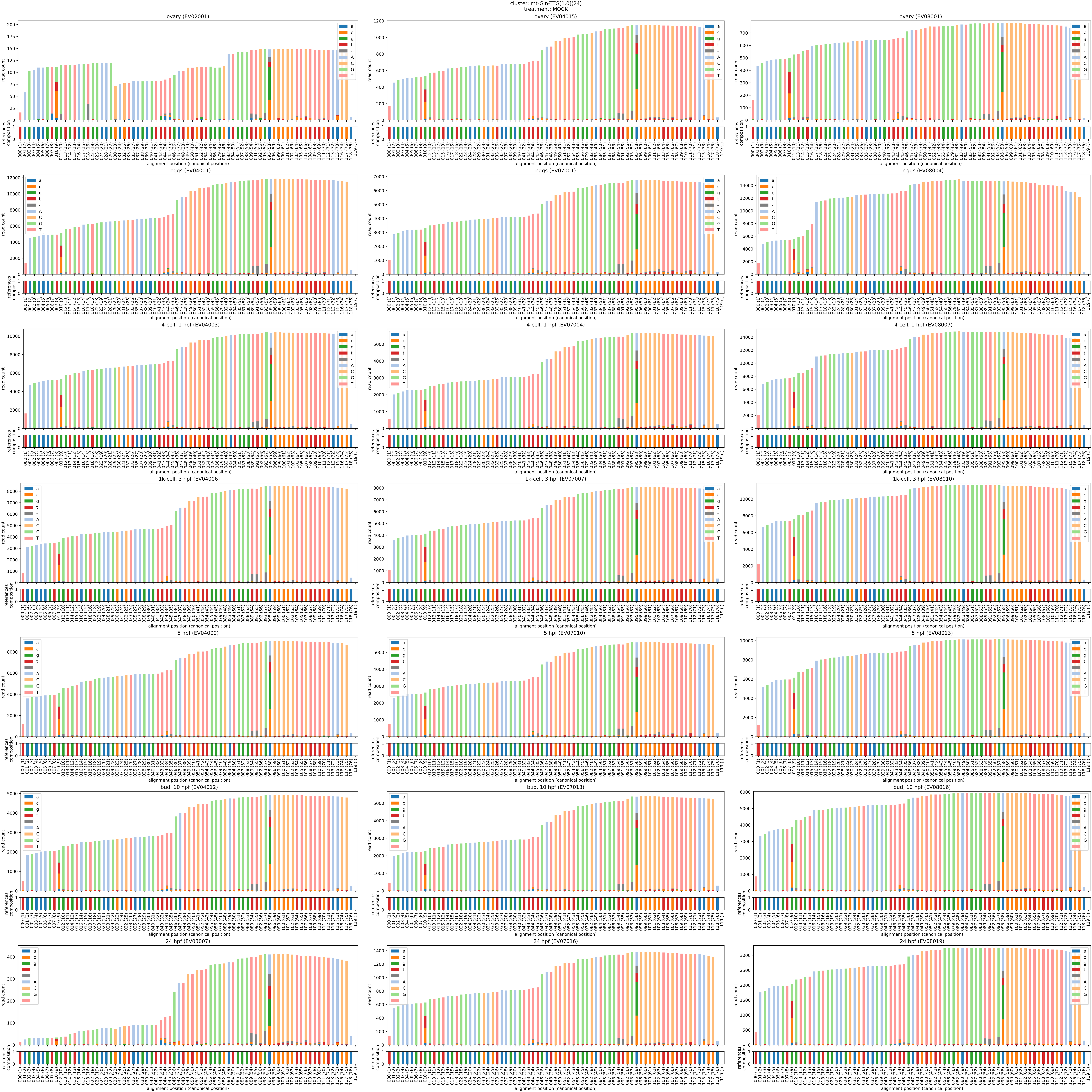
cluster: mt-Gln-TTG[1.0](24)
treatment: MOCK
ovary (EV02001)
ovary (EV04015)
ovary (EV08001)
eggs (EV04001)
eggs (EV07001)
eggs (EV08004)
4-cell, 1 hpf (EV04003)
4-cell, 1 hpf (EV07004)
4-cell, 1 hpf (EV08007)
1k-cell, 3 hpf (EV04006)
1k-cell, 3 hpf (EV07007)
1k-cell, 3 hpf (EV08010)
5 hpf (EV04009)
5 hpf (EV07010)
5 hpf (EV08013)
bud, 10 hpf (EV04012)
bud, 10 hpf (EV07013)
bud, 10 hpf (EV08016)
24 hpf (EV03007)
24 hpf (EV07016)
24 hpf (EV08019)
read count
references composition
alignment position (canonical position)
a
c
g
t
-
A
C
G
T

cluster: mt-Glu-TTC[1.0](0)
treatment: MOCK

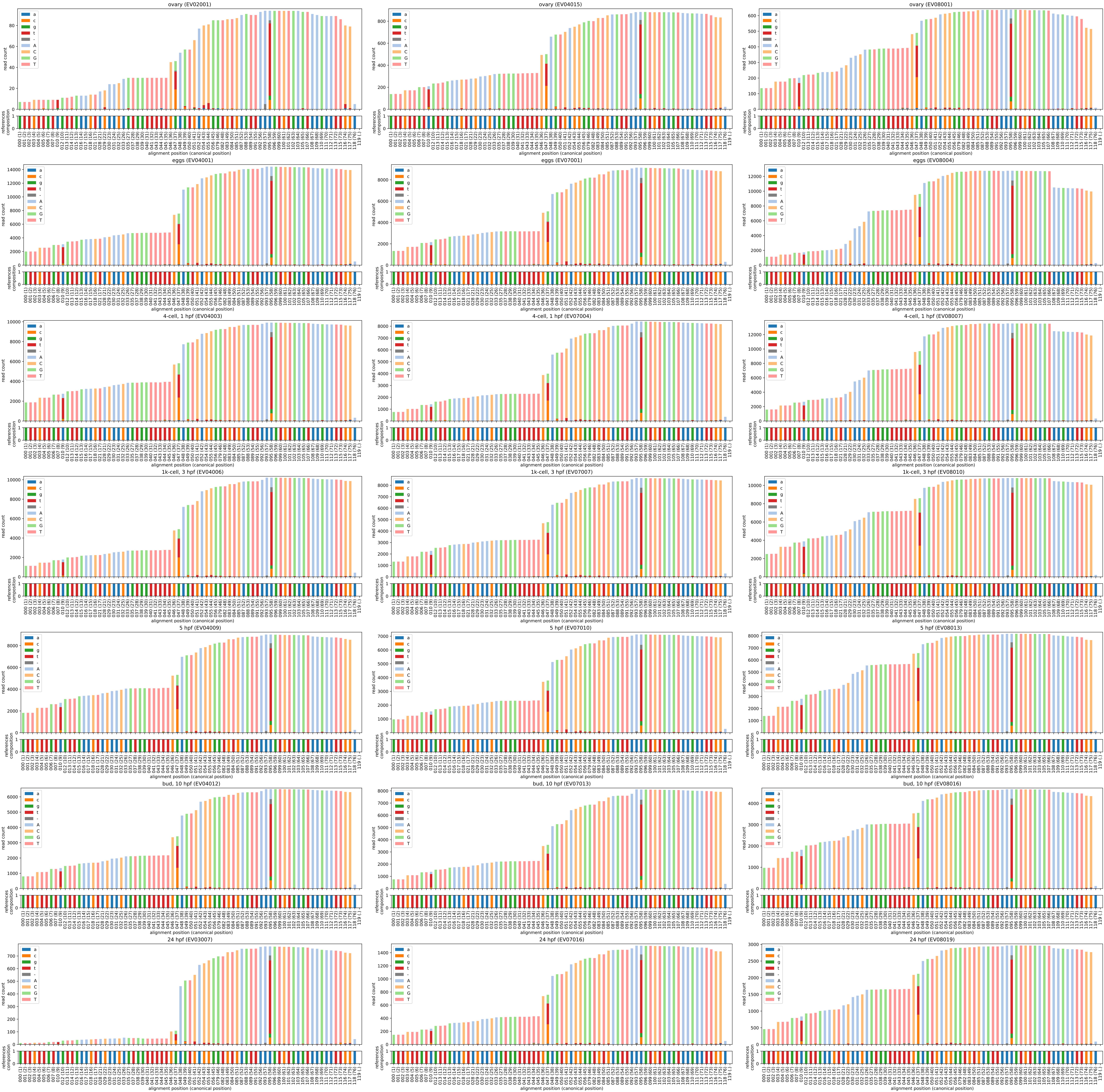

cluster: mt-Gly-TCC[1.0](19)
treatment: MOCK

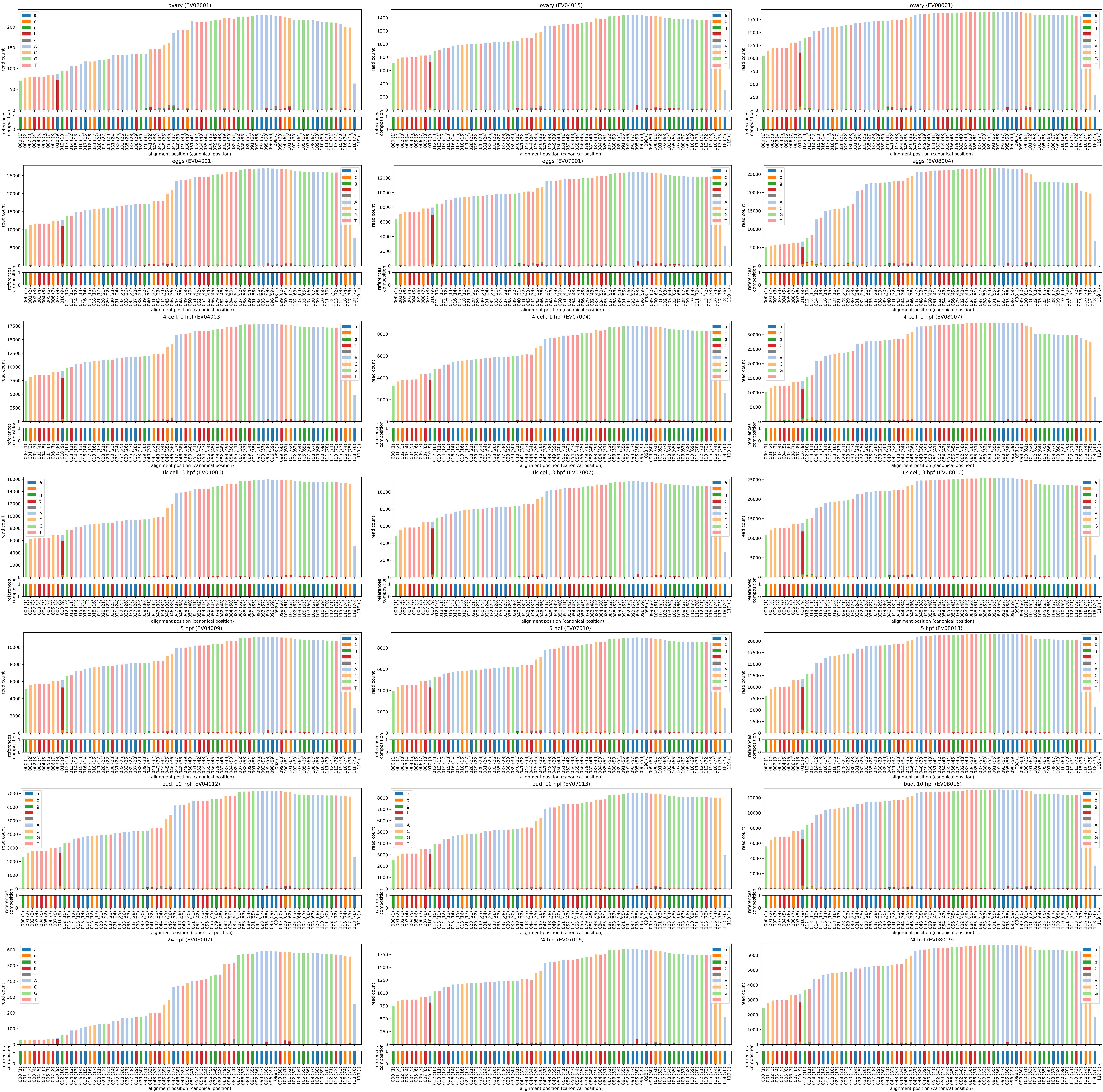

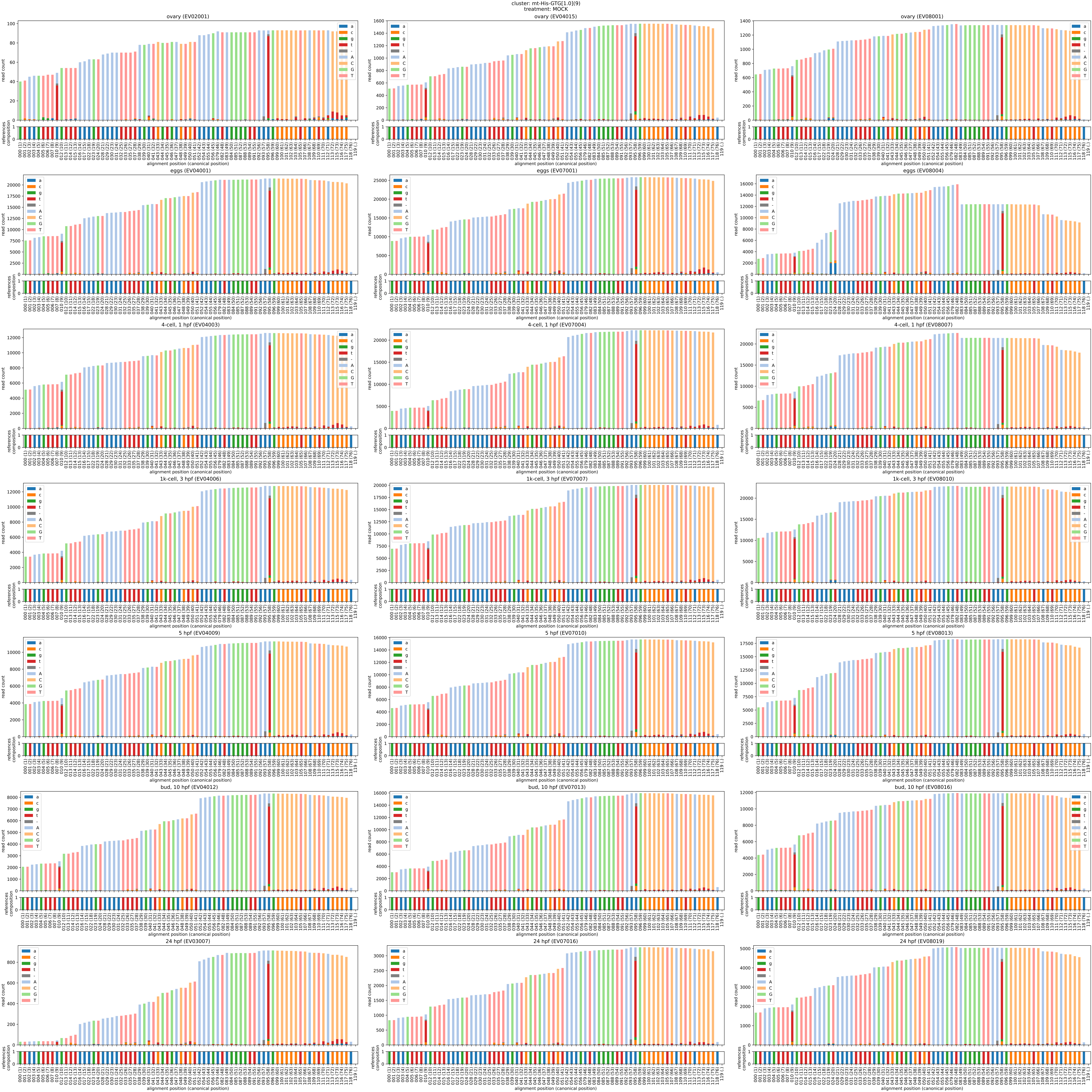
cluster: mt-His-GTG[1.0](9)
treatment: MOCK
ovary (EV02001)
ovary (EV04015)
ovary (EV08001)
eggs (EV04001)
eggs (EV07001)
eggs (EV08004)
4-cell, 1 hpf (EV04003)
4-cell, 1 hpf (EV07004)
4-cell, 1 hpf (EV08007)
1k-cell, 3 hpf (EV04006)
1k-cell, 3 hpf (EV07007)
1k-cell, 3 hpf (EV08010)
5 hpf (EV04009)
5 hpf (EV07010)
5 hpf (EV08013)
bud, 10 hpf (EV04012)
bud, 10 hpf (EV07013)
bud, 10 hpf (EV08016)
24 hpf (EV03007)
24 hpf (EV07016)
24 hpf (EV08019)
read count
references composition
alignment position (canonical position)
a
c
g
t
-
A
C
G
T

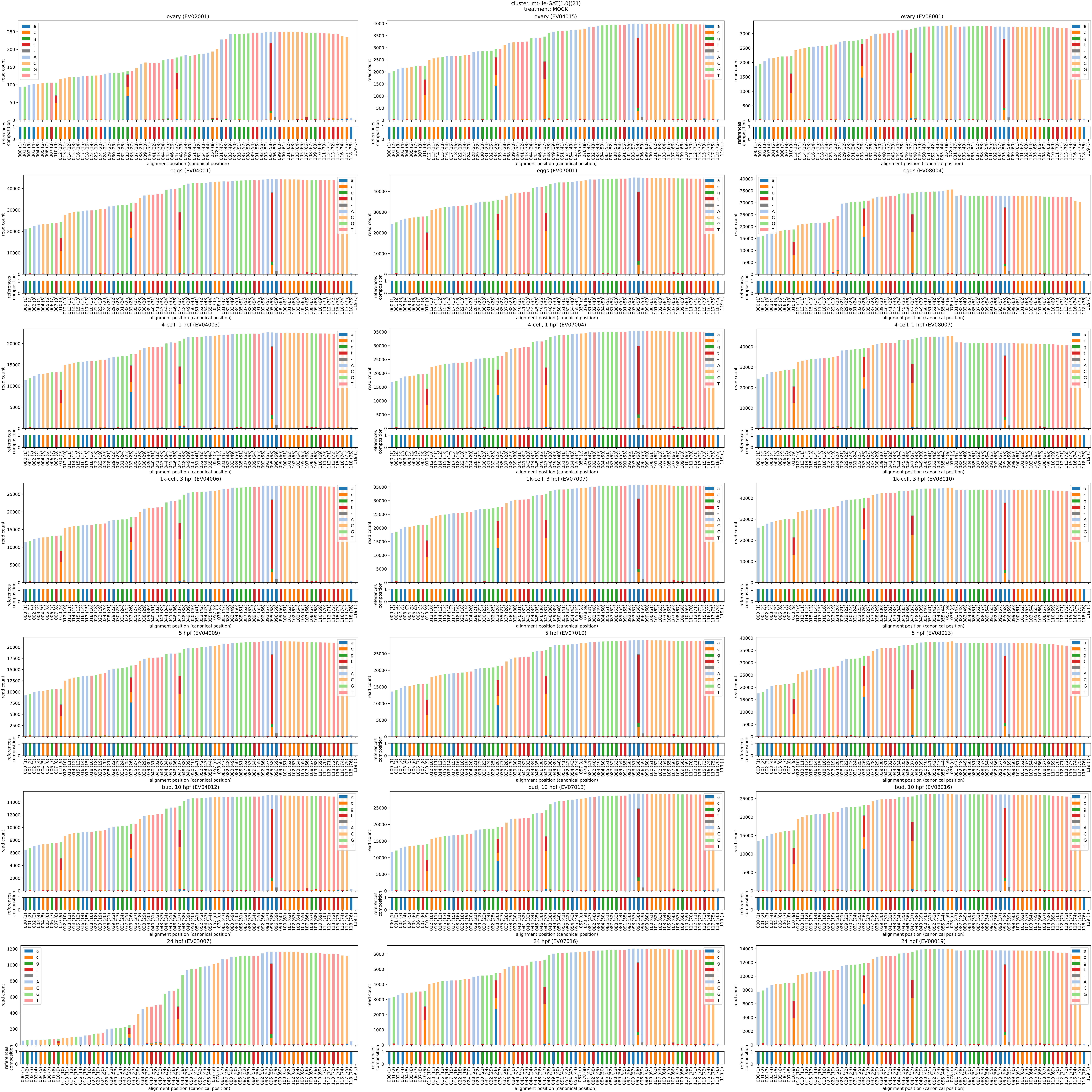
cluster: mt-Ile-GAT[1.0](21)
treatment: MOCK
ovary (EV02001)
ovary (EV04015)
ovary (EV08001)
eggs (EV04001)
eggs (EV07001)
eggs (EV08004)
4-cell, 1 hpf (EV04003)
4-cell, 1 hpf (EV07004)
4-cell, 1 hpf (EV08007)
1k-cell, 3 hpf (EV04006)
1k-cell, 3 hpf (EV07007)
1k-cell, 3 hpf (EV08010)
5 hpf (EV04009)
5 hpf (EV07010)
5 hpf (EV08013)
bud, 10 hpf (EV04012)
bud, 10 hpf (EV07013)
bud, 10 hpf (EV08016)
24 hpf (EV03007)
24 hpf (EV07016)
24 hpf (EV08019)
read count
references composition
alignment position (canonical position)
a
c
g
t
-
A
C
G
T

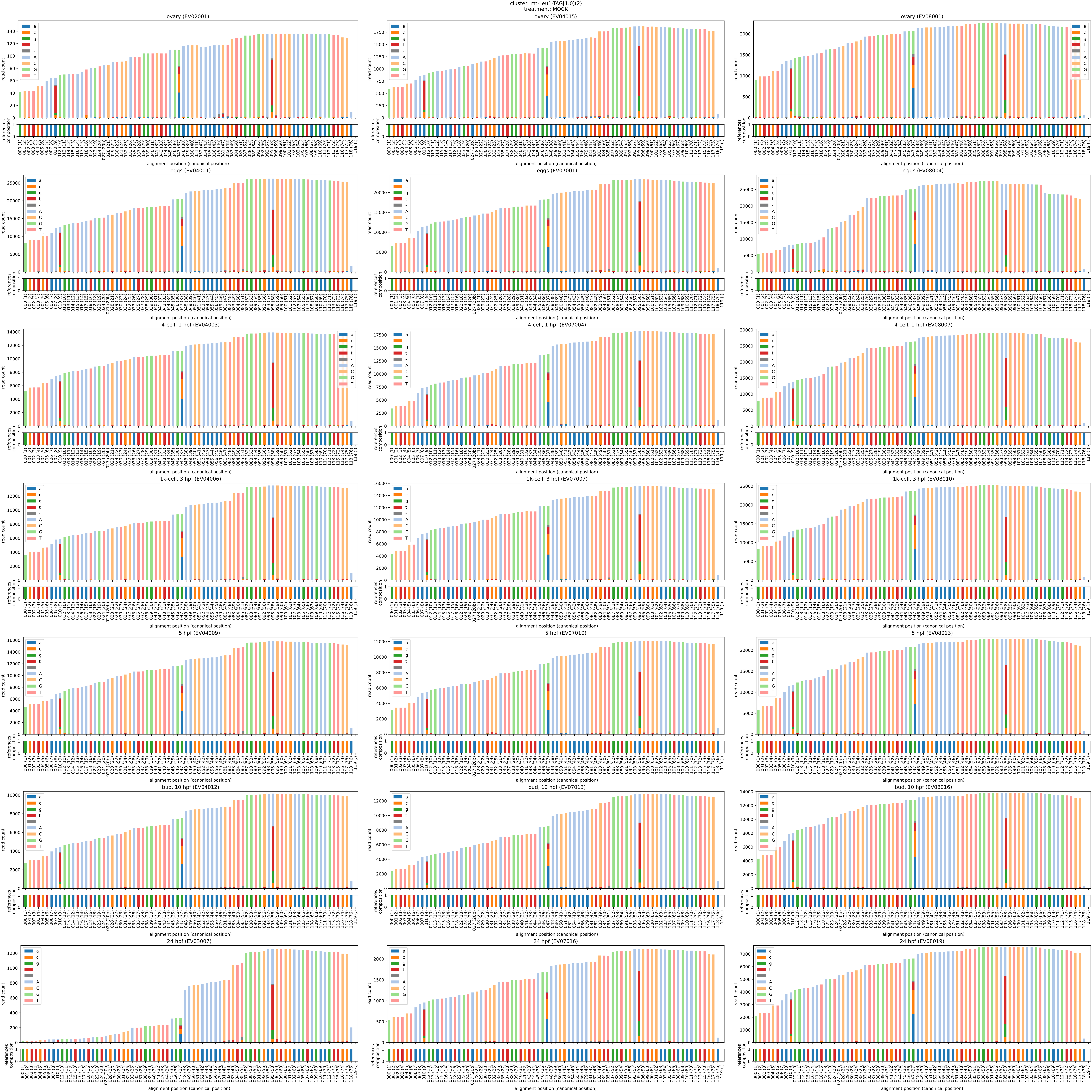
cluster: mt-Leu1-TAG[1.0](2)
treatment: MOCK
ovary (EV02001)
ovary (EV04015)
ovary (EV08001)
eggs (EV04001)
eggs (EV07001)
eggs (EV08004)
4-cell, 1 hpf (EV04003)
4-cell, 1 hpf (EV07004)
4-cell, 1 hpf (EV08007)
1k-cell, 3 hpf (EV04006)
1k-cell, 3 hpf (EV07007)
1k-cell, 3 hpf (EV08010)
5 hpf (EV04009)
5 hpf (EV07010)
5 hpf (EV08013)
bud, 10 hpf (EV04012)
bud, 10 hpf (EV07013)
bud, 10 hpf (EV08016)
24 hpf (EV03007)
24 hpf (EV07016)
24 hpf (EV08019)
a
c
g
t
-
A
C
G
T
read count
references composition
alignment position (canonical position)

cluster: mt-Leu2-TAA[1.0](15)
treatment: MOCK

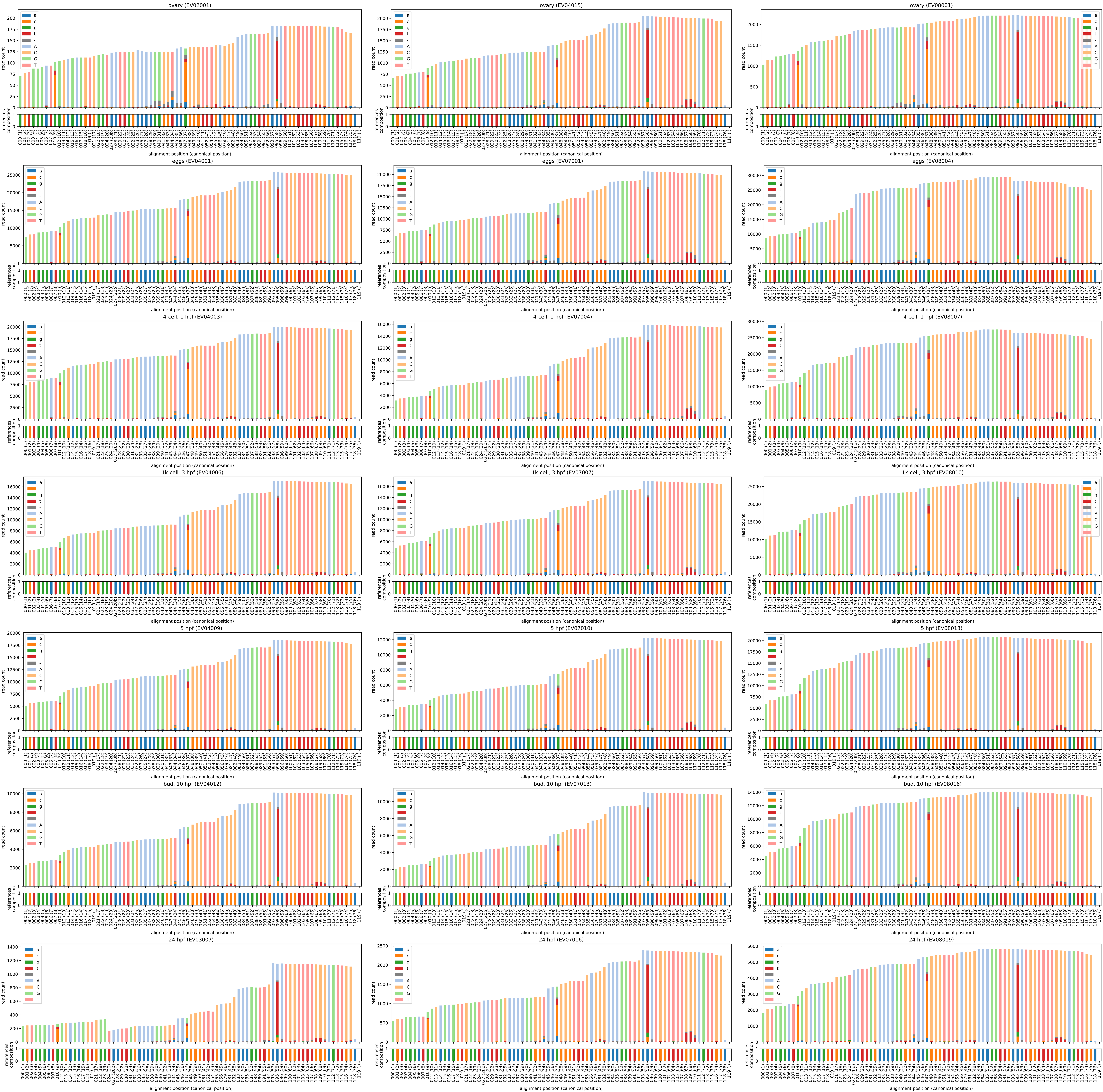

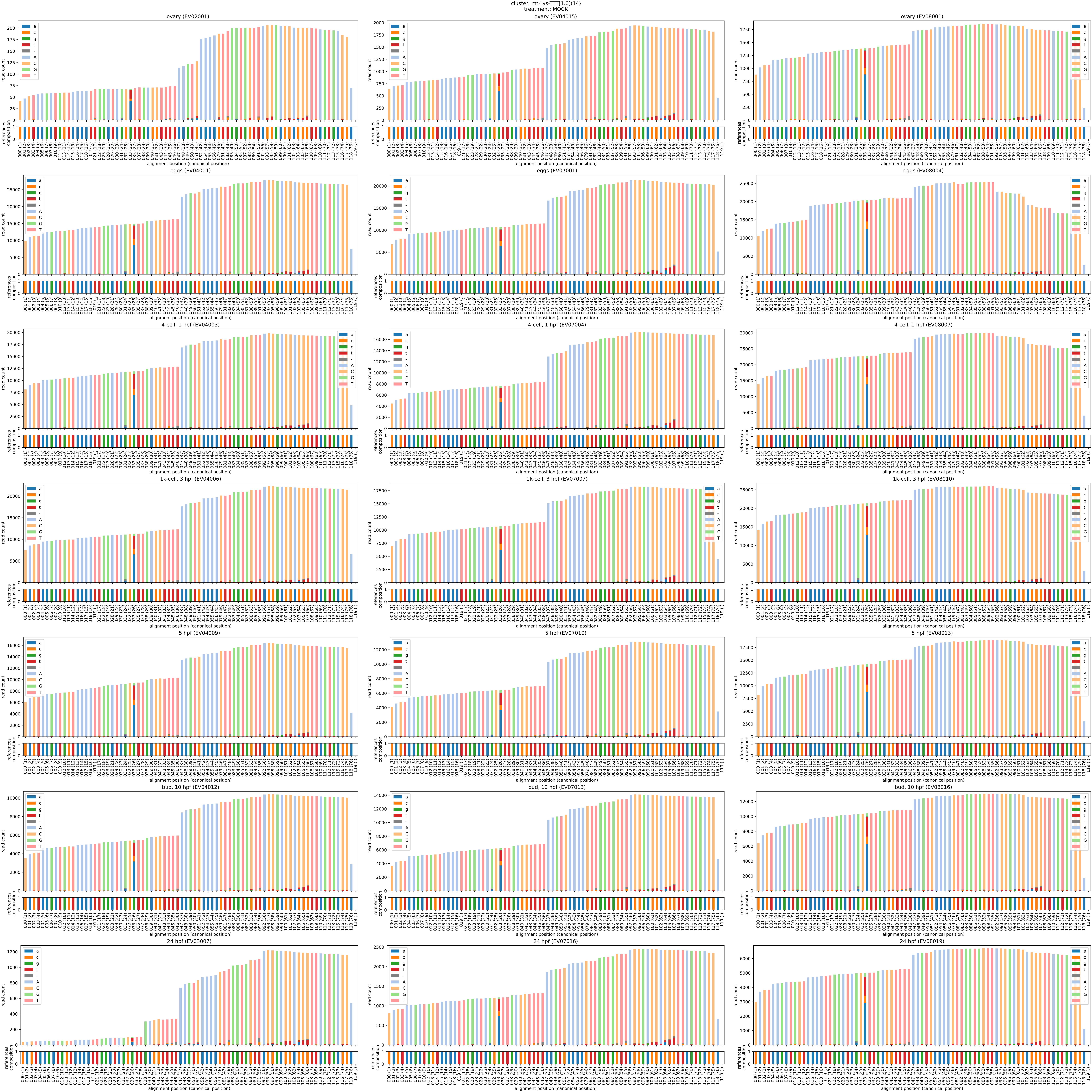
cluster: mt-Lys-TTT[1.0](14)
treatment: MOCK
ovary (EV02001)
ovary (EV04015)
ovary (EV08001)
eggs (EV04001)
eggs (EV07001)
eggs (EV08004)
4-cell, 1 hpf (EV04003)
4-cell, 1 hpf (EV07004)
4-cell, 1 hpf (EV08007)
1k-cell, 3 hpf (EV04006)
1k-cell, 3 hpf (EV07007)
1k-cell, 3 hpf (EV08010)
5 hpf (EV04009)
5 hpf (EV07010)
5 hpf (EV08013)
bud, 10 hpf (EV04012)
bud, 10 hpf (EV07013)
bud, 10 hpf (EV08016)
24 hpf (EV03007)
24 hpf (EV07016)
24 hpf (EV08019)
read count
references composition
alignment position (canonical position)
a
c
g
t
-
A
C
G
T

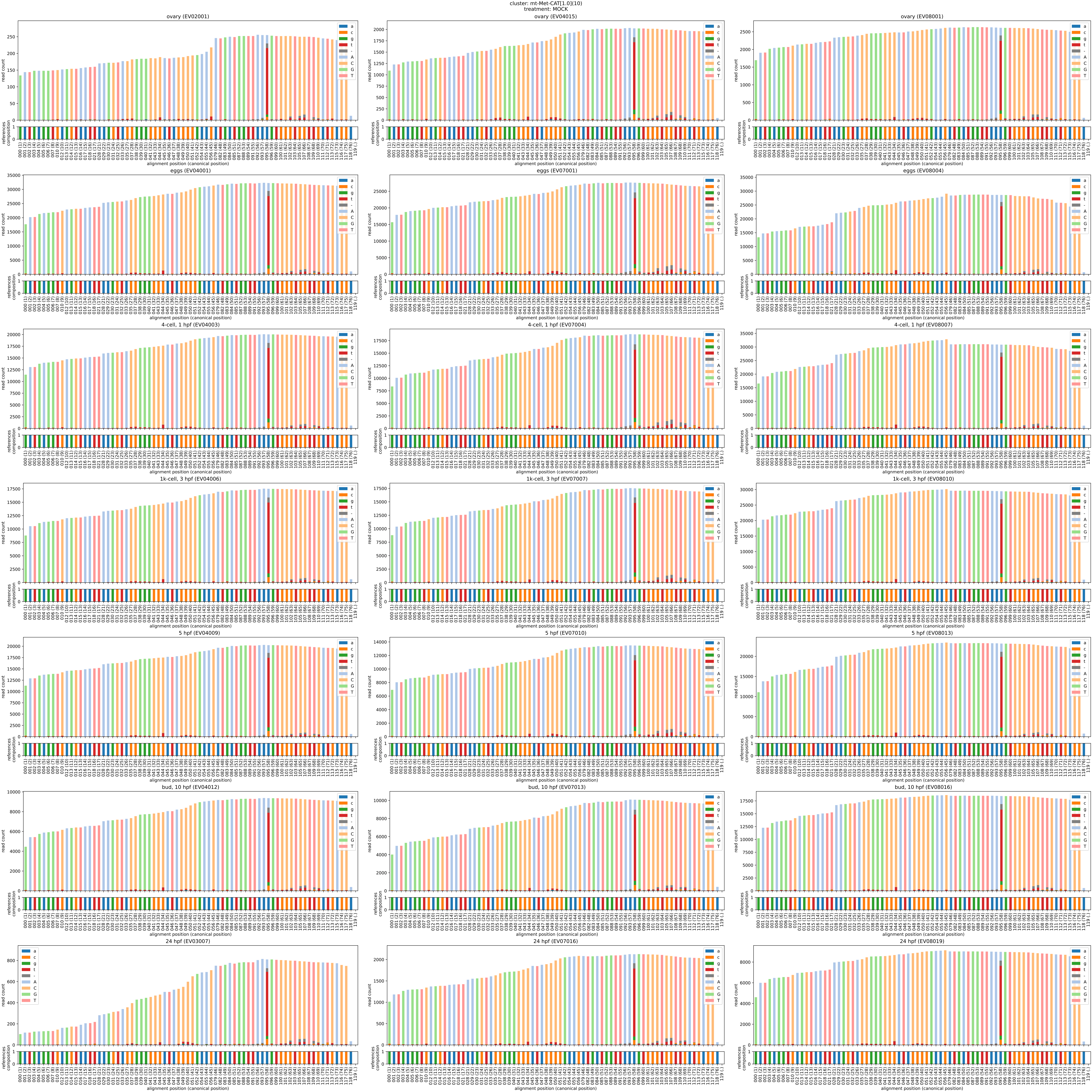
cluster: mt-Met-CAT[1.0](10)
treatment: MOCK
ovary (EV02001)
ovary (EV04015)
ovary (EV08001)
eggs (EV04001)
eggs (EV07001)
eggs (EV08004)
4-cell, 1 hpf (EV04003)
4-cell, 1 hpf (EV07004)
4-cell, 1 hpf (EV08007)
1k-cell, 3 hpf (EV04006)
1k-cell, 3 hpf (EV07007)
1k-cell, 3 hpf (EV08010)
5 hpf (EV04009)
5 hpf (EV07010)
5 hpf (EV08013)
bud, 10 hpf (EV04012)
bud, 10 hpf (EV07013)
bud, 10 hpf (EV08016)
24 hpf (EV03007)
24 hpf (EV07016)
24 hpf (EV08019)
read count
references composition
alignment position (canonical position)
a
c
g
t
-
A
C
G
T

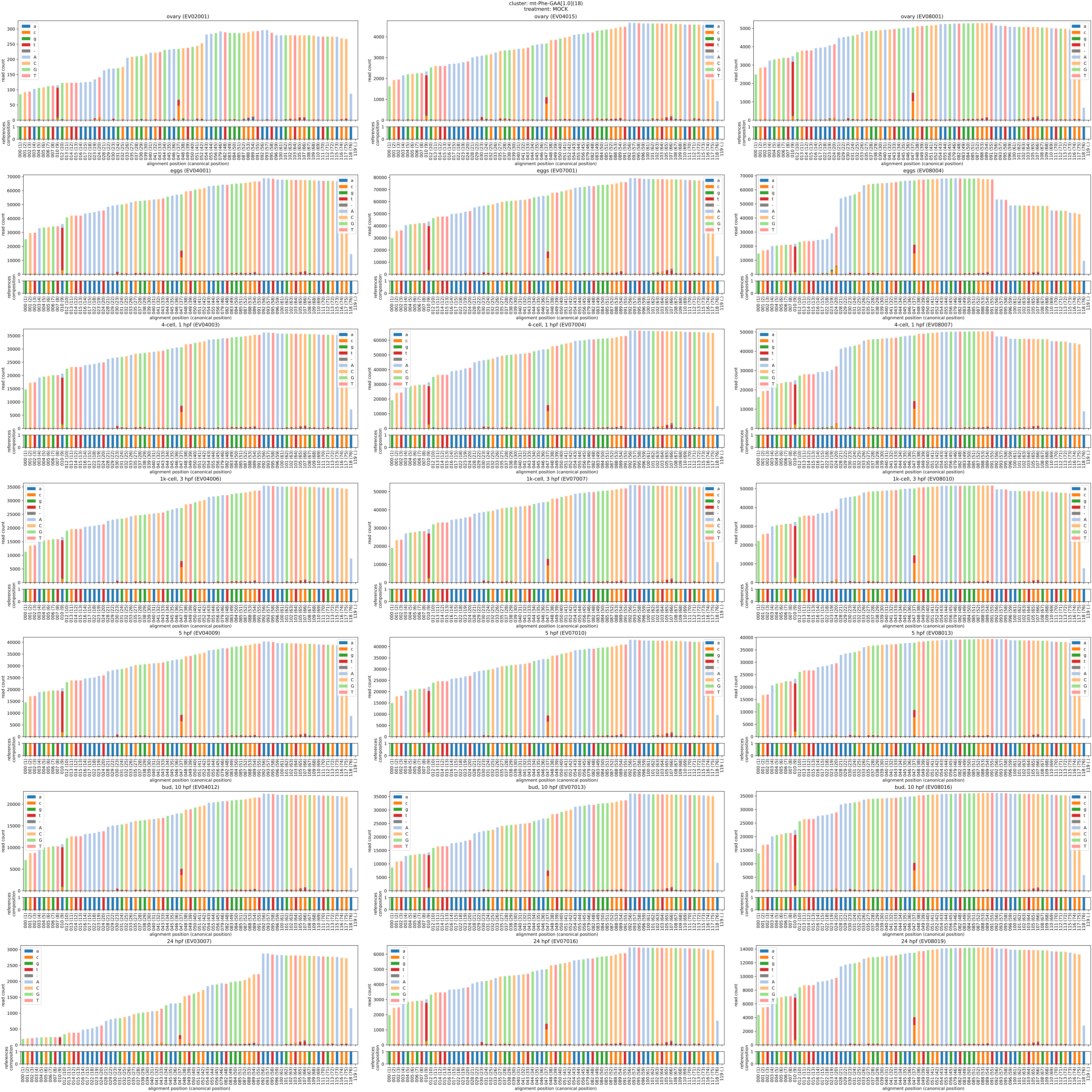

cluster: mt-Phe-GAA[1.0](18)
treatment: MOCK
ovary (EV02001)
ovary (EV04015)
ovary (EV08001)
eggs (EV04001)
eggs (EV07001)
eggs (EV08004)
4-cell, 1 hpf (EV04003)
4-cell, 1 hpf (EV07004)
4-cell, 1 hpf (EV08007)
1k-cell, 3 hpf (EV04006)
1k-cell, 3 hpf (EV07007)
1k-cell, 3 hpf (EV08010)
5 hpf (EV04009)
5 hpf (EV07010)
5 hpf (EV08013)
bud, 10 hpf (EV04012)
bud, 10 hpf (EV07013)
bud, 10 hpf (EV08016)
24 hpf (EV03007)
24 hpf (EV07016)
24 hpf (EV08019)
read count
references composition
alignment position (canonical position)
a
c
g
t
-
A
C
G
T

cluster: mt-Pro-TGG[1.0](20)
treatment: MOCK

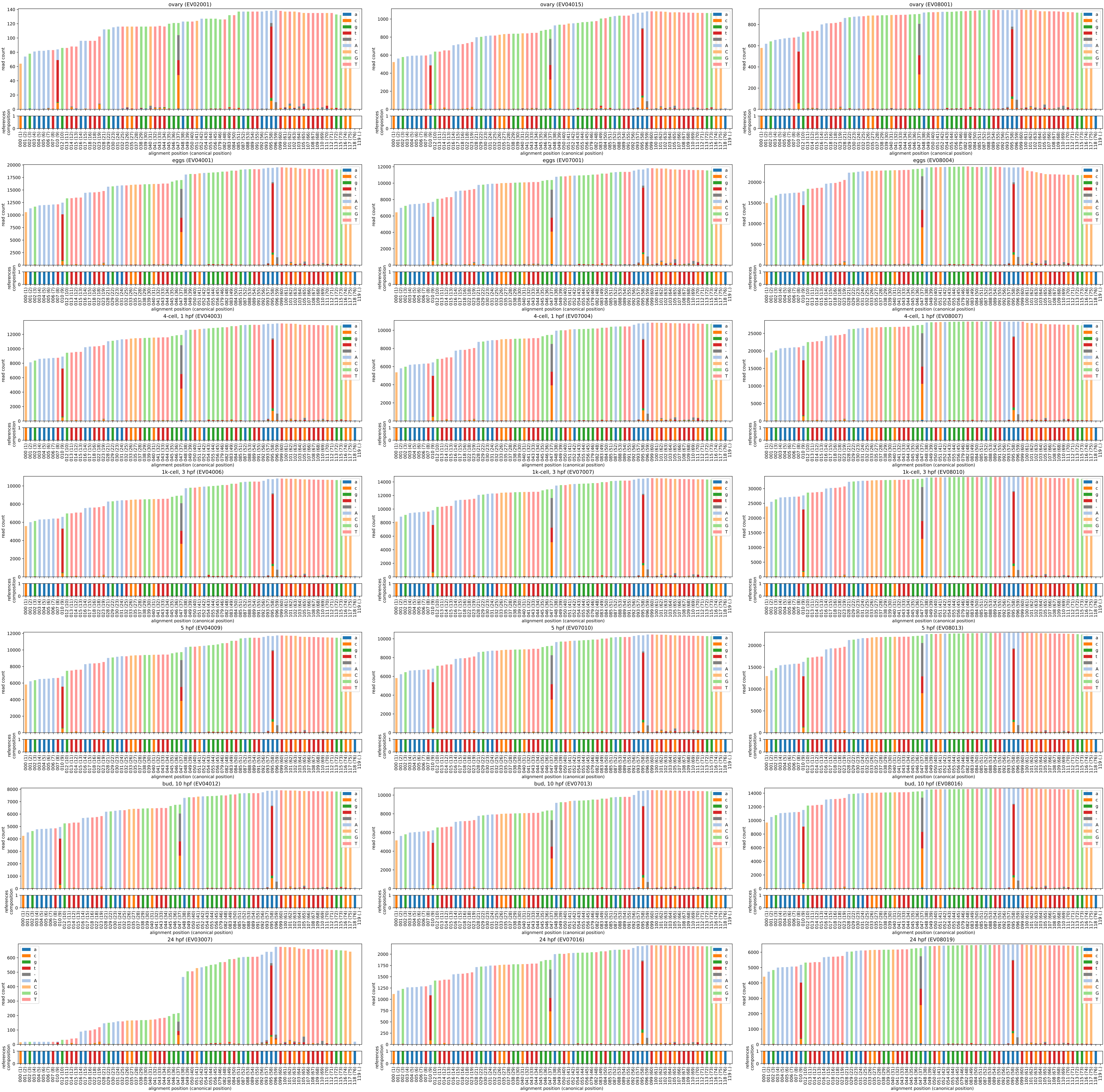

cluster: mt-Ser1-GCT[1,0](4)
treatment: MOCK

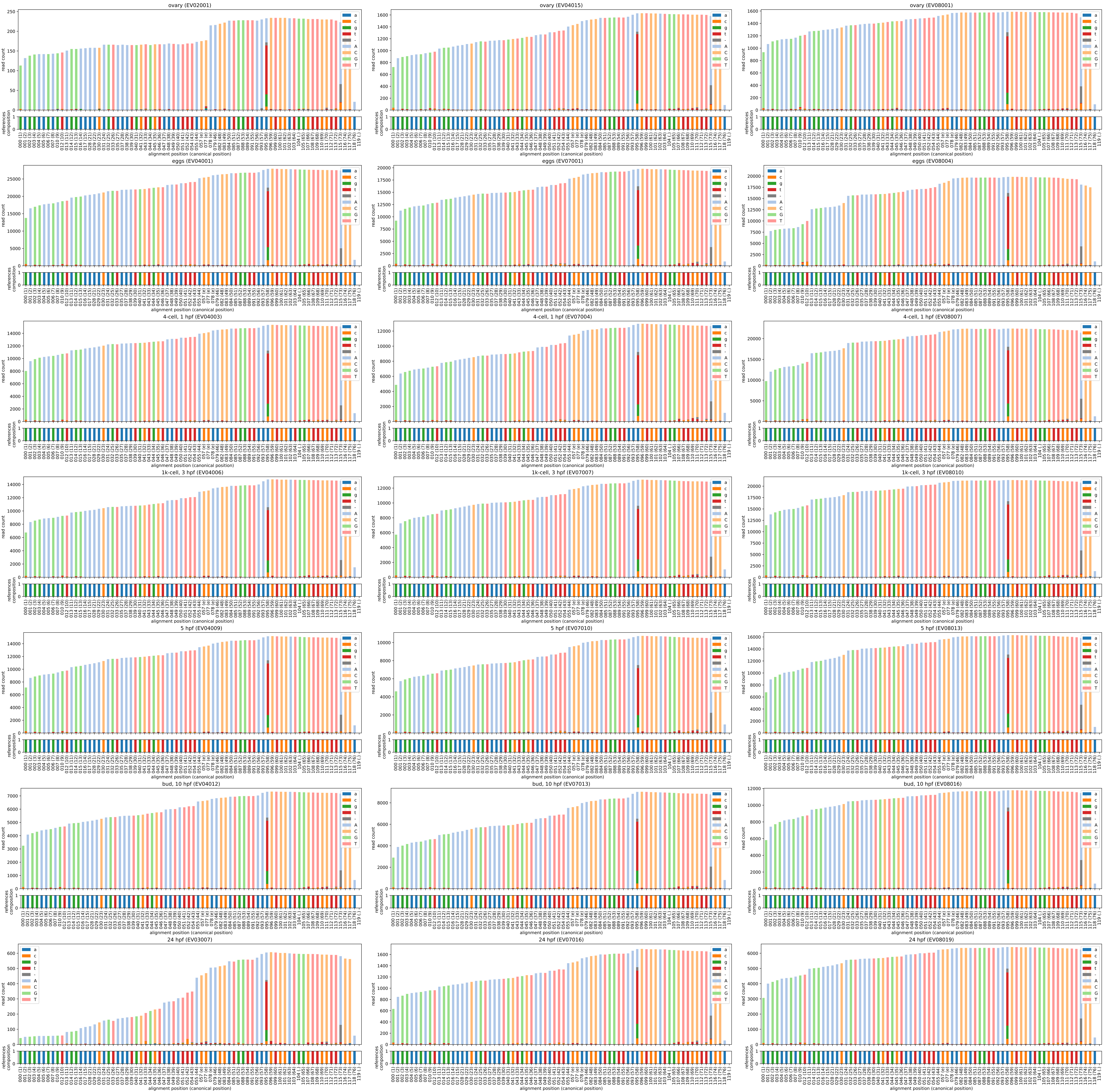

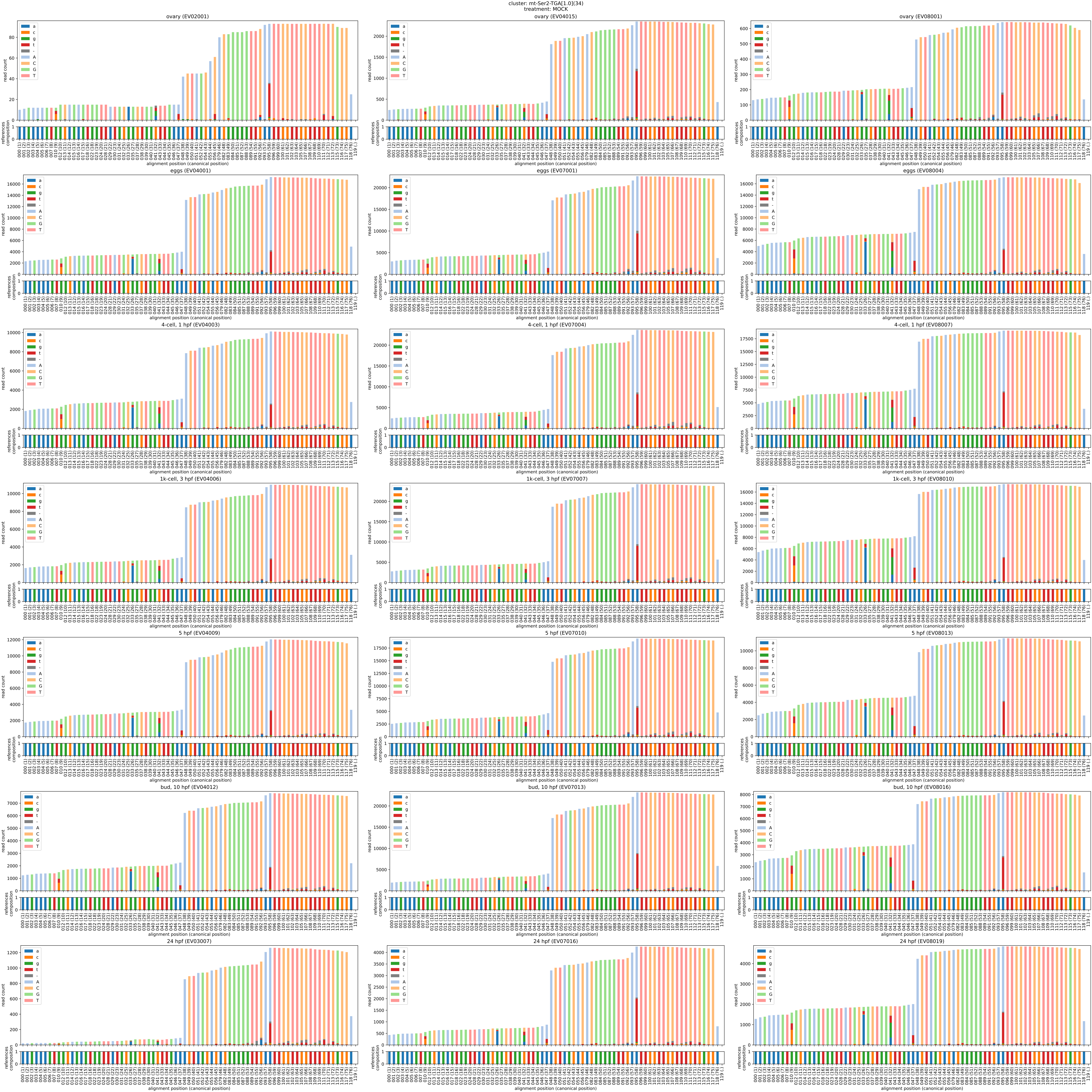

cluster: mt-Ser2-TGA[1.0](34)
treatment: MOCK
ovary (EV02001)
ovary (EV04015)
ovary (EV08001)
eggs (EV04001)
eggs (EV07001)
eggs (EV08004)
4-cell, 1 hpf (EV04003)
4-cell, 1 hpf (EV07004)
4-cell, 1 hpf (EV08007)
1k-cell, 3 hpf (EV04006)
1k-cell, 3 hpf (EV07007)
1k-cell, 3 hpf (EV08010)
5 hpf (EV04009)
5 hpf (EV07010)
5 hpf (EV08013)
bud, 10 hpf (EV04012)
bud, 10 hpf (EV07013)
bud, 10 hpf (EV08016)
24 hpf (EV03007)
24 hpf (EV07016)
24 hpf (EV08019)
read count
references composition
alignment position (canonical position)

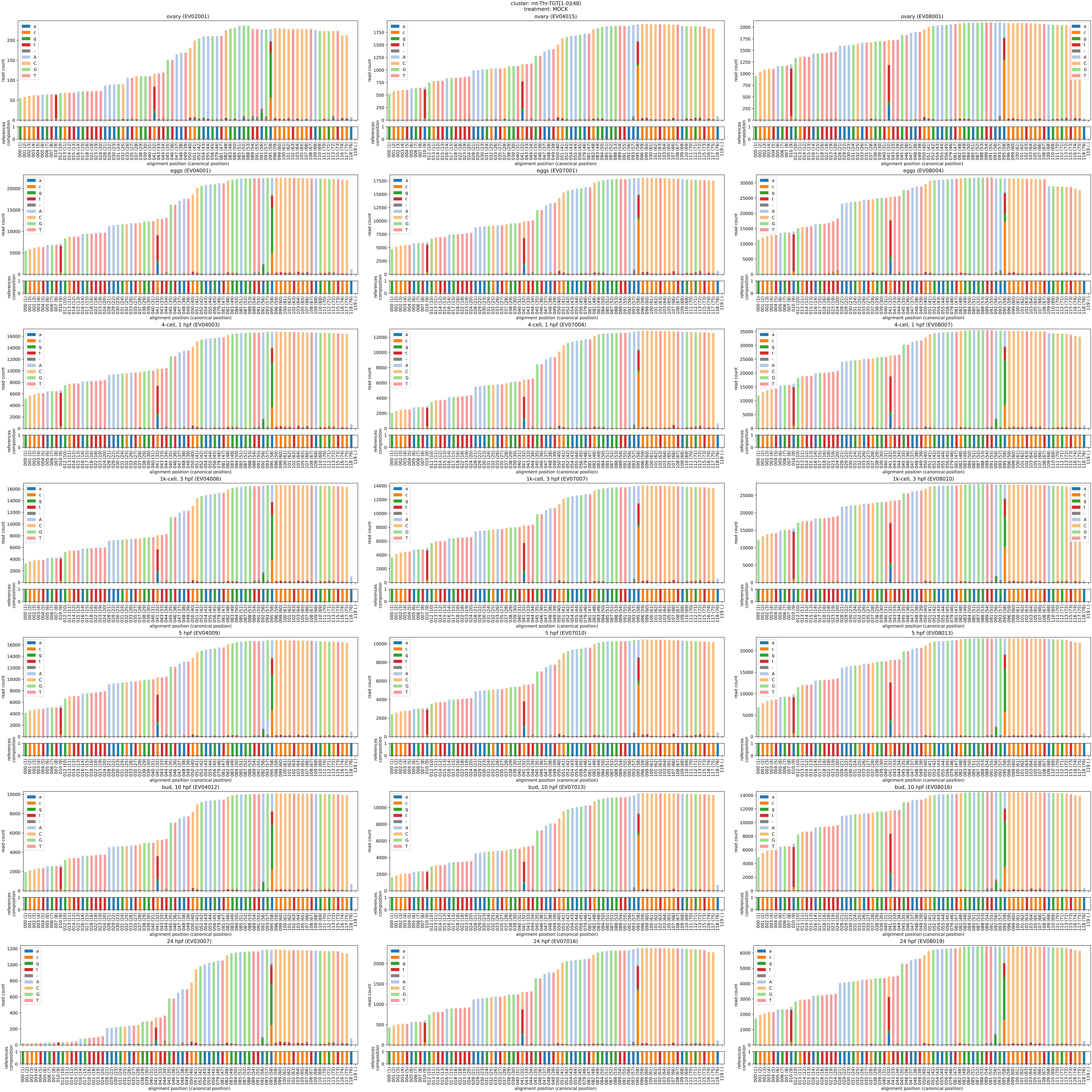
cluster: mt-Thr-TGT[1.0](48)
treatment: MOCK
ovary (EV02001)
ovary (EV04015)
ovary (EV08001)
eggs (EV04001)
eggs (EV07001)
eggs (EV08004)
4-cell, 1 hpf (EV04003)
4-cell, 1 hpf (EV07004)
4-cell, 1 hpf (EV08007)
1k-cell, 3 hpf (EV04006)
1k-cell, 3 hpf (EV07007)
1k-cell, 3 hpf (EV08010)
5 hpf (EV04009)
5 hpf (EV07010)
5 hpf (EV08013)
bud, 10 hpf (EV04012)
bud, 10 hpf (EV07013)
bud, 10 hpf (EV08016)
24 hpf (EV03007)
24 hpf (EV07016)
24 hpf (EV08019)
read count
references composition
alignment position (canonical position)
a
c
g
t
-
A
C
G
T

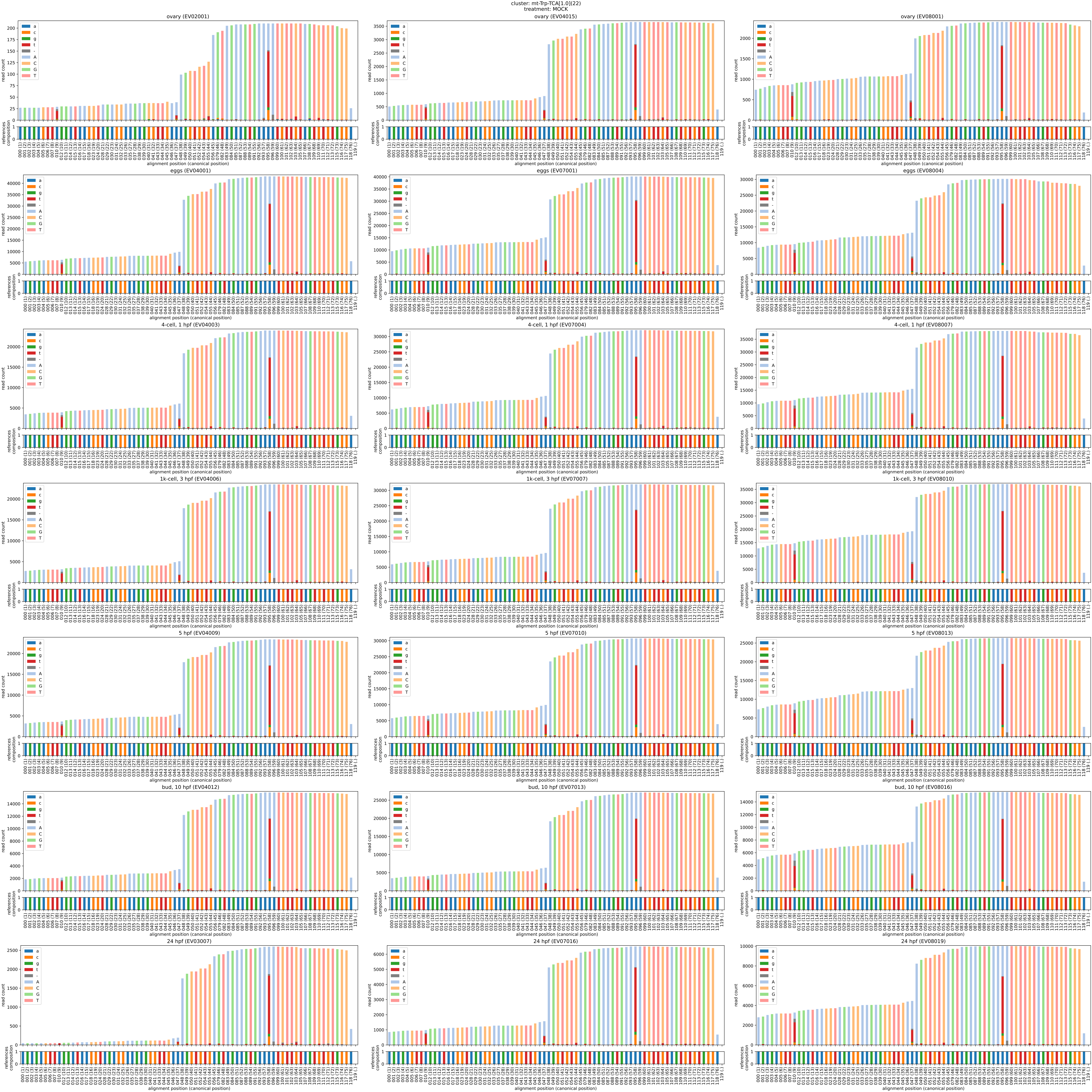
cluster: mt-Trp-TCA[1.0](22)
treatment: MOCK
ovary (EV02001)
ovary (EV04015)
ovary (EV08001)
eggs (EV04001)
eggs (EV07001)
eggs (EV08004)
4-cell, 1 hpf (EV04003)
4-cell, 1 hpf (EV07004)
4-cell, 1 hpf (EV08007)
1k-cell, 3 hpf (EV04006)
1k-cell, 3 hpf (EV07007)
1k-cell, 3 hpf (EV08010)
5 hpf (EV04009)
5 hpf (EV07010)
5 hpf (EV08013)
bud, 10 hpf (EV04012)
bud, 10 hpf (EV07013)
bud, 10 hpf (EV08016)
24 hpf (EV03007)
24 hpf (EV07016)
24 hpf (EV08019)
read count
references composition
alignment position (canonical position)
a
c
g
t
-
A
C
G
T

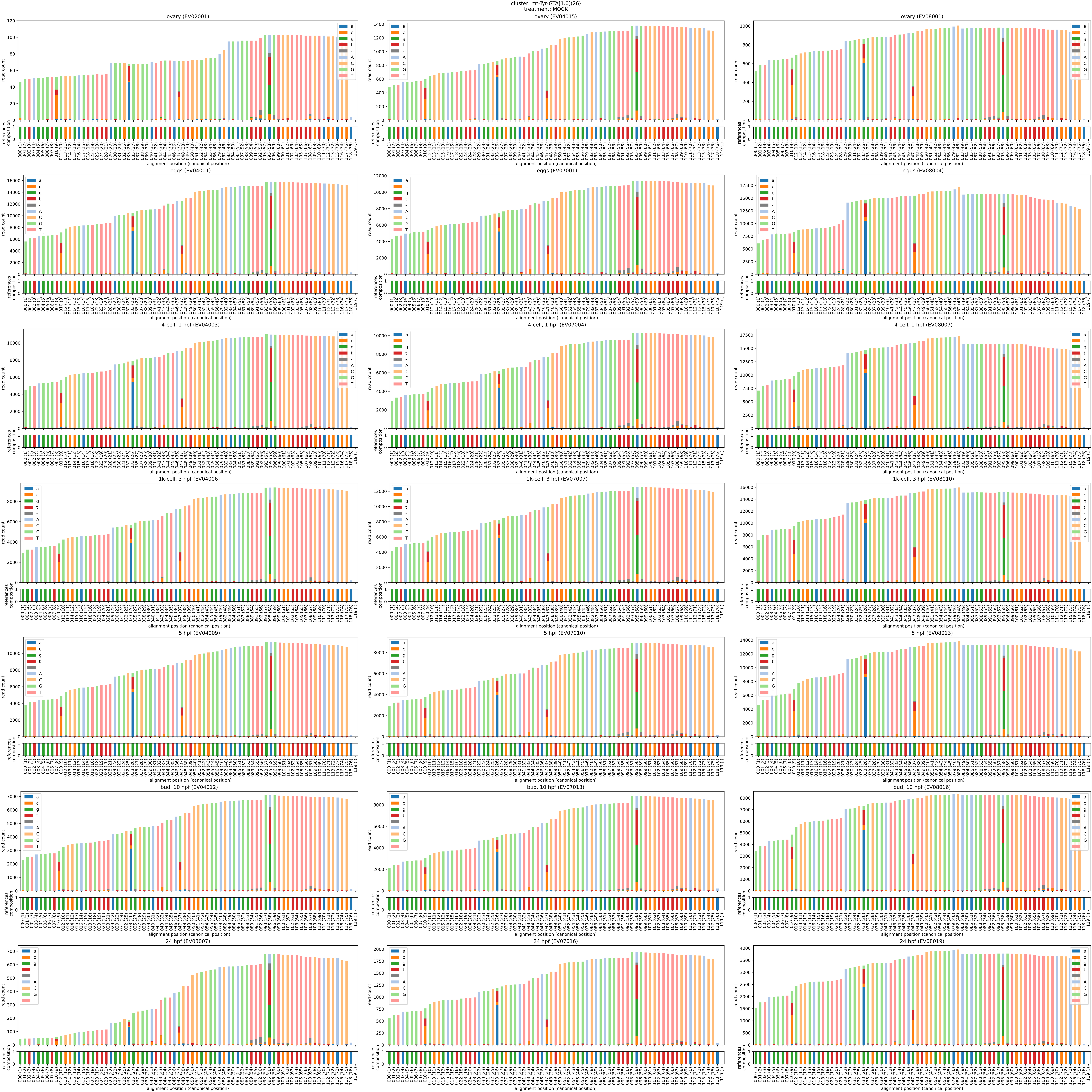

cluster: mt-Tyr-GTA[1.0](26)
treatment: MOCK
ovary (EV02001)
ovary (EV04015)
ovary (EV08001)
eggs (EV04001)
eggs (EV07001)
eggs (EV08004)
4-cell, 1 hpf (EV04003)
4-cell, 1 hpf (EV07004)
4-cell, 1 hpf (EV08007)
1k-cell, 3 hpf (EV04006)
1k-cell, 3 hpf (EV07007)
1k-cell, 3 hpf (EV08010)
5 hpf (EV04009)
5 hpf (EV07010)
5 hpf (EV08013)
bud, 10 hpf (EV04012)
bud, 10 hpf (EV07013)
bud, 10 hpf (EV08016)
24 hpf (EV03007)
24 hpf (EV07016)
24 hpf (EV08019)
read count
references composition
alignment position (canonical position)
a
c
g
t
-
A
C
G
T

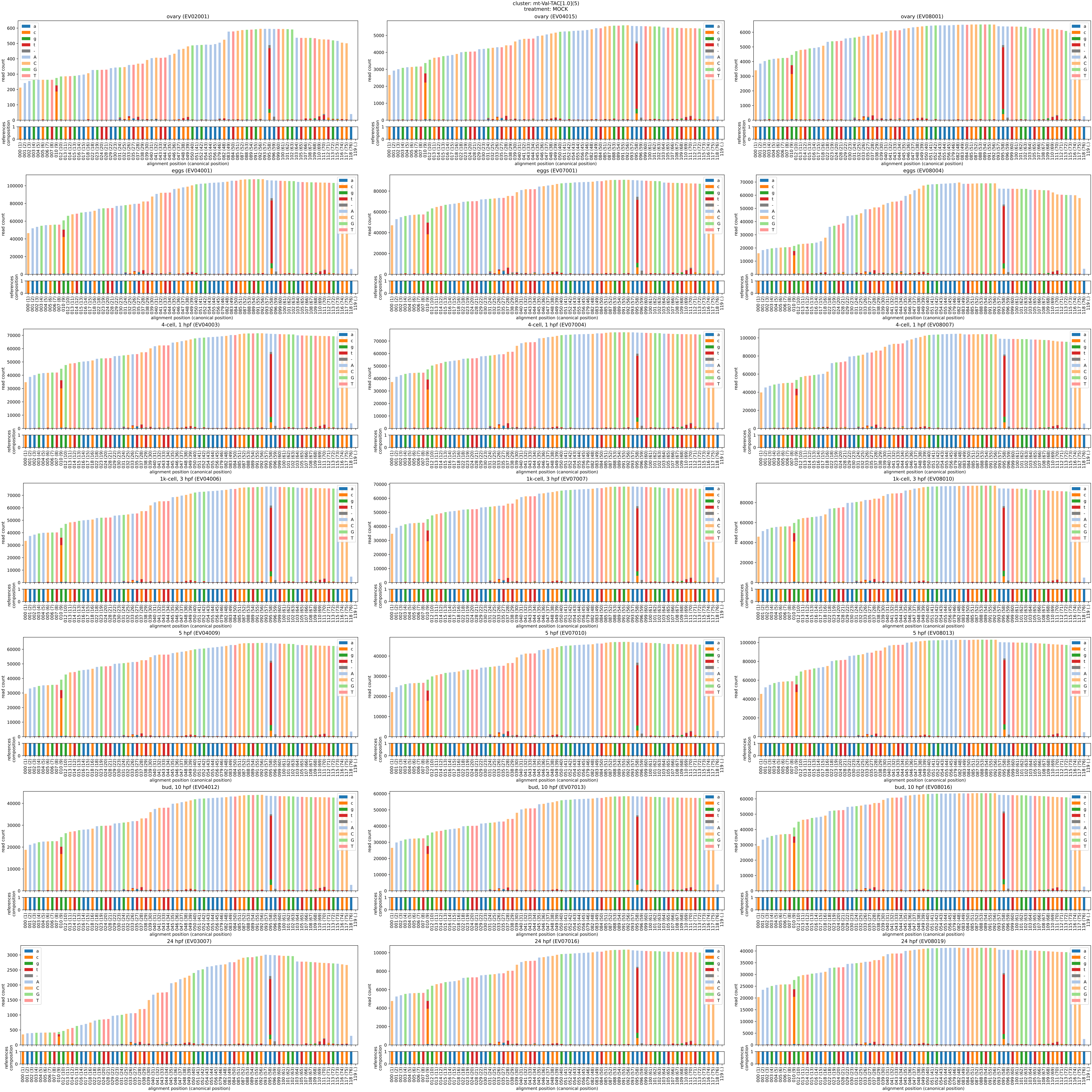
cluster: mt-Val-TAC[1.0](5)
treatment: MOCK
ovary (EV02001)
ovary (EV04015)
ovary (EV08001)
eggs (EV04001)
eggs (EV07001)
eggs (EV08004)
4-cell, 1 hpf (EV04003)
4-cell, 1 hpf (EV07004)
4-cell, 1 hpf (EV08007)
1k-cell, 3 hpf (EV04006)
1k-cell, 3 hpf (EV07007)
1k-cell, 3 hpf (EV08010)
5 hpf (EV04009)
5 hpf (EV07010)
5 hpf (EV08013)
bud, 10 hpf (EV04012)
bud, 10 hpf (EV07013)
bud, 10 hpf (EV08016)
24 hpf (EV03007)
24 hpf (EV07016)
24 hpf (EV08019)
read count
references composition
alignment position (canonical position)
a
c
g
t
-
A
C
G
T
