# Supplementary material for: tRNA expression and modification landscapes, and their dynamics during zebrafish embryo development
Source: Nucleic Acids Res. 2024 Jul 11;52(17):10575–94. doi: 10.1093/nar/gkae595 (PMC11417395; doi:10.1093/nar/gkae595)
Supplement: gkae595_Supplemental_Files [file Supplementary_file_3-coverage_plots_all-DM-v3.pdf › Supplementary_file_3-coverage_plots_all-DM-v3.pdf]

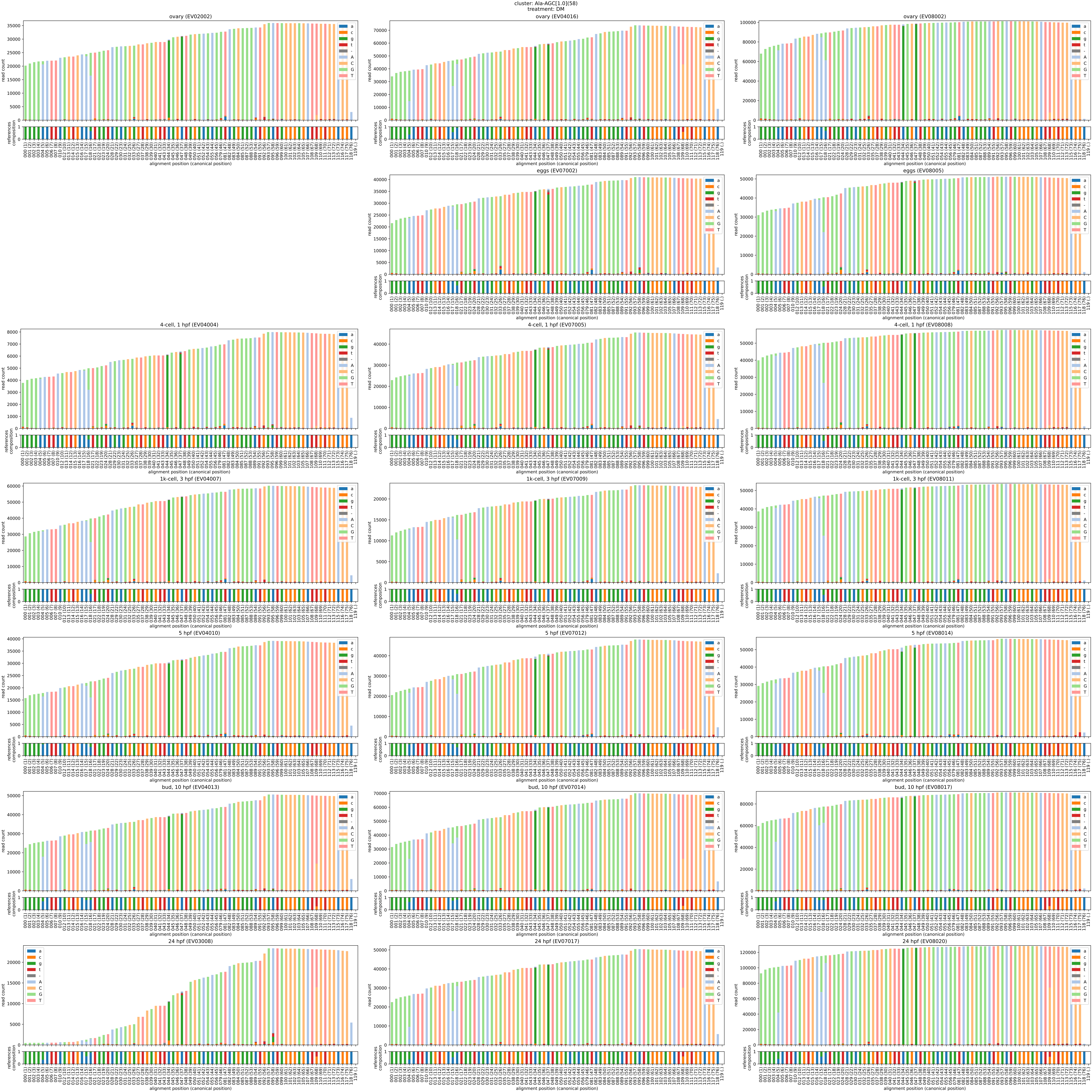
cluster: Ala-AGC[1.0](58)
treatment: DM
ovary (EV02002)
ovary (EV04016)
ovary (EV08002)
eggs (EV07002)
eggs (EV08005)
4-cell, 1 hpf (EV04004)
4-cell, 1 hpf (EV07005)
4-cell, 1 hpf (EV08008)
1k-cell, 3 hpf (EV04007)
1k-cell, 3 hpf (EV07009)
1k-cell, 3 hpf (EV08011)
5 hpf (EV04010)
5 hpf (EV07012)
5 hpf (EV08014)
bud, 10 hpf (EV04013)
bud, 10 hpf (EV07014)
bud, 10 hpf (EV08017)
24 hpf (EV03008)
24 hpf (EV07017)
24 hpf (EV08020)
read count
references composition
alignment position (canonical position)
a
c
g
t
-
A
C
G
T

cluster: Ala-TGC_Ala-CGC[0.78_0.22](59)
treatment: DM

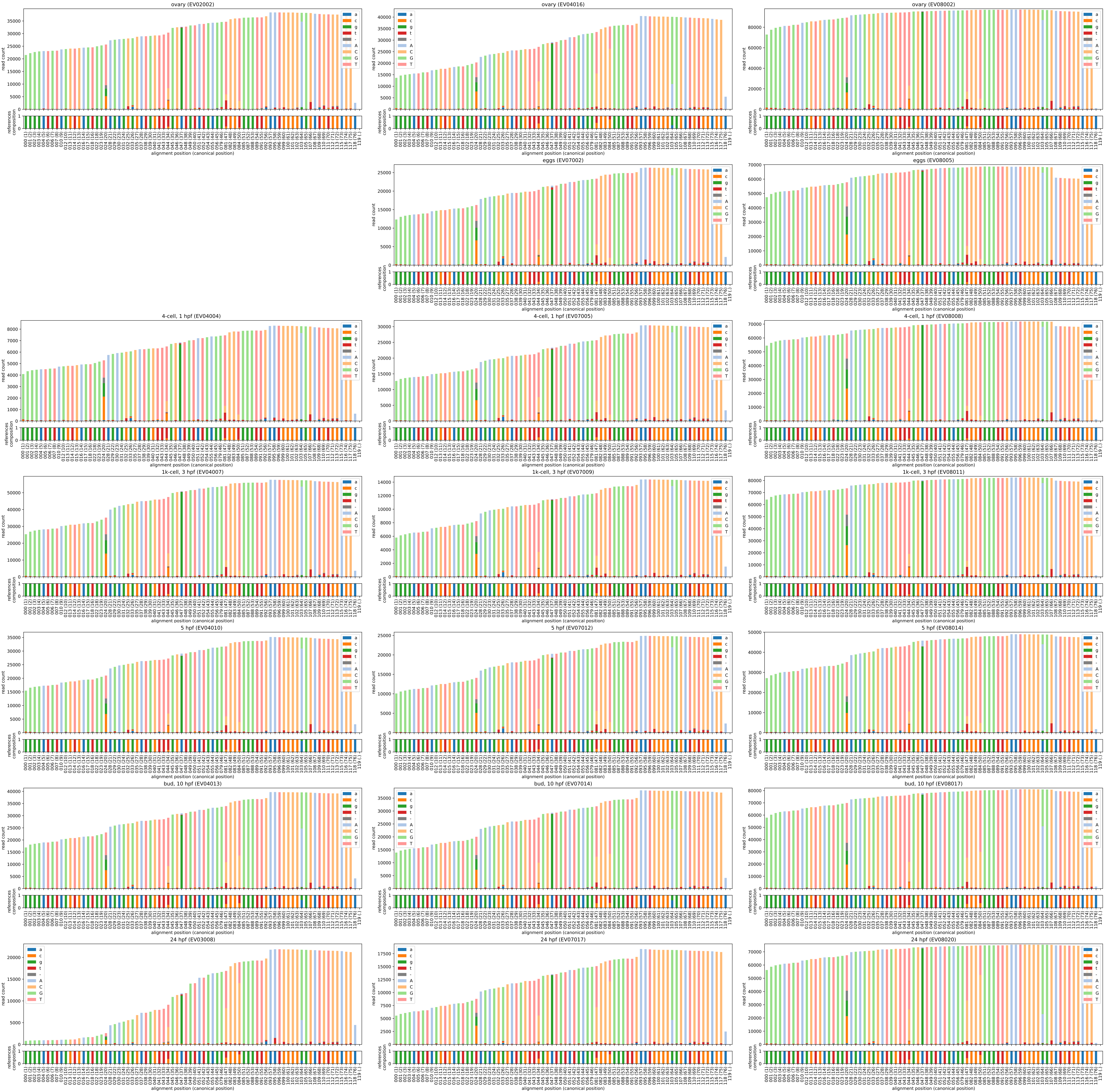

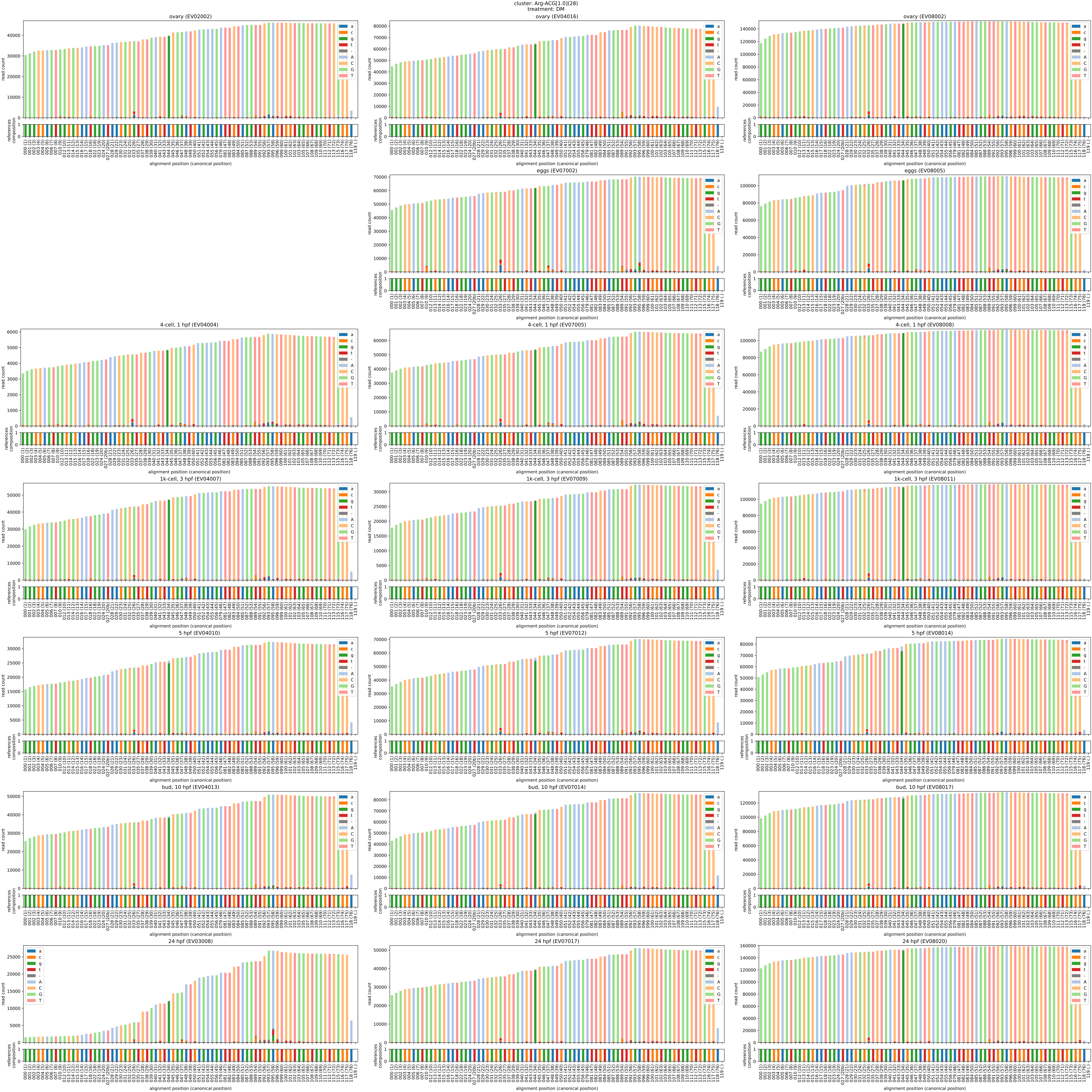
cluster: Arg-ACG[1.0](28)
treatment: DM
ovary (EV02002)
ovary (EV04016)
ovary (EV08002)
eggs (EV07002)
eggs (EV08005)
4-cell, 1 hpf (EV04004)
4-cell, 1 hpf (EV07005)
4-cell, 1 hpf (EV08008)
1k-cell, 3 hpf (EV04007)
1k-cell, 3 hpf (EV07009)
1k-cell, 3 hpf (EV08011)
5 hpf (EV04010)
5 hpf (EV07012)
5 hpf (EV08014)
bud, 10 hpf (EV04013)
bud, 10 hpf (EV07014)
bud, 10 hpf (EV08017)
24 hpf (EV03008)
24 hpf (EV07017)
24 hpf (EV08020)
read count
references composition
alignment position (canonical position)
a
c
g
t
-
A
C
G
T

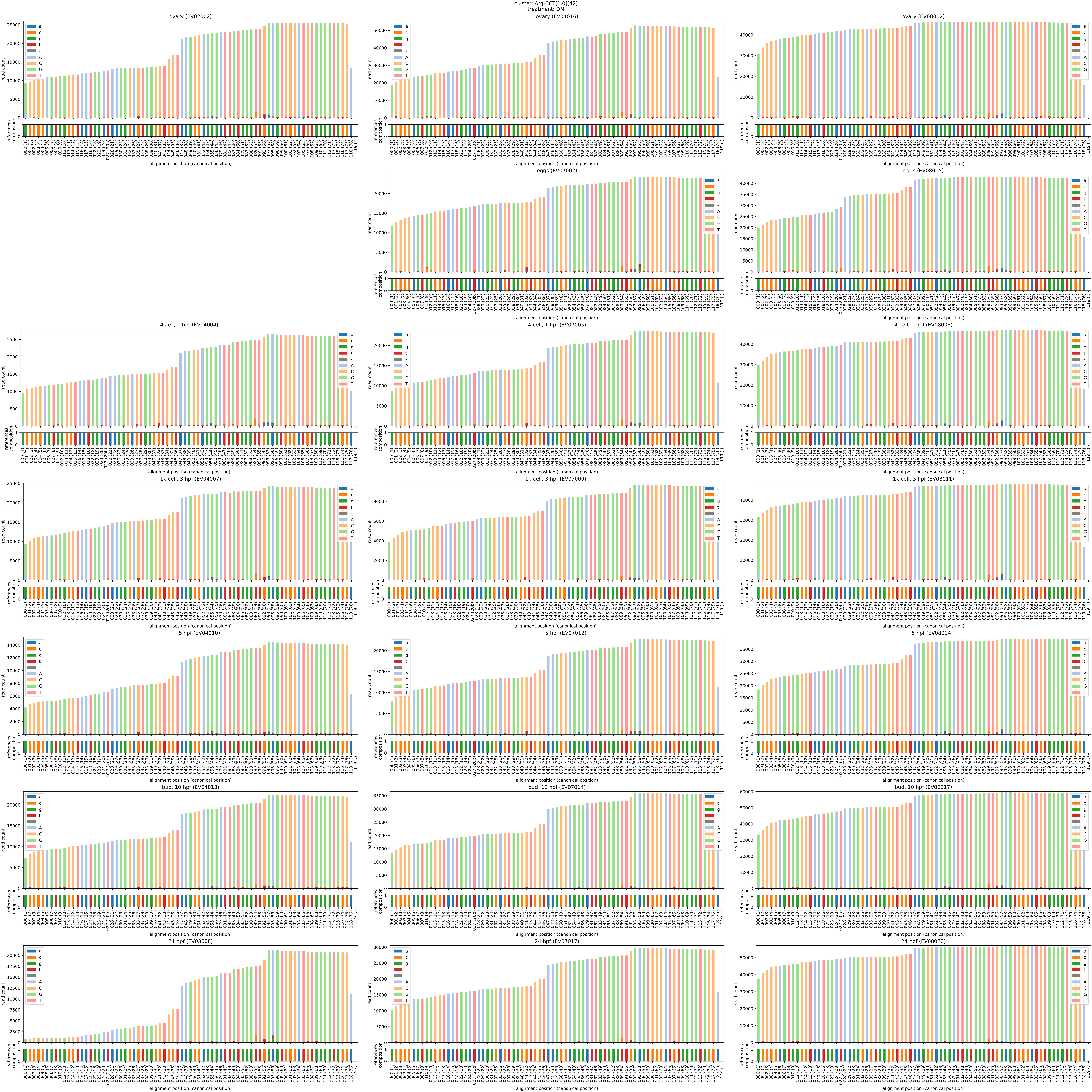
cluster: Arg-CCT[1.0](42)
treatment: DM
ovary (EV02002)
ovary (EV04016)
ovary (EV08002)
eggs (EV07002)
eggs (EV08005)
4-cell, 1 hpf (EV04004)
4-cell, 1 hpf (EV07005)
4-cell, 1 hpf (EV08008)
1k-cell, 3 hpf (EV04007)
1k-cell, 3 hpf (EV07009)
1k-cell, 3 hpf (EV08011)
5 hpf (EV04010)
5 hpf (EV07012)
5 hpf (EV08014)
bud, 10 hpf (EV04013)
bud, 10 hpf (EV07014)
bud, 10 hpf (EV08017)
24 hpf (EV03008)
24 hpf (EV07017)
24 hpf (EV08020)
read count
references composition
alignment position (canonical position)
a
c
g
t
-
A
C
G
T

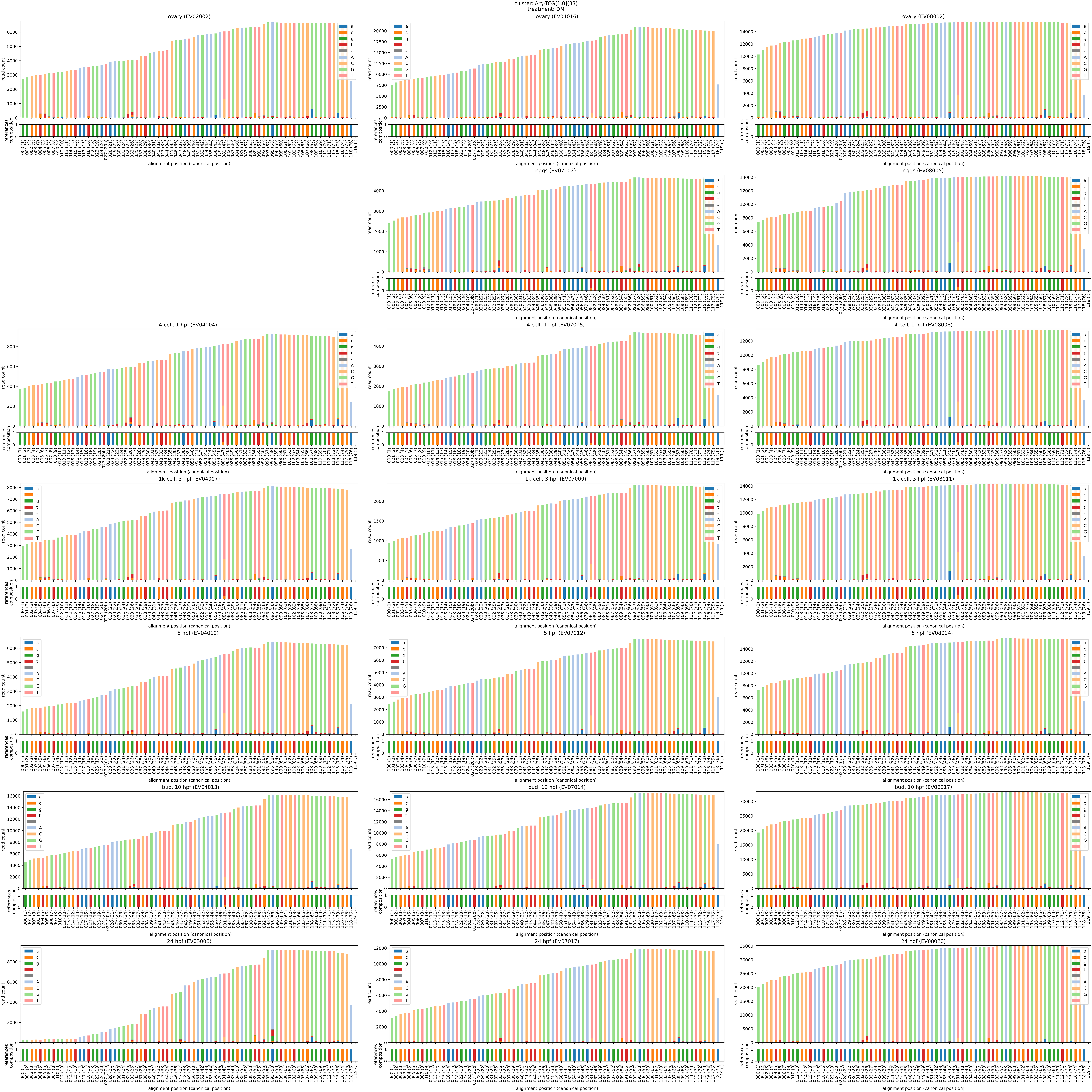
cluster: Arg-TCG[1.0](33)
treatment: DM
ovary (EV02002)
ovary (EV04016)
ovary (EV08002)
eggs (EV07002)
eggs (EV08005)
4-cell, 1 hpf (EV04004)
4-cell, 1 hpf (EV07005)
4-cell, 1 hpf (EV08008)
1k-cell, 3 hpf (EV04007)
1k-cell, 3 hpf (EV07009)
1k-cell, 3 hpf (EV08011)
5 hpf (EV04010)
5 hpf (EV07012)
5 hpf (EV08014)
bud, 10 hpf (EV04013)
bud, 10 hpf (EV07014)
bud, 10 hpf (EV08017)
24 hpf (EV03008)
24 hpf (EV07017)
24 hpf (EV08020)
read count
references composition
alignment position (canonical position)
a
c
g
t
-
A
C
G
T

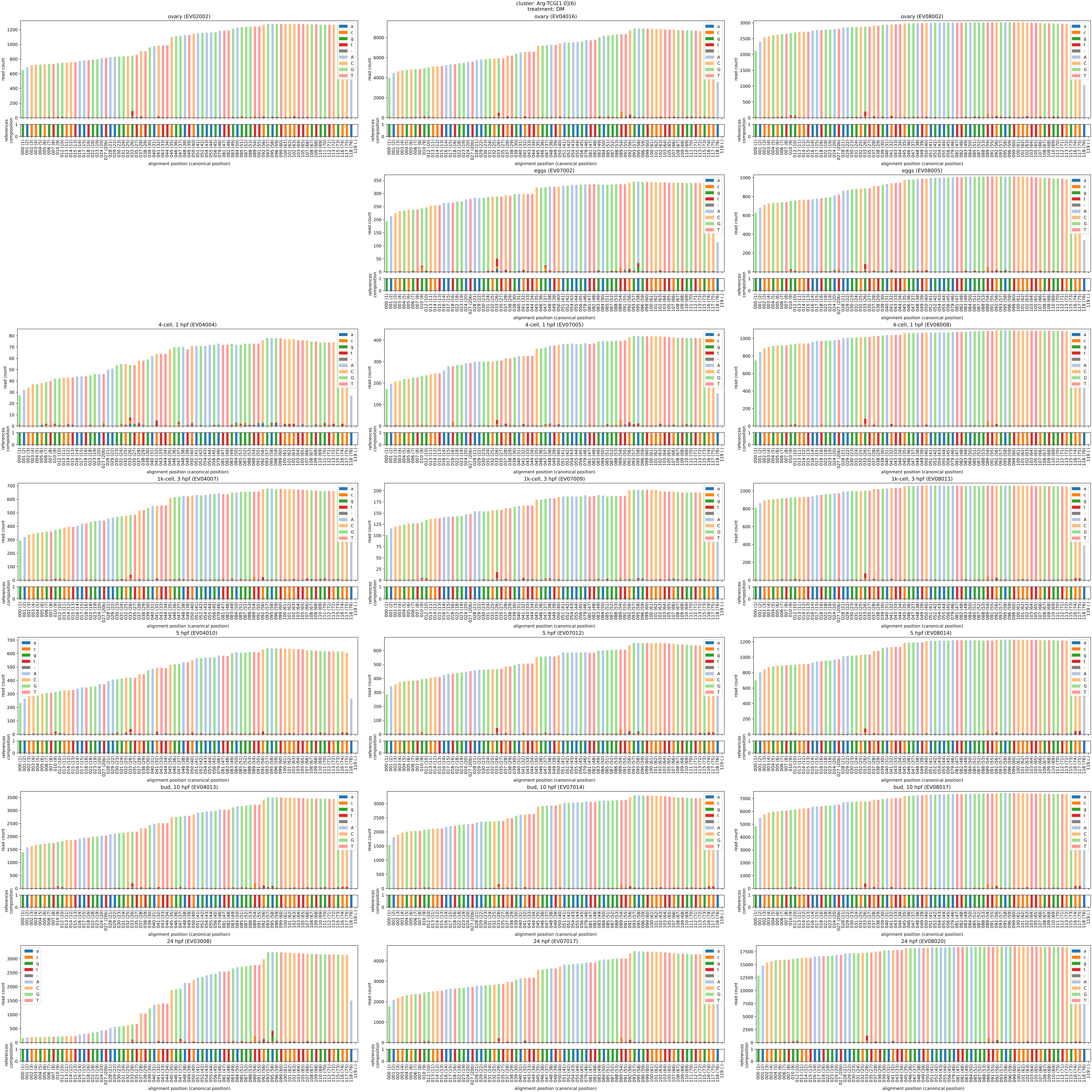
cluster: Arg-TCG[1.0](6)
treatment: DM
ovary (EV02002)
ovary (EV04016)
ovary (EV08002)
eggs (EV07002)
eggs (EV08005)
4-cell, 1 hpf (EV04004)
4-cell, 1 hpf (EV07005)
4-cell, 1 hpf (EV08008)
1k-cell, 3 hpf (EV04007)
1k-cell, 3 hpf (EV07009)
1k-cell, 3 hpf (EV08011)
5 hpf (EV04010)
5 hpf (EV07012)
5 hpf (EV08014)
bud, 10 hpf (EV04013)
bud, 10 hpf (EV07014)
bud, 10 hpf (EV08017)
24 hpf (EV03008)
24 hpf (EV07017)
24 hpf (EV08020)
read count
references composition
alignment position (canonical position)
a
c
g
t
-
A
C
G
T

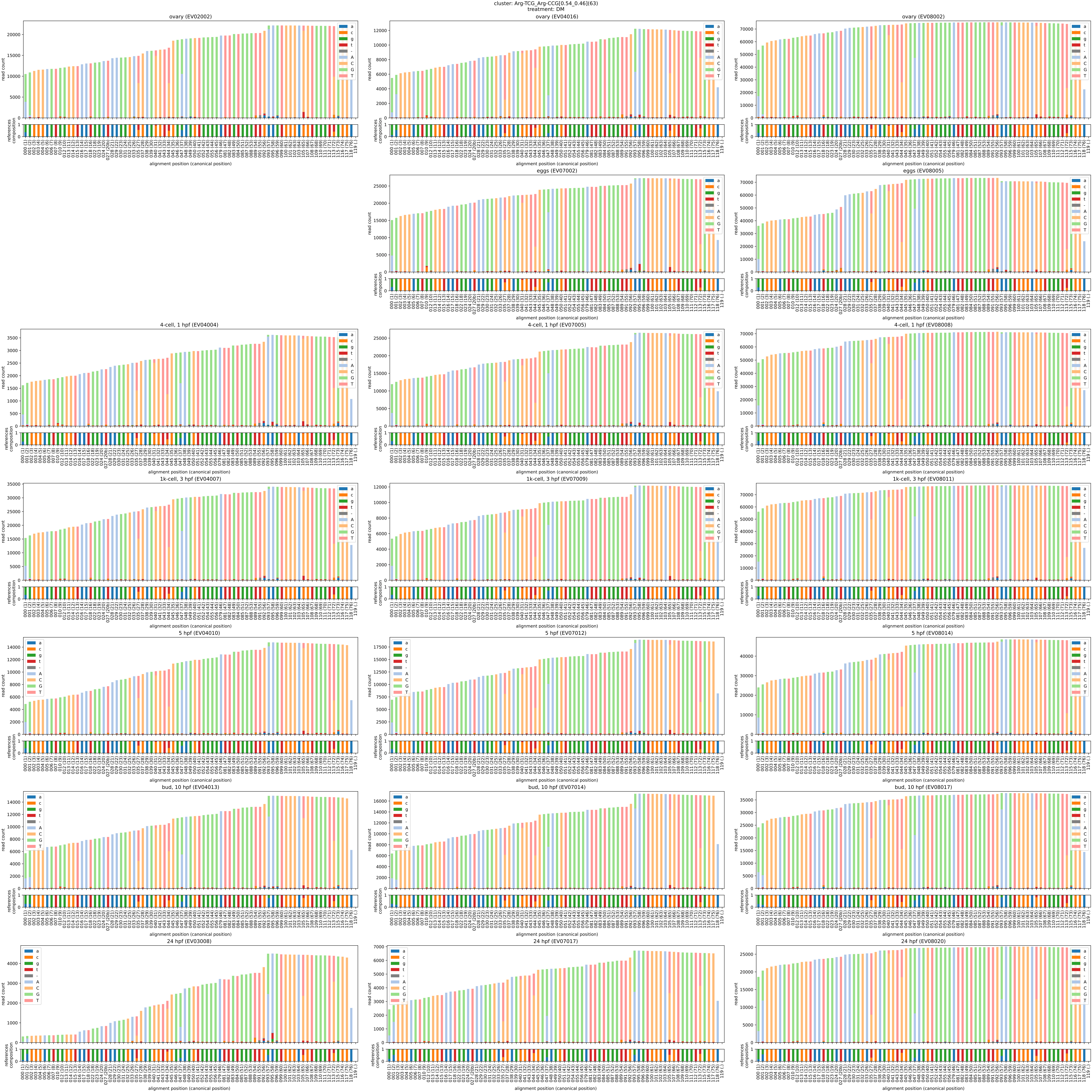
cluster: Arg-TCG_Arg-CCG[0.54_0.46](63)
treatment: DM
ovary (EV02002)
ovary (EV04016)
ovary (EV08002)
eggs (EV07002)
eggs (EV08005)
4-cell, 1 hpf (EV04004)
4-cell, 1 hpf (EV07005)
4-cell, 1 hpf (EV08008)
1k-cell, 3 hpf (EV04007)
1k-cell, 3 hpf (EV07009)
1k-cell, 3 hpf (EV08011)
5 hpf (EV04010)
5 hpf (EV07012)
5 hpf (EV08014)
bud, 10 hpf (EV04013)
bud, 10 hpf (EV07014)
bud, 10 hpf (EV08017)
24 hpf (EV03008)
24 hpf (EV07017)
24 hpf (EV08020)
read count
references composition
alignment position (canonical position)
a
c
g
t
-
A
C
G
T

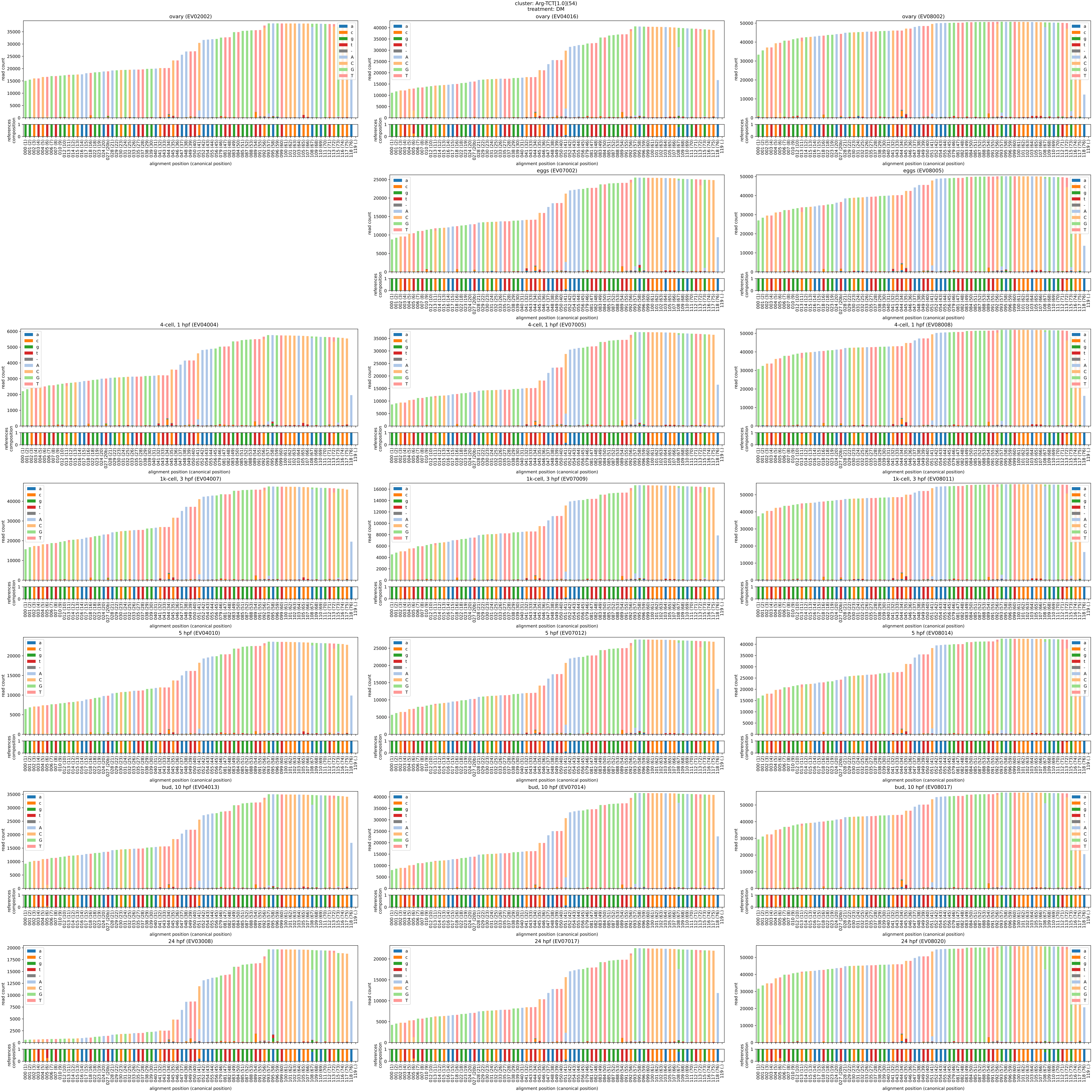
cluster: Arg-TCT[1.0](54)
treatment: DM
ovary (EV02002)
ovary (EV04016)
ovary (EV08002)
eggs (EV07002)
eggs (EV08005)
4-cell, 1 hpf (EV04004)
4-cell, 1 hpf (EV07005)
4-cell, 1 hpf (EV08008)
1k-cell, 3 hpf (EV04007)
1k-cell, 3 hpf (EV07009)
1k-cell, 3 hpf (EV08011)
5 hpf (EV04010)
5 hpf (EV07012)
5 hpf (EV08014)
bud, 10 hpf (EV04013)
bud, 10 hpf (EV07014)
bud, 10 hpf (EV08017)
24 hpf (EV03008)
24 hpf (EV07017)
24 hpf (EV08020)
read count
references composition
alignment position (canonical position)
a
c
g
t
-
A
C
G
T

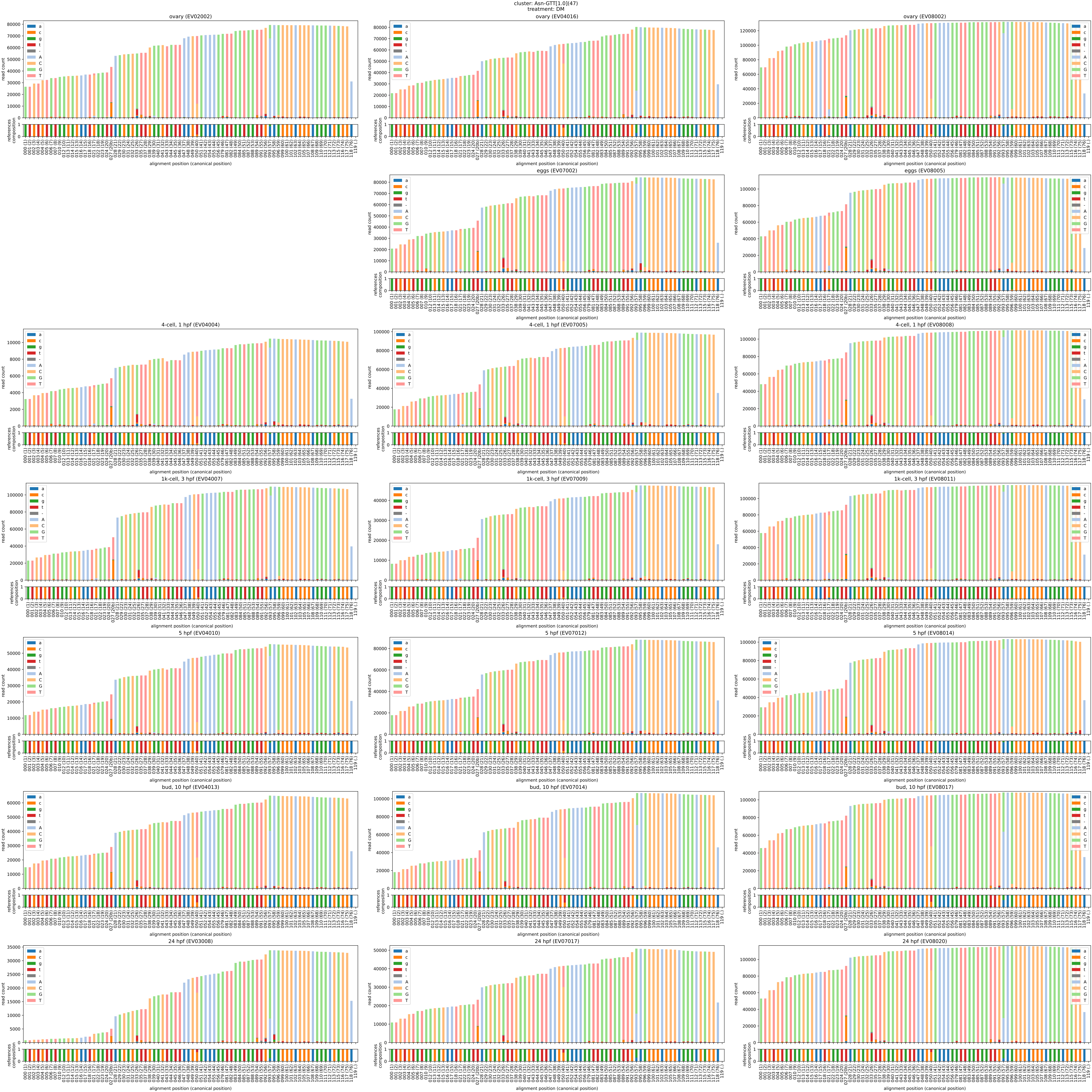

cluster: Asn-GTT[1.0](47)
treatment: DM
ovary (EV02002)
ovary (EV04016)
ovary (EV08002)
eggs (EV07002)
eggs (EV08005)
4-cell, 1 hpf (EV04004)
4-cell, 1 hpf (EV07005)
4-cell, 1 hpf (EV08008)
1k-cell, 3 hpf (EV04007)
1k-cell, 3 hpf (EV07009)
1k-cell, 3 hpf (EV08011)
5 hpf (EV04010)
5 hpf (EV07012)
5 hpf (EV08014)
bud, 10 hpf (EV04013)
bud, 10 hpf (EV07014)
bud, 10 hpf (EV08017)
24 hpf (EV03008)
24 hpf (EV07017)
24 hpf (EV08020)
read count
references composition
alignment position (canonical position)
a
c
g
t
-
A
C
G
T

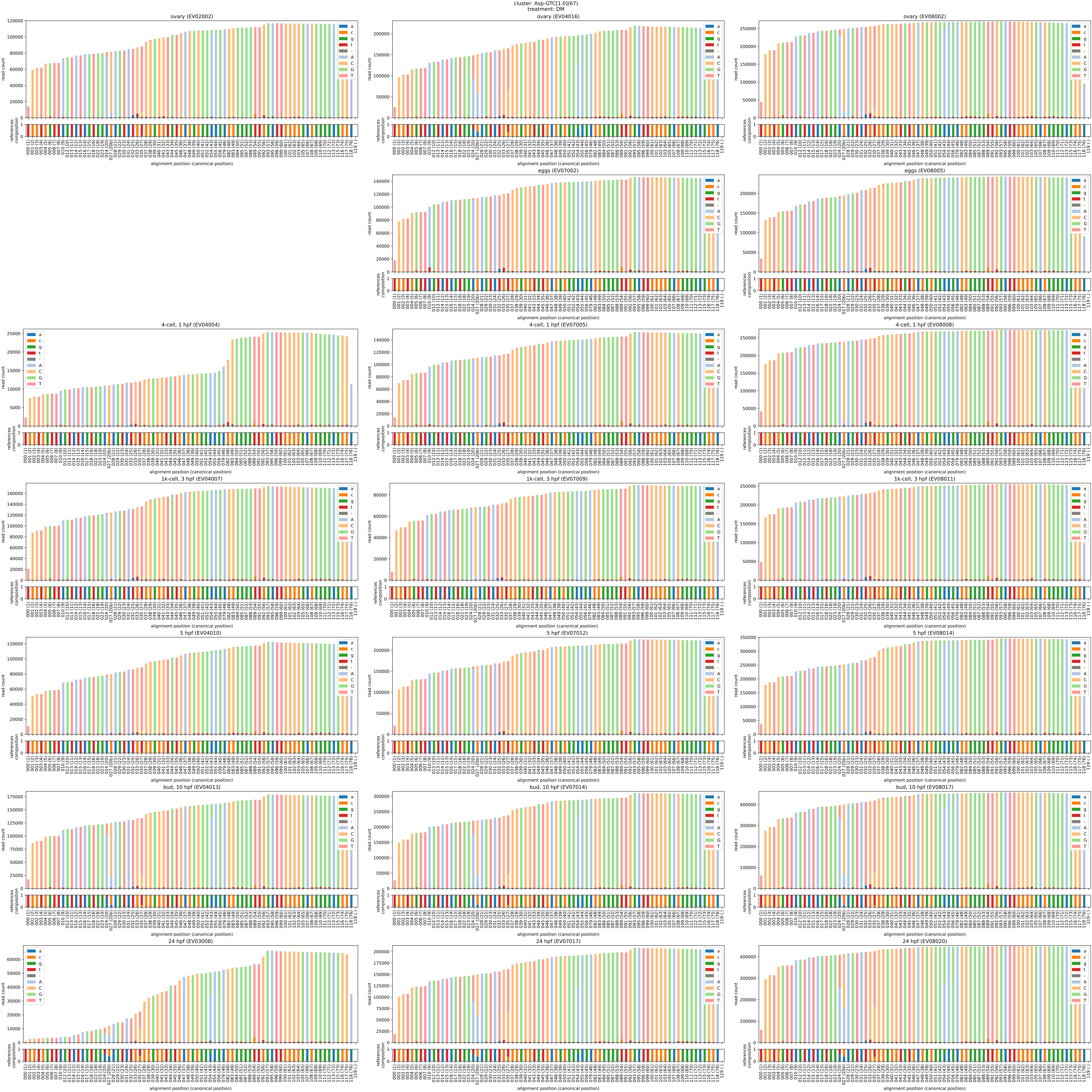
cluster: Asp-GTC[1.0](67)
treatment: DM
ovary (EV02002)
ovary (EV04016)
ovary (EV08002)
eggs (EV07002)
eggs (EV08005)
4-cell, 1 hpf (EV04004)
4-cell, 1 hpf (EV07005)
4-cell, 1 hpf (EV08008)
1k-cell, 3 hpf (EV04007)
1k-cell, 3 hpf (EV07009)
1k-cell, 3 hpf (EV08011)
5 hpf (EV04010)
5 hpf (EV07012)
5 hpf (EV08014)
bud, 10 hpf (EV04013)
bud, 10 hpf (EV07014)
bud, 10 hpf (EV08017)
24 hpf (EV03008)
24 hpf (EV07017)
24 hpf (EV08020)
read count
references composition
alignment position (canonical position)
a
c
g
t
-
A
C
G
T

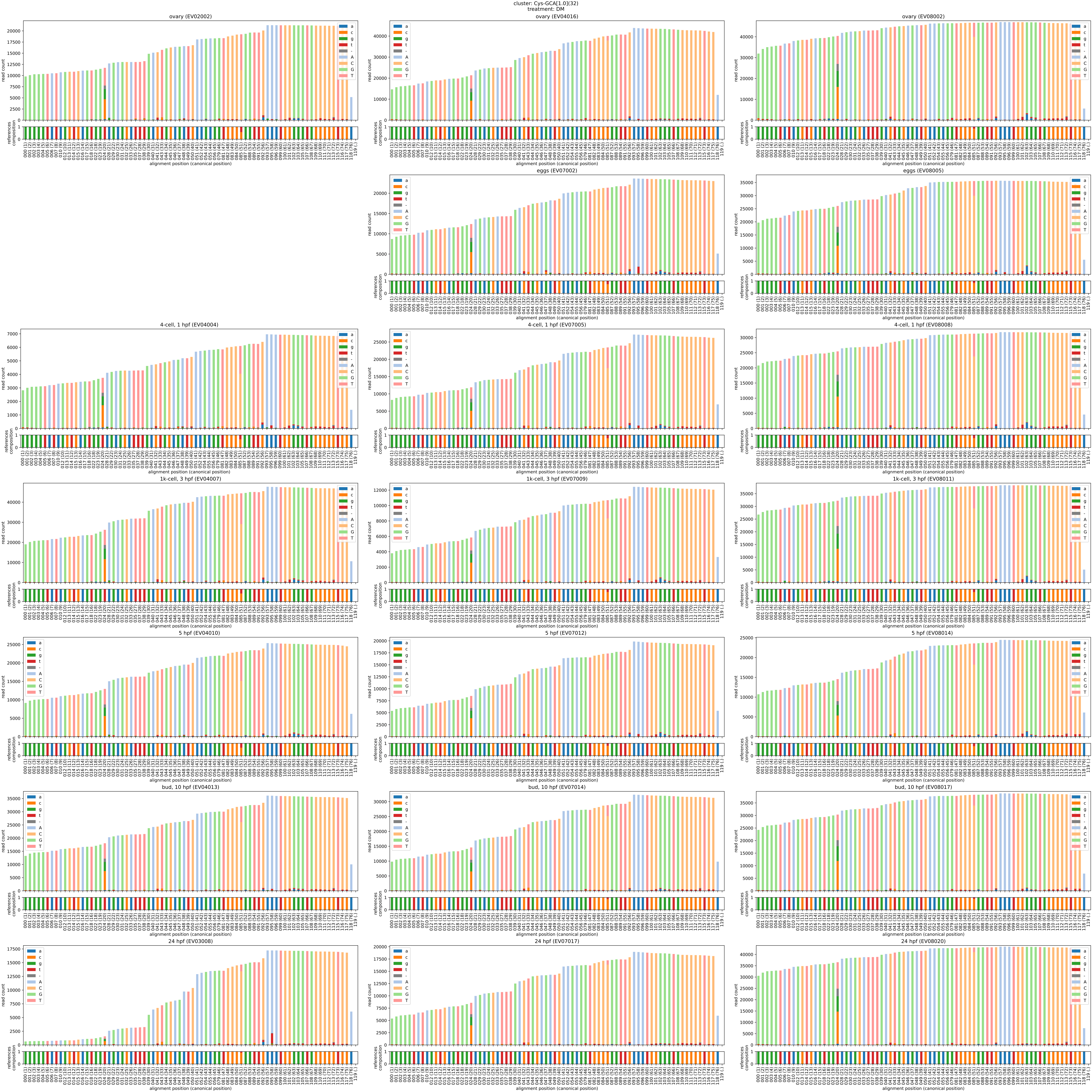
cluster: Cys-GCA[1.0](32)
treatment: DM
ovary (EV02002)
ovary (EV04016)
ovary (EV08002)
eggs (EV07002)
eggs (EV08005)
4-cell, 1 hpf (EV04004)
4-cell, 1 hpf (EV07005)
4-cell, 1 hpf (EV08008)
1k-cell, 3 hpf (EV04007)
1k-cell, 3 hpf (EV07009)
1k-cell, 3 hpf (EV08011)
5 hpf (EV04010)
5 hpf (EV07012)
5 hpf (EV08014)
bud, 10 hpf (EV04013)
bud, 10 hpf (EV07014)
bud, 10 hpf (EV08017)
24 hpf (EV03008)
24 hpf (EV07017)
24 hpf (EV08020)
read count
references composition
alignment position (canonical position)
a
c
g
t
-
A
C
G
T

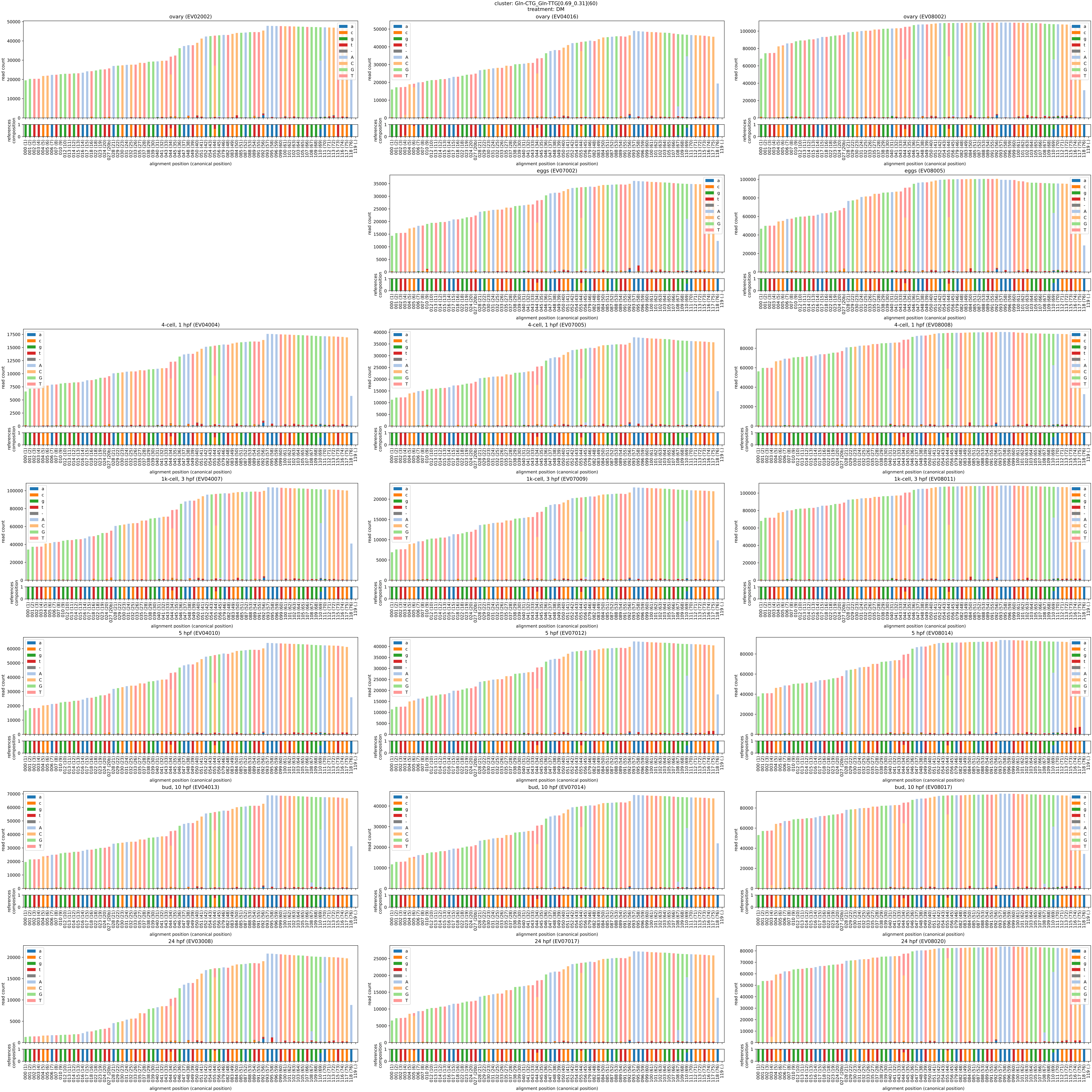
cluster: Gln-CTG_Gln-TTG[0.69_0.31](60)
treatment: DM
ovary (EV02002)
ovary (EV04016)
ovary (EV08002)
eggs (EV07002)
eggs (EV08005)
4-cell, 1 hpf (EV04004)
4-cell, 1 hpf (EV07005)
4-cell, 1 hpf (EV08008)
1k-cell, 3 hpf (EV04007)
1k-cell, 3 hpf (EV07009)
1k-cell, 3 hpf (EV08011)
5 hpf (EV04010)
5 hpf (EV07012)
5 hpf (EV08014)
bud, 10 hpf (EV04013)
bud, 10 hpf (EV07014)
bud, 10 hpf (EV08017)
24 hpf (EV03008)
24 hpf (EV07017)
24 hpf (EV08020)
read count
references composition
alignment position (canonical position)
a
c
g
t
-
A
C
G
T

cluster: Glu-CTC[1.0](49)
treatment: DM

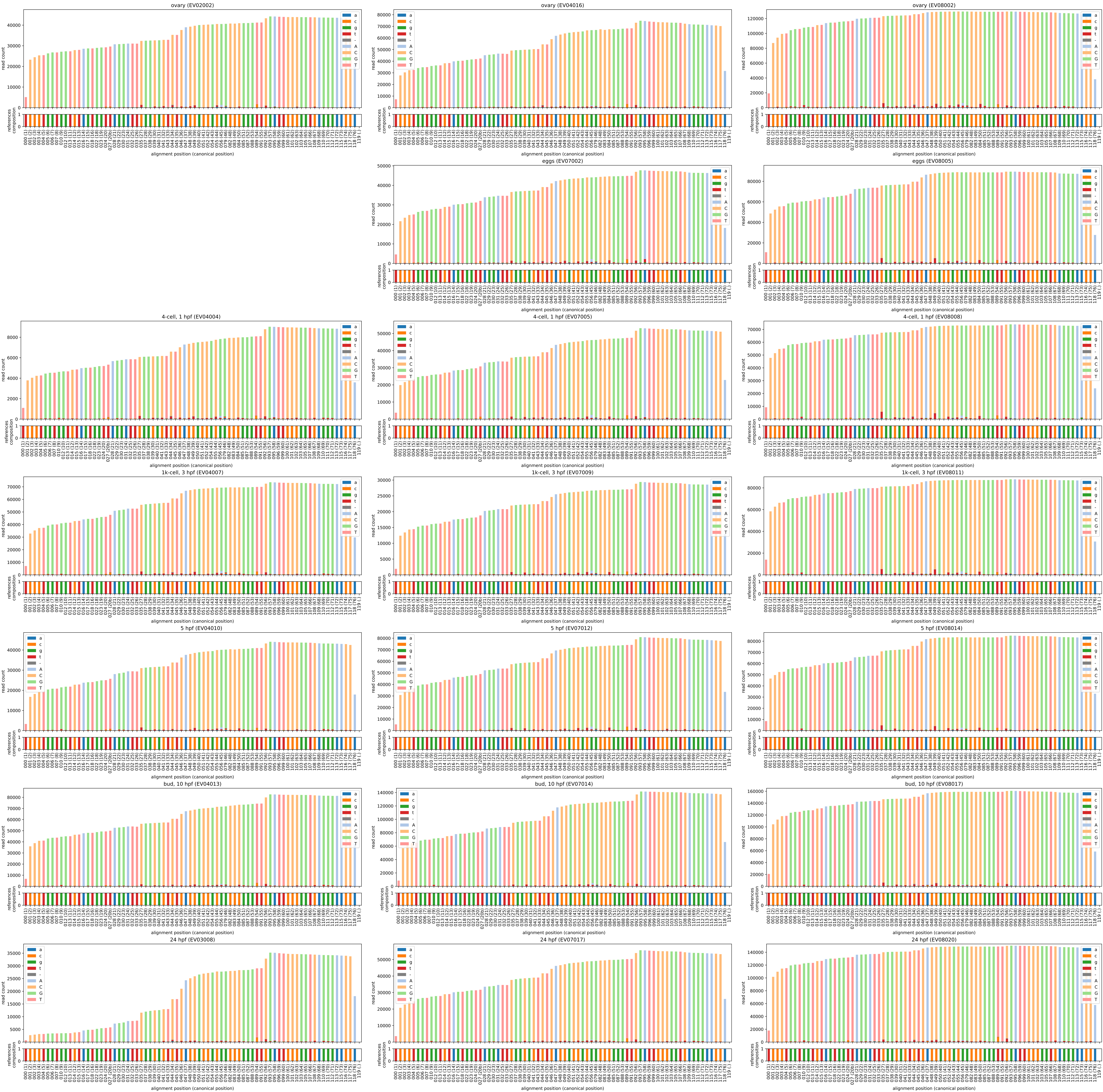

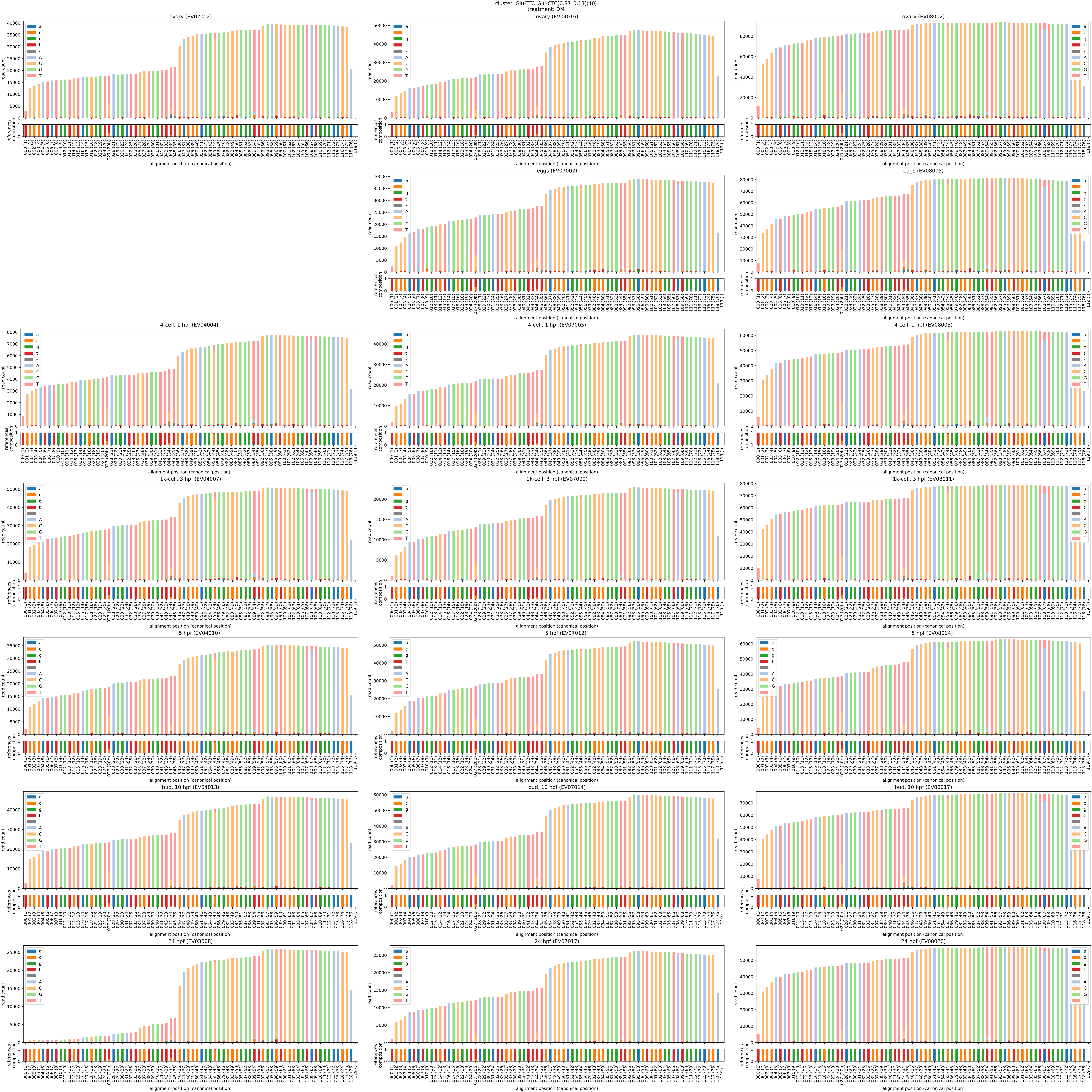

cluster: Glu-TTC_Glu-CTC[0.87_0.13](40)
treatment: DM
ovary (EV02002)
ovary (EV04016)
ovary (EV08002)
eggs (EV07002)
eggs (EV08005)
4-cell, 1 hpf (EV04004)
4-cell, 1 hpf (EV07005)
4-cell, 1 hpf (EV08008)
1k-cell, 3 hpf (EV04007)
1k-cell, 3 hpf (EV07009)
1k-cell, 3 hpf (EV08011)
5 hpf (EV04010)
5 hpf (EV07012)
5 hpf (EV08014)
bud, 10 hpf (EV04013)
bud, 10 hpf (EV07014)
bud, 10 hpf (EV08017)
24 hpf (EV03008)
24 hpf (EV07017)
24 hpf (EV08020)
read count
references composition
alignment position (canonical position)
a
c
g
t
-
A
C
G
T

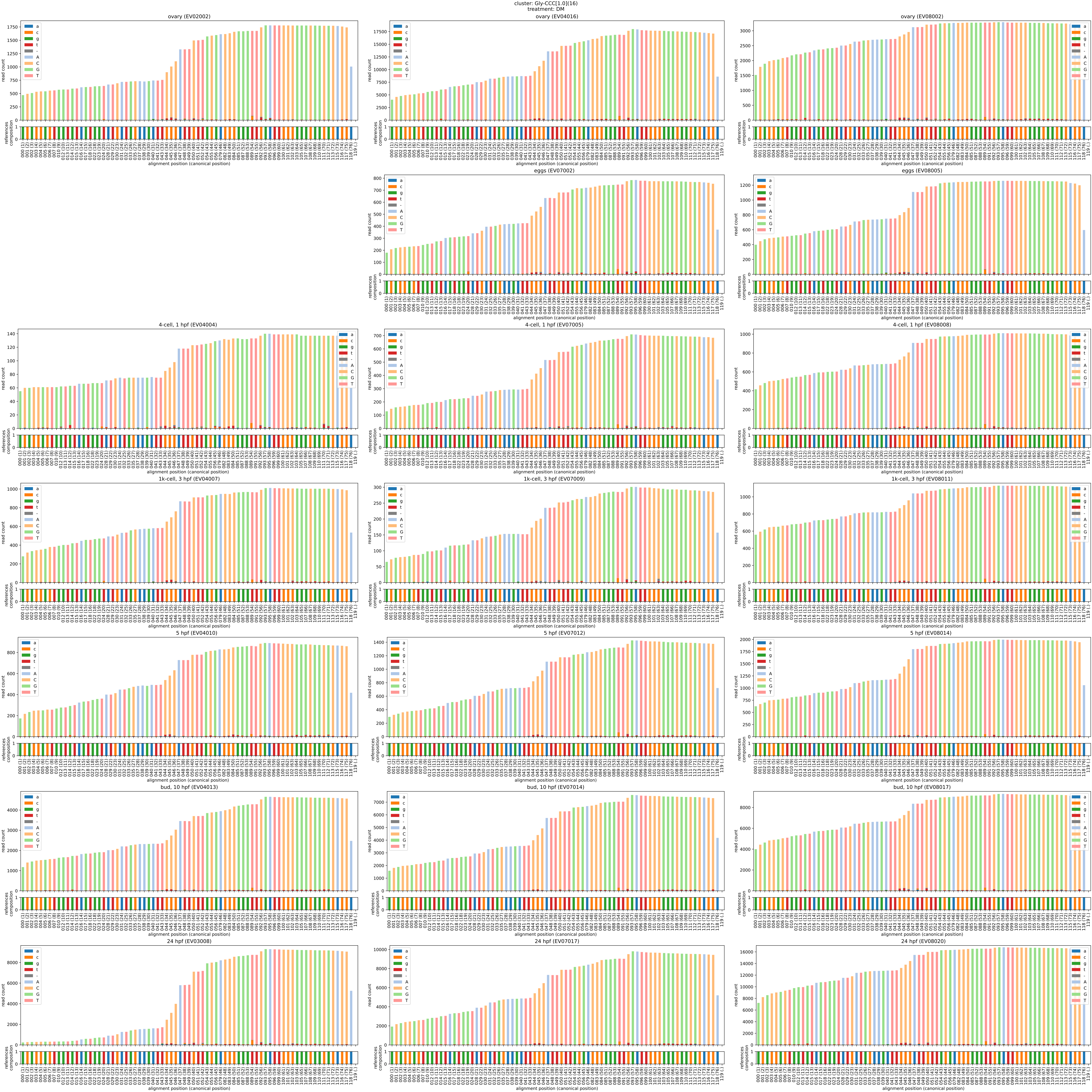
cluster: Gly-CCC[1.0](16)
treatment: DM
ovary (EV02002)
ovary (EV04016)
ovary (EV08002)
eggs (EV07002)
eggs (EV08005)
4-cell, 1 hpf (EV04004)
4-cell, 1 hpf (EV07005)
4-cell, 1 hpf (EV08008)
1k-cell, 3 hpf (EV04007)
1k-cell, 3 hpf (EV07009)
1k-cell, 3 hpf (EV08011)
5 hpf (EV04010)
5 hpf (EV07012)
5 hpf (EV08014)
bud, 10 hpf (EV04013)
bud, 10 hpf (EV07014)
bud, 10 hpf (EV08017)
24 hpf (EV03008)
24 hpf (EV07017)
24 hpf (EV08020)
a
c
g
t
-
A
C
G
T
read count
references composition
alignment position (canonical position)

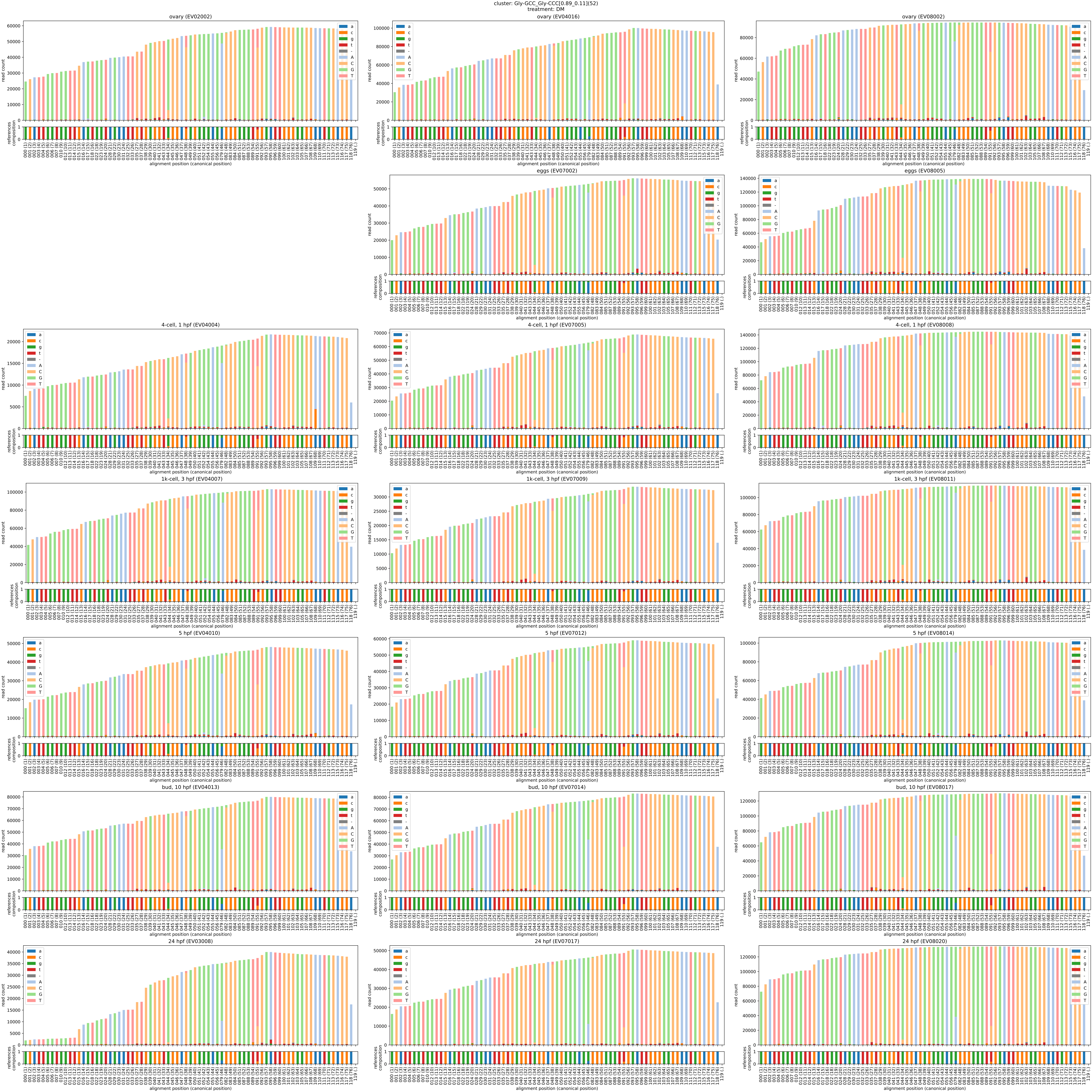
cluster: Gly-GCC_Gly-CCC[0.89_0.11](52)
treatment: DM
ovary (EV02002)
ovary (EV04016)
ovary (EV08002)
eggs (EV07002)
eggs (EV08005)
4-cell, 1 hpf (EV04004)
4-cell, 1 hpf (EV07005)
4-cell, 1 hpf (EV08008)
1k-cell, 3 hpf (EV04007)
1k-cell, 3 hpf (EV07009)
1k-cell, 3 hpf (EV08011)
5 hpf (EV04010)
5 hpf (EV07012)
5 hpf (EV08014)
bud, 10 hpf (EV04013)
bud, 10 hpf (EV07014)
bud, 10 hpf (EV08017)
24 hpf (EV03008)
24 hpf (EV07017)
24 hpf (EV08020)
read count
references composition
alignment position (canonical position)
a
c
g
t
-
A
C
G
T

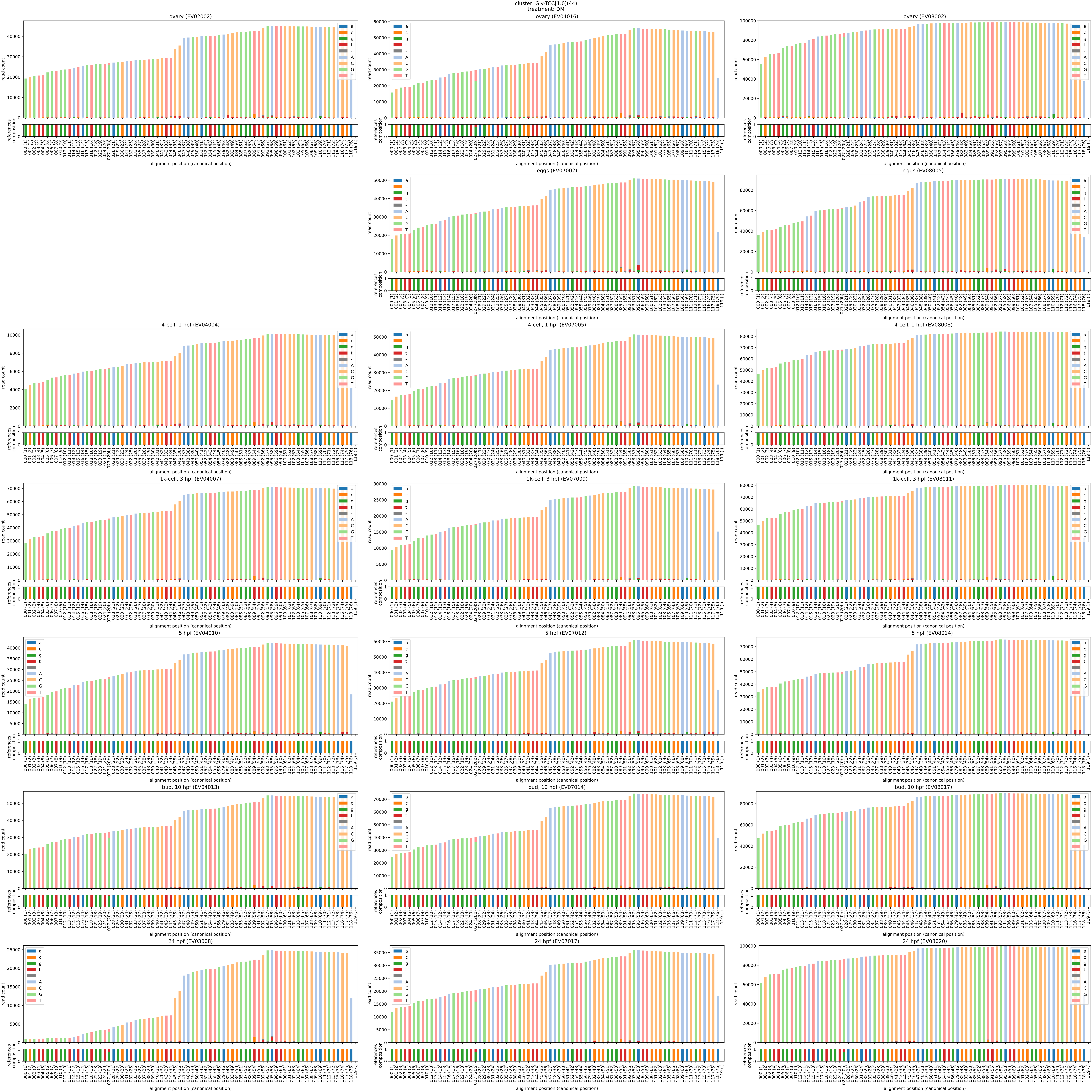
cluster: Gly-TCC[1.0](44)
treatment: DM
ovary (EV02002)
ovary (EV04016)
ovary (EV08002)
eggs (EV07002)
eggs (EV08005)
4-cell, 1 hpf (EV04004)
4-cell, 1 hpf (EV07005)
4-cell, 1 hpf (EV08008)
1k-cell, 3 hpf (EV04007)
1k-cell, 3 hpf (EV07009)
1k-cell, 3 hpf (EV08011)
5 hpf (EV04010)
5 hpf (EV07012)
5 hpf (EV08014)
bud, 10 hpf (EV04013)
bud, 10 hpf (EV07014)
bud, 10 hpf (EV08017)
24 hpf (EV03008)
24 hpf (EV07017)
24 hpf (EV08020)
read count
references composition
alignment position (canonical position)
a
c
g
t
-
A
C
G
T

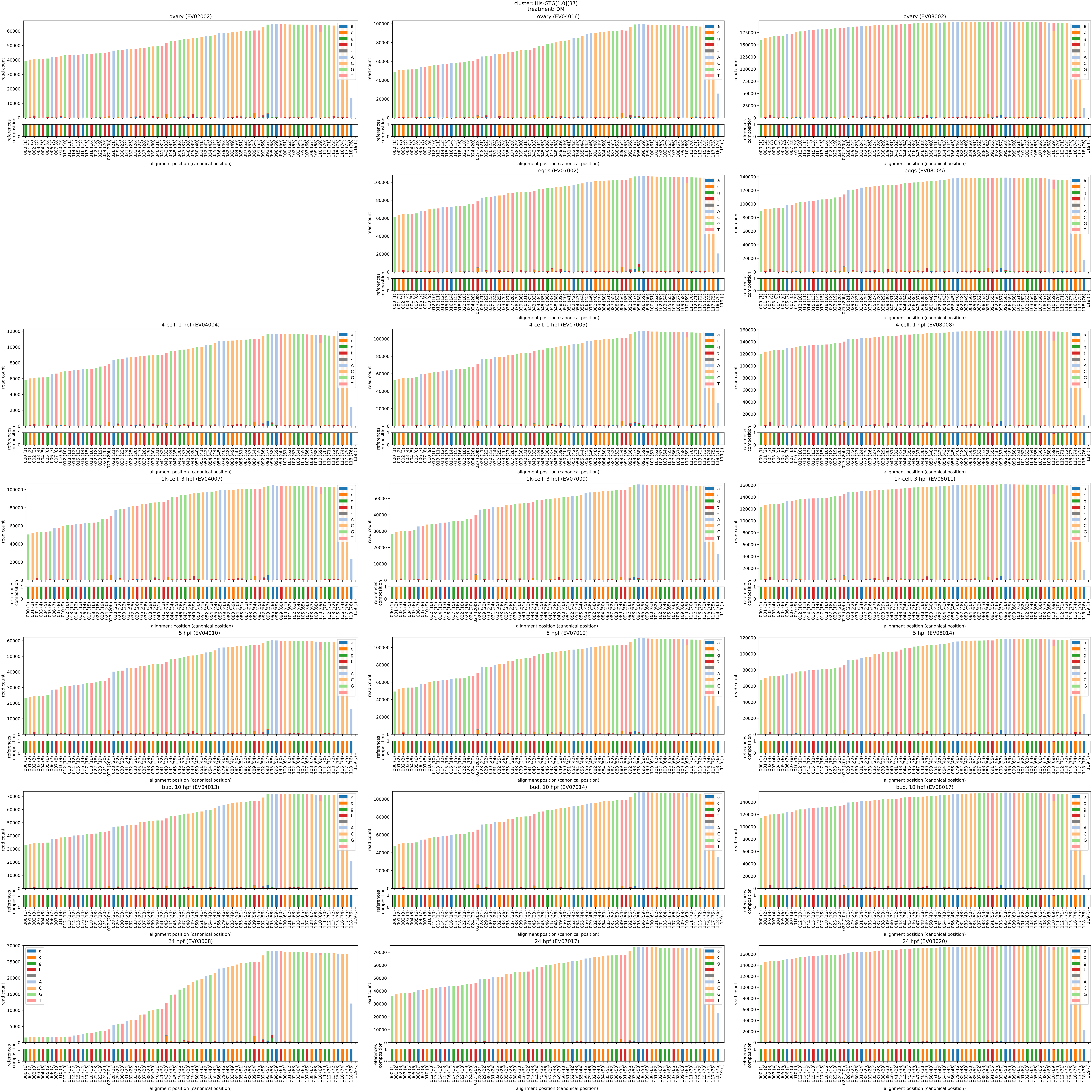

cluster: His-GTG[1.0](37)
treatment: DM
ovary (EV02002)
ovary (EV04016)
ovary (EV08002)
eggs (EV07002)
eggs (EV08005)
4-cell, 1 hpf (EV04004)
4-cell, 1 hpf (EV07005)
4-cell, 1 hpf (EV08008)
1k-cell, 3 hpf (EV04007)
1k-cell, 3 hpf (EV07009)
1k-cell, 3 hpf (EV08011)
5 hpf (EV04010)
5 hpf (EV07012)
5 hpf (EV08014)
bud, 10 hpf (EV04013)
bud, 10 hpf (EV07014)
bud, 10 hpf (EV08017)
24 hpf (EV03008)
24 hpf (EV07017)
24 hpf (EV08020)
read count
references composition
alignment position (canonical position)
a
c
g
t
-
A
C
G
T

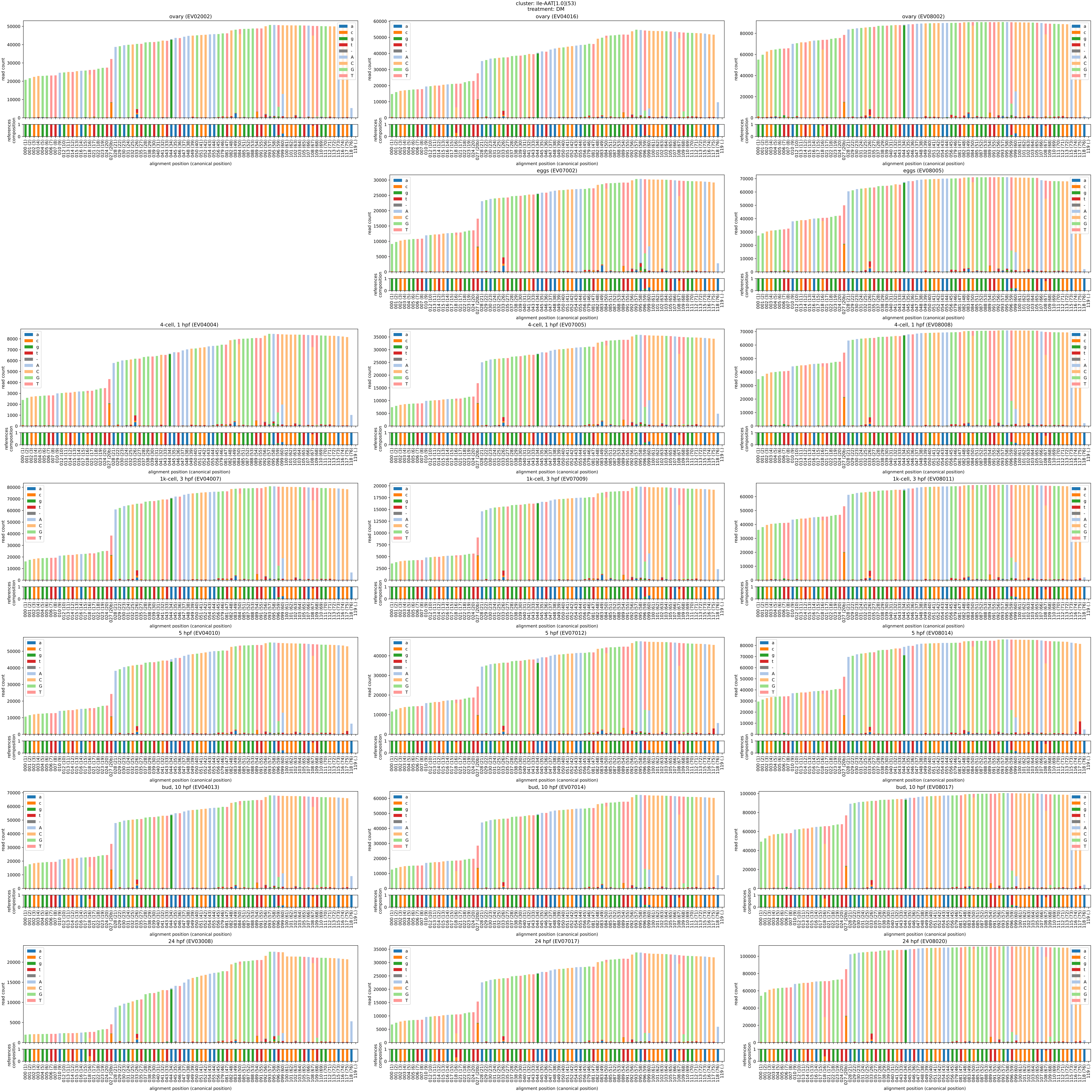

cluster: Ile-AAT[1.0](53)
treatment: DM
ovary (EV02002)
ovary (EV04016)
ovary (EV08002)
eggs (EV07002)
eggs (EV08005)
4-cell, 1 hpf (EV04004)
4-cell, 1 hpf (EV07005)
4-cell, 1 hpf (EV08008)
1k-cell, 3 hpf (EV04007)
1k-cell, 3 hpf (EV07009)
1k-cell, 3 hpf (EV08011)
5 hpf (EV04010)
5 hpf (EV07012)
5 hpf (EV08014)
bud, 10 hpf (EV04013)
bud, 10 hpf (EV07014)
bud, 10 hpf (EV08017)
24 hpf (EV03008)
24 hpf (EV07017)
24 hpf (EV08020)
read count
references composition
alignment position (canonical position)
a
c
g
t
-
A
C
G
T

cluster: Ile-TAT[1.0](43)
treatment: DM

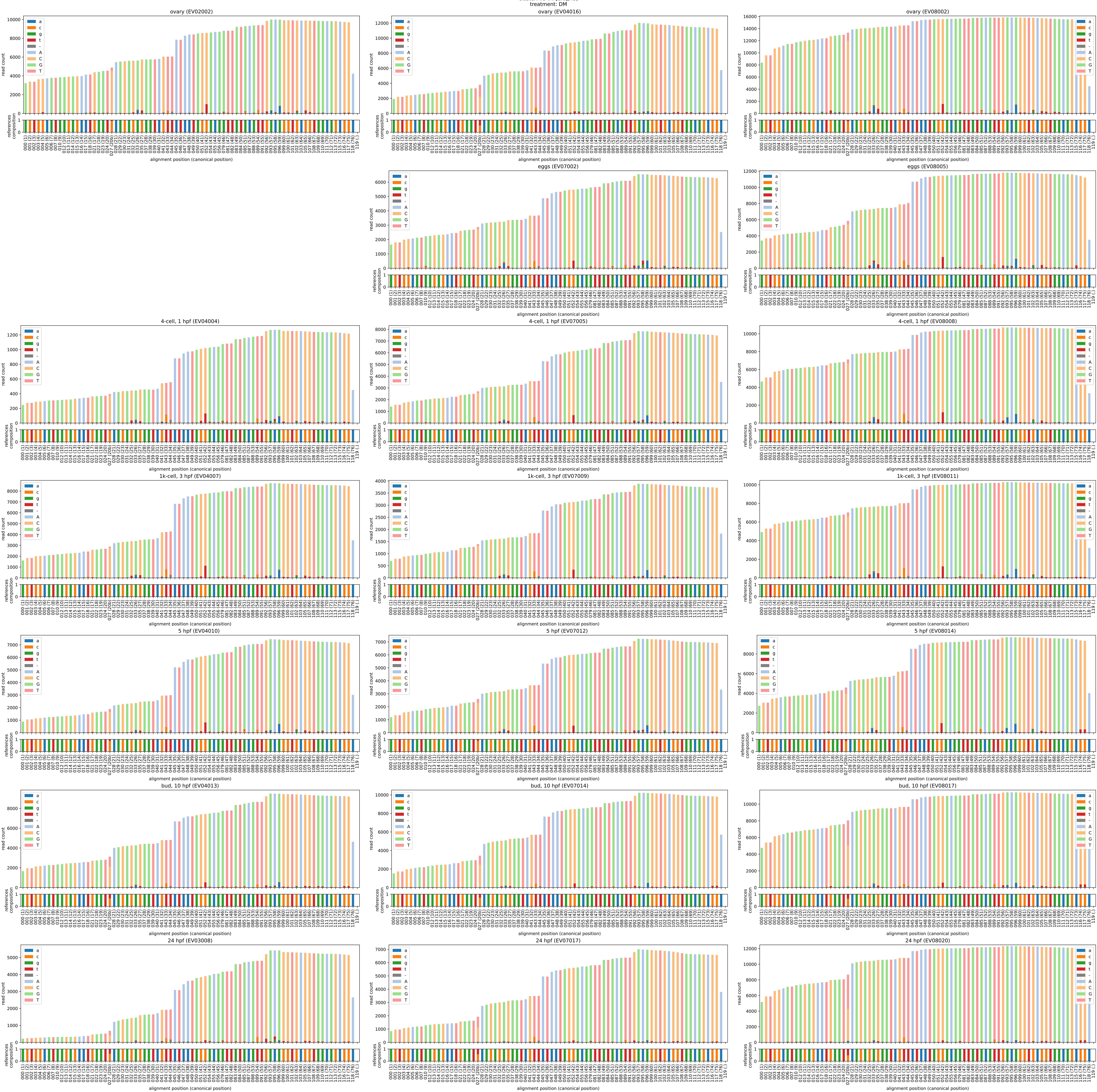

cluster: Leu-CAA[1.0](56)
treatment: DM

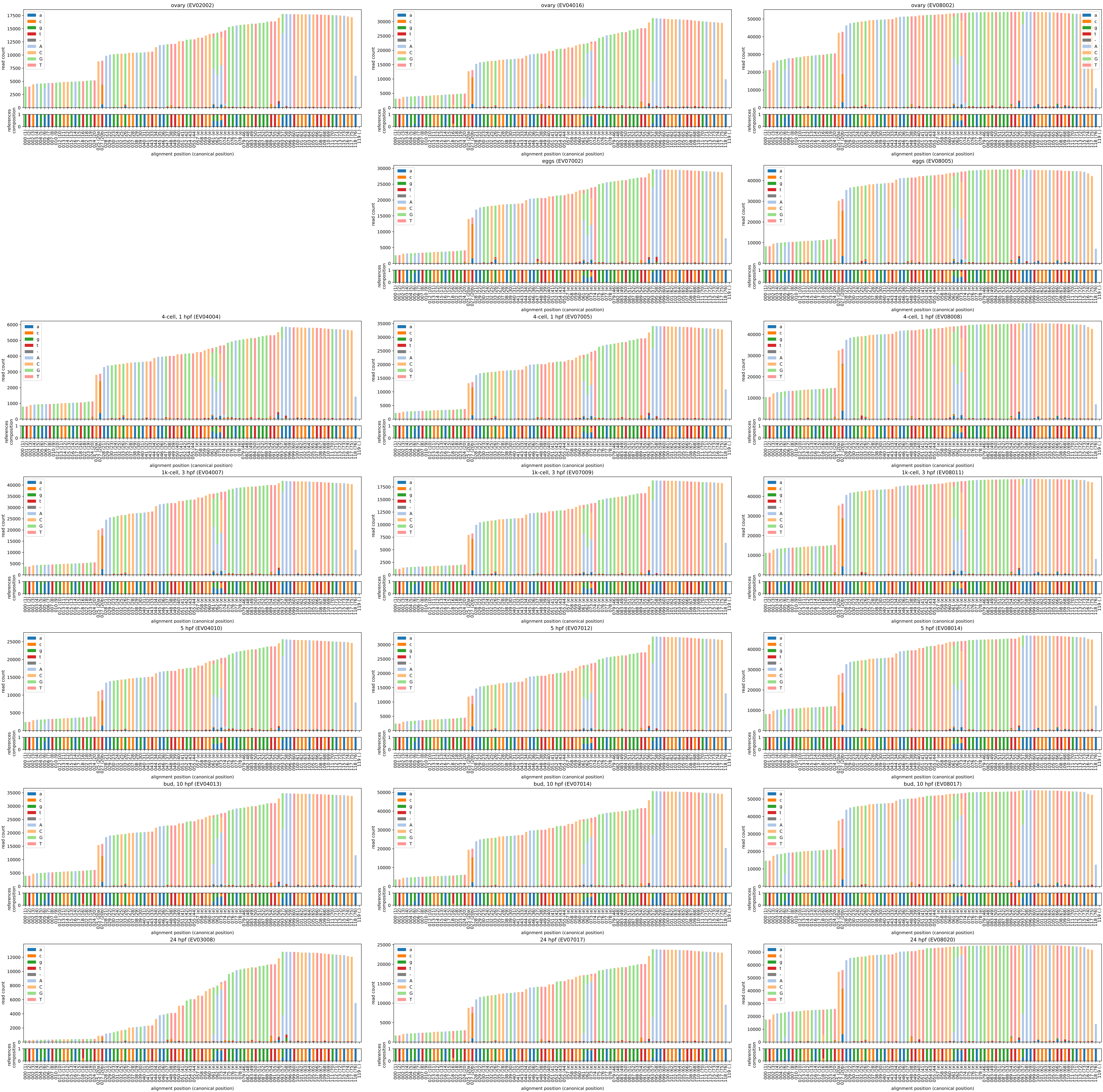

cluster: Leu-CAG[1.0](31)
treatment: DM

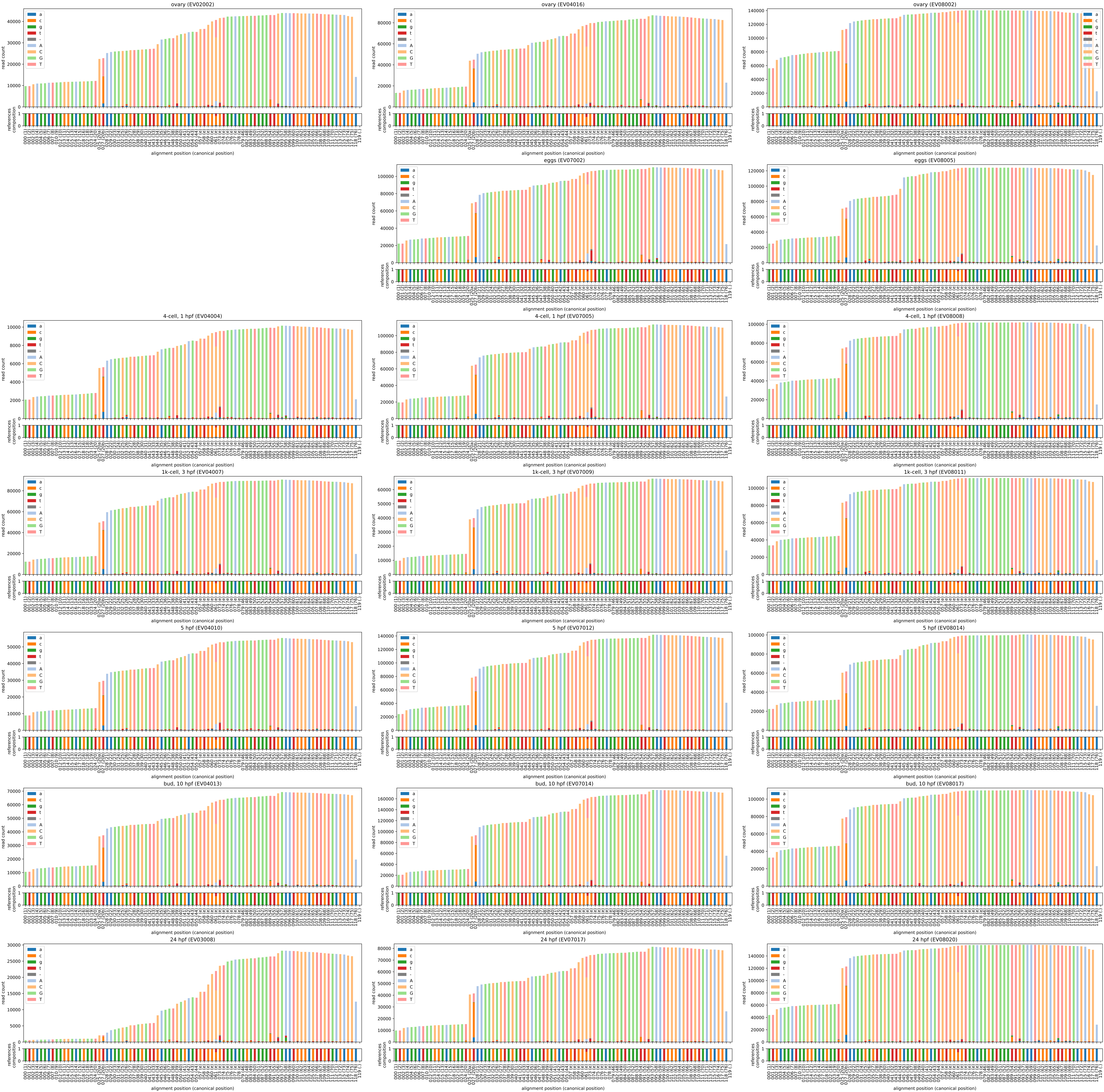

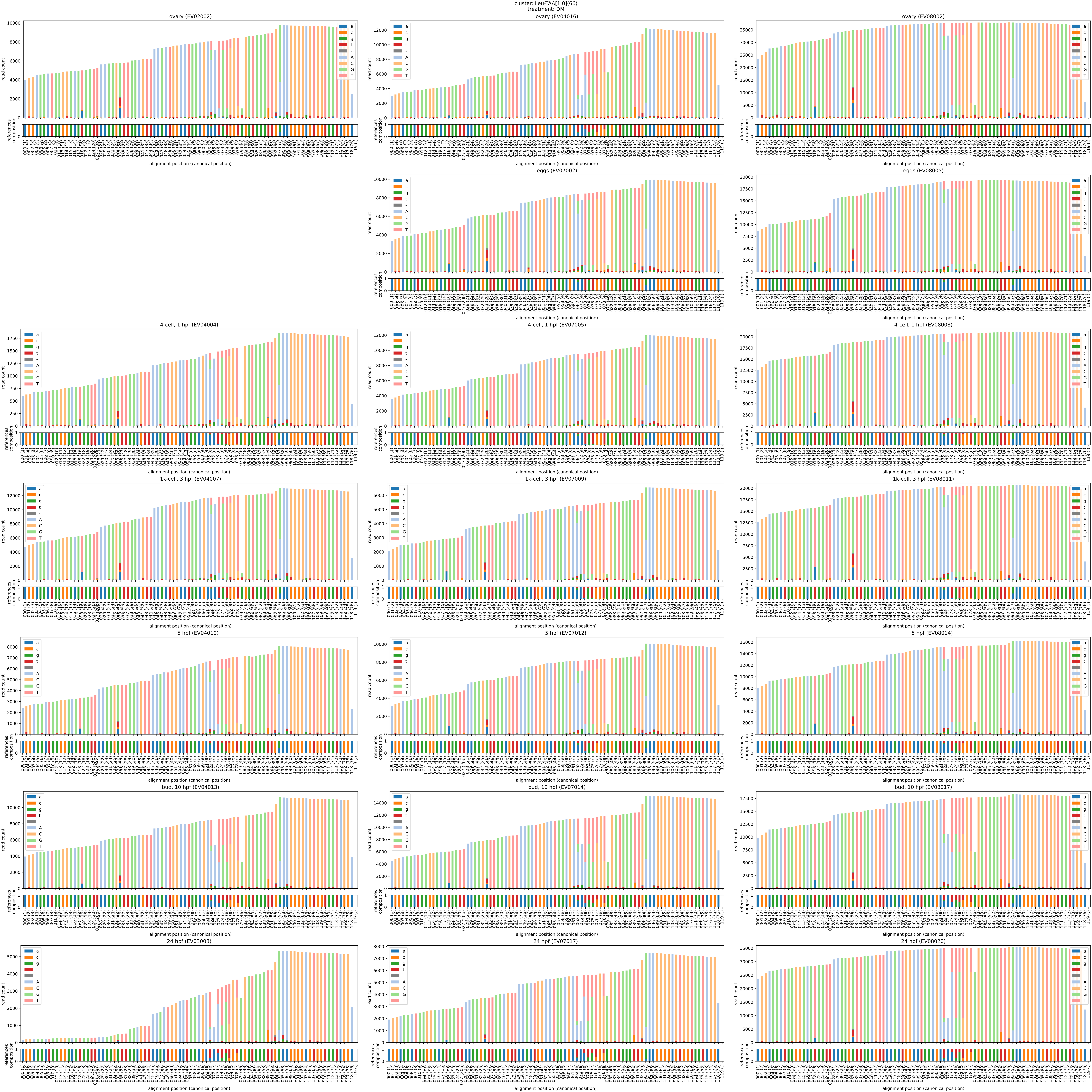
cluster: Leu-TAA[1.0](66)
treatment: DM
ovary (EV02002)
ovary (EV04016)
ovary (EV08002)
eggs (EV07002)
eggs (EV08005)
4-cell, 1 hpf (EV04004)
4-cell, 1 hpf (EV07005)
4-cell, 1 hpf (EV08008)
1k-cell, 3 hpf (EV04007)
1k-cell, 3 hpf (EV07009)
1k-cell, 3 hpf (EV08011)
5 hpf (EV04010)
5 hpf (EV07012)
5 hpf (EV08014)
bud, 10 hpf (EV04013)
bud, 10 hpf (EV07014)
bud, 10 hpf (EV08017)
24 hpf (EV03008)
24 hpf (EV07017)
24 hpf (EV08020)
read count
references composition
alignment position (canonical position)
a
c
g
t
-
A
C
G
T

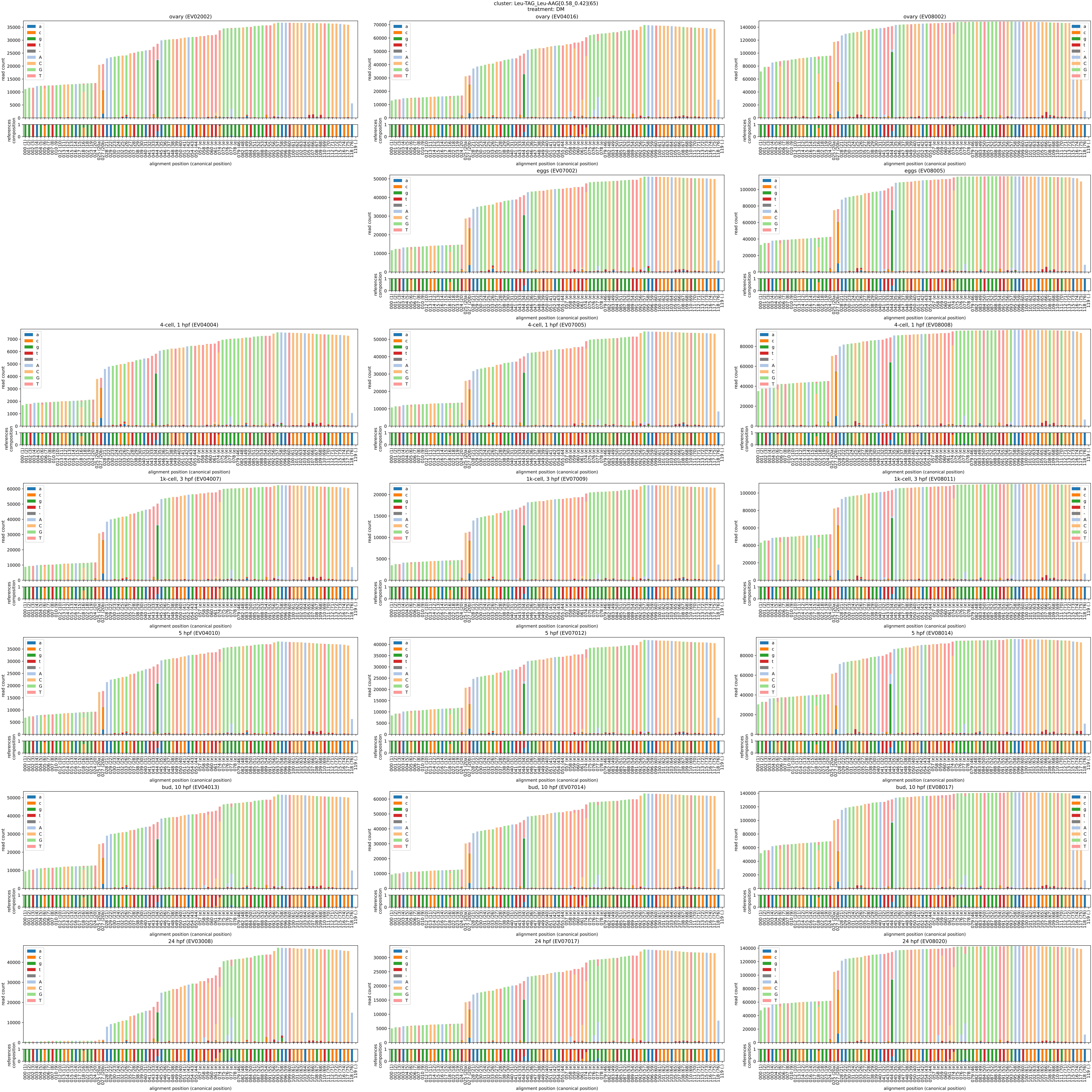
cluster: Leu-TAG_Leu-AAG[0.58_0.42](65)
treatment: DM
ovary (EV02002)
ovary (EV04016)
ovary (EV08002)
eggs (EV07002)
eggs (EV08005)
4-cell, 1 hpf (EV04004)
4-cell, 1 hpf (EV07005)
4-cell, 1 hpf (EV08008)
1k-cell, 3 hpf (EV04007)
1k-cell, 3 hpf (EV07009)
1k-cell, 3 hpf (EV08011)
5 hpf (EV04010)
5 hpf (EV07012)
5 hpf (EV08014)
bud, 10 hpf (EV04013)
bud, 10 hpf (EV07014)
bud, 10 hpf (EV08017)
24 hpf (EV03008)
24 hpf (EV07017)
24 hpf (EV08020)
read count
references composition
alignment position (canonical position)
a
c
g
t
-
A
C
G
T

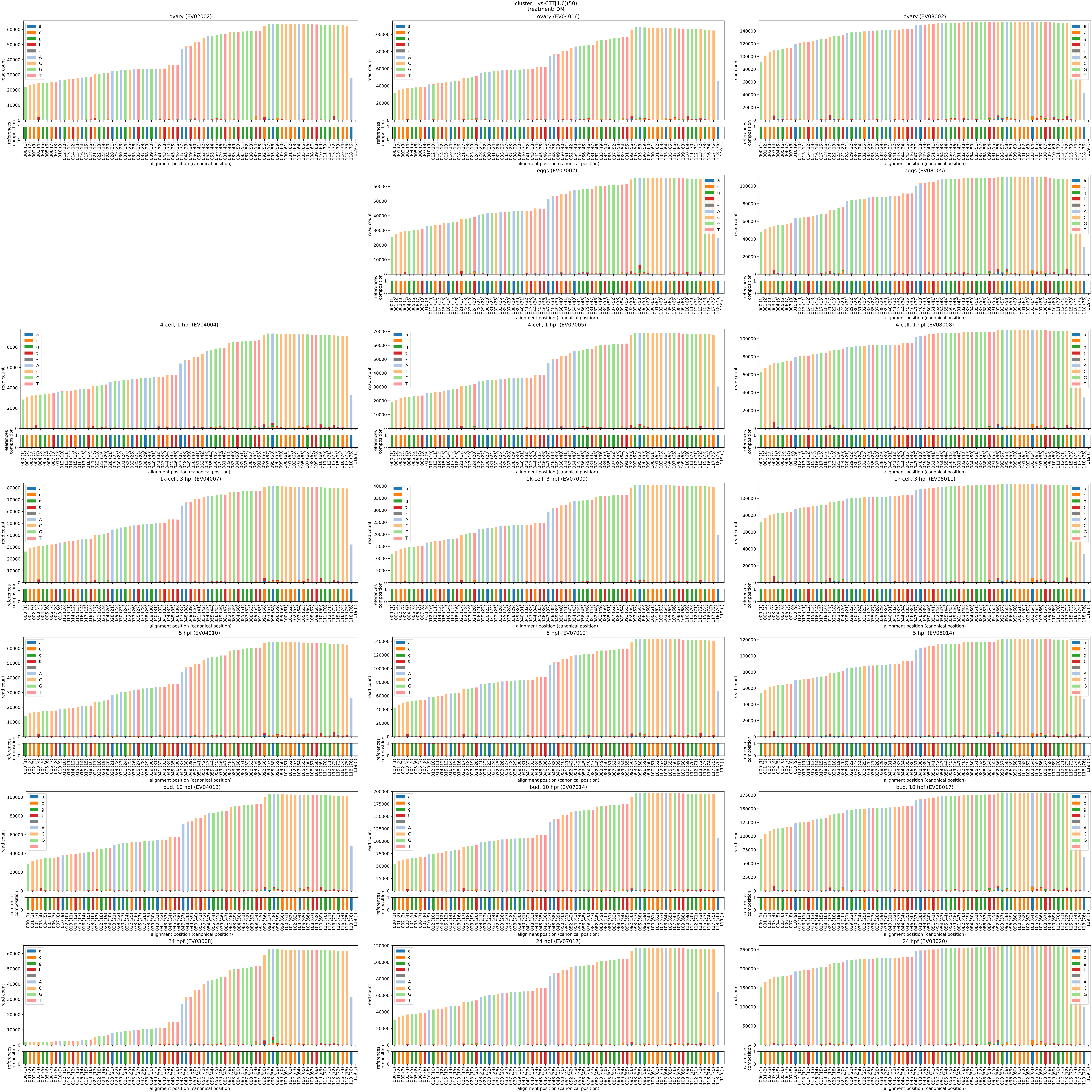
cluster: Lys-CTT[1.0](50)
treatment: DM
ovary (EV02002)
ovary (EV04016)
ovary (EV08002)
eggs (EV07002)
eggs (EV08005)
4-cell, 1 hpf (EV04004)
4-cell, 1 hpf (EV07005)
4-cell, 1 hpf (EV08008)
1k-cell, 3 hpf (EV04007)
1k-cell, 3 hpf (EV07009)
1k-cell, 3 hpf (EV08011)
5 hpf (EV04010)
5 hpf (EV07012)
5 hpf (EV08014)
bud, 10 hpf (EV04013)
bud, 10 hpf (EV07014)
bud, 10 hpf (EV08017)
24 hpf (EV03008)
24 hpf (EV07017)
24 hpf (EV08020)
read count
references composition
alignment position (canonical position)
a
c
g
t
-
A
C
G
T

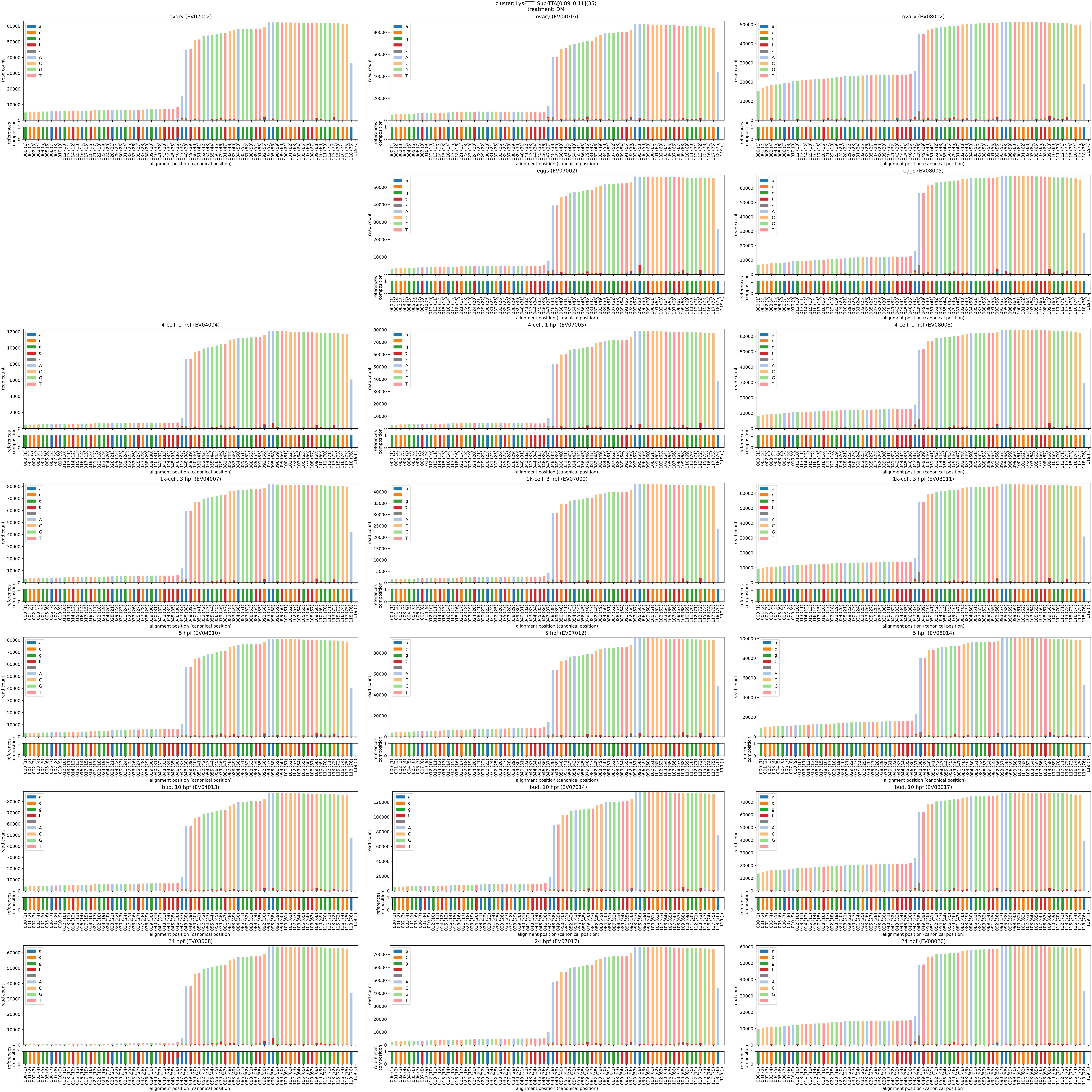
cluster: Lys-TTT_Sup-TTA[0.89_0.11](35)
treatment: DM
ovary (EV02002)
ovary (EV04016)
ovary (EV08002)
eggs (EV07002)
eggs (EV08005)
4-cell, 1 hpf (EV04004)
4-cell, 1 hpf (EV07005)
4-cell, 1 hpf (EV08008)
1k-cell, 3 hpf (EV04007)
1k-cell, 3 hpf (EV07009)
1k-cell, 3 hpf (EV08011)
5 hpf (EV04010)
5 hpf (EV07012)
5 hpf (EV08014)
bud, 10 hpf (EV04013)
bud, 10 hpf (EV07014)
bud, 10 hpf (EV08017)
24 hpf (EV03008)
24 hpf (EV07017)
24 hpf (EV08020)
read count
references composition
alignment position (canonical position)
a
c
g
t
-
A
C
G
T

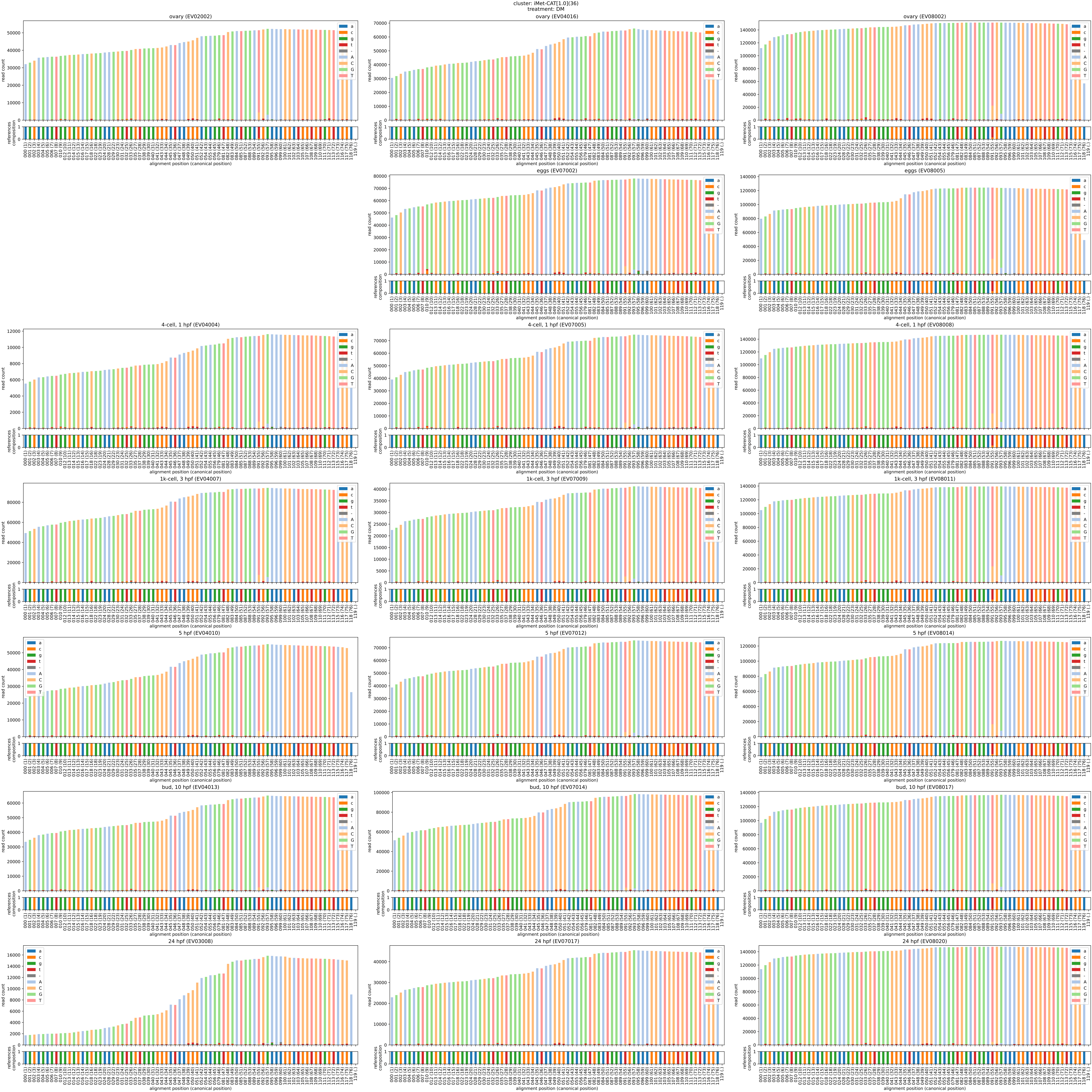
cluster: iMet-CAT[1.0](36)
treatment: DM
ovary (EV02002)
ovary (EV04016)
ovary (EV08002)
eggs (EV07002)
eggs (EV08005)
4-cell, 1 hpf (EV04004)
4-cell, 1 hpf (EV07005)
4-cell, 1 hpf (EV08008)
1k-cell, 3 hpf (EV04007)
1k-cell, 3 hpf (EV07009)
1k-cell, 3 hpf (EV08011)
5 hpf (EV04010)
5 hpf (EV07012)
5 hpf (EV08014)
bud, 10 hpf (EV04013)
bud, 10 hpf (EV07014)
bud, 10 hpf (EV08017)
24 hpf (EV03008)
24 hpf (EV07017)
24 hpf (EV08020)
read count
references composition
alignment position (canonical position)
a
c
g
t
-
A
C
G
T

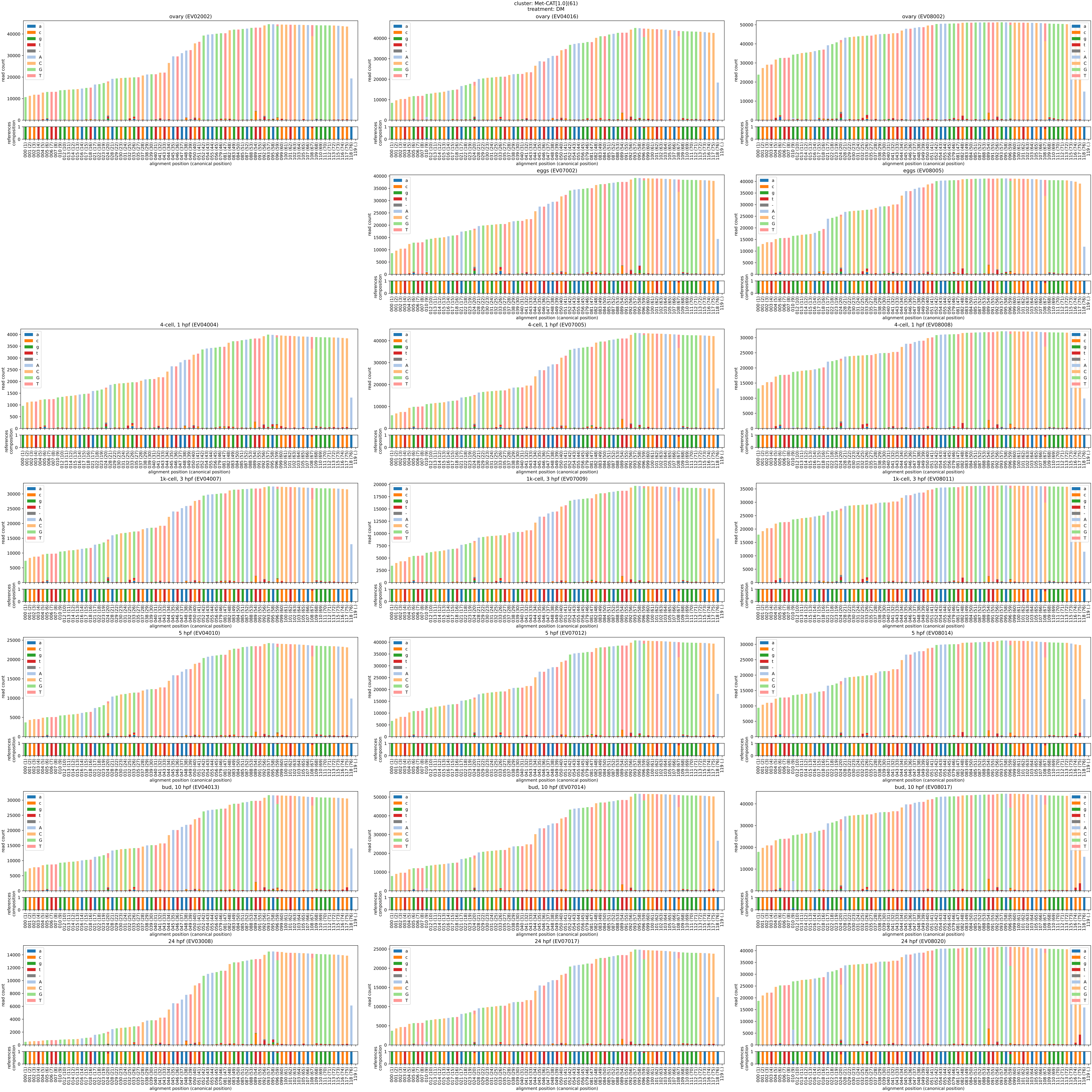
cluster: Met-CAT[1.0](61)
treatment: DM
ovary (EV02002)
ovary (EV04016)
ovary (EV08002)
eggs (EV07002)
eggs (EV08005)
4-cell, 1 hpf (EV04004)
4-cell, 1 hpf (EV07005)
4-cell, 1 hpf (EV08008)
1k-cell, 3 hpf (EV04007)
1k-cell, 3 hpf (EV07009)
1k-cell, 3 hpf (EV08011)
5 hpf (EV04010)
5 hpf (EV07012)
5 hpf (EV08014)
bud, 10 hpf (EV04013)
bud, 10 hpf (EV07014)
bud, 10 hpf (EV08017)
24 hpf (EV03008)
24 hpf (EV07017)
24 hpf (EV08020)
read count
references composition
alignment position (canonical position)
a
c
g
t
-
A
C
G
T

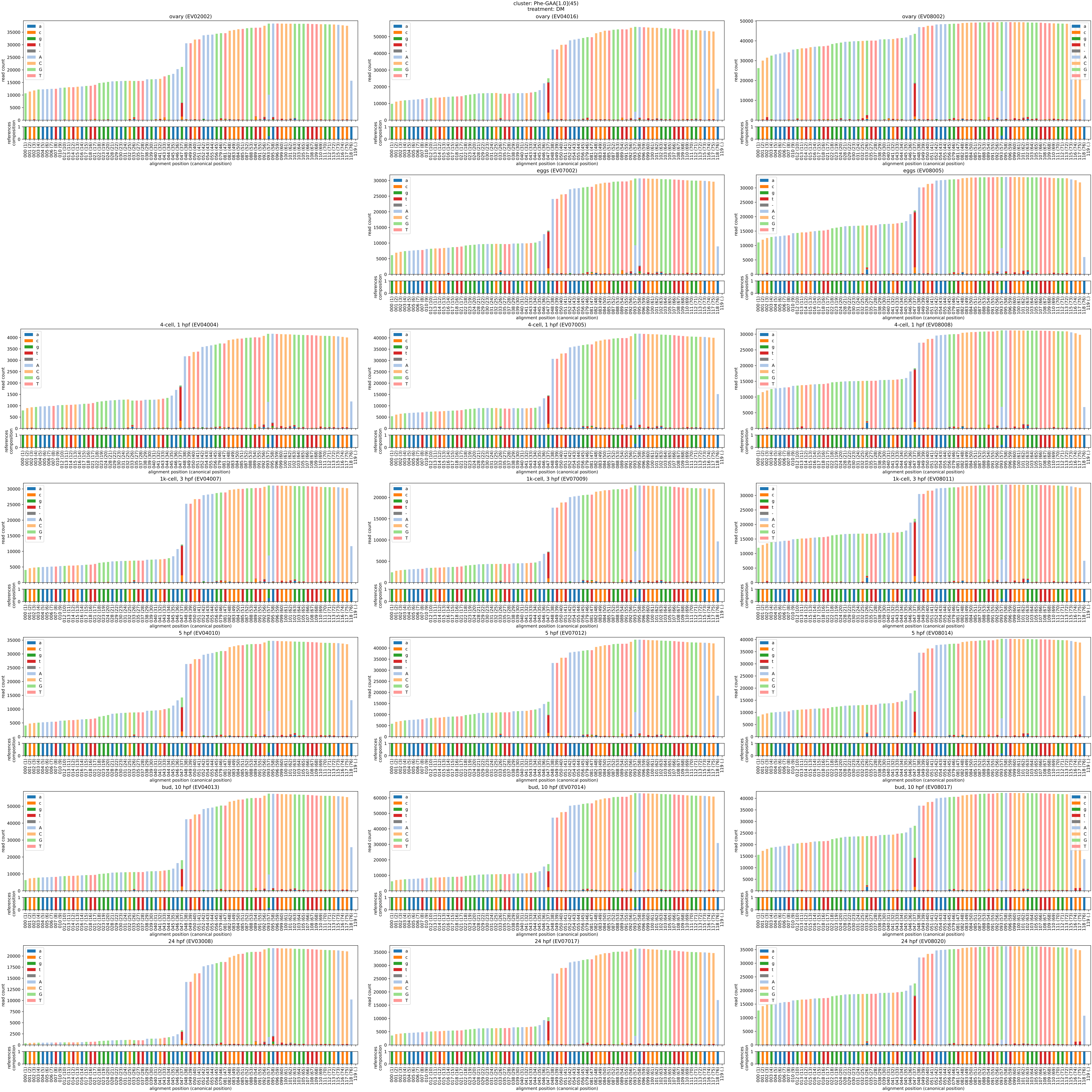

cluster: Phe-GAA[1.0](45)
treatment: DM
ovary (EV02002)
ovary (EV04016)
ovary (EV08002)
eggs (EV07002)
eggs (EV08005)
4-cell, 1 hpf (EV04004)
4-cell, 1 hpf (EV07005)
4-cell, 1 hpf (EV08008)
1k-cell, 3 hpf (EV04007)
1k-cell, 3 hpf (EV07009)
1k-cell, 3 hpf (EV08011)
5 hpf (EV04010)
5 hpf (EV07012)
5 hpf (EV08014)
bud, 10 hpf (EV04013)
bud, 10 hpf (EV07014)
bud, 10 hpf (EV08017)
24 hpf (EV03008)
24 hpf (EV07017)
24 hpf (EV08020)
read count
references composition
alignment position (canonical position)
a
c
g
t
-
A
C
G
T

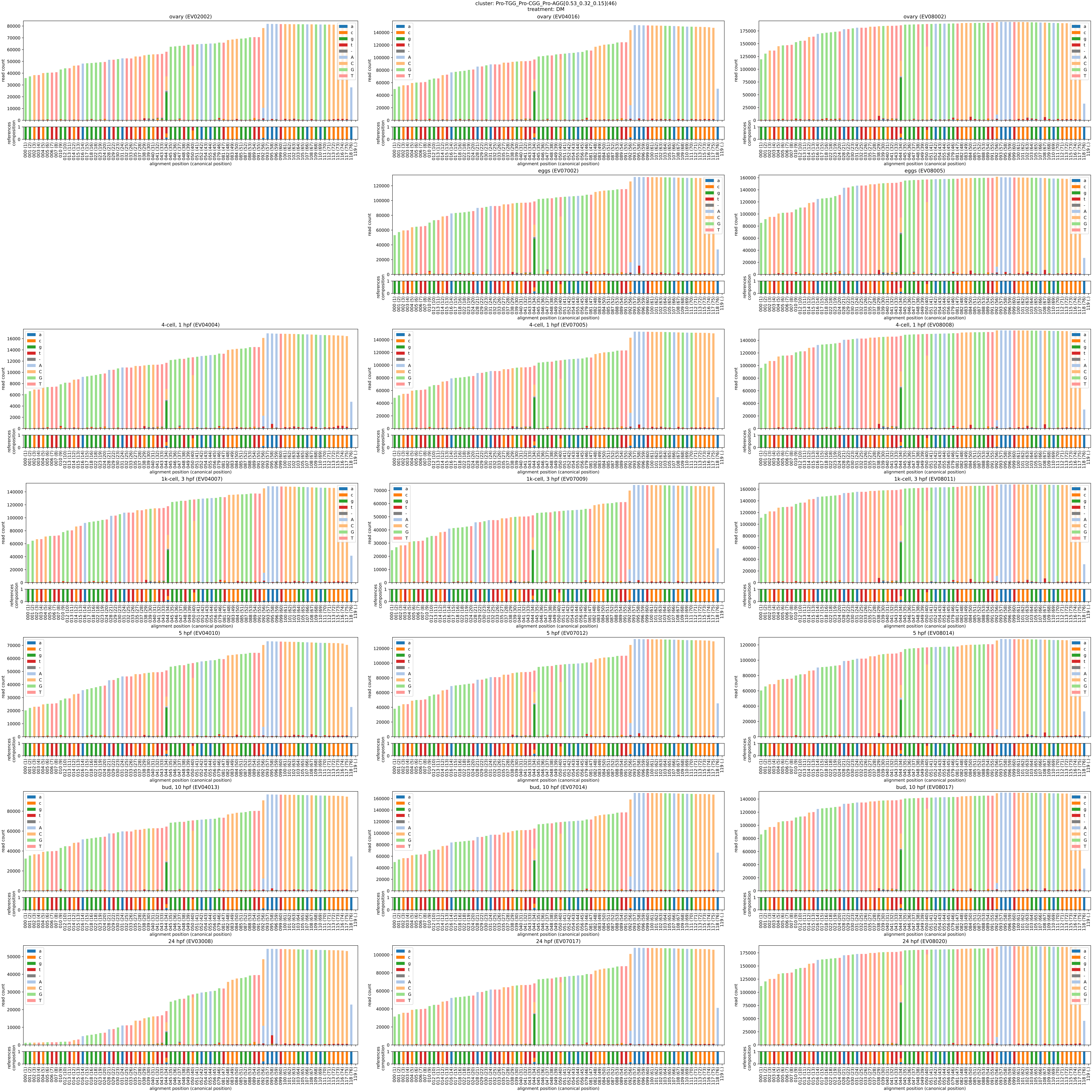
cluster: Pro-TGG_Pro-CGG_Pro-AGG[0.53_0.32_0.15](46)
treatment: DM
ovary (EV02002)
ovary (EV04016)
ovary (EV08002)
eggs (EV07002)
eggs (EV08005)
4-cell, 1 hpf (EV04004)
4-cell, 1 hpf (EV07005)
4-cell, 1 hpf (EV08008)
1k-cell, 3 hpf (EV04007)
1k-cell, 3 hpf (EV07009)
1k-cell, 3 hpf (EV08011)
5 hpf (EV04010)
5 hpf (EV07012)
5 hpf (EV08014)
bud, 10 hpf (EV04013)
bud, 10 hpf (EV07014)
bud, 10 hpf (EV08017)
24 hpf (EV03008)
24 hpf (EV07017)
24 hpf (EV08020)
read count
references composition
alignment position (canonical position)
a
c
g
t
-
A
C
G
T

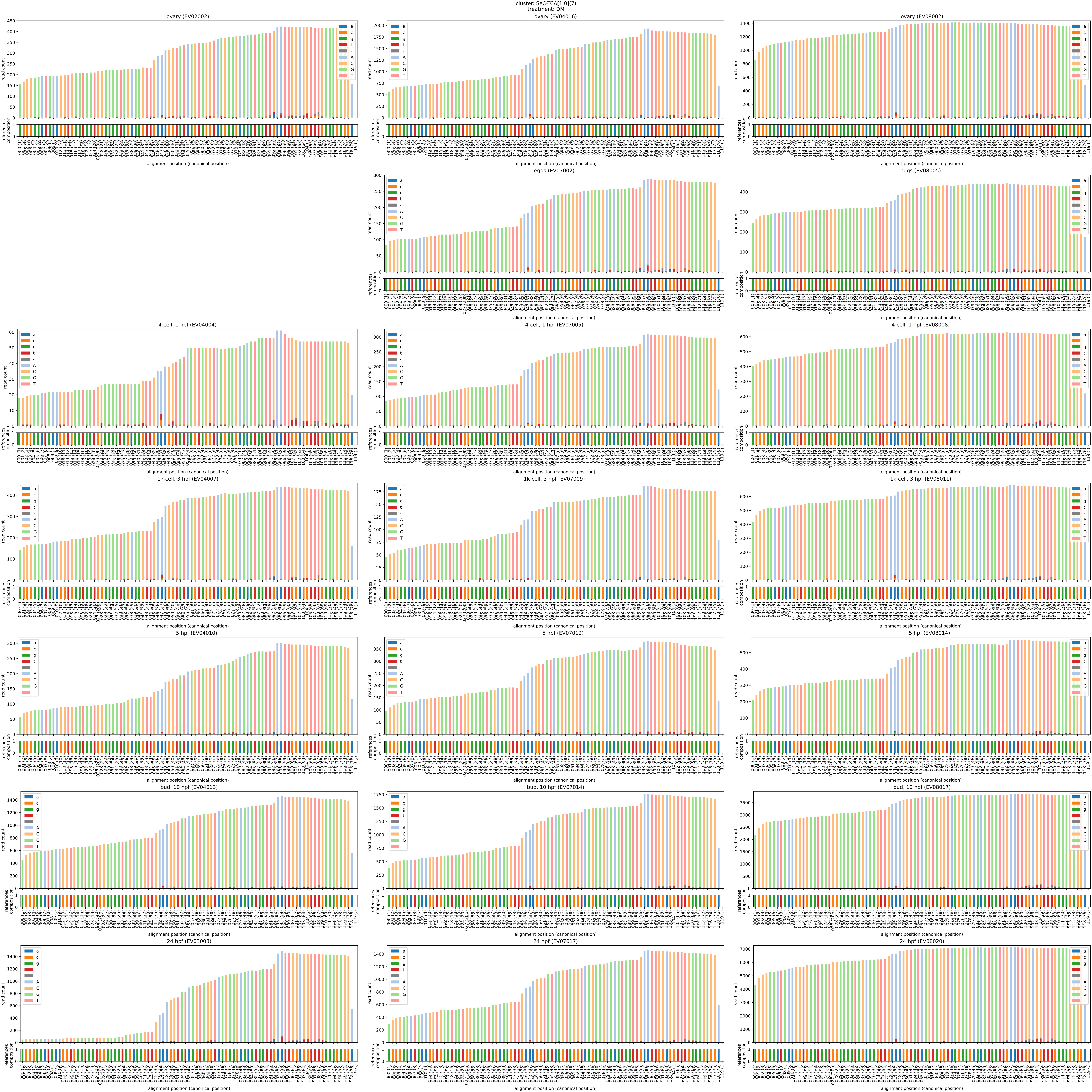
cluster: SeC-TCA[1.0](7)
treatment: DM
ovary (EV02002)
ovary (EV04016)
ovary (EV08002)
eggs (EV07002)
eggs (EV08005)
4-cell, 1 hpf (EV04004)
4-cell, 1 hpf (EV07005)
4-cell, 1 hpf (EV08008)
1k-cell, 3 hpf (EV04007)
1k-cell, 3 hpf (EV07009)
1k-cell, 3 hpf (EV08011)
5 hpf (EV04010)
5 hpf (EV07012)
5 hpf (EV08014)
bud, 10 hpf (EV04013)
bud, 10 hpf (EV07014)
bud, 10 hpf (EV08017)
24 hpf (EV03008)
24 hpf (EV07017)
24 hpf (EV08020)
read count
references composition
alignment position (canonical position)

cluster: Ser-CGA[1.0](17)
treatment: DM

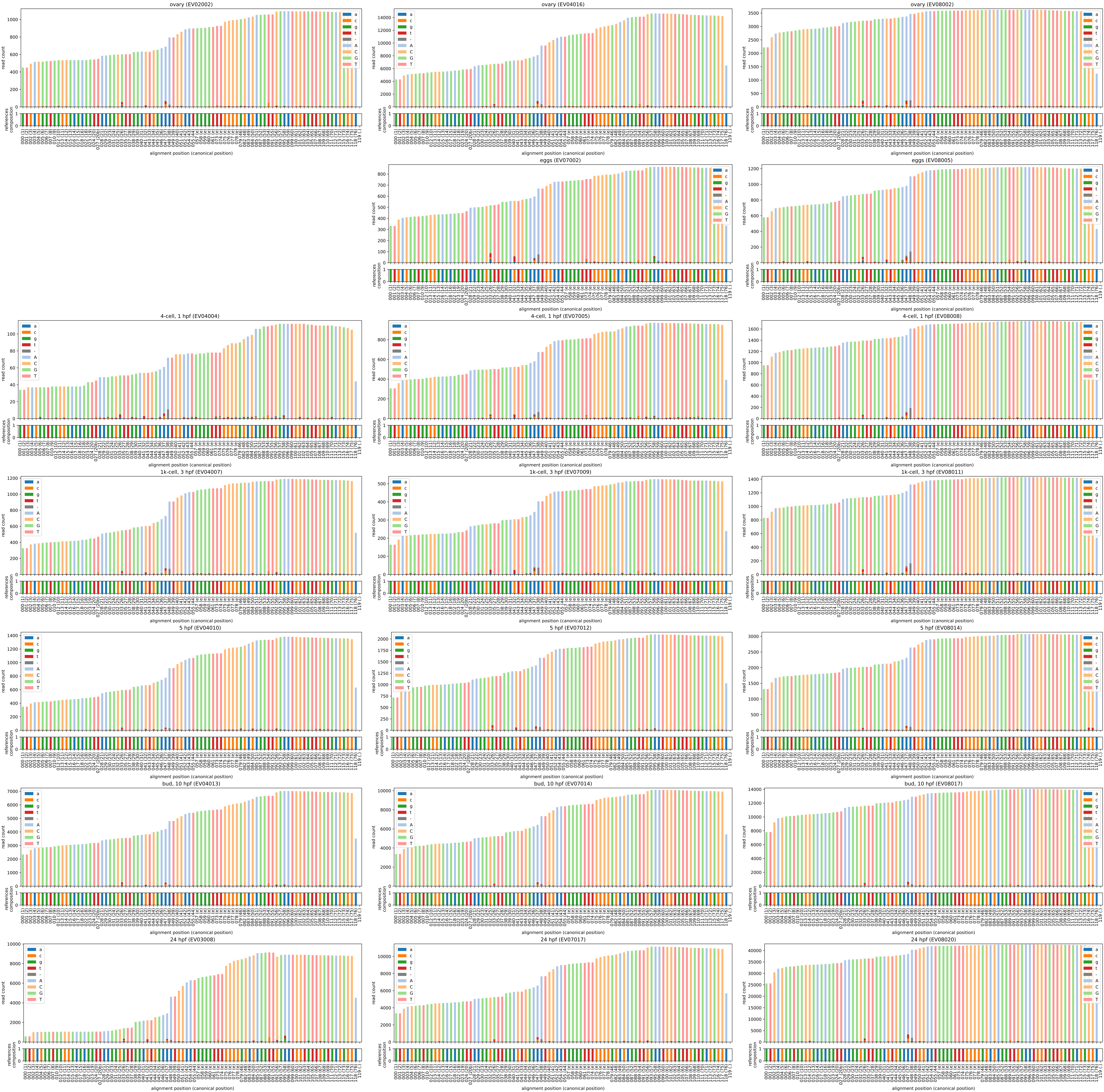

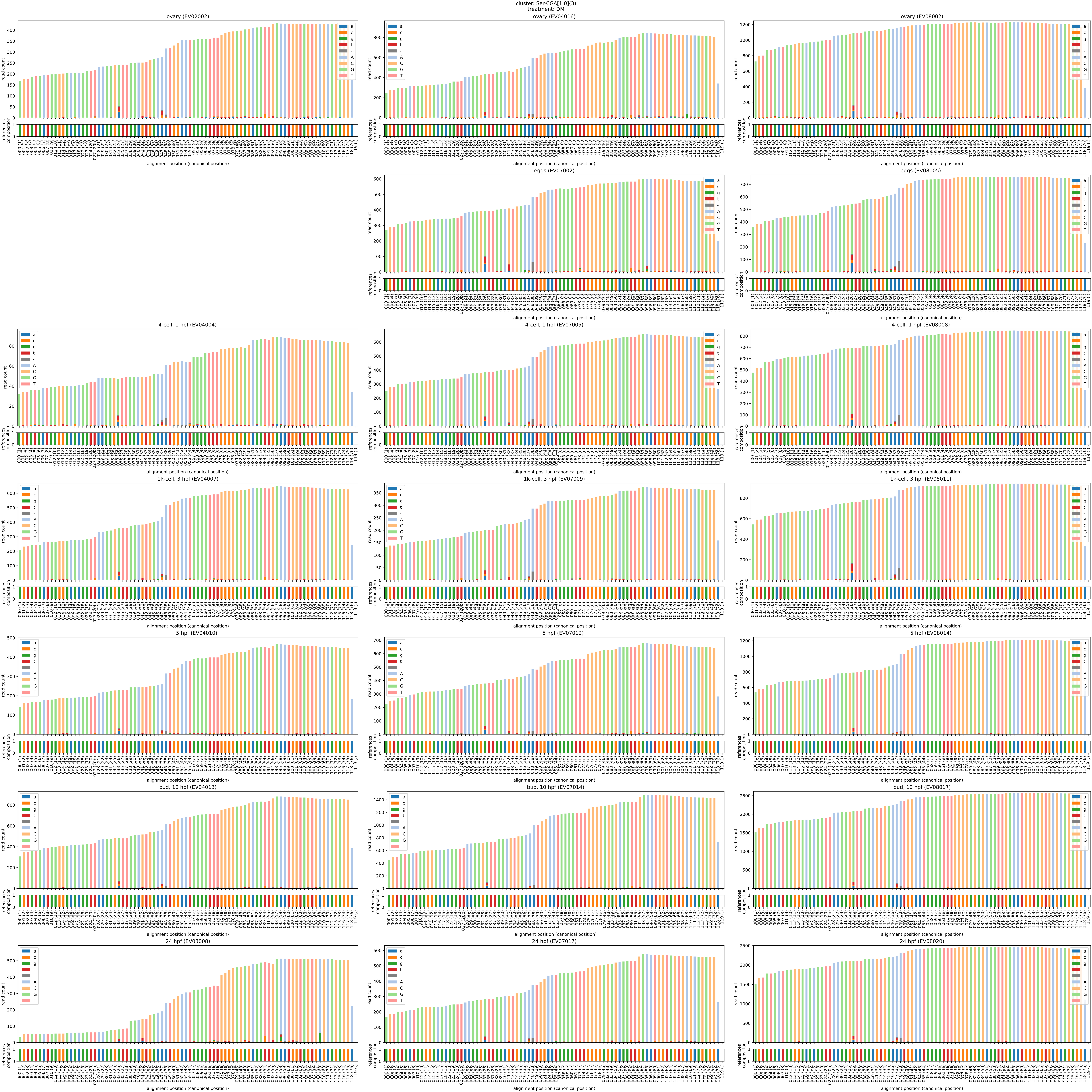
cluster: Ser-CGA[1.0](3)
treatment: DM
ovary (EV02002)
ovary (EV04016)
ovary (EV08002)
eggs (EV07002)
eggs (EV08005)
4-cell, 1 hpf (EV04004)
4-cell, 1 hpf (EV07005)
4-cell, 1 hpf (EV08008)
1k-cell, 3 hpf (EV04007)
1k-cell, 3 hpf (EV07009)
1k-cell, 3 hpf (EV08011)
5 hpf (EV04010)
5 hpf (EV07012)
5 hpf (EV08014)
bud, 10 hpf (EV04013)
bud, 10 hpf (EV07014)
bud, 10 hpf (EV08017)
24 hpf (EV03008)
24 hpf (EV07017)
24 hpf (EV08020)
read count
references composition
alignment position (canonical position)
a
c
g
t
-
A
C
G
T

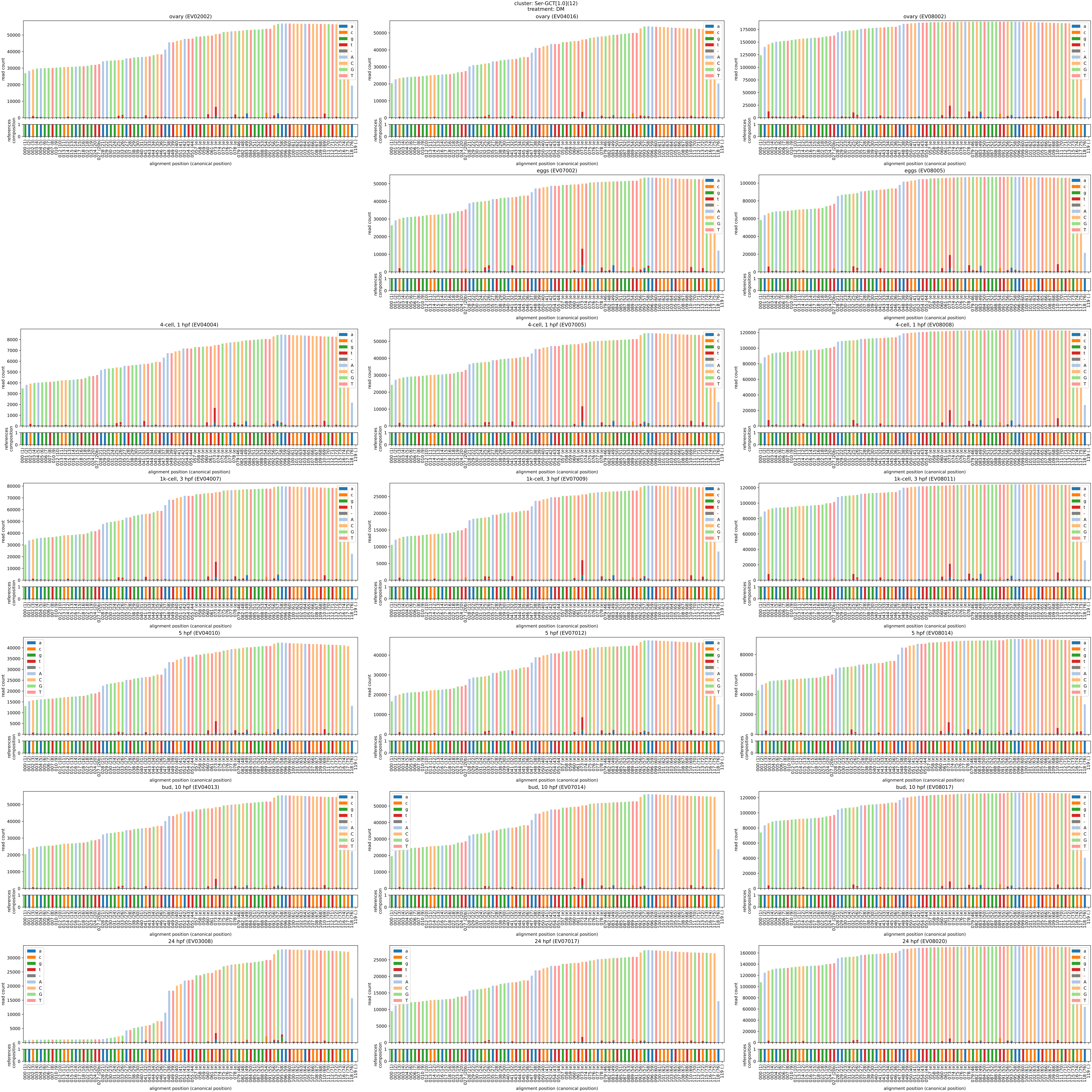
cluster: Ser-GCT[1.0](12)
treatment: DM
ovary (EV02002)
ovary (EV04016)
ovary (EV08002)
eggs (EV07002)
eggs (EV08005)
4-cell, 1 hpf (EV04004)
4-cell, 1 hpf (EV07005)
4-cell, 1 hpf (EV08008)
1k-cell, 3 hpf (EV04007)
1k-cell, 3 hpf (EV07009)
1k-cell, 3 hpf (EV08011)
5 hpf (EV04010)
5 hpf (EV07012)
5 hpf (EV08014)
bud, 10 hpf (EV04013)
bud, 10 hpf (EV07014)
bud, 10 hpf (EV08017)
24 hpf (EV03008)
24 hpf (EV07017)
24 hpf (EV08020)
read count
references composition
alignment position (canonical position)
a
c
g
t
-
A
C
G
T

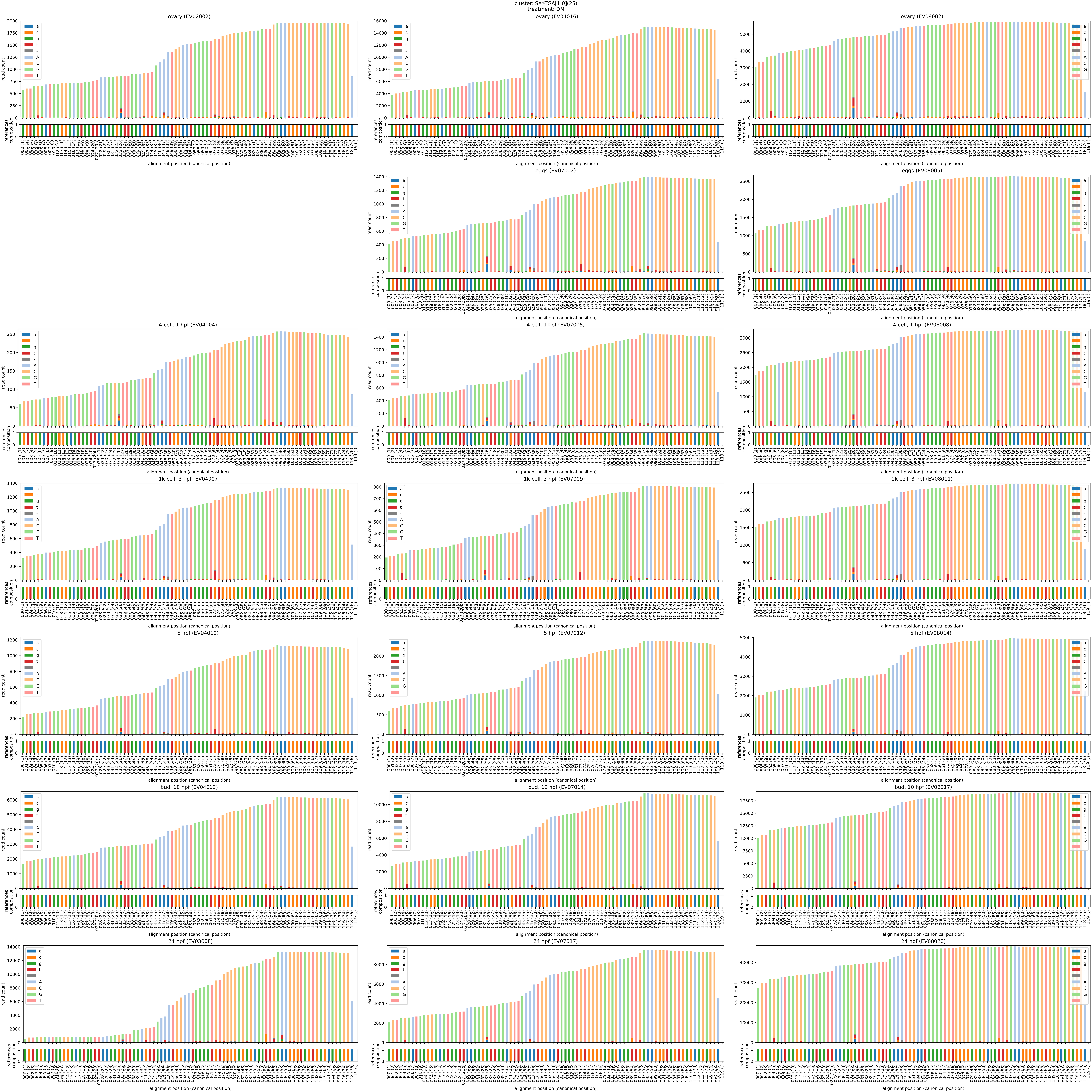
cluster: Ser-TGA[1.0](25)
treatment: DM
ovary (EV02002)
ovary (EV04016)
ovary (EV08002)
eggs (EV07002)
eggs (EV08005)
4-cell, 1 hpf (EV04004)
4-cell, 1 hpf (EV07005)
4-cell, 1 hpf (EV08008)
1k-cell, 3 hpf (EV04007)
1k-cell, 3 hpf (EV07009)
1k-cell, 3 hpf (EV08011)
5 hpf (EV04010)
5 hpf (EV07012)
5 hpf (EV08014)
bud, 10 hpf (EV04013)
bud, 10 hpf (EV07014)
bud, 10 hpf (EV08017)
24 hpf (EV03008)
24 hpf (EV07017)
24 hpf (EV08020)
read count
references composition
alignment position (canonical position)
a
c
g
t
-
A
C
G
T

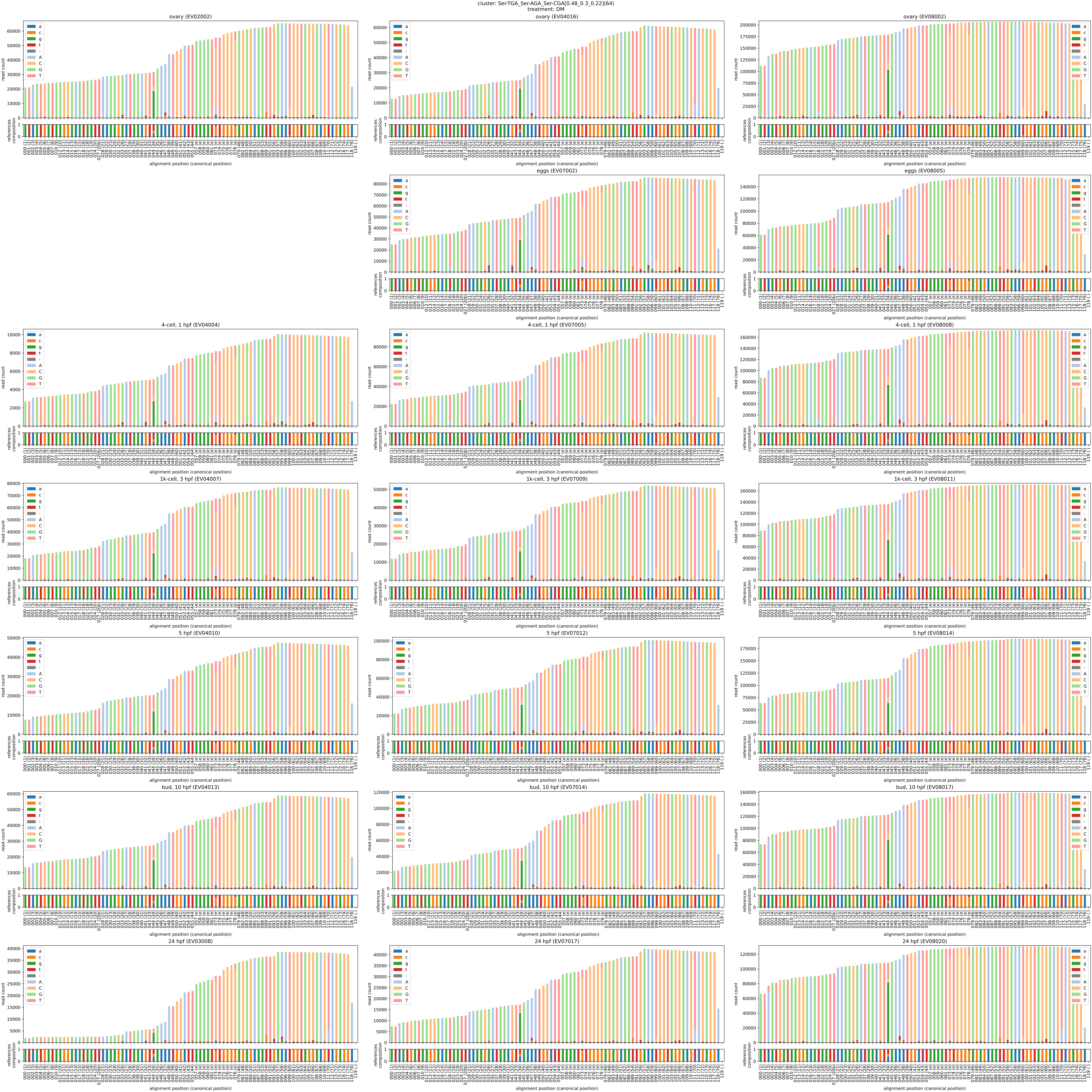
cluster: Ser-TGA_Ser-AGA_Ser-CGA[0.48_0.3_0.22](64)
treatment: DM
ovary (EV02002)
ovary (EV04016)
ovary (EV08002)
eggs (EV07002)
eggs (EV08005)
4-cell, 1 hpf (EV04004)
4-cell, 1 hpf (EV07005)
4-cell, 1 hpf (EV08008)
1k-cell, 3 hpf (EV04007)
1k-cell, 3 hpf (EV07009)
1k-cell, 3 hpf (EV08011)
5 hpf (EV04010)
5 hpf (EV07012)
5 hpf (EV08014)
bud, 10 hpf (EV04013)
bud, 10 hpf (EV07014)
bud, 10 hpf (EV08017)
24 hpf (EV03008)
24 hpf (EV07017)
24 hpf (EV08020)
read count
references composition
alignment position (canonical position)
a
c
g
t
-
A
C
G
T

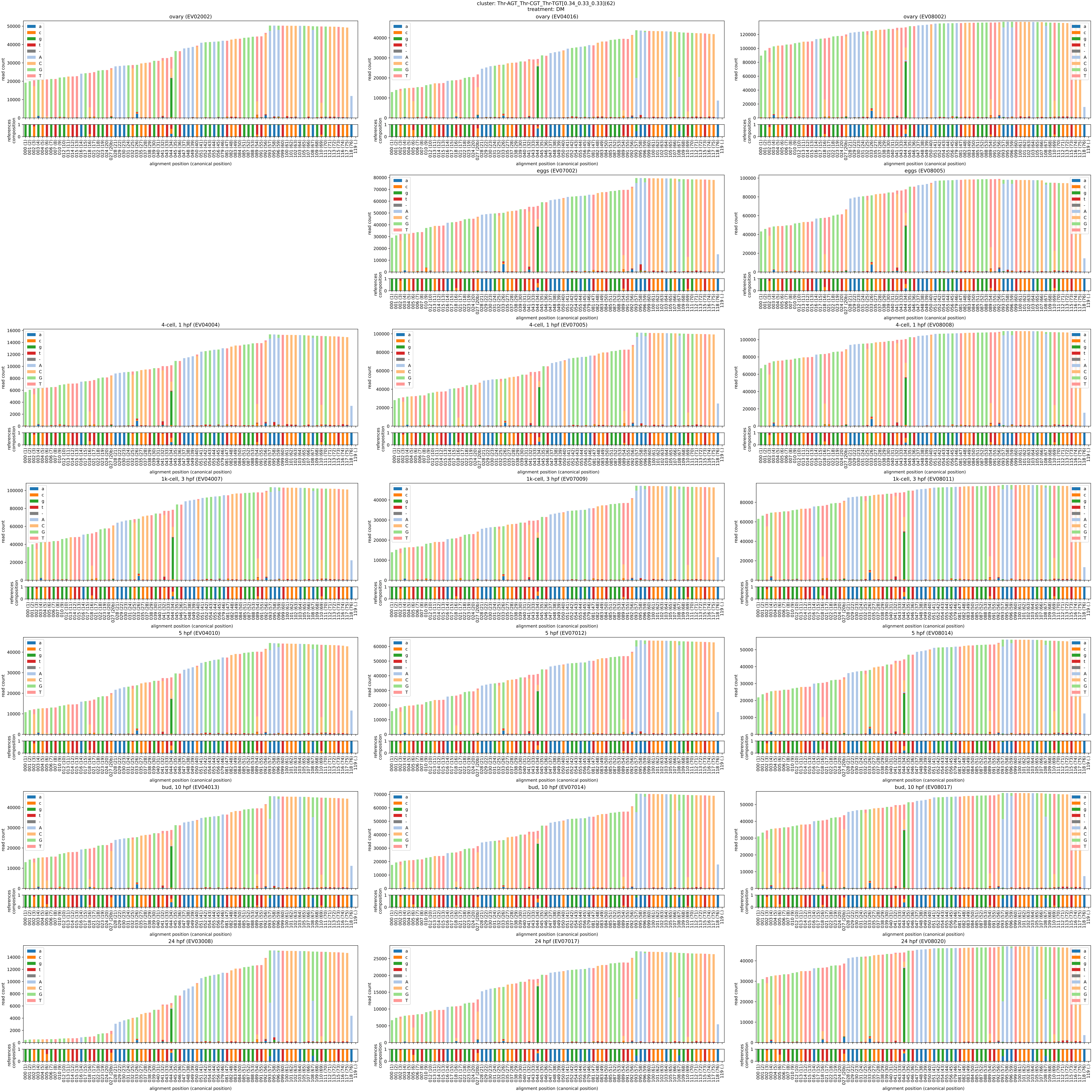
cluster: Thr-AGT_Thr-CGT_Thr-TGT[0.34_0.33_0.33](62)
treatment: DM
ovary (EV02002)
ovary (EV04016)
ovary (EV08002)
eggs (EV07002)
eggs (EV08005)
4-cell, 1 hpf (EV04004)
4-cell, 1 hpf (EV07005)
4-cell, 1 hpf (EV08008)
1k-cell, 3 hpf (EV04007)
1k-cell, 3 hpf (EV07009)
1k-cell, 3 hpf (EV08011)
5 hpf (EV04010)
5 hpf (EV07012)
5 hpf (EV08014)
bud, 10 hpf (EV04013)
bud, 10 hpf (EV07014)
bud, 10 hpf (EV08017)
24 hpf (EV03008)
24 hpf (EV07017)
24 hpf (EV08020)
read count
references composition
alignment position (canonical position)
a
c
g
t
-
A
C
G
T

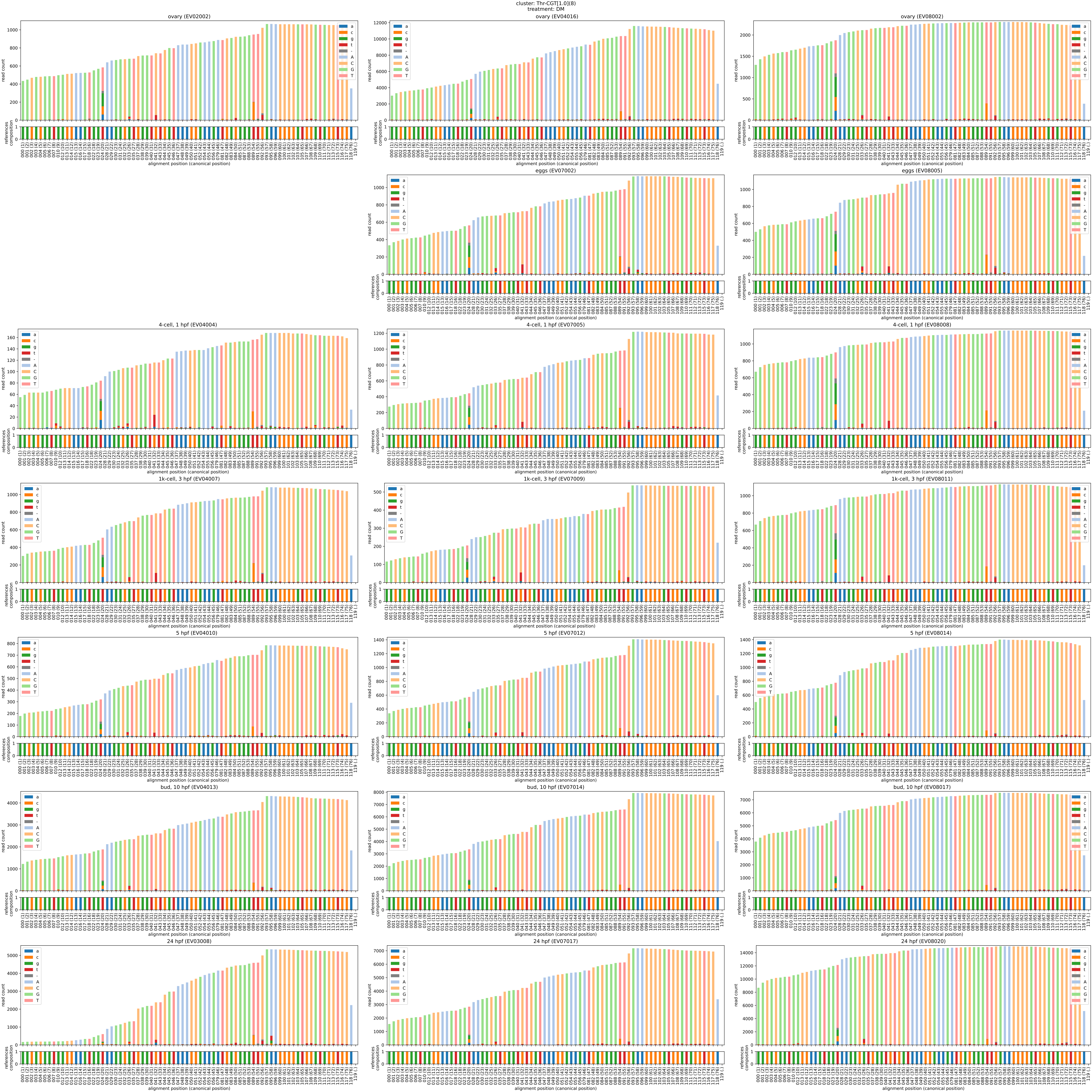

cluster: Thr-CGT[1.0](8)
treatment: DM
ovary (EV02002)
ovary (EV04016)
ovary (EV08002)
eggs (EV07002)
eggs (EV08005)
4-cell, 1 hpf (EV04004)
4-cell, 1 hpf (EV07005)
4-cell, 1 hpf (EV08008)
1k-cell, 3 hpf (EV04007)
1k-cell, 3 hpf (EV07009)
1k-cell, 3 hpf (EV08011)
5 hpf (EV04010)
5 hpf (EV07012)
5 hpf (EV08014)
bud, 10 hpf (EV04013)
bud, 10 hpf (EV07014)
bud, 10 hpf (EV08017)
24 hpf (EV03008)
24 hpf (EV07017)
24 hpf (EV08020)
read count
references composition
alignment position (canonical position)
a
c
g
t
-
A
C
G
T

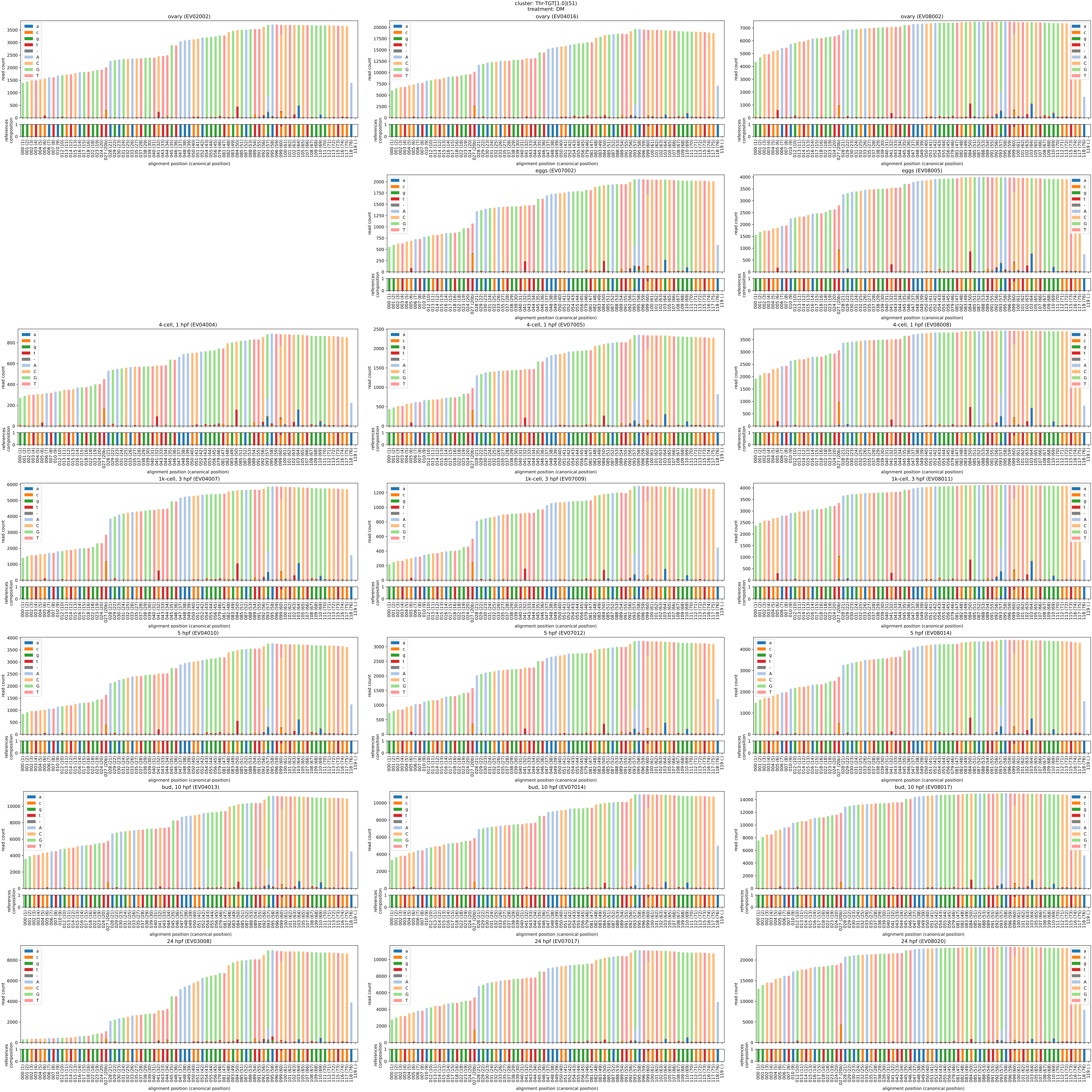
cluster: Thr-TGT[1.0](51)
treatment: DM
ovary (EV02002)
ovary (EV04016)
ovary (EV08002)
eggs (EV07002)
eggs (EV08005)
4-cell, 1 hpf (EV04004)
4-cell, 1 hpf (EV07005)
4-cell, 1 hpf (EV08008)
1k-cell, 3 hpf (EV04007)
1k-cell, 3 hpf (EV07009)
1k-cell, 3 hpf (EV08011)
5 hpf (EV04010)
5 hpf (EV07012)
5 hpf (EV08014)
bud, 10 hpf (EV04013)
bud, 10 hpf (EV07014)
bud, 10 hpf (EV08017)
24 hpf (EV03008)
24 hpf (EV07017)
24 hpf (EV08020)
read count
references composition
alignment position (canonical position)
a
c
g
t
-
A
C
G
T

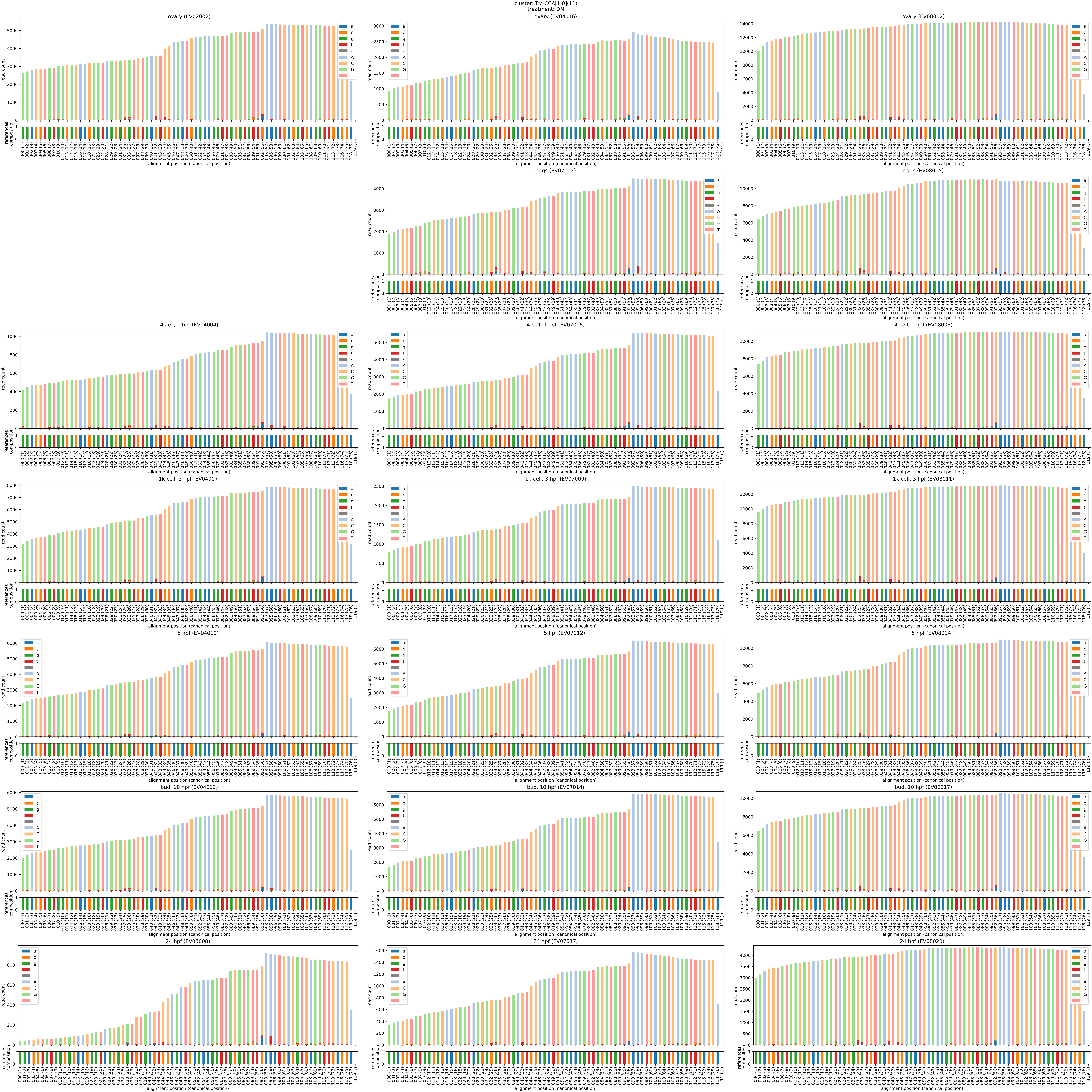
cluster: Trp-CCA[1.0](11)
treatment: DM
ovary (EV02002)
ovary (EV04016)
ovary (EV08002)
eggs (EV07002)
eggs (EV08005)
4-cell, 1 hpf (EV04004)
4-cell, 1 hpf (EV07005)
4-cell, 1 hpf (EV08008)
1k-cell, 3 hpf (EV04007)
1k-cell, 3 hpf (EV07009)
1k-cell, 3 hpf (EV08011)
5 hpf (EV04010)
5 hpf (EV07012)
5 hpf (EV08014)
bud, 10 hpf (EV04013)
bud, 10 hpf (EV07014)
bud, 10 hpf (EV08017)
24 hpf (EV03008)
24 hpf (EV07017)
24 hpf (EV08020)
read count
references composition
alignment position (canonical position)
a
c
g
t
-
A
C
G
T

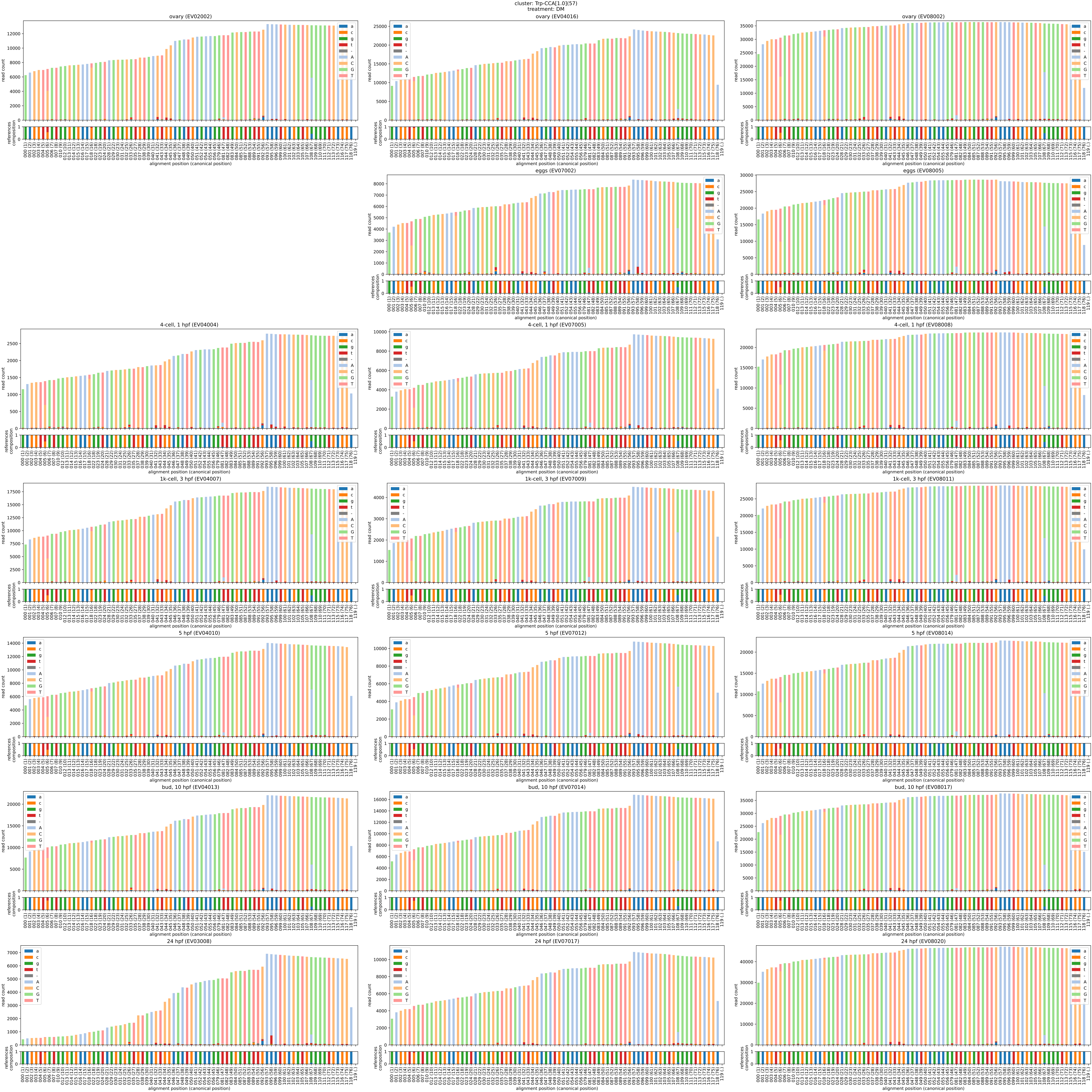
cluster: Trp-CCA[1.0](57)
treatment: DM
ovary (EV02002)
ovary (EV04016)
ovary (EV08002)
eggs (EV07002)
eggs (EV08005)
4-cell, 1 hpf (EV04004)
4-cell, 1 hpf (EV07005)
4-cell, 1 hpf (EV08008)
1k-cell, 3 hpf (EV04007)
1k-cell, 3 hpf (EV07009)
1k-cell, 3 hpf (EV08011)
5 hpf (EV04010)
5 hpf (EV07012)
5 hpf (EV08014)
bud, 10 hpf (EV04013)
bud, 10 hpf (EV07014)
bud, 10 hpf (EV08017)
24 hpf (EV03008)
24 hpf (EV07017)
24 hpf (EV08020)
read count
references composition
alignment position (canonical position)
a
c
g
t
-
A
C
G
T

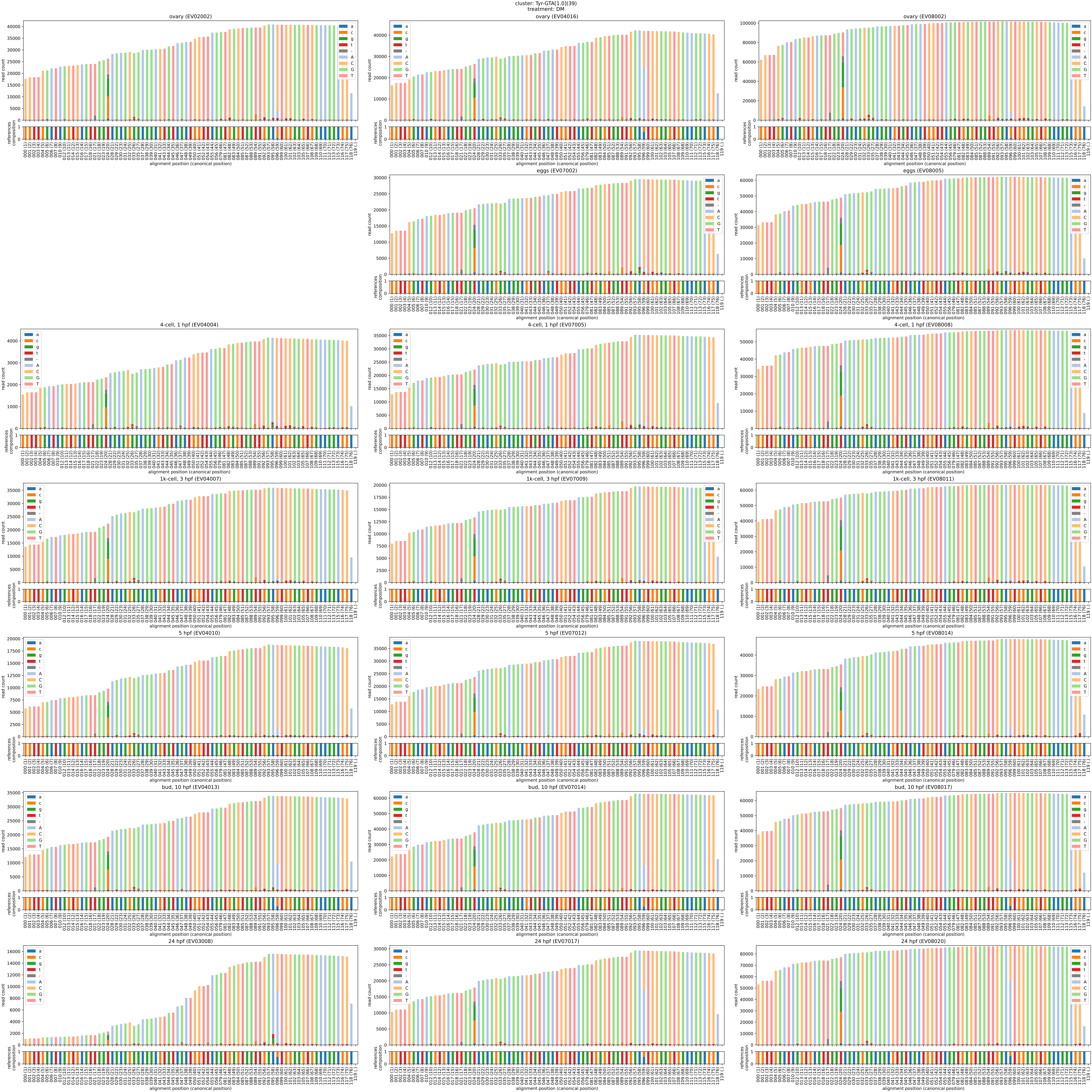
cluster: Tyr-GTA[1.0](39)
treatment: DM
ovary (EV02002)
ovary (EV04016)
ovary (EV08002)
eggs (EV07002)
eggs (EV08005)
4-cell, 1 hpf (EV04004)
4-cell, 1 hpf (EV07005)
4-cell, 1 hpf (EV08008)
1k-cell, 3 hpf (EV04007)
1k-cell, 3 hpf (EV07009)
1k-cell, 3 hpf (EV08011)
5 hpf (EV04010)
5 hpf (EV07012)
5 hpf (EV08014)
bud, 10 hpf (EV04013)
bud, 10 hpf (EV07014)
bud, 10 hpf (EV08017)
24 hpf (EV03008)
24 hpf (EV07017)
24 hpf (EV08020)
read count
references composition
alignment position (canonical position)
a
c
g
t
-
A
C
G
T

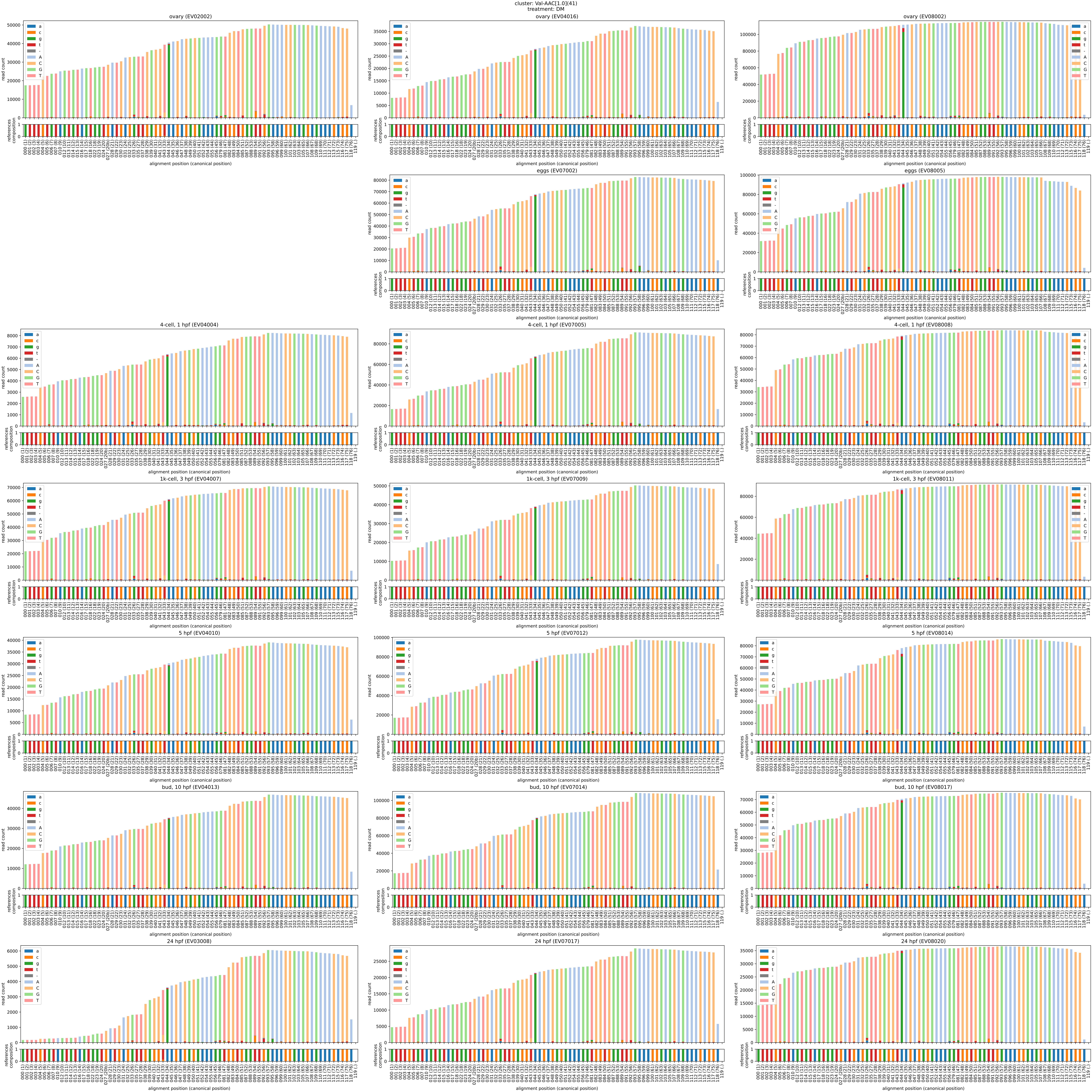
cluster: Val-AAC[1.0](41)
treatment: DM
ovary (EV02002)
ovary (EV04016)
ovary (EV08002)
eggs (EV07002)
eggs (EV08005)
4-cell, 1 hpf (EV04004)
4-cell, 1 hpf (EV07005)
4-cell, 1 hpf (EV08008)
1k-cell, 3 hpf (EV04007)
1k-cell, 3 hpf (EV07009)
1k-cell, 3 hpf (EV08011)
5 hpf (EV04010)
5 hpf (EV07012)
5 hpf (EV08014)
bud, 10 hpf (EV04013)
bud, 10 hpf (EV07014)
bud, 10 hpf (EV08017)
24 hpf (EV03008)
24 hpf (EV07017)
24 hpf (EV08020)
read count
references composition
alignment position (canonical position)
a
c
g
t
-
A
C
G
T

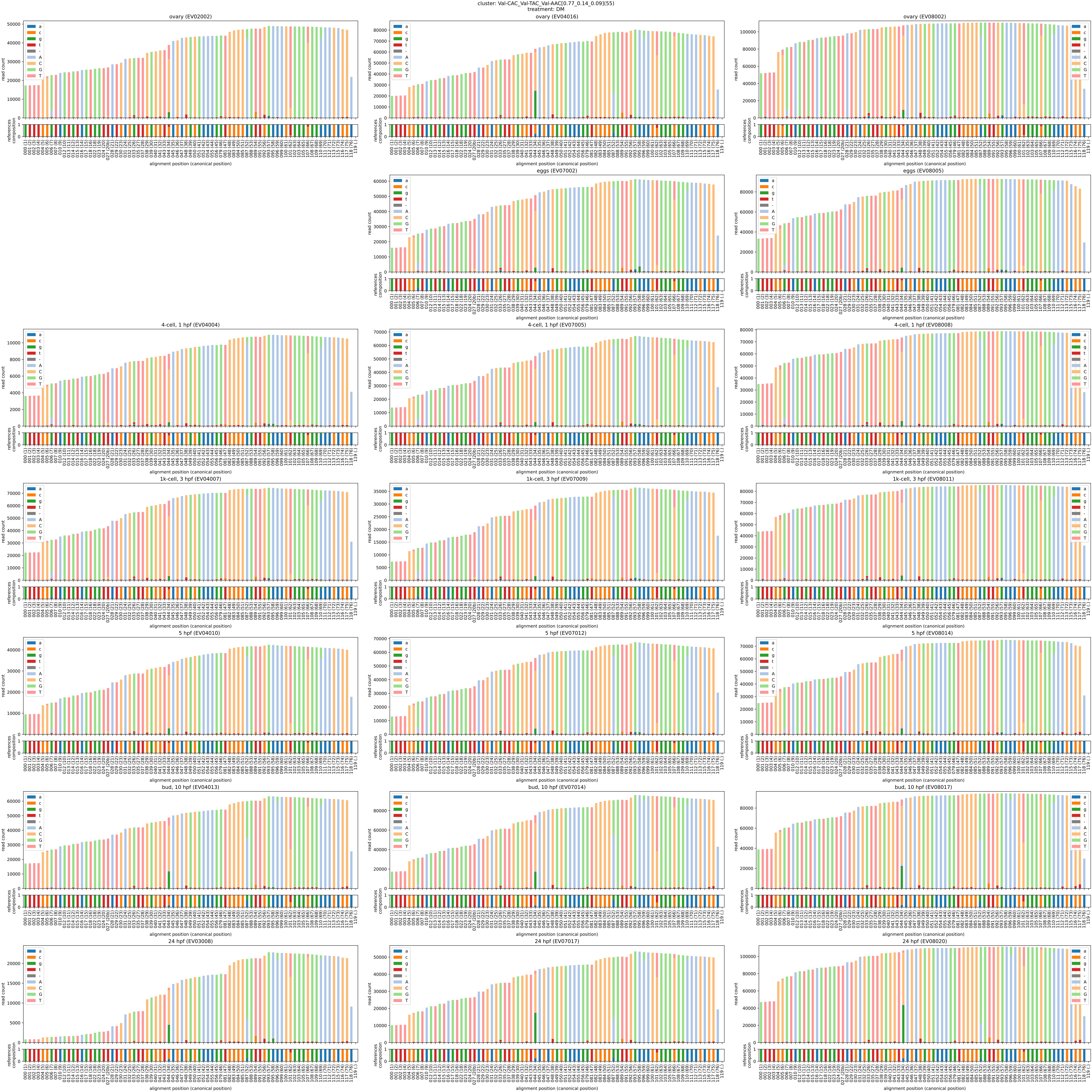
cluster: Val-CAC_Val-TAC_Val-AAC[0.77_0.14_0.09](55)
treatment: DM
ovary (EV02002)
ovary (EV04016)
ovary (EV08002)
eggs (EV07002)
eggs (EV08005)
4-cell, 1 hpf (EV04004)
4-cell, 1 hpf (EV07005)
4-cell, 1 hpf (EV08008)
1k-cell, 3 hpf (EV04007)
1k-cell, 3 hpf (EV07009)
1k-cell, 3 hpf (EV08011)
5 hpf (EV04010)
5 hpf (EV07012)
5 hpf (EV08014)
bud, 10 hpf (EV04013)
bud, 10 hpf (EV07014)
bud, 10 hpf (EV08017)
24 hpf (EV03008)
24 hpf (EV07017)
24 hpf (EV08020)
a
c
g
t
-
A
C
G
T
read count
references composition
alignment position (canonical position)

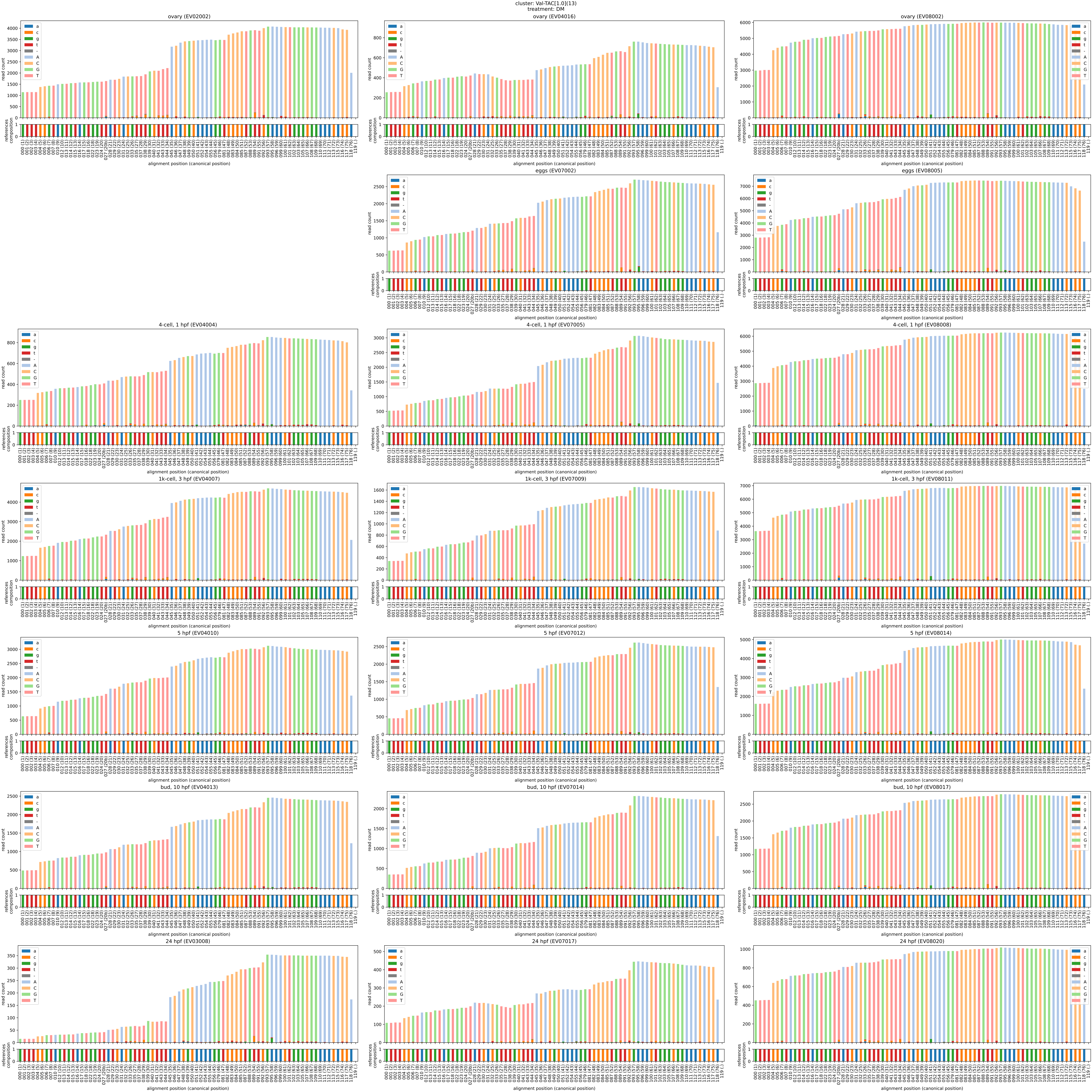

cluster: Val-TAC[1.0](13)
treatment: DM
ovary (EV02002)
ovary (EV04016)
ovary (EV08002)
eggs (EV07002)
eggs (EV08005)
4-cell, 1 hpf (EV04004)
4-cell, 1 hpf (EV07005)
4-cell, 1 hpf (EV08008)
1k-cell, 3 hpf (EV04007)
1k-cell, 3 hpf (EV07009)
1k-cell, 3 hpf (EV08011)
5 hpf (EV04010)
5 hpf (EV07012)
5 hpf (EV08014)
bud, 10 hpf (EV04013)
bud, 10 hpf (EV07014)
bud, 10 hpf (EV08017)
24 hpf (EV03008)
24 hpf (EV07017)
24 hpf (EV08020)
read count
references composition
alignment position (canonical position)
a
c
g
t
-
A
C
G
T

cluster: Val-TAC[1.0](38)
treatment: DM

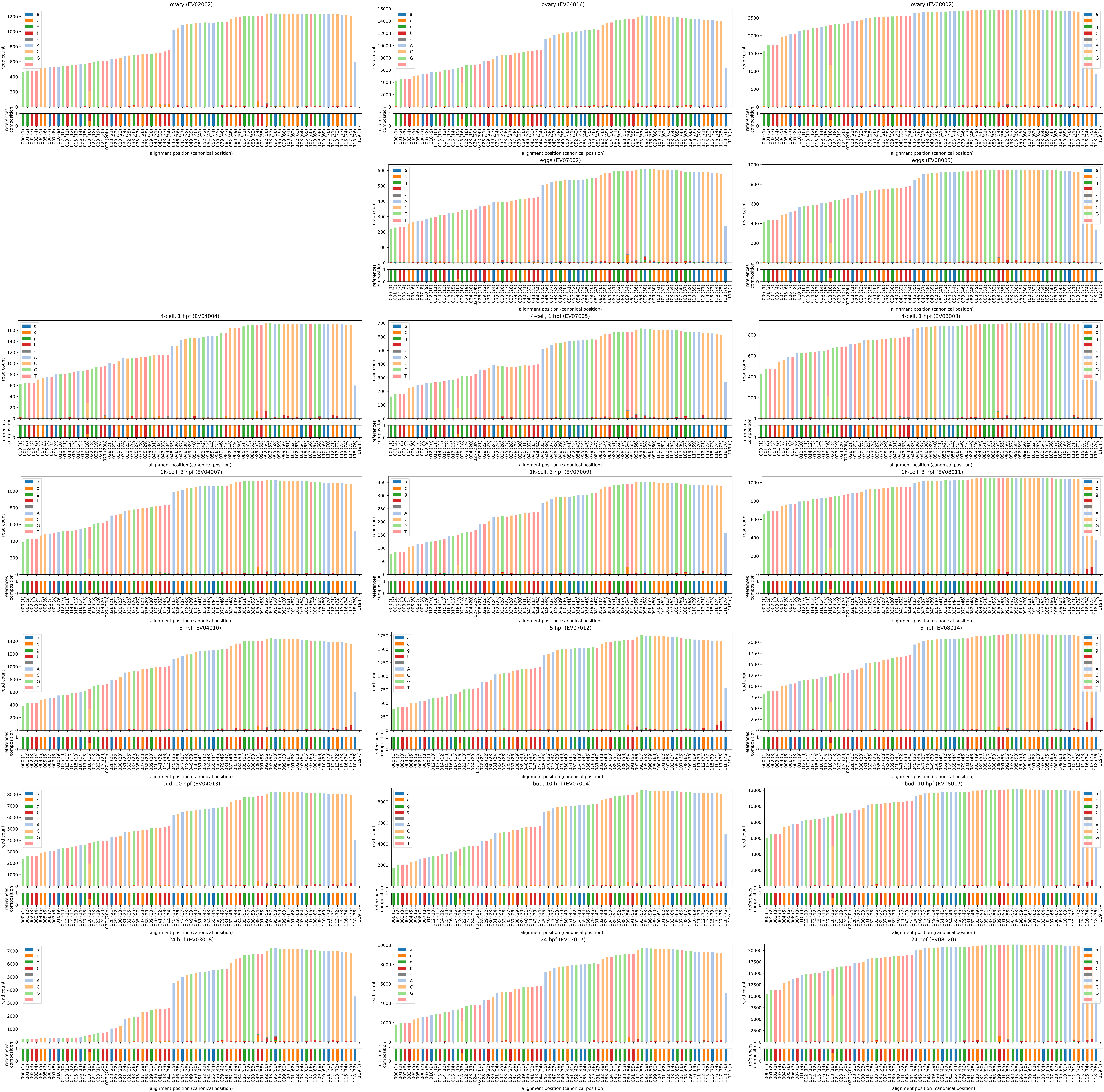

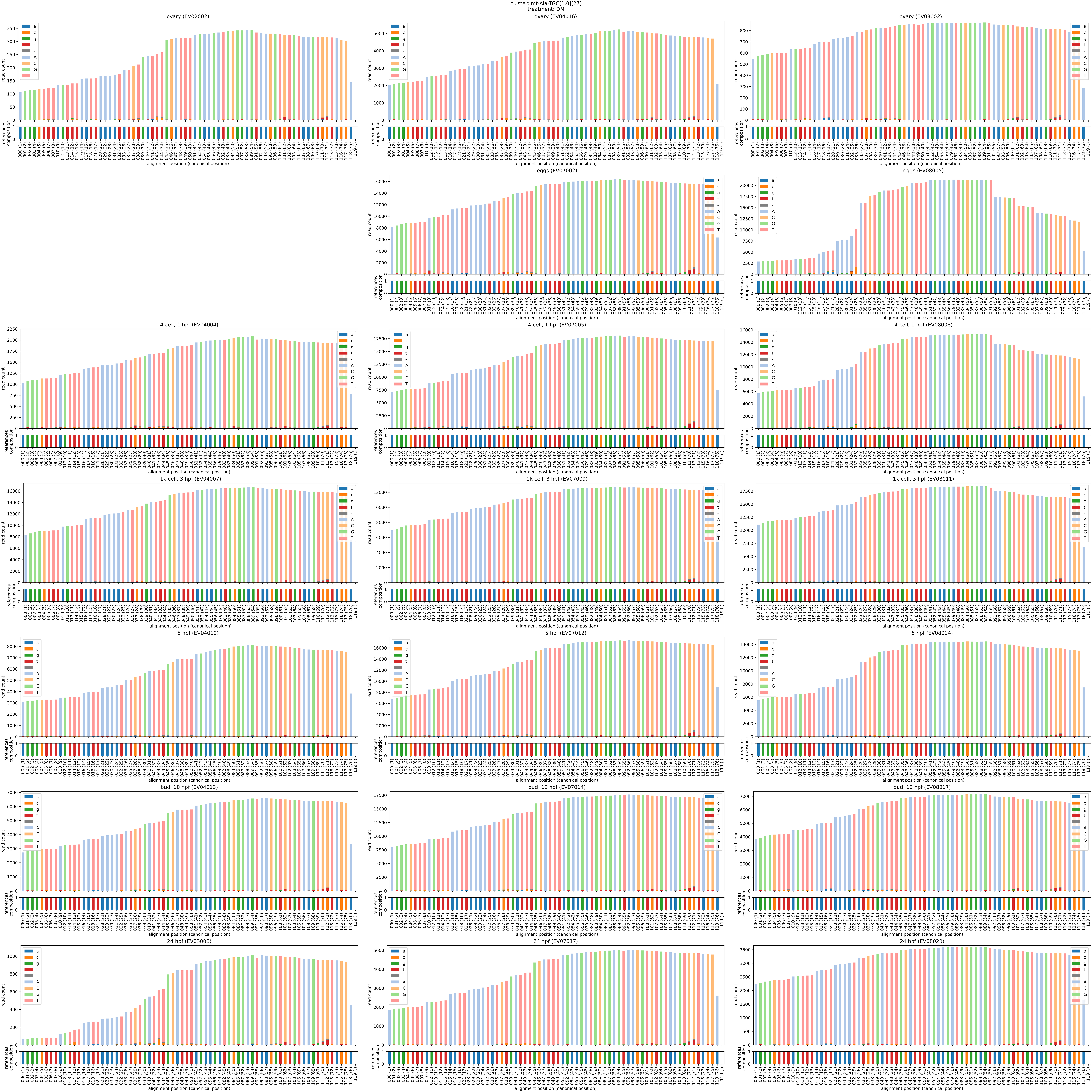

cluster: mt-Ala-TGC[1.0](27)
treatment: DM
ovary (EV02002)
ovary (EV04016)
ovary (EV08002)
eggs (EV07002)
eggs (EV08005)
4-cell, 1 hpf (EV04004)
4-cell, 1 hpf (EV07005)
4-cell, 1 hpf (EV08008)
1k-cell, 3 hpf (EV04007)
1k-cell, 3 hpf (EV07009)
1k-cell, 3 hpf (EV08011)
5 hpf (EV04010)
5 hpf (EV07012)
5 hpf (EV08014)
bud, 10 hpf (EV04013)
bud, 10 hpf (EV07014)
bud, 10 hpf (EV08017)
24 hpf (EV03008)
24 hpf (EV07017)
24 hpf (EV08020)
read count
references composition
alignment position (canonical position)
a
c
g
t
-
A
C
G
T

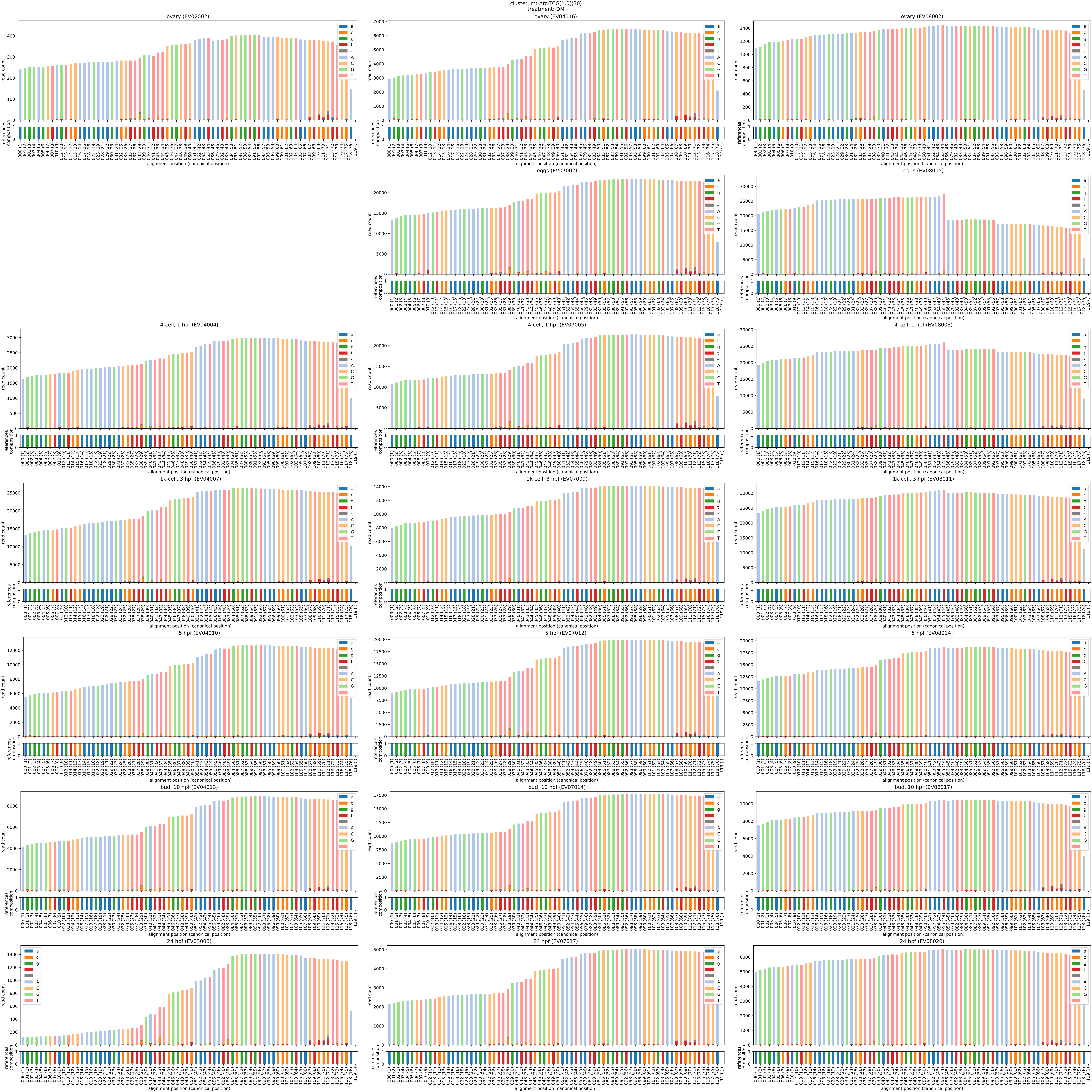
cluster: mt-Arg-TCG[1.0](30)
treatment: DM
ovary (EV02002)
ovary (EV04016)
ovary (EV08002)
eggs (EV07002)
eggs (EV08005)
4-cell, 1 hpf (EV04004)
4-cell, 1 hpf (EV07005)
4-cell, 1 hpf (EV08008)
1k-cell, 3 hpf (EV04007)
1k-cell, 3 hpf (EV07009)
1k-cell, 3 hpf (EV08011)
5 hpf (EV04010)
5 hpf (EV07012)
5 hpf (EV08014)
bud, 10 hpf (EV04013)
bud, 10 hpf (EV07014)
bud, 10 hpf (EV08017)
24 hpf (EV03008)
24 hpf (EV07017)
24 hpf (EV08020)
a
c
g
t
-
A
C
G
T
read count
references composition
alignment position (canonical position)

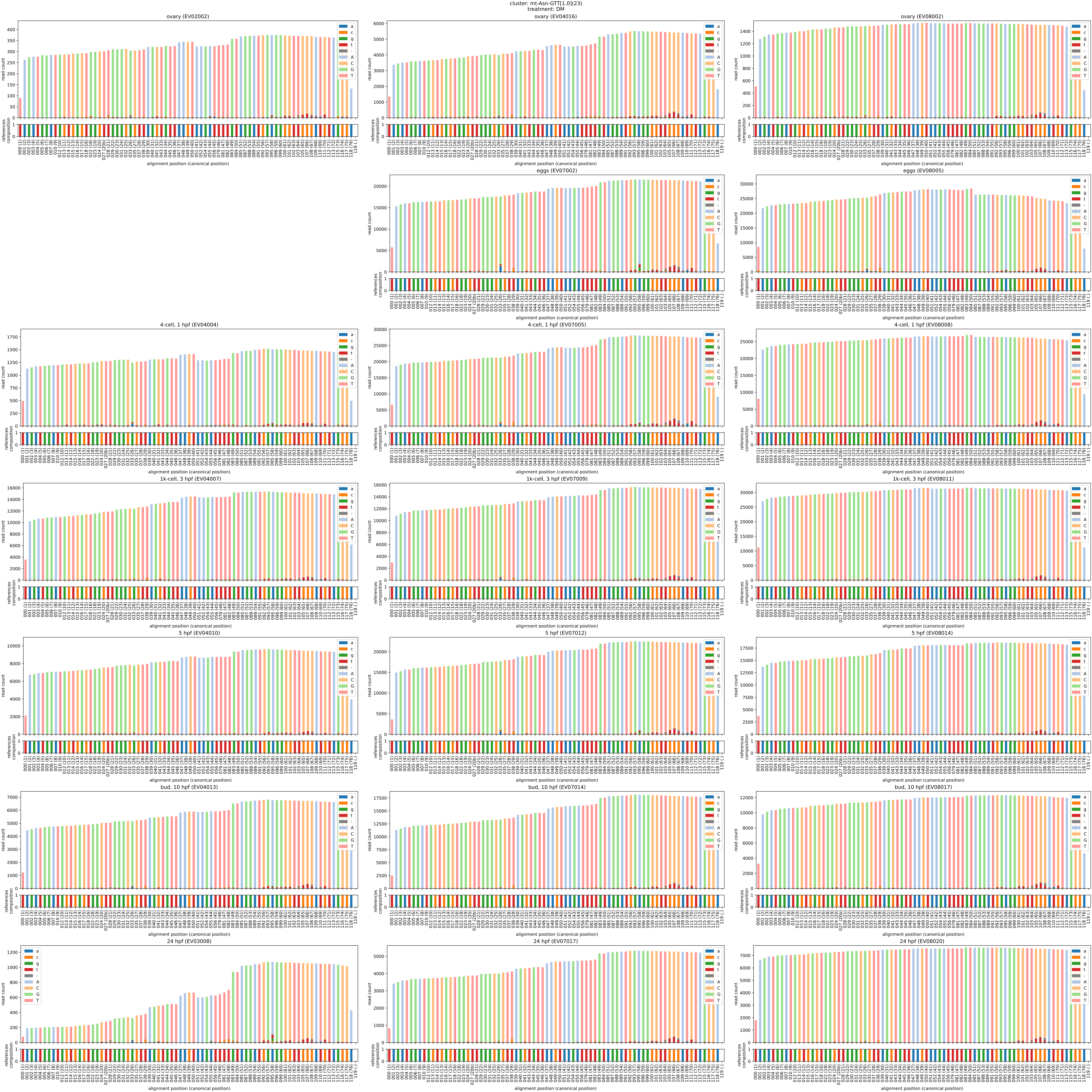

cluster: mt-Asn-GTT[1.0](23)
treatment: DM
ovary (EV02002)
ovary (EV04016)
ovary (EV08002)
eggs (EV07002)
eggs (EV08005)
4-cell, 1 hpf (EV04004)
4-cell, 1 hpf (EV07005)
4-cell, 1 hpf (EV08008)
1k-cell, 3 hpf (EV04007)
1k-cell, 3 hpf (EV07009)
1k-cell, 3 hpf (EV08011)
5 hpf (EV04010)
5 hpf (EV07012)
5 hpf (EV08014)
bud, 10 hpf (EV04013)
bud, 10 hpf (EV07014)
bud, 10 hpf (EV08017)
24 hpf (EV03008)
24 hpf (EV07017)
24 hpf (EV08020)
read count
references composition
alignment position (canonical position)
a
c
g
t
-
A
C
G
T

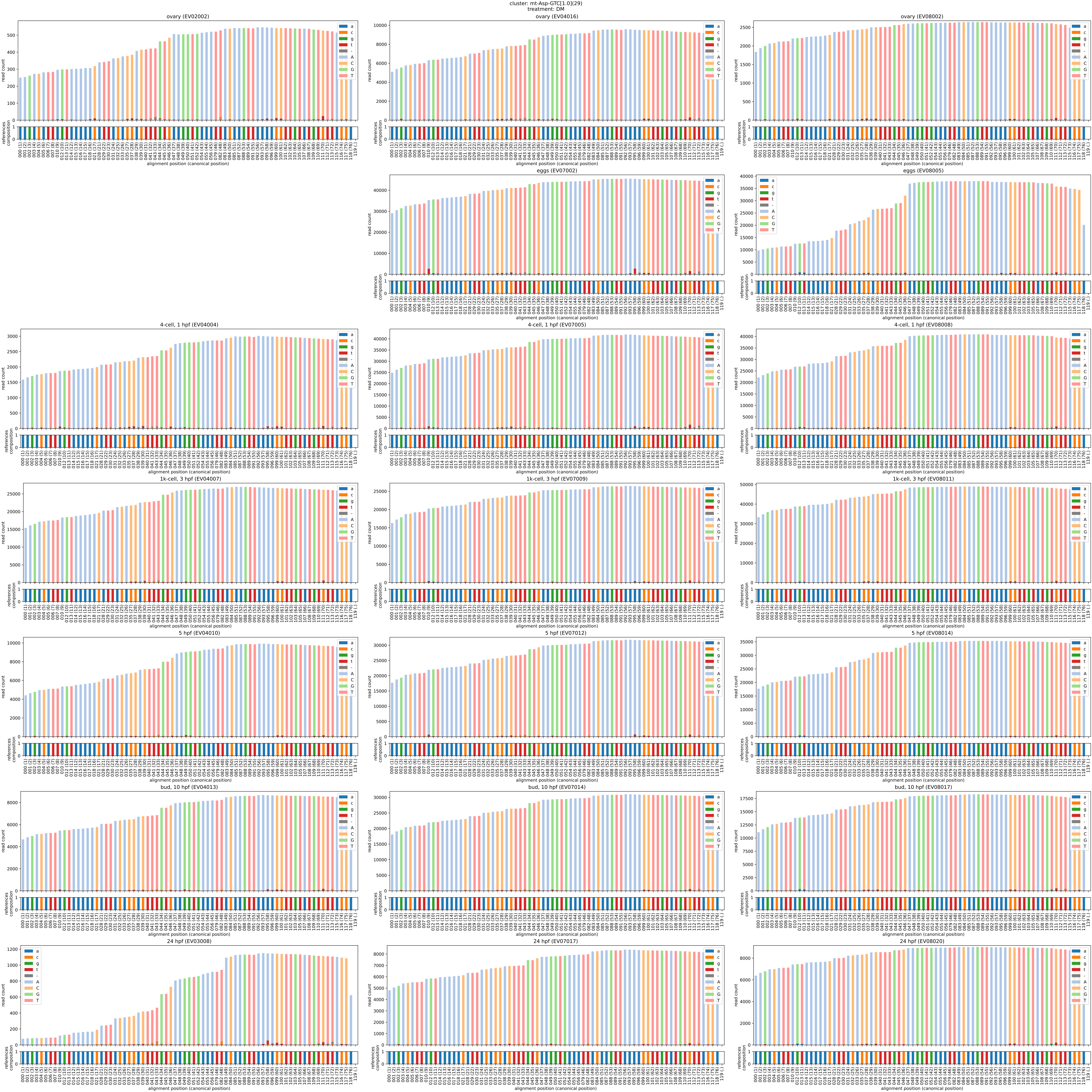
cluster: mt-Asp-GTC[1.0](29)
treatment: DM
ovary (EV02002)
ovary (EV04016)
ovary (EV08002)
eggs (EV07002)
eggs (EV08005)
4-cell, 1 hpf (EV04004)
4-cell, 1 hpf (EV07005)
4-cell, 1 hpf (EV08008)
1k-cell, 3 hpf (EV04007)
1k-cell, 3 hpf (EV07009)
1k-cell, 3 hpf (EV08011)
5 hpf (EV04010)
5 hpf (EV07012)
5 hpf (EV08014)
bud, 10 hpf (EV04013)
bud, 10 hpf (EV07014)
bud, 10 hpf (EV08017)
24 hpf (EV03008)
24 hpf (EV07017)
24 hpf (EV08020)
read count
references composition
alignment position (canonical position)
a
c
g
t
-
A
C
G
T

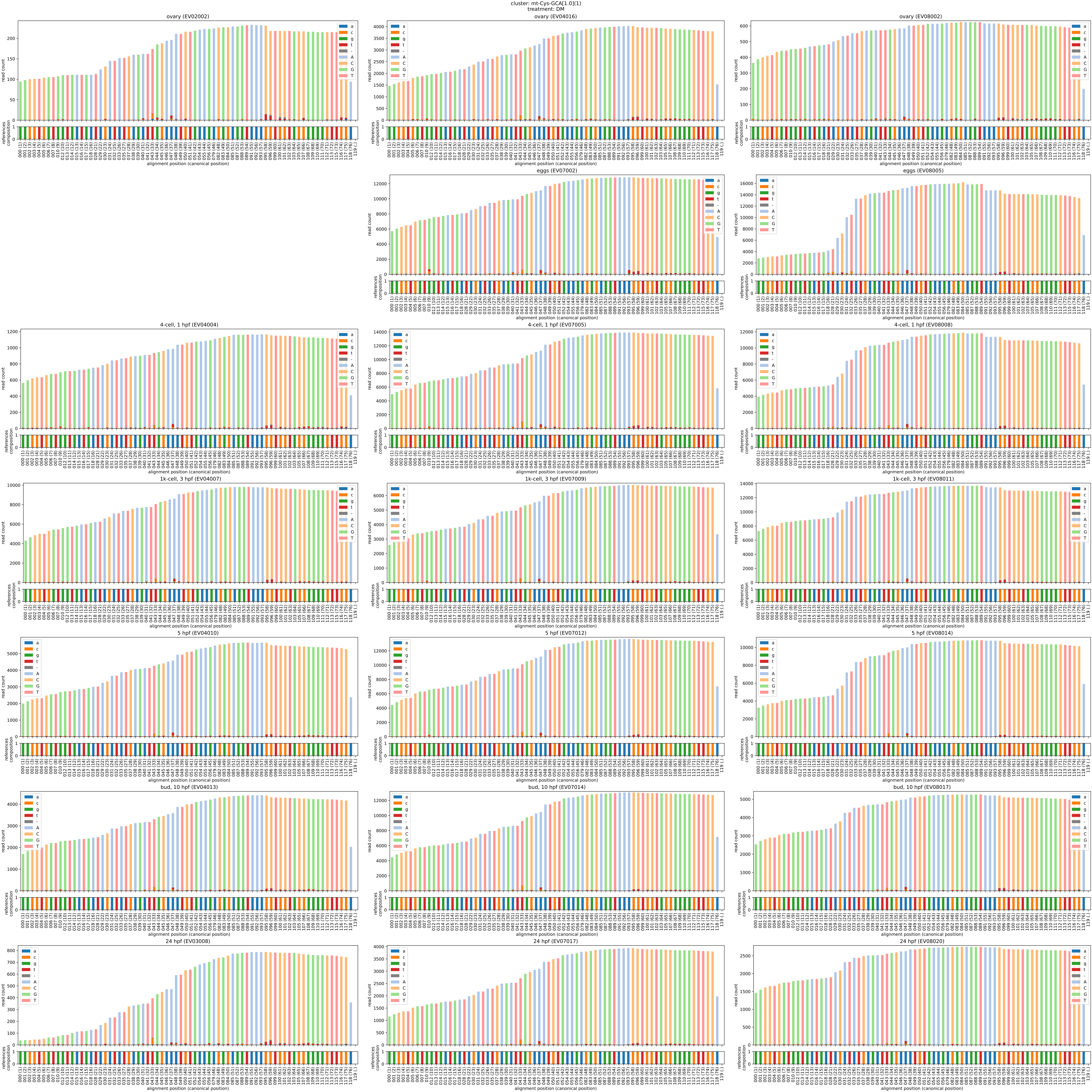
cluster: mt-Cys-GCA[1.0](1)
treatment: DM
ovary (EV02002)
ovary (EV04016)
ovary (EV08002)
eggs (EV07002)
eggs (EV08005)
4-cell, 1 hpf (EV04004)
4-cell, 1 hpf (EV07005)
4-cell, 1 hpf (EV08008)
1k-cell, 3 hpf (EV04007)
1k-cell, 3 hpf (EV07009)
1k-cell, 3 hpf (EV08011)
5 hpf (EV04010)
5 hpf (EV07012)
5 hpf (EV08014)
bud, 10 hpf (EV04013)
bud, 10 hpf (EV07014)
bud, 10 hpf (EV08017)
24 hpf (EV03008)
24 hpf (EV07017)
24 hpf (EV08020)
read count
references composition
alignment position (canonical position)
a
c
g
t
-
A
C
G
T

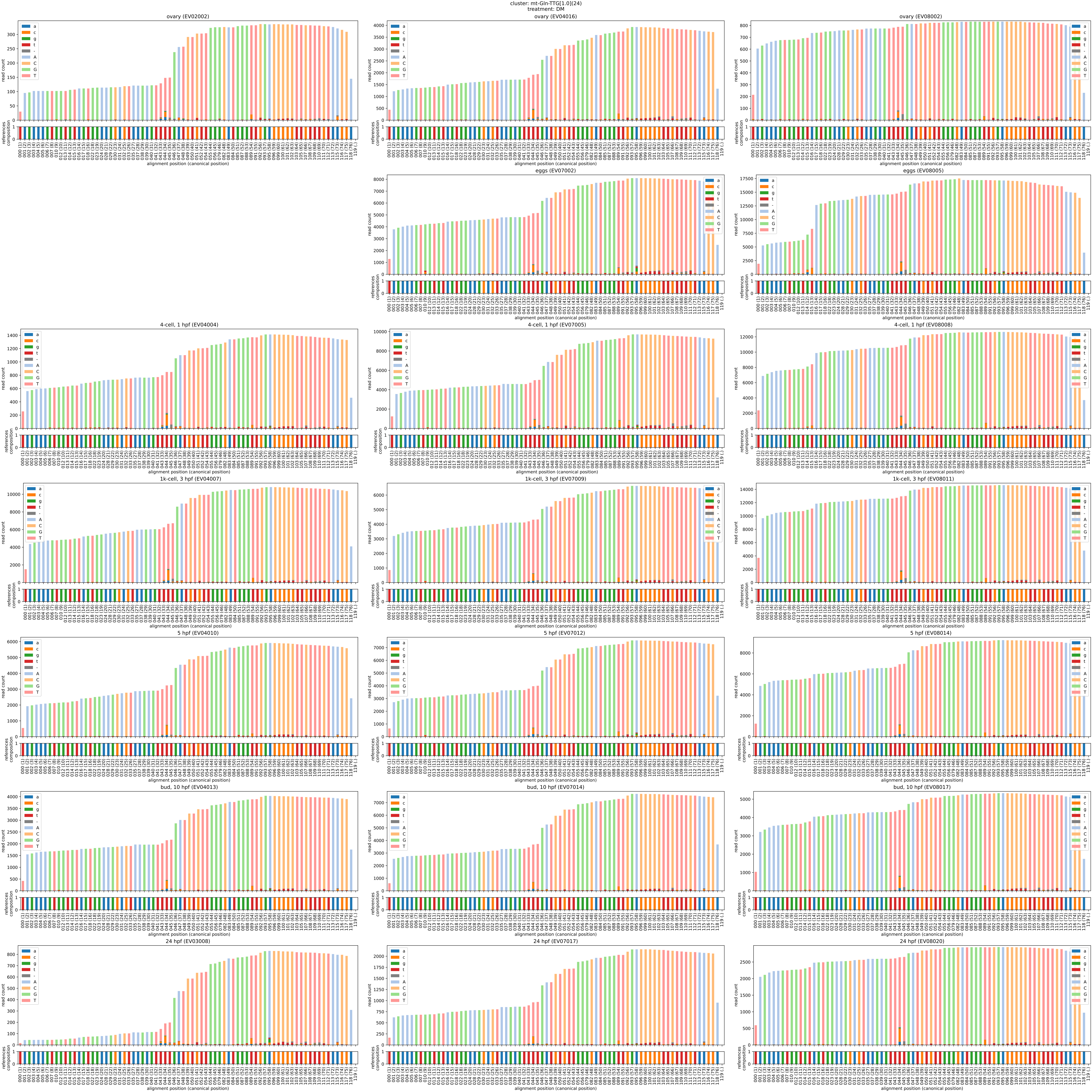

cluster: mt-Gln-TTG[1.0](24)
treatment: DM
ovary (EV02002)
ovary (EV04016)
ovary (EV08002)
eggs (EV07002)
eggs (EV08005)
4-cell, 1 hpf (EV04004)
4-cell, 1 hpf (EV07005)
4-cell, 1 hpf (EV08008)
1k-cell, 3 hpf (EV04007)
1k-cell, 3 hpf (EV07009)
1k-cell, 3 hpf (EV08011)
5 hpf (EV04010)
5 hpf (EV07012)
5 hpf (EV08014)
bud, 10 hpf (EV04013)
bud, 10 hpf (EV07014)
bud, 10 hpf (EV08017)
24 hpf (EV03008)
24 hpf (EV07017)
24 hpf (EV08020)
read count
references composition
alignment position (canonical position)
a
c
g
t
-
A
C
G
T

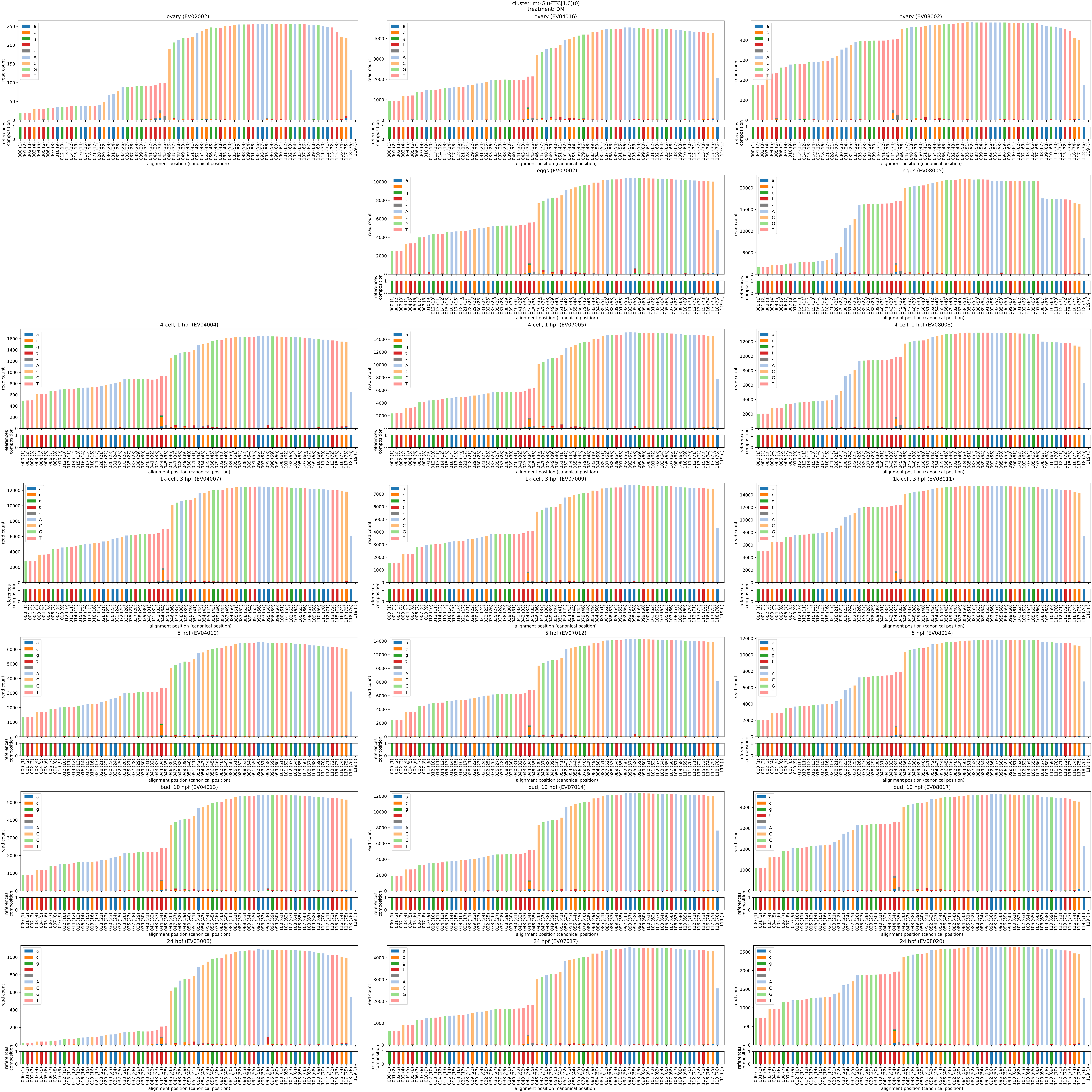
cluster: mt-Glu-TTC[1.0](0)
treatment: DM
ovary (EV02002)
ovary (EV04016)
ovary (EV08002)
eggs (EV07002)
eggs (EV08005)
4-cell, 1 hpf (EV04004)
4-cell, 1 hpf (EV07005)
4-cell, 1 hpf (EV08008)
1k-cell, 3 hpf (EV04007)
1k-cell, 3 hpf (EV07009)
1k-cell, 3 hpf (EV08011)
5 hpf (EV04010)
5 hpf (EV07012)
5 hpf (EV08014)
bud, 10 hpf (EV04013)
bud, 10 hpf (EV07014)
bud, 10 hpf (EV08017)
24 hpf (EV03008)
24 hpf (EV07017)
24 hpf (EV08020)
a
c
g
t
-
A
C
G
T
read count
references composition
alignment position (canonical position)

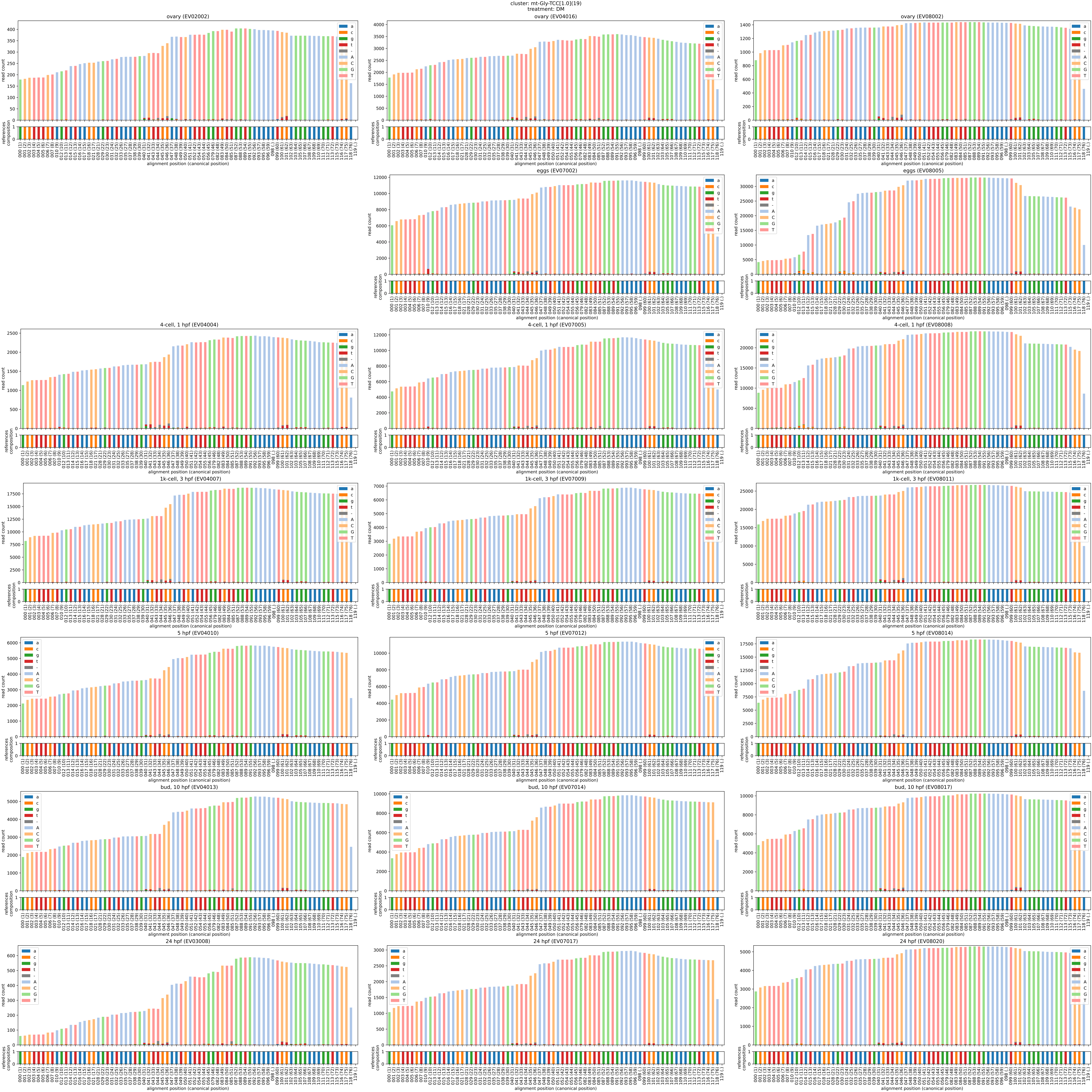
cluster: mt-Gly-TCC[1.0](19)
treatment: DM
ovary (EV02002)
ovary (EV04016)
ovary (EV08002)
eggs (EV07002)
eggs (EV08005)
4-cell, 1 hpf (EV04004)
4-cell, 1 hpf (EV07005)
4-cell, 1 hpf (EV08008)
1k-cell, 3 hpf (EV04007)
1k-cell, 3 hpf (EV07009)
1k-cell, 3 hpf (EV08011)
5 hpf (EV04010)
5 hpf (EV07012)
5 hpf (EV08014)
bud, 10 hpf (EV04013)
bud, 10 hpf (EV07014)
bud, 10 hpf (EV08017)
24 hpf (EV03008)
24 hpf (EV07017)
24 hpf (EV08020)
read count
references composition
alignment position (canonical position)
a
c
g
t
-
A
C
G
T

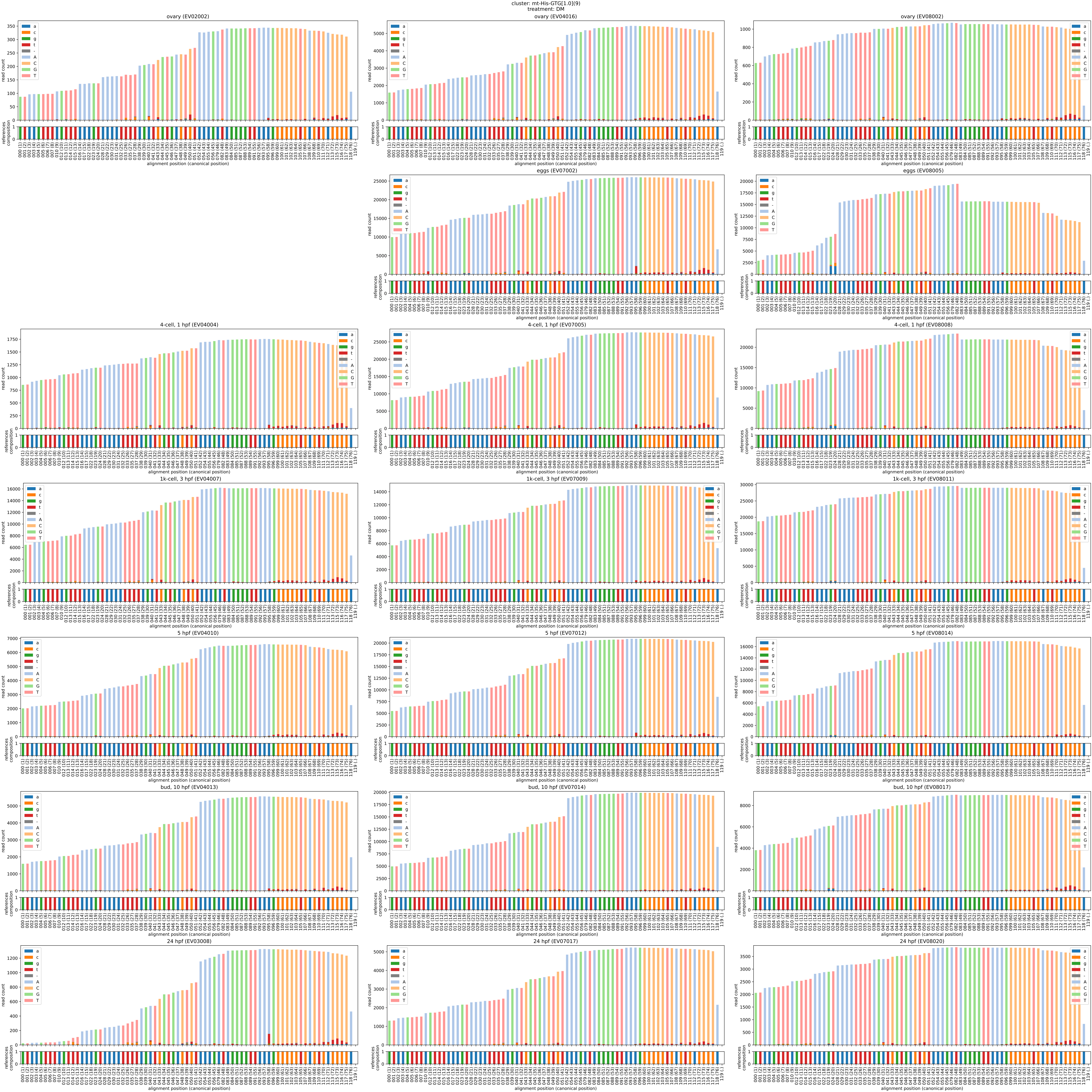
cluster: mt-His-GTG[1.0](9)
treatment: DM
ovary (EV02002)
ovary (EV04016)
ovary (EV08002)
eggs (EV07002)
eggs (EV08005)
4-cell, 1 hpf (EV04004)
4-cell, 1 hpf (EV07005)
4-cell, 1 hpf (EV08008)
1k-cell, 3 hpf (EV04007)
1k-cell, 3 hpf (EV07009)
1k-cell, 3 hpf (EV08011)
5 hpf (EV04010)
5 hpf (EV07012)
5 hpf (EV08014)
bud, 10 hpf (EV04013)
bud, 10 hpf (EV07014)
bud, 10 hpf (EV08017)
24 hpf (EV03008)
24 hpf (EV07017)
24 hpf (EV08020)
read count
references composition
alignment position (canonical position)
a
c
g
t
-
A
C
G
T

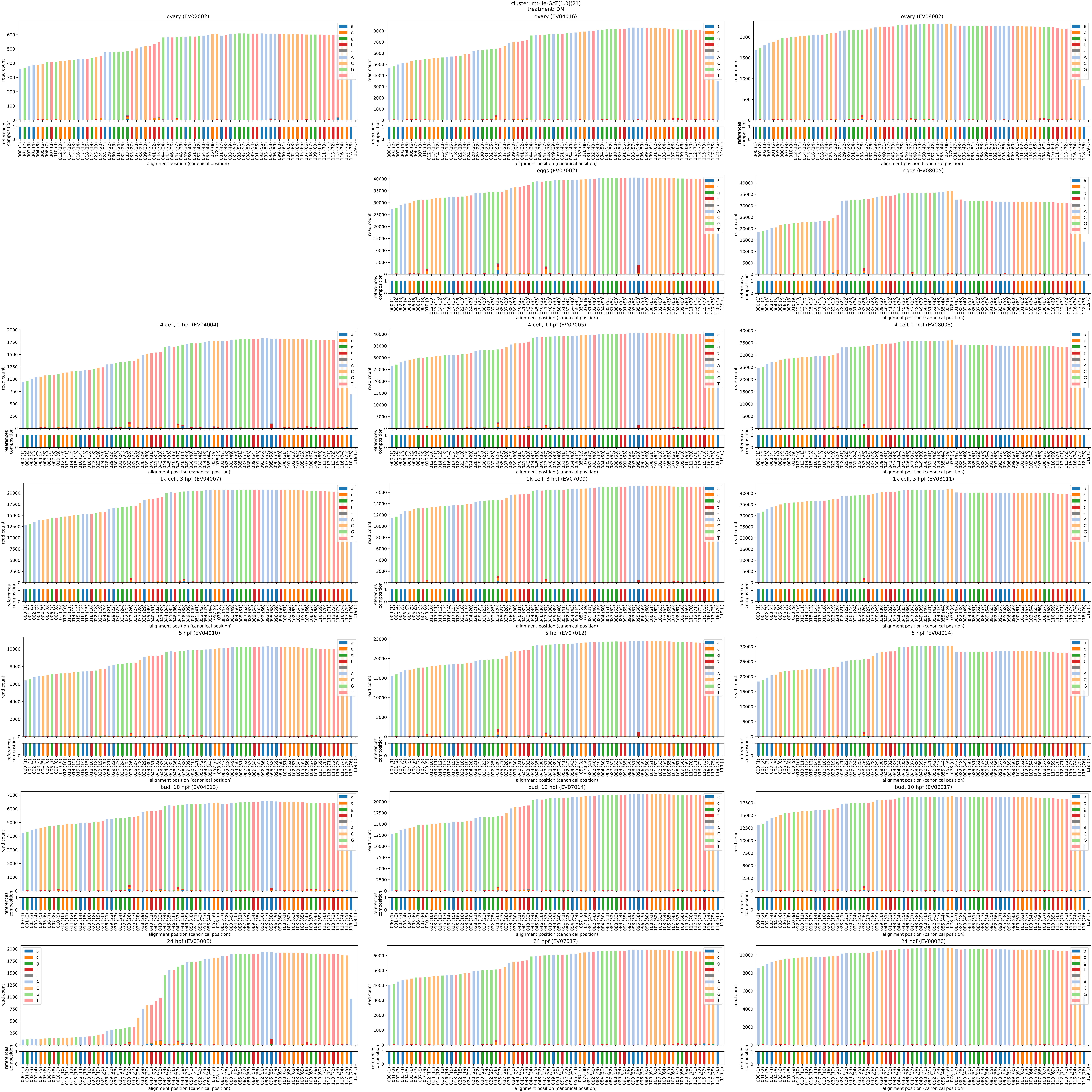

cluster: mt-Ile-GAT[1.0](21)
treatment: DM
ovary (EV02002)
ovary (EV04016)
ovary (EV08002)
eggs (EV07002)
eggs (EV08005)
4-cell, 1 hpf (EV04004)
4-cell, 1 hpf (EV07005)
4-cell, 1 hpf (EV08008)
1k-cell, 3 hpf (EV04007)
1k-cell, 3 hpf (EV07009)
1k-cell, 3 hpf (EV08011)
5 hpf (EV04010)
5 hpf (EV07012)
5 hpf (EV08014)
bud, 10 hpf (EV04013)
bud, 10 hpf (EV07014)
bud, 10 hpf (EV08017)
24 hpf (EV03008)
24 hpf (EV07017)
24 hpf (EV08020)
read count
references composition
alignment position (canonical position)
a
c
g
t
-
A
C
G
T

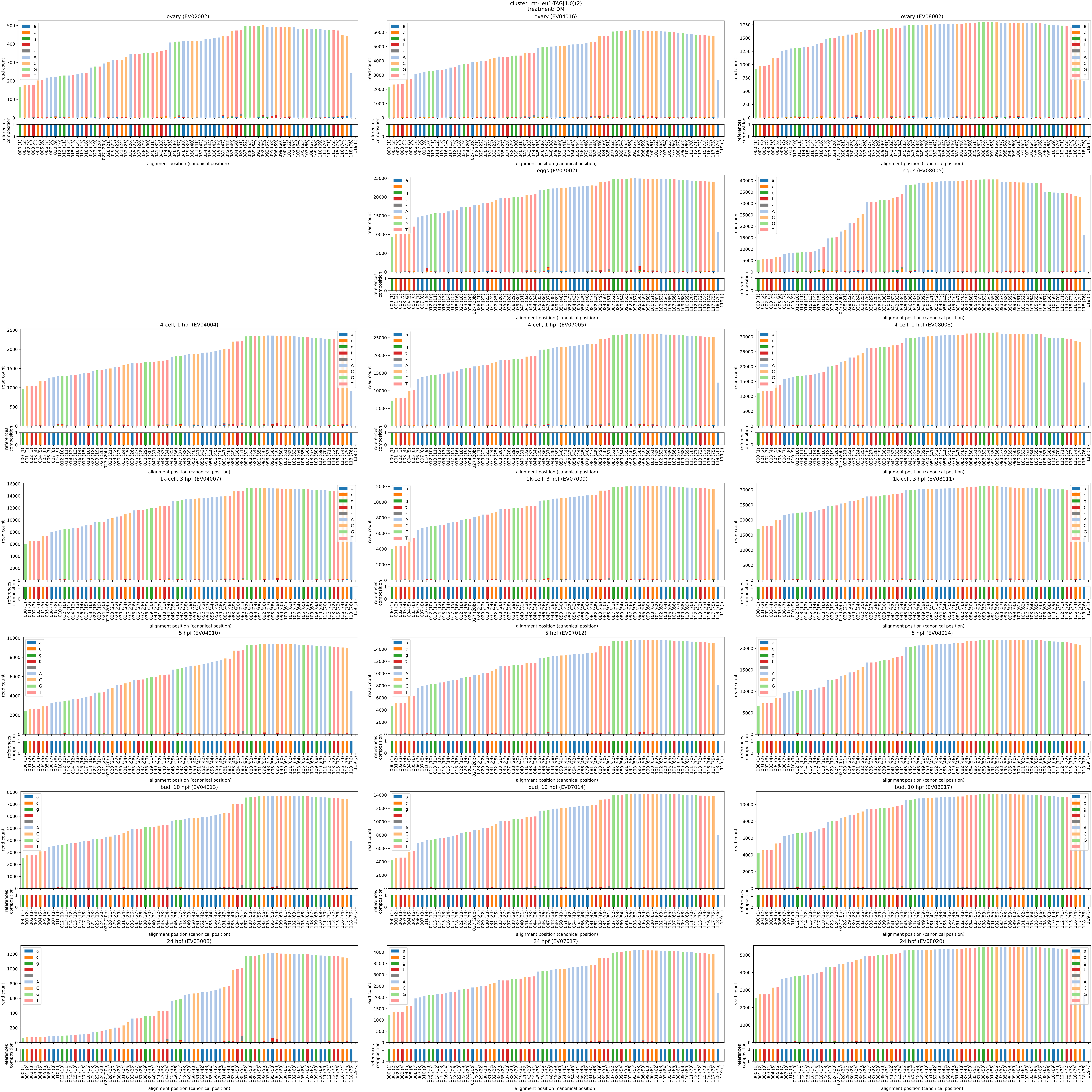
cluster: mt-Leu1-TAG[1.0](2)
treatment: DM
ovary (EV02002)
ovary (EV04016)
ovary (EV08002)
eggs (EV07002)
eggs (EV08005)
4-cell, 1 hpf (EV04004)
4-cell, 1 hpf (EV07005)
4-cell, 1 hpf (EV08008)
1k-cell, 3 hpf (EV04007)
1k-cell, 3 hpf (EV07009)
1k-cell, 3 hpf (EV08011)
5 hpf (EV04010)
5 hpf (EV07012)
5 hpf (EV08014)
bud, 10 hpf (EV04013)
bud, 10 hpf (EV07014)
bud, 10 hpf (EV08017)
24 hpf (EV03008)
24 hpf (EV07017)
24 hpf (EV08020)
read count
references composition
alignment position (canonical position)
a
c
g
t
-
A
C
G
T

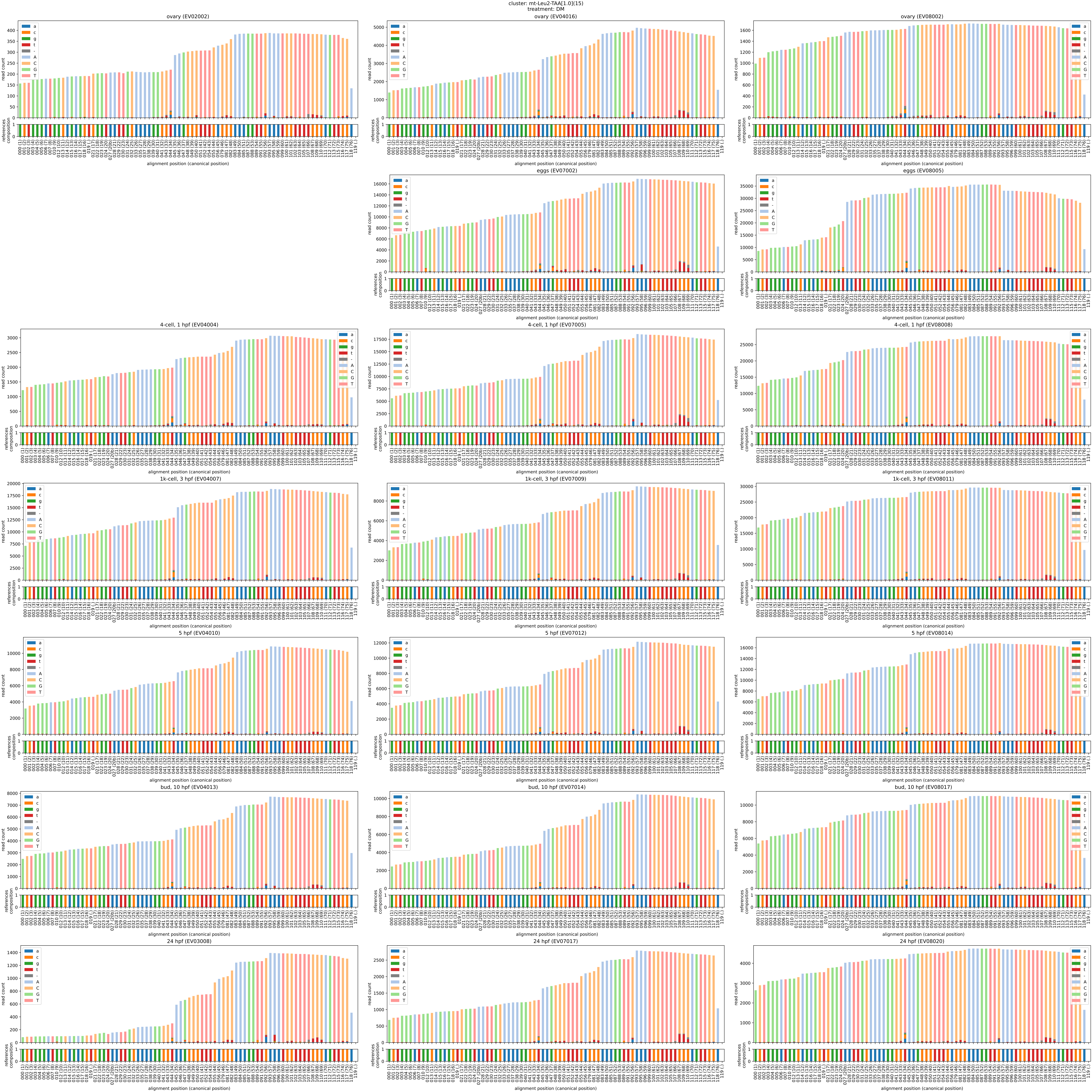
cluster: mt-Leu2-TAA[1.0](15)
treatment: DM
ovary (EV02002)
ovary (EV04016)
ovary (EV08002)
eggs (EV07002)
eggs (EV08005)
4-cell, 1 hpf (EV04004)
4-cell, 1 hpf (EV07005)
4-cell, 1 hpf (EV08008)
1k-cell, 3 hpf (EV04007)
1k-cell, 3 hpf (EV07009)
1k-cell, 3 hpf (EV08011)
5 hpf (EV04010)
5 hpf (EV07012)
5 hpf (EV08014)
bud, 10 hpf (EV04013)
bud, 10 hpf (EV07014)
bud, 10 hpf (EV08017)
24 hpf (EV03008)
24 hpf (EV07017)
24 hpf (EV08020)
read count
references composition
alignment position (canonical position)
a
c
g
t
-
A
C
G
T

cluster: mt-Lys-TTT[1.0](14)
treatment: DM

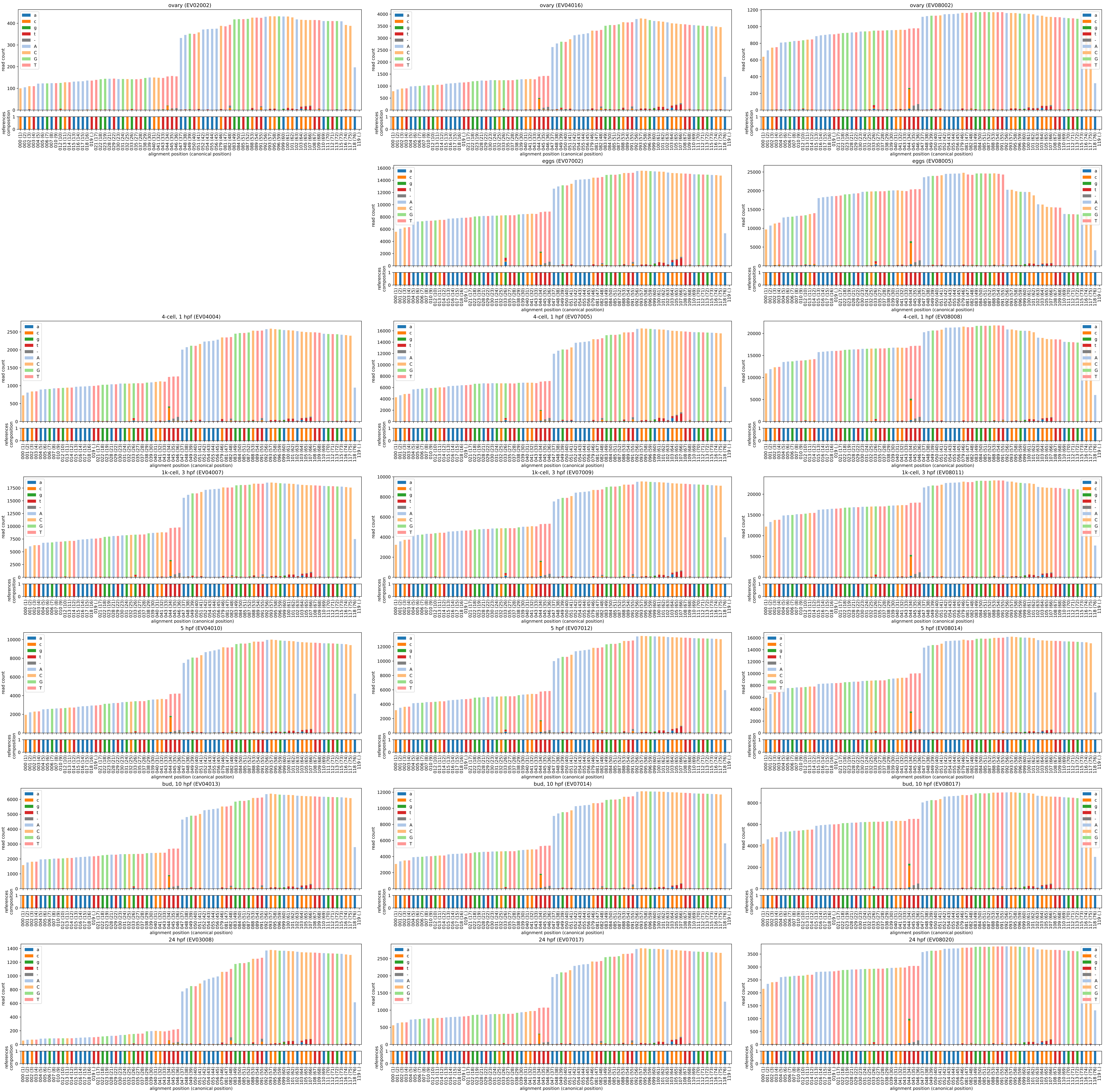

cluster: mt-Met-CAT[1.0](10)
treatment: DM

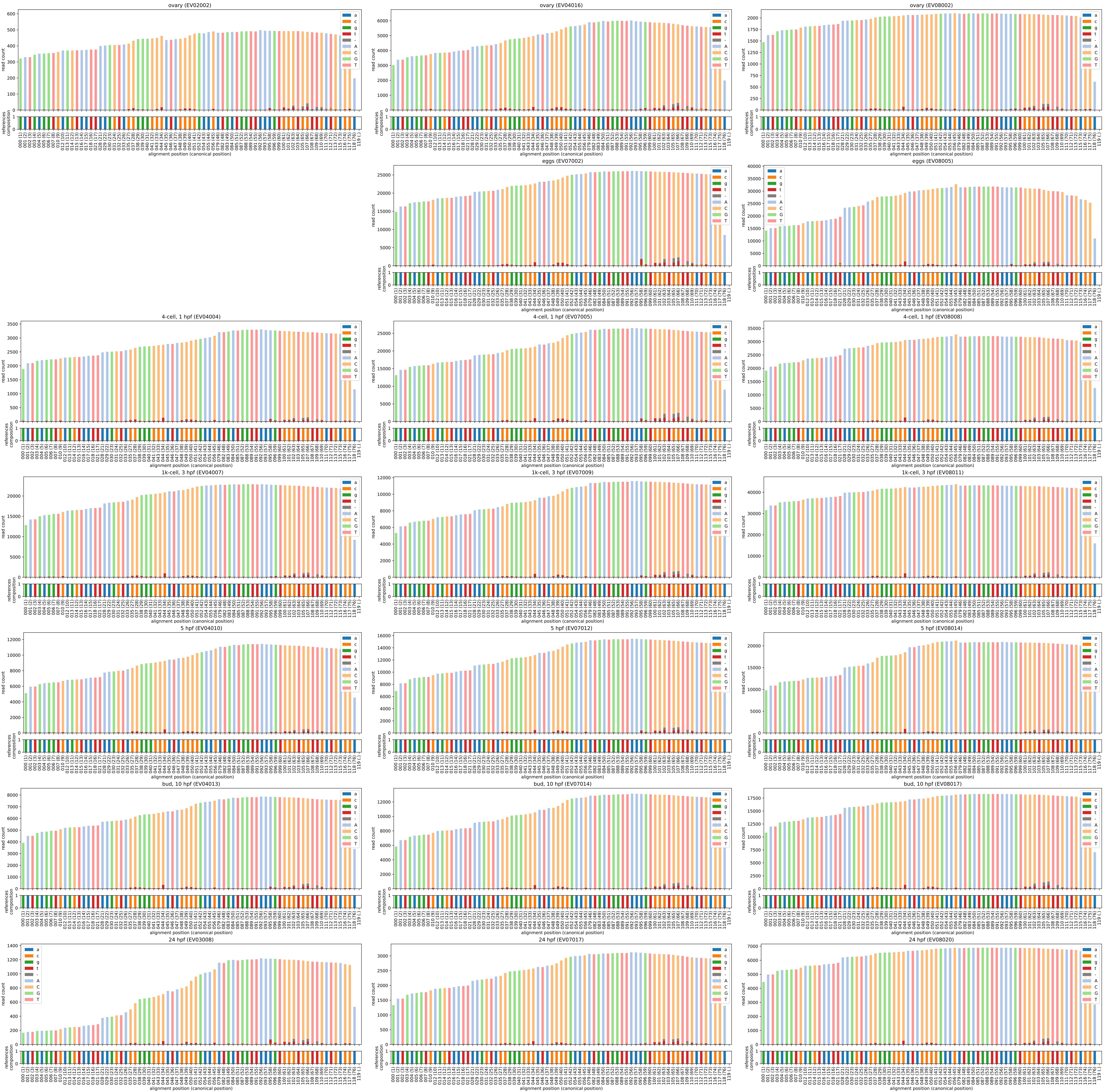

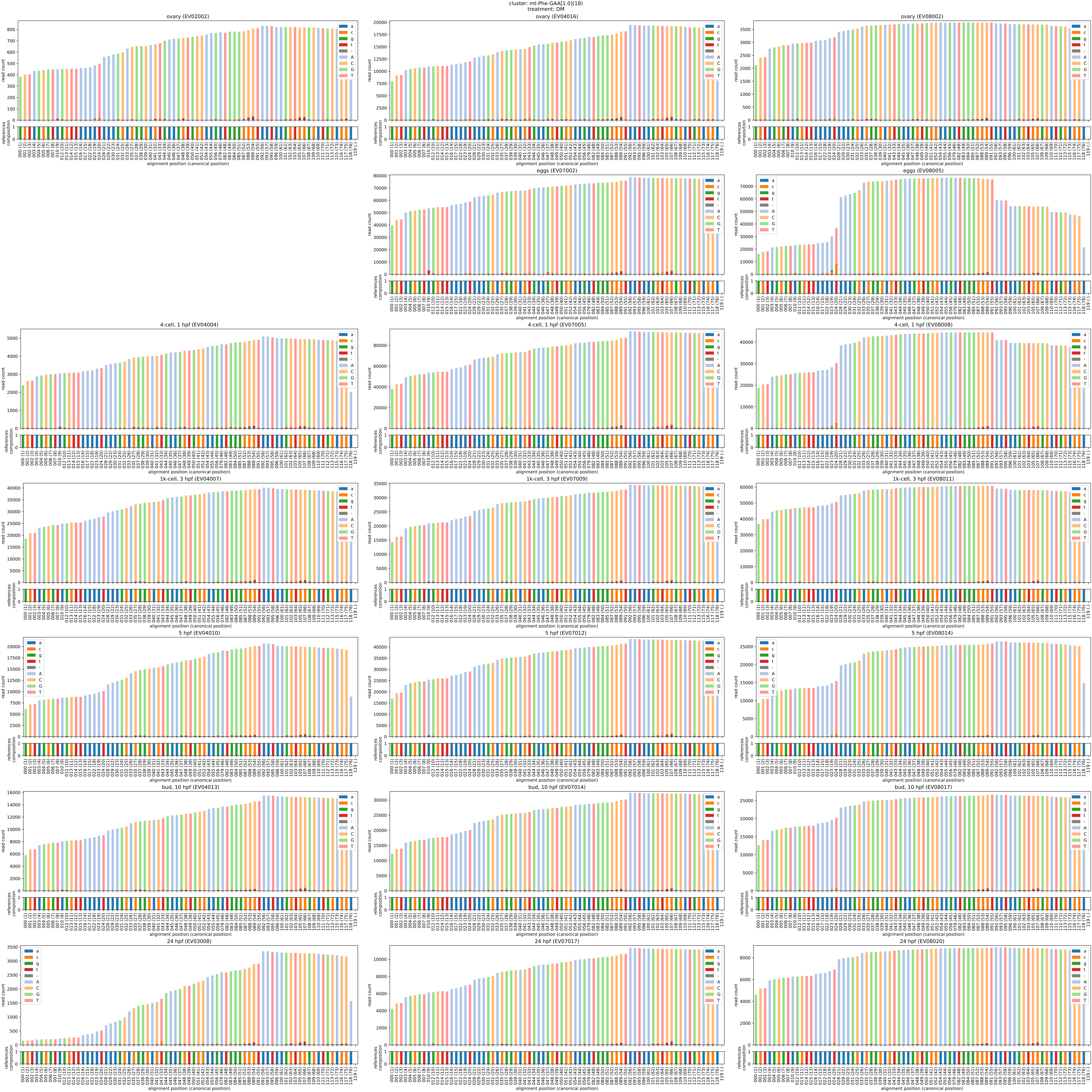

cluster: mt-Phe-GAA[1.0](18)
treatment: DM
ovary (EV02002)
ovary (EV04016)
ovary (EV08002)
eggs (EV07002)
eggs (EV08005)
4-cell, 1 hpf (EV04004)
4-cell, 1 hpf (EV07005)
4-cell, 1 hpf (EV08008)
1k-cell, 3 hpf (EV04007)
1k-cell, 3 hpf (EV07009)
1k-cell, 3 hpf (EV08011)
5 hpf (EV04010)
5 hpf (EV07012)
5 hpf (EV08014)
bud, 10 hpf (EV04013)
bud, 10 hpf (EV07014)
bud, 10 hpf (EV08017)
24 hpf (EV03008)
24 hpf (EV07017)
24 hpf (EV08020)
read count
references composition
alignment position (canonical position)
a
c
g
t
-
A
C
G
T

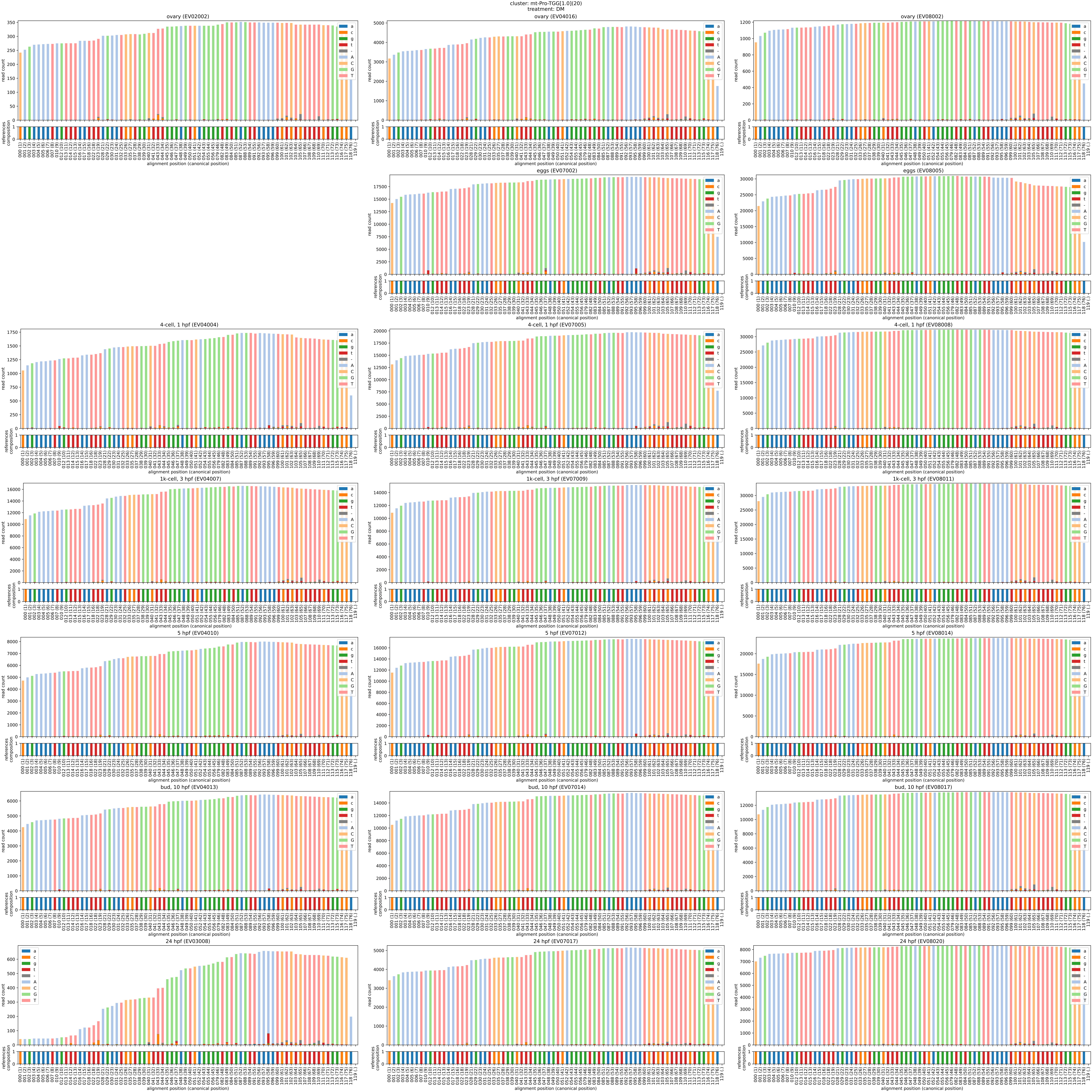
cluster: mt-Pro-TGG[1.0](20)
treatment: DM
ovary (EV02002)
ovary (EV04016)
ovary (EV08002)
eggs (EV07002)
eggs (EV08005)
4-cell, 1 hpf (EV04004)
4-cell, 1 hpf (EV07005)
4-cell, 1 hpf (EV08008)
1k-cell, 3 hpf (EV04007)
1k-cell, 3 hpf (EV07009)
1k-cell, 3 hpf (EV08011)
5 hpf (EV04010)
5 hpf (EV07012)
5 hpf (EV08014)
bud, 10 hpf (EV04013)
bud, 10 hpf (EV07014)
bud, 10 hpf (EV08017)
24 hpf (EV03008)
24 hpf (EV07017)
24 hpf (EV08020)
read count
references composition
alignment position (canonical position)
a
c
g
t
-
A
C
G
T

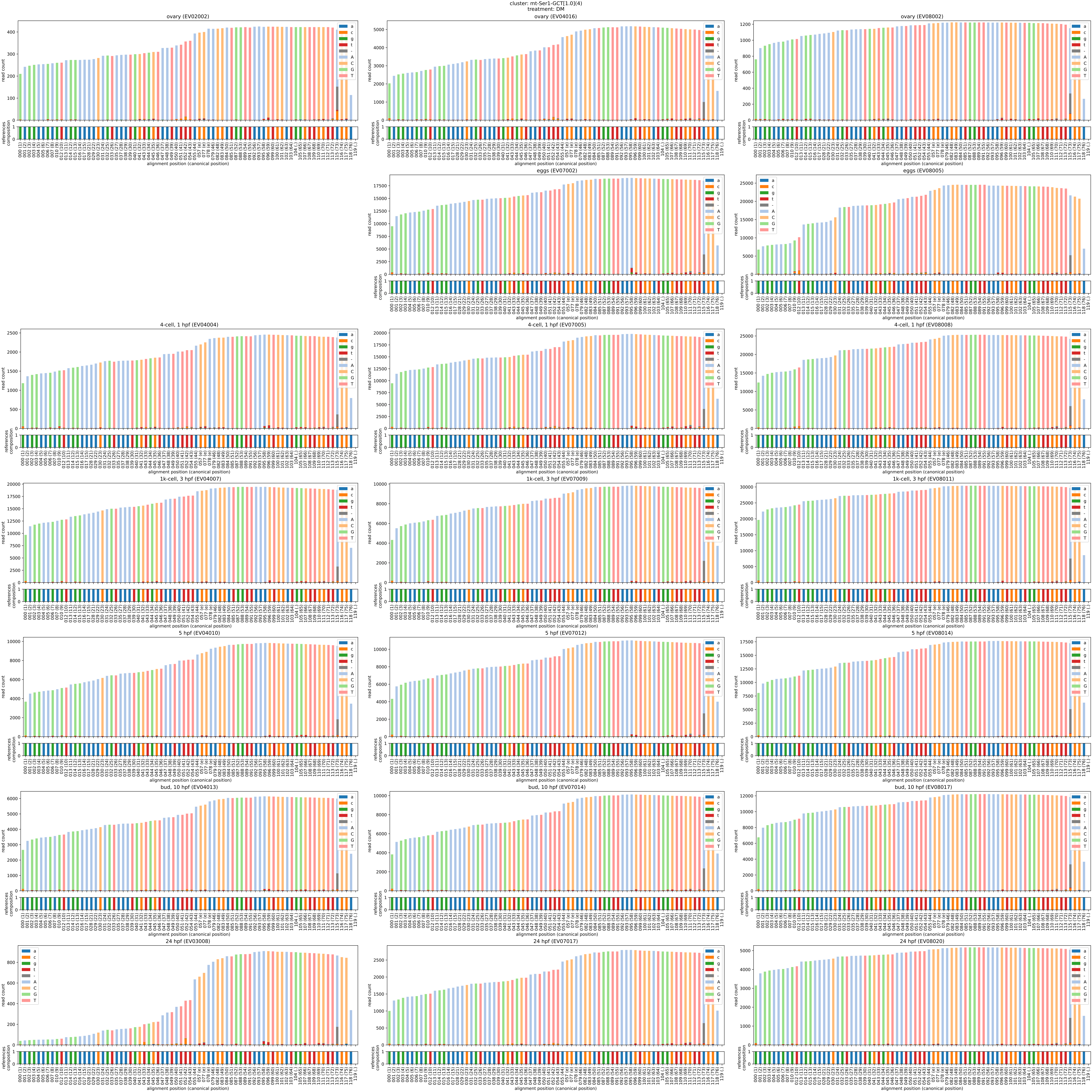
cluster: mt-Ser1-GCT[1.0](4)
treatment: DM
ovary (EV02002)
ovary (EV04016)
ovary (EV08002)
eggs (EV07002)
eggs (EV08005)
4-cell, 1 hpf (EV04004)
4-cell, 1 hpf (EV07005)
4-cell, 1 hpf (EV08008)
1k-cell, 3 hpf (EV04007)
1k-cell, 3 hpf (EV07009)
1k-cell, 3 hpf (EV08011)
5 hpf (EV04010)
5 hpf (EV07012)
5 hpf (EV08014)
bud, 10 hpf (EV04013)
bud, 10 hpf (EV07014)
bud, 10 hpf (EV08017)
24 hpf (EV03008)
24 hpf (EV07017)
24 hpf (EV08020)
read count
references composition
alignment position (canonical position)
a
c
g
t
-
A
C
G
T

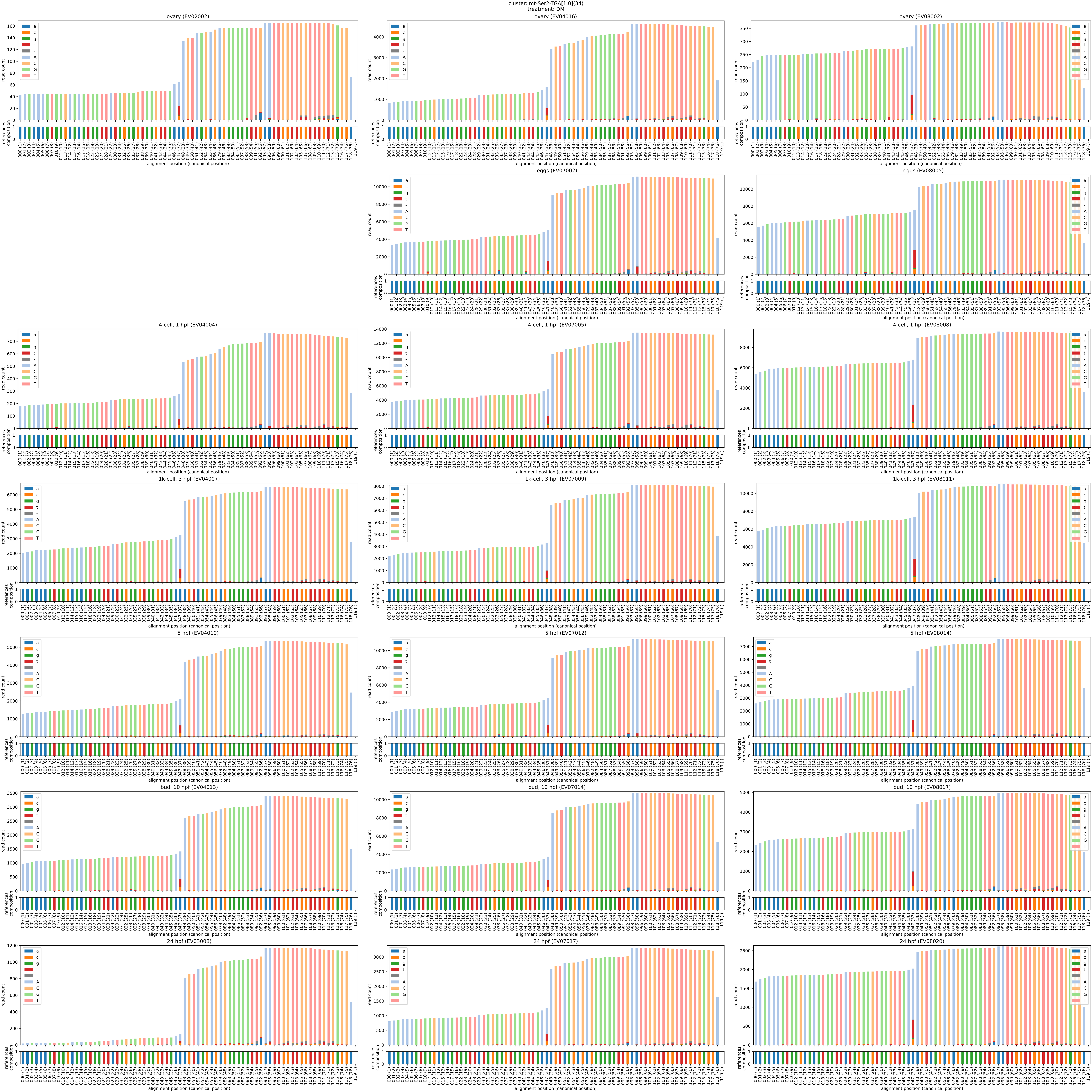

cluster: mt-Ser2-TGA[1.0](34)
treatment: DM
ovary (EV02002)
ovary (EV04016)
ovary (EV08002)
eggs (EV07002)
eggs (EV08005)
4-cell, 1 hpf (EV04004)
4-cell, 1 hpf (EV07005)
4-cell, 1 hpf (EV08008)
1k-cell, 3 hpf (EV04007)
1k-cell, 3 hpf (EV07009)
1k-cell, 3 hpf (EV08011)
5 hpf (EV04010)
5 hpf (EV07012)
5 hpf (EV08014)
bud, 10 hpf (EV04013)
bud, 10 hpf (EV07014)
bud, 10 hpf (EV08017)
24 hpf (EV03008)
24 hpf (EV07017)
24 hpf (EV08020)
read count
references composition
alignment position (canonical position)
a
c
g
t
-
A
C
G
T

cluster: mt-Thr-TGT[1.0](48)
treatment: DM

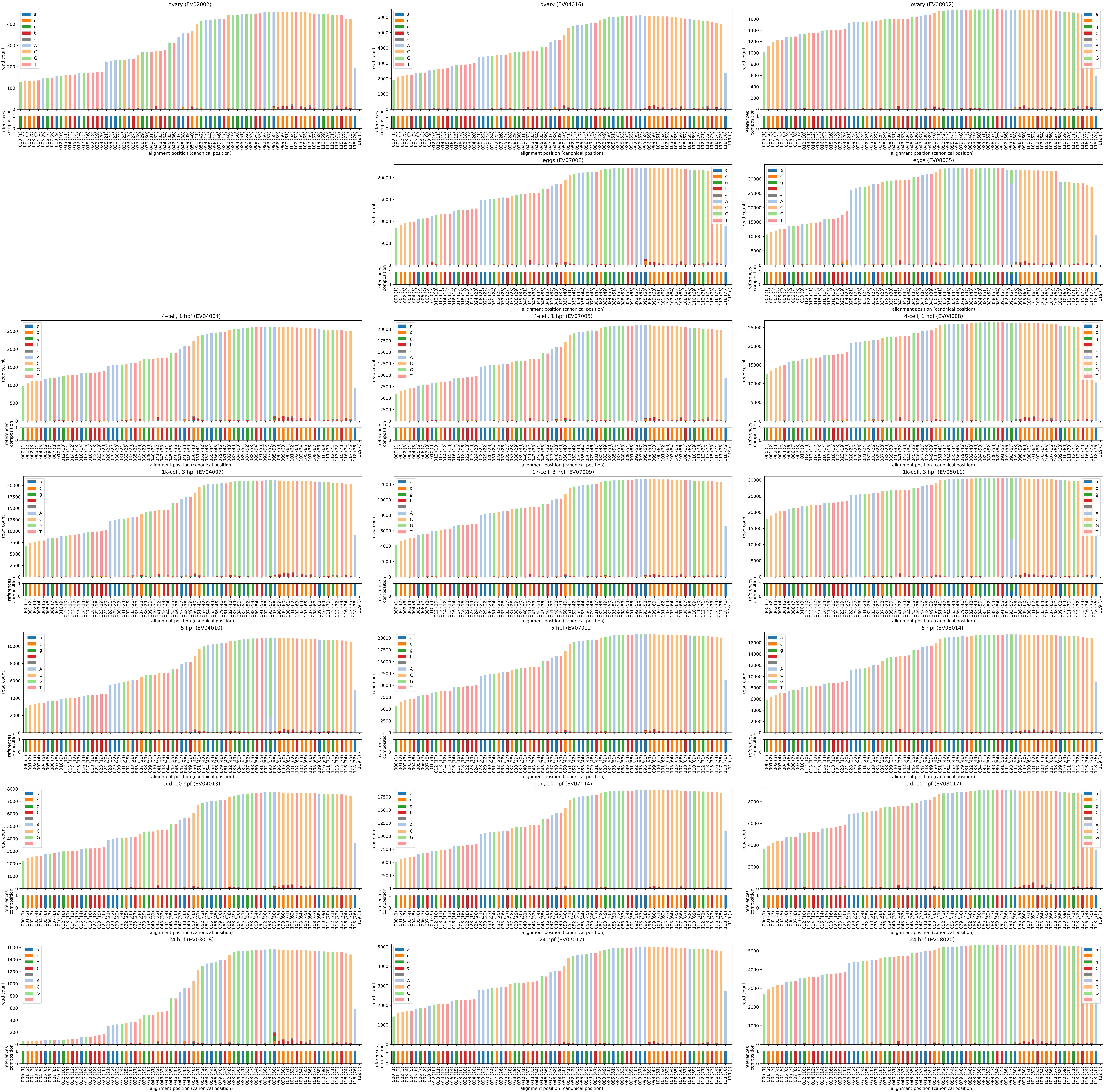

cluster: mt-Trp-TCA[1.0](22)
treatment: DM

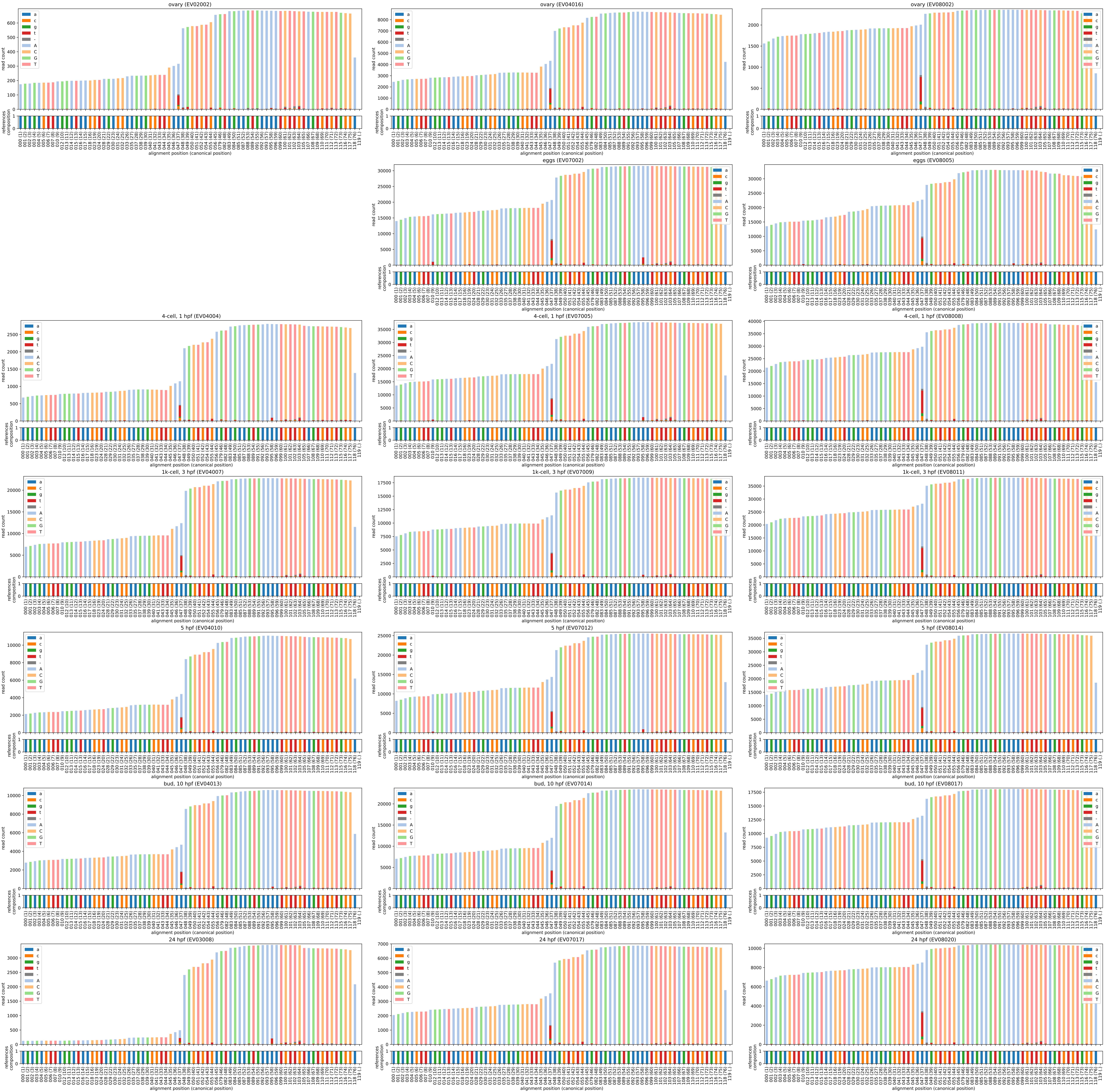

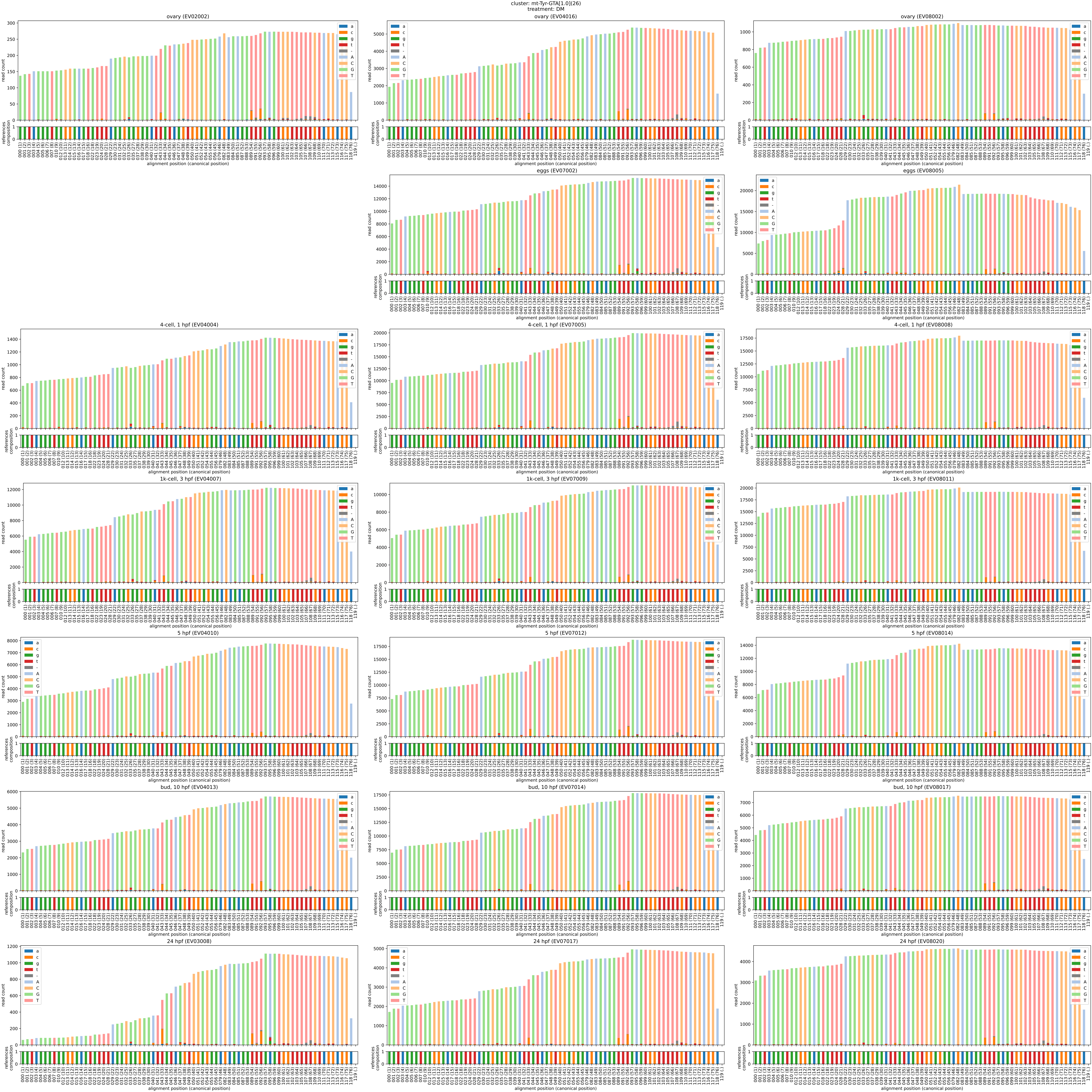
cluster: mt-Tyr-GTA[1.0](26)
treatment: DM
ovary (EV02002)
ovary (EV04016)
ovary (EV08002)
eggs (EV07002)
eggs (EV08005)
4-cell, 1 hpf (EV04004)
4-cell, 1 hpf (EV07005)
4-cell, 1 hpf (EV08008)
1k-cell, 3 hpf (EV04007)
1k-cell, 3 hpf (EV07009)
1k-cell, 3 hpf (EV08011)
5 hpf (EV04010)
5 hpf (EV07012)
5 hpf (EV08014)
bud, 10 hpf (EV04013)
bud, 10 hpf (EV07014)
bud, 10 hpf (EV08017)
24 hpf (EV03008)
24 hpf (EV07017)
24 hpf (EV08020)
read count
references composition
alignment position (canonical position)
a
c
g
t
-
A
C
G
T

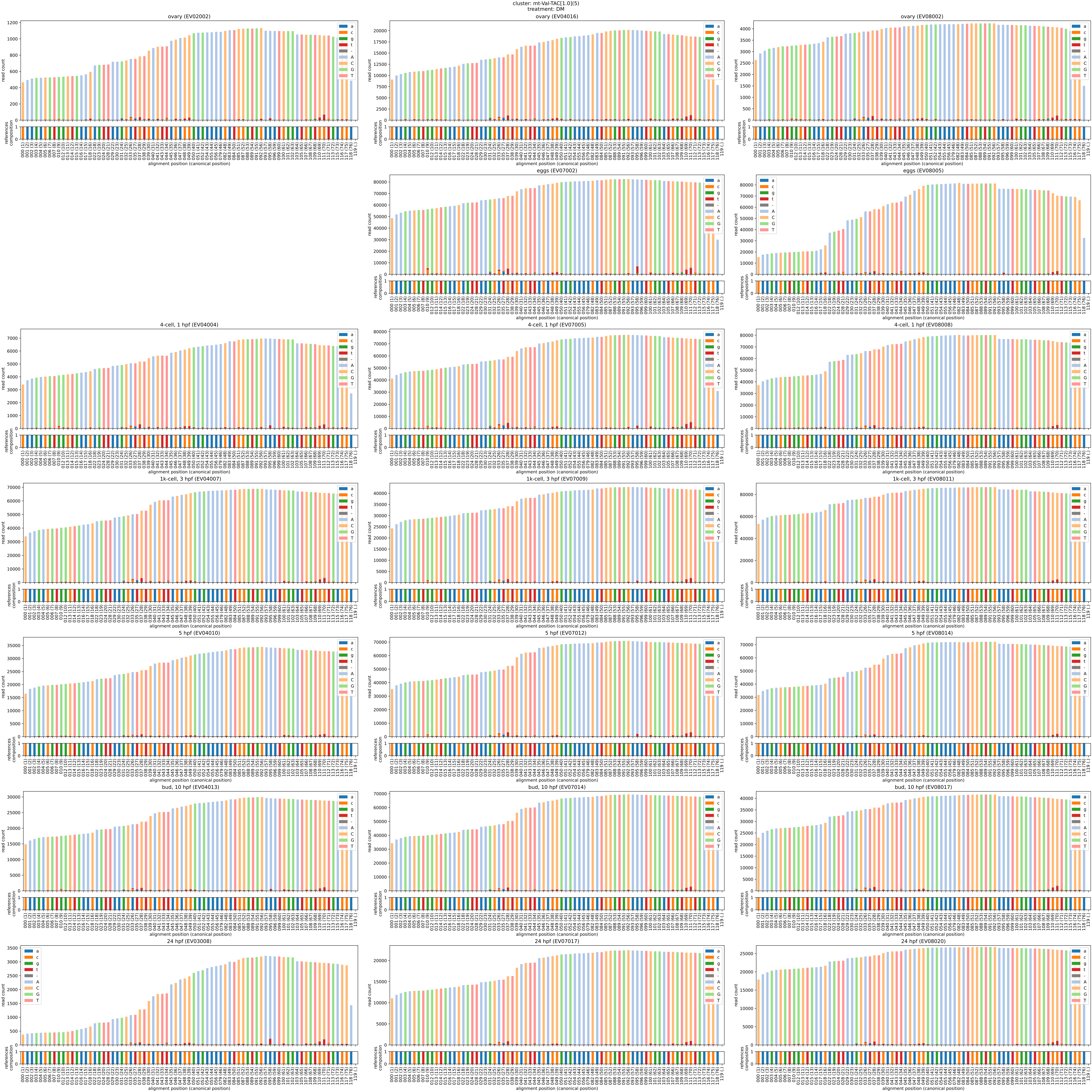
cluster: mt-Val-TAC[1.0](5)
treatment: DM
ovary (EV02002)
ovary (EV04016)
ovary (EV08002)
eggs (EV07002)
eggs (EV08005)
4-cell, 1 hpf (EV04004)
4-cell, 1 hpf (EV07005)
4-cell, 1 hpf (EV08008)
1k-cell, 3 hpf (EV04007)
1k-cell, 3 hpf (EV07009)
1k-cell, 3 hpf (EV08011)
5 hpf (EV04010)
5 hpf (EV07012)
5 hpf (EV08014)
bud, 10 hpf (EV04013)
bud, 10 hpf (EV07014)
bud, 10 hpf (EV08017)
24 hpf (EV03008)
24 hpf (EV07017)
24 hpf (EV08020)
read count
references composition
alignment position (canonical position)
a
c
g
t
-
A
C
G
T
